# Orthogonal Site-Specific Dual Bioconjugation of Aryl and Alkyl Thiols

**DOI:** 10.1021/jacs.5c02981

**Published:** 2025-05-05

**Authors:** Mark A. R. de Geus, Christian E. Stieger, Jan Vincent V. Arafiles, Jean-Romain P. J. Lotthé, Peter Schmieder, Kristin Kemnitz-Hassanin, Beate Kindt, Heinrich Leonhardt, Saskia Schmitt, Marcus Gerlach, Dominik Schumacher, Jonas Helma, Marc-André Kasper, Christian P. R. Hackenberger

**Affiliations:** † 28417 Leibniz-Forschungsinstitut für Molekulare Pharmakologie (FMP), Robert-Rössle-Straβe 10, Berlin 13125, Germany; § Department of Chemistry, Humboldt Universität zu Berlin, Brook-Taylor-Straβe 2, Berlin 12489, Germany; ∥ Faculty of Biology and Center for Molecular Biosystems (BioSysM), Human Biology and BioImaging, Ludwig-Maximilians-Universität München, Butenandtstraβe 1, Munich 81377, Germany; ‡ 689670 Tubulis GmbH, Am Klopferspitz 19a, Planegg-Martinsried, Munich, 82152 Germany

## Abstract

We introduce aryl thiols as nucleophiles for site-specific protein and antibody bioconjugation, which allows the orthogonal labeling of native cysteines for double modification strategies. In a high-yielding synthesis, we introduce aromatic thiol substituents in two amino acids (4-SH-*L*-Phe and 3-SH-*L*-Tyr), which can be site-specifically incorporated into the *C*-terminus of a protein using the enzyme *tubulin tyrosine ligase* (TTL, Tub-tag labeling). In particular, we found that 3-SH-*L*-tyrosine shows excellent water solubility and incorporation rates, similar to previously described Tyr-derivatives. 2D NMR experiments revealed a p*K*_a_ value of 5.5 for the aryl thiol modality of 3-SH-*L*-tyrosine, which matches the pH-dependent reactivity profile toward thiol-selective ethynyl-triazolyl-phosphinate (ETP) electrophiles. Most importantly, we found that the addition of glutathione had no significant effect on the reaction between ETPs and the aryl thiol at pH 7.0 and below, supporting orthogonal reactivity between the aryl and alkyl thiols. We utilized these findings to develop an orthogonal thiol-selective dual bioconjugation protocol for proteins, featuring TTL-ligation to site-specifically incorporate the arylthiol-containing amino acid derivative, followed by aryl thiolate functionalization at pH 5.5 and subsequent conjugation of cysteines at pH 8.3. This dual bioconjugation strategy was used to generate a highly fluorescent photostabilized nanobody and a fully functionalized antibody-drug conjugate carrying two different cytotoxic payloads, which displays potent cytotoxicity toward cells carrying the target antigen in addition to a strong bystander effect.

## Graphical abstract

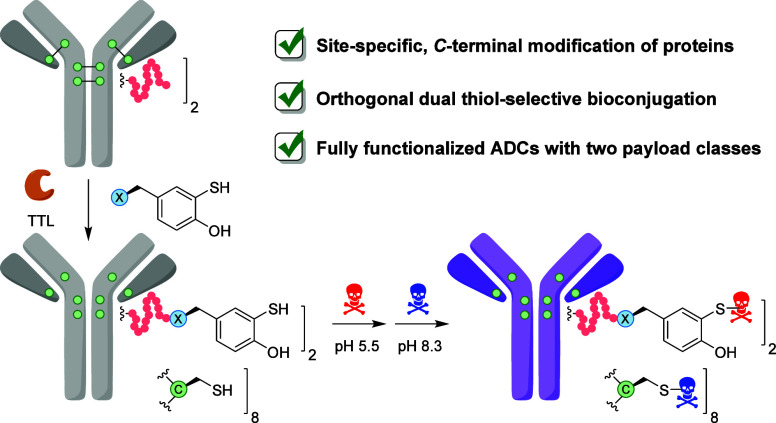

## Introduction

Chemical modification strategies enable the selective introduction of tailored functionalities to proteins, thereby providing access to protein bioconjugates with countless possibilities and prospects for the life sciences.^1–4^ The utility of such bioconjugation strategies ranges from the development of investigative tools in chemical biology, including fluorescent labeling,^5–7^ intracellular protein delivery^8,9^ and the characterization of post-translational modifications (PTMs),^10,11^ to protein–protein conjugation^12^ and the modification of therapeutic proteins.^13^ Antibody-drug conjugates (ADCs) combine the selective cellular targeting of antibodies with potent cytotoxins to obtain targeted drug delivery in oncology.^14–16^ The desire to generate homogeneous and even multipayload ADCs with defined conjugation sites and drug-to-antibody ratio (DAR) is perhaps one of the most illustrative arguments for the development of reliable methods which achieve sequential or simultaneous site-specific protein modifications.^3,4,17–21^

Protein bioconjugation strategies can be divided in the modification of native proteins, in which certain canonical amino acid residues (e.g., Lys, Cys) show suitable reactivity but lack specificity due to their ubiquitous presence, and genetically engineered proteins, which introduce selectivity at the cost of predesignated genetic modifications.^3,4^ In both scenarios, cysteine *S-*alkylation constitutes the dominant strategy due to the high nucleophilicity and chemoselectivity of the sulfhydryl moiety (Figure 1A).^22,23^ A wide scope of methods has been established for cysteine modification, with an increasing focus toward bioconjugations that occur at elevated reaction rates under stoichiometric conditions.^24,25^ Our lab contributed to this field with the P5-labeling platform,^26–28^ which allow the modular installation of unsaturated phosphorus electrophiles into functional modules,^26^ deliver highly stable conjugates and incorporate branched PEG-linkers for ADC stabilization.^29,30^

**Figure 1:**
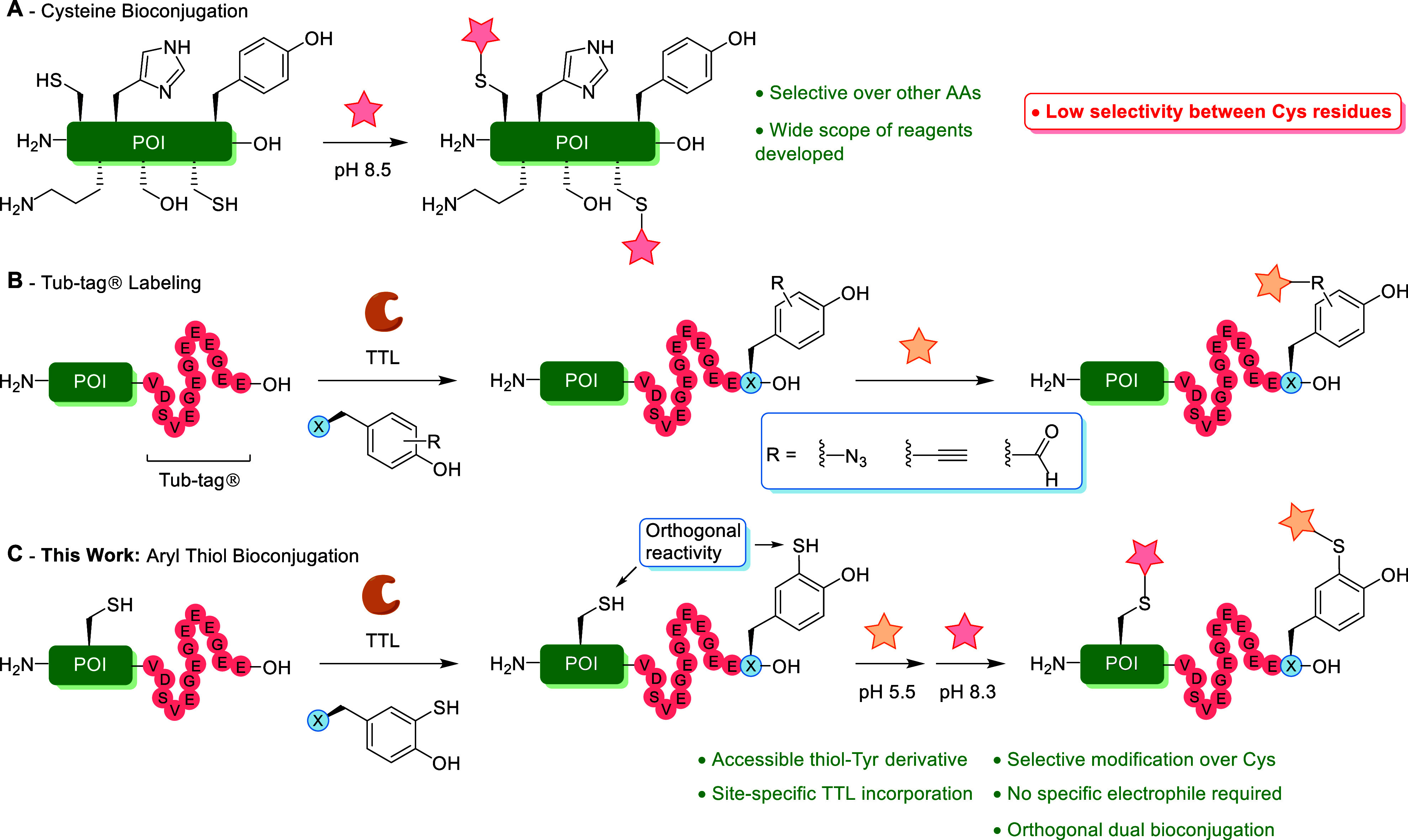
(A) Overview of cysteine bioconjugation, with particular emphasis on the challenge to obtain selectivity between individual Cys residues within proteins.^22–25^ (B) Tub-tag labeling employs the enzyme *tubulin tyrosine ligase* (TTL) to attach synthetic tyrosine derivatives on the C-terminal Tub-tag (VDSVEGEGEEEGEE). Reported tyrosine derivatives carry functional groups such as azides, alkynes, or aldehydes for subsequent chemoselective modifications.^34–37^ (C) Novel bioconjugation strategy outlined in this work, featuring a synthetic aryl thiol derivative of tyrosine which can be incorporated using TTL, followed by chemoselective modification using cysteine-selective reagents at lowered pH (5.5). This strategy is compatible for a dual thiol bioconjugation using native cysteine modification at pH 8.3 in a subsequent step.

Obtaining selectivity between individual cysteine residues within a protein remains challenging and often relies on differences in the p*K*_a_ values of individual residues as a result of their local environment.^31^ Common reagent-based strategies to circumvent this problem utilize the unique reactivity of the 1,2-aminothiol and 3-mercaptopropionic acid groups found in *N*-terminal cysteines (NCys) and *C-*terminal cysteines (CCys), respectively.^32,33^

A more complex reagent-based technique, π-clamp, genetically introduces a four-amino acid sequence (Phe-Cys-Pro-Phe) to fine-tune reactivity toward a custom perfluoroaromatic electrophile,^38,39^ enabling antibody modification^38^ and protein–protein conjugation.^40^ For selective antibody modification, unpaired Cys residues can be genetically introduced on both heavy chains, enabling site-specific modification after reoxidation of the native disulfide bonds to obtain a DAR 2 species (Thiomab).^41,42^ Another approach modifies native cysteines with an electrophile equipped with two orthogonally protected cysteines.^43^ Furthermore, a recent study has demonstrated that differential modification of native cysteines within antibodies can be achieved via novel dehydroalanine forming reagents.^44^

Thiophenolates (or aromatic thiolates; ArS^–^) constitute an appealing, yet underexplored class of nucleophiles which possess similar reactivity compared to aliphatic thiolates,^45,46^ such as cysteine or gluthathione (RCH_2_S^–^).^47,48^ An important distinction lies in the p*K*_a_ values of aryl thiols (∼ 6.5 in H_2_O)^46^ compared to alkyl thiols such as cysteine (∼ 8.5 in H_2_O).^49,50^ Based on this substantial p*K*_a_ difference, we hypothesized the selective modification of thiophenolates in the presence of native cysteines in an electrophile-independent manner, given that we can site-specifically incorporate this functional moiety into proteins. We reasoned that this step could be achieved by means of chemoenzymatic ligation.

Previous work from our laboratory and the Leonhardt group (LMU Munich) introduced Tub-tag labeling, a site-specific, chemoenzymatic modification strategy which employs the enzyme *tubulin tyrosine ligase* (TTL) to attach tyrosine derivatives to a hydrophilic recognition sequence (VDSVEGEGEEEGEE; Tub-tag) on the *C*-terminus of a protein of interest (POI, Figure 1B).^34–37^ In our current study, we introduce an aryl thiol-containing amino acid as an excellent nucleophile for site-specific protein bioconjugation. This S-Tyr derivative can be efficiently incorporated into proteins and antibodies using Tub-tag labeling, followed by selective modification in the presence of native cysteines (Figure 1C). Furthermore, we demonstrate sequential dual thiol bioconjugation with native cysteines at pH 8.3 in a subsequent step and apply this protocol toward fluorescent nanobodies and ADCs. The newly introduced nucleophile is compatible with a wide variety of unsaturated electrophiles due to its pH-dependent selectivity, and allows the generation of dual payload, fully functionalized ADCs.

## Results and Discussion

At the outset of our studies, we developed a three-step synthetic procedure to obtain thiolated derivatives of *L*-phenylanaline and *L*-tyrosine (Figure 2A). We reasoned that disulfide reagents would be optimal for both stability against oxidation and accessibility toward the corresponding sulfide upon tris­(2-carboxyethyl)­phosphine (TCEP)-mediated liberation in aqueous solution.^51^ Chlorosulfonation was used to install sulfur into *L*-Phe as previously reported.^52,53^ Instead of performing the subsequent reduction of chlorosulfonate **1** with Sn in HCl,^52,53^ we opted for reduction with phosphines in a mixture of dioxane and water.^54^ In addition to TCEP,^54^ we found that performing this transformation with triphenylphosphine^55^ not only reduced overall reagent cost, but also enabled extractive removal of triphenylphosphine oxide in the workup step. Disulfide formation was promoted by concentration of the crude **2** at elevated temperature, followed by HPLC purification to obtain **3**_**ab**_ (46% over three steps) as a mixture of symmetrical disulfides of 4-SH-*L*-Phe (**3**_**a**_)^52,53^ and 3-SH-*L*-Phe (**3**_**b**_), as determined by nuclear magnetic resonance (NMR; **3**_**a**_:**3**_**b**_, 4:1).

**Figure 2:**
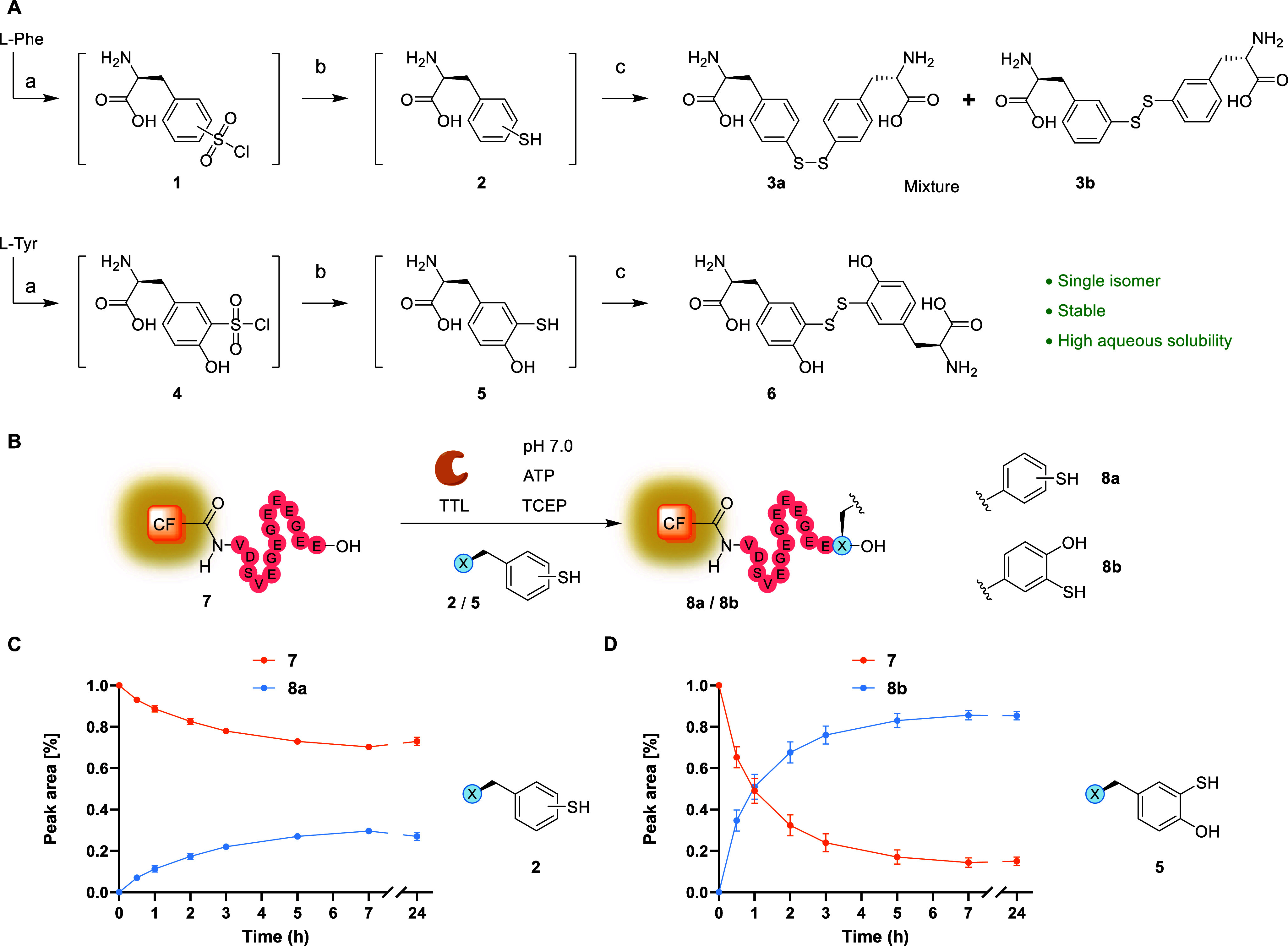
(A) Synthesis of aryl disulfide containing amino acid reagents **3**_**ab**_ and **6** from *L*-Phe and *L*-Tyr. Reagents/conditions: (a) chlorosulfonic acid (neat), −30 °C to rt; (b) PPh_3_ (4 equiv), dioxane/H_2_O (1:1), 90 °C; (c) workup, concentrate, 70 °C, 46% over 3 steps (**3**_**a**_ + **3**_**b**_), 21% over three steps (**6**). (B) Tub-tag labeling of 5(6)-CF-Tub-tag (**7**) using **2**/**5** to obtain **8a**/**8b**. (C, D) Tyrosination reactions (250 μL) were performed using the following conditions: 200 μM **7**, 1 μM SUMO-TTL (0.5 mol %), 1.25 mM **3**_**ab**_ **/6**, 25 mM TCEP, 5 mM ATP, 20 mM 3-(*N*-morpholino)­propanesulfonic acid (MOPS) pH 7.0, 100 mM KCl, 10 mM MgCl_2_, 10% (v/v) propane-1,2-diol, 37 °C, 850 rpm shaking. For **3**_**ab**_, 2.5 vol % DMSO was used as cosolvent for solubility. Crude reaction mixture samples (25 μL) were quenched with 1% TFA (25 μL) at indicated times and analyzed using LC-MS (Figures S1 and S2). Relative quantities of substrate (**7**) and product peptide (**8a**/**8b**) were determined from the corresponding peak areas in the UV spectra (λ = 220 nm). The mean values and standard deviation (SD) of three replicate reactions are shown (*N* = 3).

We reasoned that the initial chlorosulfonation step would be fully *ortho*-selective with respect to the phenol moiety of *L*-Tyr, as previously demonstrated by Drake and co-workers for 4-hydroxybenzoic acid.^56^ Furthermore, we anticipated that the 4-hydroxy group would be beneficial for TTL incorporation, as previously observed for 3-N_3_-*L*-Tyr and 3-formyl-*L*-Tyr.^34,35^ Reproducing the chlorosulfonation-reduction-disulfide formation procedure with *L*-Tyr as starting material afforded the symmetrical disulfide of 3-SH-*L*-Tyr (**6**) as the sole regioisomer in 21% yield over three steps.

With aryl disulfides **3**_**ab**_ and **6** in hand, we set out to evaluate TTL-mediated incorporation into 5,6-carboxyfluorescein Tub-tag (CF-Tub-tag, **7**, Figure 2B).^34,35^ Disulfides **3**_**ab**_ and **6** (1.25 mM) were used for *in situ* formation of the corresponding aryl thiols (**2** and **5**, 2.5 mM), which were incubated with CF-Tub-tag **7** (0.2 mM) in the presence of SUMO-TTL (1 μM, 0.5 mol %) at pH 7.0. The poor aqueous solubility of **2**/**3**_**ab**_ necessitated usage of 2.5 vol % DMSO as cosolvent, while the ligation with **5**/**6** was performed under full aqueous conditions. TCEP (25 mM) was used to form aryl thiols **2**/**5** and to subsequently suppress (DMSO mediated)^57^ oxidative coupling to form diaryl disulfides within the reaction mixture. We did not investigate dithiothreitol (DTT) or l-glutathione (GSH) as reductants, as these could potentially interfere with subsequent bioconjugation reactions employing thiophilic electrophiles.

Enzymatic conversion of **7** to form **8a**/**8b** was measured in a time-course experiment (t = 30, 60, 180, 300, 420 min and 24 h, N = 3, Figure 2 **C–D**, Figures S1 and S2) using relative peak intensity measured by liquid chromatography–mass spectrometry (LC-MS).^34,35^ Ligation using **2** slowly reached a plateau at ∼ 30% ligation. In contrast, the TTL incorporation with **5** was both rapid and efficient, obtaining ≥ 75% ligation after 3 h and reaching a plateau at ∼ 85% ligation. These assay results are in accordance with the previously described 3-azido-*L*-Tyr and 3-formyl-*L*-Tyr reagents,^34^ and place further emphasis on the substrate promiscuity of TTL.^35^ We decided to proceed our study with disulfide reagent **6** based on its regioisomeric purity, high aqueous solubility and excellent TTL incorporation.

Next, we aimed to characterize the properties and reactivity of amino acid **5** toward thiophilic electrophiles. First, we performed 2D NMR analysis to accurately determine the p*K*_a_ value of the aryl thiol moiety of **5** (Figure 3A, Figures S3 and S4). Disulfide **6** (5 mM) was treated with TCEP (10 mM) in 0.2 M citrate (pH 4.5 – 6.0) and phosphate (pH 6.0 – 8.0) buffers containing 10% D_2_O. Highly acidic (pH 1.0) and basic (pH 13.0) reference samples were obtained using 1% HCl (w/w) and 1% NaOH (w/w), respectively. The pH dependent signals of the aromatic carbons 6 (C6) in (^1^H–^13^C)-HMQC (Figure 3A, Figure S3) and 3 (C3) in (^1^H–^13^C)-HMBC (Figure S4) were used to calculate the p*K*_a_, obtaining a value of 5.5 for both measurements. These results confirm that the 4-hydroxy group not only promotes aqueous solubility and TTL ligation, but also greatly influences the acidity of the aryl thiol, as the calculated p*K*_a_ value for **5** is markedly lower than the reported value of 6.5 for thiophenol.^46^

**Figure 3:**
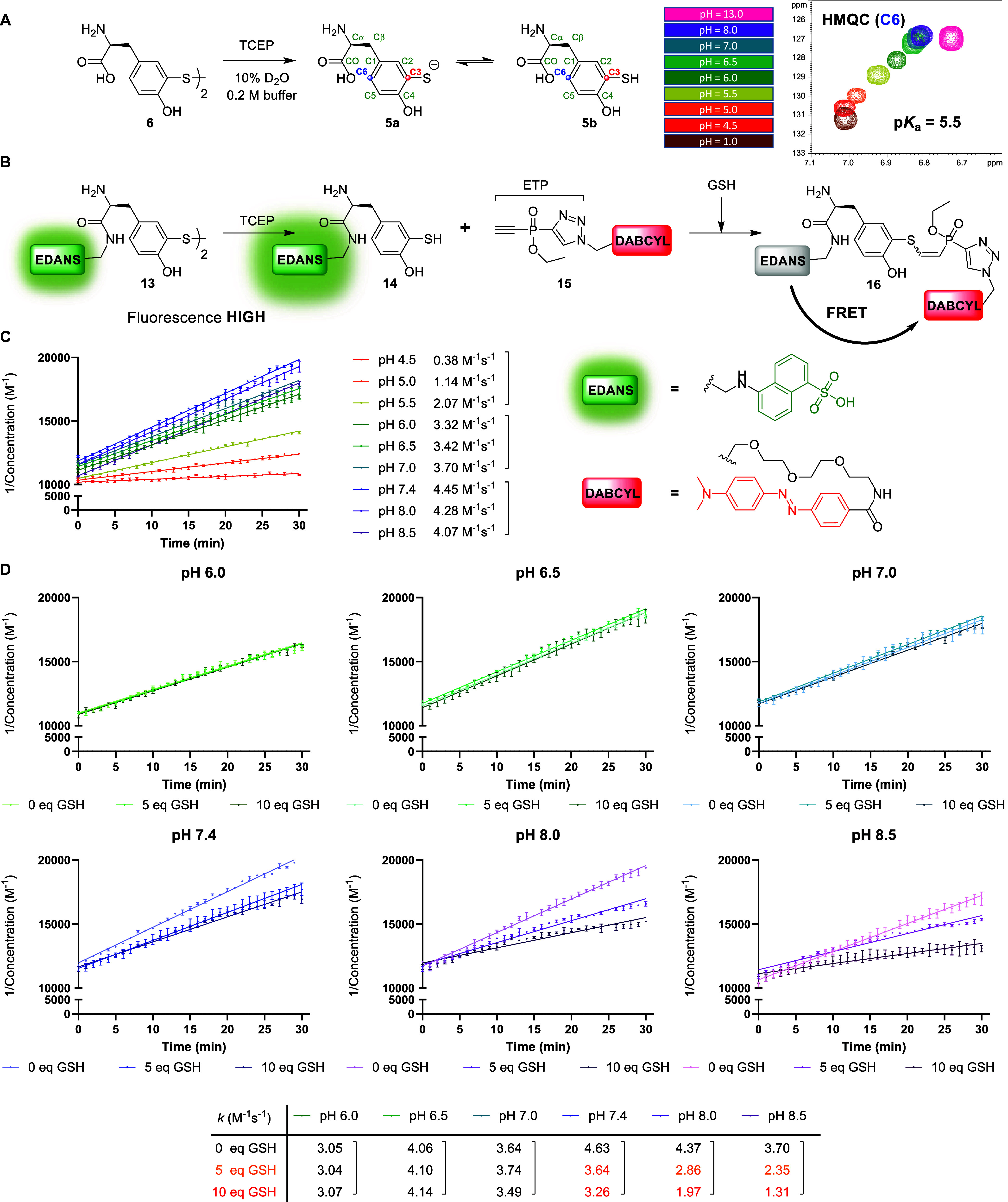
(A) 2D NMR analysis to determine the p*K*_a_ value of the 2-mercaptophenol moiety of **5**. (B) Fluorescent plate reader assay to monitor the reaction kinetics between EDANS-aryl thiol **14** and ETP-DABCYL **15**. Formation of conjugate **16** quenches the fluorescence emitted (λex = 340 nm, λem = 495 nm) by virtue of FRET. (C) Aryl thiol addition kinetics determined for the reaction between **14** (100 μM; preformed by treating 50 μM **13** with 200 μM TCEP) and **15** (100 μM) to form **16** at pH 4.5–8.5 (0.2 M citrate/phosphate/Tris buffer, 10% MeCN, 5% DMSO). Mean values and standard deviation (SD) are shown (*N* = 1, *n* = 2). Data was recorded in three independent experiments. (D) Competition assay for aryl thiol addition kinetics where the effect of 0/5/10 equiv of glutathione (GSH; with 1 equiv additional TCEP per GSH) was investigated for pH 6.0 – 8.5. Mean values and standard deviation (SD) are shown (*N* = 1, *n* = 2). Data was collected in six independent experiments.

Ethynyl-triazolyl-phosphinates (ETPs)^58^ were selected as electrophiles for characterizing the aryl thiolate reactivity of **5** due to their excellent reactivity and modular accessibility via Cu^I^-catalyzed azide alkyne cycloaddition (CuAAC).^59,60^ Disulfide **6** (5 mM) was treated with 4 equiv of TCEP and 2 equiv of ETP-Biotin **9** ^58^ in 0.2 M phosphate buffer pH 6.0 (containing ∼ 10% MeCN and DMSO). Full conversion was observed after 10 min and HPLC purification afforded aryl thiol ETP-Biotin conjugate **10-Z/E** in 43% yield (Figure S5). HRMS and NMR analysis confirmed the proposed structure, and revealed the resulting alkene was primarily (>90%) in the Z-configuration (Figures S6 and S7). Performing the equivalent reaction with disulfide **3**_**ab**_ at pH 7.0 for 15 min afforded the main product (from **3**_**a**_) exclusively as the Z-isomer (**11-Z**) in 23% yield (Figures S8 and S9) in addition to a mixture of **11-Z** and **12-Z** (from **3**_**b**_ **,** data not shown). These initial results are in agreement with previous experimental^26,28,58^ and computational^61^ investigations into the reactivity of alkyl thiol addition to unsaturated P5 electrophiles.

Subsequently, we developed a plate reader assay based on fluorescence quenching to further characterize the nucleophilic reactivity of the aryl thiolate with ETP electrophiles (Figure 3B). We reasoned that equipping disulfide **6** with the 5-(2-aminoethylamino)­naphthalene-1-sulfonic acid (EDANS) fluorophore would result in a fluorescent signal which is prone to Förster resonance energy transfer (FRET) quenching upon conjugation to an ETP equipped with a 4-[4-(dimethylamino)­phenylazo]­benzoyl (DABCYL)­quencher.^62–65^ EDANS-diaryl disulfide **13** and ETP-DABCYL **15** were synthesized as described in Scheme S1. We initially subjected **13** (50 μM) to TCEP (200 μM) and **15** (200 μM) in 100 μL phosphate buffered saline (PBS; pH 7.4 with 5% DMSO). TFA quenching after 120 min followed by LC-MS analysis confirmed complete conversion to the aryl thiol-ETP adduct **16** (Figure S10). To improve reactant solubility, we used 0.2 M citrate/phosphate/Tris buffer with 5% DMSO and 10% MeCN as cosolvents. We synthesized **16** and NH_2_-DABCYL **21** (Scheme S1) to set up control measurements for 100% and 0% fluorescence, thereby enabling data normalization for each measured time point. Finally, the concentration of **15** was lowered to 100 μM (1 mol equiv with respect to the reduced EDANS aryl sulfide **14**) to enable simple linear regression for rate constant determination.

Using these assay conditions, we analyzed the aryl thiolate – ETP reaction for pH 4.5 – pH 8.5 and obtained kinetic rate constants (*k*) ranging from 0.38 to 4.5 M^–1^s^–1^ (Figure 3C, Figures S11 – 13, N = 1, n = 2). Interestingly, *k* values change dramatically from pH 4.5 (0.38 M^–1^s^–1^) to pH 5.0 (1.1 M^–1^s^–1^), pH 5.5 (2.1 M^–1^s^–1^) and pH 6.0 (3.3 M^–1^s^–1^), which is in agreement with the experimentally determined p*K*_a_ value of 5.5. The highest *k* value was observed at pH 7.4 (4.45 M^–1^s^–1^), which decreased to 4.07 M^–1^s^–1^ at pH 8.5.

Next, we repeated the fluorescent plate reader assay using GSH (0, 5, and 10 equiv; 1 equiv of additional TCEP per GSH) as a competing cysteine nucleophile for pH values 6.0 – 8.5 (Figure 3D, Figures S14 – 19, N = 1, n = 2). The rate of aryl thiol addition on the ETP electrophile was virtually identical at pH 6.0, but was heavily affected by competing GSH at pH 7.4 – 8.5. These observations are in agreement with the p*K*_a_ value of GSH (8.5 – 8.8)^66–68^ and moreover imply that selective aryl thiolate modification within cysteine-containing proteins can be achieved at lower pH values (≤6.0) with near-stoichiometric equivalents of electrophile. Furthermore, this data supports the hypothesis that aryl thiol **5** is a valuable reaction handle that could be selectively modified in the presence of endogenous thiols. Finally, we utilized the fluorescently quenched aryl thiol-ETP adduct **16** as a tool to examine the stability of the aryl thiol-ETP bond in human serum, PBS with 10 mM GSH, 0.2 M citrate buffer pH 4.5 and 0.2 M Tris buffer pH 8.5 (Figure S20; 5% DMSO). These results confirmed the excellent stability of the aryl thiol-ETP linkage. As a control experiment, we also examined the stability of aryl thiol-maleimide adduct **24** in human serum and PBS with 10 mM GSH (Scheme S2, Figure S21; 5% DMSO). Here, the observed rapid increase in fluorescence corresponds to retro-Michael addition of maleimide conjugate **24** with serum proteins/GSH.^69^

Subsequently, we translated our findings toward the labeling of camelid-derived single domain antibodies (sdAbs; V_H_Hs; nanobodies).^70,71^ GBP1, a nanobody that binds green fluorescent protein (GFP) for fluorescence enhancement (GFP-Enhancer; PDB: 3K1K),^72^ was previously employed for Tub-tag labeling,^34,35,37^ and served as starting point for our protein conjugation experiments. Preliminary experiments with GBP1-Tub-tag (**25**, 25 μM), aryl disulfide **6** (1.25 mM), TCEP (25 mM) and SUMO-TTL (1 μM, 4 mol %) revealed the sensitivity of this ligation with protein substrates toward high concentrations of TCEP, showing incomplete ligation (data not shown). Therefore, the conditions were adjusted toward usage of 2 mol equiv of TCEP (2.5 mM) to ensure compatibility toward a wide scope of proteins while retaining a reductive environment. This modification was confirmed with the previously discussed CF-Tub-tag **7** (0.2 mM) labeling assay (Figure S22). Next, we genetically inserted a Cys-containing motif (AGCGA) into the C-terminal region of GBP1-Tub-tag (**25**) to obtain the nanobody mutant GBP1-C-Tub-tag **26**, thereby enabling the incorporation of **5** and subsequent modification of the aryl thiol modality in the presence of a competing, freely accessible cysteine residue.

Tub-tag labeling of **26** (100 μM) with **6** (1.25 mM), TCEP (2.5 mM) and SUMO-TTL (6 μM, 6 mol %) for 4 h (37 °C) afforded full conversion toward GBP1-C-Tub-tag-STyr (**27**) (Figure 4A, Table S1). Intact protein-MS of **27** revealed a moderate degree of oxidation from the aryl thiol to the corresponding sulfinic acid (M + 32) and sulfonic acid (M + 48), which contrasted the previously observed disulfide reformation for **5** (back to **6**) in the absence of reducing conditions or a Michael acceptor (e.g., Figure S10). The reaction mixture (130 μL) was rebuffered with Zeba Spin desalting (0.5 mL, 7K MWCO) to remove excess small molecules (e.g., aryl thiol **5**) and to adjust the pH value. Subsequently, incubation with TCEP at 37 °C (30 min) was followed by adding the respective ETP electrophile at 25 °C (16–20 h). Modification of **27** with ETP-Biotin **9** (Figure 4A, Table S2) was initially attempted at pH 6.0 (0.2 M phosphate buffer) with 1000 μM TCEP and 200 μM **9**, resulting in a significant degree of overmodification (17% based on intact protein-MS). Stepwise adjustment of the conditions to pH 5.5 (0.2 M citrate buffer) with 300 μM TCEP and 120 μM **9** selectively afforded full conversion to GBP1-C-Tub-tag-STyr­(Biotin) (**28**).

**Figure 4:**
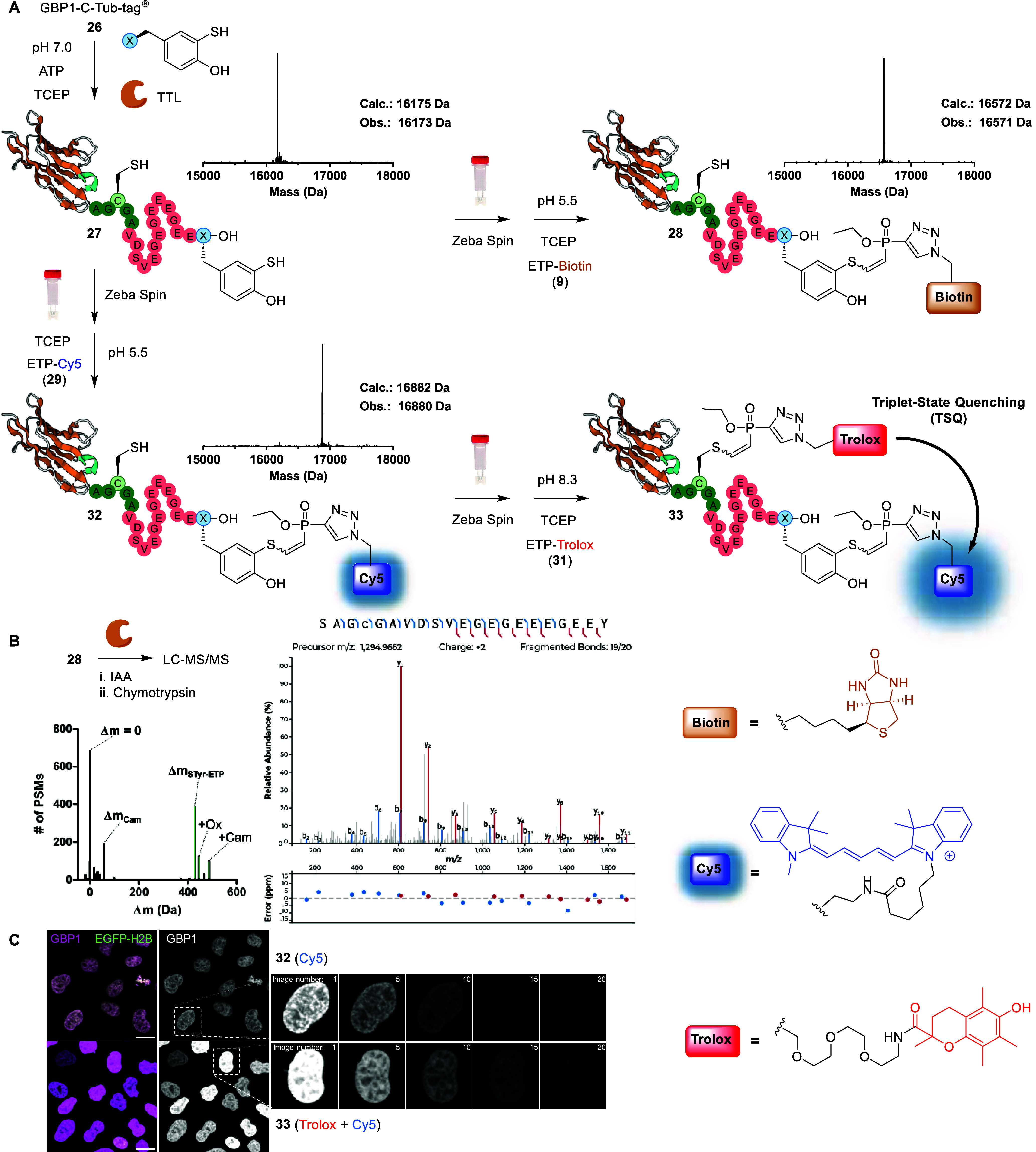
(A) Tub-tag labeling and subsequent (dual) bioconjugation of GBP1-C-Tub-tag (**26**), including intact protein-MS traces for **27**, **28**, and **32**. Top left: Tub-tag labeling of **26** with aryl disulfide **6**, TCEP, ATP, and SUMO-TTL to afford GBP1-C-Tub-tag-STyr (**27**). Full conditions in Table S1. Top right: selective aryl thiol modification of **27** with ETP-Biotin **9** to obtain GBP1-C-Tub-tag-STyr­(Biotin) (**28**). Full conditions and optimization in Table S2. Bottom left: selective aryl thiol modification of **27** with ETP-Cy5 **29** to obtain GBP1-C-Tub-tag-STyr­(Cy5) (**32**). Full conditions and optimization in Table S3. Bottom right: cysteine modification of **32** with ETP-Trolox **31** to obtain nanobody double conjugate GBP1-C­(Trolox)-Tub-tag-STyr­(Cy5) (**33**). Full conditions and optimization in Table S4. (B) Conjugate **28** was treated with iodoacetamide (IAA) prior to digestion with chymotrypsin and analyzed by LC-MS/MS. Left: histogram of the modifications detected in MS-Fragger. Δ*m* of STyr-ETP (**5** + **9**) is highlighted in green. Ox = oxidation (15.99 Da), Cam = carbamidomethylation (+57.02 Da). Right: MS/MS spectrum identifying the linkage site of **28**. (C) Confocal microscopy of conjugates **32** and **33** (λex = 640 nm, λem = 685/50 nm, scale bar = 20 μm) bound to HeLa-H2B-EGFP cells. Left: images obtained by exposure to 5% laser power. Right: images obtained at 5 s intervals by exposure to 100% laser power, thereby inducing photobleaching.

To substantiate the selectivity of the aryl thiolate – ETP reaction, **28** was treated with excess iodoacetamide (IAA), digested with chymotrypsin and analyzed by high-resolution liquid-chromatography coupled tandem mass spectrometry (LC-MS/MS). The data was analyzed by performing an open search to identify the modification pattern in an unbiased fashion.^73^ This confirmed that the most prominent modification corresponds to the expected mass of **5** alkylated with ETP **9** (Δ*m*_exp_, 429 (397 + 32) with respect to *L*-Tyr), indicating stable modification (Figure 4B). To further validate the site-selectivity, we used the obtained modification mass to perform a closed search. This resulted in more than 400 peptide spectrum matches (PSMs) that localize the modification to the terminal tyrosine, with several spectra also containing the IAA modified Cys-129, thus unambiguously verifying 3-SH-*L*-Tyr-146 as the mayor site of modification. In addition, some spectra identified only Cys-129 or both Cys-129 and 3-SH-*L*-Tyr-146 to be modified with ETP **9**. However, label free quantification revealed that these correspond to <1% of the total modified peptides (Supporting Excel Table).

We reasoned that the selective modification of **27** could be combined with another, successive ETP modification to target the genetically inserted Cys residue. Intramolecular photostabilization is an emerging strategy to enhance the performance of fluorophores.^74–78^ An established example of this concept is the stabilization of cyanine fluorophores^74^ by means of the triplet-state quencher (TSQ) Trolox.^79^ We hypothesized that the hydrophilic and flexible Tub-tag sequence would allow sufficient proximity for photostabilization^75^ between the mutated Cys residue and the *C*-terminally introduced aryl thiol. ETP-Cy5 (**29**) and ETP-Trolox (**31**) were synthesized as described in Scheme S3 in one and two steps, respectively. Modification of **27** with **29** (Figure 4A, Table S3) was performed at pH 5.5 (0.2 M citrate buffer) with 300 μM TCEP, in which 145 μM ETP-Cy5 **29** was used to obtain GBP1-C-Tub-tag-STyr­(Cy5) (**32**) with sufficient conversion and without overmodification (≥90% based on intact protein-MS). The increased equivalency of **29** (compared to **9**) required to reach full conversion for the aryl thiol addition is most likely due to the pronounced hydrophobicity of the Cy5 fluorophore. Subsequent rebuffering of **32** into 50 mM Tris buffer (1 mM EDTA, 100 mM NaCl, pH 8.3) was followed by modification with ETP-Trolox **31** (150 μM) to obtain the nanobody double conjugate GBP1-C­(Trolox)-Tub-tag-STyr­(Cy5) (**33**; Figure 4A, Table S4).

The fluorescence photostability of nanobody conjugates **32** and **33** was compared using confocal microscopy. HeLa-Kyoto cells stably expressing a fusion protein of histone 2B-green fluorescent protein (HeLa-H2B-GFP) were fixed and permeabilized, followed by overnight immunostaining at 4 °C using 2.5 μg/mL of **32/33**. After washing, initial confocal images were obtained using 5% laser power (λex = 640 nm, λem = 685/50 nm). Both **32** and **33** colocalized well with the H2B-GFP signal within the nuclei (Figure 4C). The images also show that cells treated with **33** showed higher Cy5 fluorescence versus **32**-treated cells. Subsequently, fluorescence photobleaching was performed by exposing nanobody-stained cells to 100% laser power at 5 s intervals. Under these photobleaching conditions, cells treated with **33** displayed an enhanced initial fluorescence signal compared to **32**-treated cells. Stability against photobleaching was moderately enhanced for **33**-treated cells, in which the fluorescence signal remained after 30 images while **32-**treated cells were photobleached after 15 images (Figure 4C, Figure S23). These results confirm the intramolecular photostabilization of Cy5-Trolox nanobody conjugate **33** and provide a clear example of how the selective and successive installation of two thiophilic electrophiles enables additive functionality within this nanobody system.

We were intrigued to utilize our newly developed methodology toward the construction of dual payload ADCs. This advanced type of ADCs can deliver two (or more) therapeutic agents with complementary effects to the target cell, thus restricting drug resistance.^15,21^ We selected Brentuximab-LC-Tub-tag (**34**, Figure 5)^80^ as model antibody system for Tub-tag labeling with **5**, with the aim to subsequently functionalize the aryl thiolate using an ETP-drug conjugate. Provided that the initial ETP reaction would selectively afford a DAR 2 species, we rationalized that this modification could be combined with a successive DAR 8 modification (targeting native Cys residues) to obtain a DAR 10 dual payload ADC. We reasoned that our compact hydrophilic P5 electrophiles would be ideal for this second step, since they readily generate homogeneous DAR 8 species.^30^ Combining the tubulin-binding payloads monomethyl auristatin E (MMAE) and monomethyl auristatin F (MMAF) into a single ADC has shown to be a powerful strategy to overcome both antigen heterogeneity and drug resistance.^21,43,81,82^ MMAE is a highly potent, cell permeable drug which can diffuse into the tumor microenvironment after killing of the target cell, allowing it to exert ‘bystander killing’.^83^ However, the potency of MMAE is restricted by its removal from tumor cells that carry drug efflux transporters such as multidrug resistance protein 1 (MDR-1).^84^ Although structurally similar to MMAE, MMAF is cell impermeable due to its carboxylate moiety. This disables the bystander effect, but also ensures that MMAF stays in the target cell after ADC-mediated delivery. Based on these considerations, we synthesized ETP-PEG_3_-Val-Cit-PAB-MMAF (**36**, synthetic details in Scheme S4) as reagent for aryl thiolate modification and utilized the previously described P5­(PEG_12_)-Val-Cit-PAB-MMAE (**37**)^30^ for native Cys modification.

**Figure 5:**
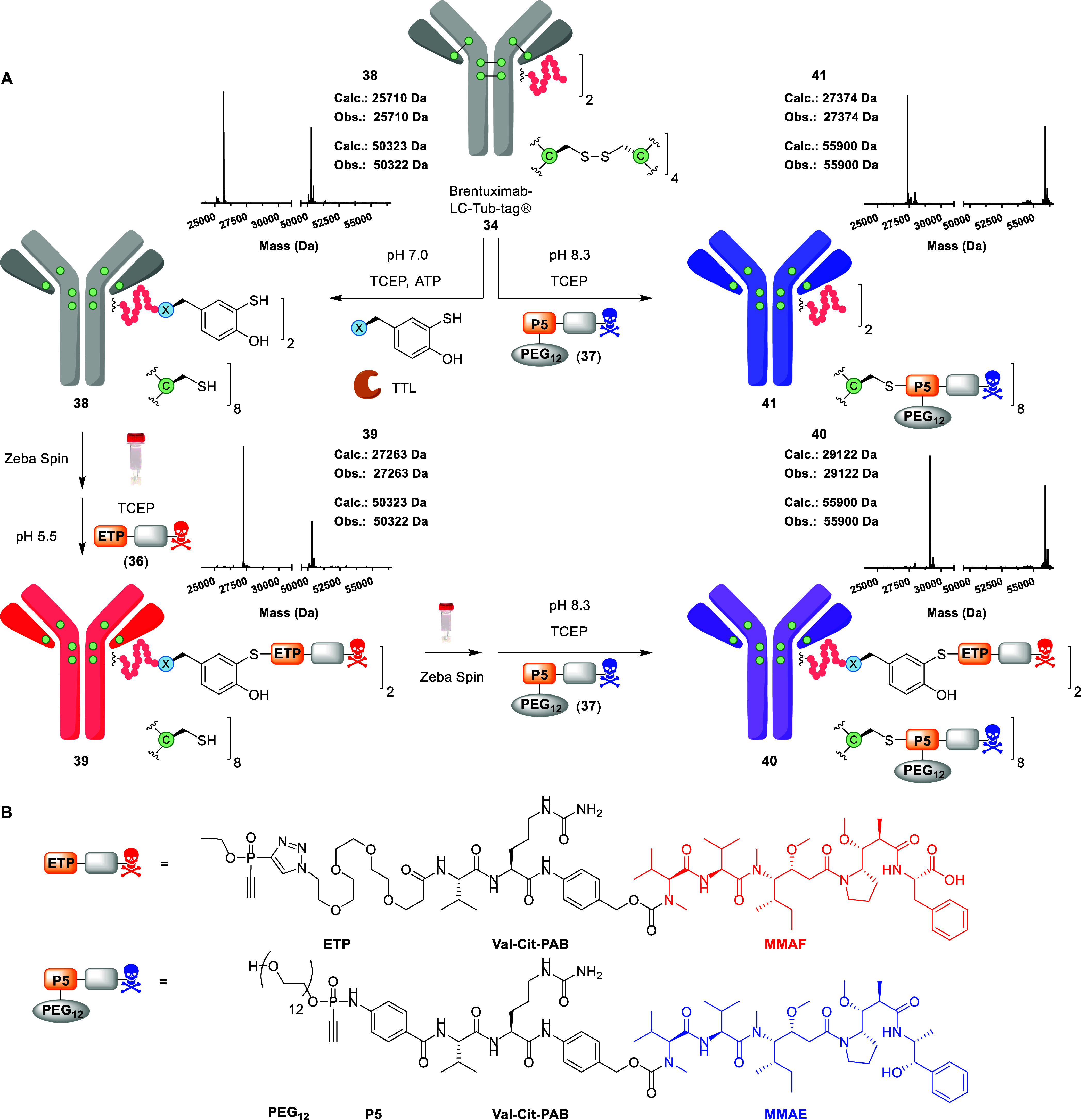
(A) Tub-tag labeling and subsequent (dual) bioconjugation of Brentuximab-LC-Tub-tag (**34**), including intact protein-MS traces for **38–41** and legend tables. Top left: Tub-tag labeling of **34** with aryl disulfide **6**, TCEP, ATP and SUMO-TTL to afford Brentuximab-LC-Tub-tag-STyr (**38**). Full conditions in Table S5. Bottom left: selective aryl thiol modification of **38** with ETP-PEG_3_-Val-Cit-PAB-MMAF (**36**) to obtain Brentuximab DAR 2 MMAF conjugate **39**. Full conditions and optimization in Table S6. Bottom right: Cysteine modification of DAR 2 conjugate **39** with P5­(PEG_12_)-Val-Cit-PAB-MMAE (**37**) to obtain Brentuximab DAR 2 MMAF DAR 8 MMAE double conjugate **40**. Full conditions in the SI. Top right: Cysteine modification of Brentuximab-LC-Tub-tag (**34**) with P5­(PEG_12_)-Val-Cit-PAB-MMAE **37** to obtain Brentuximab DAR 8 MMAE conjugate **41**. Full conditions in the SI. (B) Complete chemical structures of the thiophilic drug payloads **36** and **37**.

Tub-tag labeling of **34** (40 μM, 130 μL volume) with **6** (1.25 mM) and TCEP (2.5 mM) was optimized toward full formation of Bren-LC-Tub-tag-STyr (**38**) after 4 h at 37 °C (Figure 5, Table S5). Subsequently, we investigated the DAR 2 MMAF modification of **38** (Figure 5, Table S6) using the previously outlined steps: Zeba Spin rebuffering (0.5 mL, 7K MWCO), TCEP reduction (37 °C, 30 min) and ETP modification (25 °C, 16–20 h). An initial attempt using 1000 μM TCEP for the reduction and 160 μM **36** (2 equiv per LC) for the aryl thiol modification at pH 6.0 resulted in moderate conversion to **39** (55% based on intact protein-MS). We optimized this selective modification to near-completion (≥95% based on intact protein MS) by employing a double rebuffering step, reducing the TCEP concentration to 750 μM, and performing the modification with 240 μM **36** (3 equiv per LC) at pH 5.5 (0.2 M citrate buffer; Table S6). The high degree of selectivity for the ETP modification was also confirmed by hydrophobic interaction chromatography (HIC, Table S6).

MMAF-conjugate **39** was subjected to Zeba Spin rebuffering (0.5 mL, 7K MWCO), TCEP reduction (480 μM, 37 °C, 30 min) and P5 modification (800 μM, 25 °C, 16–20 h) at pH 8.3 (50 mM Tris buffer) to obtain DAR 10 dual conjugate **40** (Figure 5). Analogously, **34** was rebuffered to pH 8.3 (50 mM Tris buffer) and reduced with TCEP (400 μM, 37 °C, 30 min) prior to P5 modification (640 μM, 25 °C, 16–20 h), yielding the DAR 8 conjugate **41** (Figure 5). The final conjugates (**39**, **40** and **41)** were purified by fast protein liquid chromatography (FPLC) prior to analysis by HIC and size exclusion chromatography (SEC; Figure S24).

With ADCs **39** – **41** in hand, we evaluated the cytotoxicity on a panel of five CD30+ cell lines (SR-786, L-540, SU-DHL-1, Karpas-299, and L-428) after 96 h of incubation (Figure 6, Figure S25). Furthermore, we also examined the supernatant-based bystander cytotoxicity on HL-60 cells for each CD30+ cell line tested (96 h of incubation with supernatant). In most cases, ADC **39** (DAR 2 MMAF) suffices for the direct, CD30-mediated cell killing with EC50 values ranging from 23.2 to 65.5 pM (Figure S25). However, the cell impermeability of MMAF excludes bystander cytotoxicity for ADC **39**. Conversely, ADCs **40** and **41** display bystander cytotoxicity (EC50 ranging from 162 – 271 pM; Figure S25) due to the high cell permeability of MMAE. As expected, ADC **40** generally displays the most potent EC50 values due to its high drug loading. However, the discrepancy between ADCs **39** – **41** is perhaps best illustrated with the L-428 cell line (Figure 6 **C and** [Fig fig6] **E**), which displays upregulation of the MDR1 gene.^85^ Here, the increased expression of P glycoprotein (Pgp) drastically reduces the efficacy of ADC **41** (DAR 8 MMAE) due to increased MMAE efflux. While the DAR 2 MMAF conjugate (**39**) proved sufficient for direct cell killing and the DAR 8 MMAE conjugate (**41**) could still be used to induce bystander cell death, the DAR 10 MMAF-MMAE dual conjugate (**40**) exclusively succeeded to achieve both these effects toward the resistant L-428 cell line.

**Figure 6:**
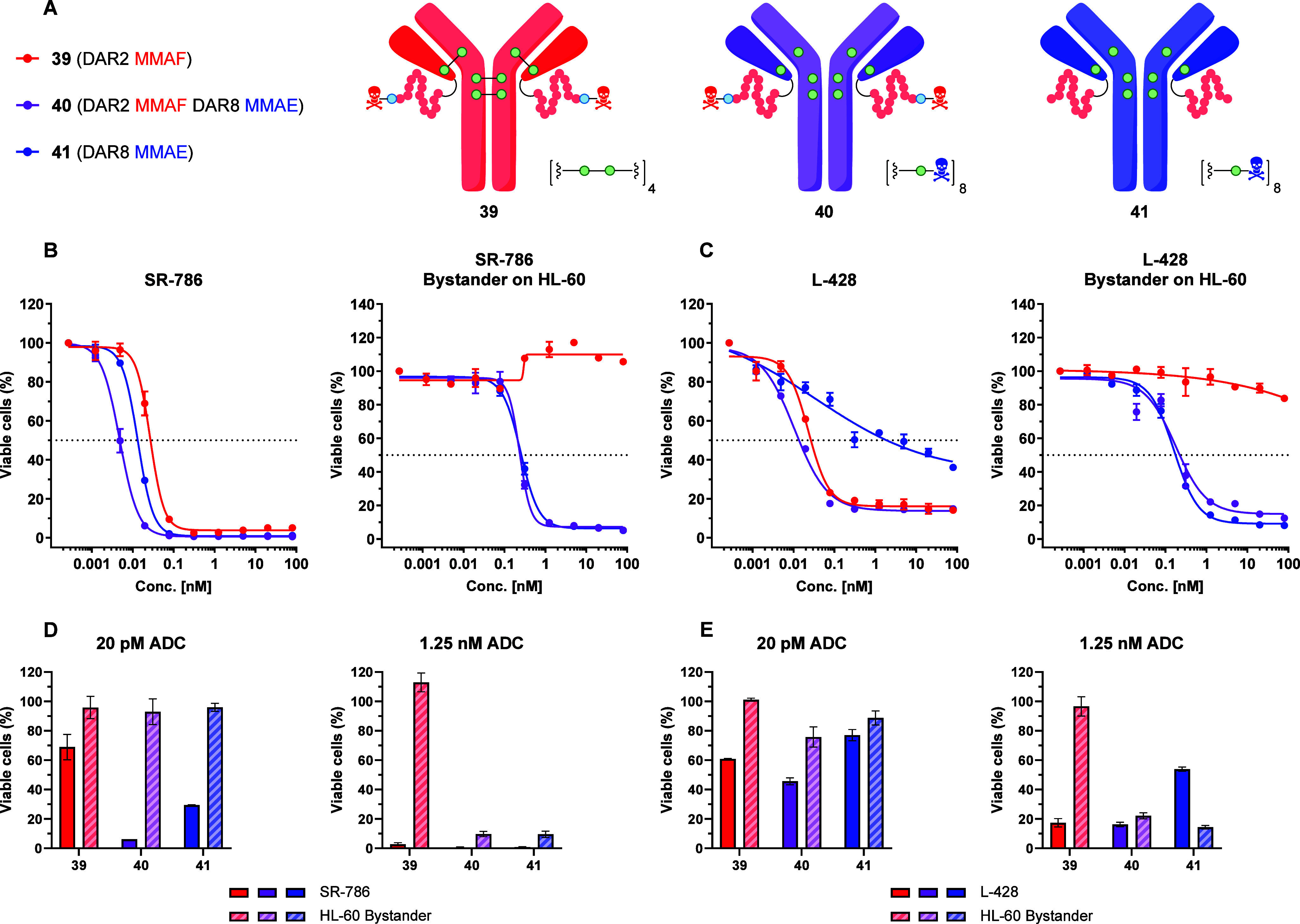
*In vitro* evaluation of ADCs **39**–**41** toward CD30-mediated cytotoxicity (96 h incubation) and supernatant-based bystander cytotoxicity (on HL-60 cells; 96 h incubation). (A) Graph legend with ADC structures **39**–**41**. (B, C) Direct and bystander (HL-60) cytotoxicity for cell lines SR-786 and L-428. Graphs display the concentration of ADC (0–80 nM; 4-fold dilution steps) on the *x*-axis and the % of viable cells on the *y*-axis (where 100% represents the medium control). Mean values and standard error of the mean (SEM) for one experiment is shown (*N* = 1, *n* = 2). Additional cell lines tested (L-540, SU-DHL-1, Karpas-299) are shown in Figure S25. (D, E) Bar graphs compare the measured % of viable cells at selected ADC concentrations (20 pM and 1.25 nM) for direct (left bars) and bystander (HL-60, right bars) cytotoxicity observed for SR-786 and L-428 cells. Data represents a selection of B and C (mean values and SEM; *N* = 1, *n* = 2).

## Conclusions

With this study, we demonstrate the feasibility of selective protein and antibody bioconjugation using aryl thiol nucleophiles with several thiol-reactive unsaturated electrophiles. For the site-specific incorporation of aryl thiols we introduce a new amino acid, 3-SH-*L*-Tyr, which is compatible with rapid *C*-terminal Tub-tag labeling. The p*K*_a_ value of 5.5 for the aryl thiol of 3-SH-*L*-Tyr allows the subsequent aryl thiolate modification in the presence of native Cys residues under mildly acidic conditions. Furthermore, we paired this method with successive Cys labeling using P5-labeling reagents to obtain an orthogonal thiol-selective dual bioconjugation protocol for proteins. Finally, we apply our labeling protocol toward a highly fluorescent photostabilized nanobody and a fully functionalized dual payload ADC. By combining the effects of MMAF and MMAE, this conjugate delivered both a potent antigen-mediated cytotoxic response toward target cells irrespective of MDR1 upregulation, in addition to displaying a strong bystander effect and is therefore perfectly equipped to address the complexity of heterogeneous tumor populations as well as payload mediated resistance mechanisms.

Because this method relies on a substantial p*K*_a_ difference between the aryl thiol nucleophile and native Cys residues, it starkly contrasts many site-specific protein modification strategies in the field which often utilize custom electrophilic reagents to achieve their desired selectivity or specificity. This unique property could be valuable for the ongoing efforts to combine site-specific bioconjugation strategies to achieve homogeneous multipayload ADCs.^20,21^ In addition, the unique properties of aryl thiols also hold promise for other applications, for instance in the engineering of designer enzymes using 4-SH-*L*-Phe for Au-mediated catalysis,^86^ thereby reaching beyond the realm of bioconjugation chemistry.

## Supplementary Material





## Notes

Supporting figures, schemes, and tables; experimental procedures and methods, and characterization data (PDF)
Supporting Excel Table: proteomic analysis of Biotin-labeled nanobody conjugate **28** (XLSX)
