# Supplementary material for: Orthogonal Site-Specific Dual Bioconjugation of Aryl and Alkyl Thiols
Source: J Am Chem Soc. 2025 May 5;147(22):18888–900. doi: 10.1021/jacs.5c02981 (PMC12147148; doi:10.1021/jacs.5c02981)
Supplement: Supplementary file 1 [file ja5c02981_si_001.pdf]

Supporting Information (SI):

Orthogonal Site-Specific Dual Bioconjugation of Aryl and Alkyl Thiols

Mark A. R. de Geus[†], Christian E. Stieger[†, §], Jan Vincent V. Arafiles[†], Jean-Romain P. J. Lotthé[†, §], Peter Schmieder[†], Kristin Kemnitz-Hassanin[†], Beate Kindt[†], Heinrich Leonhardt[||], Saskia Schmitt[‡], Marcus Gerlach[‡], Dominik Schumacher[‡], Jonas Helma[‡], Marc-André Kasper[‡] and Christian P. R. Hackenberger[†, §, *]

[†]Leibniz-Forschungsinstitut für Molekulare Pharmakologie (FMP), Robert-Rössle-Straße 10, 13125 Berlin, Germany
[§]Department of Chemistry, Humboldt Universität zu Berlin, Brook-Taylor-Straße 2, 12489 Berlin, Germany
[||]Faculty of Biology and Center for Molecular Biosystems (BioSysM), Human Biology and BioImaging, Ludwig-Maximilians-Universität München, Butenandtstraße 1, 81377 Munich, Germany
[‡]Tubulis GmbH, Am Klopferspitz 19a, 82152 Planegg-Martinsried, Germany

*E-mail: hackenbe@fmp-berlin.com

Table of Contents

1. Supplementary Figures 6
Figure S1 6
Figure S2 7
Figure S3 8
Figure S4 9
Figure S5 10
Figure S6 11
Figure S7 12
Figure S8 13
Figure S9 14
Figure S10 15
Figure S11 16
Figure S12 17
Figure S13 18
Figure S14 19
Figure S15 20
Figure S16 21
Figure S17 22
Figure S18 23
Figure S19 24
Figure S20 25
Figure S21 26
Figure S22 27
Figure S23 28
Figure S24 29
Figure S25 30
2. Supplementary Schemes 31
Scheme. S1 31
Scheme. S2 32
Scheme. S3 33
Scheme. S4 34
3. Supplementary Tables 35
Table S1 35
Table S2 36
Table S3 37

Table S4 38
Table S5 39
Table S6 40
4. General Information 41
4.1 Chemicals and Solvents 41
4.2 Flash- and thin layer chromatography 41
4.3 Semi-preparative HPLC.. 41
4.4 NMR-Spectroscopy.. 41
4.5 UPLC-UV/MS.. 42
4.6 HR-MS.. 42
4.7 Intact protein MS 42
4.8 Protein concentration determination 42
4.9 Analytical hydrophilic interaction chromatography (HIC) 43
4.10 Analytical hydrophilic interaction chromatography (SEC) 43
4.11 LC-MS/MS.. 43
5. Experimental Procedures – Organic Synthesis 44
6. Experimental Procedures – Physical Organic Chemistry.. 59
6.1 2-D NMR assay for pK_a determination 59
6.2 FRET assay to determine reaction rates 75
6.3 FRET assay to determine aryl thiol-ETP/Maleimide conjugate stability 88
7. Experimental Procedures – Biochemistry & Bioconjugation.. 90
7.1 SUMO-TTL expression and purification 90
7.2 Tub-tag® labeling of 5(6)-CF-Tub-tag® peptide 7 91
7.3 Construction of pET22- pelB-His10-GBP1-C-Tub-tag® plasmid.. 102
7.4 Expression and purification of GBP1-C-Tub-tag® (26).. 104
7.5 Bioconjugation experiments with GBP1-C-Tub-tag® (26) 105
7.6 Validation of selectivity for aryl thiolate – ETP reaction 111
7.7 Fluorescence bleaching experiments.. 112
7.8 Bioconjugation experiments with Brentuximab-LC-Tub-tag® (34).. 114
7.9 CD30 bystander cell killing assays.. 130
8. LC-MS spectra [Organic Synthesis] 142
LC-MS spectrum of 1 [Crude reaction mixture] 143
LC-MS spectrum of 2 [Crude reaction mixture] 144
LC-MS spectrum of 3_{ab} 145
LC-MS spectrum of 4 [Crude reaction mixture] 146
LC-MS spectrum of 5 [Crude reaction mixture] 147

LC-MS spectra of 6 148
LC-MS spectrum of 7 150
LC-MS spectrum of 9 151
LC-MS spectrum of 10-Z/E 152
LC-MS spectrum of 11-Z 153
LC-MS spectrum of 17 154
LC-MS spectrum of 13 155
LC-MS spectrum of 19 156
LC-MS spectrum of 15 157
LC-MS spectrum of 16 158
LC-MS spectrum of 20 [Crude reaction mixture] 159
LC-MS spectrum of 21 160
LC-MS spectrum of 23 161
LC-MS spectrum of 24 162
LC-MS spectrum of 29 163
LC-MS spectrum of 30 164
LC-MS spectrum of 31 165
LC-MS spectrum of 35 166
LC-MS spectrum of 36 167
9. NMR spectra [Organic Synthesis] 168
^{1}H, ^{13}C, ^{1}H-COSY, (^{1}H,^{13}C)-HSQC, (^{1}H,^{13}C)-HMBC spectra of 3_{ab} (DMSO-d_6 – 310K) 169
^{1}H, ^{13}C-APT, ^{1}H-COSY and (^{1}H,^{13}C)-HSQC spectra of 6 (D_2O) 174
^{1}H, ^{13}C-APT, ^{1}H-COSY and (^{1}H,^{13}C)-HSQC spectra of 6 (DMSO-d_6) 176
^{1}H and ^{31}P spectra of ethyl diethynyl phosphinate[1,2] ($CDCL_3$) 178
^{1}H, ^{1}H (^{31}P-decoupled), ^{1}H-COSY, ^{31}P, (^{1}H,^{31}P)-HMBC, ^{13}C, (^{1}H,^{13}C)-HSQC, (^{1}H,^{13}C)-HMQC and (^{1}H,^{13}C)-HMBC spectra of 10-Z/E (DMSO-d_6) 179
^{1}H, ^{31}P, (^{1}H,^{31}P)-HMBC spectra of 10-Z (D_2O) 184
^{1}H, ^{1}H (^{31}P-decoupled), ^{1}H-COSY, ^{31}P, (^{1}H,^{31}P)-HMBC spectra of 11-Z (DMSO-d_6) 186
^{1}H, ^{13}C-APT, ^{1}H-COSY and (^{1}H,^{13}C)-HSQC spectra of 17 (DMSO-d_6) 189
^{1}H, ^{13}C, ^{1}H-COSY and (^{1}H,^{13}C)-HSQC spectra of 13 (DMSO-d_6) 192
^{1}H and ^{13}C spectra of 19 (DMSO-d_6) 196
^{1}H, ^{13}C, ^{31}P and (^{1}H,^{13}C)-HSQC spectra of 15 (DMSO-d_6) 197
^{1}H, ^{13}C-APT, ^{1}H-COSY and (^{1}H,^{13}C)-HSQC spectra of 21 (MeOD) 199
^{1}H, ^{13}C, ^{1}H-COSY and (^{1}H,^{13}C)-HSQC spectra of 23 (DMSO-d_6) 200
^{1}H, ^{13}C, ^{1}H-COSY and (^{1}H,^{13}C)-HSQC spectra of 30 (DMSO-d_6) 203
^{1}H, ^{13}C, ^{31}P, ^{1}H-COSY and (^{1}H,^{13}C)-HSQC spectra of 31 (DMSO-d_6) 206

10. Supporting References 210

1. Supplementary Figures

Figure S1

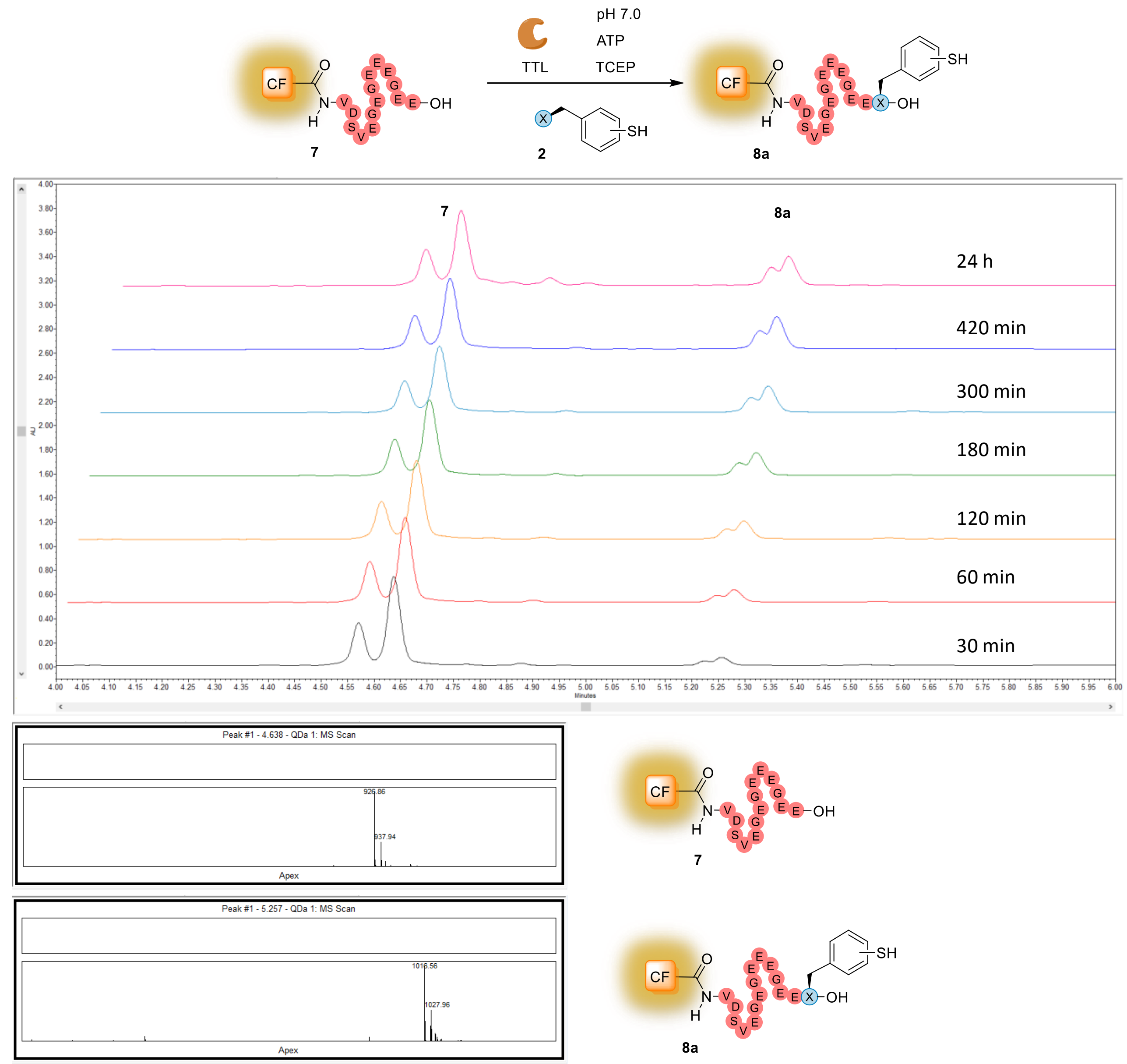

Figure S1. Overlay of UV chromatograms (λ = 220 nm, 4.0 – 6.0 min) for the chemoenzymatic ligation of **7** to form **8a** using **2** (from disulfide **3_{ab}**). Crude reaction mixture samples (25 µL) were quenched with 1% TFA (25 µL) at indicated reaction times (30, 60, 120, 180, 300, 420 min and 24 h) and analyzed using LC-MS (C18, linear gradient 5 → 95% MeCN in H_2O, 0.1% TFA, 15 min, 5 µL injection). The m/z values for **7** (R_t (min): 4.64 (ESI-MS (m/z): 926.86 (M + $2H^{2+}$), 937.94 (M + H + Na^{2+})) and **8a** (R_t (min): 5.26 (ESI-MS (m/z): 1016.56 (M + $2H^{2+}$), 1027.96 (M + H + Na^{2+})) were shown. The data shown is a representative dataset (N = 1). Quantification of the dataset shown here is presented in SI section 7.2.

Figure S2

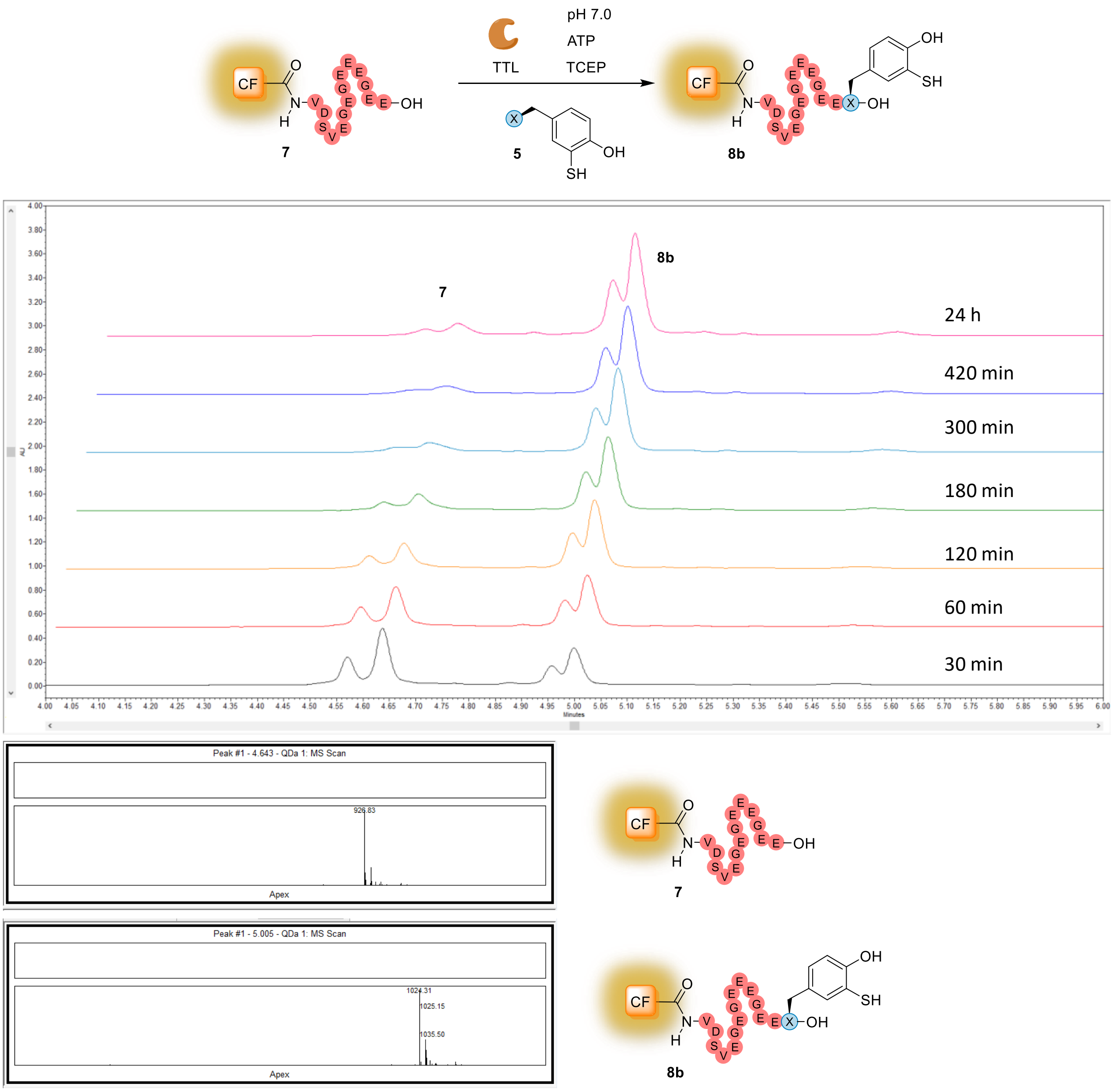

Figure S2. Overlay of UV chromatograms (λ = 220 nm, 4.0 – 6.0 min) for the chemoenzymatic ligation of **7** to form **8b** using **5** (from disulfide **6**). Crude reaction mixture samples (25 µL) were quenched with 1% TFA (25 µL) at indicated reaction times (30, 60, 120, 180, 300, 420 min and 24 h) and analyzed using LC-MS (C18, linear gradient 5 → 95% MeCN in H2O, 0.1% TFA, 15 min, 5 µL injection). The m/z values for **7** (Rt (min): 4.64 (ESI-MS (m/z): 926.83 (M + $2H^{2+}$)) and **8b** (Rt (min): 5.00 (ESI-MS (m/z): 1024.31 (M + $2H^{2+}$), 1035.50 (M + H + Na^{2+})) were shown. The data shown is a representative dataset (N = 1). Quantification of the dataset shown here is presented in SI section 7.2.

Figure S3

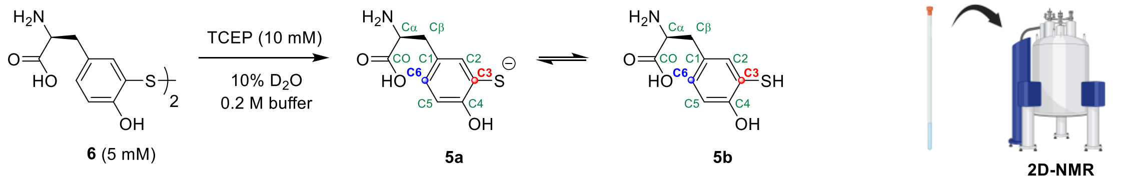

pH	δ_{exp}	$-(\delta_{exp} - \delta_{HCl})$
1,0000	131,20	0,00
4,5000	130,65	0,55
5,0000	129,98	1,22
5,5000	128,98	2,22
6,0000	128,10	3,10
6,5000	127,27	3,93
7,0000	127,01	4,19
8,0000	126,83	4,37
13,000	126,94	4,26

$$\delta_{exp} = \frac{\delta_{HCl} + \delta_{NaOH} * 10^{(pH - pKa)}}{1 + 10^{(pH - pKa)}}$$

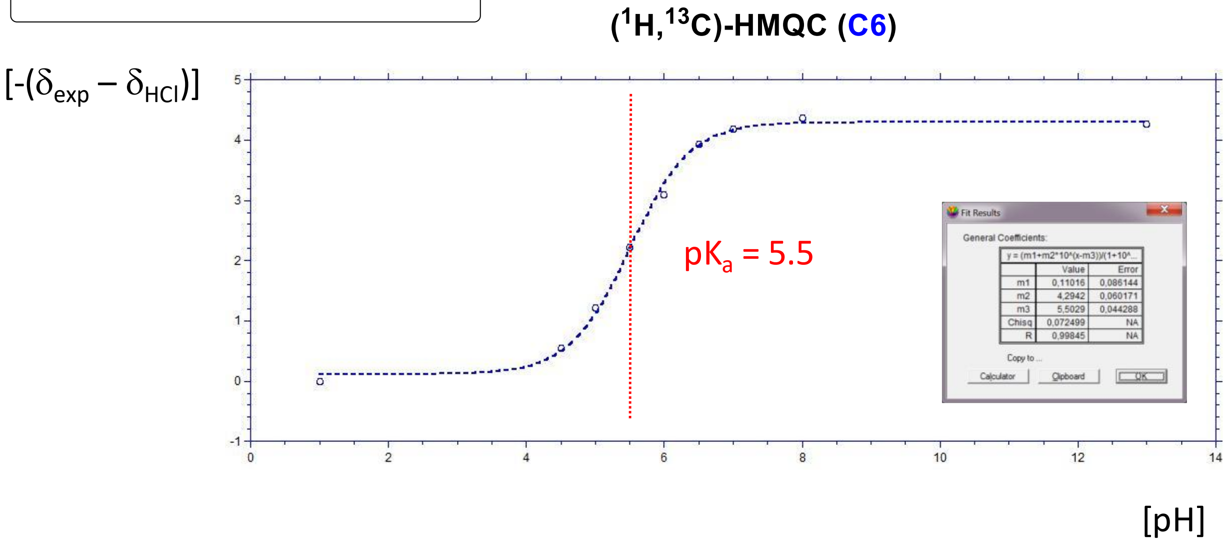

Figure S3. 2-D NMR analysis to determine the pK_a value of the thiol moiety of **5**. Disulfide **6** (5 mM) was treated with TCEP (10 mM) in 0.2 M citrate (pH 4.5 – 6.0) and phosphate (pH 6.0 – 8.0) buffers containing 10% D_2O. Highly acidic (pH 1.0) and basic (pH 13.0) reference samples were obtained using 1% HCl (w/w) and 1% NaOH (w/w), respectively. For this analysis, the signal for carbon 6 (C6) measured in (^{1}H-^{13}C)-HMQC was used. The difference between the chemical shift at pH 1.0 and the individual measurements was used to calculate the pK_a. Complete datasets used and further experimental details are presented in SI section 6.1.

Figure S4

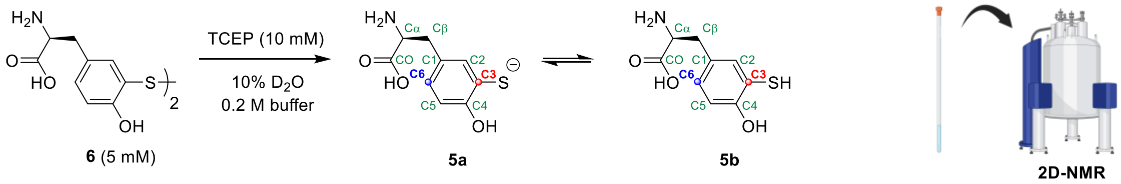

pH	δ_{exp}	$(\delta_{exp} - \delta_{HCl})$
1,0000	121,47	0,0000
4,5000	122,78	1,3100
5,0000	124,91	3,4400
5,5000	128,50	7,0300
6,0000	131,53	10,060
6,5000	133,67	12,200
7,0000	134,60	13,130
8,0000	135,06	13,590
13,000	134,69	13,220

$$\delta_{exp} = \frac{\delta_{HCl} + \delta_{NaOH} * 10^{(pH - pKa)}}{1 + 10^{(pH - pKa)}}$$

(^{1}H,^{13}C)-HMBC (C3)

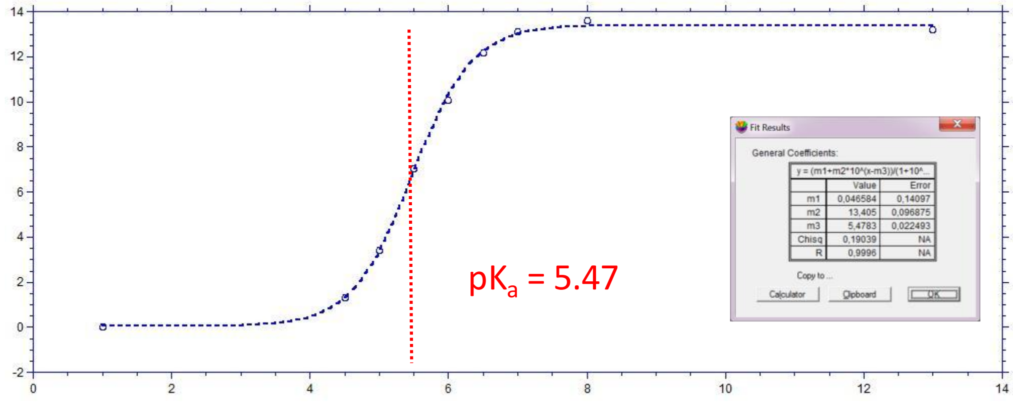

[pH]

Figure S4. 2-D NMR analysis to determine the pK_a value of the thiol moiety of **5**. Disulfide **6** (5 mM) was treated with TCEP (10 mM) in 0.2 M citrate (pH 4.5 – 6.0) and phosphate (pH 6.0 – 8.0) buffers containing 10% D2O. Highly acidic (pH 1.0) and basic (pH 13.0) reference samples were obtained using 1% HCl (w/w) and 1% NaOH (w/w), respectively. For this analysis, the signal for carbon 3 (C3) measured in (1H-13C)-HMBC was used. More specifically, the 2D intersection of H5 -> C3. The difference between the chemical shift at pH 1.0 and the individual measurements was used to calculate the pK_a. Complete datasets used and further experimental details are presented in SI section 6.1.

Figure S5

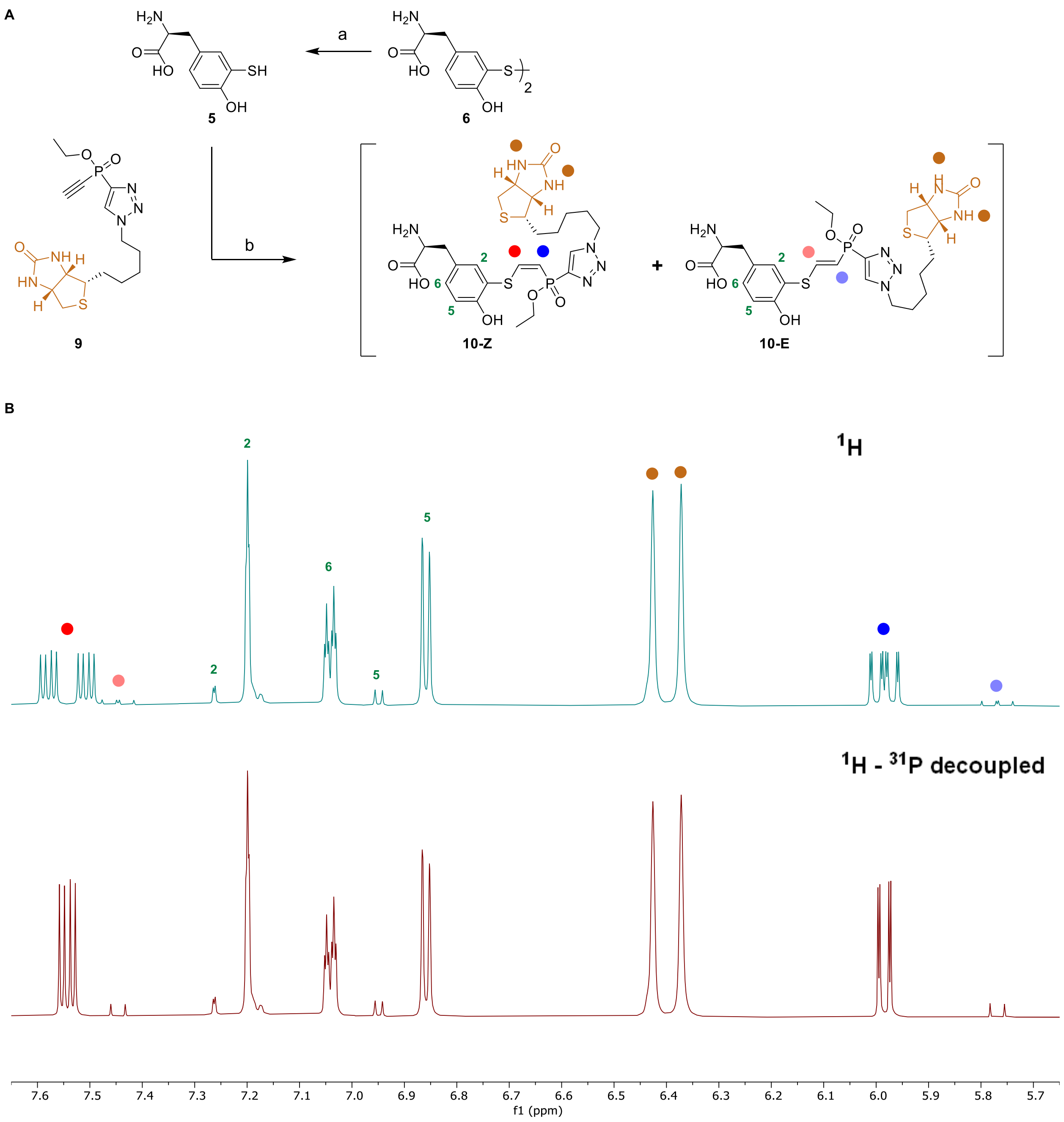

Figure S5. (A) Reaction between **5** (from **6** upon TCEP liberation) and ETP-biotin **9** to form conjugate **10** as an E/Z mixture (**10-Z** / **10-E**). Reagents/conditions: (a) TCEP (4.0 eq), 0.2 M phosphate buffer pH 6.0, MeCN, rt, 2 min; (b) **9** (2.03 eq), DMSO, rt, 10 min, 43% (**10-Z** + **10-E** mixture; ≥ 90% **10-Z**). (B) NMR analysis of **10-Z** + **10-E** mixture (7.65 – 5.65 ppm, ^{1}H and ^{1}H with ^{31}P decoupling), indicating key hydrogen atom signals. **Figure S6** and **Figure S7** provide in-depth analysis of the alkene ^{1}H NMR signals.

Figure S6

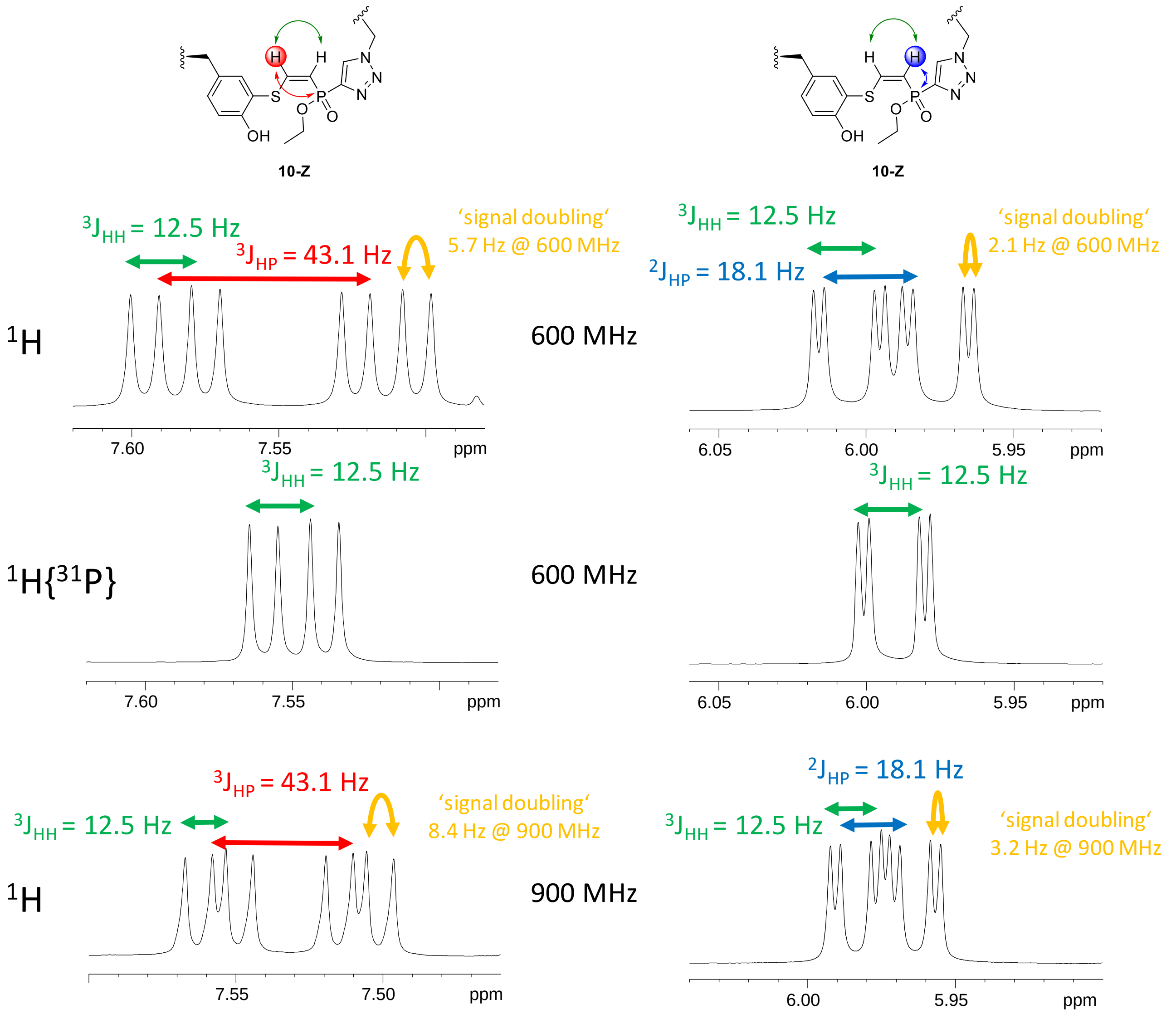

Figure S6. Alkene ^{1}H NMR signals (600 MHz, 600 MHz with ^{31}P decoupling and 900 MHz) observed for conjugate **10-Z**. For **10-Z**, an additional 'signal doubling' was observed beyond the expected $^{3}J_{HH}$ (green) and $^{3}J_{HP}$ (red/blue) couplings. The possibility of a third J-coupling for the 'signal doubling' was ruled out because the perceived 'signal doubling' changes when shifting from 600 MHz to 900 MHz (by a factor of 1.5). We propose that the observed signals represent the 2 diastereoisomers (due to the racemic phosphorus atom found in reagent **9**).

Figure S7

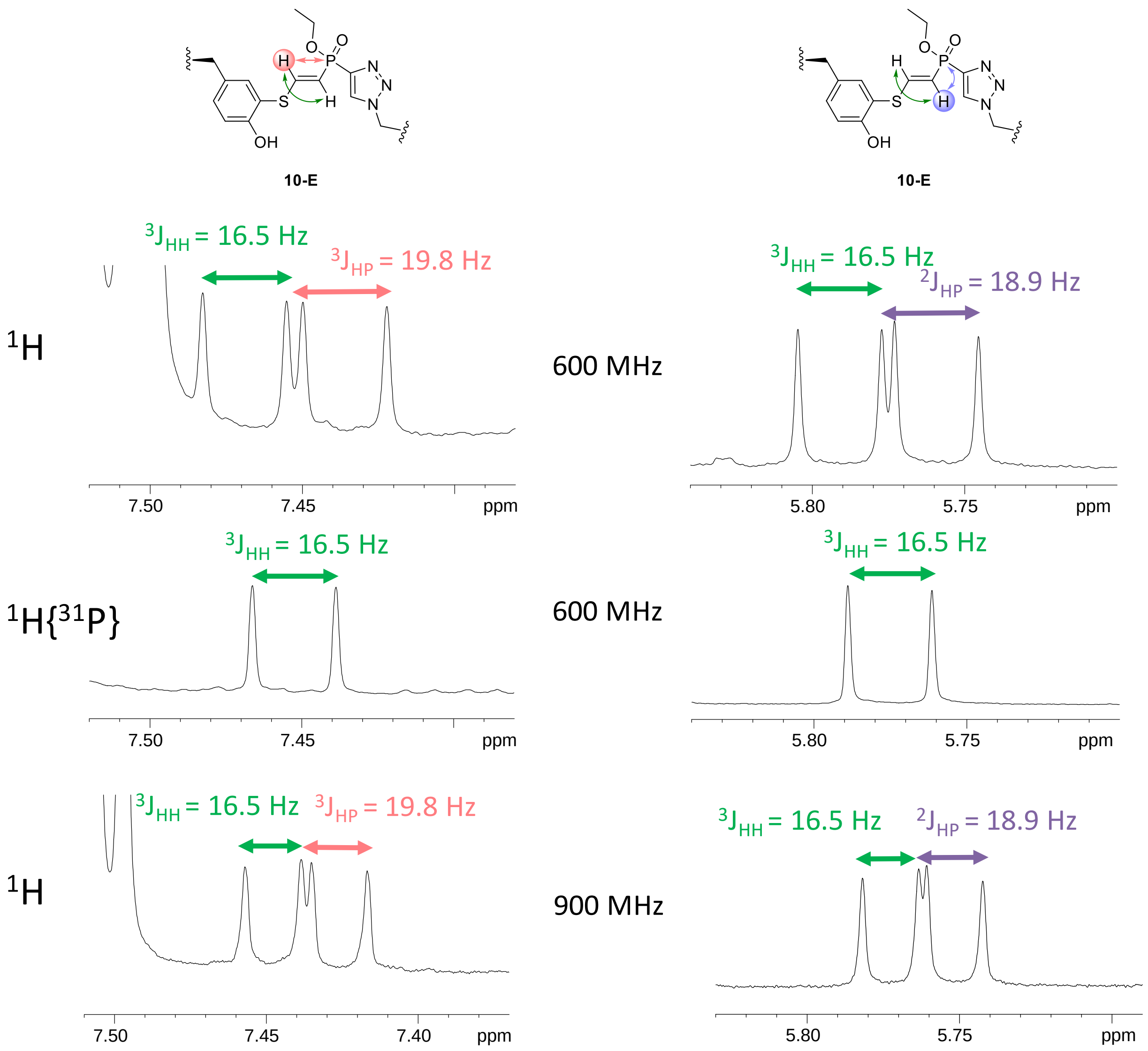

Figure S7. Alkene ^1H NMR signals (600 MHz, 600 MHz with ^{31}P decoupling and 900 MHz) observed for conjugate **10-E**. For **10-E**, the predicted $^3J_{HH}$ (green) and $^3J_{HP}$ (pink/purple) couplings are present.

Figure S8

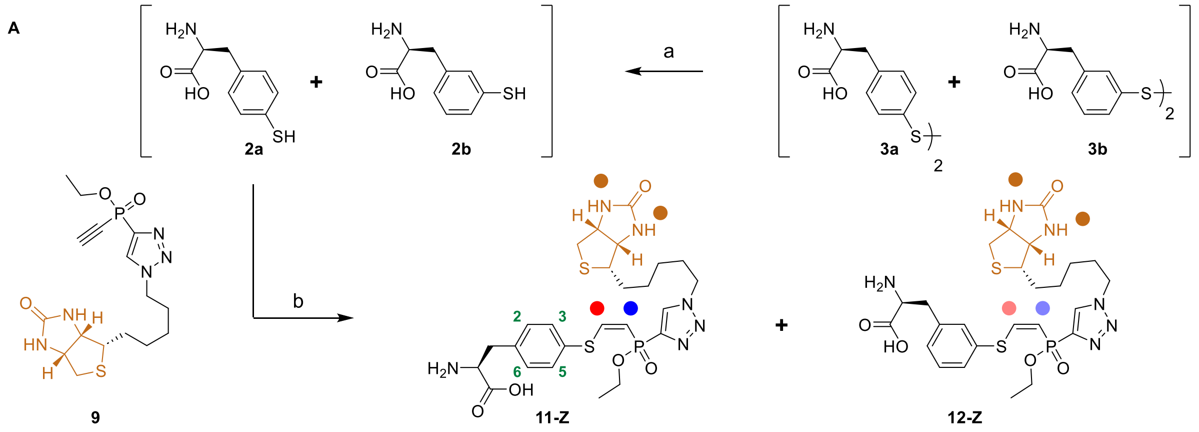

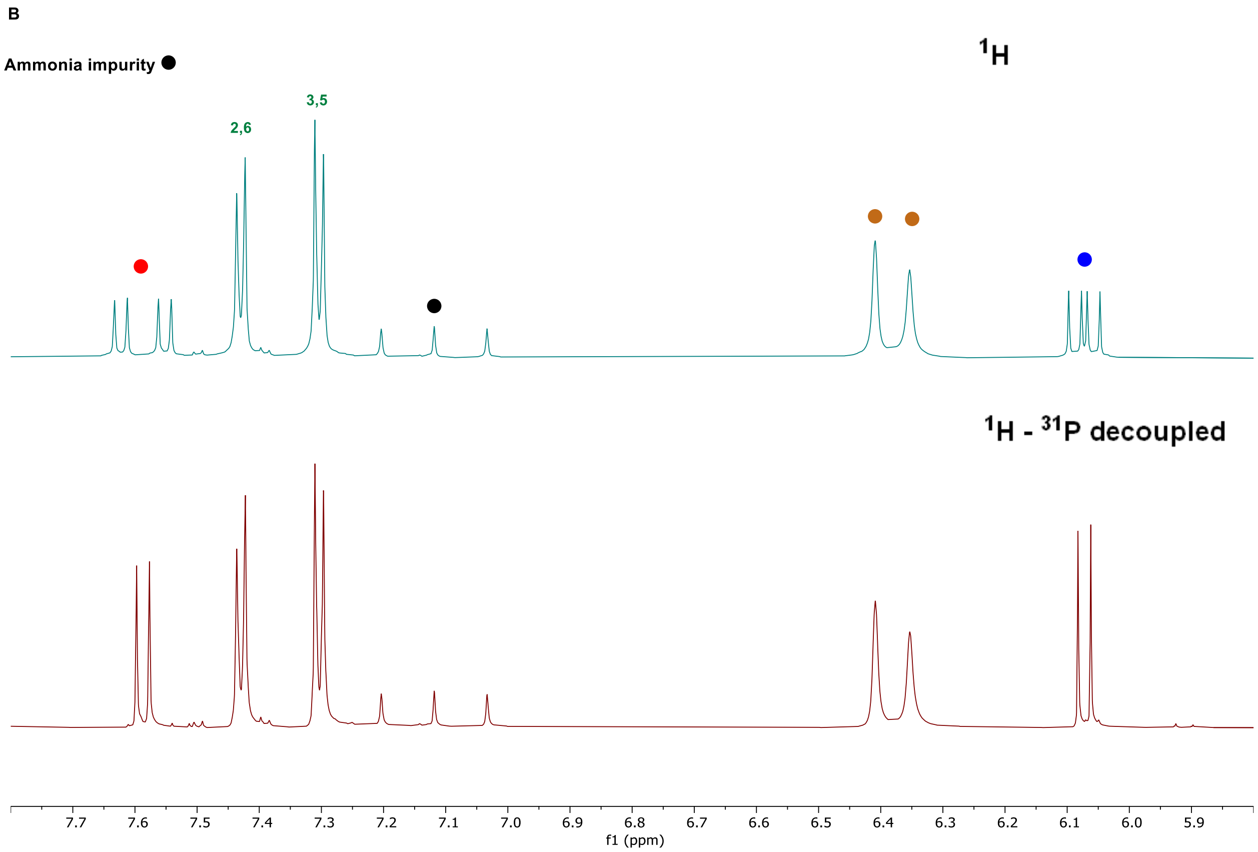

Figure S8. (A) Reaction between **2ab** (from **3ab** upon TCEP liberation) and ETP-biotin **9** to form conjugates **11-Z** and **12-Z**. Reagents/conditions: (a) TCEP (4.0 eq), 0.2 M phosphate buffer pH 7.0, MeCN, DMSO, rt, 2 min; (b) **9** (2.0 eq), DMSO, rt, 15 min, 23% (**11-Z**), 6% (**11-Z** + **12-Z**). (B) NMR analysis of **11-Z** (7.8 – 5.8 ppm, ^{1}H and ^{1}H with ^{31}P decoupling), indicating key hydrogen atom signals.

Figure S9

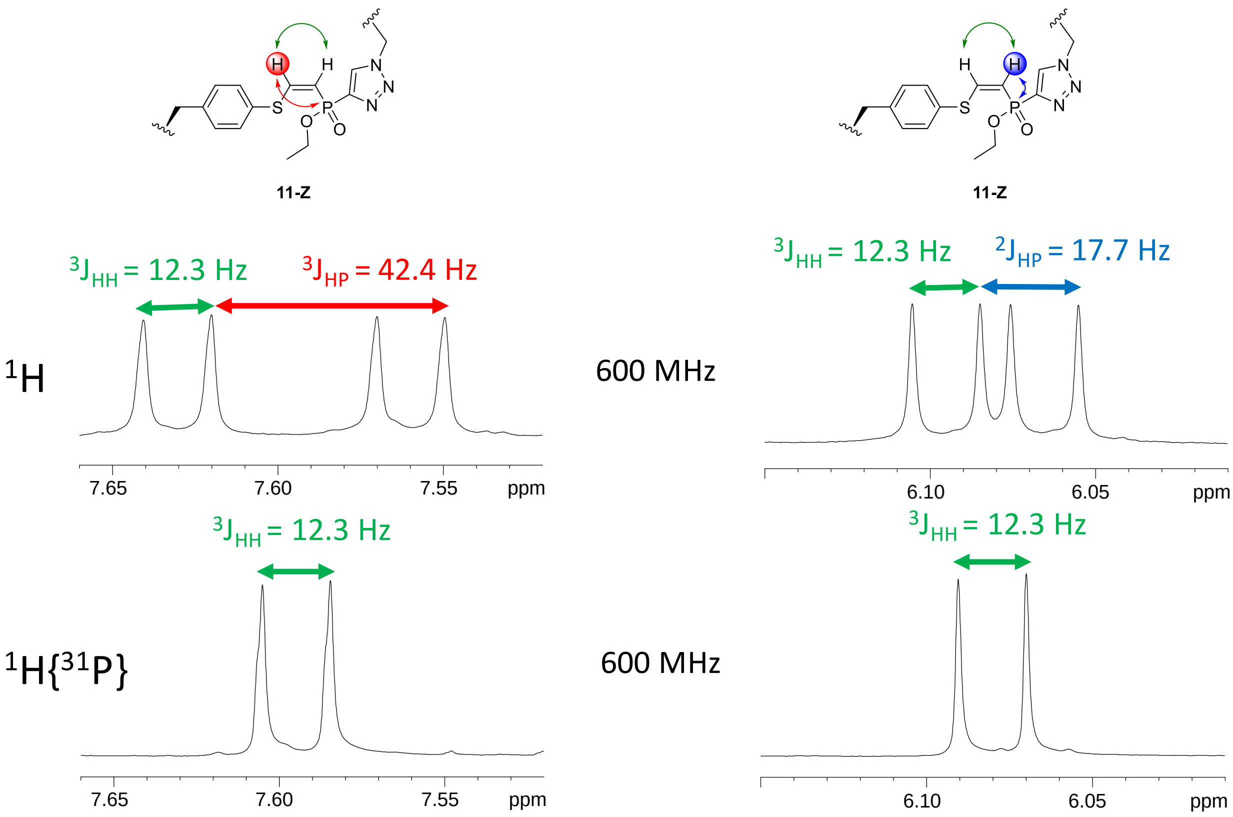

Figure S9. Alkene ^{1}H NMR signals (600 MHz and 600 MHz with ^{31}P decoupling) observed for conjugate **11-Z**. For **11-Z**, the predicted $^{3}J_{HH}$ (green) and $^{3}J_{HP}$ (red/blue) couplings are present.

Figure S10

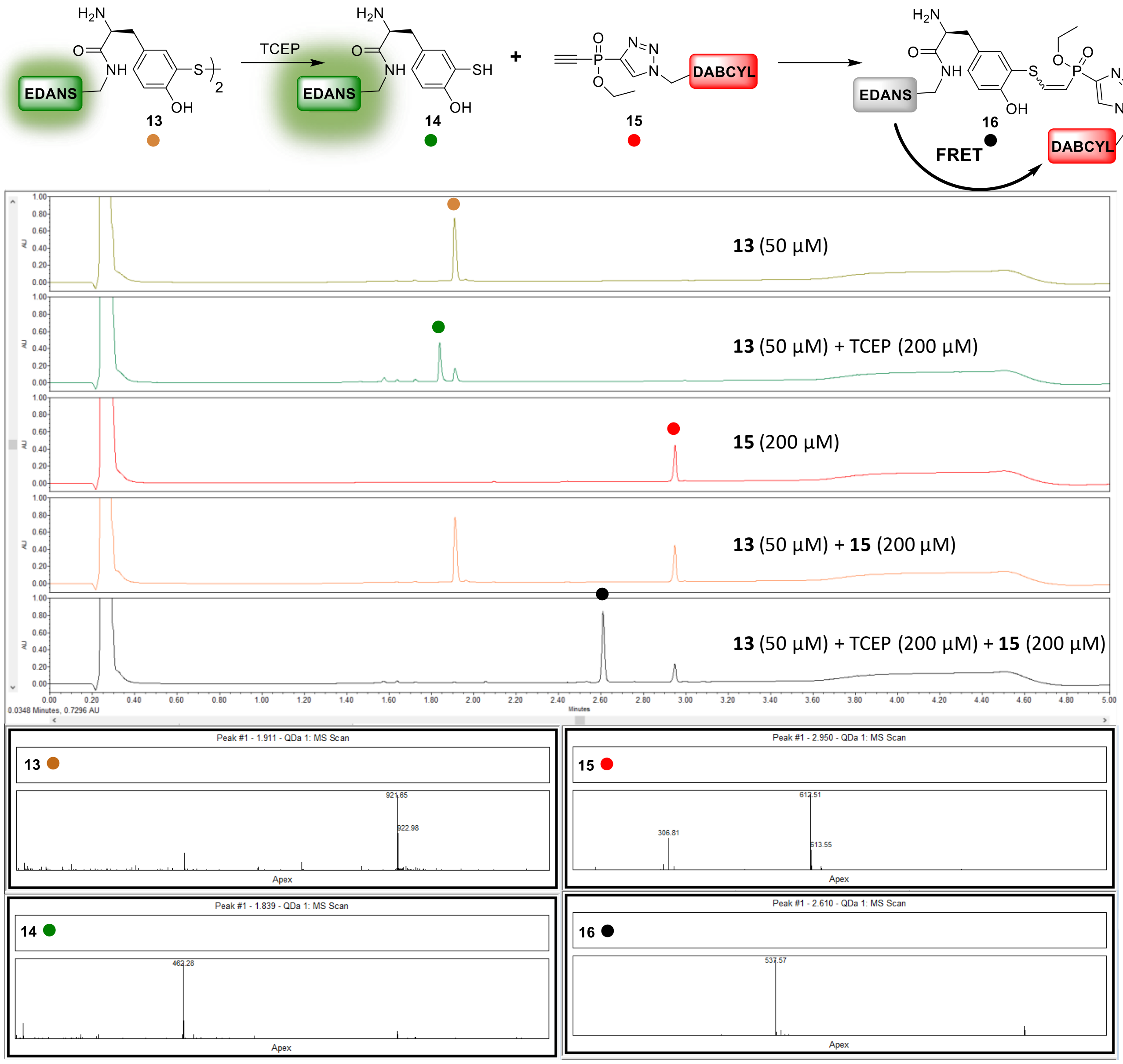

Figure S10. Preliminary experiment to establish aryl thiol – ETP reactivity at 100 µM of aryl thiol **14** using two molar equivalents of DABCYL-ETP **15**. Aryl disulfide **13** (50 µM) was treated with TCEP (200 µM). After mixing the reaction volume, DABCYL-ETP **15** (200 µM) was added and the reaction (100 µL volume, PBS with 5% DMSO) was allowed to proceed at room temperature under periodic shaking. After 120 min, a 50 µL sample was quenched (50 µL of 1% TFA) and 5 µL was injected for LC-MS analysis (linear gradient 5 → 60% MeCN, 0.1% TFA, 5 min). Reference solutions were analyzed in the same way after 120 min: **13** (50 µM), **13** (50 µM) + TCEP (200 µM), **15** (200 µM), **13** (50 µM) + **15** (200 µM). The mass traces of the main signals are shown: **13**: R_t (min): 1.91 (ESI-MS (m/z): 921.65 (M+H$^+$)); **14**: R_t (min): 1.84 (ESI-MS (m/z): 462.28 (M+H$^+$)); **15**: R_t (min): 2.95 (ESI-MS (m/z): 306.81 (M+2H^{2+}), 612.51 (M+H$^+$)); **16**: R_t (min): 2.61 (ESI-MS (m/z): 537.57 (M+2H^{2+})).

Figure S11

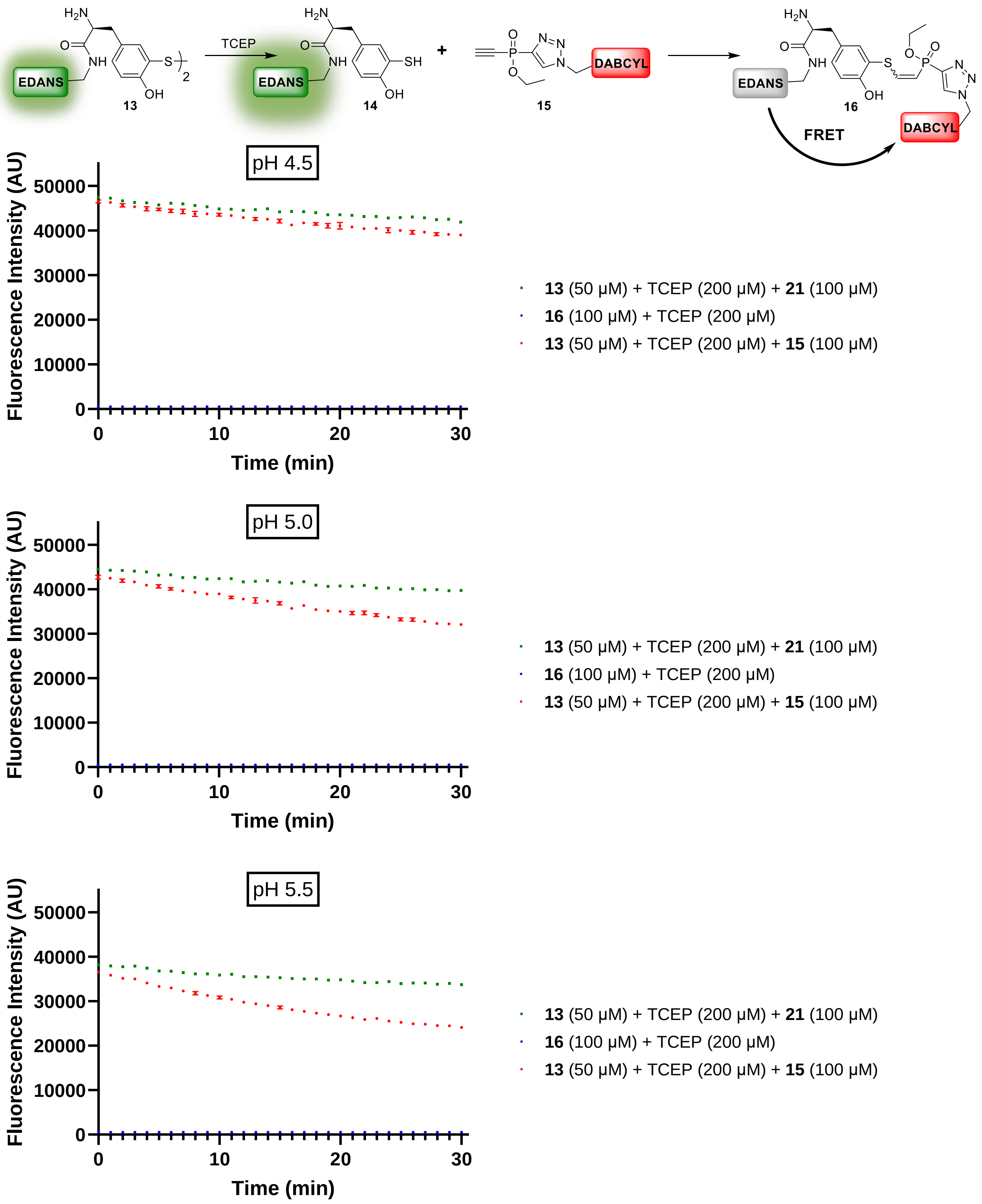

Figure S11. Raw fluorescence data (λex = 340 nm, λem = 495 nm) obtained for the reaction between EDANS aryl thiol **14** and DABCYL-ETP **15** at pH 4.5 – 5.5 (0.2 M citrate buffer, 10% MeCN, 5% DMSO). Displayed are the reaction (**13** + **15** + TCEP) and the controls for 100% (**13** + **21** + TCEP) and 0% (**16** + TCEP) fluorescence. These datasets were recorded together in a single experiment (N = 1, n = 2).

Figure S12

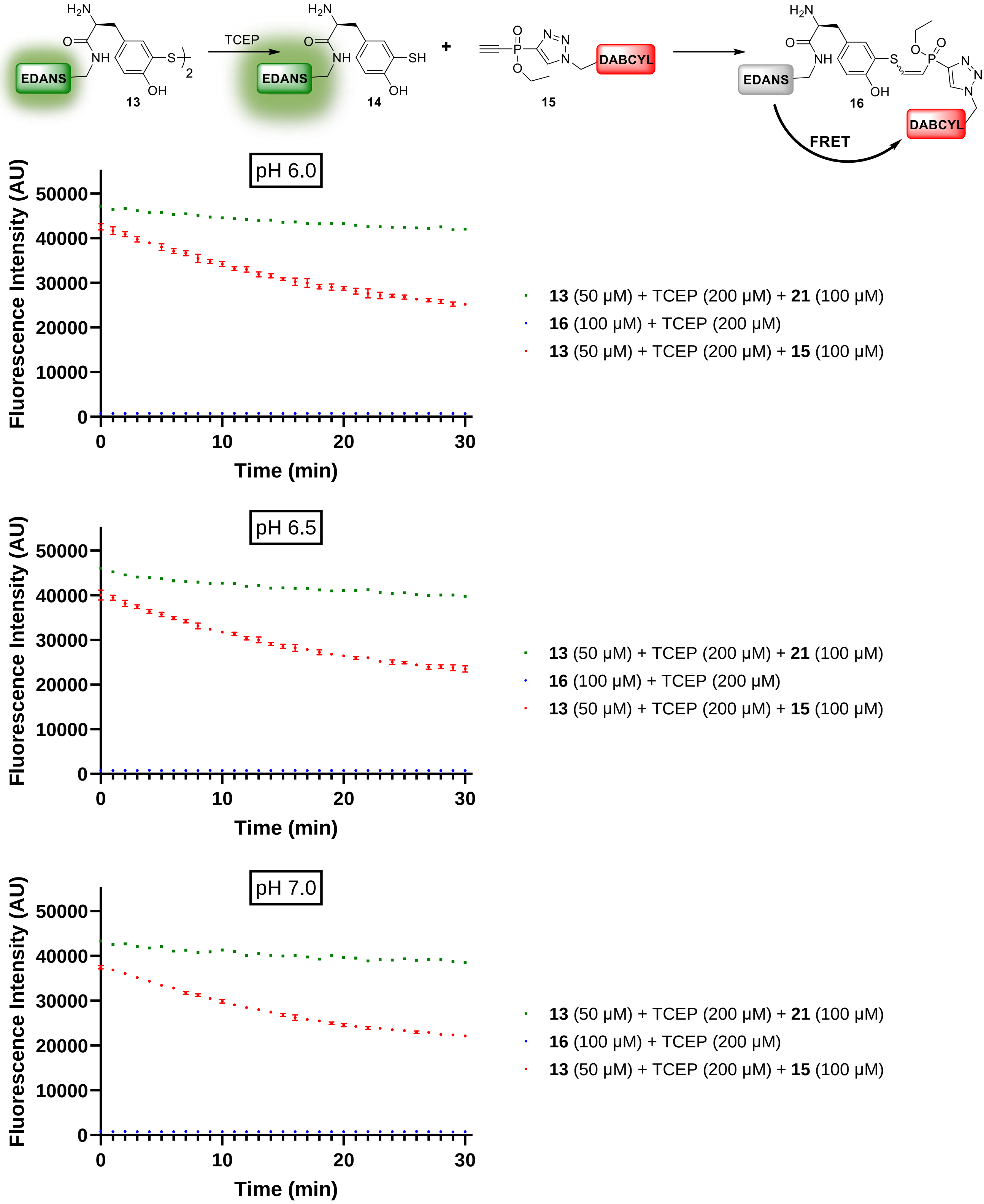

Figure S12. Raw fluorescence data (λex = 340 nm, λem = 495 nm) obtained for the reaction between EDANS aryl thiol **14** and DABCYL-ETP **15** at pH 6.0 – 7.0 (0.2 M phosphate buffer, 10% MeCN, 5% DMSO). Displayed are the reaction (**13** + **15** + TCEP) and the controls for 100% (**13** + **21** + TCEP) and 0% (**16** + TCEP) fluorescence. These datasets were recorded together in a single experiment (N = 1, n = 2).

Figure S13

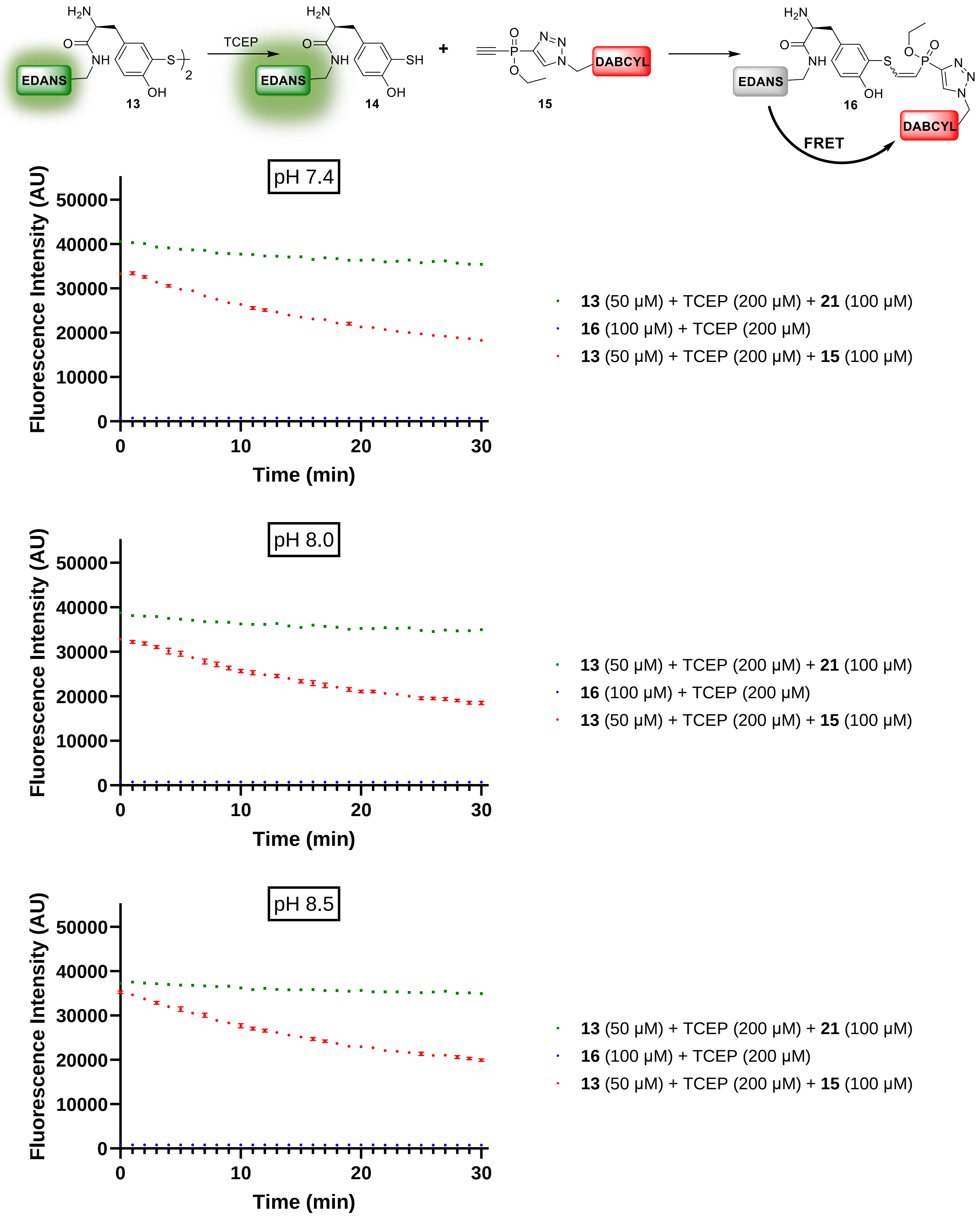

Figure S13. Raw fluorescence data (λex = 340 nm, λem = 495 nm) obtained for the reaction between EDANS aryl thiol **14** and DABCYL-ETP **15** at pH 7.4 – 8.5 (0.2 M phosphate buffer / 0.2 M tris buffer, 10% MeCN, 5% DMSO). Displayed are the reaction (**13** + **15** + TCEP) and the controls for 100% (**13** + **21** + TCEP) and 0% (**16** + TCEP) fluorescence. These datasets were recorded together in a single experiment (N = 1, n = 2).

Figure S14

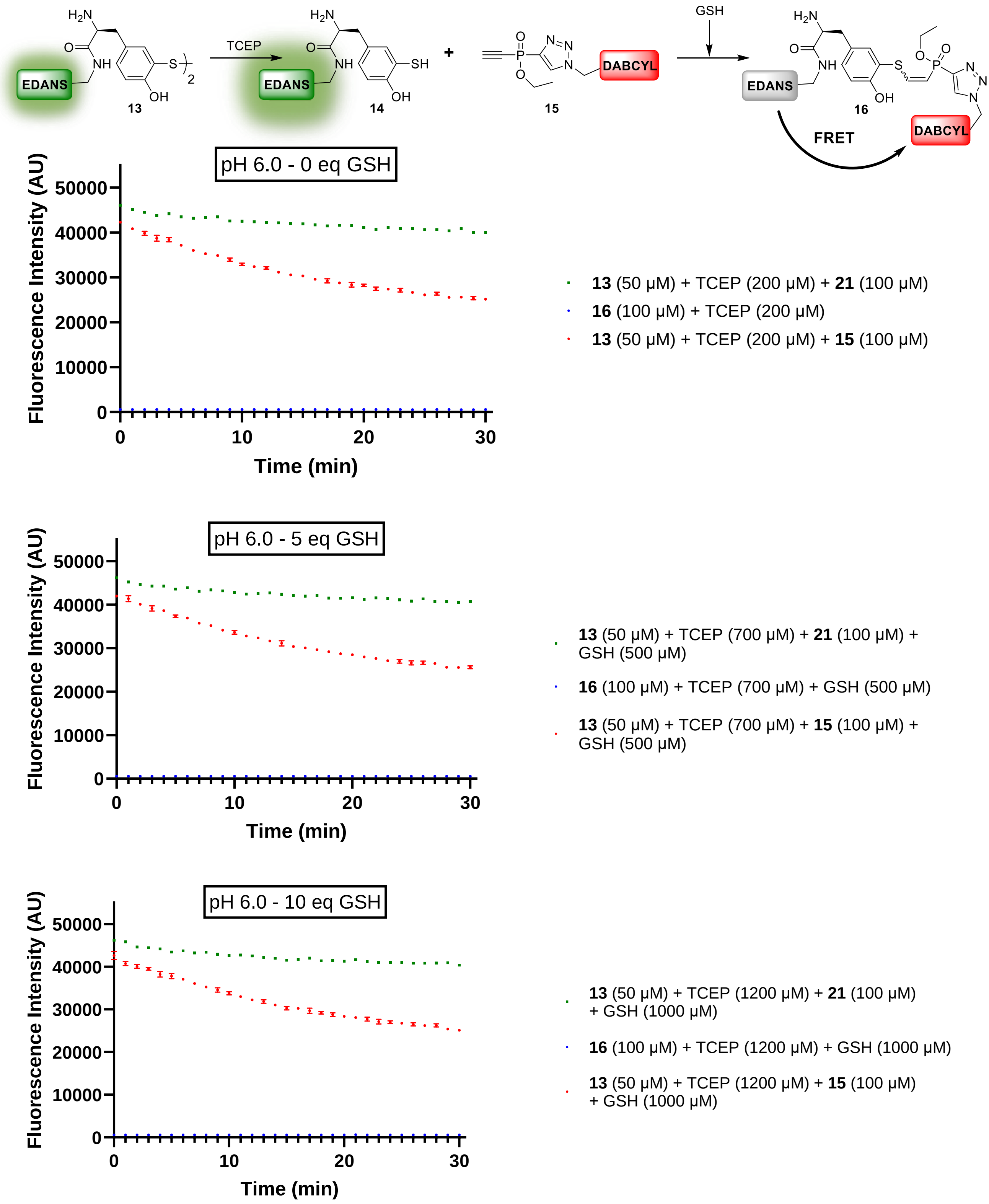

Figure S14. Raw fluorescence data (λex = 340 nm, λem = 495 nm) obtained for the reaction between EDANS aryl thiol **14** and DABCYL-ETP **15** at pH 6.0 (0.2 M phosphate buffer, 10% MeCN, 5% DMSO) with gluthathione (GSH) competition. Displayed are the reaction (**13** + **15** + TCEP) and the controls for 100% (**13** + **21** + TCEP) and 0% (**16** + TCEP) fluorescence with 0 / 5 / 10 equivalents of GSH competition. These datasets were recorded together in a single experiment (N = 1, n = 2).

Figure S15

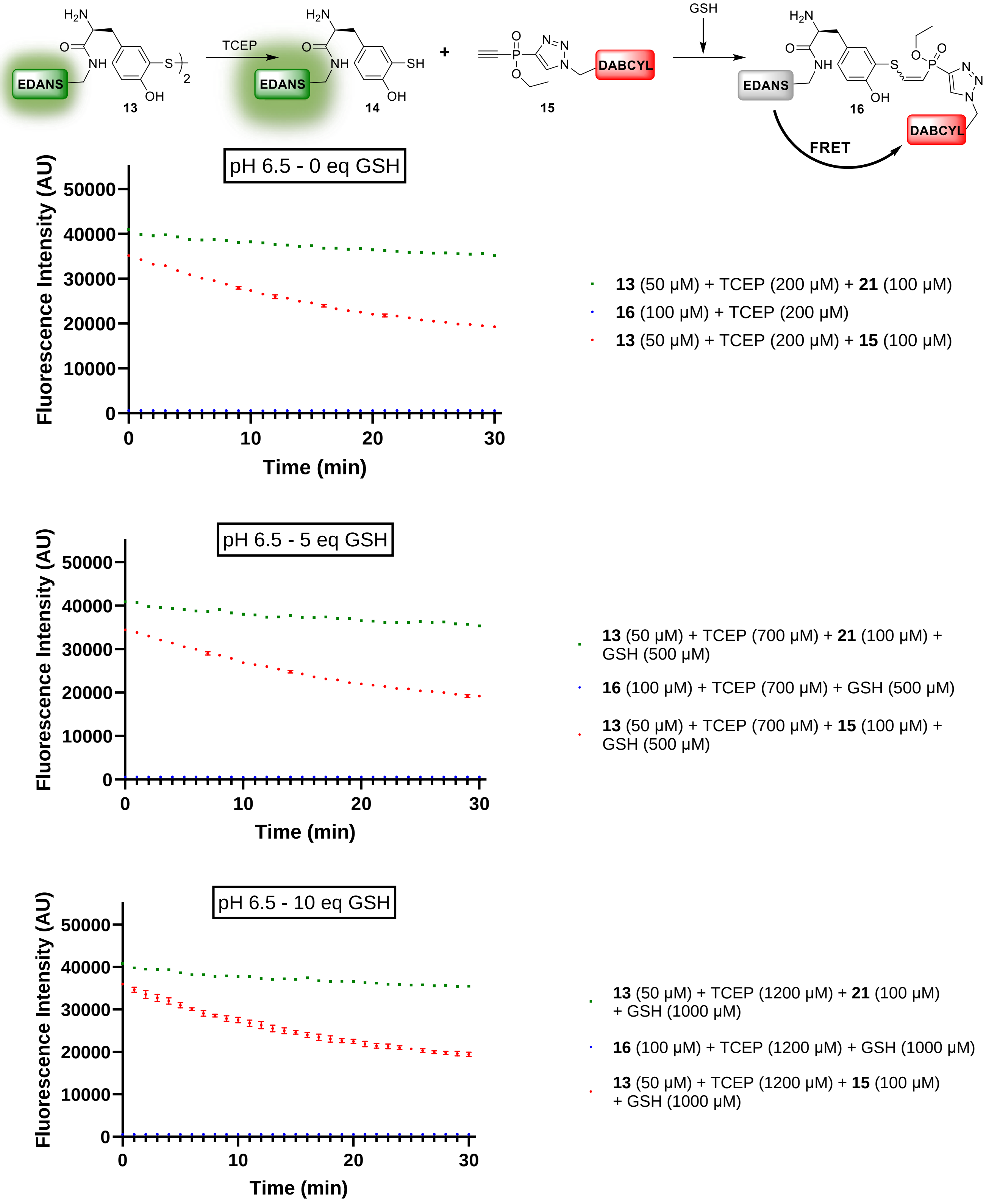

Figure S15. Raw fluorescence data (λex = 340 nm, λem = 495 nm) obtained for the reaction between EDANS aryl thiol **14** and DABCYL-ETP **15** at pH 6.5 (0.2 M phosphate buffer, 10% MeCN, 5% DMSO) with gluthathione (GSH) competition. Displayed are the reaction (**13** + **15** + TCEP) and the controls for 100% (**13** + **21** + TCEP) and 0% (**16** + TCEP) fluorescence with 0 / 5 / 10 equivalents of GSH competition. These datasets were recorded together in a single experiment (N = 1, n = 2).

Figure S16

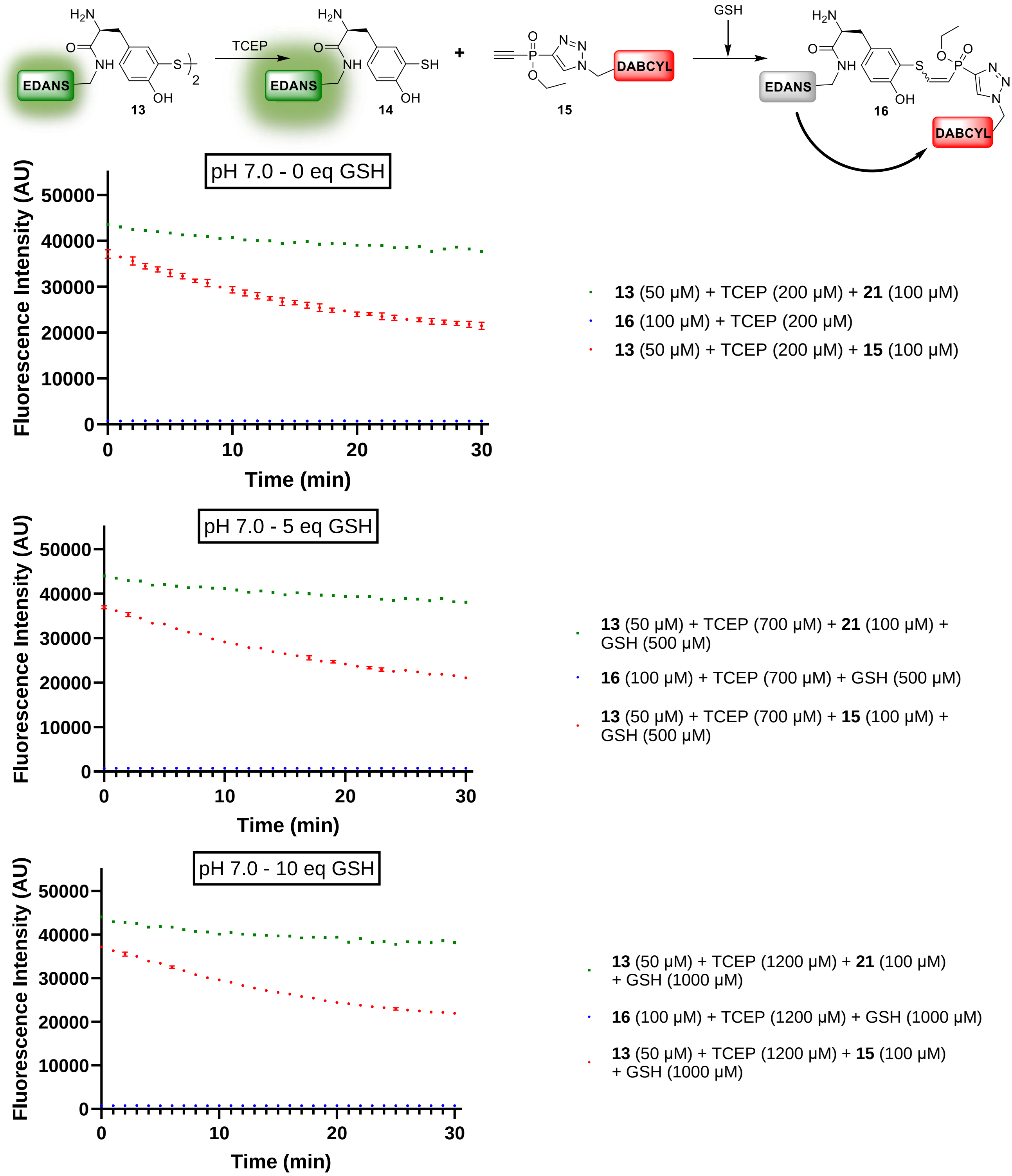

Figure S16. Raw fluorescence data (λex = 340 nm, λem = 495 nm) obtained for the reaction between EDANS aryl thiol **14** and DABCYL-ETP **15** at pH 7.0 (0.2 M phosphate buffer, 10% MeCN, 5% DMSO) with gluthathione (GSH) competition. Displayed are the reaction (**13** + **15** + TCEP) and the controls for 100% (**13** + **21** + TCEP) and 0% (**16** + TCEP) fluorescence with 0 / 5 / 10 equivalents of GSH competition. These datasets were recorded together in a single experiment (N = 1, n = 2).

Figure S17

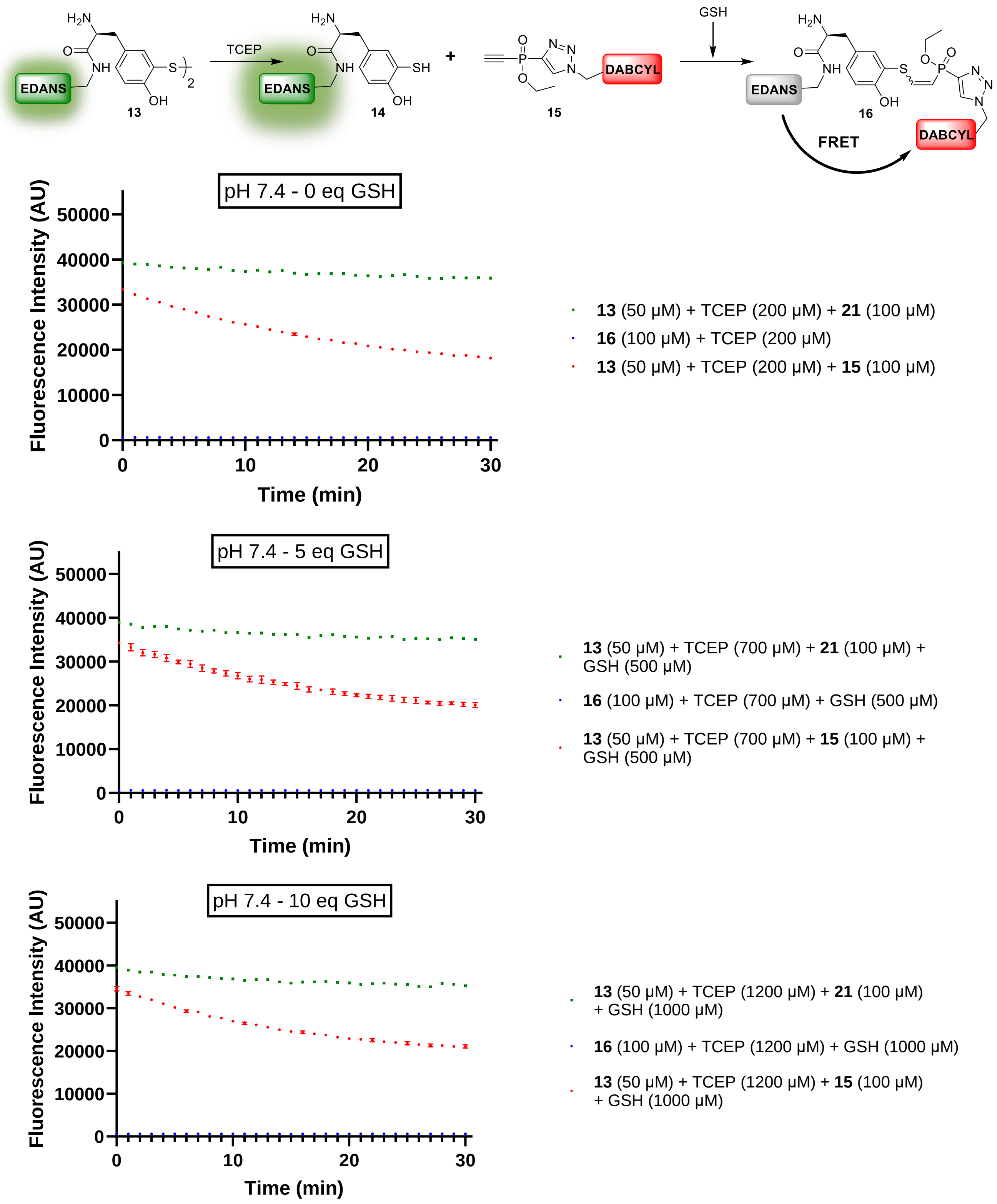

Figure S17. Raw fluorescence data (λex = 340 nm, λem = 495 nm) obtained for the reaction between EDANS aryl thiol **14** and DABCYL-ETP **15** at pH 7.4 (0.2 M phosphate buffer, 10% MeCN, 5% DMSO) with gluthathione (GSH) competition. Displayed are the reaction (**13** + **15** + TCEP) and the controls for 100% (**13** + **21** + TCEP) and 0% (**16** + TCEP) fluorescence with 0 / 5 / 10 equivalents of GSH competition. These datasets were recorded together in a single experiment (N = 1, n = 2).

Figure S18

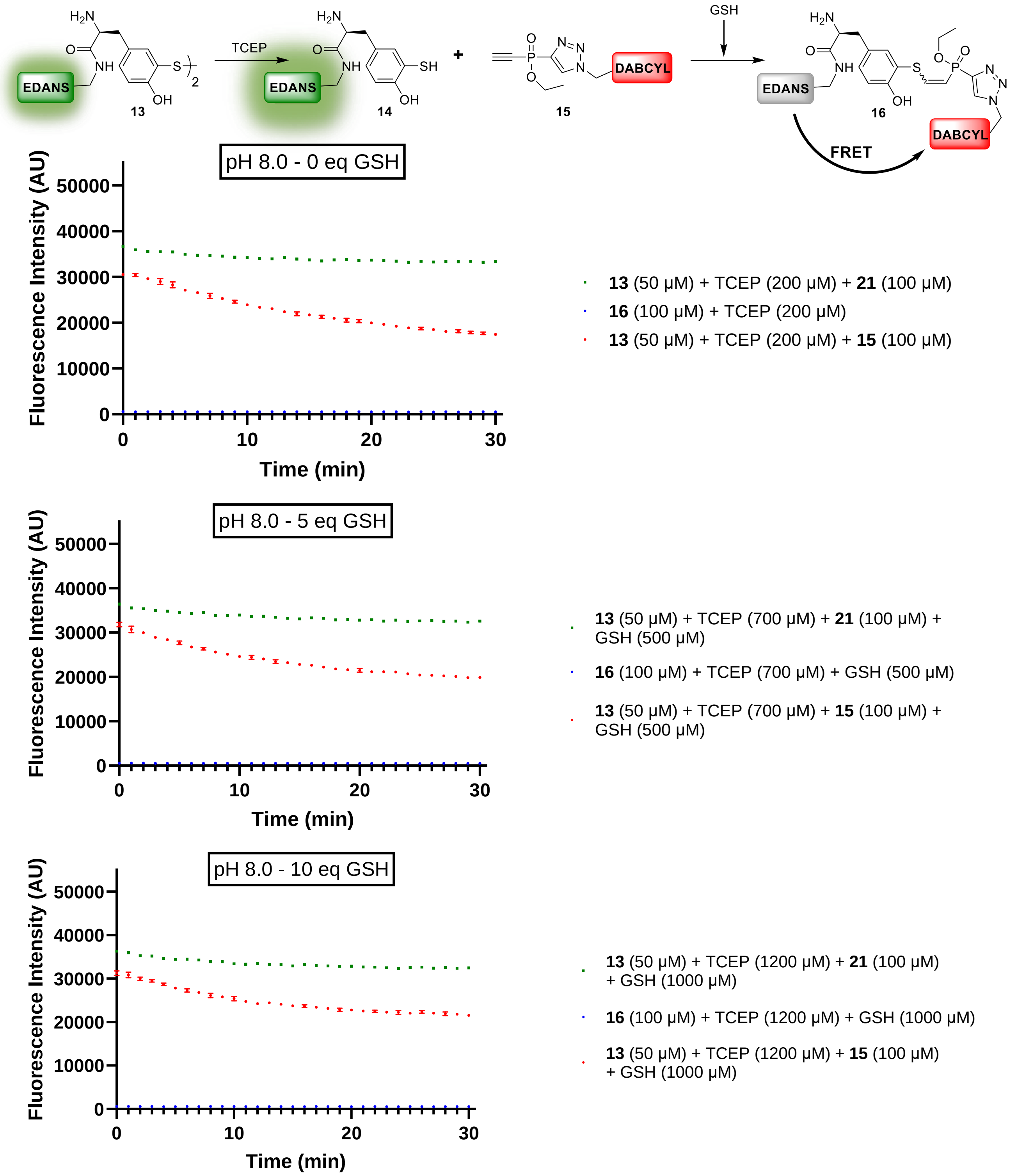

Figure S18. Raw fluorescence data (λex = 340 nm, λem = 495 nm) obtained for the reaction between EDANS aryl thiol **14** and DABCYL-ETP **15** at pH 8.0 (0.2 M phosphate buffer, 10% MeCN, 5% DMSO) with gluthathione (GSH) competition. Displayed are the reaction (**13** + **15** + TCEP) and the controls for 100% (**13** + **21** + TCEP) and 0% (**16** + TCEP) fluorescence with 0 / 5 / 10 equivalents of GSH competition. These datasets were recorded together in a single experiment (N = 1, n = 2).

Figure S19

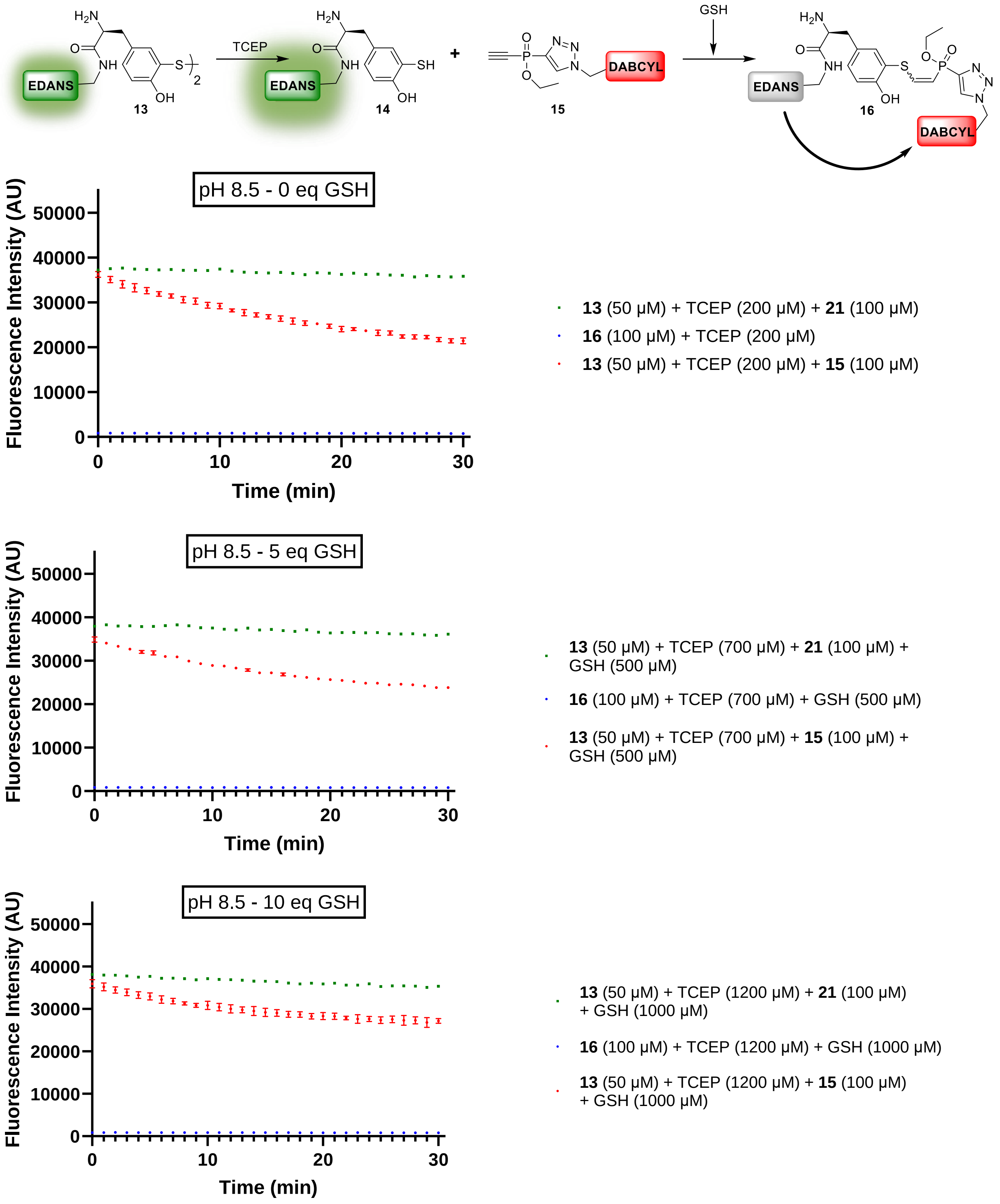

Figure S19. Raw fluorescence data (λex = 340 nm, λem = 495 nm) obtained for the reaction between EDANS aryl thiol **14** and DABCYL-ETP **15** at pH 8.5 (0.2 M tris buffer, 10% MeCN, 5% DMSO) with gluthathione (GSH) competition. Displayed are the reaction (**13** + **15** + TCEP) and the controls for 100% (**13** + **21** + TCEP) and 0% (**16** + TCEP) fluorescence with 0 / 5 / 10 equivalents of GSH competition. These datasets were recorded together in a single experiment (N = 1, n = 2).

Figure S20

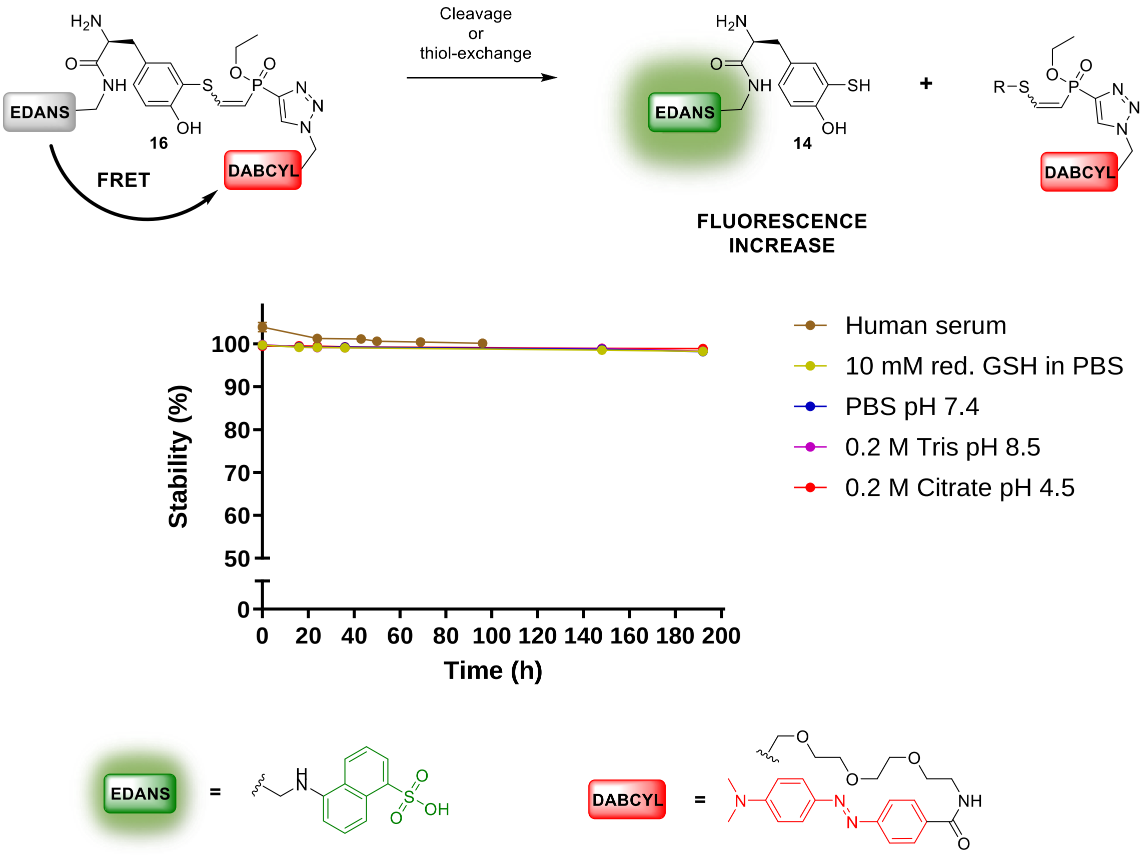

Figure S20. Fluorescent plate reader assay to monitor the stability of aryl thiol-ETP adduct **16**. Compound **16** quenches the fluorescence emitted (λex = 340 nm, λem = 495 nm) by virtue of FRET. Cleavage of thiol exchange of **16** would liberate the EDANS fluorophore (**14**), causing an increase in fluorescence. The stability of **16** (10 μM) was determined in human serum, 10 mM reduced GSH in PBS pH 7.4, PBS pH 7.4, 0.2 M Tris buffer pH 8.5 and 0.2 M citrate buffer pH 4.5. Mean values and standard deviation (SD) are shown (N = 1, n ≥ 3). Datasets were recorded in two separate experiments.

Figure S21

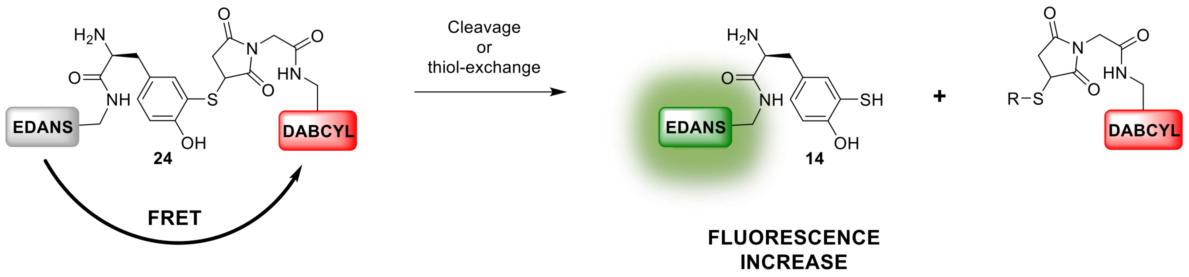

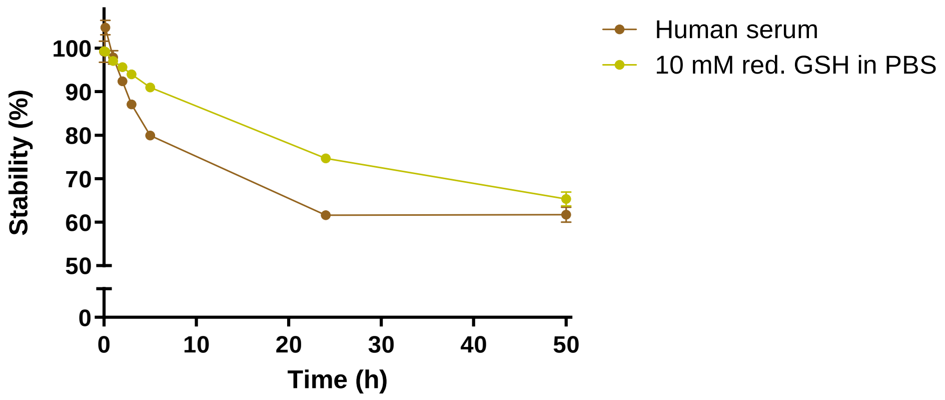

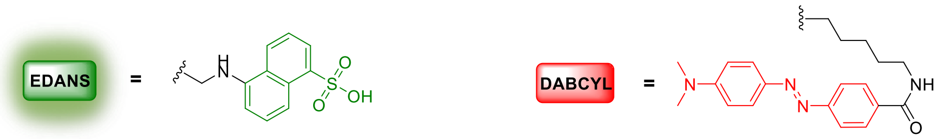

Figure S21. Fluorescent plate reader assay to monitor the stability of aryl thiol-maleimide adduct **24**. Compound **24** quenches the fluorescence emitted (λex = 340 nm, λem = 495 nm) by virtue of FRET. Cleavage of thiol exchange of **24** would liberate the EDANS fluorophore (**14**), causing an increase in fluorescence. The stability of **24** (10 µM) was determined in human serum and 10 mM reduced GSH in PBS pH 7.4. Mean values and standard deviation (SD) are shown (N = 1, n ≥ 3).

Figure S22

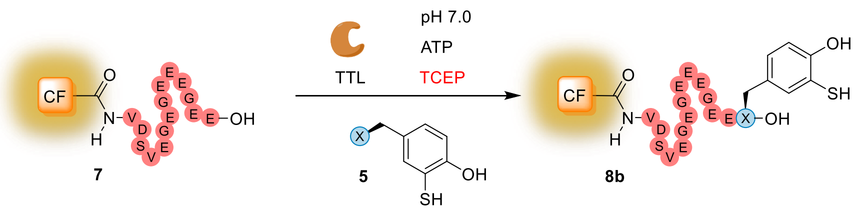

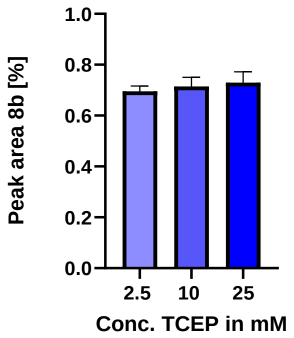

Figure S22. Tub-tag® labeling of 5(6)-CF-Tub-tag® (**7**) to form **8b** using **5** (from disulfide **6**) with variable concentrations of TCEP. Tyrosination reactions were performed in a 250 µL solution consisting of 200 µM 5(6)-CF-Tub-tag®(**7**), 1 µM SUMO-TTL (0.5 mol%), 1.25 mM **6**, 25 / 10 / 2.5 mM TCEP, 5 mM ATP, 20 mM 3-(*N*-morpholino)propanesulfonic acid (MOPS) pH 7.0, 100 mM KCl, 10 mM $MgCl_2$, 10% (v/v) propane-1,2-diol, 37°C, 850 rpm shaking. Crude reaction mixture samples (125 µL) were quenched with 1% TFA (125 µL) after 5 hours of reaction time and analyzed using LC-MS (C18, linear gradient 5 → 95% MeCN in H_2O, 0.1% TFA, 15 min, 5 µL injection). Relative quantities of substrate and product peptide were determined from the corresponding peak areas in the UV spectra (λ = 220 nm). The mean values and standard deviation (SD) of two replicate reactions are shown (N = 2). Quantification of the dataset shown here is presented in SI section 7.2.

Figure S23

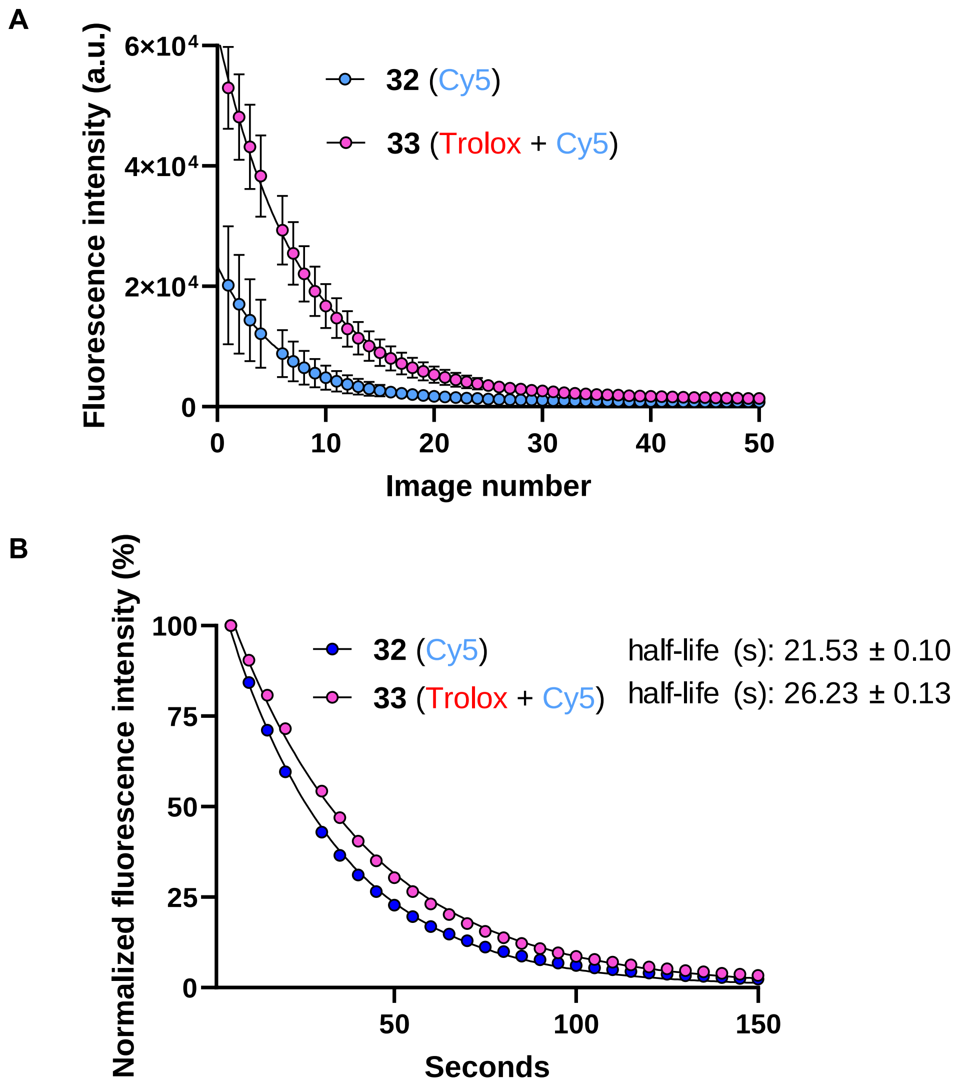

Figure S23. (A) Quantification of fluorescence intensity upon photobleaching conjugates **32** and **33** (λex = 640 nm, λem = 685/50 nm) bound to HeLa-H2B-EGFP cells (**Figure 4C**). Images were obtained at 5 second intervals by exposure to 100% laser power with 100 ms exposure time, thereby inducing photobleaching. Each point in the graph shows the mean fluorescence intensity of 45 nuclei ± standard deviation (SD) for the indicated image number. (B) Normalization of the fluorescence intensity to the maximum value obtained for each conjugate. The x-axis was converted from image number to seconds, which enabled determination of the half-life during photobleaching (t1/2) using one phase decay (calculation parameters presented in SI section 7.7).

Figure S24

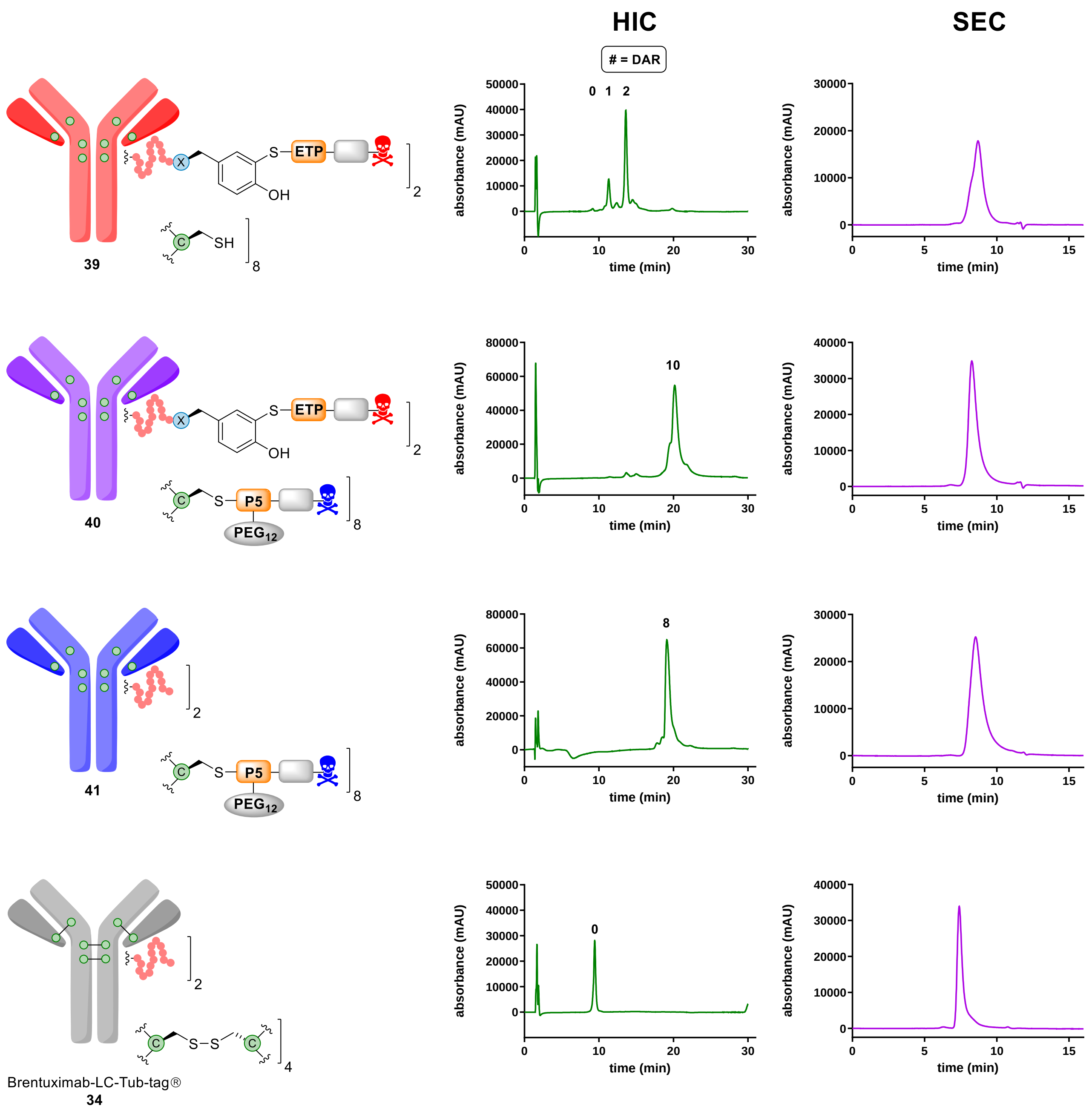

Figure S24. Analytical hydrophobic interaction chromatography (HIC) and size exclusion chromatography (SEC) for ADCs **39** - **41** after purification by fast protein liquid chromatography (FPLC), using Brentuximab-LC-Tub-tag® (**34**) as reference. For HIC analyses, peaks were annotated with their respective drug-to-antibody ratio (DAR).

Figure S25

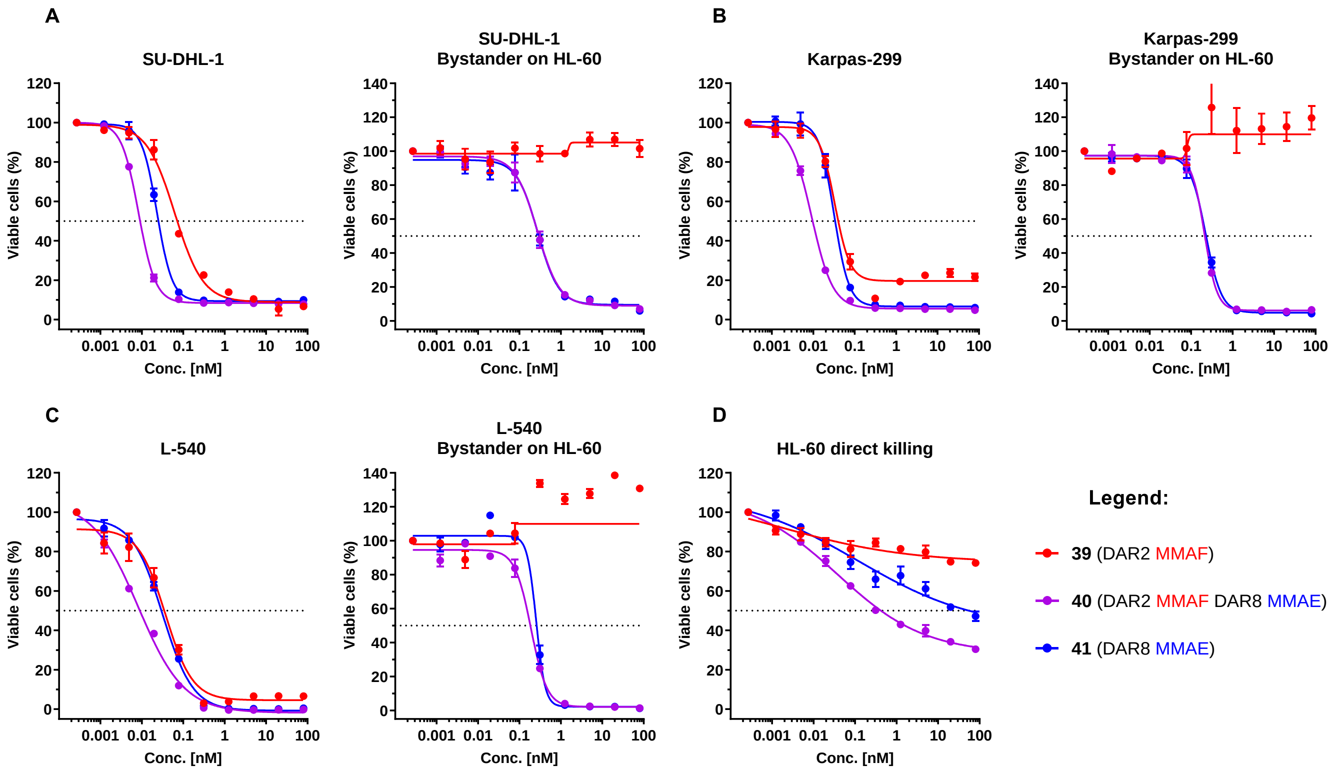

E

Cell line (CD30 level)	SR-786 (+++)		L-540 (+++)		Karpas-299 (++)		SU-DHL-1 (+)		L-428 (+)		HL60 (-)
EC50 (pM)	Direct	HL60	Direct	HL60	Direct	HL60	Direct	HL60	Direct	HL60	Direct
**39** (DAR2)	26.8	>80000	37.3	>80000	33.1	>80000	62.5	>80000	23.2	>80000	>80000
**40** (DAR 10)	4.80	234	8.22	190	9.11	209	8.33	271	10.5	179	(38.9)
**41** (DAR 8)	13.3	250	31.8	254	31.7	225	23.2	276	(35.1)	162	(116)

Figure S25. *In vitro* evaluation of ADCs **39** - **41** towards CD30+ mediated cytotoxicity (96 hours incubation) and supernatant-based bystander cytotoxicity (on HL-60 cells; 96 hours incubation). (A – C) Direct and bystander (HL-60) cytotoxicity for cell lines SU-DHL-1, Karpas-299 and L-540. Graphs display the concentration of ADC (0 - 80 nM; four-fold dilution steps) on the x-axis and the % of viable cells on the y-axis (where 100% represents the medium control). Mean values and standard error of the mean (SEM) for one experiment is shown (N = 1, n = 2). (D) Direct cytotoxicity for cell line HL-60, serving as a control for the bystander cytotoxicity experiments. (E) Summary of the data presented in **Figure 6** and **Figure S25** (A – D), indicating CD30 expression per cell line (+++, ++, + or -) and displaying EC50 values in pM. EC50 values were displayed for graphs where the % of viable cells dropped below 50% and marked with brackets for graphs where the % of viable cells remained above 25%. When these criterea were not met, the EC50 value was denoted as '>80000 pM'.

2. Supplementary Schemes

Scheme. S1

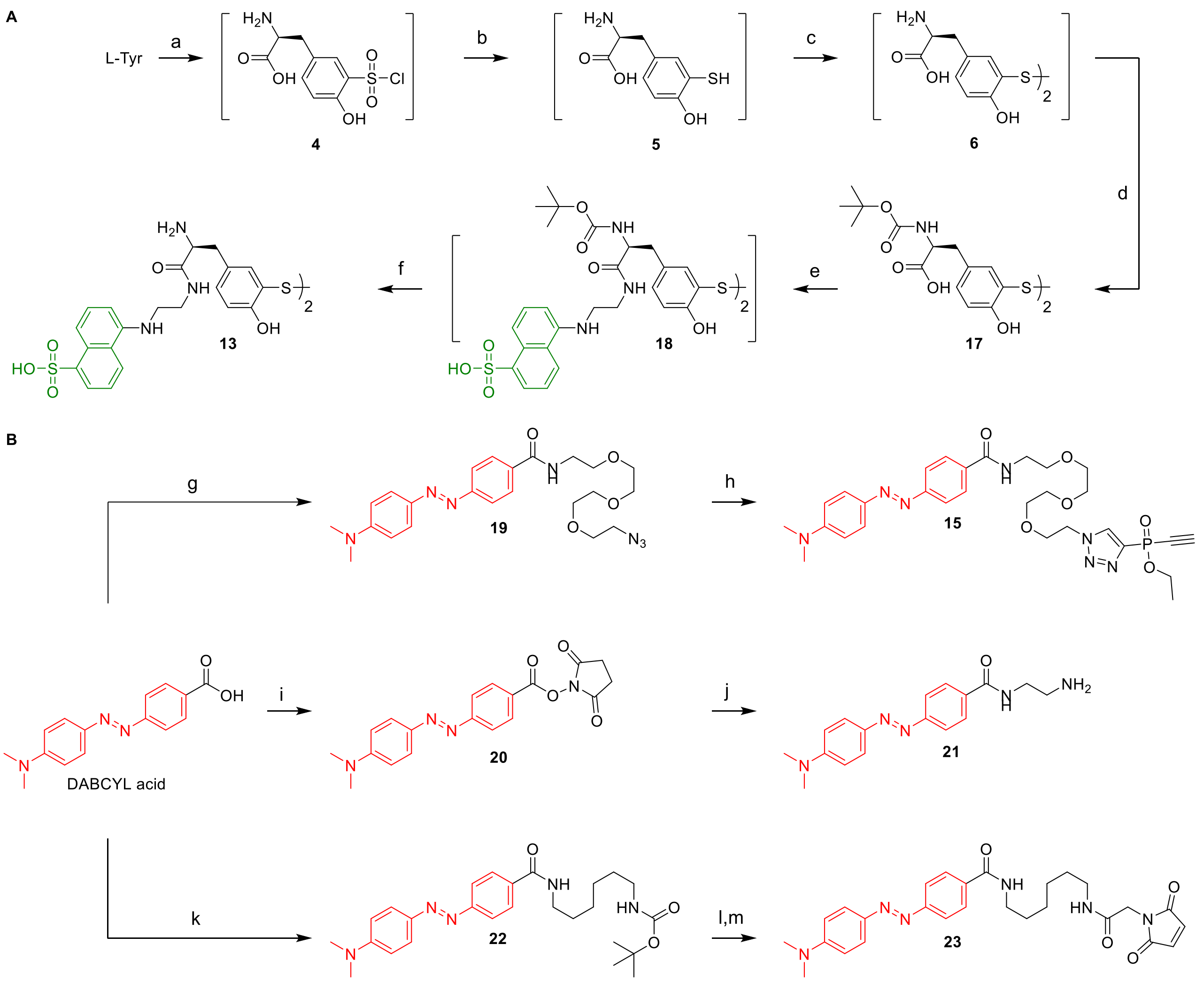

Scheme S1: (A) Synthesis of EDANS diaryl disulfide **13**. (B) Synthesis of DABCYL-ETP **15**, DABCYL-NH2 **21**, and DABCYL-Maleimide **23**. Reagents/conditions: (a) chlorosulfonic acid (neat), −30°C to rt; (b) PPh_3 (4 eq), dioxane/H_2O (1:1), 90°C; (c) workup, concentrate, O_2, 70°C; (d) NaOH, Boc_2O, dioxane/H_2O (1:1), rt, 27% over four steps; (e) EDANS, HCTU, DIPEA, DMF, rt; (f) TFA, rt, 59% over two steps; (g) HATU, DIPEA, DMF, rt, 73%; (h) ethyl diethynyl phosphinate[1,2], Cu(I)Br, THPTA, MeCN, 0.2 M phosphate buffer pH 6.0, rt, 59%; (i) *N*-hydroxysuccinimide, DMF, EDC, rt; (j) ethylenediamine, DIPEA, 68% over two steps; (k) Boc-hexanediamine, HATU, DIPEA, DMF, rt; (l) TFA/DCM (1:1), rt; (m) maleimidoacetic acid *N*-hydroxysuccinimide ester, DIPEA, DMF, rt, 12% over three steps.

Scheme. S2

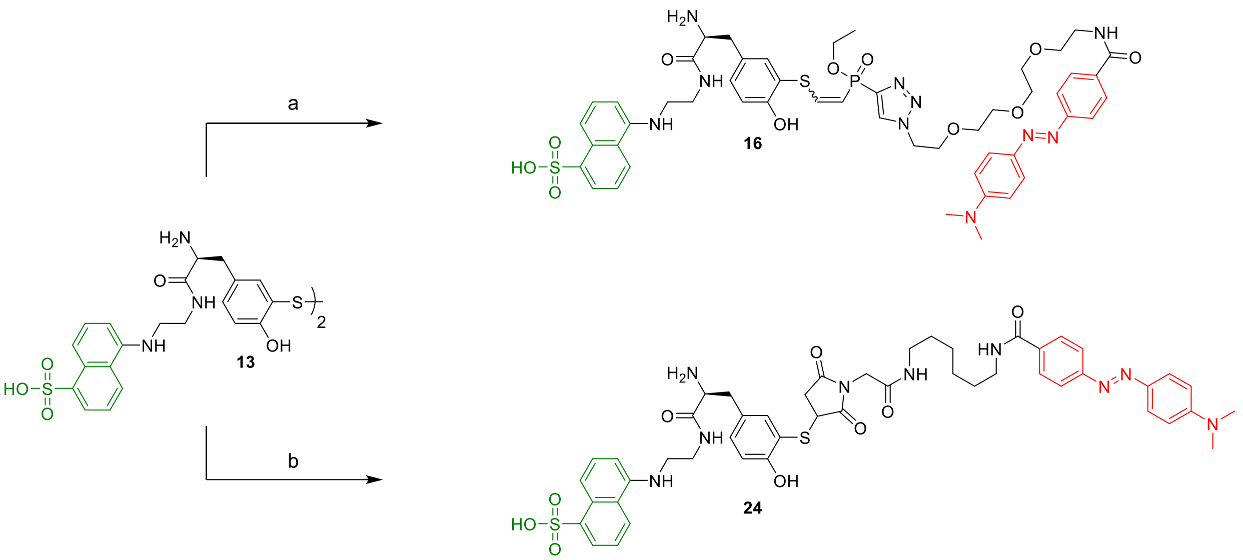

Scheme S2: Synthesis of EDANS-aryl thiol DABCYL-ETP adduct **16** and EDANS-aryl thiol DABCYL-maleimide adduct **24**. Reagents/conditions: (a) i. TCEP, MeCN, DMSO, 0.2 M phosphate buffer pH 6.0, rt; ii. **15**, rt, 52%; (b) i. TCEP, MeCN, DMSO, PBS pH 7.4, rt; ii. **23**, rt, 40%.

Scheme. S3

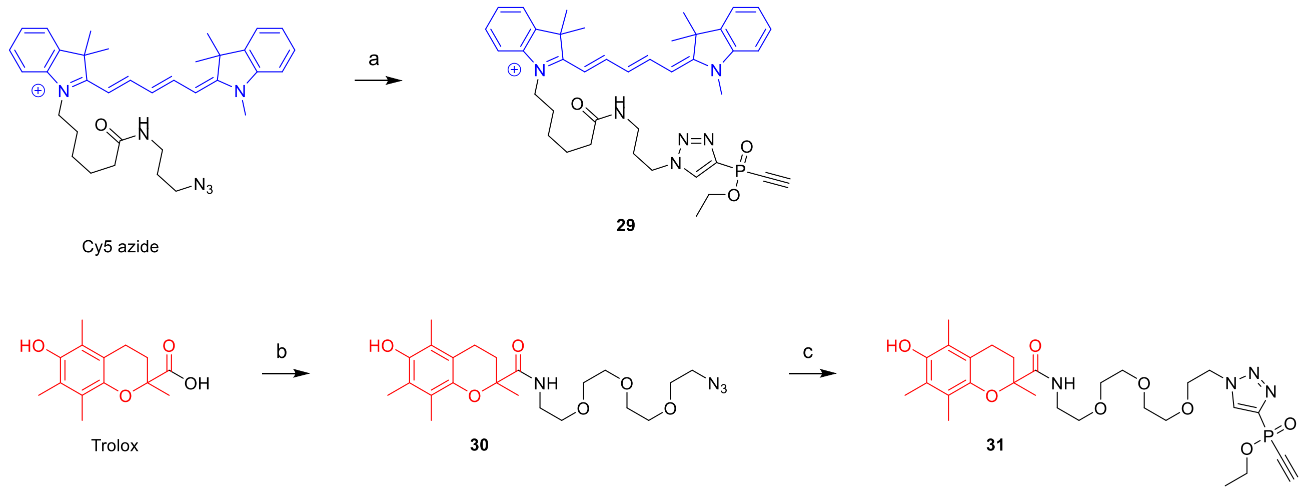

Scheme S3: Synthesis of ETP-Cy5 (**29**) and ETP-Trolox (**31**). Reagents/conditions: (a) ethyl diethynyl phosphinate[1,2], Cu(I)Br, THPTA, MeCN, 0.2 M phosphate buffer pH 6.0, rt, 72%; (b) 11-azido-3,6,9-trioxaundecan-1-amine, PyBOP, DIPEA, DMSO, rt, 87%; (c) ethyl diethynyl phosphinate[1,2], Cu(I)Br, THPTA, MeCN, 0.2 M phosphate buffer pH 6.0, rt, 41%.

Scheme. S4

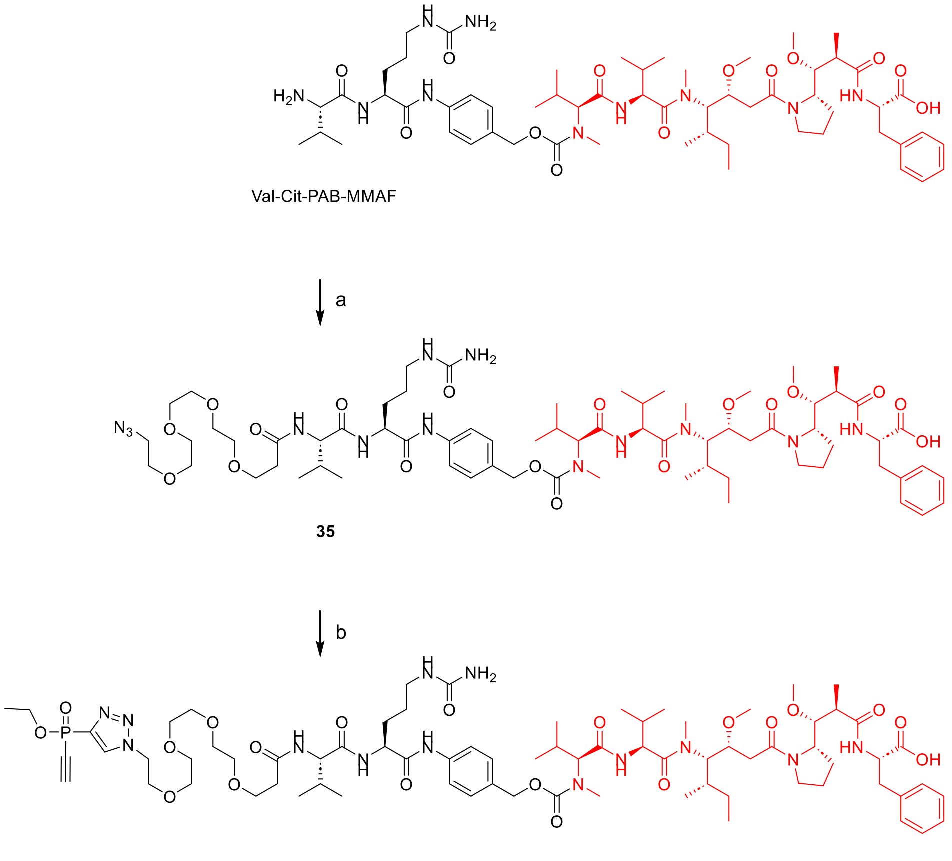

36

Scheme S4: Synthesis of ETP-PEG$_3$-Val-Cit-PAB-MMAF **36**. Reagents/conditions: (a) *N*-Succinimidyl 15-azido-4,7,10,13-tetraoxapentadecanoate, DIPEA, DMSO, rt, 60%; (b) ethyl diethynyl phosphinate[1,2], Cu(I)Br, THPTA, DMSO, MeCN, 0.2 M phosphate buffer pH 6.0, rt, 63%.

3. Supplementary Tables

Table S1

Table S1: Tub-tag® labeling of GBP1-C-Tub-tag® (**26**) with aryl disulfide **6**, TCEP, ATP and SUMO-TTL to afford GBP1-C-Tub-tag®-STyr (**27**).

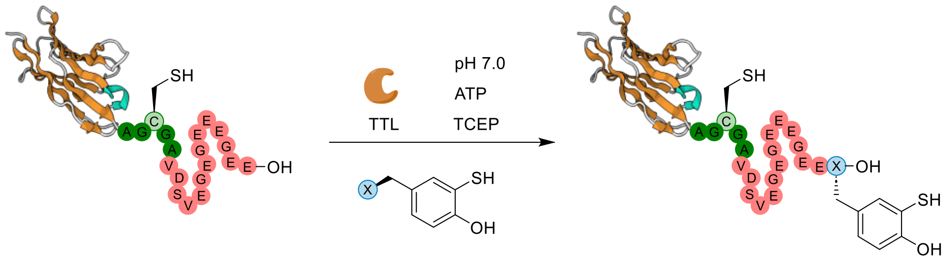

	Reaction conditions[a]				MS Analysis Nanobody[b]			
Entry	[**26**] (μM)	[SUMO-TTL] (μM (mol%))	[TCEP] (mM)	[**6**] (mM)	Nb-SH **27** (%)	Nb-SO_2H (%)	Nb-SO_3H (%)	Nb **26** (%)
N = 10	100	6 (6)	2.5	1.25	73.5 – 89.7	8.2 – 20.3	0.4 – 6.2	0.0 – 0.1

[a]Tyrosination reactions were performed in a 130 μL solution using the following conditions: 100 μM GBP1-C-Tub-tag® (**26**), 6 μM SUMO-TTL (6.0 mol%), 1.25 mM aryl disulfide **6**, 2.5 mM TCEP, 5 mM ATP in **TTL buffer** (20 mM 3-(*N*-morpholino)propanesulfonic acid (MOPS) pH 7.0, 100 mM KCl, 10 mM $MgCl_2$, 10% (v/v) propane-1,2-diol) at 37°C, 850 rpm shaking for 4 hours. [b]A crude reaction mixture sample (1 μL) was drawn after 4 hours and diluted with PBS (100 μL). The sample was analyzed using intact protein MS (10 μL injection). The relative signal intensities of major products are reported (%) for a sample set of 10 experiments (N = 10; displaying lower and upper limits for each species).

Table S2

Table S2: Selective aryl thiol modification of GBP1-C-Tub-tag®-STyr (**27**) with ETP-Biotin **9** to obtain GBP1-C-Tub-tag®-STyr(Biotin) (**28**). Entry 4 was displayed in **Figure 4A**. Intact protein MS spectra (raw and deconvoluted) are presented in SI section 7.5.

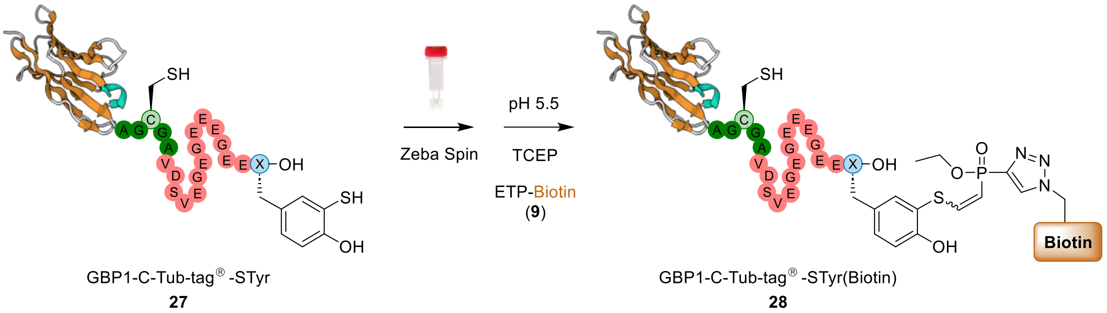

	Reaction conditions[a]				MS Analysis Nanobody[b]			
**Entry**	Zeba Spin	pH	[TCEP] (µM)	[**9**] (µM)	Nb-S-Biotin **28** (%)	Nb, Nb-SH, Nb-S_{ox} (%)	Nb-S-Biotin **28** + **9** (%)	Nb-S-Biotin **28** + **9** + TCEP (%)
1	x1	6.0	1000	200	82.8	0.5	9.0	7.8
2	x1	6.0	500	150	93.2	1.2	3.9	1.8
3	x1	5.5	500	150	93.6	0.6	3.4	2.4
4	x1	5.5	300	120	96.3	1.2	1.9	0.5

[a]The Tub-tag® reaction mixture (to form **27**; 130 µL) was rebuffered to 0.2 M phosphate/citrate buffer using Zeba® Spin desalting columns. The rebuffered solution was treated with TCEP, homogenized and incubated at 37°C, 850 rpm shaking for 30 minutes. Afterwards, the reaction mixture was briefly allowed to cool down to room temperature before adding ETP-Biotin **9**. The reaction mixture was carefully homogenized and incubated at 25°C, 850 rpm shaking for 16-20 hours. [b]A crude reaction mixture sample (1 µL) was drawn after 16-20 hours and diluted with PBS (100 µL). The sample was analyzed using intact protein MS (10 µL injection). The relative signal intensities of major products are reported (%).

Table S3

Table S3: Selective aryl thiol modification of GBP1-C-Tub-tag®-STyr (**27**) with ETP-Cy5 **29** to obtain GBP1-C-Tub-tag®-STyr(Cy5) (**32**). Entry 3 was displayed in **Figure 4A**. Intact protein MS spectra (raw and deconvoluted) are presented in SI section 7.5.

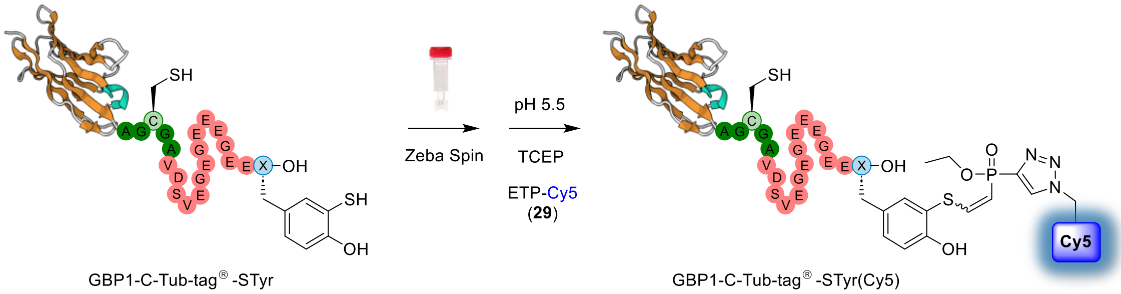

	Reaction conditions[a]				MS Analysis Nanobody[b]			
**Entry**	Zeba Spin	pH	[TCEP] (µM)	[**29**] (µM)	Nb-S-Cy5 **32** (%)	Nb, Nb-SH, $Nb\text{-}S_{ox}$ (%)	Nb-S-Cy5 **32** + **29** (%)	Nb-S-Cy5 **32** + **29** + TCEP (%)
1	x1	5.5	300	100	69.0	30.2	0.6	0.1
Cont.*	-	-	100	65	89.7	4.8	5.4	0.2
2	x1	5.5	300	130	89.5	8.7	1.6	0.2
3	x1	5.5	300	145	93.6	4.8	1.4	0.2

[a]The Tub-tag® reaction mixture (to form **27**; 130 µL) was rebuffered to 0.2 M citrate buffer pH 5.5 using Zeba® Spin desalting columns. The rebuffered solution was treated with TCEP, homogenized and incubated at 37°C, 850 rpm shaking for 30 minutes. Afterwards, the reaction mixture was briefly allowed to cool down to room temperature before adding ETP-Cy5 **29**. The reaction mixture was carefully homogenized and incubated at 25°C, 850 rpm shaking for 16-20 hours. [b]A crude reaction mixture sample (1 µL) was drawn after 16-20 hours and diluted with PBS (100 µL). The sample was analyzed using intact protein MS (10 µL injection). The relative signal intensities of major products are reported (%). *For entry 1, the sample was treated once more with TCEP / ETP-Cy5 **29** after the initial reaction procedure.

Table S4

Table S4: Cysteine modification of GBP1-C-Tub-tag®-STyr(Cy5) (**32**) with ETP-Trolox **31** to obtain nanobody double conjugate GBP1-C(Trolox)-Tub-tag®-STyr(Cy5) (**33**). Intact protein MS spectra (raw and deconvoluted) are presented in SI section 7.5.

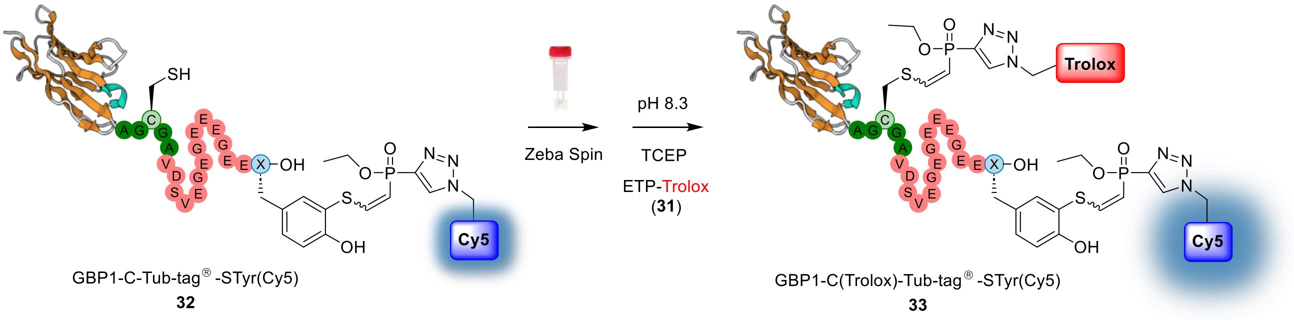

	Reaction conditions[a]				MS Analysis Nanobody[b]			
**Entry**	Zeba Spin	pH	[TCEP] (µM)	[**31**] (µM)	Nb-S-Cy5-Trolox **33** (%)	Nb-S-Cy5-Trolox **33** + TCEP (%)	Nb, Nb-SH, Nb-S_{ox}, Nb **32**, (%)	Nb **32** + **29**, Nb **32** + **29** + TCEP (%)
1	x1	8.3	300	150	63.8	29.5	0.9	5.8
2	x1	8.3	125	150	78.5	15.7	2.2	3.6

[a]The reaction mixture to form **32** (130 µL) was rebuffered to 50 mM Tris buffer (1 mM EDTA, 100 mM NaCl, pH 8.3) using Zeba® Spin desalting columns. The rebuffered solution was treated with TCEP, homogenized and incubated at 37°C, 850 rpm shaking for 30 minutes. Afterwards, the reaction mixture was briefly allowed to cool down to room temperature before adding ETP-Trolox **31**. The reaction mixture was carefully homogenized and incubated at 25°C, 850 rpm shaking for 16-20 hours. [b]A crude reaction mixture sample (1 µL) was drawn after 16-20 hours and diluted with PBS (100 µL). The sample was analyzed using intact protein MS (10 µL injection). The relative signal intensities of major products are reported (%).

Table S5

Table S5: Tub-tag® labeling of Brentuximab-LC-Tub-tag® (**34**) with aryl disulfide **6**, TCEP, ATP and SUMO-TTL to afford Brentuximab-LC-Tub-tag®-STyr (**38**). Intact protein MS spectra (raw and deconvoluted) are presented in section 7.8.

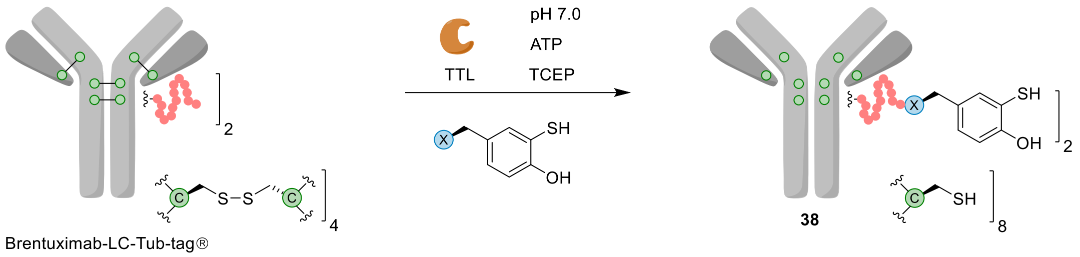

Brentuximab-LC-Tub-tag®
34

	Reaction conditions[a]				MS Analysis Light Chain (LC)[b]			
**Entry**	[**34**] (µM)	[SUMO-TTL] (µM (mol%))	[TCEP] (mM)	[**6**] (mM)	LC-SH (%)	LC-SO_2H (%)	LC-SO_3H (%)	LC (%)
1	40	5 (12.5)	2.5	1.25	72.7	6.7	4.1	16.5
2 (N ≥ 10)	100	12.5 (31.3)	2.5	1.25	79.2 – 89.9	7.3 – 15.8	2.8 – 4.9	0.0

[a]Tyrosination reactions were performed in a 130 µL solution using the following conditions: 40 µM Brentuximab-LC-Tub-tag® (**34**), 12.5 µM SUMO-TTL (31.3 mol%), 1.25 mM aryl disulfide **6**, 2.5 mM TCEP, 5 mM ATP in **TTL buffer** (20 mM 3-(*N*-morpholino)propanesulfonic acid (MOPS) pH 7.0, 100 mM KCl, 10 mM $MgCl_2$, 10% (v/v) propane-1,2-diol) at 37°C, 850 rpm shaking for 4 hours. [b]A crude reaction mixture sample (1 µL) was drawn after 4 hours and diluted with PBS (180 µL) and TCEP (50 mM in PBS, 20 µL) in an Eppendorf tube. The sample was incubated at 37°C, 850 rpm shaking for ≥ 15 – 30 minutes and subsequently transferred to an MS vial for analysis using intact protein MS (3 µL injection). The relative signal intensities of major products are reported (%) for a sample set of over 10 experiments (N ≥ 10; displaying lower and upper limits for each species).

Table S6

Table S6: Top: selective aryl thiol modification of Brentuximab-LC-Tub-tag®-STyr (**38**) with ETP-PEG$_3$-Val-Cit-PAB-MMAF (**36**) to obtain Brentuximab DAR 2 MMAF conjugate **39**. Entry 9 was displayed in **Figure 5A**. Intact protein MS spectra (raw and deconvoluted) are presented in SI section 7.8. Bottom: analytical hydrophobic interaction chromatography (HIC) for selected entries.

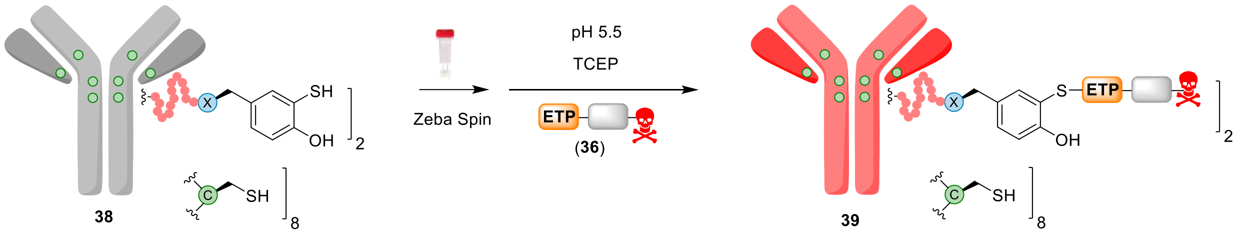

	Reaction conditions[a]				MS Analysis Light Chain (LC)[b]			MS Analysis Heavy Chain (HC)[c]
Entry	Zeba Spin	pH	[TCEP] (µM)	[36] (µM)	LC-SH + 36 (%)	LC, LC-SH, LC-S$_{ox}$ (%)	LC-SH + 36 + 36 (%)	HC (%)
1	x1	6.0	1000	160	55.5	44.6	0.0	98.9
2	x1	6.0	1000	160	54.6	44.9	0.0	98.5
Cont.	-	-	1000	80	80.1	19.8	0.0	97.7
3	x1	6.0	1000	240	86.7	13.3	0.0	97.8
4	x2	6.0	1000	160	87.6	12.5	0.0	97.9
5	x2	6.0	1000	240	92.6	7.4	0.0	96.4
6	x2	6.0	750	240	90.4	5.5	4.1	89.1
7	x2	5.5	1000	240	96.5	2.6	1.0	94.2
8	x2	5.5	750	160	87.3	12.7	0.0	99.1
9	x2	5.5	750	240	97.1	1.9	1.1	96.8

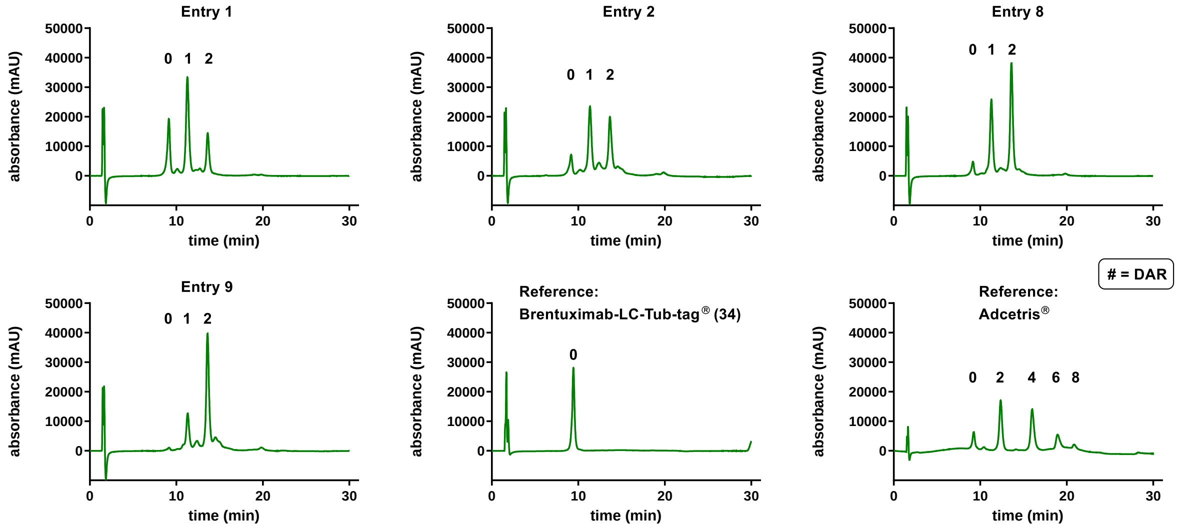

[a]The Tub-tag® reaction mixture (to form **38**; 130 µL) was rebuffered to 0.2 M phosphate/citrate buffer using Zeba® Spin desalting columns. The rebuffered solution was treated with TCEP, homogenized and incubated at 37°C, 850 rpm shaking for 30 minutes. Afterwards, the reaction mixture was briefly allowed to cool down to room temperature before adding ETP-PEG$_3$-Val-Cit-PAB-MMAF (**36**). The reaction mixture was carefully homogenized and incubated at 25°C, 850 rpm shaking for 16-20 hours. [b]A crude reaction mixture sample (1 µL) was drawn after 16-20 hours and diluted with PBS (180 µL) and TCEP (50 mM in PBS, 20 µL) in an Eppendorf tube. The sample was incubated at 37°C, 850 rpm shaking for ≥ 15 – 30 minutes and subsequently transferred to an MS vial for analysis using intact protein MS (3 µL injection). The relative signal intensities of major products are reported (%).

4. General Information

4.1 Chemicals and Solvents

Commercially available reagents and solvents were used as received without further purification. Dry solvents were purchased from Acros Organics (Thermo Fisher scientific, USA). Aminoacids and resins for SPPS were purchased from Novabiochem (Merck, USA) or Iris Biotech GmbH (Germany). Moisture and oxygen sensitive reactions were performed under N_2/Ar atmosphere (balloon or Schlenk line). DIPEA was stored over KOH pellets.

4.2 Flash- and thin layer chromatography

Flash column chromatography was performed using NORMASIL 60® silica gel 40-63 μm (VWR international, USA). Glass TLC plates, silica gel 60 W coated with fluorescent indicator F254s, were purchased from Merck (Merck Group, Germany). Spots were visualized by fluorescence depletion with a 254 nm lamp or manganese staining (10 g K_2CO_3, 1.5 g $KMnO_4$, 0.1 g NaOH in 200 ml H_2O), followed by heating.

4.3 Semi-preparative HPLC

Semi-preparative HPLC was performed on a Gilson PLC 2050 system, using a gradient of MeCN in H_2O (0.1% TFA) as mobile phase with one of the following stationary phases (Macherey-Nagel GmbH & Co. Kg, Germany):

- VP250/10 Nucleodur C_{18} HTec, 5 μM
- VP250/21 Nucleodur 100-5 C_{18} ec, 5 μM
- VP250/32 Nucleodur C_{18} HTec, 5 μM

4.4 NMR-Spectroscopy

NMR spectra (^{1}H, ^{13}C, ^{31}P, ^{1}H COSY, HSQC and HMBC) were recorded with a Bruker Avance III 600 MHz spectrometer (Bruker Corp., USA) at ambient temperature unless stated otherwise. Chemical shifts are reported as δ values (ppm) and were referenced to tetramethylsilane (δ = 0.00 ppm) or the residual solvent peak as internal standard ($CDCl_3$: 7.26 [ppm]; DMSO-d_6: 2.50 [ppm]; 4.79 D_2O [ppm] for ^{1}H-spectra and $CDCl_3$: 77.16 [ppm]; DMSO-d_6: 39.52 [ppm] for ^{13}C-spectra). *J* couplings are reported in Hz. Signal multiplicities are abbreviated as follows: s: singlet; d: doublet; t: triplet; q: quartet; p: pentet (quintet); m: multiplet; app.: apparent.

Whenever product mixtures are described (**3**$_{ab}$, **10-Z**$_{ab}$, **10-E**$_{ab}$), the number of H or C atoms reported matches the amount of H or C atoms that corresponds to an equal (1:1) product ratio. The actual product ratio is still reported in the text and displayed in the relevant spectra.

4.5 UPLC-UV/MS

UPLC-UV/MS traces were recorded on a Waters H-class instrument equipped with a quaternary solvent manager, a Waters autosampler, a Waters TUV detector and a Waters Acquity QDa detector with an Acquity UPLC BEH C18 1.7 µm, 2.1 x 50 mm RP column with a flow rate of 0.6 mL/min (Waters Corp., USA). The following gradients were used:

linear gradient 5 → 95% MeCN, 0.1% TFA, 15 min: 0.1% TFA in H_2O; B: 0.1% TFA in MeCN.
5% B 0 - 1.5 min → 5-95% B 1.5-13 min → 95% B 13-13.9 min → 5% B 13.9-15 min;

linear gradient 5 → 95% MeCN, 0.1% TFA, 5 min: 0.1% TFA in H_2O; B: 0.1% TFA in MeCN.
5% B 0 - 0.5 min → 5-95% B 0.5-3 min → 95% B 3-3.9 min → 5% B 3.9-5 min.

4.6 HR-MS

High resolution ESI-MS spectra were recorded on a Waters H-class instrument equipped with a quaternary solvent manager, a Waters sample manager-FTN, a Waters PDA detector and a Waters column manager with an Acquity UPLC protein BEH C18 column (1.7 µm, 2.1 mm x 50 mm). Samples were eluted with a flow rate of 0.3 mL/min. The following gradient was used: **"QTof":** 0.01% FA in H_2O; B: 0.01% FA in MeCN. 5% B: 0-1 min; 5 to 95% B: 1-7min; 95% B: 7 to 8.5 min. Mass analysis was conducted with a Waters XEVO G2-XS QTof analyzer.

4.7 Intact protein MS

Intact proteins were analyzed using a Waters H-class instrument equipped with a quaternary solvent manager, a Waters sample manager-FTN, a Waters PDA detector and a Waters column manager with an Acquity UPLC protein BEH C4 column (300 Å, 1.7 µm, 2.1 mm x 50 mm). Proteins were eluted with a flow rate of 0.3 mL/min with 80°C column temperature. The following gradient was used: A: 0.01% FA in H_2O; B: 0.01% FA in MeCN. 5-95% B 0-6 min. Mass analysis was conducted with a Waters XEVO G2-XS QTof analyzer. Raw data was deconvoluted with MaxEnt 1.

4.8 Protein concentration determination

Protein concentrations were determined by absorption spectroscopy measurements at 280 nm using the extinction coefficient and molecular weight of the protein on a NanoDrop ND-1000. In addition or as alternative concentrations were determined by BCA assay (Thermo Fisher Scientific, USA) according to the manufacturer's protocol.

4.9 Analytical hydrophilic interaction chromatography (HIC)

Analytical hydrophobic interaction chromatography (HIC) was conducted using an analytical HPLC (Shimadzu LC-20A Prominence System including a diode array detector). Antibody/ADC samples (0.3 mg/mL; 50 µL injection) were loaded onto a HIC column (TSKgel Butyl-NPR 4.6 mm I.D. x 10 cm, 2.5 µm from TOSOH) and analyzed during a 30-minute gradient with a flow rate of 0.5 mL/min using a phosphate buffer at pH 7 as the mobile phase, consisting of Eluent A (25 mM Na_2HPO_4 / NaH_2PO_4 +1,5M ($(NH_4)_2SO_4$) and Eluent B (25 mM Na_2HPO_4 / NaH_2PO_4 + 20% Isopropanol).

0 – 20 min	0 – 100% B
20 – 25 min	100% B
25 – 30 min	100 – 0% B

UV chromatograms were recorded at 280 nm. Data was plotted using GraphPad Prism 10.3.1. Quantification of different DAR species was achieved after integration of the peak areas at 280 nm in the Shimadzu software.

4.10 Analytical hydrophilic interaction chromatography (SEC)

Analytical size-exclusion chromatography (SEC) was conducted using an analytical HPLC (Shimadzu LC-20A Prominence System including a diode array detector). Antibody/ADC samples (0.3 mg/mL; 50 µL injection) were loaded onto a SEC column (TSKgel G3000SW_{XL} 7.8 mm I.D. x 30 cm, 5.0 µm from TOSOH) and analyzed during a 16-minute isocratic gradient with a flow rate of 1.0 mL/min using a phosphate buffer at pH 6.8 (100 mM Na_2HPO_4 / KH_2PO_4, 100 mM Na_2SO_4) as the mobile phase. UV chromatograms were recorded at 280 nm. Data was plotted using GraphPad Prism 10.3.1.

4.11 LC-MS/MS

LC-MS analysis was performed using an UltiMate 3000 RSLC nano LC system coupled on-line to an Orbitrap Fusion mass spectrometer (Thermo Fisher Scientific). For sample loading a PepMap C-18 trap-column (Thermo Fischer Scientific) of 0.075 mm ID x 50 mm length, 3 µm particle size and 100 Å pore size was used. The loading mobile phase A contained 1% acetonitrile and 0.05% TFA acid in water, and mobile phase B 0.05% TFA acid in acetonitrile. Reversed-phase separation was performed using a 50 cm analytical column (in-house packed with Poroshell 120 EC-C18, 2.7µm, Agilent Technologies) with mobile phase A contained 0.1% formic acid in water, and mobile phase B 0.1% formic acid in acetonitrile using a 45 minutes gradient (4%B 0-5 minutes; 5-40%B in 5-30 minutes; 40%B 30-35 minutes; 40-50%B 35-38minutes; 50%B 38-40.5 minutes; 50-80%B 40.5-41 minutes; 80%B 41-44 minutes; 80-4%B 44-45 minutes) or a 93 minutes gradient (4-5%B 0-8 minutes; 5-25%B in 8-74 minutes; 25-28%B 74-80 minutes; 28-31%B 80-86 minutes; 31-36%B 86-92 minutes; 36-40%B 92-95 minutes; 40-50%B 95-96 minutes; 50-80%B 96-101 minutes; 80%B 101-104 minutes; 80-4%B 104-104.1 minutes).

Data was acquired using survey scans in a range of 375 to 1500 m/z with a resolution of 120000 and an AGC target value of 4e5. Precursor ions with charge states 2-5 were isolated with a mass selecting quadrupole (isolation window 1.6 m/z) with 10 sec dynamic exclusion (40 seconds for 93 minutes gradient). Precursor ions were fragmented using higher-energy collisional dissociation (HCD) applying a normalized collision energy (NCE) of 30. The maximum injection time was set to 54 ms to collect 2e6 precursor ions. Fragment ion spectra were acquired in the Orbitrap with a resolution of 30000 (FWHM).

5. Experimental Procedures – Organic Synthesis

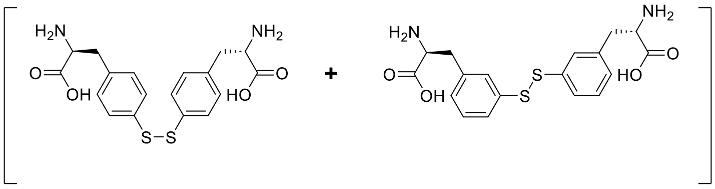

Symmetrical disulfide $\mathbf{3_{ab}}$:

L-phenylanaline (200 mg, 1.21 mmol, 1.0 equiv) was placed in a 10 mL Schlenk-flask under argon. The flask was cooled to −30°C (acetone bath) before slowly adding chlorosulfonic acid (1.20 mL, 18.0 mmol, 15 equiv) using a glass syringe (Hamilton® 10 mL Gastight Syringe, Model 1010 RN) with a large removable needle. The reaction mixture was allowed to warm to room temperature while stirring for 4 h. LC-MS (linear gradient 5 → 95% MeCN, 0.1% TFA, 5 min): R_t (min): 1.65 (ESI-MS (m/z): 264.05 ($M+H^+$)) confirmed the formation of chlorosulfonate **1**.

The reaction mixture was quenched by slowly adding it to a 50 mL round-bottom flask containing H_2O (6.0 mL) which was cooled in an ice-bath. The aqueous mixture was diluted with 1,4-dioxane (7.2 mL) before adding PPh_3 (1.27 g, 4.84 mmol, 4.0 equiv). The reaction mixture was purged with argon, sealed (stopper/parafilm), and stirred for 4 h at 90°C (oil bath). LC-MS (linear gradient 5 → 95% MeCN, 0.1% TFA, 5 min): R_t (min): 1.23 (ESI-MS (m/z): 198.10 ($M+H^+$)) confirmed the formation of aryl thiol **2**.

The crude reaction mixture was partially concentrated *in vacuo* (80 mbar, 40°C; to remove 1,4-dioxane) before diluting the aqueous mixture with 1 M HCl (20 mL) and washing the resulting aqueous layer with Et_2O/EtOAc (1:1, 30 mL). The organic phase was extracted with 1 M HCl (10 mL). The combined aqueous layers were concentrated *in vacuo* (70°C) to obtain a yellow oil. The crude reaction mixture was diluted by addition of DMSO, H_2O/MeCN (0.1 % TFA), filtered, purified by semi-preparative HPLC (C-18; 5 → 60% MeCN, 0.1% TFA) and lyophilized to obtain a mixture of symmetrical disulfides of 4-SH-*L*-Phe ($\mathbf{3_a}$)[3,4] and 3-SH-*L*-Phe ($\mathbf{3_b}$) ($\mathbf{3_a}$:$\mathbf{3_b}$, 4:1; 108.29 mg, 275.91 µmol, 46%) as an off-white solid:

^{1}H NMR (600 MHz, DMSO-d$_6$, 310 K) δ 8.32 (s, 8NH, $\mathbf{3_a}$ + $\mathbf{3_b}$), 7.50 (app. d, *J* = 8.2 Hz, 4H, $\mathbf{3_a}$), 7.47 – 7.43 (m, 4H, $\mathbf{3_b}$), 7.38 – 7.34 (m, 2H, $\mathbf{3_b}$), 7.28 (app. d, *J* = 8.2 Hz, 4H, $\mathbf{3_a}$), 7.22 – 7.19 (m, 2H, $\mathbf{3_b}$), 4.22 – 4.12 (m, 4H, $\mathbf{3_a}$ + $\mathbf{3_b}$), 3.17 – 3.03 (m, 8H, $\mathbf{3_a}$ + $\mathbf{3_b}$);

^{13}C NMR (151 MHz, DMSO-d$_6$, 310 K) δ 170.13 (x4, $\mathbf{3_a}$ + $\mathbf{3_b}$), 136.39 (x2, $\mathbf{3_b}$), 136.11 (x2, $\mathbf{3_b}$), 134.67 (x2, $\mathbf{3_a}$), 134.63 (x2, $\mathbf{3_a}$), 130.52 (x4, $\mathbf{3_a}$), 129.64 (x2, $\mathbf{3_b}$), 128.69 (x2, $\mathbf{3_b}$), 128.07 (x2, $\mathbf{3_b}$), 127.42 (x4, $\mathbf{3_a}$), 125.64 (x2, $\mathbf{3_b}$), 52.98 (x4, $\mathbf{3_a}$ + $\mathbf{3_b}$), 35.52 (x2, $\mathbf{3_b}$), 35.23 (x2, $\mathbf{3_a}$);

LC-MS (linear gradient 5 → 95% MeCN, 0.1% TFA, 5 min): R_t (min): 1.83 (ESI-MS (m/z): 197.12 ($M+2H^{2+}$), 393.26 ($M+H^+$));

HRMS: calculated for $C_9H_{11}NO_2S$ 197.0505 [monomer M+2H]$^{2+}$; found 197.0482;

HRMS: calculated for $C_{18}H_{21}N_2O_4S_2$ 393.0937 $[M+H]^+$; found 393.0941.

Notes:

- The addition of chlorosulfonic acid should NOT be attempted with regular, disposable syringe/needle systems with plastic components due to material instability

- The reaction time for the both the chlorosulfonation step and the reduction with PPh_3 can be extended from a 4-hour period to an overnight reaction if required

- The solubility of aryl disulfide $\mathbf{3_{ab}}$ in aqueous mixtures (before/after HPLC) can also be improved by adding small amounts of TFA.

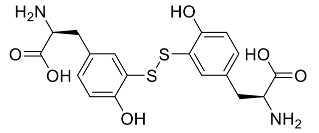

Symmetrical disulfide **6:**

L-tyrosine (226 mg, 1.25 mmol, 1.0 equiv) was placed in a 10 mL Schlenk-flask under argon. The flask was cooled to −30°C (acetone bath) before slowly adding chlorosulfonic acid (1.26 mL, 18.8 mmol, 15 equiv) using a glass syringe (Hamilton® 10 mL Gastight Syringe, Model 1010 RN) with a large removable needle. The reaction mixture was allowed to warm to room temperature while stirring for 4 h. LC-MS (linear gradient 5 → 60% MeCN, 0.1% TFA, 5 min): R_t (min): 1.80 (ESI-MS (m/z): 279.85 ($M+H^+$)) confirmed the formation of chlorosulfonate **4**.

The reaction mixture was quenched by slowly adding it to a 50 mL round-bottom flask containing H_2O (6.25 mL) which was cooled in an ice-bath. The aqueous mixture was diluted with 1,4-dioxane (7.5 mL) before adding PPh_3 (1.31 g, 5.00 mmol, 4.0 equiv). The reaction mixture was purged with argon, sealed (stopper/parafilm), and stirred for 4 h at 90°C (oil bath). LC-MS (linear gradient 5 → 60% MeCN, 0.1% TFA, 5 min): R_t (min): 1.42 (ESI-MS (m/z): 213.99 ($M+H^+$)) confirmed the formation of aryl thiol **5**.

The crude reaction mixture was partially concentrated *in vacuo* (80 mbar, 40°C; to remove 1,4-dioxane) before diluting the aqueous mixture with 1 M HCl (20 mL) and washing the resulting aqueous layer with Et_2O/EtOAc (1:1, 30 mL). The organic phase was extracted with 1 M HCl (10 mL). The combined aqueous layers were concentrated *in vacuo* (70°C) to initially obtain a colourless oil which progressively changed color to yield a brown oil. The crude reaction mixture was diluted by addition of H_2O/MeCN (0.1% TFA), filtered, purified by semi-preparative HPLC (C-18; 5 → 40% MeCN, 0.1% TFA) and lyophilized to obtain aryl disulfide **6** (56.5 mg, 133 µmol, 21%) as an off-white solid:

^{1}H NMR (600 MHz, D_2O) δ 7.31 – 7.23 (m, 2H), 7.18 – 7.08 (m, 2H), 6.96 – 6.86 (m, 2H), 4.18 – 4.08 (m, 2H), 3.21 – 3.11 (m, 2H), 3.09 – 2.99 (m, 2H);

^{13}C NMR (151 MHz, D_2O) δ 171.77 (x2), 154.69 (x2), 133.61 (x2), 131.69 (x2), 126.74 (x2), 122.06 (x2), 116.31 (x2), 54.43 (x2), 34.77 (x2);

^{1}H NMR (600 MHz, DMSO-d_6) δ 7.44 (d, *J* = 1.8 Hz, 2H), 6.98 (dd, *J* = 8.2, 2.0 Hz, 2H), 6.79 (d, *J* = 8.2 Hz, 2H), 3.93 (t, *J* = 6.3 Hz, 2H), 3.01 (dd, *J* = 14.2, 6.1 Hz, 2H), 2.96 (dd, *J* = 14.2, 6.5 Hz, 2H);

^{13}C NMR (151 MHz, DMSO-d_6) δ 170.69 (x2), 154.31 (x2), 129.75 (x4), 126.63 (x2), 122.00 (x2), 115.24 (x2), 53.88 (x2), 35.19 (x2);

LC-MS (linear gradient 5 → 60% MeCN, 0.1% TFA, 5 min): R_t (min): 1.78 (ESI-MS (m/z): 213.01 ($M+2H^{2+}$), 424.95 ($M+H^+$));

LC-MS (linear gradient 5 → 95% MeCN, 0.1% TFA, 15 min): R_t (min): 1.71 (ESI-MS (m/z): 425.27 ($M+H^+$));

HRMS: calculated for $C_{18}H_{22}NO_3S$ 213.0454 $[M+2H]^{2+}$; found 213.0355.

HRMS: calculated for $C_{18}H_{21}N_2O_6S_2$ 425.0836 $[M+H]^+$; found 425.0814.

<u>Notes:</u>

-The addition of chlorosulfonic acid should <u>NOT</u> be attempted with regular, disposable syringe/needle systems with plastic components due to material instability

- The reaction time for the both the chlorosulfonation step and the reduction with PPh_3 can be extended from a 4-hour period to an overnight reaction if required

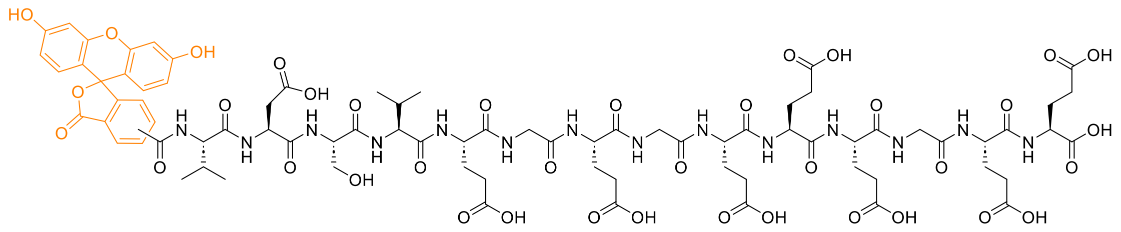

5,6-carboxyfluorescein Tub-tag® (CF-Tub-tag®) **7** was prepared as previously described by Schumacher et al.[5,6]

The Tub-tag® peptide sequence (VDSVEGEGEEEGEE) was synthesized by standard automated Fmoc SPPS in a linear synthesis. Starting from Fmoc-L-Glu(tBu)-Wang resin (50 µmol, 1.0 equiv), single peptide couplings (5.0 equiv Fmoc-AA, 5.0 equiv HCTU, 5.0 equiv Oxyma, 10 equiv DIPEA for 40 min), Fmoc deprotections (20% piperidine), and washing steps were performed in DMF. Standard sidechain protections were used (Asp-OtBu, Ser-OtBu, Glu-OtBu). After the final Fmoc deprotection, the resin material was manually washed with DMF (3 x 3 mL), followed by adding DMF (3 mL) containing 5,6-carboxyfluorescein (76 mg, 0.20 mmol, 4.0 equiv), HOBt (31 mg, 0.20 mmol, 4.0 equiv), HBTU (76 mg, 0.20 mmol, 4.0 equiv) and DIPEA (70 µL, 0.40 mmol, 8.0 equiv). The reaction mixture was shaken for 1 h at room temperature and washed with DMF (3 x 3 mL). The same coupling conditions were repeated for 1 h before washing the resin with DMF (3 x 3 mL) and DCM (3 x 3 mL). Peptide deprotection was initiated by adding a mixture of TFA/TIS/H_2O (95 : 2.5 : 2.5; 3 mL) and shaking the reaction for 4 h at room temperature. The crude reaction mixture was added dropwise to pre-cooled (-20°C), anhydrous Et_2O (45 mL) in a 50 mL falcon tube. The suspension was centrifuged (5000 rcf, 5 min, 4°C) and the supernatant was decanted to obtain the crude peptide precipitate which was dried over a stream of N_2. The crude material was redissolved in a mixture of DMSO and H_2O/MeCN (0.1% TFA) using sonication at 40°C, purified by semi-preparative HPLC (C-18; 5 → 95% MeCN, 0.1% TFA) and lyophilized to obtain CF-Tub-tag® **7** (24.18 mg, 13.06 µmol, 26%) as a yellow solid:

LC-MS (linear gradient 5 → 95% MeCN, 0.1% TFA, 15 min): R_t (min): 4.61 (ESI-MS (m/z): 926.84 ($M+2H^{2+}$), 938.02 ($M+H+Na^{2+}$);

HRMS: calculated for $C_{79}H_{100}N_{14}O_{38}$ 926.3156 $[M+2]^{2+}$; found 926.3209.

Spectroscopic data was in agreement with literature.[5,6]

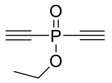

Ethyl diethynyl phosphinate was prepared as previously described by Stieger et al.[1,2]

A 50 mL Schlenk-flask was charged with anhydrous THF (5 mL) and ethyl dichlorophosphite (575 µL, 5.03 mmol, 1.0 equiv) under argon. The solution was cooled to -78°C (acetone bath) before adding ethynylmagnesium bromide (22.1 mL, 0.5 M in THF, 11.1 mmol, 2.2 Eq) dropwise. The reaction mixture was allowed to warm to room temperature and stirred for 2 h. Subsequently, the reaction mixture was poured into an ice-cold solution of H_2O_2 (40 mL, 1.5% Wt, 20 mmol, 3.9 equiv). The crude reaction mixture was extracted with DCM (3 x 50 mL). The combined organic layers were washed with H_2O (50 mL), dried over $MgSO_4$, filtered, concentrated *in vacuo* (including co-evaporation with $CHCl_3$) to obtain the title compound (605 mg, 4.26 mmol, 85 %) as a dark oil which solidified over time:

^{1}H NMR (600 MHz, $CDCl_3$) δ 4.26 (dq, *J* = 9.5, 7.0 Hz, 2H), 3.12 (d, *J* = 12.7 Hz, 2H), 1.44 (t, *J* = 7.0 Hz, 3H);

^{31}P NMR (243 MHz, $CDCl_3$) δ -22.70.

Spectroscopic data was in agreement with literature.[1,2]

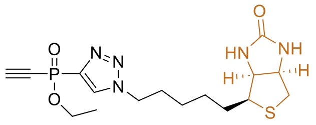

ETP-Biotin **9** was synthesized according to a modified procedure (Stieger et al.).[2]

Biotin-azide[7] (20.5 mg, 80.3 µmol, 1.0 equiv) was dissolved in a mixture of DMSO (400 µL) and MeCN (200 µL) before mixing the solution with 0.2 M phosphate buffer pH 6.0 (400 µL). Ethyl diethynyl phosphinate[1,2] (114 mg, 803 µmol, 10 equiv) was added to the solution.

MeCN (400 µL) was added to dry CuBr (≥ 5 mg) to obtain a green, saturated solution of CuBr. THPTA (tris((1-benzyl-4-triazolyl)methyl)amine; 100 mM in PBS pH 7.4, 60 µL, 6.0 µmol, 7.5 mol%) was added, and the resulting colorless solution was added to the reaction mixture to initiate CuAAC. The reaction mixture was stirred at room temperature for 15 min and subsequently quenched by addition of TFA (100 µL). The crude reaction mixture was diluted by addition of H_2O/MeCN (0.1 % TFA), purified by semi-preparative HPLC (C-18; 5 → 60% MeCN, 0.1% TFA) and lyophilized to obtain ETP-Biotin **9** (19.8 mg, 49.9 µmol, 62%) as a solid:

LC-MS (linear gradient 5 → 95% MeCN, 0.1% TFA, 15 min): R_t (min): 3.98 (ESI-MS (m/z): 398.33 ($M+H^+$), 420.34 ($M+Na^+$)).

Spectroscopic data was in agreement with literature.[2]

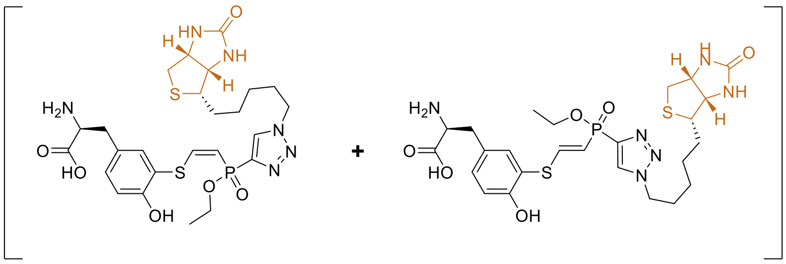

Aryl thiol-ETP-biotin conjugate **10-Z/E:**

Aryl disulfide **6** (5.45 mg, 12.8 µmol, 1.0 equiv) was dissolved in a mixture of 0.2 M phosphate buffer pH 6.0 (1800 µL) and MeCN (200 µL) before adding TCEP (103 µL, 500 mM in PBS pH 7.4, 51.4 µmol, 4.0 equiv). The solution was homogenized in a round-bottom flask for 2 min, after which ETP-Biotin **9** (260 µL, 100 mM in DMSO, 26.0 µmol, 2.03 equiv) was added. The reaction mixture was stirred at room temperature for 10 min and subsequently quenched by addition of TFA (50 µL). The crude reaction mixture was diluted by addition of H_2O (0.1 % TFA), purified by semi-preparative HPLC (C-18; 5 → 95% MeCN, 0.1% TFA) and lyophilized to obtain aryl thiol-Biotin ETP conjugate **10-Z$_{ab}$/E$_{ab}$** (**10-Z$_{ab}$:10-E$_{ab}$**, ± 95:5; 6.67 mg, 10.9 µmol, 43%) as a solid:

^{1}H NMR (600 MHz, DMSO-d$_6$) δ 10.35 (s, 2OH, **10-E$_{ab}$**), 10.24 (s, 2OH, **10-Z$_{ab}$**), 8.66 (s, 2H, **10-Z$_{ab}$**), 8.57 (s, 2H, **10-E$_{ab}$**), 7.54 (dd, *J* = 43.2, 12.4, 1H, **10-Z$_a$**), 7.54 (dd, *J* = 43.2, 12.4, 1H, **10-Z$_b$**), 7.45 (dd, *J* = 19.8, 16.6 Hz, 2H, **10-E$_{ab}$**), 7.26 (d, *J* = 2.3 Hz, 2H, **10-E$_{ab}$**), 7.20 (d, *J* = 2.5 Hz, 1H, **10-Z$_a$**), 7.20 (d, *J* = 2.5 Hz, 1H, **10-Z$_b$**), 7.04 (dd, *J* = 8.4, 2.5 Hz, 1H, **10-Z$_a$**), 7.04 (dd, *J* = 8.4, 2.5 Hz, 1H, **10-Z$_b$**), 6.95 (d, *J* = 8.4 Hz, 2H, **10-E$_{ab}$**), 6.86 (d, *J* = 8.2 Hz, 2H, **10-Z$_{ab}$**), 6.43 (s, 4NH, **10-Z$_{ab}$/E$_{ab}$**), 6.37 (s, 4NH, **10-Z$_{ab}$/E$_{ab}$**), 5.98 (dd, *J* = 18.2, 12.4, 1H, **10-Z$_a$**), 5.98 (dd, *J* = 18.2, 12.4, 1H, **10-Z$_b$**), 5.77 (dd, *J* = 19.1, 16.5 Hz, 2H, **10-E$_{ab}$**), 4.43 (t, *J* = 7.1 Hz, 4H, **10-Z$_{ab}$**), 4.40 (t, *J* = 7.1 Hz, 4H, **10-E$_{ab}$**), 4.30 (m, 4H, **10-Z$_{ab}$/E$_{ab}$**), 4.14 – 4.09 (m, 4H, **10-Z$_{ab}$/E$_{ab}$**), 4.08 – 3.85 (m, 12H, **10-Z$_{ab}$/E$_{ab}$**), 3.09 – 3.00 (m, 8H, **10-Z$_{ab}$/E$_{ab}$**), 2.95 (m, 4H, **10-Z$_{ab}$/E$_{ab}$**), 2.83 – 2.78 (m, 4H, **10-Z$_{ab}$/E$_{ab}$**), 2.57 (d, *J* = 12.4 Hz, 4H, **10-Z$_{ab}$/E$_{ab}$**), 1.84 (m, 8H, **10-Z$_{ab}$/E$_{ab}$**), 1.58 (m, 4H, **10-Z$_{ab}$/E$_{ab}$**), 1.48 – 1.29 (m, 12H, **10-Z$_{ab}$/E$_{ab}$**), 1.27 (t, *J* = 7.0 Hz, 6H, **10-Z$_{ab}$**), 1.25 – 1.22 (m, 4H, **10-Z$_{ab}$/E$_{ab}$**), 1.20 (t, *J* = 7.0 Hz, 6H, **10-E$_{ab}$**).

1H NMR (^{31}P-decoupled, 600 MHz, DMSO-d$_6$) δ 10.35 (s, 2OH, **10-E$_{ab}$**), 10.24 (s, 2OH, **10-Z$_{ab}$**), 8.66 (s, 2H, **10-Z$_{ab}$**), 8.57 (s, 2H, **10-E$_{ab}$**), 7.54 (d, *J* = 12.4, 1H, **10-Z$_a$**), 7.54 (d, *J* = 12.4, 1H, **10-Z$_b$**), 7.45 (d, *J* = 16.6 Hz, 2H, **10-E$_{ab}$**), 7.26 (d, *J* = 2.3 Hz, 2H, **10-E$_{ab}$**), 7.20 (d, *J* = 2.5 Hz, 1H, **10-Z$_a$**), 7.20 (d, *J* = 2.5 Hz, 1H, **10-Z$_b$**), 7.04 (dd, *J* = 8.4, 2.5 Hz, 1H, **10-Z$_a$**), 7.04 (dd, *J* = 8.4, 2.5 Hz, 1H, **10-Z$_b$**), 6.95 (d, *J* = 8.4 Hz, 2H, **10-E$_{ab}$**), 6.86 (d, *J* = 8.2 Hz, 2H, **10-Z$_{ab}$**), 6.43 (s, 4NH, **10-Z$_{ab}$/E$_{ab}$**), 6.37 (s, 4NH, **10-Z$_{ab}$/E$_{ab}$**), 5.98 (d, *J* = 12.4, 1H, **10-Z$_a$**), 5.98 (d, *J* = 12.4, 1H, **10-Z$_b$**), 5.77 (d, *J* = 16.5 Hz, 2H, **10-E$_{ab}$**), 4.43 (t, *J* = 7.1 Hz, 4H, **10-Z$_{ab}$**), 4.40 (t, *J* = 7.1 Hz, 4H, **10-E$_{ab}$**), 4.30 (m, 4H, **10-Z$_{ab}$/E$_{ab}$**), 4.14 – 4.09 (m, 4H, **10-Z$_{ab}$/E$_{ab}$**), 4.08 – 3.85 (m, 12H, **10-Z$_{ab}$/E$_{ab}$**), 3.09 – 3.00 (m, 8H, **10-Z$_{ab}$/E$_{ab}$**), 2.95 (m, 4H, **10-Z$_{ab}$/E$_{ab}$**), 2.83 – 2.78 (m, 4H, **10-Z$_{ab}$/E$_{ab}$**), 2.57 (d, *J* = 12.4 Hz, 4H, **10-Z$_{ab}$/E$_{ab}$**), 1.84 (m, 8H, **10-Z$_{ab}$/E$_{ab}$**), 1.58 (m, 4H, **10-Z$_{ab}$/E$_{ab}$**), 1.48 – 1.29 (m, 12H, **10-Z$_{ab}$/E$_{ab}$**), 1.27 (t, *J* = 7.0 Hz, 6H, **10-Z$_{ab}$**), 1.25 – 1.22 (m, 4H, **10-Z$_{ab}$/E$_{ab}$**), 1.20 (t, *J* = 7.0 Hz, 6H, **10-E$_{ab}$**).

^{31}P NMR (243 MHz, DMSO-d$_6$) δ 18.94;

^{13}C NMR (151 MHz, DMSO-d$_6$) δ 170.47 (**10-Z$_a$**), 170.46 (**10-Z$_b$**), 170.42 (x2, **10-E$_{ab}$**), 162.77 (x4, **10-Z$_{ab}$/E$_{ab}$**), 155.14 (x2, **10-Z$_a$/E$_a$**), 155.12 (x2, **10-Z$_b$/E$_b$**), 150.85 (x4, **10-Z$_{ab}$/E$_{ab}$**), 139.69 (d, *J* = 177.7 Hz, x2, **10-Z$_a$/E$_a$**), 139.69 (d, *J* = 177.7 Hz, x2, **10-Z$_b$/E$_b$**), 132.67 (x2, **10-Z$_a$/E$_a$**), 132.65 (x2, **10-Z$_b$/E$_b$**), 131.04 (d, *J* = 29.2 Hz, x2, **10-Z$_a$/E$_a$**), 131.04 (d, *J* = 29.2 Hz, x2, **10-Z$_b$/E$_b$**), 130.61 (x2, **10-Z$_a$/E$_a$**), 130.60 (x2, **10-Z$_b$/E$_b$**), 126.25 (x2, **10-Z$_a$/E$_a$**), 126.23 (x2, **10-Z$_b$/E$_b$**), 120.14 (x2, **10-Z$_a$/E$_a$**), 120.12 (x2, **10-Z$_b$/E$_b$**), 115.85 (x2, **10-Z$_a$/E$_a$**), 115.84 (x2, **10-Z$_b$/E$_b$**), 113.31 (d, *J* = 144.8 Hz, x2, **10-Z$_a$/E$_a$**), 113.31 (d, *J* = 144.8 Hz, x2, **10-Z$_b$/E$_b$**), 61.02 (x4, **10-Z$_{ab}$/E$_{ab}$**), 60.68 (d, *J* = 5.6 Hz, x2, **10-Z$_a$/E$_a$**), 60.68 (d, *J* = 5.6 Hz, x2, **10-Z$_b$/E$_b$**), 59.22 (x4, **10-Z$_{ab}$/E$_{ab}$**), 55.43 (x4, **10-Z$_{ab}$/E$_{ab}$**), 53.56 (x2, **10-Z$_a$/E$_a$**), 53.55 (x2, **10-Z$_b$/E$_b$**), 49.48 (x4, **10-Z$_{ab}$/E$_{ab}$**), 40.06 (x2, **10-E$_{ab}$**), 39.86 (x2, **10-Z$_{ab}$**), 34.92 (x2, **10-Z$_a$/E$_a$**), 34.91 (x2, **10-Z$_b$/E$_b$**), 29.41 (x2, **10-Z$_{ab}$**), 29.37 (x2, **10-E$_{ab}$**), 28.11 (x4, **10-Z$_{ab}$/E$_{ab}$**), 27.90 (x4, **10-**

Z_{ab}/E_{ab}), 25.87 (x2, **10-E_{ab}**), 25.84 (x2, **10-Z_{ab}**), 16.37 (d, *J* = 6.3 Hz, x2, **10-Z_a/E_a**), 16.30 (d, *J* = 6.3 Hz, x2, **10-Z_b/E_b**).

LC-MS (linear gradient 5 → 95% MeCN, 0.1% TFA, 15 min): R_t (min): 4.06 (ESI-MS (m/z): 306.19 ($M+2H^{2+}$), 611.42 ($M+H^+$));

HRMS: calculated for $C_{25}H_{36}N_6O_6PS_2$ 611.1870 $[M+H]^+$; found 611.1833.

Re-purification of the Z/E-mixture by semi-preparative HPLC (C-18; 5 → 60% MeCN, 0.1% TFA) enabled isolation of the Z-isomer **10-Z_{ab}** (4.42 mg, 7.24 µmol, 29%):

^{1}H NMR (600 MHz, D_2O) δ 8.65 (s, 1H, **10-Z_a**), 8.65 (s, 1H, **10-Z_b**), 7.54 (dd, *J* = 45.3, 12.3, 1H, **10-Z_a**), 7.54 (dd, *J* = 45.3, 12.3, 1H, **10-Z_b**), 7.23 (dd, *J* = 8.3, 2.1 Hz, 1H, **10-Z_a**), 7.22 (dd, *J* = 8.3, 2.1 Hz, 1H, **10-Z_b**), 7.18 (d, *J* = 2.1, 1H, **10-Z_a**), 7.17 (d, *J* = 2.1, 1H, **10-Z_b**), 6.98 (d, *J* = 8.3, 1H, **10-Z_a**), 6.98 (d, *J* = 8.3, 1H, **10-Z_b**), 6.20 (dd, *J* = 15.4, 12.2, 1H, **10-Z_a**), 6.20 (dd, *J* = 15.4, 12.2, 1H, **10-Z_b**), 4.62 – 4.50 (m, 6H, **10-Z_{ab}**), 4.30 – 4.16 (m, 6H, **10-Z_{ab}**), 3.27 – 3.21 (m, 2H, **10-Z_{ab}**), 3.19 – 3.13 (m, 2H, **10-Z_{ab}**), 3.13 – 3.07 (m, 2H, **10-Z_{ab}**), 2.94 – 2.86 (m, 2H, **10-Z_{ab}**), 2.72 (d, *J* = 13.1 Hz, 2H, **10-Z_{ab}**), 1.96 – 1.88 (m, 4H, **10-Z_{ab}**), 1.56 – 1.46 (m, 2H, **10-Z_{ab}**), 1.40 (t, *J* = 7.0 Hz, 6H, **10-Z_{ab}**), 1.37 – 1.31 (m, 2H, **10-Z_{ab}**), 1.31 – 1.14 (m, 8H, **10-Z_{ab}**).

^{31}P NMR (243 MHz, D_2O) δ 22.96.

Notes:
*-We propose that the perceived 'signal doubling' for **10-Z_{ab}** (^{1}H) is caused by the presence of two diastereoisomers (due to the racemic phosphorus atom found in reagent **9**). This is further outlined in **Figure S5 – S7**.*

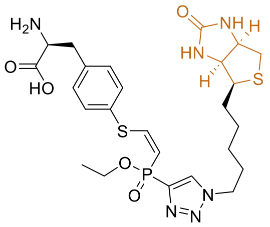

Aryl thiol-ETP-biotin conjugate **11-Z:**

Aryl disulfide **3_{ab}** (1.50 mg, 3.80 µmol, 1.0 equiv) was partially dissolved in a mixture of 0.2 M phosphate buffer pH 7.0 (1900 µL), MeCN (100 µL) and DMSO (100 µL) upon sonication. TCEP (31 µL, 500 mM in PBS pH 7.4, 15 µmol, 4.0 equiv) was added while stirring the solution, which improved the overall solubility. The solution was homogenized further in a round-bottom flask for 2 min, after which ETP-Biotin **9** (380 µL, 20 mM in DMSO, 7.6 µmol, 2.0 equiv) was added. The reaction mixture was stirred at room temperature for 15 min and subsequently quenched by addition of TFA (50 µL). The crude reaction mixture was diluted by addition of H_2O (0.1 % TFA), purified by semi-preparative HPLC (C-18; 5 → 60% MeCN, 0.1% TFA) and lyophilized to obtain the main product, aryl thiol-Biotin ETP conjugate **11-Z** (1.05 mg, 1.77 µmol, 23%), as a solid:

^{1}H NMR (600 MHz, DMSO-d_6) δ 8.67 (s, 1H), 8.26 (s, 2NH), 7.59 (dd, *J* = 42.3, 12.4 Hz, 1H), 7.43 (d, *J* = 8.2 Hz, 2H), 7.30 (d, *J* = 8.4 Hz, 2H), 6.41 (s, 1NH), 6.35 (s, 1NH), 6.07 (dd, *J* = 17.8, 12.3 Hz, 1H), 4.43 (t, *J* = 7.1 Hz, 2H), 4.30 (dd, *J* = 7.9, 4.7 Hz, 1H), 4.21 (t, *J* = 6.6 Hz, 1H), 4.13 – 4.10 (m, 1H), 4.07 – 3.94 (m, 2H), 3.12 (dd, *J* = 14.4, 6.3 Hz, 2H), 3.09 – 3.04 (m, 2H), 2.81 (dd, *J* = 12.4, 5.1 Hz, 1H), 2.57 (d, *J* = 12.4 Hz, 1H), 1.84 (m, 2H), 1.62 – 1.54 (m, 1H), 1.46 – 1.29 (m, 3H), 1.27 (t, *J* = 7.1 Hz, 3H), 1.25 – 1.18 (m, 2H);

^{1}H NMR (^{31}P-decoupled, 600 MHz, DMSO-d_6) δ 8.67 (s, 1H), 8.26 (s, 2NH), 7.59 (d, *J* = 12.4 Hz, 1H), 7.43 (d, *J* = 8.2 Hz, 2H), 7.30 (d, *J* = 8.4 Hz, 2H), 6.41 (s, 1HN), 6.35 (s, 1NH), 6.07 (d, *J* = 12.4 Hz, 1H), 4.43 (t, *J* = 7.1 Hz, 2H), 4.30 (dd, *J* = 7.9, 4.8 Hz, 1H), 4.21 (t, *J* = 6.7 Hz, 1H), 4.13 – 4.09 (m, 1H), 4.04 (dq, *J* = 10.2, 7.0 Hz, 1H), 3.98 (dq, *J* = 10.2, 7.0 Hz, 1H), 3.12 (dd, *J* = 14.4, 6.3 Hz, 2H), 3.09 – 3.04 (m, 2H), 2.81 (dd, *J* = 12.4, 5.1 Hz, 1H), 2.57 (d, *J* = 12.4 Hz, 1H), 1.84 (m, 2H), 1.62 – 1.55 (m, 1H), 1.46 – 1.29 (m, 3H), 1.27 (t, *J* = 7.0 Hz, 3H), 1.25 – 1.18 (m, 2H);

^{31}P NMR (243 MHz, DMSO-d_6) δ 18.64;

LC-MS (linear gradient 5 → 95% MeCN, 0.1% TFA, 15 min): R_t (min): 3.99 (ESI-MS (m/z): 595.19 (M+H$^+$), 1189.60 (2M+H$^+$));

HRMS: calculated for $C_{25}H_{36}N_6O_5PS_2$ 595.1921 [M+H]$^+$; found 595.1922.

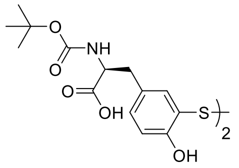

Boc-protected aryl disulfide **17:**

*The procedure for aryl disulfide **6** was initially repeated at a larger scale:*

L-tyrosine (1.00 g, 5.52 mmol, 1.0 equiv) was placed in a 10 mL Schlenk-flask under argon. The flask was cooled to −30°C (acetone bath) before slowly adding chlorosulfonic acid (5.29 mL, 79.0 mmol, 15 equiv) using a glass syringe (Hamilton® 10 mL Gastight Syringe, Model 1010 RN) with a large removable needle. The reaction mixture was allowed to warm to room temperature while stirring for 4 h. LC-MS (linear gradient 5 → 60% MeCN, 0.1% TFA, 5 min): R_t (min): 1.80 (ESI-MS (m/z): 279.85 ($M+H^+$)) confirmed the formation of chlorosulfonate **4**.

The reaction mixture was quenched by slowly adding it (dropping funnel) to a 250 mL round-bottom flask containing H_2O (26.5 mL) which was cooled in an ice-bath. The aqueous mixture was diluted with 1,4-dioxane (32 mL) before adding PPh_3 (5.79 g, 22.1 mmol, 4.0 equiv). The reaction mixture was purged with argon, sealed (stopper/parafilm), and stirred for 4 h at 90°C (oil bath). LC-MS (linear gradient 5 → 60% MeCN, 0.1% TFA, 5 min): R_t (min): 1.42 (ESI-MS (m/z): 213.99 ($M+H^+$)) confirmed the formation of aryl thiol **5**.

The crude reaction mixture was partially concentrated *in vacuo* (80 mbar, 40°C; to remove 1,4-dioxane) before diluting the aqueous mixture with 1 M HCl (100 mL) and washing the resulting aqueous layer with Et_2O/EtOAc (1:1, 3 x 60 mL). The aqueous layer was concentrated *in vacuo* (70°C) to initially obtain a colourless oil which progressively changed color to yield a brown oil, containing aryl disulfide **6**.

The crude oil was redissolved in H_2O / 1,4-dioxane (1:1, 50 mL), cooled on ice and basified by slowly adding NaOH (satd., ~ 20 - 30 mL) until the pH was adjusted to 9. An equal volume of 1,4-dioxane (~ 20 - 30 mL) and di-*tert*-butyldicarbonate (Boc_2O; 12.1 g, 55.2 mmol, 10 equiv) were added, and the reaction mixture was stirred overnight at room temperature. The crude reaction mixture was partially concentrated *in vacuo* (80 mbar, 40°C; to remove 1,4-dioxane), after which the aqueous phase was washed with EtOAc (3 x 60 mL). Subsequently, the aqueous phase was acidified to pH 4 by slowly adding 37% HCl (~ 7 mL) under stirring at 0°C (ice bath). The acidified aqueous phase was extracted with EtOAc (3 x 60 mL). The combined organic layers were dried over $MgSO_4$, filtered, concentrated *in vacuo* and purified by silica gel chromatography (10 → 40% EtOAc in hexane, 5% AcOH) to obtain Boc-protected aryl disulfide **17** (469.72 mg, 781.89 µmol, 27% over four steps) as a pale-yellow foam: R_f = 0.5 (60% EtOAc in hexane, 5% AcOH):

^{1}H NMR (600 MHz, DMSO-d$_6$) δ 7.36 (s, 2H), 6.95 (d, *J* = 8.1 Hz, 2H), 6.89 (d, *J* = 8.2 Hz, 2NH), 6.73 (d, *J* = 8.2 Hz, 2H), 4.03 – 3.94 (m, 2H), 2.87 (dd, *J* = 13.7, 4.3 Hz, 2H), 2.71 (dd, *J* = 13.7, 10.0 Hz, 2H), 1.32 (s, 18H);

^{13}C NMR (151 MHz, DMSO-d$_6$) δ 173.54 (x2), 155.37 (x2), 153.69 (x2), 129.60 (x2), 129.32 (x2), 129.15 (x2), 121.40 (x2), 115.00 (x2), 78.04 (x2), 55.36 (x2), 35.76 (x2), 28.18 (x6);

LC-MS (linear gradient 5 → 95% MeCN, 0.1% TFA, 15 min): R_t (min): 6.23 (ESI-MS (m/z): 469.00 (M-Boc-Boc+Na^++Na^+), 647.25 ($M+Na^+$);

HRMS: calculated for $C_{28}H_{37}N_2O_{10}S_2$ 625.1884 $[M+H]^+$; found 625.1901.

<u>Notes:</u>

-The addition of chlorosulfonic acid should <u>NOT</u> be attempted with regular, disposable syringe/needle systems with plastic components due to material instability

- The reaction time for the both the chlorosulfonation step and the reduction with PPh_3 can be extended from a 4-hour period to an overnight reaction if required

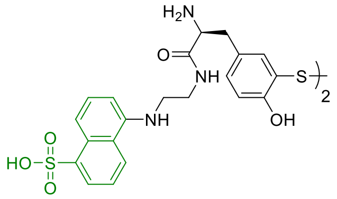

EDANS-aryl disulfide **13:**

Boc-protected aryl disulfide **17** (30.74 mg, 49.21 µmol, 1.0 equiv), 5-((2-Aminoethyl)aminonaphthalene-1-sulfonic acid (EDANS, sodium salt; 70.97 mg, 246.2 µmol, 5.0 eq), anhydrous DIPEA (84.0 µL, 482 µmol, 9.8 eq) were dissolved in anhydrous DMF (700 µL). HCTU (100.44 mg, 242.79 µmol, 4.9 equiv) was added and the reaction mixture was stirred overnight at room temperature. Subsequently, TFA (3 mL) was added to obtain a crude, brown reaction mixture, which was diluted by addition of H_2O (0.1 % TFA), filtered, purified by semi-preparative HPLC (C-18; 5 → 95% MeCN, 0.1% TFA) and lyophilized to obtain EDANS-aryl disulfide **13** (25.12 mg, 15 µmol, 59% over two steps) as a solid:

^{1}H NMR (600 MHz, DMSO-d_6) δ 10.17 (s, 2OH), 8.50 (t, *J* = 5.5 Hz, 2NH), 8.14 (d, *J* = 8.5 Hz, 2H), 8.07 (s, 2NH), 8.07 (s, 2NH), 8.03 (d, *J* = 8.4 Hz, 2H), 7.93 (d, *J* = 7.0 Hz, 2H), 7.29 (t, *J* = 7.8 Hz, 2H), 7.27 – 7.20 (m, 4H), 6.85 (d, *J* = 8.0 Hz, 2H), 6.73 (d, *J* = 8.1 Hz, 2H), 6.52 (d, *J* = 7.6 Hz, 2H), 3.85 – 3.79 (m, 2H), 3.40 – 3.32 (m, 2H), 3.31 – 3.24 (m, 2H), 3.23 – 3.10 (m, 4H), 2.85 (dd, *J* = 14.0, 6.9 Hz, 2H), 2.77 (dd, *J* = 14.0, 7.1 Hz, 2H).

^{13}C NMR (151 MHz, DMSO-d_6) δ 168.26 (x2), 154.13 (x2), 143.71 (x2), 143.50 (x2), 130.10 (x2), 129.24 (x2), 128.91 (x2), 126.38 (x2), 125.95 (x2), 124.50 (x2), 123.68 (x2), 122.99 (x2), 122.43 (x2), 121.70 (x2), 115.88 (x2), 115.25 (x2), 103.14 (x2), 53.79 (x2), 42.80 (x2), 37.67 (x2), 36.33 (x2);

LC-MS (linear gradient 5 → 95% MeCN, 0.1% TFA, 15 min): R_t (min): 2.94 (ESI-MS (m/z): 461.15 ($M+2H^{2+}$), 921.32 ($M+H^+$));

HRMS: calculated for $C_{42}H_{46}N_6O_{10}S_4$ 461.1074 $[M+2H]^{2+}$; found 461.1062;

HRMS: calculated for $C_{42}H_{45}N_6O_{10}S_4$ 921.2075 $[M+H]^+$; found 921.2034.

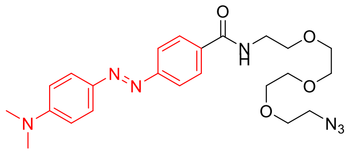

DABCYL azide **19:**

DABCYL acid (20 mg, 74 µmol, 1.0 equiv) and HATU (28 mg, 74 µmol, 1.0 equiv) were dissolved in DMF (400 µL) followed by the addition of DIPEA (39 µL, 0.22 mmol, 3.0 equiv). After 2 min, 11-azido-3,6,9-trioxaundecan-1-amine (18 µL, 20 mg, 92 µmol, 1.2 equiv) was added and the reaction mixture was stirred for 15 min at room temperature. The crude reaction mixture was purified by semi-preparative HPLC (C-18; 5 → 95% MeCN, 0.1% TFA) and lyophilized to obtain DABCYL azide **19** (25.3 mg, 53.9 µmol, 73%) as a purple solid:

^{1}H NMR (600 MHz, $CDCl_3$) δ 8.06 (d, *J* = 8.9 Hz, 2H), 7.96 (d, *J* = 8.7 Hz, 2H), 7.91 (d, *J* = 8.5 Hz, 2H), 6.93 (t, *J* = 4.5 Hz, 1NH), 6.89 (d, *J* = 8.9 Hz, 2H), 3.73 – 3.66 (m, 10H), 3.66 – 3.60 (m, 4H), 3.34 (t, *J* = 5.1 Hz, 2H), 3.18 (s, 6H);

^{13}C NMR (151 MHz, $CDCl_3$) δ 167.26, 153.68, 152.55, 143.03, 134.77 (x2), 128.29 (x2), 127.80, 121.80 (x2), 113.23 (x2), 70.82 (x2), 70.72, 70.42, 70.18, 69.84, 50.80, 41.22 (x2), 40.10;

LC-MS (linear gradient 5 → 95% MeCN, 0.1% TFA, 5 min): R_t (min): 2.51 (ESI-MS (m/z): 470.45 (M+H$^+$));

HRMS: calculated for $C_{23}H_{32}N_7O_4$ 470.2510 [M+H]$^+$; found 470.2513.

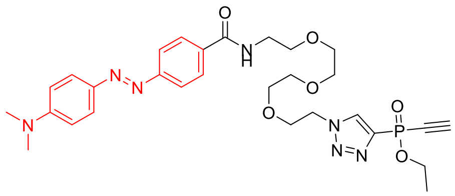

ETP-DABCYL **15:**

DABCYL azide **19** (12 mg, 26 µmol, 1.0 equiv) was dissolved in a mixture of MeCN (200 µL) and 0.2 M phosphate buffer pH 6.0 (200 µL). Ethyl diethynyl phosphinate[1,2] (18 mg, 0.13 mmol, 5.0 equiv) was added. MeCN (200 µL) was added to dry CuBr (≥ 3 mg) to obtain a green, saturated solution of CuBr. THPTA (tris((1-benzyl-4-triazolyl)methyl)amine; 100 mM in PBS pH 7.4, 30 µL, 3.0 µmol, 12 mol%) was added, and the resulting colorless solution was added to the reaction mixture to initiate CuAAC. The reaction mixture was stirred at room temperature for 5 min and subsequently quenched by addition of TFA (50 µL). The crude reaction mixture was diluted by addition of H_2O/MeCN (0.1 % TFA), purified by semi-preparative HPLC (C-18; 5 → 95% MeCN, 0.1% TFA) and lyophilized to obtain ETP-DABCYL **15** (9.2 mg, 15 µmol, 59%) as a solid:

^{1}H NMR (600 MHz, DMSO-d_6) δ 8.67 (s, 1H), 8.59 (t, *J* = 5.6 Hz, 1NH), 7.98 (d, *J* = 8.5 Hz, 2H), 7.85 – 7.78 (m, 4H), 6.85 (d, *J* = 9.2 Hz, 2H), 4.71 (d, *J* = 11.6 Hz, 1H), 4.62 (t, *J* = 5.3 Hz, 2H), 4.20 – 4.11 (m, 2H), 3.85 (t, *J* = 5.3 Hz, 2H), 3.57 – 3.50 (m, 6H), 3.50 – 3.46 (m, 4H), 3.43 (q, *J* = 5.9 Hz, 2H), 3.08 (s, 6H), 1.30 (t, *J* = 7.0 Hz, 3H);

^{13}C NMR (151 MHz, DMSO-d_6) δ 165.71, 153.93, 152.87, 142.65, 138.58 (d, *J* = 213.6 Hz), 134.65, 131.90 (d, *J* = 34.3 Hz), 128.32 (x2), 125.12 (x2), 121.47 (x2), 111.63 (x2), 94.54 (d, *J* = 39.5 Hz), 77.26 (d, *J* = 219.9 Hz), 69.70, 69.59 (x2), 69.49, 68.87, 68.22, 62.63 (d, *J* = 6.2 Hz), 49.62, 39.94, 16.03 (d, *J* = 6.6 Hz);

^{31}P NMR (243 MHz, DMSO) δ -4.77;

LC-MS (linear gradient 5 → 95% MeCN, 0.1% TFA, 5 min): R_t (min): 2.33 (ESI-MS (m/z): 306.76 (M+2H^{2+}), 612.53 (M+2H^{2+}));

HRMS: calculated for $C_{29}H_{39}N_7O_6P$ 612.2694 [M+H]$^+$; found 612.2670.

Note: the ^{13}C NMR signals of the terminal N-methyl groups (CH_3 x 2) overlap with the solvent peak (δ 39.52 ppm). This is confirmed on the HSQC measurement.

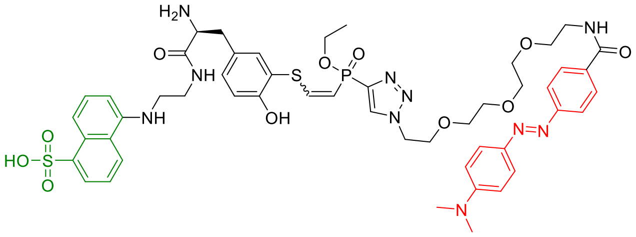

EDANS aryl thiol ETP-DABCYL conjugate **16:**

EDANS aryl disulfide **13** (200 µL, 6.25 mM in DMSO, 1.25 µmol, 1.0 equiv) was added to a mixture of 0.2 M phosphate buffer pH 6.0 (2000 µL), MeCN (400 µL) and TCEP (10 µL, 500 mM in PBS pH 7.4, 5.00 µmol, 4.0 equiv). The solution was homogenized in a round-bottom flask for 2 min, after which ETP-DABCYL **15** (110 µL, 25 mM in DMSO, 2.75 µmol, 2.2 equiv) was added. The reaction mixture was stirred at room temperature for 90 min and subsequently quenched by addition of TFA (50 µL). The crude reaction mixture was diluted by addition of H_2O (0.1 % TFA), purified by semi-preparative HPLC (C-18; 5 → 60% MeCN, 0.1% TFA) and lyophilized to obtain EDANS aryl thiol ETP-DABCYL conjugate **16** (1.38 mg, 1.29 µmol, 52%) as a solid:

LC-MS (linear gradient 5 → 95% MeCN, 0.1% TFA, 15 min): R_t (min): 5.20 (ESI-MS (m/z): 537.61 ($M+2H^{2+}$));

HRMS: calculated for $C_{50}H_{63}N_{10}O_{11}PS_2$ 537.1923 $[M+2H]^{2+}$; found 537.1919;

HRMS: calculated for $C_{50}H_{62}N_{10}O_{11}PS_2$ 1073.3773 $[M+H]^+$; found 1073.3842.

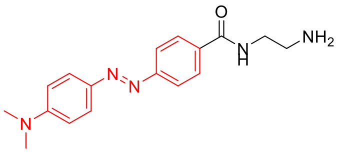

NH_2-DABCYL **21:**

DABCYL acid (99.5 mg, 369 µmol, 1.0 equiv) was dissolved in anhydrous DMF (1.0 mL). *N*-hydroxysuccinimide (47.8 mg, 415 µmol, 1.1 equiv) and EDC · HCl (105.1 mg, 548.3 µmol, 1.5 equiv) were added. The reaction mixture was stirred for 2 h at room temperature, after which formation of the intermediate NHS ester **20** was confirmed by LC-MS (linear gradient 5 → 95% MeCN, 0.1% TFA, 5 min): R_t (min): 2.56 (ESI-MS (m/z): 367.29 ($M+H^+$)). The crude, dark red reaction mixture was purified by silica gel chromatography (0 → 2% MeOH in DCM). The product fractions (**20**) were concentrated *in vacuo* before adding DIPEA (74 µL, 425 µmol, 1.15 equiv) and ethylenediamine (3.5 mL, 52 mmol, 140 equiv). The reaction mixture was stirred for 30 min at room temperature and purified by silica gel chromatography (10 → 20% MeOH in DCM) to obtain NH_2-DABCYL **21** (77.92 mg, 250 µmol, 68%) as a solid:

^{1}H NMR (600 MHz, MeOD) δ 8.00 – 7.93 (m, 2H), 7.89 – 7.81 (m, 4H), 6.87 – 6.80 (m, 2H), 3.50 (t, *J* = 6.3 Hz, 2H), 3.10 (s, 6H), 2.90 (t, *J* = 6.3 Hz, 2H);

^{13}C NMR (151 MHz, MeOD) δ 170.06, 156.48, 154.69, 144.87, 135.71, 129.33 (x2), 126.41 (x2), 122.95 (x2), 112.64 (x2), 43.19, 41.96, 40.37 (x2);

LC-MS (linear gradient 5 → 95% MeCN, 0.1% TFA, 15 min): R_t (min): 1.93 (ESI-MS (m/z): 312.11 ($M+H^+$), 334.12 ($M+Na^+$), 645.39 ($2M+Na^+$));

HRMS: calculated for $C_{17}H_{22}N_5O$ 312.1819 $[M+H]^+$; found 312.1812.

Spectroscopic data was in agreement with literature.[8]

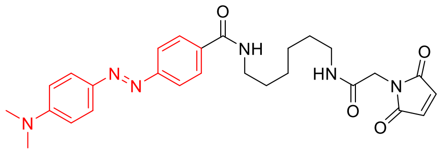

Maleimide-DABCYL **23:**

DABCYL acid (15 mg, 56 µmol, 1.0 equiv) and HATU (21 mg, 56 µmol, 1.0 equiv) were dissolved in DMF (200 µL). DIPEA (15 µL, 86 µmol, 1.5 equiv) and *N*-Boc-1,6-hexanediamine (12 mg, 56 µmol, 1.0 equiv) were added and the reaction was stirred for 30 min at room temperature. The reaction was quenched by addition of TFA (0.1% *v/v* in H_2O), purified by semi-preparative HPLC (C-18; 5 → 95% MeCN in H_2O, 0.1% TFA) and lyophilized to obtain Boc-NH-DABCYL intermediate **22** as a purple solid. Compound **22** was dissolved in DCM / TFA (1:1, 2 mL) and incubated for 15 min before the solvent was evaporated under a stream of N_2. The crude NH_2-DABCYL was suspended in DMF (200 µL), followed by the addition of maleimidoacetic acid *N*-hydroxysuccinimide ester (14 mg, 56 µmol, 1.0 equiv) and DIPEA (20 µL, 110 µmol, 2.1 equiv). The reaction was stirred for 1 hour at room temperature, quenched by addition of TFA (0.1% *v/v* in H_2O). The crude reaction mixture was purified by semi-preparative HPLC (C-18; 5 → 95% MeCN, 0.1% TFA) and lyophilized to obtain Maleimide-DABCYL **23** (3.3 mg, 6.5 µmol, 12% over three steps) as a purple solid:

^{1}H NMR (600 MHz, DMSO-d_6) δ 8.52 (t, *J* = 5.6 Hz, 1NH), 8.09 (t, *J* = 5.6 Hz, 1NH), 8.01 – 7.93 (m, 2H), 7.85 – 7.78 (m, 4H), 7.08 (s, 2H), 6.89 – 6.82 (m, 2H), 4.00 (s, 2H), 3.27 (q, *J* = 6.9 Hz, 3H), 3.08 (s, 6H), 3.05 (q, *J* = 7.0 Hz, 2H), 1.53 (p, *J* = 7.4 Hz, 2H), 1.41 (p, *J* = 7.0 Hz, 2H), 1.35 – 1.26 (m, 4H);

^{13}C NMR (151 MHz, DMSO-d_6) δ 170.67 (x2), 165.79, 165.47, 153.86, 152.82, 142.63, 134.97, 134.83 (x2), 128.25 (x2), 125.04 (x2), 121.45 (x2), 111.57 (x2), 40.06, 38.59, 29.05, 28.92, 26.15, 26.00;

LC-MS (linear gradient 5 → 95% MeCN, 0.1% TFA, 15 min): R_t (min): 6.16 (ESI-MS (m/z): 505.44 ($M+H^+$));

HRMS: calculated for $C_{27}H_{33}N_6O_4$ 505.2558 $[M+H]^+$; found 505.2590.

Note: several ^{13}C NMR signals (CH_3 x 2, CH_2 x 1) overlap with the solvent peak (δ 39.52 ppm). This is confirmed on the HSQC measurement.

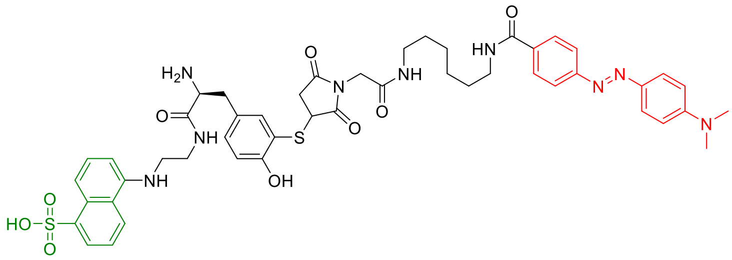

EDANS aryl thiol Maleimide-DABCYL conjugate **24:**

EDANS aryl disulfide **13** (100 µL, 6.25 mM in DMSO, 0.625 µmol, 1.0 equiv) was added to a mixture of PBS (pH 7.4, 1000 µL), MeCN (1000 µL) and TCEP (50.0 µL, 50 mM in PBS pH 7.4, 2.50 µmol, 4.0 equiv). The solution was homogenized in a round-bottom flask for 2 min, after which Maleimide-DABCYL **23** (131 µL, 10 mM in DMSO, 1.31 µmol, 2.1 equiv) was added and the reaction mixture was stirred for 5 min at room temperature. The reaction was subsequently quenched by addition of TFA (50 µL), diluted by addition of H_2O/MeCN (0.1 % TFA), purified by semi-preparative HPLC (C-18; 5 → 95% MeCN, 0.1% TFA) and lyophilized to obtain EDANS aryl thiol Maleimide-DABCYL conjugate **24** (0.5 mg, 0.5 µmol, 40%) as a solid:

LC-MS (linear gradient 5 → 95% MeCN, 0.1% TFA, 15 min): R_t (min): 5.54 (ESI-MS (m/z): 483.92 ($M+2H^{2+}$), 966.57 ($M+H^+$));

HRMS: calculated for $C_{48}H_{57}N_9O_9S_2$ 483.6855 $[M+2H]^{2+}$; found 483.6817;

HRMS: calculated for $C_{48}H_{56}N_9O_9S_2$ 966.3637 $[M+H]^+$; found 966.3721.

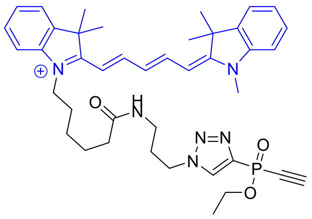

ETP-Cy5 **29:**

Cy5 azide, chloride salt (Lumiprobe, 10 mg, 17 µmol, 1.0 equiv) was dissolved in a mixture of DMSO (200 µL), MeCN (200 µL) and 0.2 M phosphate buffer pH 6.0 (400 µL). Ethyl diethynyl phosphinate[1,2] (24.0 mg, 170 µmol, 10 equiv) was added. MeCN (200 µL) was added to dry CuBr (≥ 3 mg) to obtain a green, saturated solution of CuBr. THPTA (tris((1-benzyl-4-triazolyl)methyl)amine; 30 µL, 100 mM in PBS pH 7.4, 3.0 µmol, 18 mol%) was added, and the resulting colorless solution was added to the reaction mixture to initiate CuAAC. The reaction mixture was stirred at room temperature for 15 min and subsequently quenched by addition of TFA (50 µL). The crude reaction mixture was diluted by addition of H_2O/MeCN (0.1 % TFA), purified by semi-preparative HPLC (C-18; 5 → 95% MeCN, 0.1% TFA) and lyophilized to obtain ETP-Cy5 **29** (8.84 mg, 11.9 µmol, 72%) as a solid:

LC-MS (linear gradient 5 → 95% MeCN, 0.1% TFA, 15 min): R_t (min): 7.30 (ESI-MS (m/z): 707.36 ($M+H^+$));

HRMS: calculated for $C_{41}H_{52}N_6O_3P$ 707.3833 $[M]^+$; found 707.3908.

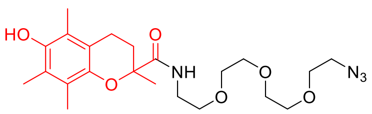

Trolox azide **30:**

Trolox (6-hydroxy-2,5,7,8-tetramethylchroman-2-carboxylic acid; 15 mg, 60 µmol, 1.0 equiv) and PyBOP (62 mg, 0.12 mmol, 2.0 equiv) were dissolved in DMSO (600 µL) followed by the addition of anhydrous DIPEA (31 µL, 0.18 mmol, 3.0 equiv). The reaction mixture was stirred for 5 min at room temperature before adding 11-azido-3,6,9-trioxaundecan-1-amine (24 µL, 26 mg, 0.12 µmol, 2.0 equiv), after which the reaction was allowed to proceed overnight at room temperature. The crude reaction mixture was diluted by addition of H_2O/MeCN (0.1 % TFA), purified by semi-preparative HPLC (C-18; 5 → 95% MeCN, 0.1% TFA) and lyophilized to obtain trolox azide **30** (23.4 mg, 51.9 µmol, 87%) as a colorless oil:

^{1}H NMR (600 MHz, DMSO-d_6) δ 7.49 (s, 1OH), 7.18 (t, J = 5.8 Hz, 1NH), 3.57 (t, J = 4.7 Hz, 2H), 3.55 – 3.51 (m, 2H), 3.51 – 3.48 (m, 2H), 3.46 – 3.42 (m, 2H), 3.42 – 3.28 (m, 6H), 3.22 (q, J = 5.7 Hz, 2H), 2.57 – 2.51 (m, 1H), 2.46 – 2.37 (m, 1H), 2.19 – 2.12 (m, 1H), 2.09 (s, 3H), 2.07 (s, 3H), 1.99 (s, 3H), 1.37 (s, 3H);

^{13}C NMR (151 MHz, DMSO-d_6) δ 173.30, 145.88, 143.70, 122.65, 120.99, 120.24, 117.05, 77.24, 69.76 (x2), 69.63, 69.58, 69.22, 68.79, 49.96, 38.41, 29.35, 24.07, 19.98, 12.70, 11.90, 11.75;

LC-MS (linear gradient 5 → 95% MeCN, 0.1% TFA, 15 min): R_t (min): 6.32 (ESI-MS (m/z): 451.09 ($M+H^+$), 473.07 ($M+Na^+$));

HRMS: calculated for $C_{22}H_{35}N_4O_6$ 451.2551 $[M+H]^+$; found 451.2562;

HRMS: calculated for $C_{22}H_{34}N_4NaO_6$ 473.2371 $[M+Na]^+$; found 473.2329.

Note: one ^{1}H NMR signal overlaps with a pyrrolidine impurity signal (δ 1.73 ppm). This is confirmed on the HSQC measurement.

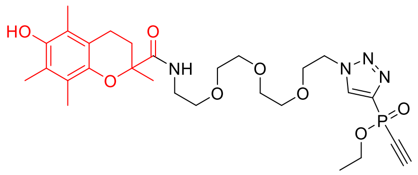

ETP-Trolox **31:**

Trolox azide **30** (300 µL, 75 mM in DMSO, 22.5 µmol, 1.0 equiv) was dissolved in a mixture of MeCN (200 µL) and 0.2 M phosphate buffer pH 6.0 (400 µL). Ethyl diethynyl phosphinate[1,2] (32 mg, 225 µmol, 10 equiv) was added. MeCN (200 µL) was added to dry CuBr (≥ 3 mg) to obtain a green, saturated solution of CuBr. THPTA (tris((1-benzyl-4-triazolyl)methyl)amine; 30 µL, 100 mM in PBS pH 7.4, 3.0 µmol, 13 mol%) was added, and the resulting colorless solution was added to the reaction mixture to initiate CuAAC. The reaction mixture was stirred at room temperature for 15 min and subsequently quenched by addition of TFA (50 µL). The crude reaction mixture was diluted by addition of H_2O/MeCN (0.1% TFA), purified by semi-preparative HPLC (C-18; 5 → 95% MeCN, 0.1% TFA) and lyophilized to obtain ETP-Trolox **31** (5.48 mg, 9.25 µmol, 41%) as a solid:

^{1}H NMR (600 MHz, DMSO-d$_6$) δ 8.65 (s, 1H), 7.48 (s, 1OH), 7.19 (t, *J* = 5.8 Hz, 1NH), 4.70 (d, *J* = 11.6 Hz, 1H), 4.61 (t, *J* = 5.3 Hz, 2H), 4.21 – 4.09 (m, 2H), 3.84 (t, *J* = 5.3 Hz, 2H), 3.52 – 3.28 (m, 10H), 3.21 (q, *J* = 5.6 Hz, 2H), 2.56 – 2.51 (m, 1H), 2.45 – 2.36 (m, 1H), 2.15 (dt, *J* = 12.5, 6.1 Hz, 1H), 2.08 (s, 3H), 2.07 (s, 3H), 1.99 (s, 3H), 1.73 (ddd, *J* = 14.2, 8.5, 6.0 Hz, 1H), 1.36 (s, 3H), 1.30 (t, *J* = 7.0 Hz, 3H);

^{13}C NMR (151 MHz, DMSO-d$_6$) δ 173.31, 145.87, 143.70, 138.56 (d, *J* = 213.6 Hz), 131.85 (d, *J* = 34.0 Hz), 122.65, 121.01, 120.23, 117.05, 94.48 (d, *J* = 39.5 Hz), 77.24 (d, *J* = 220.6 Hz), 77.23, 69.67, 69.54 (x2), 69.42, 68.77, 68.20, 62.58 (d, *J* = 6.2 Hz), 49.57, 38.40, 29.36, 24.07, 19.98, 16.01 (d, *J* = 6.9 Hz), 12.72, 11.91, 11.76;

^{31}P NMR (243 MHz, DMSO-d$_6$) δ -4.80;

LC-MS (linear gradient 5 → 95% MeCN, 0.1% TFA, 15 min): R_t (min): 5.84 (ESI-MS (m/z): 593.21 (M+H$^+$) 615.22 (M+Na$^+$));

HRMS: calculated for $C_{28}H_{42}N_4O_8P$ 593.2735 [M+H]$^+$; found 593.2733;

HRMS: calculated for $C_{28}H_{41}N_4NaO_8P$ 615.2554 [M+Na]$^+$; found 615.2581.

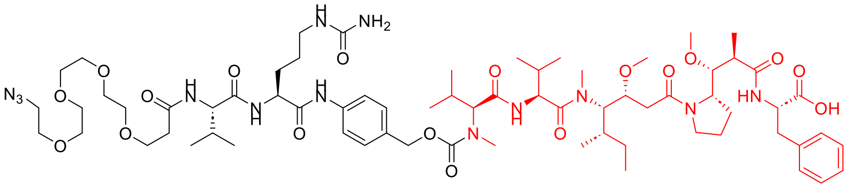

Azido-PEG$_3$-Val-Cit-PAB-MMAF **35:**

A solution of Val-Cit-PAB-MMAF sodium salt (MedChemExpress; 172 µL, 50 mM in DMSO, 8.6 µmol, 1.0 equiv) was transferred to a 5 mL round bottom flask. *N*-Succinimidyl 15-azido-4,7,10,13-tetraoxapentadecanoate (20 µL, 1.0 M in DMSO, 20 µmol, 2.3 equiv) and DIPEA (4.5 µL, 26 µmol, 3.0 equiv) were added. The reaction mixture was stirred at room temperature for 2 h and subsequently diluted by addition of H_2O/MeCN (0.1 % TFA), purified by semi-preparative HPLC (C-18; 5 → 95% MeCN, 0.1% TFA) and lyophilized to obtain azido-PEG$_3$-Val-Cit-PAB-MMAF **35** (7.24 mg, 5.13 µmol, 60%) as a solid:

LC-MS (linear gradient 5 → 95% MeCN, 0.1% TFA, 15 min): R_t (min): 7.85 (ESI-MS (m/z): 706.16 (M+2H^{2+}));

HRMS: calculated for $C_{69}H_{113}N_{13}O_{18}$ 705.9158 [M+2H]$^{2+}$; found 705.9138;

HRMS: calculated for $C_{69}H_{112}N_{13}O_{18}$ 1410.8243 [M+H]$^+$; found 1410.8376.

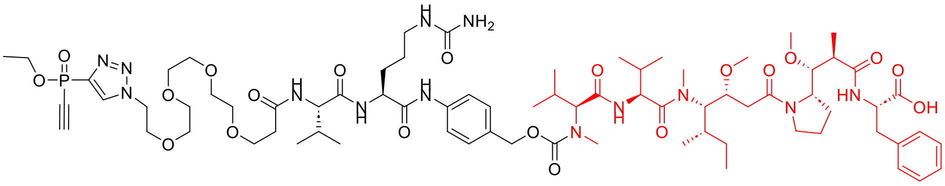

ETP-PEG$_3$-Val-Cit-PAB-MMAF **36:**

Azido-PEG$_3$-Val-Cit-PAB-MMAF **35** (205 µL, 25 mM in DMSO, 5.13 µmol, 1.0 equiv) was dissolved in a mixture of MeCN (100 µL) and 0.2 M phosphate buffer pH 6.0 (400 µL). Ethyl diethynyl phosphinate[1,2] (10.9 mg, 77.0 µmol, 15 equiv) was added. MeCN (200 µL) was added to dry CuBr (≥ 3 mg) to obtain a green, saturated solution of CuBr. THPTA (tris((1-benzyl-4-triazolyl)methyl)amine; 30 µL, 100 mM in PBS pH 7.4, 3.0 µmol, 58 mol%) was added, and the resulting colorless solution was added to the reaction mixture to initiate CuAAC. The reaction mixture was stirred at room temperature for 15 min and subsequently quenched by addition of 1% TFA (1 mL). The crude reaction mixture was diluted by addition of H_2O/MeCN (0.1 % TFA), purified by semi-preparative HPLC (C-18; 5 → 95% MeCN, 0.1% TFA) and lyophilized to obtain ETP-PEG$_3$-Val-Cit-PAB-MMAF **36** (5.0 mg, 3.2 µmol, 63%) as a solid:

LC-MS (linear gradient 5 → 95% MeCN, 0.1% TFA, 15 min): R_t (min): 7.53 (ESI-MS (m/z): 777.14 (M+2H^{2+}));

HRMS: calculated for $C_{75}H_{120}N_{13}O_{20}P$ 776.9250 [M+2H]$^{2+}$; found 776.9191;

HRMS: calculated for $C_{75}H_{119}N_{13}O_{20}P$ 1552.8427 [M+H]$^+$; found 1552.7515.

6. Experimental Procedures – Physical Organic Chemistry

6.1 2-D NMR assay for pK_a determination

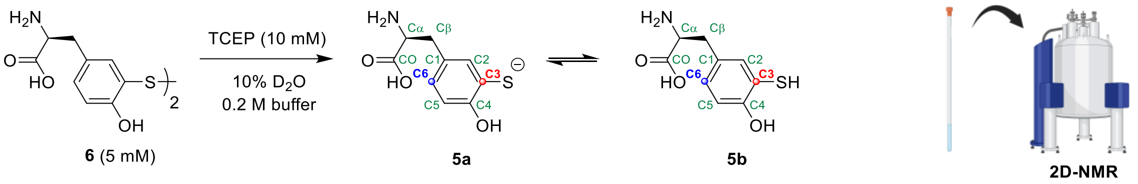

Sample preparation

Aryl disulfide **6** (30 µL, 50 mM in D_2O, 1.5 µmol, 1.0 equiv) and TCEP · HCl (6 µL, 500 mM in H_2O - pH 7.0, 3.0 µmol, 2.0 equiv; solution in sealed vial; Sigma Aldrich 646547-10X1ML) were added to an aqueous solution (264 µL). The sample was carefully mixed and transferred to a 3.0 mm NMR tube for experimentation.

Solutions used:*
pH 1.0 - 1% (w/w) HCl in H_2O
pH 4.5 – 0.2 M citrate buffer (citric acid monohydrate / tri-sodium citrate dihydrate)
pH 5.0 – 0.2 M citrate buffer
pH 5.5 – 0.2 M citrate buffer
pH 6.0 – 0.2 M phosphate buffer (NaH_2PO_4 / Na_2HPO_4)
pH 6.5 – 0.2 M phosphate buffer
pH 7.0 – 0.2 M phosphate buffer
pH 8.0 – 0.2 M phosphate buffer
pH 13.0 – 1% (w/w) NaOH in H_2O

*Solutions were prepared in 250 mL volume; pH values for all buffered solutions were measured using a pH-meter and adjusted by addition of aq. HCl or NaOH as required.

Sample measurements

NMR spectra were recorded at 300 K at 600 MHz (^{1}H frequency) on Bruker AV-III spectrometers using a cryogenically cooled TCI probe equipped with one-axis self-shielded gradients. The software used to control the spectrometer was topspin 3.5 pl6. For each sample the same set of two one-dimensional and two two-dimensional spectra was recorded. One-dimensional ^{1}H-spectra with presaturation ofthe water resonance were recorded using 16k complex points, an acquisition time of 1.6 sec and 8 scans. A second type of one-dimensional ^{1}H-spectrum was recorded utilizing a WATERGATE watersupression[9] and using the same parameters (except the number of scans was 32). The two two-dimensional spectra that were recorded to obtain the chemical shifts of the carbon resonances were an HMQC[10,11] and an HMBC,[12,13] both were performed in there gradient enhanced version with additional presaturation to minimize the water resonance. The HMQCs were recorded using 8 scans to obtain 512 x 128 complex points with acquisition times of 51.2 msec and 5.1 msec in F2 and F1 respectively. The HMBCs were recorded with 48 scans, using 2048 x 150 complex point and acquisition times of 204.8 and 4.8. msec in F2 and F1 respectively.

Data were processed using topspin3.5pl6, typically a squared sine bell shifted by 90° was used in both dimensions, in case of the HMBC a unshifted sine bell was used in F2, a magnitude calculation in F2

follow the 2D-FT. Datasets were processed to yield a data matrix of 4096 by 2048 points. The spectra were transferred to the program NMRFAM-SPARKY[14] for assignment.

Determination of pK$_a$ values

Upon recording HMQC and HMBC for all 9 samples (pH values 1.0, 4.5, 5.0, 5.5, 6.0, 6.5, 7.0, 8.0, 13.0), the two spectra were used to confirm the assignment for each pH value and to extract the chemical shifts of selected resonances. To ease the fitting the difference between the chemical shift of each spectrum and the spectrum at pH = 1.0 was calculated. In the HMQC the chemical shifts of C6 (the carbon opposite the sulfur) was used, in the HMBC the chemical shift of C3 (the carbon to which the sulfur is attached; the 2D intersection of H5 -> C3).

To determine the pK_a value, an equation derived from the Henderson-Hasselbach-Equation was fitted to the graph of the chemical shift difference ($d_{exp} – d_{HCl}$) plotted against the pH value:

$$(\delta_{exp} - \delta_{HCl}) = C + \frac{(\delta_{NaOH} - \delta_{HCl}) * 10^{(pH - pKa)}}{1 + 10^{(pH - pKa)}}$$

To perform the fit, the program Kaleidagraph for Windows (Synergy Software, Reading, PA, USA) was used, that utilizes a Levenberg-Marquardt algorithm for its general curve fits. The resulting pK_a values were in good agreement: 5.50 for the peaks of C6 in the HMQC and 5.48 for the peaks of C3 in the HMBC.

Reference measurement in PBS pH 7.4 (10% D_2O) – full spectra (Mnova)

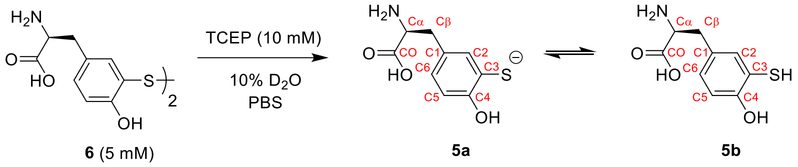

Pre-saturation (H_2O): ^{1}H NMR (600 MHz, H_2O+D_2O) δ 7.17 (s, 1H), 6.81 (d, *J* = 8.2 Hz, 1H), 6.77 (d, *J* = 8.1 Hz, 1H), 3.84 (dd, *J* = 8.5, 4.8 Hz, 1H), 3.07 (dd, *J* = 14.6, 4.9 Hz, 1H), 2.87 (dd, *J* = 14.6, 8.2 Hz, 1H).

Watergate: ^{1}H NMR (600 MHz, H_2O+D_2O) δ 7.17 (d, *J* = 2.1 Hz, 1H), 6.81 (dd, *J* = 8.2, 2.2 Hz, 1H), 6.77 (d, *J* = 8.1 Hz, 1H), 3.88 – 3.80 (m, 1H),* 3.07 (dd, *J* = 14.8, 5.0 Hz, 1H), 2.86 (dd, *J* = 14.6, 8.2 Hz, 1H).

*Note: the signal at 3.88 – 3.80 was reduced in intensity due to the Watergate effect.

^{13}C NMR (151 MHz, H_2O+D_2O) δ 174.13, 152.69, 132.58, 127.21, 126.01 (x2), 113.99, 56.28, 35.60.

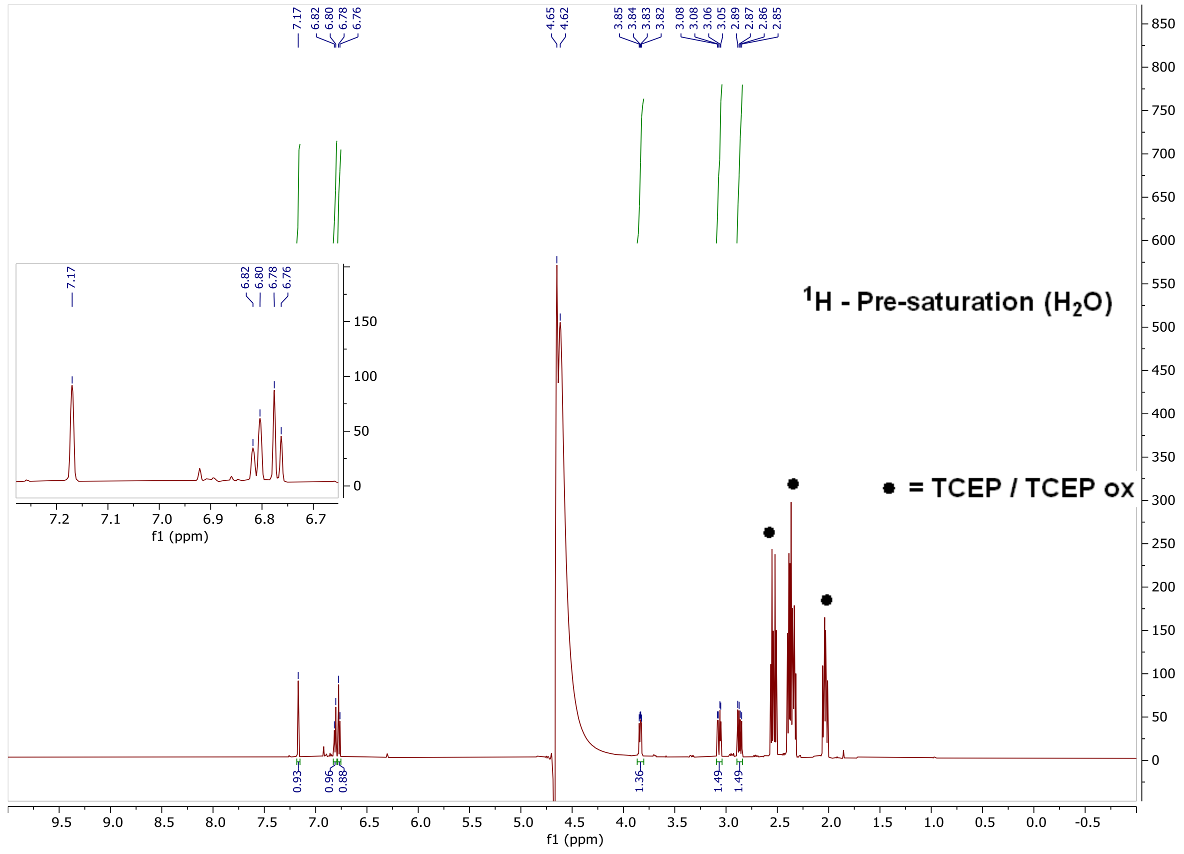

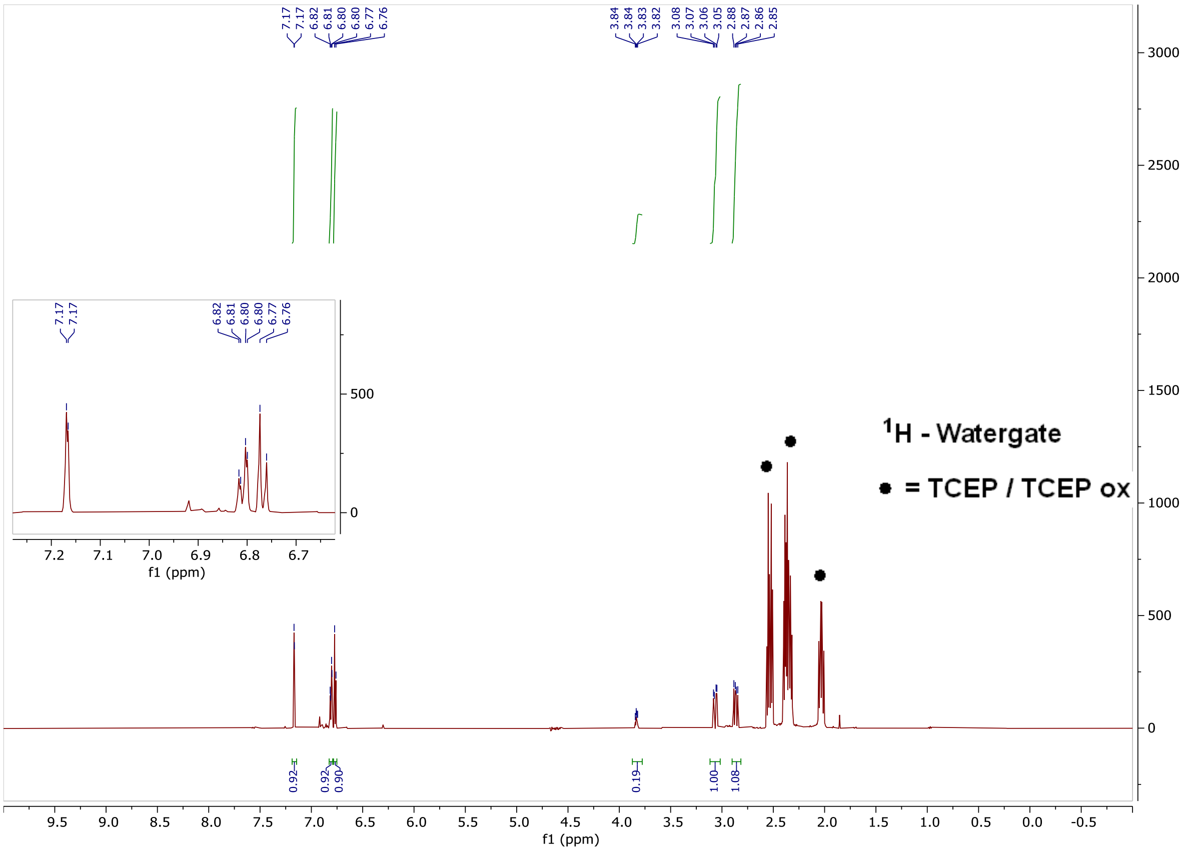

1H - Watergate
● = TCEP / TCEP ox
f1 (ppm)

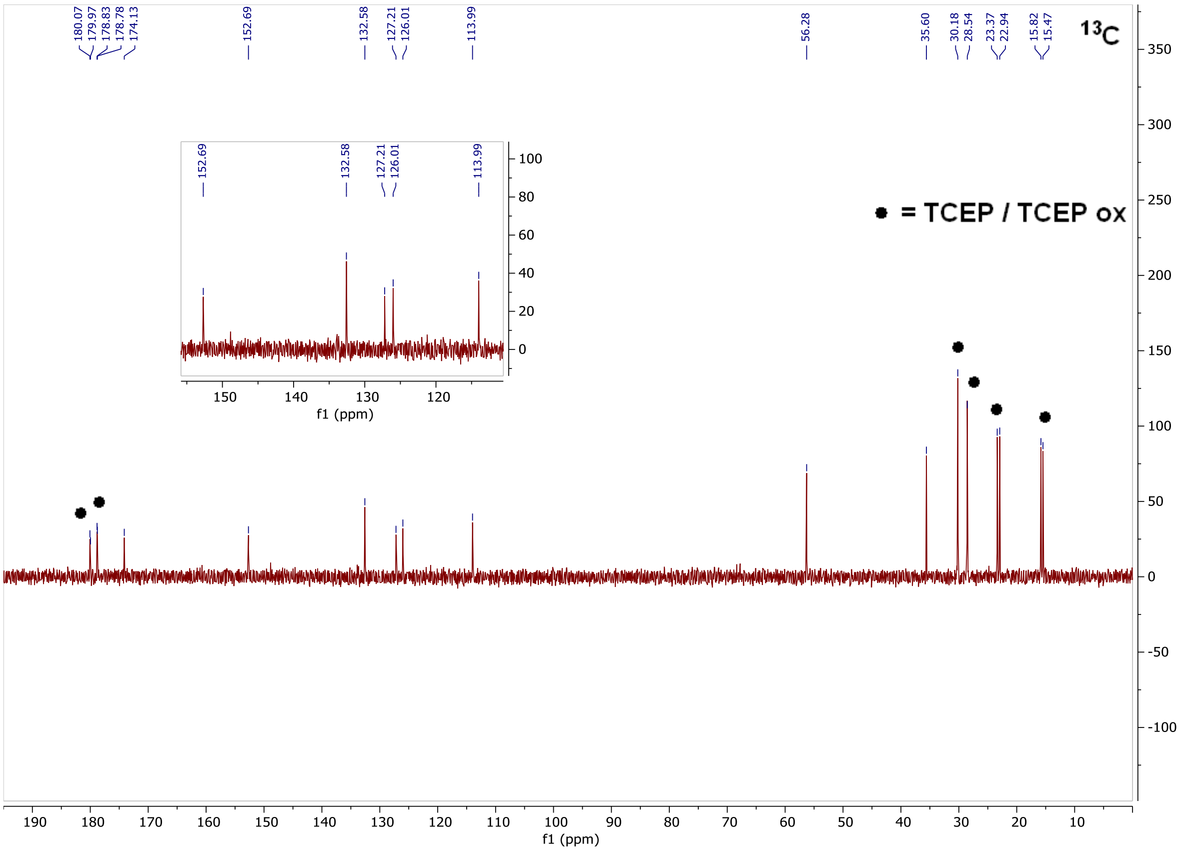

13C
● = TCEP / TCEP ox
f1 (ppm)

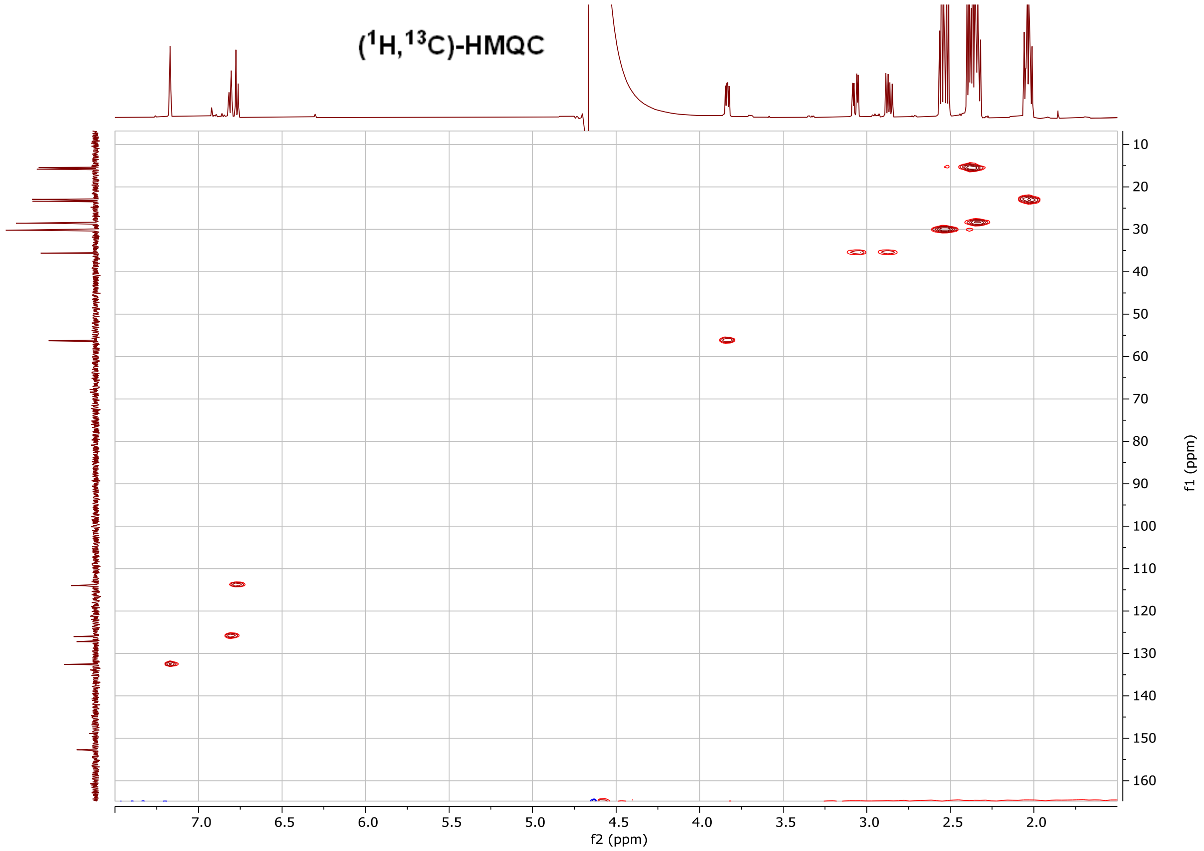
(^{1}H,^{13}C)-HMQC
f1 (ppm)
f2 (ppm)

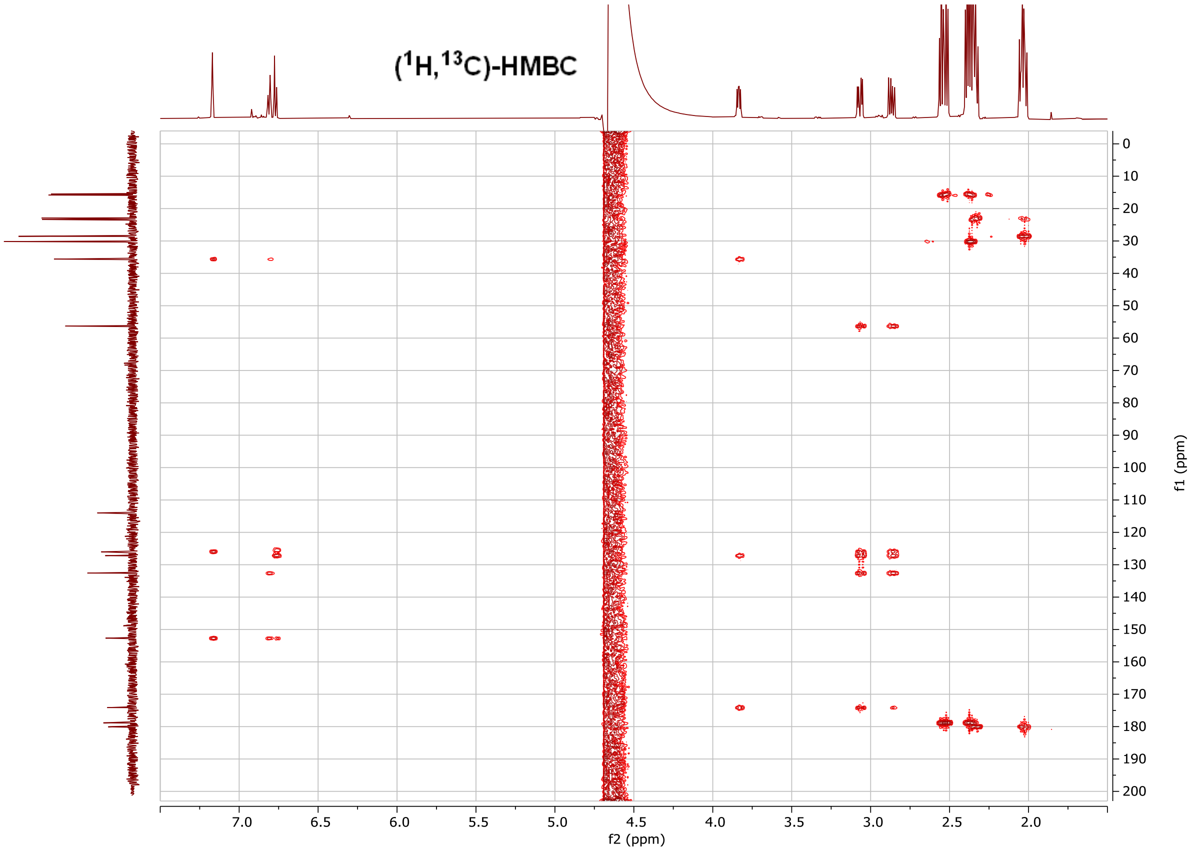
(^{1}H,^{13}C)-HMBC
f1 (ppm)
f2 (ppm)

Reference measurement in PBS pH 7.4 (10% D₂O) – full assignment (1/2)

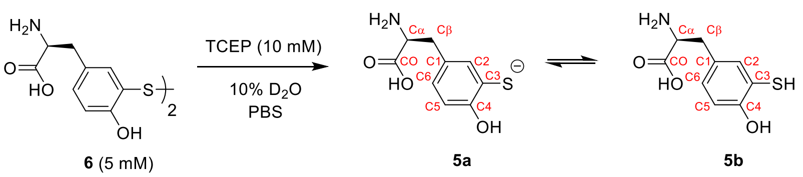

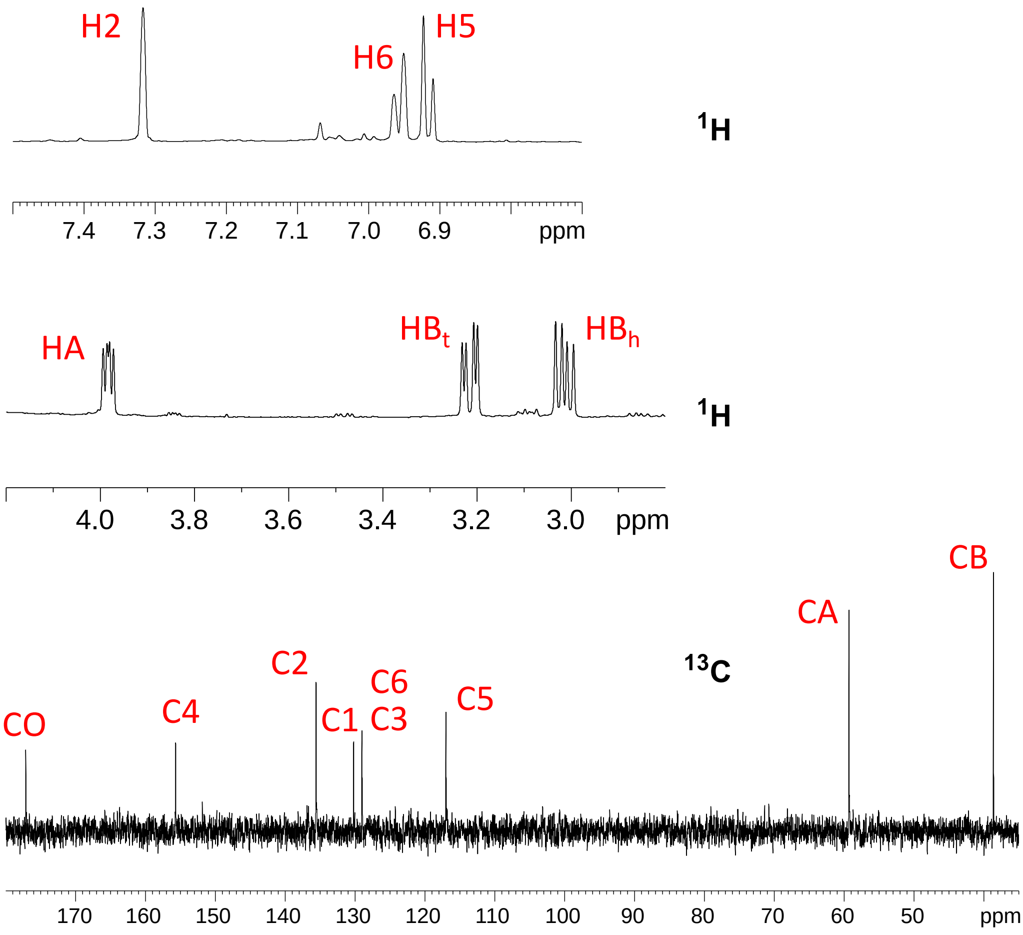

Reference measurement for 6 in PBS pH 7.4 (10% D_2O) – full assignment (2/2)

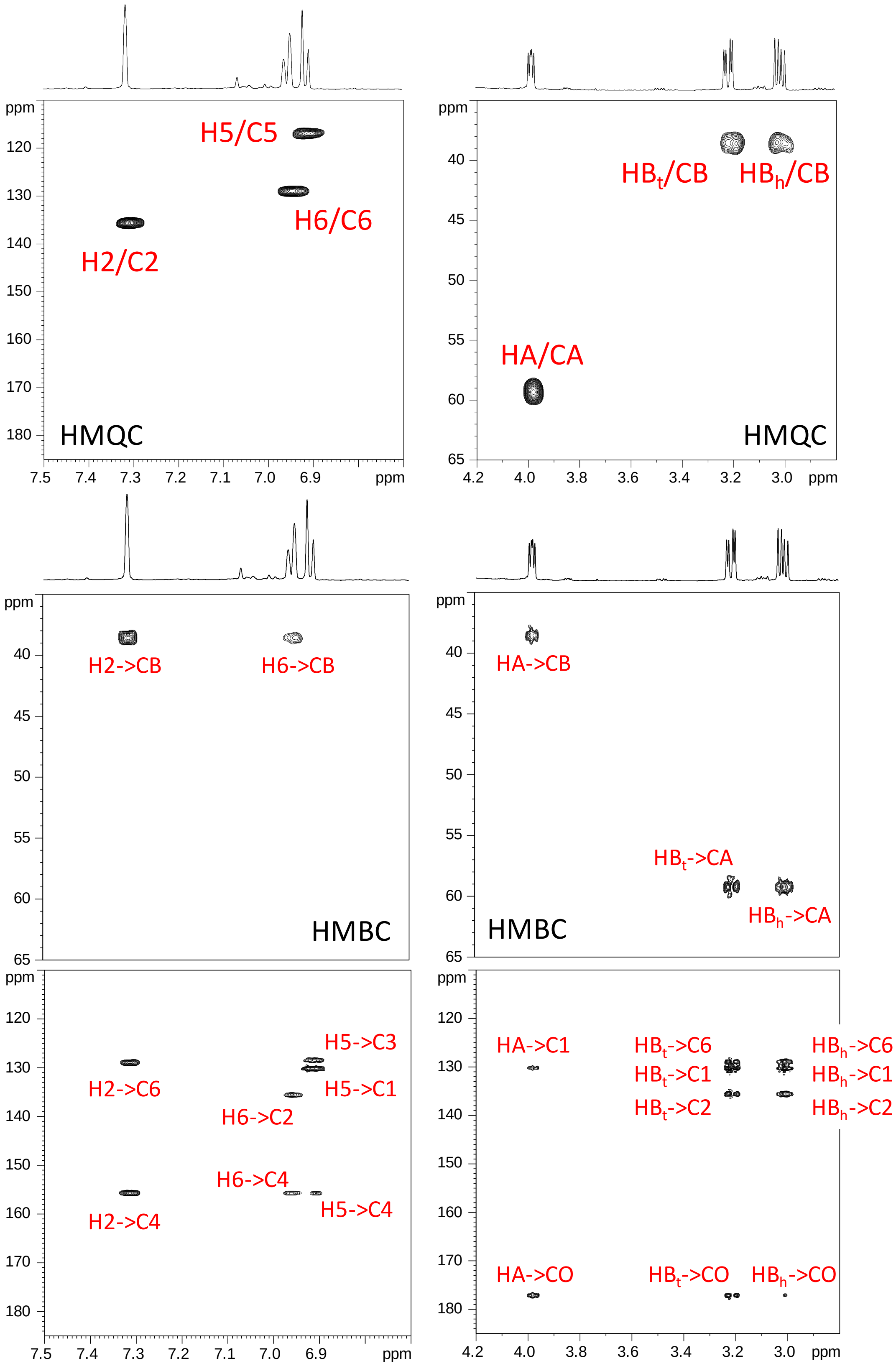

HMBC / HMQC overlay assignments per pH value (x9)

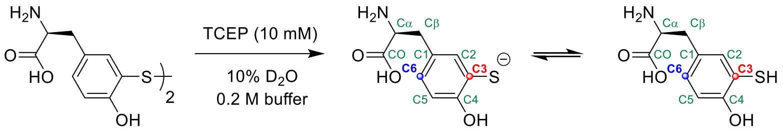

pH = 1.0

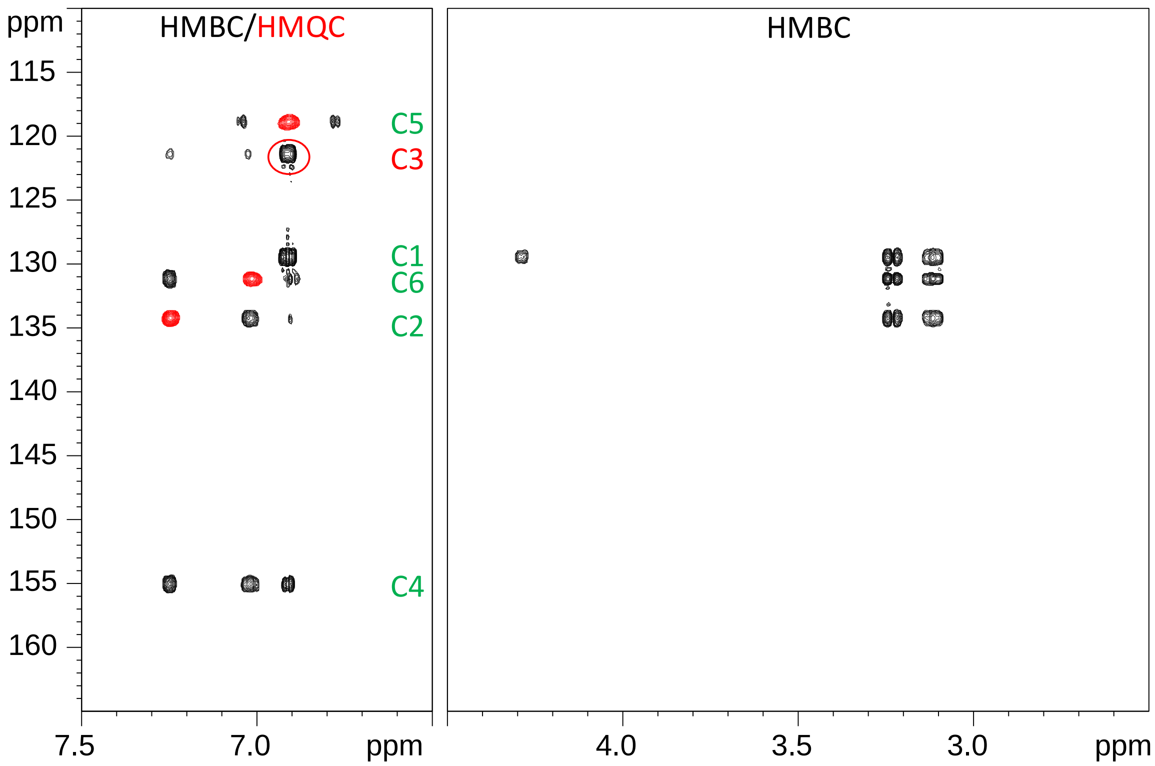

pH 1.0: HMBC/HMQC overlay for the measurement of **6** (5 mM) upon treatment with TCEP (10 mM) in 1% HCl (pH 1.0) containing 10% D_2O. The HMBC signal for C3 was encircled.

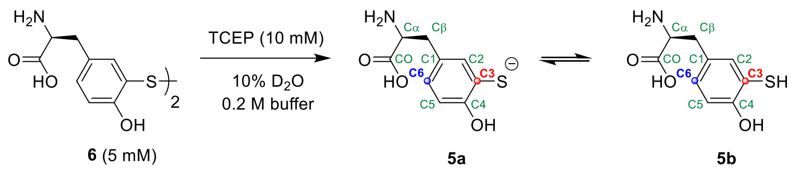

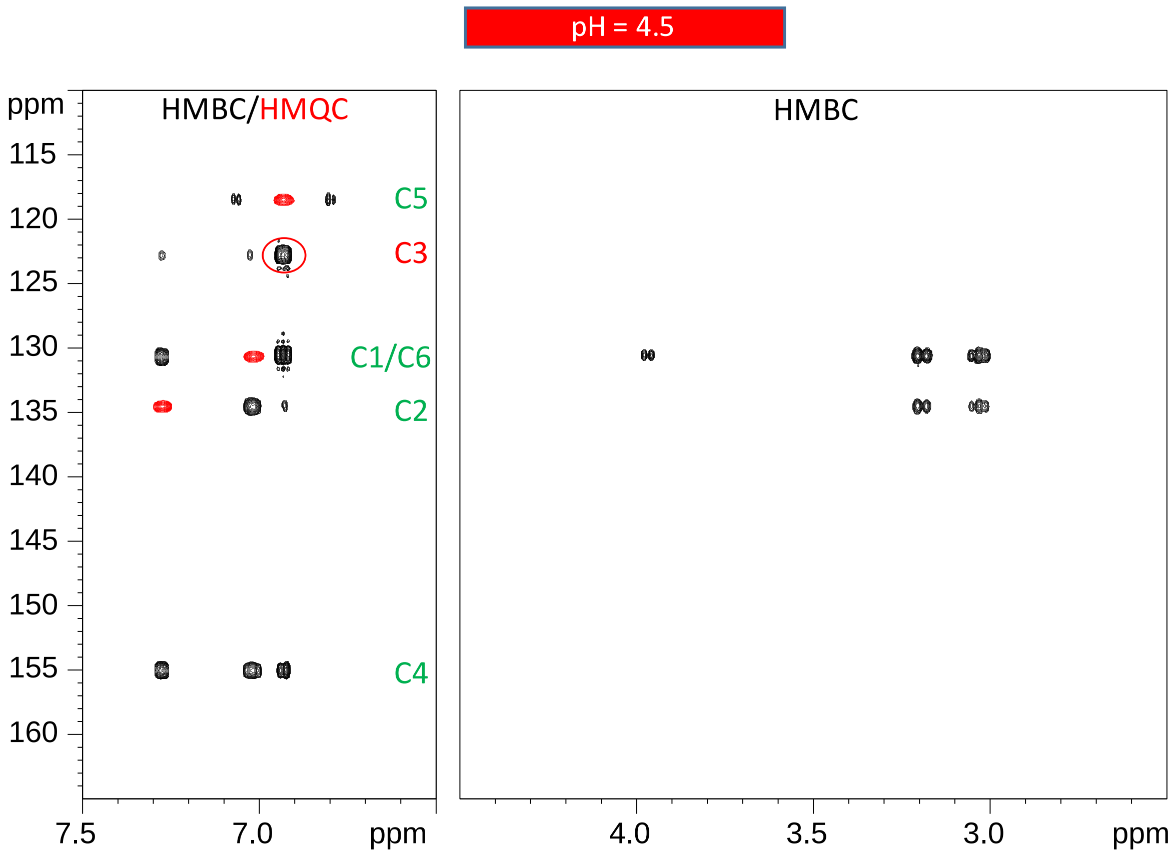

pH 4.5: HMBC/HMQC overlay for the measurement of **6** (5 mM) upon treatment with TCEP (10 mM) in 0.2 M citrate buffer (pH 4.5) containing 10% D_2O. The HMBC signal for C3 was encircled.

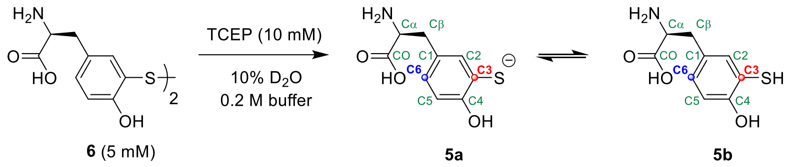

pH = 5.0

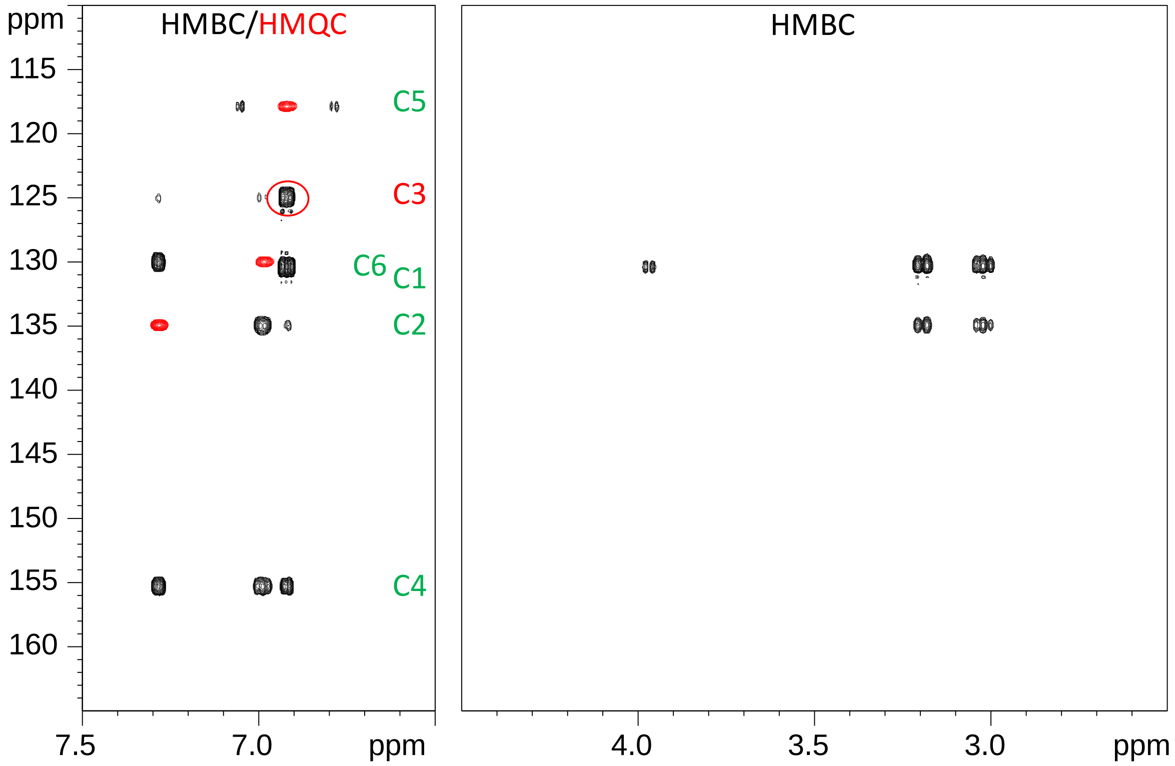

pH 5.0: HMBC/HMQC overlay for the measurement of **6** (5 mM) upon treatment with TCEP (10 mM) in 0.2 M citrate buffer (pH 5.0) containing 10% D_2O. The HMBC signal for C3 was encircled.

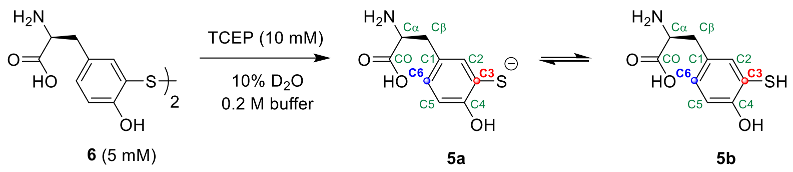

pH = 5.5

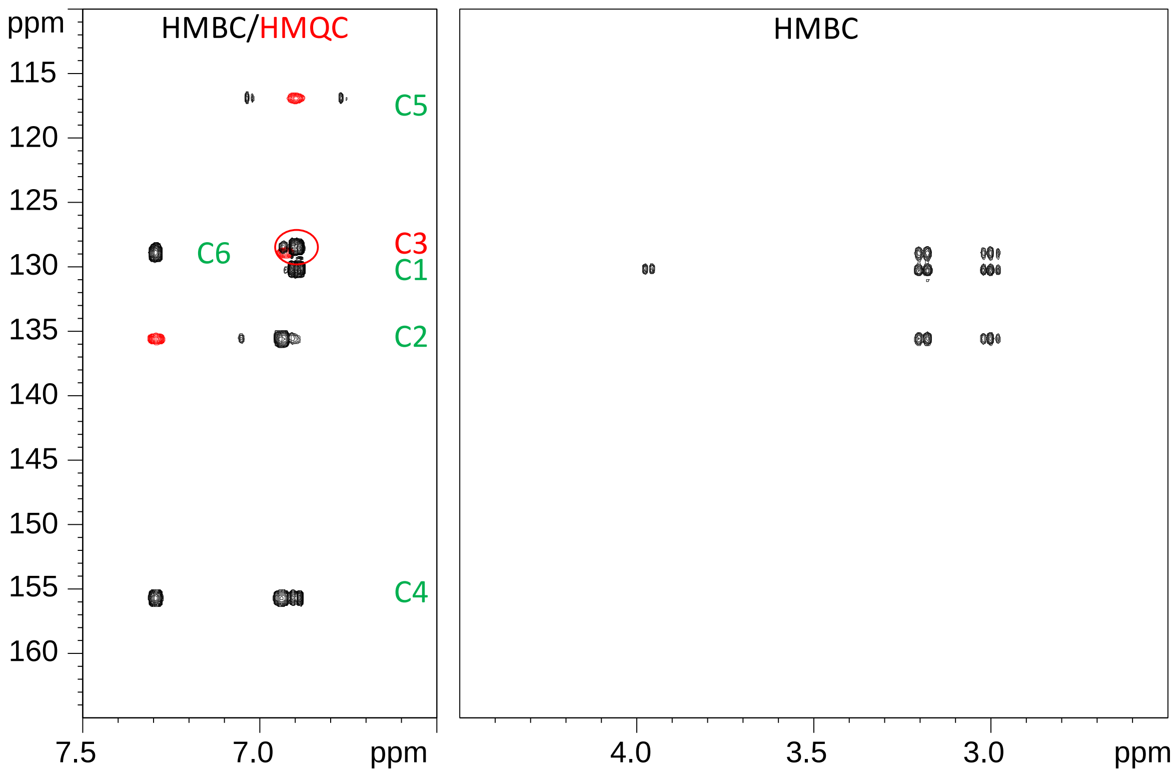

pH 5.5: HMBC/HMQC overlay for the measurement of **6** (5 mM) upon treatment with TCEP (10 mM) in 0.2 M citrate buffer (pH 5.5) containing 10% D_2O. The HMBC signal for C3 was encircled.

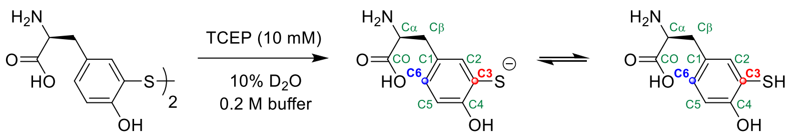

6 (5 mM) **5a** **5b**

pH = 6.0

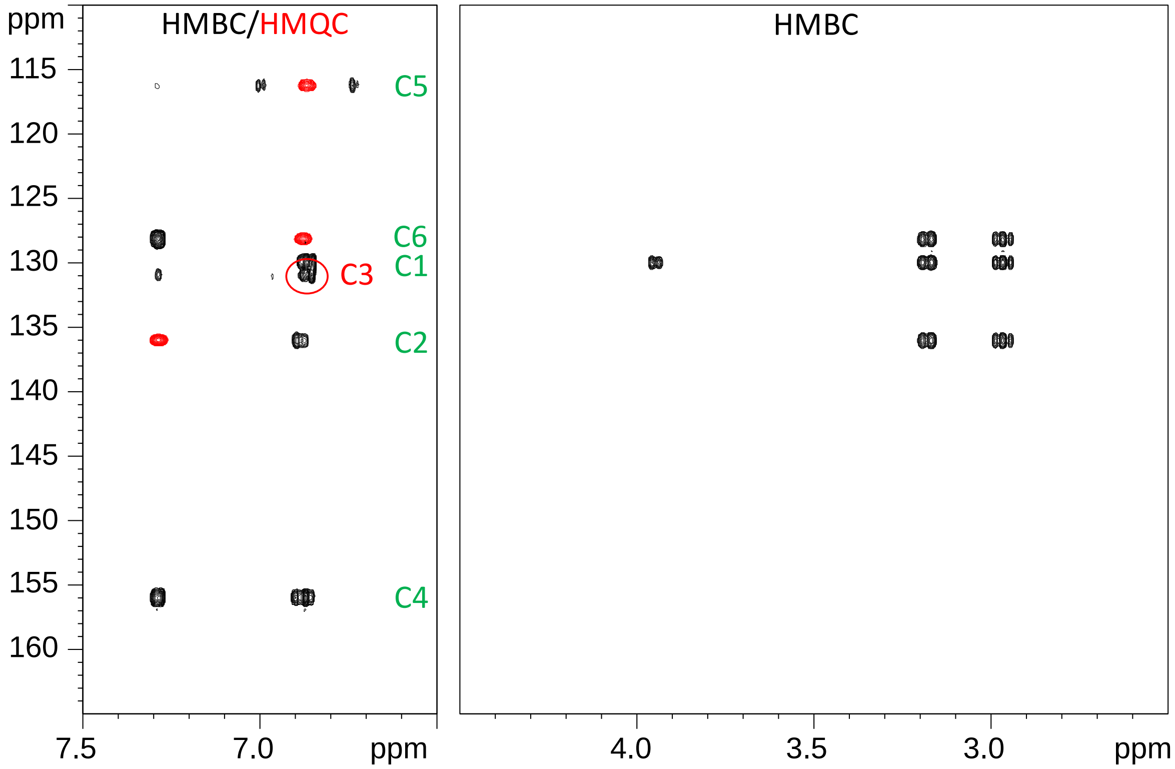

pH 6.0: HMBC/HMQC overlay for the measurement of **6** (5 mM) upon treatment with TCEP (10 mM) in 0.2 M phosphate buffer (pH 6.0) containing 10% D_2O. The HMBC signal for C3 was encircled.

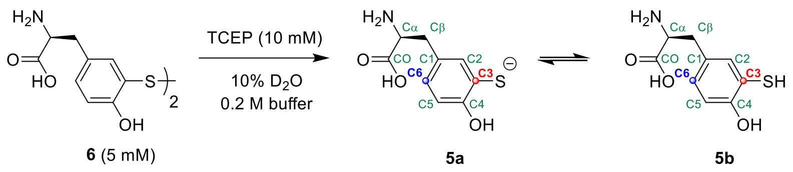

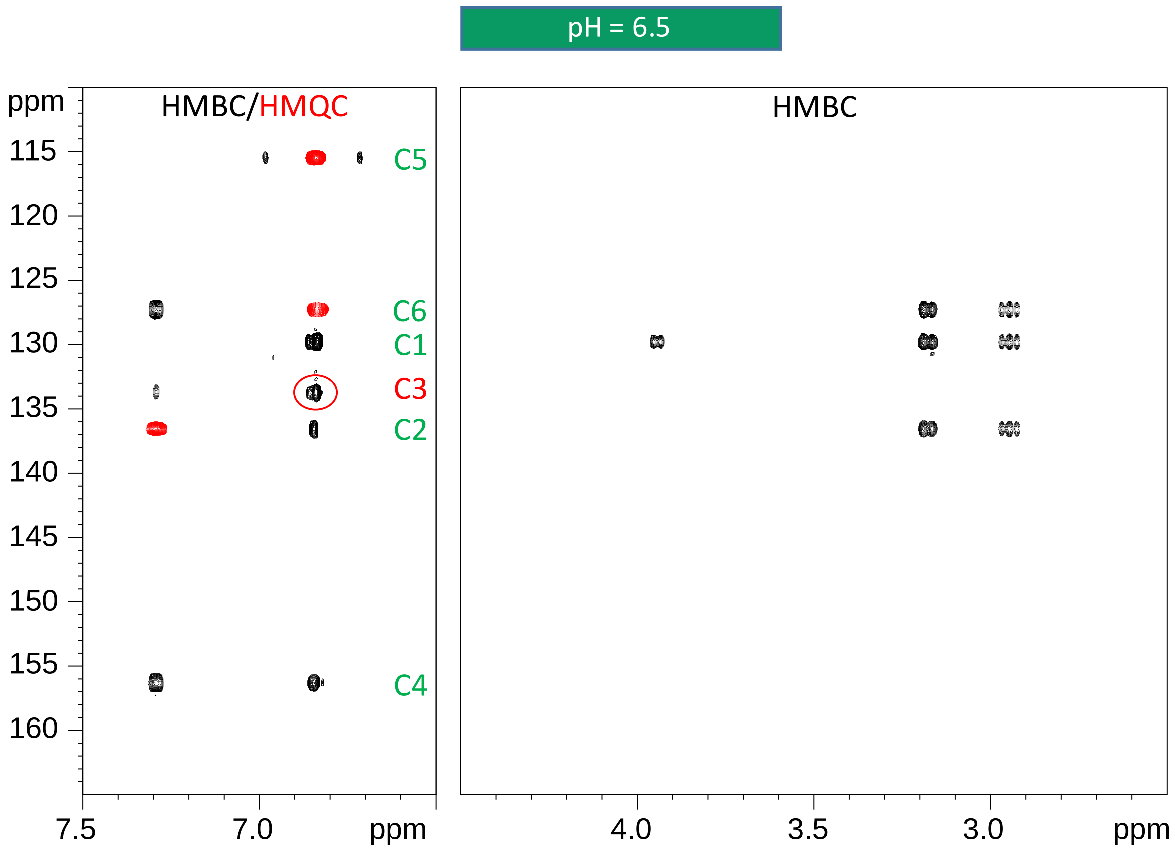

pH 6.5: HMBC/HMQC overlay for the measurement of **6** (5 mM) upon treatment with TCEP (10 mM) in 0.2 M phosphate buffer (pH 6.5) containing 10% D_2O. The HMBC signal for C3 was encircled.

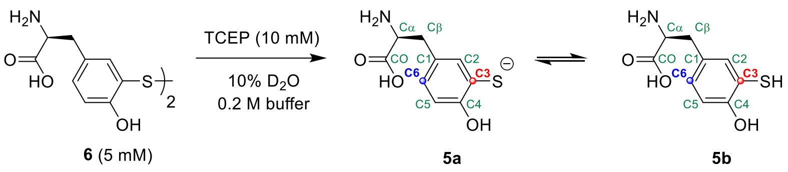

pH = 7.0

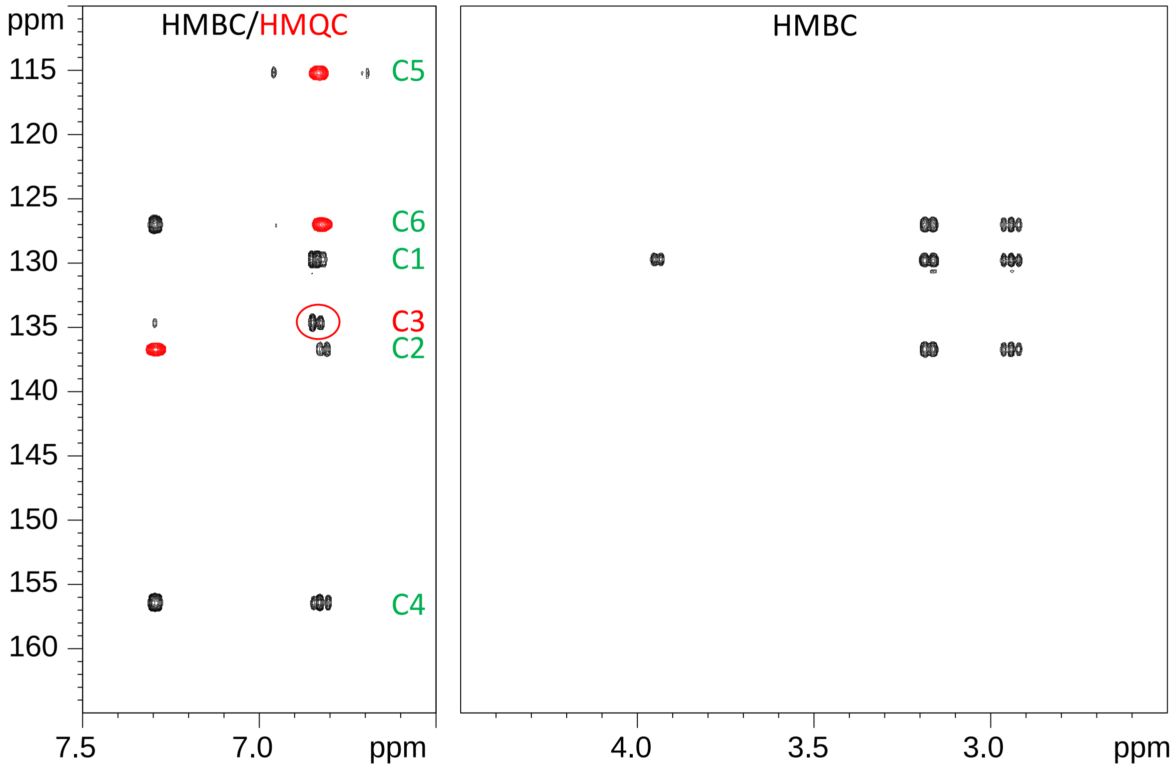

pH 7.0: HMBC/HMQC overlay for the measurement of **6** (5 mM) upon treatment with TCEP (10 mM) in 0.2 M phosphate buffer (pH 7.0) containing 10% D_2O. The HMBC signal for C3 was encircled.

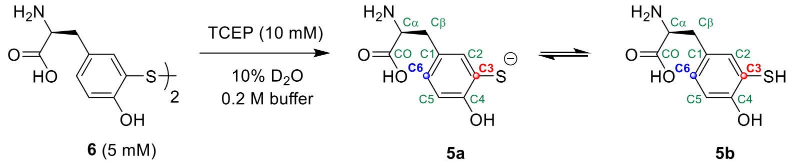

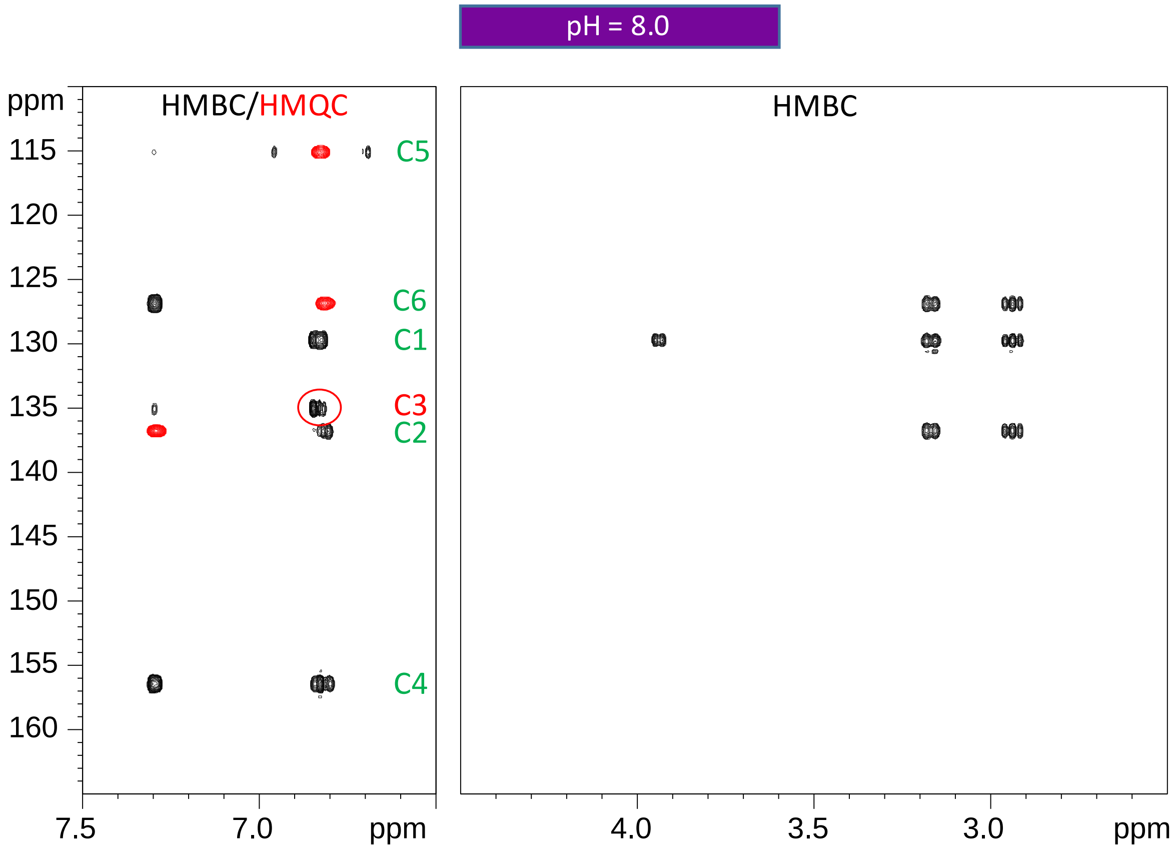

pH 8.0: HMBC/HMQC overlay for the measurement of **6** (5 mM) upon treatment with TCEP (10 mM) in 0.2 M phosphate buffer (pH 8.0) containing 10% D_2O. The HMBC signal for C3 was encircled.

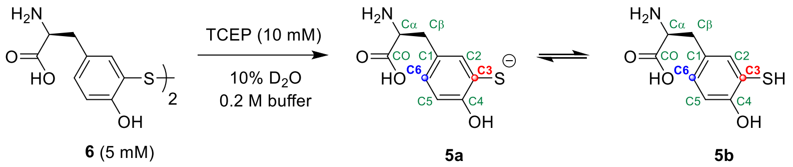

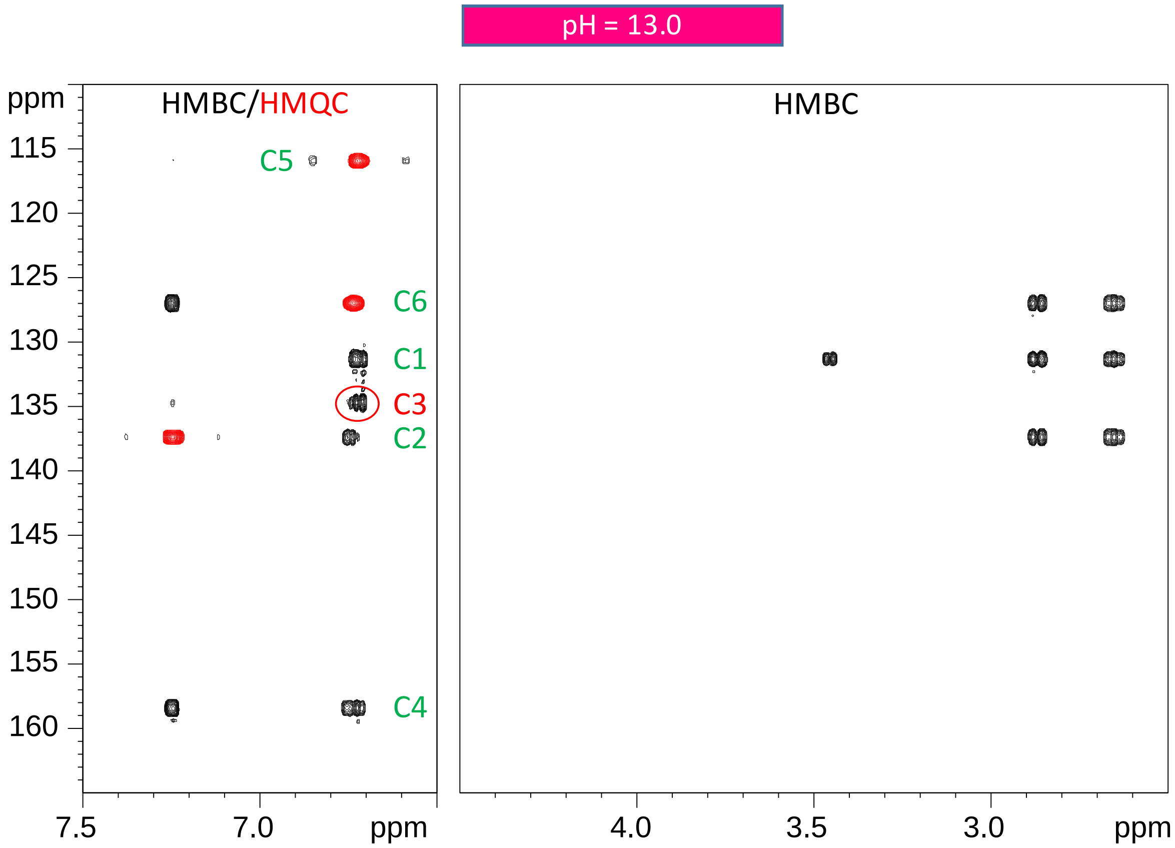

pH 13.0: HMBC/HMQC overlay for the measurement of **6** (5 mM) upon treatment with TCEP (10 mM) in 1% NaOH (pH 13.0) containing 10% D_2O. The HMBC signal for C3 was encircled.

6.2 FRET assay to determine reaction rates

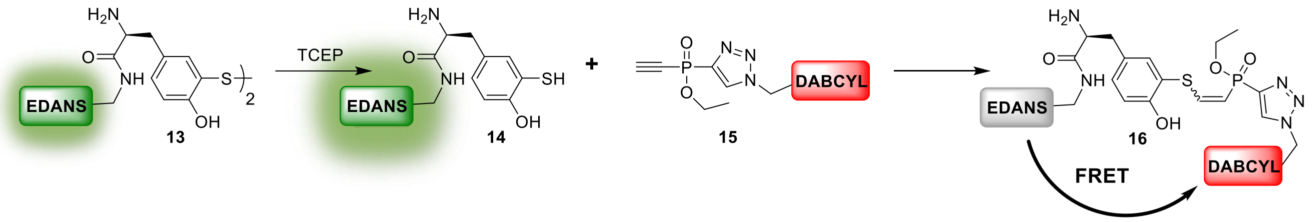

<u>Sample preparation – pH range (pH 4.5 – 8.5)</u>

A 96-well flat bottom black polystyrene plate (clear bottom) was used for the assay. Target wells were initially loaded with:

1) 95 µL of 0.2 M citrate/phosphate/tris buffer (pH 4.5 – 8.5; *see section 6.1 for details on buffers*), containing 10.5% (v/v) MeCN and 210.5 µM TCEP.

2) Addition of:

**100% fluorescence control**	**(n = 2):**	5.0 µL of **16** (2 mM in DMSO)
**0% fluorescence control**	**(n = 2):**	2.5 µL of **13** (2 mM in DMSO)
**Reaction mixture**	**(n = 2):**	2.5 µL of **13** (2 mM in DMSO)

3) Careful mixing of the solutions using a multichannel pipet; allow for a waiting time of 2.5 – 5 minutes before moving to the next step.

4) Addition of:

**100% fluorescence control**	**(n = 2):**	-
**0% fluorescence control**	**(n = 2):**	2.5 µL of DABCYL-NH_2 **21** (4 mM in DMSO)*
**Reaction mixture**	**(n = 2):**	2.5 µL of **15** (4 mM in DMSO)

We used DABCYL-NH_2 **21 instead of DABCYL-acid because the effect of DABCYL-acid was not similar across a large pH range. This is most likely due to the presence of an amine and a carboxylic acid. Compound **21** has two amino functionalities which makes its effect more reliable.*

5) Careful mixing of the solutions using a multichannel pipet, followed by starting the measurement.

Final conditions (10% MeCN, 5% DMSO):

**100% fluorescence control**	**(n = 2):**	TCEP (200 µM) + **16** (100 µM)
**0% fluorescence control**	**(n = 2):**	TCEP (200 µM) + **13** (50 µM) + **21** (100 µM)
**Reaction mixture**	**(n = 2):**	TCEP (200 µM) + **13** (50 µM) + **15** (100 µM)

Sample preparation – GSH competition (pH 6.0 – 8.5)

The GSH competition experiments were similar in sample preparation, with the clear exception for the first step to introduce GSH and additional TCEP (equimolar additional TCEP for GSH added):

0 eq GSH: 95 µL of 0.2 M phosphate/tris buffer (pH 4.5 – 8.5), containing 10.5% (v/v) MeCN and 210.5 µM TCEP.
5 eq GSH: 95 µL of 0.2 M phosphate/tris buffer (pH 4.5 – 8.5), containing 10.5% (v/v) MeCN, 526.3 µM GSH and (210.5 + 526.3) µM TCEP.
10 eq GSH: 95 µL of 0.2 M phosphate/tris buffer (pH 4.5 – 8.5), containing 10.5% (v/v) MeCN, 1052.6 µM GSH and (210.5 + 1052.6) µM TCEP.

Final conditions (10% MeCN, 5% DMSO):
100% fluorescence control (n = 2):
TCEP (200/700/1200 µM) + GSH (0/500/1000 µM) + **16** (100 µM)
0% fluorescence control (n = 2):
TCEP (200/700/1200 µM) + GSH (0/500/1000 µM) + **13** (50 µM) + **21** (100 µM)
Reaction mixture (n = 2):
TCEP (200/700/1200 µM) + GSH (0/500/1000 µM) + **13** (50 µM) + **15** (100 µM)

Measurement conditions

A Tecan Infinite 200 Pro plate reader was employed, utilizing a kinetic mode which measures every minute. The machine was set to 25°C, and the 96-well plate was shaken for 5 seconds prior to each measurement. The specific machine parameters are listed below:

**Target Temperature**	25 °C
**Kinetic**	
Shaking (Linear) Duration	5 s
Shaking (Linear) Amplitude	1 mm
**Fluorescence**	
Interval time	01:00 minute
Mode:	Fluorescence Top Reading
Excitation Wavelength	340 nm
Emission Wavelength	495 nm
Excitation Bandwidth	9 nm
Emission Bandwidth	20 nm
Gain (Manual)	90*
Number of Flashes	3
Integration Time	20 µs
Lag Time	0 µs
Settle Time	0 ms
Z-Position (Manual)	20000 µm

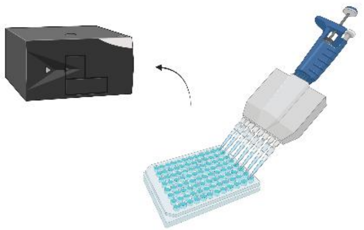

**For pH 4.5 – 5.0 – 5.5, a gain of 85 was used to ensure that the measured fluorescence would remain within the maximum fluorescence intensity of the machine (60.000 AU).*

Data processing

Normalized value (fraction)
= (value – min) / (max – min)

Concentration (M) =
fraction * 0.0001

Max / Min
average
per timepoint

RAW data
values

Time (min)	AVERAGE 100%	AVERAGE 0%	Normalized values (fraction)		Concentration (M)	
0	46144.5	811.5	0.966172545	0.939282642	9.66173E-05	9.39283E-05
1	45753.5	803	0.940412231	0.919300119	9.40412E-05	9.193E-05
2	45626.5	795	0.917814483	0.912037295	9.17814E-05	9.12037E-05
3.001667	45713	817	0.905158589	0.884978617	9.05159E-05	8.84979E-05
4.001667	45042	804	0.891767259	0.873977124	8.91767E-05	8.73977E-05
5.001667	45279.5	810.5	0.863331759	0.854719018	8.63332E-05	8.54719E-05
6.001667	44512.5	798.5	0.880301505	0.84312806	8.80302E-05	8.43128E-05
7.001667	44370	798.5	0.855387122	0.825229795	8.55387E-05	8.2523E-05
8.001667	44550.5	790	0.829469499	0.811782315	8.29469E-05	8.11782E-05
9.001667	44011.5	794	0.828206166	0.808862151	8.28206E-05	8.08862E-05
10.00167	43473.5	783.5	0.820695713	0.796989927	8.20696E-05	7.9699E-05
11.00167	43757.5	784	0.800679489	0.772452791	8.00679E-05	7.72453E-05
12.00167	43353.5	778.5	0.77976512	0.765437463	7.79765E-05	7.65437E-05
13.00167	42822.5	784.5	0.763321281	0.758278224	7.63321E-05	7.58278E-05
14.00167	42569	778	0.780167979	0.739920078	7.80168E-05	7.3992E-05
15.00333	42714	781.5	0.744255649	0.735026531	7.44256E-05	7.35027E-05
16.00167	42779.5	790.5	0.748041154	0.721200791	7.48041E-05	7.21201E-05
17.00333	42058	768.5	0.730585258	0.709829376	7.30585E-05	7.09829E-05
18.00167	41919	758.5	0.731878864	0.6992262	7.31879E-05	6.99226E-05
19.00167	42309	781.5	0.705303714	0.680813918	7.05304E-05	6.80814E-05
20.00333	41840	773.5	0.700656253	0.681005199	7.00656E-05	6.81005E-05
21.00333	41481.5	765	0.683948768	0.672331856	6.83949E-05	6.72332E-05
22.00333	41668	764	0.689223548	0.662233522	6.89224E-05	6.62234E-05
23.00333	41226	769.5	0.677629058	0.658274937	6.77629E-05	6.58275E-05
24.005	41456.5	770	0.655352513	0.642940533	6.55353E-05	6.42941E-05
25.00333	41107.5	777.5	0.660389288	0.635122737	6.60389E-05	6.35123E-05
26.00333	40771	754	0.643826374	0.63390559	6.43826E-05	6.33906E-05
27.00333	40628	783.5	0.64507021	0.623963157	6.4507E-05	6.23963E-05
28.00333	40736	755.5	0.630069659	0.615287453	6.3007E-05	6.15287E-05
29.00333	40547	761	0.630347358	0.613004574	6.30347E-05	6.13005E-05
30.00333	40514	764.5	0.616976314	0.598762249	6.16976E-05	5.98762E-05

1) RAW data were generated in Microsoft Excel by the Tecan Infinite 200 Pro plate reader.
For each experiment, a timeframe of 30 minutes was used for kinetic data calculations.

2) For each timepoint, the average value of the 100% (max) and 0% (min) fluorescence was used to obtain the normalized fluorescence value (fraction) for each reaction measurement using the formula: (value – min) / (max – min).
This value represents the fraction of starting material **14** left in the reaction mixture.

3) The normalized value was converted to concentration (M) by multiplying the value by 0.0001.

4) The data was plotted as 1/concentration (M^{-1}) on the y-axis and time (min) on the x-axis.

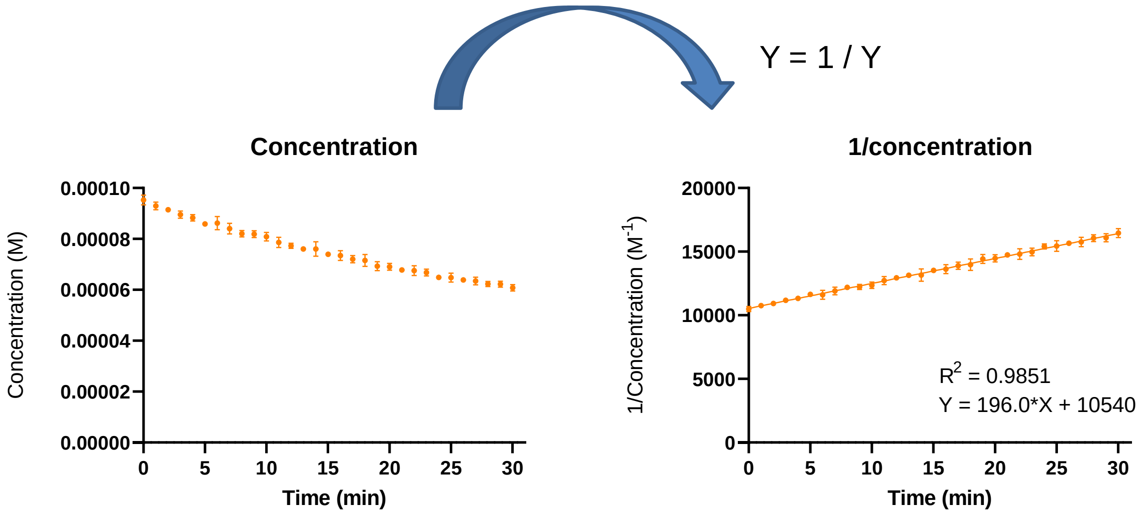

Prism:
Simple Linear Regression

5) The second order rate constant was determined for equal concentration of the two reactants (50 µM **13** → 100 µM **14; ** 100 µM **15**).

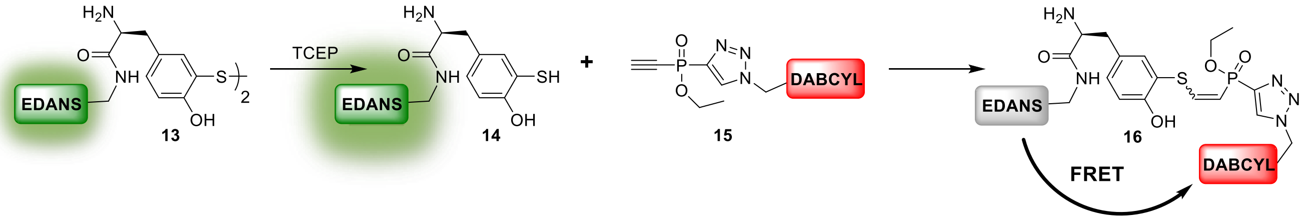

$$v = k[A][B] \qquad \boldsymbol{If}\ [\boldsymbol{A}] = [\boldsymbol{B}] \qquad d[A] = k\,dt$$

$$\frac{d[A]}{dt} = k[A][B] \qquad \frac{d[A]}{dt} = k[A]^2 \qquad \frac{1}{[A]} = kt + 1/[A_0]$$

Based on this, for the graph of 1/c over time, the corresponding linear slope (determined by simple linear regression in Graphpad Prism 10.3.1) is the second order rate constant (*k*).

Raw Data – Fig. S11-S19

Simple Linear Regression – General note

Simple linear regression was performed in Graphpad Prism 10.3.1 using time in minutes on the x-axis. The *k* values were manually converted from $M^{-1}min^{-1}$ to $M^{-1}s^{-1}$.

Simple Linear Regression - Results (pH 4.5 – 5.5; Fig. 3C)

Tabular results

	Simple linear regression Tabular results	A pH 4.5	B pH 5.0	C pH 5.5
1	**Best-fit values**			
2	Slope	22.58	68.47	124.4
3	Y-intercept	10187	10338	10481
4	X-intercept	-451.1	-151.0	-84.24
5	1/slope	0.04428	0.01461	0.008037
6				
7	**Std. Error**			
8	Slope	1.252	1.368	1.327
9	Y-intercept	21.87	23.89	23.18
10				
11	**95% Confidence Intervals**			
12	Slope	20.08 to 25.09	65.73 to 71.20	121.8 to 127.1
13	Y-intercept	10143 to 10231	10290 to 10386	10435 to 10528
14	X-intercept	-509.2 to -404.5	-157.9 to -144.6	-86.41 to -82.16
15				
16	**Goodness of Fit**			
17	R squared	0.8443	0.9766	0.9932
18	Sy.x	88.18	96.33	93.48
19				
20	**Is slope significantly non-zero?**			
21	F	325.4	2506	8786
22	DFn, DFd	1, 60	1, 60	1, 60
23	P value	<0.0001	<0.0001	<0.0001
24	Deviation from zero?	Significant	Significant	Significant
25				
26	**Equation**	Y = 22.58*X + 10187	Y = 68.47*X + 10338	Y = 124.4*X + 10481
27				
28	**Data**			
29	Number of X values	62	62	62
30	Maximum number of Y replicates	2	2	2
31	Total number of values	62	62	62
32	Number of missing values	0	0	0

Simple Linear Regression - Results (pH 6.0 – 7.0; Fig. 3C)

Tabular results

Simple linear regression
Tabular results

		D	E	F
		pH 6.0	pH 6.5	pH 7.0
1	**Best-fit values**			
2	Slope	199.4	204.9	222.0
3	Y-intercept	11125	11410	11548
4	X-intercept	-55.79	-55.69	-52.01
5	1/slope	0.005015	0.004881	0.004504
6				
7	**Std. Error**			
8	Slope	3.358	3.464	3.534
9	Y-intercept	58.64	60.50	61.73
10				
11	**95% Confidence Intervals**			
12	Slope	192.7 to 206.1	197.9 to 211.8	215.0 to 229.1
13	Y-intercept	11007 to 11242	11289 to 11531	11425 to 11672
14	X-intercept	-58.27 to -53.46	-58.19 to -53.36	-54.23 to -49.92
15				
16	**Goodness of Fit**			
17	R squared	0.9833	0.9831	0.9850
18	Sy.x	236.5	244.0	248.9
19				
20	**Is slope significantly non-zero?**			
21	F	3527	3498	3947
22	DFn, DFd	1, 60	1, 60	1, 60
23	P value	<0.0001	<0.0001	<0.0001
24	Deviation from zero?	Significant	Significant	Significant
25				
26	**Equation**	Y = 199.4*X + 11125	Y = 204.9*X + 11410	Y = 222.0*X + 11548
27				
28	**Data**			
29	Number of X values	62	62	62
30	Maximum number of Y replicates	2	2	2
31	Total number of values	62	62	62
32	Number of missing values	0	0	0

Simple Linear Regression - Results (pH 7.4 – 8.5; Fig. 3C)

Tabular results

Simple linear regression
Tabular results

		G	H	I
		pH 7.4	pH 8.0	pH 8.5
1	**Best-fit values**			
2	Slope	267.0	257.0	244.0
3	Y-intercept	11851	11589	10676
4	X-intercept	-44.39	-45.10	-43.76
5	1/slope	0.003746	0.003892	0.004099
6				
7	**Std. Error**			
8	Slope	2.230	3.229	2.275
9	Y-intercept	38.94	56.39	39.74
10				
11	**95% Confidence Intervals**			
12	Slope	262.5 to 271.4	250.5 to 263.4	239.4 to 248.5
13	Y-intercept	11773 to 11929	11477 to 11702	10597 to 10756
14	X-intercept	-45.41 to -43.41	-46.67 to -43.61	-44.89 to -42.67
15				
16	**Goodness of Fit**			
17	R squared	0.9958	0.9906	0.9948
18	Sy.x	157.0	227.4	160.3
19				
20	**Is slope significantly non-zero?**			
21	F	14335	6333	11497
22	DFn, DFd	1, 60	1, 60	1, 60
23	P value	<0.0001	<0.0001	<0.0001
24	Deviation from zero?	Significant	Significant	Significant
25				
26	**Equation**	Y = 267.0*X + 11851	Y = 257.0*X + 11589	Y = 244.0*X + 10676
27				
28	**Data**			
29	Number of X values	62	62	62
30	Maximum number of Y replicates	2	2	2
31	Total number of values	62	62	62
32	Number of missing values	0	0	0

Simple Linear Regression - Results (pH 6.0 – GSH competition; Fig. 3D)

Tabular results

	Simple linear regression<br>Tabular results	A<br>0 eq GSH	B<br>5 eq GSH	C<br>10 eq GSH
1	**Best-fit values**			
2	Slope	182.7	182.5	184.3
3	Y-intercept	11001	10939	10875
4	X-intercept	-60.20	-59.95	-59.02
5	1/slope	0.005472	0.005480	0.005427
6				
7	**Std. Error**			
8	Slope	2.992	2.801	2.662
9	Y-intercept	52.25	48.91	46.49
10				
11	**95% Confidence Intervals**			
12	Slope	176.8 to 188.7	176.9 to 188.1	178.9 to 189.6
13	Y-intercept	10897 to 11106	10841 to 11037	10782 to 10968
14	X-intercept	-62.77 to -57.80	-62.34 to -57.70	-61.24 to -56.93
15				
16	**Goodness of Fit**			
17	R squared	0.9842	0.9861	0.9876
18	Sy.x	210.7	197.2	187.5
19				
20	**Is slope significantly non-zero?**			
21	F	3731	4244	4792
22	DFn, DFd	1, 60	1, 60	1, 60
23	P value	<0.0001	<0.0001	<0.0001
24	Deviation from zero?	Significant	Significant	Significant
25				
26	**Equation**	Y = 182.7*X + 11001	Y = 182.5*X + 10939	Y = 184.3*X + 10875
27				
28	**Data**			
29	Number of X values	62	62	62
30	Maximum number of Y replicates	2	2	2
31	Total number of values	62	62	62
32	Number of missing values	0	0	0

Simple Linear Regression - Results (pH 6.5 – GSH competition; Fig. 3D)

	Simple linear regression Tabular results	A 0 eq GSH	B 5 eq GSH	C 10 eq GSH
1	**Best-fit values**			
2	Slope	243.7	246.1	248.5
3	Y-intercept	11547	11741	11401
4	X-intercept	-47.39	-47.71	-45.87
5	1/slope	0.004104	0.004064	0.004023
6				
7	**Std. Error**			
8	Slope	2.462	2.746	4.429
9	Y-intercept	43.00	47.96	77.35
10				
11	**95% Confidence Intervals**			
12	Slope	238.8 to 248.6	240.6 to 251.6	239.7 to 257.4
13	Y-intercept	11461 to 11633	11645 to 11837	11246 to 11556
14	X-intercept	-48.69 to -46.14	-49.16 to -46.33	-48.15 to -43.75
15				
16	**Goodness of Fit**			
17	R squared	0.9939	0.9926	0.9813
18	Sy.x	173.4	193.4	311.9
19				
20	**Is slope significantly non-zero?**			
21	F	9797	8031	3149
22	DFn, DFd	1, 60	1, 60	1, 60
23	P value	<0.0001	<0.0001	<0.0001
24	Deviation from zero?	Significant	Significant	Significant
25				
26	**Equation**	Y = 243.7*X + 11547	Y = 246.1*X + 11741	Y = 248.5*X + 11401
27				
28	**Data**			
29	Number of X values	62	62	62
30	Maximum number of Y replicates	2	2	2
31	Total number of values	62	62	62
32	Number of missing values	0	0	0

Simple Linear Regression - Results (pH 7.0 – GSH competition; Fig. 3D)

Tabular results

	Simple linear regression<br>Tabular results	A<br>0 eq GSH	B<br>5 eq GSH	C<br>10 eq GSH
1	**Best-fit values**			
2	Slope	218.1	224.2	209.4
3	Y-intercept	11741	11855	11712
4	X-intercept	-53.84	-52.87	-55.94
5	1/slope	0.004586	0.004460	0.004776
6				
7	**Std. Error**			
8	Slope	4.549	2.560	2.695
9	Y-intercept	79.45	44.70	47.06
10				
11	**95% Confidence Intervals**			
12	Slope	209.0 to 227.2	219.1 to 229.3	204.0 to 214.8
13	Y-intercept	11582 to 11900	11766 to 11944	11618 to 11806
14	X-intercept	-56.86 to -51.06	-54.47 to -51.34	-57.83 to -54.14
15				
16	**Goodness of Fit**			
17	R squared	0.9745	0.9922	0.9902
18	Sy.x	320.4	180.3	189.8
19				
20	**Is slope significantly non-zero?**			
21	F	2297	7673	6036
22	DFn, DFd	1, 60	1, 60	1, 60
23	P value	<0.0001	<0.0001	<0.0001
24	Deviation from zero?	Significant	Significant	Significant
25				
26	**Equation**	Y = 218.1*X + 11741	Y = 224.2*X + 11855	Y = 209.4*X + 11712
27				
28	**Data**			
29	Number of X values	62	62	62
30	Maximum number of Y replicates	2	2	2
31	Total number of values	62	62	62
32	Number of missing values	0	0	0

Simple Linear Regression - Results (pH 7.4 – GSH competition; Fig. 3D)

Tabular results

Simple linear regression Tabular results	A<br>0 eq GSH	B<br>5 eq GSH	C<br>10 eq GSH
1 **Best-fit values**			
2 Slope	278.0	218.6	195.3
3 Y-intercept	11967	11537	11652
4 X-intercept	-43.05	-52.78	-59.65
5 1/slope	0.003598	0.004575	0.005119
6			
7 **Std. Error**			
8 Slope	2.578	4.262	3.262
9 Y-intercept	45.02	74.44	56.97
10			
11 **95% Confidence Intervals**			
12 Slope	272.8 to 283.1	210.0 to 227.1	188.8 to 201.9
13 Y-intercept	11877 to 12057	11388 to 11686	11538 to 11766
14 X-intercept	-44.16 to -41.98	-55.56 to -50.22	-62.25 to -57.22
15			
16 **Goodness of Fit**			
17 R squared	0.9949	0.9777	0.9835
18 Sy.x	181.6	300.2	229.7
19			
20 **Is slope significantly non-zero?**			
21 F	11625	2630	3586
22 DFn, DFd	1, 60	1, 60	1, 60
23 P value	<0.0001	<0.0001	<0.0001
24 Deviation from zero?	Significant	Significant	Significant
25			
26 **Equation**	Y = 278.0*X + 11967	Y = 218.6*X + 11537	Y = 195.3*X + 11652
27			
28 **Data**			
29 Number of X values	62	62	62
30 Maximum number of Y replicates	2	2	2
31 Total number of values	62	62	62
32 Number of missing values	0	0	0

Simple Linear Regression - Results (pH 8.0 – GSH competition; Fig. 3D)

Tabular results

Simple linear regression Tabular results		A 0 eq GSH	B 5 eq GSH	C 10 eq GSH
1	**Best-fit values**			
2	Slope	262.1	171.3	118.3
3	Y-intercept	11716	11837	11955
4	X-intercept	-44.71	-69.08	-101.0
5	1/slope	0.003816	0.005836	0.008450
6				
7	**Std. Error**			
8	Slope	2.994	3.736	4.163
9	Y-intercept	52.29	65.25	72.71
10				
11	**95% Confidence Intervals**			
12	Slope	256.1 to 268.0	163.9 to 178.8	110.0 to 126.7
13	Y-intercept	11611 to 11820	11706 to 11967	11809 to 12100
14	X-intercept	-46.12 to -43.36	-72.94 to -65.55	-109.8 to -93.37
15				
16	**Goodness of Fit**			
17	R squared	0.9922	0.9723	0.9309
18	Sy.x	210.9	263.1	293.2
19				
20	**Is slope significantly non-zero?**			
21	F	7660	2103	808.0
22	DFn, DFd	1, 60	1, 60	1, 60
23	P value	<0.0001	<0.0001	<0.0001
24	Deviation from zero?	Significant	Significant	Significant
25				
26	**Equation**	Y = 262.1*X + 11716	Y = 171.3*X + 11837	Y = 118.3*X + 11955
27				
28	**Data**			
29	Number of X values	62	62	62
30	Maximum number of Y replicates	2	2	2
31	Total number of values	62	62	62
32	Number of missing values	0	0	0

Simple Linear Regression - Results (pH 8.5 – GSH competition; Fig. 3D)

Tabular results

	Simple linear regression<br>Tabular results	A<br>0 eq GSH	B<br>5 eq GSH	C<br>10 eq GSH
1	**Best-fit values**			
2	Slope	220.0	141.0	78.54
3	Y-intercept	10640	11433	11128
4	X-intercept	-48.36	-81.06	-141.7
5	1/slope	0.004546	0.007090	0.01273
6				
7	**Std. Error**			
8	Slope	3.601	3.502	4.809
9	Y-intercept	62.89	61.16	83.98
10				
11	**95% Confidence Intervals**			
12	Slope	212.8 to 227.2	134.0 to 148.1	68.92 to 88.16
13	Y-intercept	10514 to 10766	11311 to 11555	10960 to 11296
14	X-intercept	-50.53 to -46.33	-86.10 to -76.49	-163.6 to -124.5
15				
16	**Goodness of Fit**			
17	R squared	0.9842	0.9643	0.8164
18	Sy.x	253.6	246.6	338.7
19				
20	**Is slope significantly non-zero?**			
21	F	3732	1622	266.8
22	DFn, DFd	1, 60	1, 60	1, 60
23	P value	<0.0001	<0.0001	<0.0001
24	Deviation from zero?	Significant	Significant	Significant
25				
26	**Equation**	Y = 220.0*X + 10640	Y = 141.0*X + 11433	Y = 78.54*X + 11128
27				
28	**Data**			
29	Number of X values	62	62	62
30	Maximum number of Y replicates	2	2	2
31	Total number of values	62	62	62
32	Number of missing values	0	0	0

6.3 FRET assay to determine aryl thiol-ETP/Maleimide conjugate stability

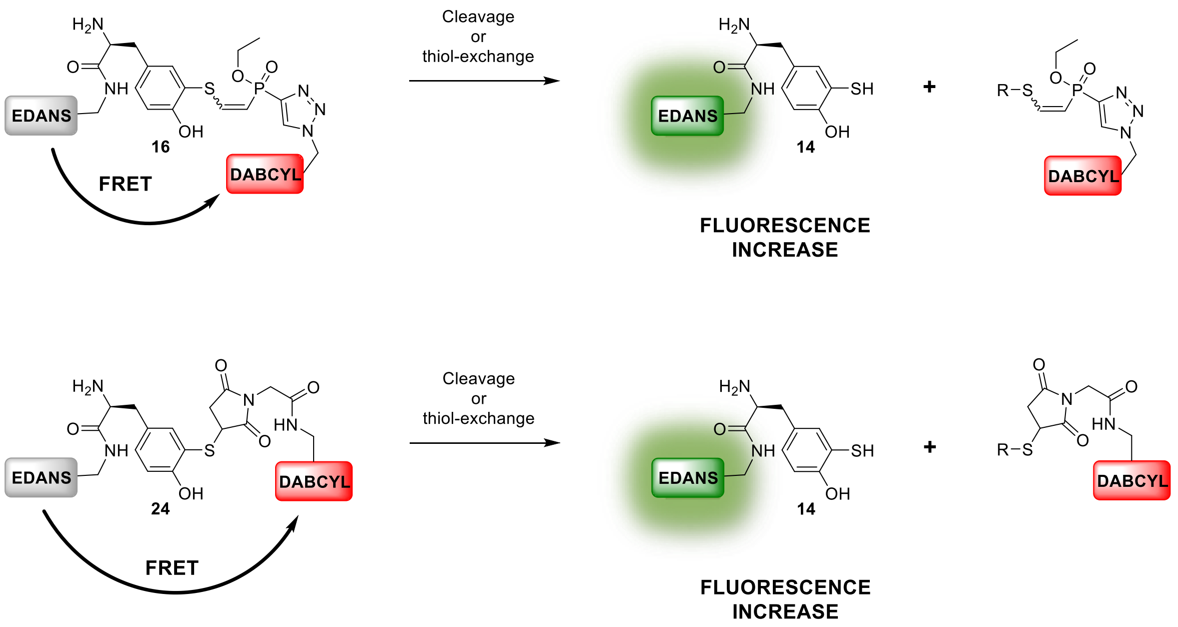

Sample preparation

Stability studies were conducted in a 96-well flat bottom black polystyrene plate (clear bottom) as previously described by Stieger et al.[1,2] Human serum was purchased from Sigma Aldrich and used without further manipulation. Reduced glutathione was dissolved in PBS, neutralized and diluted to the final concentration of 10.5 mM.

Target wells were initially loaded with:

1) 95 µL of test solution (human serum; 10.5 mM reduced GSH in PBS pH 7.4; PBS pH 7.4; 0.2 M citrate buffer pH 4.5; 0.2 M tris buffer pH 8.5; *see section 6.1 for details on buffers*).

2) Addition of:

**100% fluorescence control**	**(n ≥ 3):**	5.0 µL of EDANS & DABCYL-NH_2 **21** (200 µM in DMSO)
**Solvent background control**	**(n ≥ 3):**	5.0 µL of DMSO
**Stability experiment**	**(n ≥ 3):**	5.0 µL of **16** or **24** (200 µM in DMSO)

3) Careful mixing of the solutions using a multichannel pipet, followed by starting the measurement.

Final conditions (5% DMSO):

**100% fluorescence control**	**(n ≥ 3):**	EDANS (10 µM), **21** (10 µM)
**Solvent background control**	**(n ≥ 3):**	-
**Stability experiment**	**(n ≥ 3):**	**16** or **24** (10 µM)

Measurement conditions

A Tecan Infinite 200 Pro plate reader was employed, utilizing single measurements at indicated timepoints. The specific machine parameters are listed below:

**Target Temperature**	25 °C
Mode:	Fluorescence Top Reading
Excitation Wavelength	340 nm
Emission Wavelength	495 nm
Excitation Bandwidth	9 nm
Emission Bandwidth	20 nm
Gain (Manual)	100
Number of Flashes	25
Integration Time	20 µs
Lag Time	0 µs
Settle Time	0 ms
Z-Position (Manual)	20000 µm

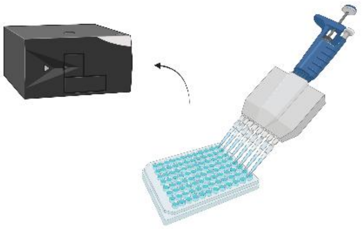

Collected datapoints

16:
human serum: 0, 24, 43, 50, 69, 96 h.
10 mM reduced GSH in PBS pH 7.4, PBS pH 7.4, 0.2 M citrate buffer pH 4.5, 0.2 M tris buffer pH 8.5: 0, 16, 24, 36, 148, 192 h.

24:
human serum, 10 mM reduced GSH in PBS pH 7.4:
0, 0.16, 1, 2, 3, 5, 24, 50 h.

Data processing

1) RAW data were generated in Microsoft Excel by the Tecan Infinite 200 Pro plate reader.

2) For each timepoint, the measured values were subtracted by the fluorescence measured in the solvent background control for each corresponding test solution.

2) For each timepoint, the adjusted values were normalized to the 100% fluorescence control (10 µM EDANS & 10 µM DABCYL-NH2 (**21**)) in the corresponding test solution.

This allowed the calculation of stability datapoints using:
1 - % cleavage

Where % cleavage was calculated using:
(value – solvent background) / (average of (100% control – solvent background))

7. Experimental Procedures – Biochemistry & Bioconjugation

7.1 SUMO-TTL expression and purification

SUMO-TTL expression was based on previously published protocols.[5,6] The TTL (*Canis lupus*) coding sequence was amplified from a mammalian expression vector,[15] cloned into pET28-SUMO3 (EMBL-Heidelberg, Protein Expression Facility) and expressed in *E. coli* BL21(DE3) as SUMO-TTL fusion protein with an *N*-terminal 6xHis-Tag.

BL21(DE3) *E. coli* were transformed with the SUMO-TTL plasmid by heat-shock, followed by inoculation onto LB-kanamycin agar plates and overnight incubation at 37°C. Starter cultures were made by inoculating single colonies into 5 mL of LB-kanamycin broth and further incubation at 37°C and 180 rpm. Starter cultures were added into 500 mL of LB-kanamycin broth and incubated at 37°C with 180 rpm shaking until the optical density at 600 nm (OD_{600}) reached 0.6. The bacterial culture was cooled in the cold room for 30 min before the induction of expression by adding isopropyl ß-D-1-thiogalactopyranoside (IPTG) to a final concentration of 0.5 mM and incubation at 18°C with 180 rpm shaking overnight. Induced cells were collected by centrifugation (4000 *g*, 4 °C, 15 min) and stored at −20 °C until purification.

The pellet of cells was defrosted on ice, resuspended in PBS, centrifuged (4000 *g*, 4°C, 15 min) and incubated in lysis buffer (PBS containing Lysozyme (100 µg/mL), DNAse (25 µg/mL) and PMSF (2 mM)) followed by sonication (2×, 6 min, 25% amplitude, max. 14°C) and debris centrifugation (50000 *g*, 4°C, 15 min). SUMO-TTL was manually purified from the lysate using HisPur™ Ni-NTA resin (Thermo Scientific™; 2 mL; 10 mM imidazole in PBS → 500 mM imidazole in PBS). Elution fractions were pooled and concentrated to ≤ 0.5 mL by ultrafiltration (10 kDa MWCO). SUMO-TTL was further purified by size exclusion chromatography (SEC; NGC Chromatography System, BioRad) using a Superdex™ 75 16/60 column (GE Healthcare) in **TTL buffer** (20 mM 3-(*N*-morpholino)propanesulfonic acid (MOPS) pH 7.0, 100 mM KCl, 10 mM $MgCl_2$, 10% (v/v) propane-1,2-diol). Peak fractions were pooled, concentrated to 8.5 – 30 µM, aliquoted, shock-frozen and stored at −70 °C.

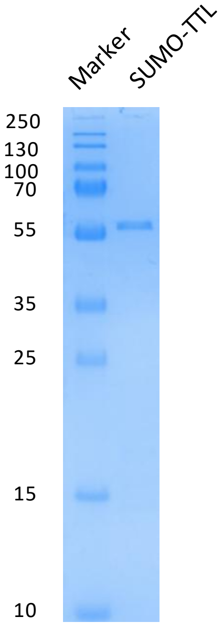

7.2 Tub-tag® labeling of 5(6)-CF-Tub-tag® peptide 7

Tyrosination reactions were performed in a 250 µL solution using the following conditions: 200 µM 5(6)-CF-Tub-tag® (**7**), 1 µM SUMO-TTL (0.5 mol%), 1.25 mM thio-tyrosine derivative **3**$_{ab}$ **/ 6**, 25 mM TCEP, 5 mM ATP in **TTL buffer** (20 mM 3-(*N*-morpholino)propanesulfonic acid (MOPS) pH 7.0, 100 mM KCl, 10 mM $MgCl_2$, 10% (v/v) propane-1,2-diol) at 37°C, 850 rpm shaking. For **3**$_{ab}$, 2.5 vol% DMSO was used as co-solvent for solubility.

Important notes:

- ATP stock solutions (25 mM in TTL buffer) were prepared from ATP · 2 Na and neutralized using 2 molar equivalents of KOH. Stock solutions were aliquoted, stored at −20°C and thawed before use.

- TCEP stock solutions were prepared by diluting commercially available 0.5 M aliquots (pH 7.0, adjusted with ammonium hydroxide) from Sigma Aldrich (Merck, USA) into the desired concentration with TTL buffer. Alternatively, stock solutions were prepared from TCEP · HCl and TTL buffer using 3.25 molar equivalents of KOH for neutralization. Stock solutions were aliquoted, stored at −20°C and thawed before use.

- Stock solutions for tyrosine derivatives (50 mM in DMSO for **3**$_{ab}$ and 50 mM in TTL buffer for **6**) and CF-Tub-tag peptide **7** (5 mM in TTL buffer) were aliquoted, stored at −20°C and thawed before use.

- SUMO-TTL stock solution aliquots (8.5 µM in TTL buffer) were stored at −70°C (see section 7.1). Aliquots were thawed on ice, carefully mixed and directly added to the reaction mixture as the final component. After carefully homogenizing the reaction mixture, the tyrosination reaction was launched at 37°C, 850 rpm shaking.

Analysis:

Crude reaction mixture samples (25 µL) were quenched with 1% TFA (25 µL) at indicated times and analyzed using LC-MS (C18, 5 → 95% MeCN in H_2O, 0.1% TFA, 15 min, 5 µL injection). Relative quantities of substrate and product peptide were determined from the corresponding peak areas in the UV spectra (λ = 220 nm).

Quantification of chemo-enzymatic reaction (Figure 2C; Figure S1).

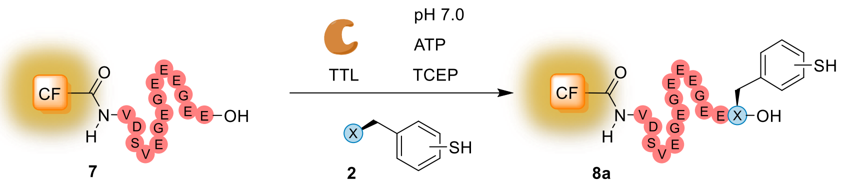

Relative signal quantification – incorporation of 2 – 30 / 60 min (Representative dataset)

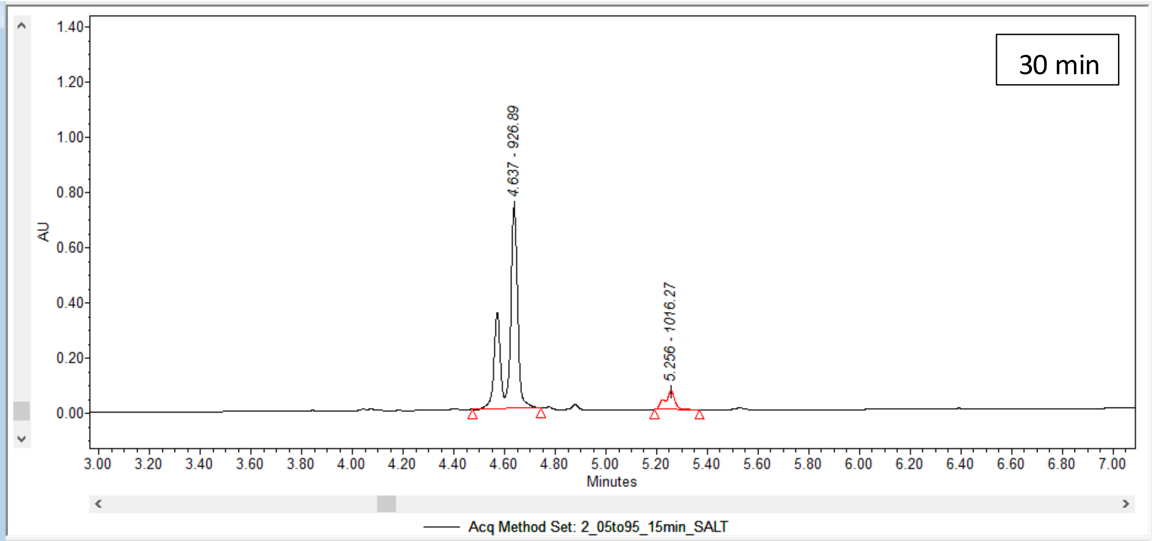

	Name	Migration Time (min)	Area (µV*sec)	% Area	Height (µV)	Int Type	Amount	Units	Peak Type	Peak Codes	Corrected Area~ (µV*sec)	MS Match1 Score	MS Match2 Score	MS Match3 Score	Base Peak (Combined) (m/z)	Assigned Mass	Assigned Mass Value (m/z)	Notes
1		4.637	2002341	91.70	730690	bb			Unknown						926.88			
2		5.256	181139	8.30	66152	bb			Unknown						1016.35			

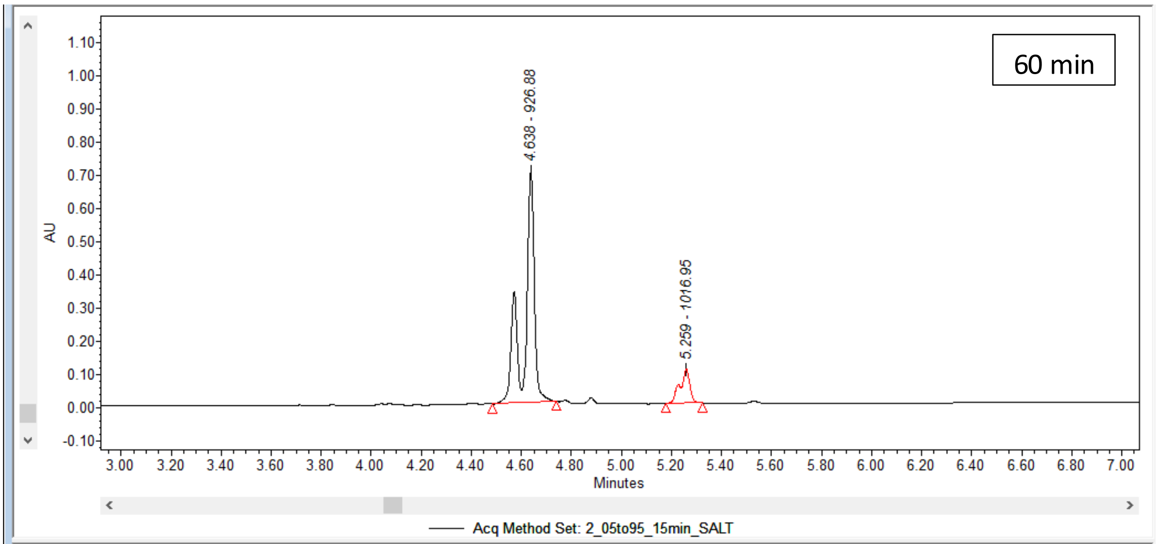

	Name	Migration Time (min)	Area (µV*sec)	% Area	Height (µV)	Int Type	Amount	Units	Peak Type	Peak Codes	Corrected Area~ (µV*sec)	MS Match1 Score	MS Match2 Score	MS Match3 Score	Base Peak (Combined) (m/z)	Assigned Mass	Assigned Mass Value (m/z)	Notes
1		4.638	1927111	87.29	696414	bb			Unknown						926.94			
2		5.259	280517	12.71	101479	bb			Unknown						1016.45			

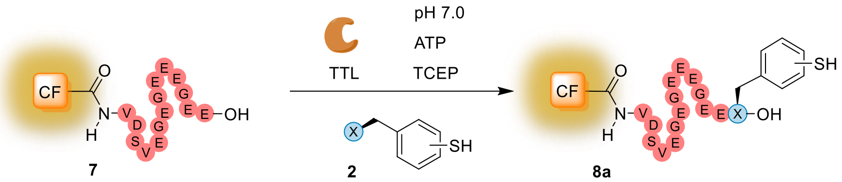

7 2 8a

Relative signal quantification – incorporation of 2 – 120 / 180 min (Representative dataset)

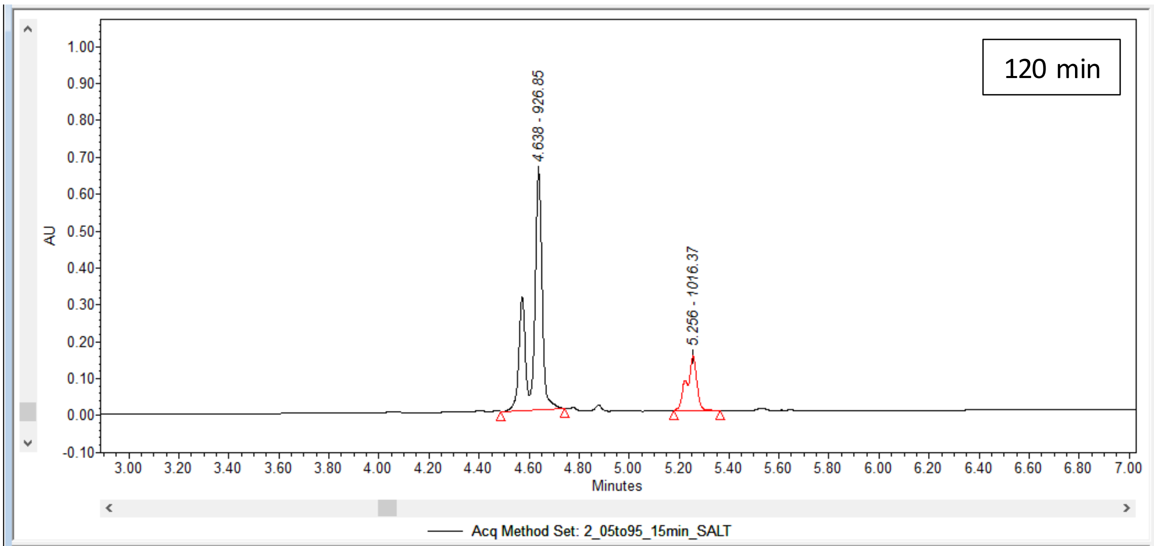

	Name	Migration Time (min)	Area (µV*sec)	% Area	Height (µV)	Int Type	Amount	Units	Peak Type	Peak Codes	Corrected Area~ (µV*sec)	MS Match1 Score	MS Match2 Score	MS Match3 Score	Base Peak (Combined) (m/z)	Assigned Mass	Assigned Mass Value (m/z)	Notes
1		4.638	1803815	81.31	645535	bb			Unknown						926.89			
2		5.256	414650	18.69	147863	bb			Unknown						1016.50			

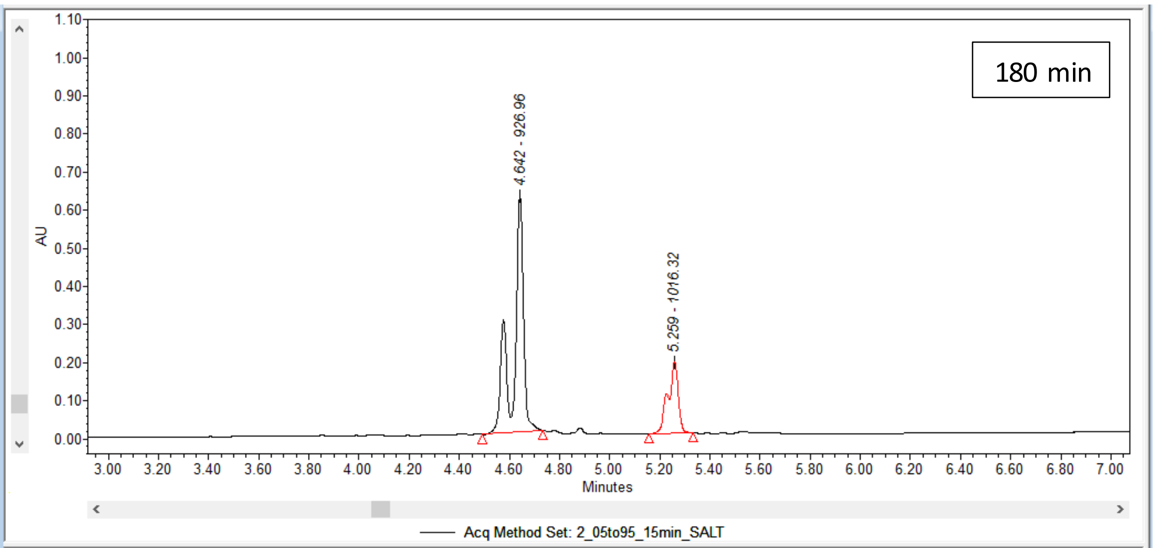

	Name	Migration Time (min)	Area (µV*sec)	% Area	Height (µV)	Int Type	Amount	Units	Peak Type	Peak Codes	Corrected Area~ (µV*sec)	MS Match1 Score	MS Match2 Score	MS Match3 Score	Base Peak (Combined) (m/z)	Assigned Mass	Assigned Mass Value (m/z)	Notes
1		4.642	1722752	76.57	620662	bb			Unknown						926.90			
2		5.259	527280	23.43	187999	bb			Unknown						1016.54			

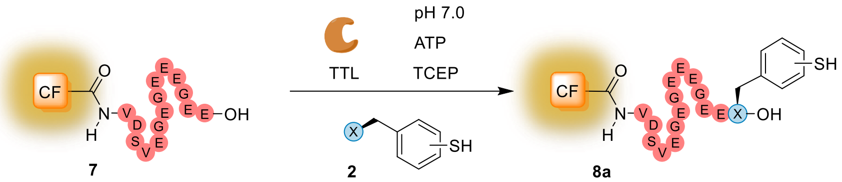

7 **2** **8a**

Relative signal quantification – incorporation of 2 – 300 / 420 min (Representative dataset)

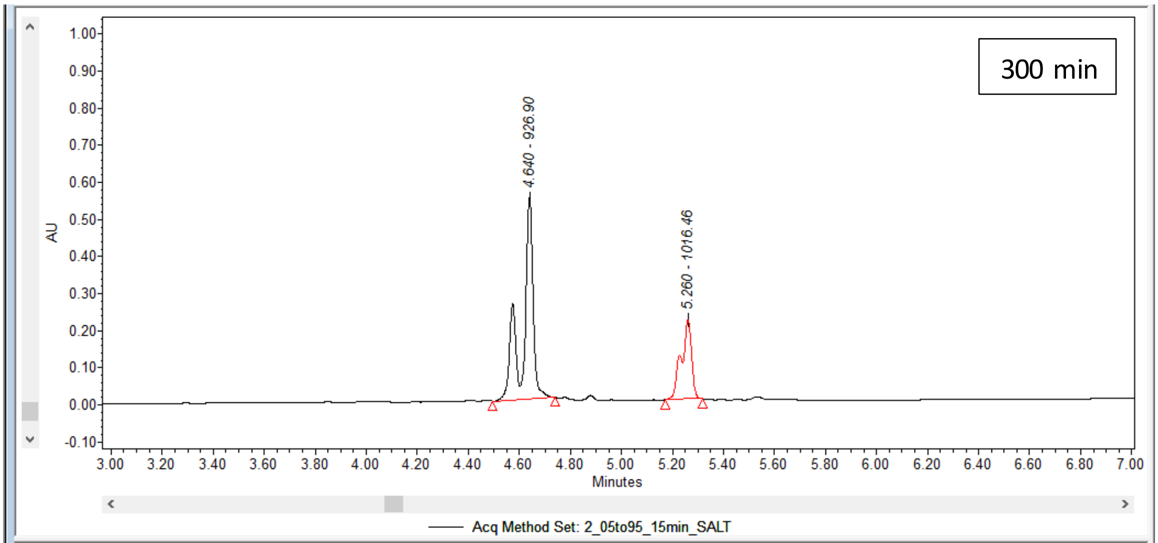

	Name	Migration Time (min)	Area (µV*sec)	% Area	Height (µV)	Int Type	Amount	Units	Peak Type	Peak Codes	Corrected Area~ (µV*sec)	MS Match1 Score	MS Match2 Score	MS Match3 Score	Base Peak (Combined) (m/z)	Assigned Mass	Assigned Mass Value (m/z)	Notes
1		4.640	1524342	71.78	542873	bb			Unknown						926.90			
2		5.260	599339	28.22	214637	bb			Unknown						1016.46			

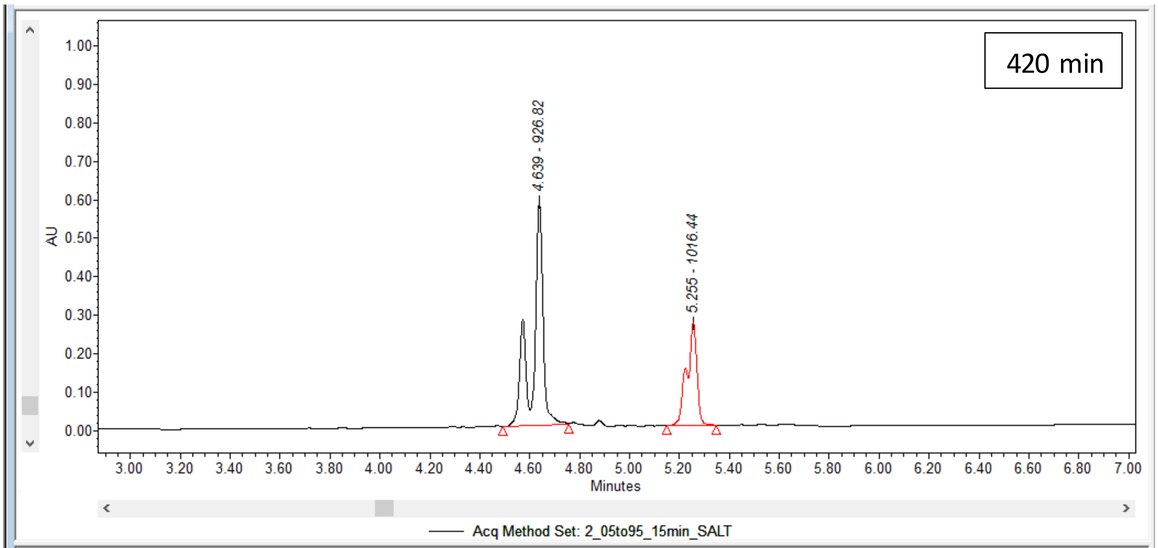

	Name	Migration Time (min)	Area (µV*sec)	% Area	Height (µV)	Int Type	Amount	Units	Peak Type	Peak Codes	Corrected Area~ (µV*sec)	MS Match1 Score	MS Match2 Score	MS Match3 Score	Base Peak (Combined) (m/z)	Assigned Mass	Assigned Mass Value (m/z)	Notes
1		4.639	1659722	68.79	580650	bb			Unknown						926.89			
2		5.255	753114	31.21	265984	bb			Unknown						1016.51			

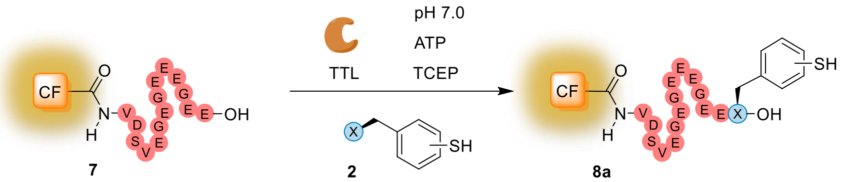

7 **2** **8a**

Relative signal quantification – incorporation of 2 – 24 h
(Representative dataset)

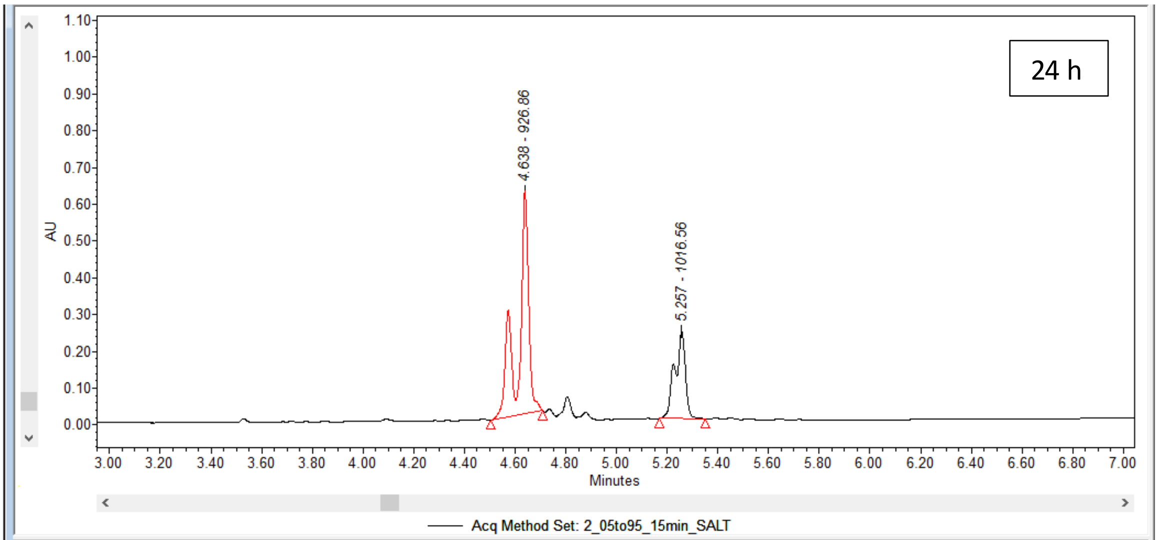

	Name	Migration Time (min)	Area (µV*sec)	% Area	Height (µV)	Int Type	Amount	Units	Peak Type	Peak Codes	Corrected Area~ (µV*sec)	MS Match1 Score	MS Match2 Score	MS Match3 Score	Base Peak (Combined) (m/z)	Assigned Mass	Assigned Mass Value (m/z)	Notes
1		4.638	1715885	71.16	605488	bb			Unknown						926.88			
2		5.257	695287	28.84	239390	bb			Unknown						1016.50			

Quantification of chemo-enzymatic reaction (Figure 2D; Figure S2)

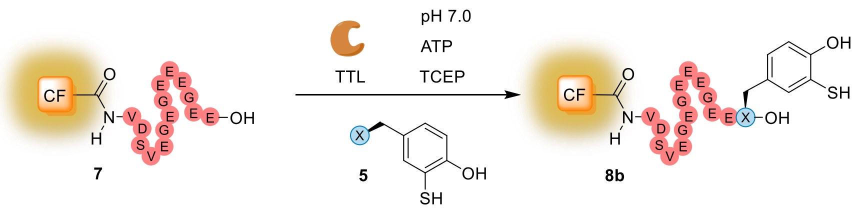

Relative signal quantification – incorporation of 5 – 30 / 60 min (Representative dataset)

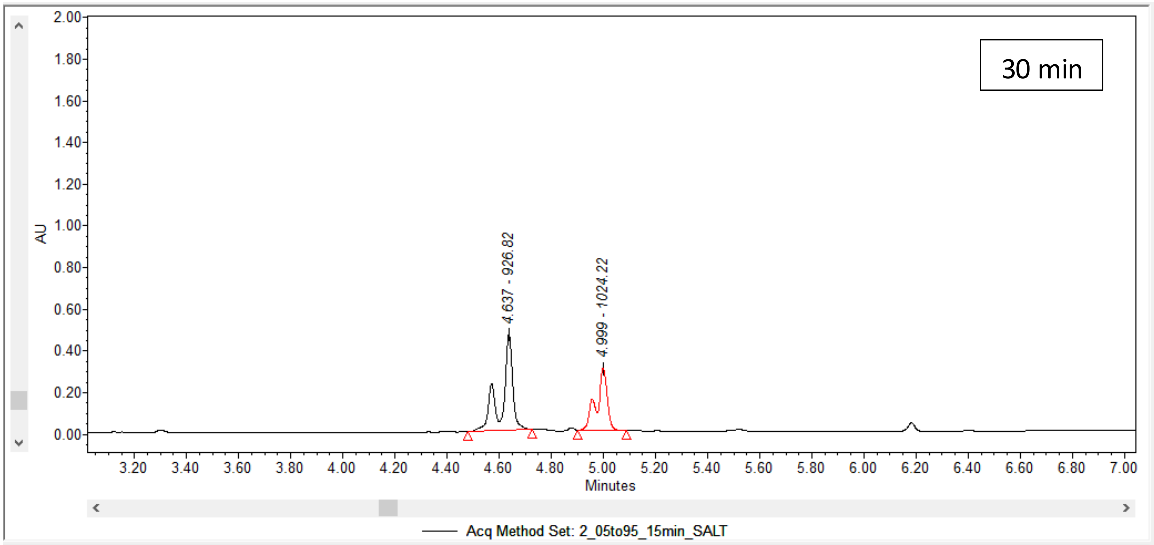

	Name	Migration Time (min)	Area (µV*sec)	% Area	Height (µV)	Int Type	Amount	Units	Peak Type	Peak Codes	Corrected Area~ (µV*sec)	MS Match1 Score	MS Match2 Score	MS Match3 Score	Base Peak (Combined) (m/z)	Assigned Mass	Assigned Mass Value (m/z)	Notes
1		4.637	1346506	61.13	461190	bb			Unknown						926.91			
2		4.999	856304	38.87	300658	bb			Unknown						1024.47			

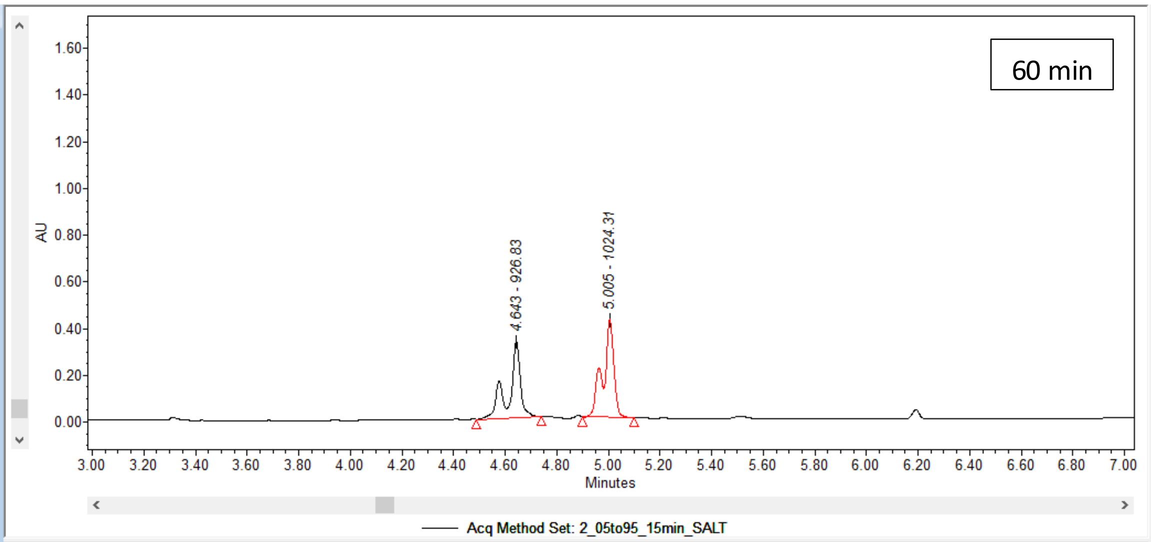

	Name	Migration Time (min)	Area (µV*sec)	% Area	Height (µV)	Int Type	Amount	Units	Peak Type	Peak Codes	Corrected Area~ (µV*sec)	MS Match1 Score	MS Match2 Score	MS Match3 Score	Base Peak (Combined) (m/z)	Assigned Mass	Assigned Mass Value (m/z)	Notes
1		4.643	1013279	45.22	324616	bb			Unknown						926.86			
2		5.005	1227475	54.78	418686	bb			Unknown						1024.49			

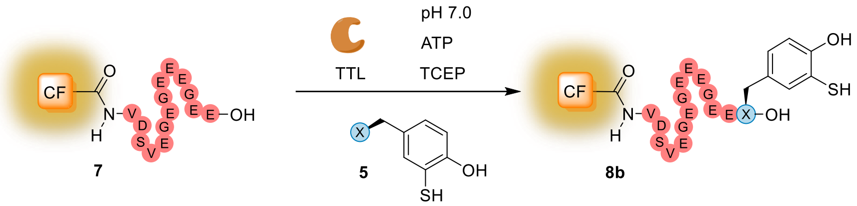

Relative signal quantification – incorporation of 5 – 120 / 180 min (Representative dataset)

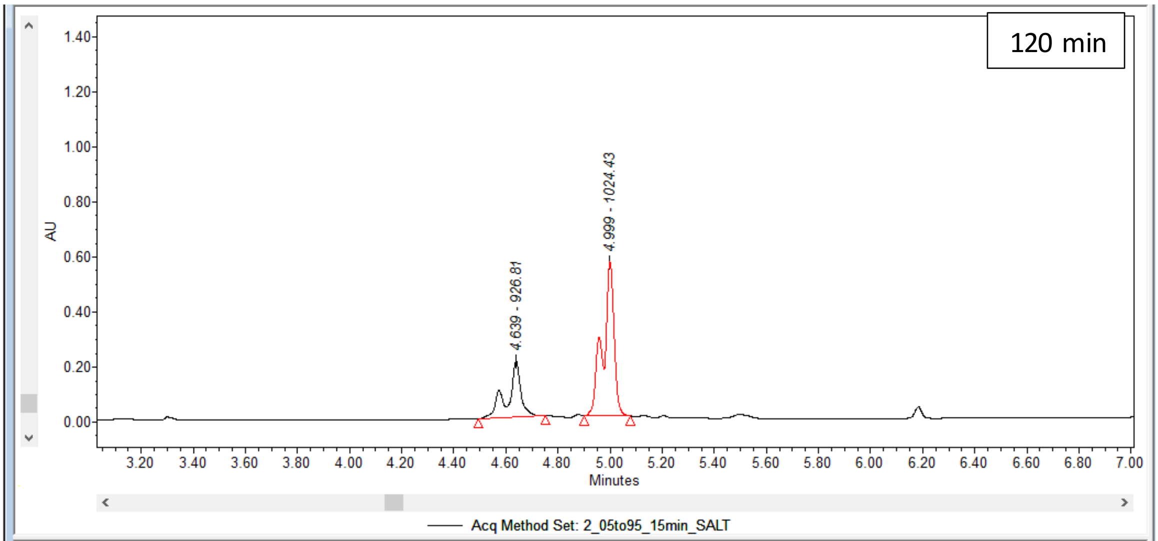

	Name	Migration Time (min)	Area (µV*sec)	% Area	Height (µV)	Int Type	Amount	Units	Peak Type	Peak Codes	Corrected Area~ (µV*sec)	MS Match1 Score	MS Match2 Score	MS Match3 Score	Base Peak (Combined) (m/z)	Assigned Mass	Assigned Mass Value (m/z)	Notes
1		4.639	726436	30.50	204498	bb			Unknown						926.79			
2		4.999	1655572	69.50	560471	bb			Unknown						1024.51			

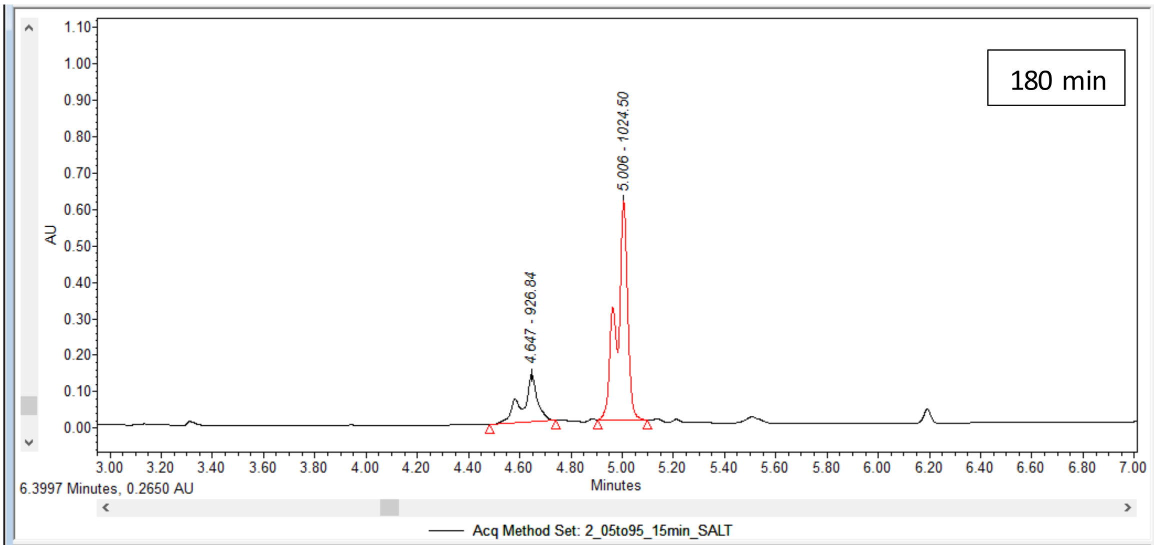

	Name	Migration Time (min)	Area (µV*sec)	% Area	Height (µV)	Int Type	Amount	Units	Peak Type	Peak Codes	Corrected Area~ (µV*sec)	MS Match1 Score	MS Match2 Score	MS Match3 Score	Base Peak (Combined) (m/z)	Assigned Mass	Assigned Mass Value (m/z)	Notes
1		4.647	509006	22.12	129142	bb			Unknown						926.86			
2		5.006	1792258	77.88	602287	bb			Unknown						1024.50			

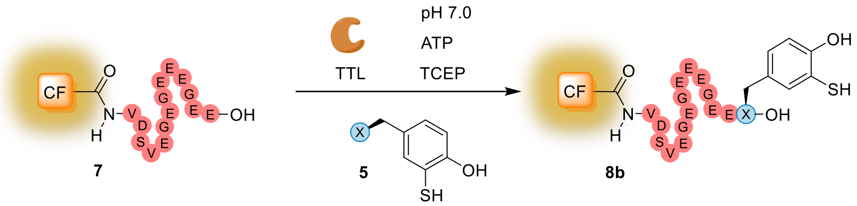

Relative signal quantification – incorporation of 5 – 300 / 420 min (Representative dataset)

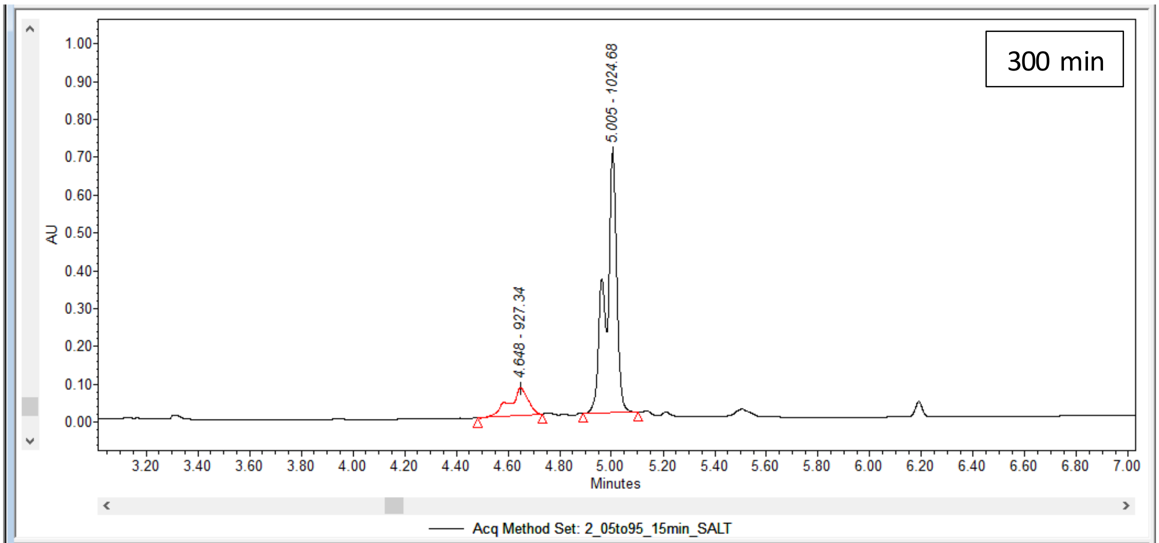

	Name	Migration Time (min)	Area (µV*sec)	% Area	Height (µV)	Int Type	Amount	Units	Peak Type	Peak Codes	Corrected Area~ (µV*sec)	MS Match1 Score	MS Match2 Score	MS Match3 Score	Base Peak (Combined) (m/z)	Assigned Mass	Assigned Mass Value (m/z)	Notes
1		4.648	365868	15.19	72812	bb			Unknown						926.82			
2		5.005	2043531	84.81	686526	bb			Unknown						1024.54			

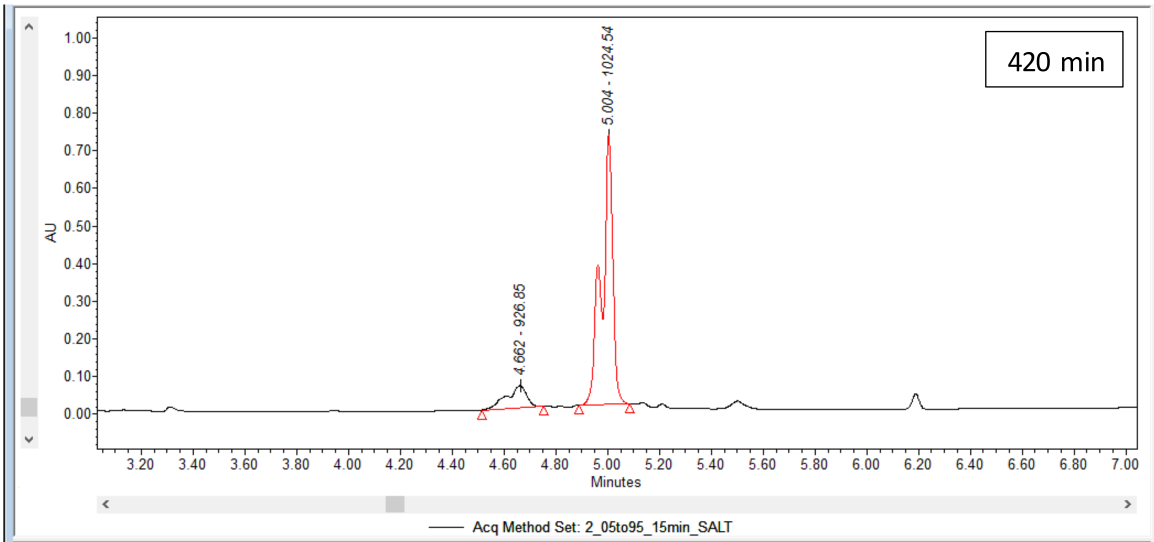

	Name	Migration Time (min)	Area (µV*sec)	% Area	Height (µV)	Int Type	Amount	Units	Peak Type	Peak Codes	Corrected Area~ (µV*sec)	MS Match1 Score	MS Match2 Score	MS Match3 Score	Base Peak (Combined) (m/z)	Assigned Mass	Assigned Mass Value (m/z)	Notes
1		4.662	310371	12.71	58637	bb			Unknown						591.38			
2		5.004	2131950	87.29	715497	bb			Unknown						1024.52			

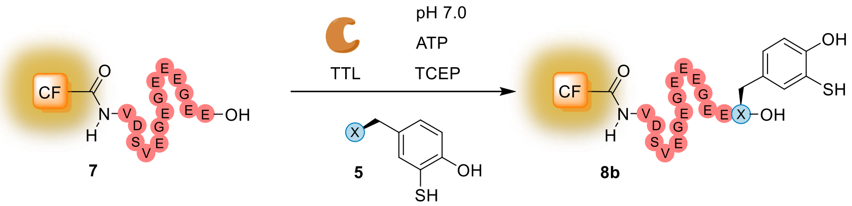

Relative signal quantification – incorporation of 5 – 24 h
(Representative dataset)

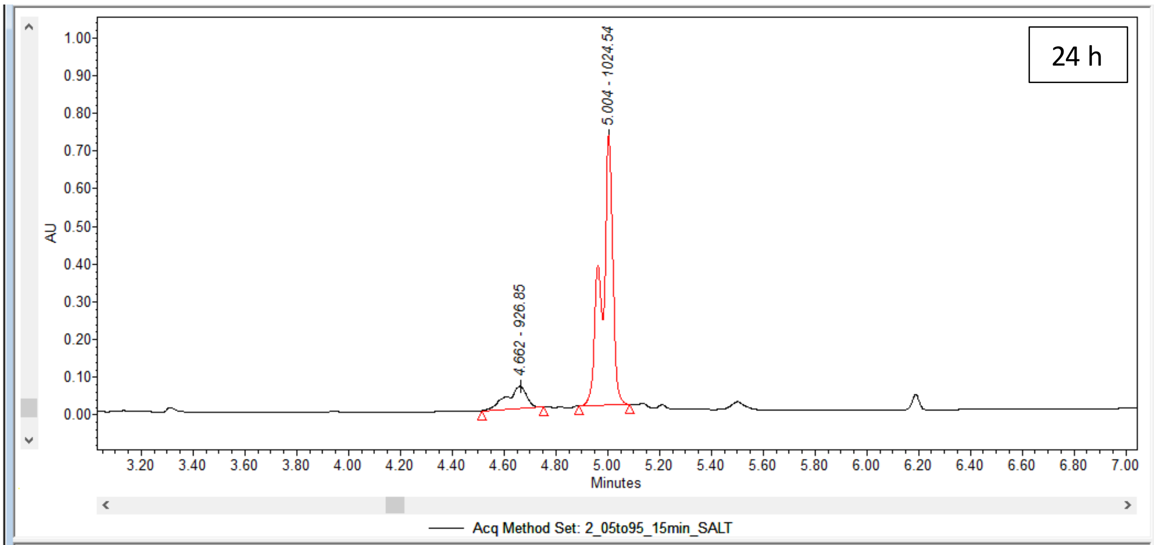

	Name	Migration Time (min)	Area (µV*sec)	% Area	Height (µV)	Int Type	Amount	Units	Peak Type	Peak Codes	Corrected Area~ (µV*sec)	MS Match1 Score	MS Match2 Score	MS Match3 Score	Base Peak (Combined) (m/z)	Assigned Mass	Assigned Mass Value (m/z)	Notes
1		4.662	310371	12.71	58637	bb			Unknown						591.38			
2		5.004	2131950	87.29	715497	bb			Unknown						1024.52			

Quantification of chemo-enzymatic reaction (Figure S22)

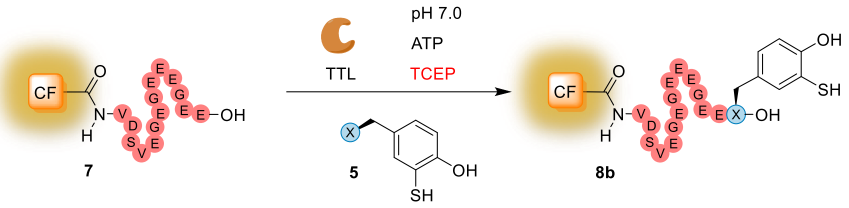

Relative signal quantification – incorporation of 5 – 5 hours
(Representative dataset)

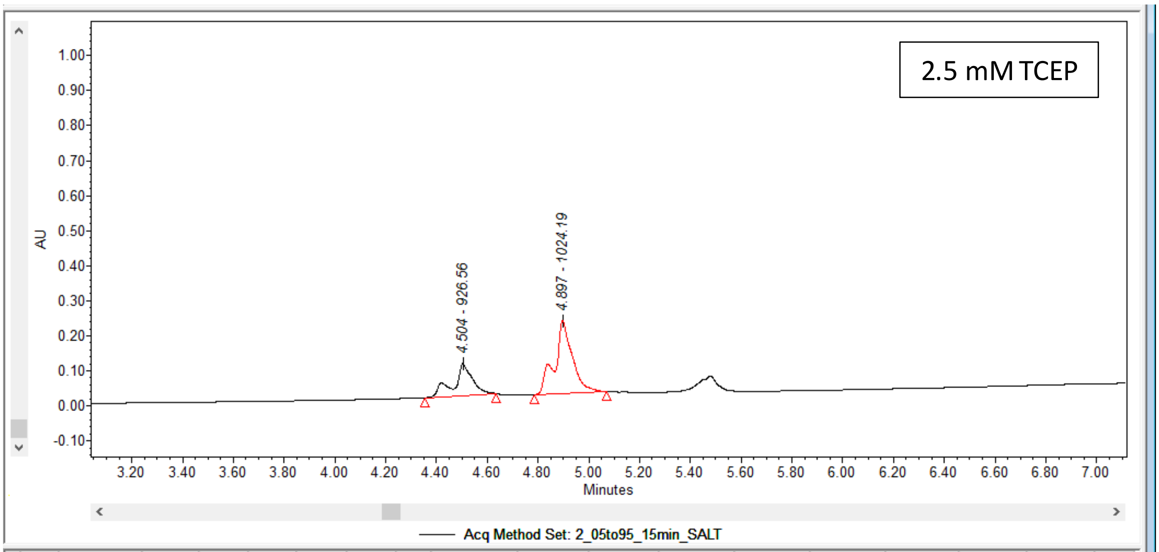

	Name	Migration Time (min)	Area (µV*sec)	% Area	Height (µV)	Int Type	Amount	Units	Peak Type	Peak Codes	Corrected Area~ (µV*sec)	MS Match1 Score	MS Match2 Score	MS Match3 Score	Base Peak (Combined) (m/z)	Assigned Mass	Assigned Mass Value (m/z)	Notes
1		4.504	469766	31.78	92019	bb			Unknown						926.40			
2		4.897	1008532	68.22	208760	bb			Unknown						1024.18			

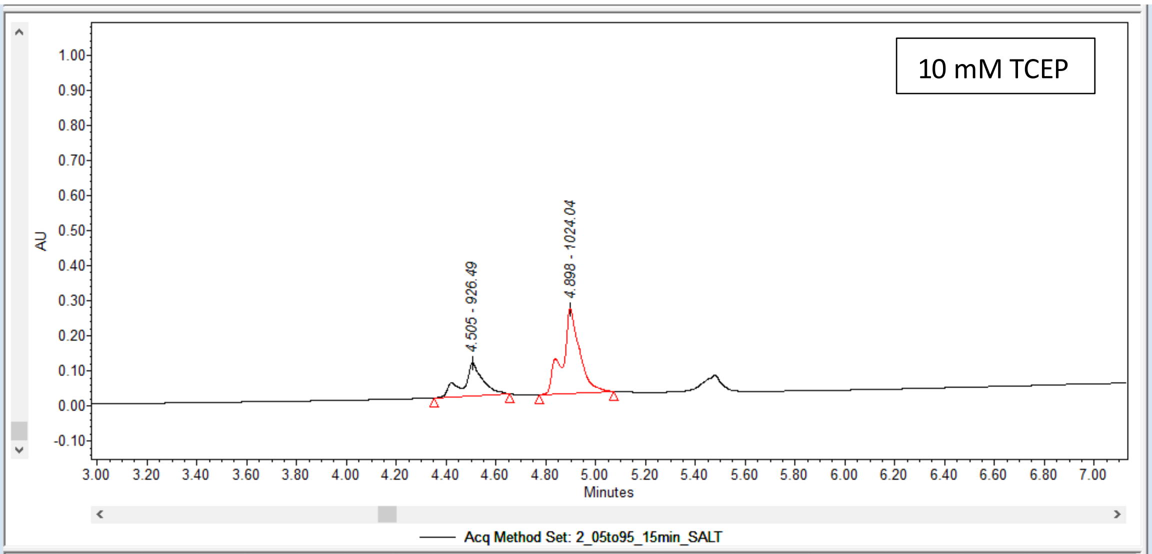

	Name	Migration Time (min)	Area (µV*sec)	% Area	Height (µV)	Int Type	Amount	Units	Peak Type	Peak Codes	Corrected Area~ (µV*sec)	MS Match1 Score	MS Match2 Score	MS Match3 Score	Base Peak (Combined) (m/z)	Assigned Mass	Assigned Mass Value (m/z)	Notes
1		4.505	515274	30.55	94982	bb			Unknown						926.44			
2		4.898	1171393	69.45	240598	bb			Unknown						1024.15			

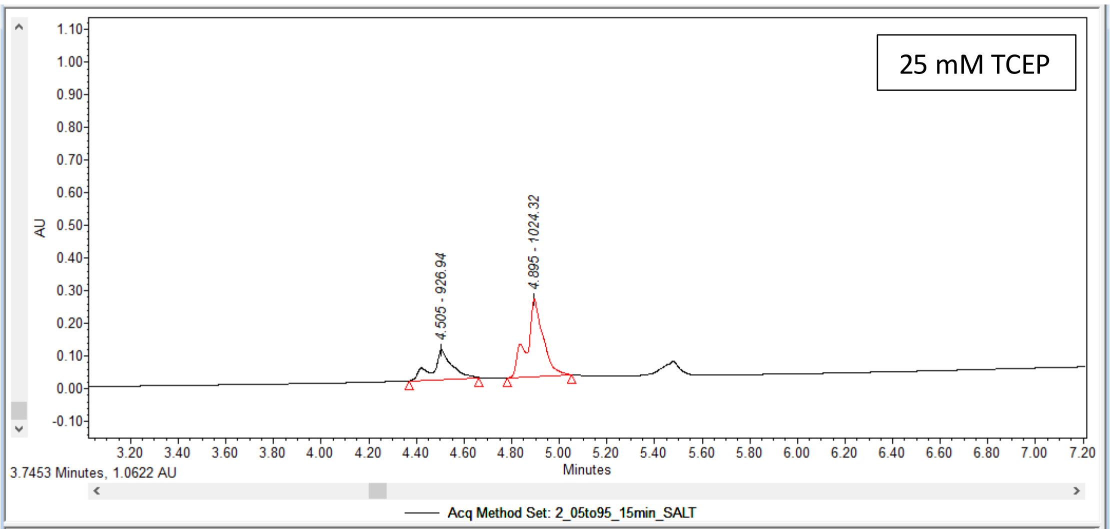

	Name	Migration Time (min)	Area (μV*sec)	% Area	Height (μV)	Int Type	Amount	Units	Peak Type	Peak Codes	Corrected Area~ (μV*sec)	MS Match1 Score	MS Match2 Score	MS Match3 Score	Base Peak (Combined) (m/z)	Assigned Mass	Assigned Mass Value (m/z)	Notes
1		4.505	497643	30.34	90439	bb			Unknown						926.47			
2		4.895	1142619	69.66	237113	bb			Unknown						1024.20			

7.3 Construction of pET22- pelB-His10-GBP1-C-Tub-tag® plasmid

pET22- pelB-His10-GBP1-C-Tub-tag® recombinant expression plasmid was constructed using Q5® Site-Directed Mutagenesis Kit (New England Biolabs) according to the manufacturer's specifications. Briefly, the following were combined in a 0.2 mL PCR tube: 25 ng of purified pET22-pelB-His10-GBP1-Tub-tag® plasmid,[5,6] 0.5 µM of forward primer (5'-TGGAGCGGTGGATAGCGTGGAAGGC-3') and reverse primer (5'-CATCCCGCTGAGGAGACGGTGACCTG-3'), 1X Q5 Hot Start High-Fidelity Master Mix, and nuclease-free water (final volume = 25 µL). The plasmid was amplified and linearized by polymerase chain reactions using the following cycling conditions: Initial denaturation (98 °C, 30 s), [Denaturation (98 °C, 10 s), Annealing (60 °C, 30 s), Extension (72 °C, 2.5 min) for 25 cycles], Final extension (72 °C, 2 min), Hold (4 °C). Ligation was performed by combining 1 µL of the PCR product, KLD reaction buffer (supplied), KLD enzyme mix (supplied), and nuclease-free water (final volume = 10 µL), and incubating the reaction mix at room temperature (~25 °C) for 10 min. The constructed plasmid was transformed into DH5α *E. coli* by heat shock, plated onto Luria-Bertani (LB) with carbenicillin (LB-Carb), and incubated at 37 °C for 14 h. Colonies were propagated in 5 mL LB broth with carbenicillin overnight at 37 °C. Plasmids were purified using GeneJET Plasmid Miniprep Kit (Thermo), concentrations were measured by NanoDrop, and the sequence was confirmed by standard DNA sequencing. Below is the sequence of the constructed plasmid:

tggcgaatgggacgcgccctgtagcggcgcattaagcgcggcgggtgtggtggttacgcgcagcgtgaccgctacacttgccagcgccctagcg
cccgctcctttcgctttcttcccttcctttctcgccacgttcgccggctttccccgtcaagctctaaatcgggggctccctttagggttccgatttagtgc
tttacggcacctcgaccccaaaaaacttgattagggtgatggttcacgtagtgggccatcgccctgatagacggtttttcgccctttgacgttggag
tccacgttctttaatagtggactcttgttccaaactggaacaacactcaaccctatctcggtctattcttttgatttataagggattttgccgatttcgg
cctattggttaaaaaatgagctgatttaacaaaaatttaacgcgaattttaacaaaatattaacgtttacaatttcaggtggcacttttcggggaaa
tgtgcgcggaacccctatttgtttatttttctaaatacattcaaatatgtatccgctcatgagacaataaccctgataaatgcttcaataatattgaa
aaaggaagagtatgagtattcaacatttccgtgtcgcccttattcccttttttgcggcattttgccttcctgtttttgctcacccagaaacgctggtga
aagtaaaagatgctgaagatcagttgggtgcacgagtgggttacatcgaactggatctcaacagcggtaagatccttgagagttttcgccccgaa
gaacgttttccaatgatgagcacttttaaagttctgctatgtggcgcggtattatcccgtattgacgccgggcaagagcaactcggtcgccgcatac
actattctcagaatgacttggttgagtactcaccagtcacagaaaagcatcttacggatggcatgacagtaagagaattatgcagtgctgccata
accatgagtgataacactgcggccaacttacttctgacaacgatcggaggaccgaaggagctaaccgcttttttgcacaacatgggggatcatgt
aactcgccttgatcgttgggaaccggagctgaatgaagccataccaaacgacgagcgtgacaccacgatgcctgcagcaatggcaacaacgttg
cgcaaactattaactggcgaactacttactctagcttcccggcaacaattaatagactggatggaggcggataaagttgcaggaccacttctgcg
ctcggcccttccggctggctggtttattgctgataaatctggagccggtgagcgtgggtctcgcggtatcattgcagcactggggccagatggtaa
gccctcccgtatcgtagttatctacacgacggggagtcaggcaactatggatgaacgaaatagacagatcgctgagataggtgcctcactgatta
agcattggtaactgtcagaccaagtttactcatatatactttagattgatttaaaacttcatttttaatttaaaaggatctaggtgaagatcctttttg
ataatctcatgaccaaaatcccttaacgtgagttttcgttccactgagcgtcagaccccgtagaaaagatcaaaggatcttcttgagatcctttttt
ctgcgcgtaatctgctgcttgcaaacaaaaaaaccaccgctaccagcggtggtttgtttgccggatcaagagctaccaactctttttccgaaggta
actggcttcagcagagcgcagataccaaatactgtccttctagtgtagccgtagttaggccaccacttcaagaactctgtagcaccgcctacatac
ctcgctctgctaatcctgttaccagtggctgctgccagtggcgataagtcgtgtcttaccgggttggactcaagacgatagttaccggataaggcgc
agcggtcgggctgaacggggggttcgtgcacacagcccagcttggagcgaacgacctacaccgaactgagatacctacagcgtgagctatgag
aaagcgccacgcttcccgaagggagaaaggcggacaggtatccggtaagcggcagggtcggaacaggagagcgcacgagggagcttccagg
gggaaacgcctggtatctttatagtcctgtcgggtttcgccacctctgacttgagcgtcgatttttgtgatgctcgtcaggggggcggagcctatgg
aaaaacgccagcaacgcggcctttttacggttcctggccttttgctggccttttgctcacatgttctttcctgcgttatcccctgattctgtggataacc
gtattaccgcctttgagtgagctgataccgctcgccgcagccgaacgaccgagcgcagcgagtcagtgagcgaggaagcggaagagcgcctga
tgcggtattttctccttacgcatctgtgcggtatttcacaccgcatatatggtgcactctcagtacaatctgctctgatgccgcatagttaagccagta
tacactccgctatcgctacgtgactgggtcatggctgcgccccgacacccgccaacacccgctgacgcgccctgacgggcttgtctgctcccggca

tccgcttacagacaagctgtgaccgtctccgggagctgcatgtgtcagaggttttcaccgtcatcaccgaaacgcgcgaggcagctgcggtaaag
ctcatcagcgtggtcgtgaagcgattcacagatgtctgcctgttcatccgcgtccagctcgttgagtttctccagaagcgttaatgtctggcttctga
taaagcgggccatgttaagggcggttttttcctgtttggtcactgatgcctccgtgtaagggggatttctgttcatgggggtaatgataccgatgaa
acgagagaggatgctcacgatacgggttactgatgatgaacatgcccggttactggaacgttgtgagggtaaacaactggcggtatggatgcgg
cgggaccagagaaaaatcactcagggtcaatgccagcgcttcgttaatacagatgtaggtgttccacagggtagccagcagcatcctgcgatgc
agatccggaacataatggtgcagggcgctgacttccgcgtttccagactttacgaaacacggaaaccgaagaccattcatgttgttgctcaggtc
gcagacgttttgcagcagcagtcgcttcacgttcgctcgcgtatcggtgattcattctgctaaccagtaaggcaaccccgccagcctagccgggtc
ctcaacgacaggagcacgatcatgcgcacccgtggggccgccatgccggcgataatggcctgcttctcgccgaaacgtttggtggcgggaccag
tgacgaaggcttgagcgagggcgtgcaagattccgaataccgcaagcgacaggccgatcatcgtcgcgctccagcgaaagcggtcctcgccga
aaatgacccagagcgctgccggcacctgtcctacgagttgcatgataaagaagacagtcataagtgcggcgacgatagtcatgccccgcgccca
ccggaaggagctgactgggttgaaggctctcaagggcatcggtcgagatcccggtgcctaatgagtgagctaacttacattaattgcgttgcgctc
actgcccgctttccagtcgggaaacctgtcgtgccagctgcattaatgaatcggccaacgcgcggggagaggcggtttgcgtattgggcgccagg
gtggtttttcttttcaccagtgagacgggcaacagctgattgcccttcaccgcctggccctgagagagttgcagcaagcggtccacgctggtttgcc
ccagcaggcgaaaatcctgtttgatggtggttaacggcgggatataacatgagctgtcttcggtatcgtcgtatcccactaccgagatatccgcac
caacgcgcagcccggactcggtaatggcgcgcattgcgcccagcgccatctgatcgttggcaaccagcatcgcagtgggaacgatgccctcattc
agcatttgcatggtttgttgaaaaccggacatggcactccagtcgccttcccgttccgctatcggctgaatttgattgcgagtgagatatttatgcca
gccagccagacgcagacgcgccgagacagaacttaatgggcccgctaacagcgcgatttgctggtgacccaatgcgaccagatgctccacgccc
agtcgcgtaccgtcttcatgggagaaaataatactgttgatgggtgtctggtcagagacatcaagaaataacgccggaacattagtgcaggcag
cttccacagcaatggcatcctggtcatccagcggatagttaatgatcagcccactgacgcgttgcgcgagaagattgtgcaccgccgctttacagg
cttcgacgccgcttcgttctaccatcgacaccaccacgctggcacccagttgatcggcgcgagatttaatcgccgcgacaatttgcgacggcgcgt
gcagggccagactggaggtggcaacgccaatcagcaacgactgtttgcccgccagttgttgtgccacgcggttgggaatgtaattcagctccgcc
atcgccgcttccactttttcccgcgttttcgcagaaacgtggctggcctggttcaccacgcgggaaacggtctgataagagacaccggcatactct
gcgacatcgtataacgttactggtttcacattcaccaccctgaattgactctcttccgggcgctatcatgccataccgcgaaaggttttgcgccattc
gatggtgtccgggatctcgacgctctcccttatgcgactcctgcattaggaagcagcccagtagtaggttgaggccgttgagcaccgccgccgcaa
ggaatggtgcatgcaaggagatggcgcccaacagtcccccggccacggggcctgccaccatacccacgccgaaacaagcgctcatgagcccga
agtggcgagcccgatcttccccatcggtgatgtcggcgatataggcgccagcaaccgcacctgtggcgccggtgatgccggccacgatgcgtccg
gcgtagaggatcgagatctcgatcccgcgaaattaatacgactcactataggggaattgtgagcggataacaattcccctctagaaataattttgt
ttaactttaagaaggagatatacatatgaaatacctgctgccgaccgctgctgctggtctgctgctcctcgctgcccagccggcgatggccatggc
ccatcatcaccatcaccatcatcaccatcatgatgtgcagctggtggagtctgggggagccttggtgcagccgggggggtctctgagactctcctg
tgcagcctctggattccccgtcaatcgctatagtatgaggtggtaccgccaggctccagggaaggagcgcgagtgggtcgcgggtatgagtagtg
ctggtgatcgttcaagttatgaagactccgtgaagggccgattcaccatctccagagacgacgccaggaatacggtgtatctgcaaatgaacagc
ctgaaacctgaggacacggccgtgtattactgtaatgtcaatgtgggctttgagtactggggccaggggacccaggtcaccgtctcctcagcggg
atgtggagcggtggatagcgtggaaggcgaaggcgaagaagaaggcgaagaataagaattcgagctccgtcgacaagcttgcggccgcactc
gagcaccaccaccaccaccactgagatccggctgctaacaaagcccgaaaggaagctgagttggctgctgccaccgctgagcaataactagcat
aaccccttggggcctctaaacgggtcttgaggggttttttgctgaaaggaggaactatatccggat

7.4 Expression and purification of GBP1-C-Tub-tag® (26)

BL21(DE3) *E. coli* were transformed with the constructed pET22- pelB-His10-GBP1-C-Tub-tag® by heat-shock, followed by inoculation onto LB-Carb agar plates and overnight incubation at 37°C. Starter cultures were made by inoculating single colonies into 5 mL of LB-carb broth and further incubation at 37°C and 180 rpm. Starter cultures were added into 500 mL of LB-carb broth and incubated at 37°C with 180 rpm shaking until the optical density at 600 nm (OD_{600}) reached 0.6. The bacterial culture was cooled in the cold room for 30 min before the induction of expression by adding isopropyl ß-D-1-thiogalactopyranoside (IPTG) to a final concentration of 1 mM and incubation at 18°C with 180 rpm shaking overnight. Induced cells were collected by centrifugation (4000 *g*, 4°C, 15 min) and stored at −20°C until purification.

The pellet of cells was defrosted on ice, washed once with PBS, resuspended in PBS with 10 mM imidazole and lysed using sonication (2×, 2 min, 30% amplitude), followed by debris centrifugation (25000 *g*, 4°C, 30 min). Before the purification of **26**, dithiothreitol (DTT) was added to the clarified lysates (final concentration of 2 mM), followed by loading onto 5 mL of PureCube 100 Ni-INDIGO agarose (Cube Biotech) pre-equilibrated with PBS with 10 mM imidazole. The beads were washed with 20 column volumes of PBS with 10 mM imidazole. The protein was then eluted using 15 mL of PBS with 500 mM imidazole. Elution fractions were combined and concentrated by ultrafiltration (5 kDa MWCO) to 500 µL. GBP1-C-Tub-tag® (**26**) was further purified by size exclusion chromatography (SEC; NGC Chromatography System, BioRad)) using a Superdex™ 75 16/60 column (GE Healthcare) in **TTL buffer** (20 mM 3-(*N*-morpholino)propanesulfonic acid (MOPS) pH 7.0, 100 mM KCl, 10 mM $MgCl_2$, 10% (v/v) propane-1,2-diol). Peak fractions were pooled, concentrated to around 170 µM, aliquoted, shock-frozen and stored at −70°C.

GBP1-C-Tub-tag® (**26**) was characterized by 12% SDS-PAGE (left) and intact protein MS (right).

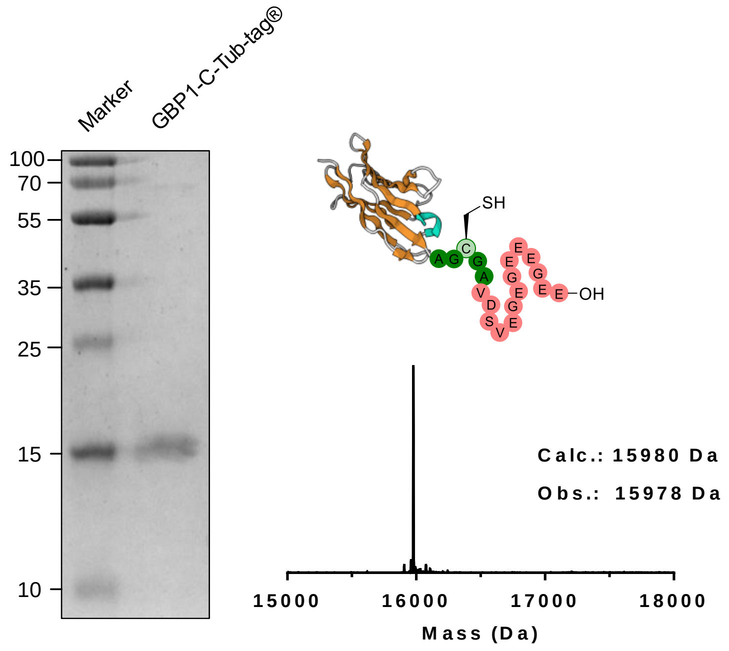

7.5 Bioconjugation experiments with GBP1-C-Tub-tag® (26)

1) *Chemoenzymatic tyrosination [26 → 27]*

Tyrosination reactions were performed in a 130 µL solution (Eppendorf Protein LoBind® tubes) using the following conditions: 100 µM GBP1-C-Tub-tag® (**26**), 6 µM SUMO-TTL (6.0 mol%), 1.25 mM aryl disulfide **6**, 2.5 mM TCEP, 5 mM ATP in **TTL buffer** (20 mM 3-(*N*-morpholino)propanesulfonic acid (MOPS) pH 7.0, 100 mM KCl, 10 mM $MgCl_2$, 10% (v/v) propane-1,2-diol) at 37°C, 850 rpm shaking for 4 hours.

<u>Important notes:</u>

- ATP stock solutions (25 mM in TTL buffer) were prepared from ATP · 2 Na and neutralized using 2 molar equivalents of KOH. TCEP stock solutions (100 mM in TTL buffer) were prepared by diluting commercially available 0.5 M aliquots (pH 7.0, adjusted with ammonium hydroxide) from Sigma Aldrich (Merck, USA) into the desired concentration with TTL buffer. Stock solutions for aryl disulfide **6** were prepared in TTL buffer (50 mM). Stock solutions were aliquoted, stored at −20°C and thawed before use.

- Stock solutions for the initial components (**6**, ATP and TCEP) and any additional TTL buffer required were added to the Eppendorf Protein LoBind® tubes and the mixture was homogenized.

- GBP1-C-Tub-tag® (**26**) stock solution aliquots (~ 170 µM in TTL buffer) were stored at −70°C (see section 7.4). Aliquots were thawed on ice, carefully mixed and added to the reaction mixture before homogenizing the reaction mixture.

- SUMO-TTL stock solution aliquots (20 - 25 µM in TTL buffer) were stored at −70°C (see section 7.1). Aliquots were thawed on ice, carefully mixed and directly added to the reaction mixture as the final component. After carefully homogenizing the reaction mixture, the tyrosination reaction was launched at 37°C, 850 rpm shaking for 4 hours.

<u>Analysis:</u>

A crude reaction mixture sample (1 µL) was drawn after 4 hours and diluted with PBS (100 µL). The sample was analyzed using intact protein MS (10 µL injection).

<u>Workup:</u>

The reaction mixture was rebuffered to 0.2 M citrate buffer pH 5.5 (Eppendorf Protein LoBind® tube) using Zeba® Spin desalting columns (0.5 mL, 7K MWCO; Thermo Fischer) according to the manufacturer's instruction.

2) *Aryl thiolate (3-SH-L-Tyr) – ETP modification [27 → 28 / 32]*

The rebuffered solution (pH 5.5) was treated with TCEP (300 µM final concentration; 3 equiv). The reaction mixture was carefully homogenized and incubated at 37°C, 850 rpm shaking for 30 minutes. Afterwards, the reaction mixture was briefly allowed to cool down to room temperature before adding Biotin-ETP **9** or Cy5-ETP **29** (120 µM or 145 µM, respectively; 1.2 or 1.45 equiv; ≤ 1% DMSO). The reaction mixture was carefully homogenized and incubated at 25°C, 850 rpm shaking for 16-20 hours.

Important notes:
- The procedure (TCEP reduction for 30 min followed by ETP reagent addition) was initiated directly after rebuffering reaction mixture from step 1 to pH 5.5.

- TCEP stock solutions (50 mM in 0.2 M citrate buffer pH 5.5) were prepared by diluting commercially available 0.5 M aliquots (pH 7.0, adjusted with ammonium hydroxide) from Sigma Aldrich (Merck, USA) into the desired concentration with 0.2 M citrate buffer pH 5.5. Stock solutions for Biotin-ETP **9** or Cy5-ETP **29** were prepared in DMSO (20 mM). Stock solutions were aliquoted, stored at −20°C and thawed before use.

Analysis:
A crude reaction mixture sample (1 µL) was drawn after 16-20 hours and diluted with PBS (100 µL). The sample was analyzed using intact protein MS (10 µL injection).

Workup:
The reaction mixture was rebuffered to PBS or 50 mM Tris buffer (1 mM EDTA, 100 mM NaCl) pH 8.3 using Zeba® Spin desalting columns (0.5 mL, 7K MWCO; Thermo Fischer) according to the manufacturer's instruction.

3) *Alkyl thiolate (Cys) – ETP modification [32 → 33]*

The rebuffered solution (pH 8.3) was treated with Trolox-ETP **31** (150 µM; 1.5 equiv; ≤ 1% DMSO). The reaction mixture was carefully homogenized and incubated at 25°C, 850 rpm shaking for 16-20 hours.

Important notes:
- The procedure (ETP reagent addition) was initiated directly after rebuffering the reaction mixture from step 2 to pH 8.3.

- Stock solutions for Trolox-ETP **31** were prepared in DMSO (20 mM). Stock solutions were aliquoted, stored at −20°C and thawed before use.

Analysis:
A crude reaction mixture sample (1 µL) was drawn after 16-20 hours and diluted with PBS (100 µL). The sample was analyzed using intact protein MS (10 µL injection).

Workup:
The reaction mixture was rebuffered to PBS using Zeba® Spin desalting columns (0.5 mL, 7K MWCO; Thermo Fischer) according to the manufacturer's instruction.

Intact protein MS: Raw and Deconvoluted Spectra – Table S1

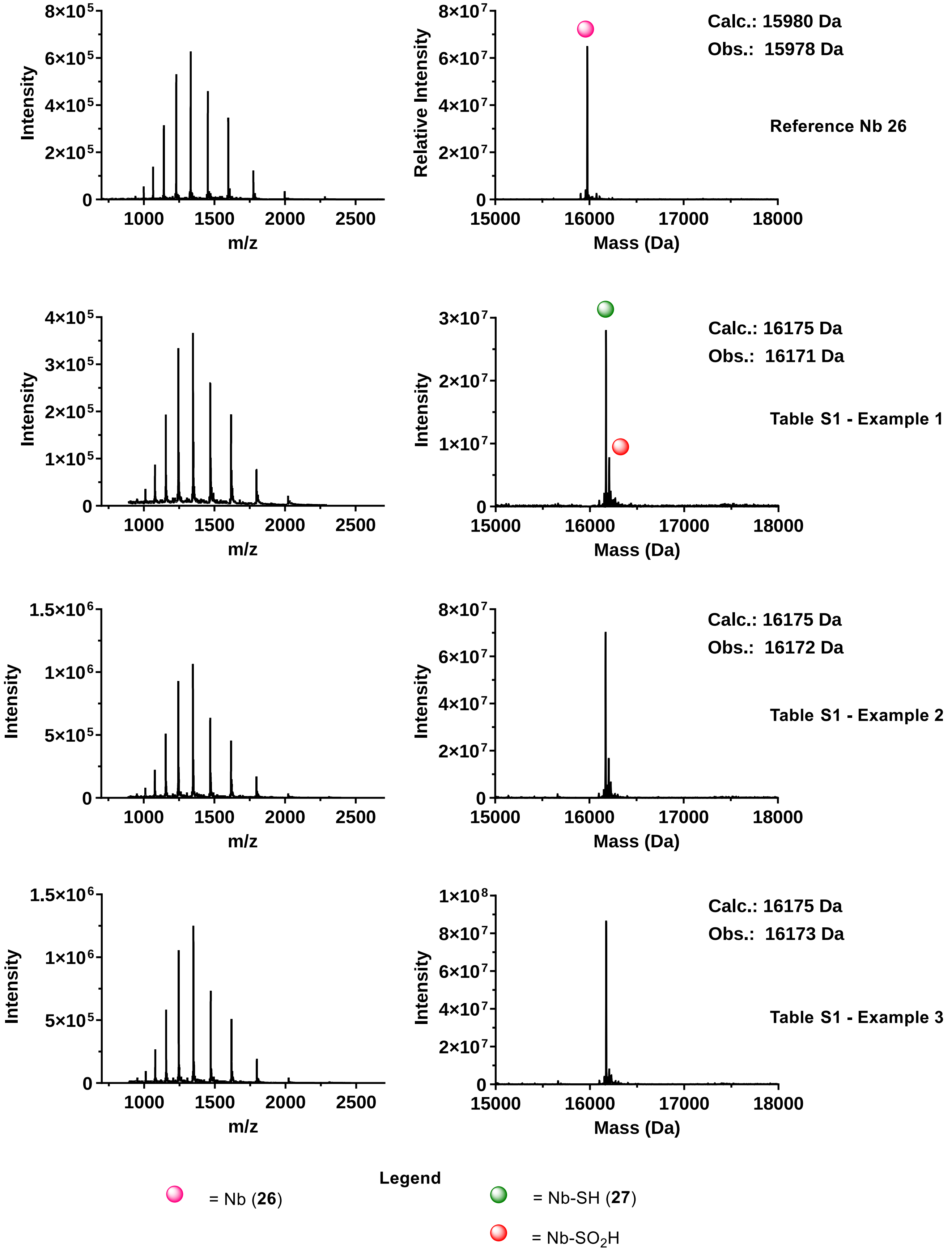

Intact protein MS: Raw and Deconvoluted Spectra – Table S2

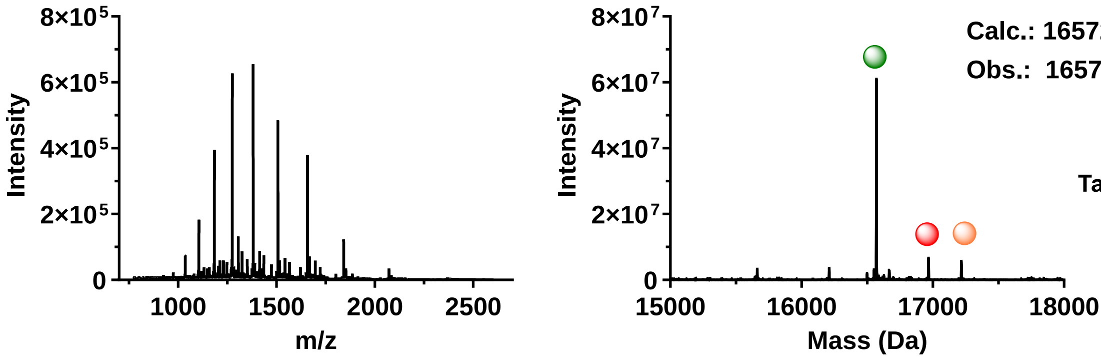

Calc.: 16572 Da
Obs.: 16571 Da

Table S2 - Entry 1

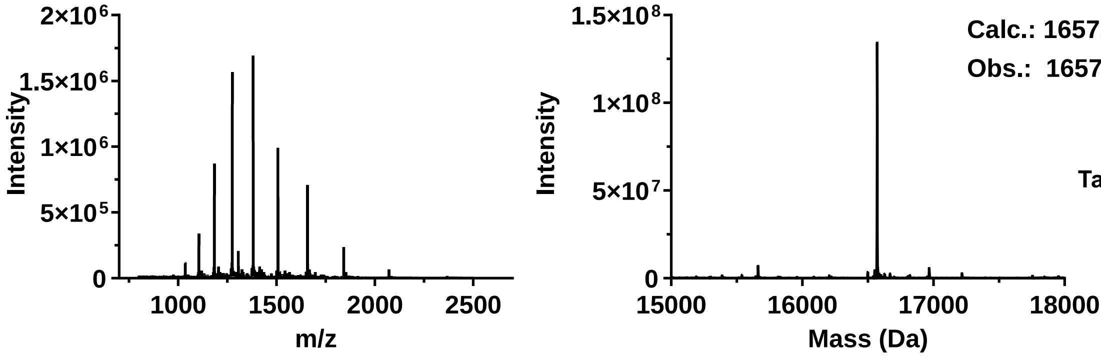

Calc.: 16572 Da
Obs.: 16571 Da

Table S2 - Entry 2

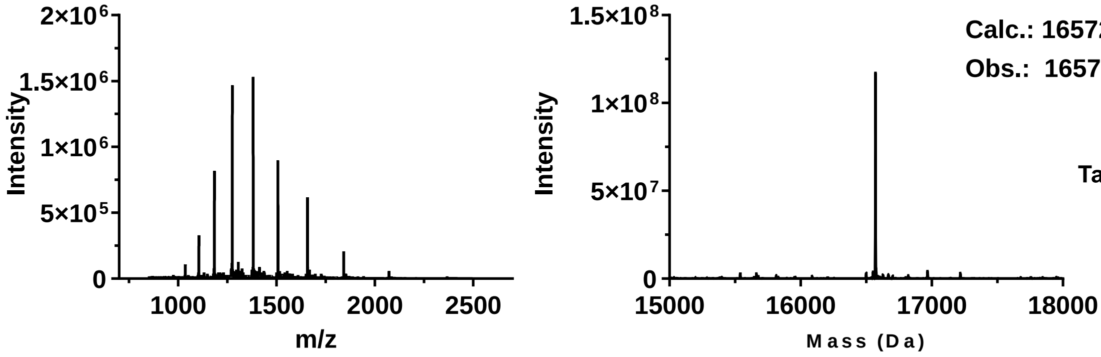

Calc.: 16572 Da
Obs.: 16571 Da

Table S2 - Entry 3

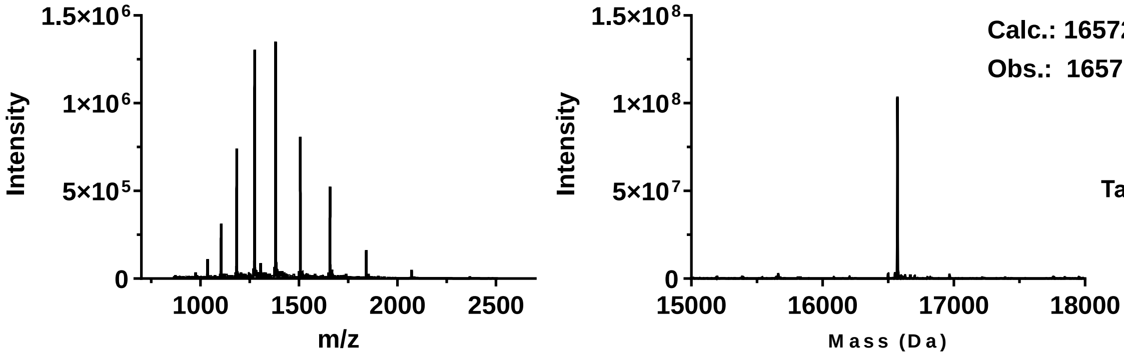

Calc.: 16572 Da
Obs.: 16571 Da

Table S2 - Entry 4

Legend

= Nb-S-Biotin (**28**)

= Nb-S-Biotin (**28**) + **9**

= Nb-S-Biotin (**28**) + **9** + TCEP

Intact protein MS: Raw and Deconvoluted Spectra – Table S3

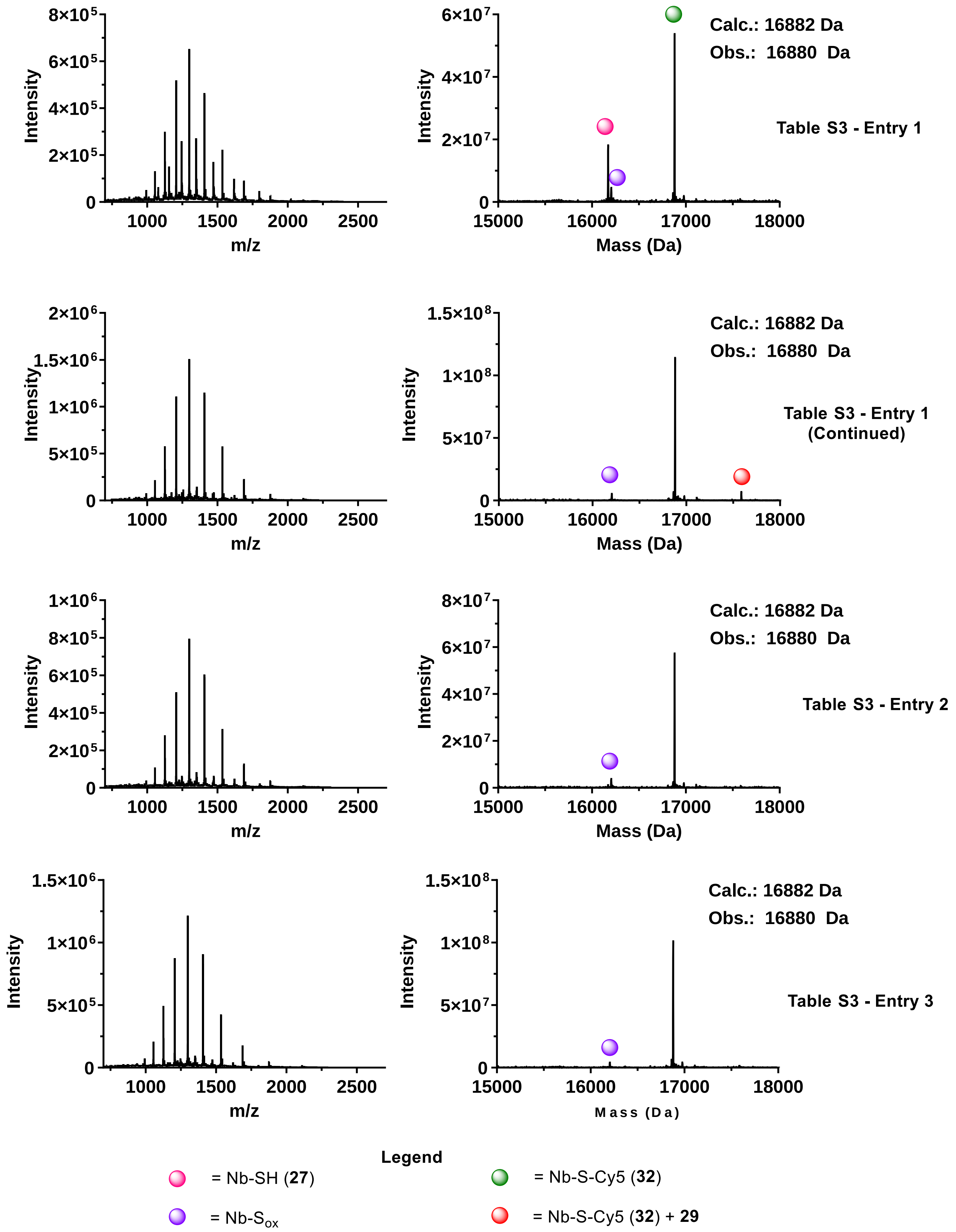

Intact protein MS: Raw and Deconvoluted Spectra – Table S4

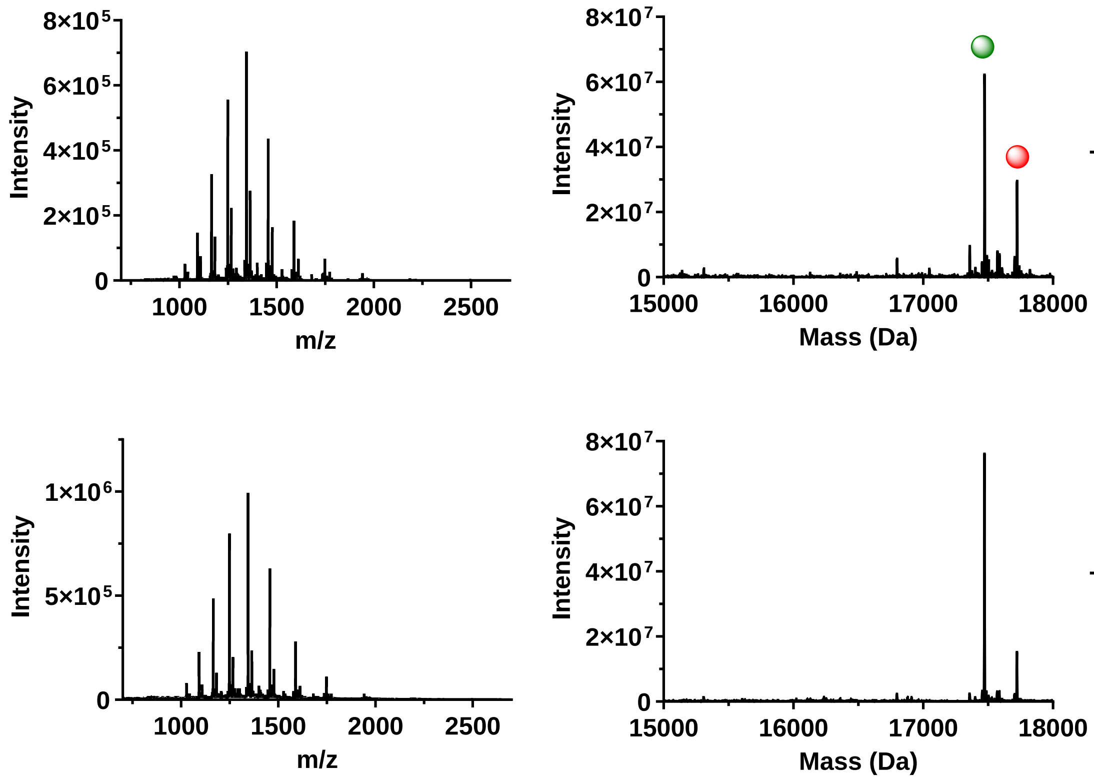

Calc.: 17474 Da
Obs.: 17473 Da

Table S4 - Entry 1

Calc.: 17474 Da
Obs.: 17472 Da

Table S4 - Entry 2

Legend

(green) = Nb-S-Cy5-Trolox (**33**)

(red) = Nb-S-Cy5-Trolox (**33**) + TCEP

7.6 Validation of selectivity for aryl thiolate – ETP reaction

Upon formation of GBP1-C-Tub-tag®-STyr(Biotin) **28**, the sample was reduced (10 mM TCEP, 56°C, 30 min), rebuffered to Tris-buffer (pH 8.5) containing 10 mM iodoacetamide and incubated for 30 minutes at room temperature. Subsequently, the protein was diluted to a concentration of 1 mg/mL and digested with trypsin (Promega, 1:20, w/w) at 37°C overnight. The next day, an aliquot of the trypsinized peptide-mixture was additionally treated with Chymotrypsin (Roche, 1:20, w/w) for 2 h at 37°C. Peptides were desalted using Stage Tips and stored at −20°C until measurement. Before MS/MS analysis a quality-control run was conducted on a Waters XEVO G2-XS QTof instrument to ensure complete digestion.

LC-MS/MS analysis was performed using an UltiMate 3000 RSLC nano LC system coupled on-line to an Orbitrap Fusion mass spectrometer (Thermo Fisher Scientific). For sample loading a PepMap C-18 trap-column (Thermo Fischer Scientific) of 0.075 mm ID x 50 mm length, 3 µm particle size and 100 Å pore size was used. The loading mobile phase A contained 1% acetonitrile and 0.05% TFA in water, and mobile phase B 0.05% TFA in acetonitrile. Reversed-phase separation was performed using a 50 cm analytical column (in-house packed with Poroshell 120 EC-C18, 2.7µm, Agilent Technologies) with mobile phase A contained 0.1% formic acid in water, and mobile phase B 0.1% formic acid in acetonitrile using a 60 minutes gradient. Data was acquired using survey scans in a range of 375 to 1500 m/z with a resolution of 120k and an AGC target value of 4e5. Precursor ions with charge states 2-8 were isolated with a mass selecting quadrupole (isolation window 1.6 m/z) with 10 sec dynamic exclusion (+/- 10 ppm). Precursor ions were fragmented using higher-energy collisional dissociation (HCD) applying a normalized collision energy (NCE) of 30. The maximum injection time was set to 22 ms to collect 5e4 precursor ions. Fragment ion spectra were acquired in the Orbitrap with a resolution of 15k (FWHM).

The obtained raw-data was analyzed using FragPipe (v21) using the built-in open-search workflow.[16] The following MSFragger[17] settings were applied: Precursor mass tolerance: -50 to +700 Da; Fragment mass tolerance: +/-20 ppm; Mass calibration & parameter optimization enabled; Isotope error: 0; Enzyme: Unspecific, Peptide length: 7-50 AA; Peptide mass range: 500-5000 Da; Variable modifications: Oxidation (M, +15.9949 Da, up to 3x), Acetylation (*N*-term, +42.0106 Da); no fixed modification. Validation was performed using Crystal-C,[18] and PeptideProphet (using default settings). A protein-level FDR of 5% was applied. Additionally, PTM-Sheperd[19] was enabled (using the default settings). Peptides were quantified using IonQuant.[20] The best scoring PSM containing the desired modification-pattern was manually validates and blotted using interactive spectrum annotator.[21]

7.7 Fluorescence bleaching experiments

HeLa-Kyoto cells stably expressing a fusion protein of histone 2B-green fluorescent protein (HeLa-H2B-GFP) were seeded into 8-well glass bottom slides (IBIDI) and allowed to attach for 48 h. Cell culture media were discarded and the cells were fixed with 4% paraformaldehyde for 15 min at 37 °C, followed by thrice washing with PBS. Fixed cells were permeabilized with 0.2% Triton-X solution for 5 min at room temperature, washed thrice with PBS, and blocked with 5% FCS in PBS solution for 30 min at room temperature. The cells were immunostained with 1.25 µg/mL of **32** or **33** in 5% FCS in PBS overnight at 4°C with constant rocking motion. The treated cells were washed thrice with PBS (5 min each washing) and 200 µL PBS was added before imaging.

Imaging was performed using a Nikon-CSU spinning disk microscope with a CSU-X1 (Andor) confocal scanner unit using a Plan Apo 40× NA 0.95 air objective (Nikon) and an EMCCD (AU888, Andor). Standard laser, a quad Dicroic (633-647, AHF), and emission filters were used in the acquisition of confocal fluorescence images [GFP (H2B-GFP): λex = 488 nm, λem = 525/50 nm; iRFP (Cy5): λex = 640 nm, λem = 685/50 nm]. For the bleaching experiment, the samples were imaged every 5 s for 15 min at iRFP laser power of 100% with 100 ms exposure time.

Images were processed using FIJI (ver. 2.1.0-1).[22] For **Figure 4C**, the wide-field images were captured with iRFP laser power of 5%, while the regions-of-interest (ROIs), indicated by a white dashed box, were bleached according to the above protocol. Images also indicate the image number among 180 captured images. The images were set at the same contrast. For **Figure S23A**, a total of 45 nuclei were bleached according to the above protocol. The corresponding Cy5 fluorescence intensities (arbitrary unit, a.u.) of each nucleus for each image were measured and averaged, and graphed using GraphPad Prism 10.3.1. Each point in the graph shows the mean fluorescence intensity of 45 nuclei ± standard deviation (SD) for the indicated image number. For **Figure S23B**, the fluorescence data in **Figure S23A** was normalized to the maximum value obtained for each conjugate. The x-axis was converted from image number to seconds, which enabled determination of the half-life during photobleaching (t1/2) using one phase decay.

One phase decay of normalized fluorescence intensity to determine half-life (Figure S23B)

Table of results

	Nonlin fit<br>Table of results	A<br>**32** (Cy5)	B<br>**33** (Trolox + Cy5)
1	**One phase decay**		
2	**Best-fit values**		
3	Y0	115.5	117.1
4	Plateau	0.3952	0.3209
5	K	0.03219	0.02643
6	Half Life	21.53	26.23
7	Tau	31.06	37.84
8	Span	115.1	116.7
9	**95% CI (profile likelihood)**		
10	Y0	115.1 to 115.9	116.6 to 117.5
11	Plateau	0.3626 to 0.4278	0.2819 to 0.3600
12	K	0.03204 to 0.03235	0.02630 to 0.02655
13	Half Life	21.43 to 21.63	26.10 to 26.35
14	Tau	30.91 to 31.21	37.66 to 38.02
15	**Goodness of Fit**		
16	Degrees of Freedom	7197	7197
17	R squared	0.9899	0.9889
18	Sum of Squares	12367	17176
19	Sy.x	1.311	1.545
20	**Constraints**		
21	K	K > 0	K > 0
22			
23	**Number of points**		
24	# of X values	9000	9000
25	# Y values analyzed	7200	7200

7.8 Bioconjugation experiments with Brentuximab-LC-Tub-tag® (34)

1) Chemoenzymatic tyrosination [34 → 38]

Tyrosination reactions were performed in a 130 µL solution (Eppendorf Protein LoBind® tubes) using the following conditions: 40 µM Brentuximab-LC-Tub-tag® (**34**), 12.5 µM SUMO-TTL (31.3 mol%), 1.25 mM aryl disulfide **6**, 2.5 mM TCEP, 5 mM ATP in **TTL buffer** (20 mM 3-(*N*-morpholino)propanesulfonic acid (MOPS) pH 7.0, 100 mM KCl, 10 mM $MgCl_2$, 10% (v/v) propane-1,2-diol) at 37°C, 850 rpm shaking for 4 hours.

<u>Important notes:</u>
- ATP stock solutions (25 mM in TTL buffer) were prepared from ATP · 2 Na and neutralized using 2 molar equivalents of KOH. TCEP stock solutions (100 mM in TTL buffer) were prepared by diluting commercially available 0.5 M aliquots (pH 7.0, adjusted with ammonium hydroxide) from Sigma Aldrich (Merck, USA) into the desired concentration with TTL buffer. Stock solutions for aryl disulfide **6** were prepared in TTL buffer (50 mM). Stock solutions were aliquoted, stored at −20°C and thawed before use.

- Stock solutions for the initial components (**6**, ATP and TCEP) and any additional TTL buffer / propane-1,2-diol required were added to the Eppendorf Protein LoBind® tubes and the mixture was homogenized.

-Brentuximab-LC-Tub-tag® (**34**) was used as received by Tubulis GmbH.[23] Stock solution aliquots (130 µM in TTL buffer without propane-1,2-diol) were stored at −70°C. Aliquots were thawed on ice, carefully mixed and added to the reaction mixture before homogenizing the reaction mixture.

- SUMO-TTL stock solution aliquots (22.5 - 30 µM in TTL buffer) were stored at −70°C (see section 7.1). Aliquots were thawed on ice, carefully mixed and directly added to the reaction mixture as the final component. After carefully homogenizing the reaction mixture, the tyrosination reaction was launched at 37°C, 850 rpm shaking for 4 hours.

<u>Analysis:</u>
A crude reaction mixture sample (1 µL) was drawn after 4 hours and diluted with PBS (180 µL) and TCEP (50 mM in PBS, 20 µL) in an Eppendorf tube. The sample was incubated at 37°C, 850 rpm shaking for ≥ 15 – 30 minutes and subsequently transferred to an MS vial for analysis using intact protein MS (3 µL injection).

<u>Workup:</u>
The reaction mixture was rebuffered (2× sequentially) to 0.2 M citrate buffer pH 5.5 (Eppendorf Protein LoBind® tube) using Zeba® Spin desalting columns (0.5 mL, 7K MWCO; Thermo Fischer) according to the manufacturer's instruction.

2) Aryl thiolate (3-SH-L-Tyr) – ETP modification [38 → 39]

The rebuffered solution (pH 5.5) was treated with TCEP (750 µM final concentration; 18.75 equiv). The reaction mixture was carefully homogenized and incubated at 37°C, 850 rpm shaking for 30 minutes. Afterwards, the reaction mixture was briefly allowed to cool down to room temperature before adding ETP-PEG$_3$-Val-Cit-PAB-MMAF **36** (240 µM final concentration, 6.0 equiv; ≤ 2.5% DMSO). The reaction mixture was carefully homogenized and incubated at 25°C, 850 rpm shaking for 16-20 hours.

Important notes:
- The procedure (TCEP reduction for 30 min followed by ETP reagent addition) was initiated directly after rebuffering reaction mixture from step 1 to pH 5.5.

- TCEP stock solutions (100 mM in 0.2 M citrate buffer pH 5.5) were prepared by diluting commercially available 0.5 M aliquots (pH 7.0, adjusted with ammonium hydroxide) from Sigma Aldrich (Merck, USA) into the desired concentration with 0.2 M citrate buffer pH 5.5. Stock solutions for ETP-PEG$_3$-Val-Cit-PAB-MMAF **36** were prepared in DMSO (10 mM). Stock solutions were aliquoted, stored at −20°C and thawed before use.

Analysis:
A crude reaction mixture sample (1 µL) was drawn after 16-20 hours and diluted with PBS (180 µL) and TCEP (50 mM in PBS, 20 µL) in an Eppendorf tube. The sample was incubated at 37°C, 850 rpm shaking for ≥ 15 – 30 minutes and subsequently transferred to an MS vial for analysis using intact protein MS (3 µL injection).

Workup:
The reaction mixture was rebuffered to 50 mM Tris buffer (1 mM EDTA, 100 mM NaCl) pH 8.3 using Zeba® Spin desalting columns (0.5 mL, 7K MWCO; Thermo Fischer) according to the manufacturer's instruction.

(Alternative) Purification:
Alternatively, the reaction mixture was purified by size-exclusion chromatography with a 25 mL Superose™ 6 Increase 10/300GL (GE Healthcare, United States) under a flow rate of 0.5 mL/min using sterile PBS (Merck, Germany) as the mobile phase. Antibody containing fractions were pooled and the final concentration was determined (see 4.8).
The purified sample (± 0.3 mg/mL) was analyzed using intact protein MS (as described before, but diluting 20 µL of purified sample with PBS (160 µL) and TCEP (50 mM in PBS, 20 µL)), analytical HIC (see 4.9) and analytical SEC (see 4.10). The remainder of purified sample (2000 µL total volume) was aliquoted, shock-frozen and stored at −70°C.

3) Alkyl thiolate (Cys) – P5 modification [39 → 40]

The rebuffered solution (pH 8.3) was treated with TCEP (480 µM final concentration; 12.0 equiv). The reaction mixture was carefully homogenized and incubated at 37°C, 850 rpm shaking for 30 minutes. Afterwards, the reaction mixture was briefly allowed to cool down to room temperature before adding P5(PEG_{12})-Val-Cit-PAB-MMAE **37** (640 µM final concentration, 16.0 equiv; ≤ 2.0% DMSO). The reaction mixture was carefully homogenized and incubated at 25°C, 850 rpm shaking for 16-20 hours.

Important notes:
- The procedure (TCEP reduction for 30 min followed by P5 reagent addition) was initiated directly after rebuffering the reaction mixture from step 2 to pH 8.3.

- TCEP stock solutions (40 mM in 50 mM Tris buffer (1 mM EDTA, 100 mM NaCl) pH 8.3) were prepared by diluting commercially available 0.5 M aliquots (pH 7.0, adjusted with ammonium hydroxide) from Sigma Aldrich (Merck, USA) into the desired concentration with 50 mM Tris buffer (1 mM EDTA, 100 mM NaCl) pH 8.3. Stock solutions for P5(PEG_{12})-Val-Cit-PAB-MMAE **37** were prepared in DMSO (40 mM). Stock solutions were aliquoted, stored at −20°C and thawed before use.

Analysis:
A crude reaction mixture sample (1 µL) was drawn after 16-20 hours and diluted with PBS (180 µL) and TCEP (50 mM in PBS, 20 µL) in an Eppendorf tube. The sample was incubated at 37°C, 850 rpm shaking for ≥ 15 – 30 minutes and subsequently transferred to an MS vial for analysis using intact protein MS (3 µL injection).

Purification:
The reaction mixture was purified by size-exclusion chromatography with a 25 mL Superose™ 6 Increase 10/300GL (GE Healthcare, United States) under a flow rate of 0.5 mL/min using sterile PBS (Merck, Germany) as the mobile phase. Antibody containing fractions were pooled and the final concentration was determined (see 4.8).
The purified sample (± 0.3 mg/mL) was analyzed using intact protein MS (as described before, but diluting 20 µL of purified sample with PBS (160 µL) and TCEP (50 mM in PBS, 20 µL)), analytical HIC (see 4.9) and analytical SEC (see 4.10). The remainder of purified sample (2000 µL total volume) was aliquoted, shock-frozen and stored at −70°C.

***Control ADC:* Alkyl thiolate (Cys) – P5 modification [34→ 41]**

The P5 modification reaction was performed in an Eppendorf Protein LoBind® tube using the following conditions: 40 µM Brentuximab-LC-Tub-tag® (**34**), 400 µM TCEP, 640 µM P5(PEG_{12})-Val-Cit-PAB-MMAE (**37**) in 50 mM Tris buffer (1 mM EDTA, 100 mM NaCl) pH 8.3 at 37°C, 850 rpm shaking for 16-20 hours.

Important notes:
- TCEP stock solutions (40 mM in 50 mM Tris buffer (1 mM EDTA, 100 mM NaCl) pH 8.3) were prepared by diluting commercially available 0.5 M aliquots (pH 7.0, adjusted with ammonium hydroxide) from Sigma Aldrich (Merck, USA) into the desired concentration with 50 mM Tris buffer (1 mM EDTA, 100 mM NaCl) pH 8.3. Stock solutions for P5(PEG_{12})-Val-Cit-PAB-MMAE **37** were prepared in DMSO (40 mM). Stock solutions were aliquoted, stored at −20°C and thawed before use.

-Brentuximab-LC-Tub-tag® (**34**) was used as received by Tubulis GmbH.[23] Stock solution aliquots (130 µM in TTL buffer without propane-1,2-diol) were stored at −70°C. Aliquots were thawed on ice and carefully mixed.

- Brentuximab-LC-Tub-tag® **34** (40 µL) was diluted with 50 mM Tris buffer (1 mM EDTA, 100 mM NaCl) pH 8.3 (90 µL) and the resulting solution was rebuffered to 50 mM Tris buffer (1 mM EDTA, 100 mM NaCl) pH 8.3 using Zeba® Spin desalting columns (0.5 mL, 7K MWCO; Thermo Fischer) according to the manufacturer's instruction.

- The rebuffered solution (pH 8.3) was treated with TCEP (400 µM final concentration; 10.0 equiv). The reaction mixture was carefully homogenized and incubated at 37°C, 850 rpm shaking for 30 minutes. Afterwards, the reaction mixture was briefly allowed to cool down to room temperature before adding P5(PEG_{12})-Val-Cit-PAB-MMAE **37** (640 µM final concentration, 16.0 equiv; ≤ 2.0% DMSO). The reaction mixture was carefully homogenized and incubated at 25°C, 850 rpm shaking for 16-20 hours.

Analysis:
A crude reaction mixture sample (1 µL) was drawn after 16-20 hours and diluted with PBS (180 µL) and TCEP (50 mM in PBS, 20 µL) in an Eppendorf tube. The sample was incubated at 37°C, 850 rpm shaking for ≥ 15 – 30 minutes and subsequently transferred to an MS vial for analysis using intact protein MS (3 µL injection).

Purification:
The reaction mixture was purified by size-exclusion chromatography with a 25 mL Superose™ 6 Increase 10/300GL (GE Healthcare, United States) under a flow rate of 0.5 mL/min using sterile PBS (Merck, Germany) as the mobile phase. Antibody containing fractions were pooled and the final concentration was determined (see 4.8).
The purified sample (± 0.3 mg/mL) was analyzed using intact protein MS (as described before, but diluting 20 µL of purified sample with PBS (160 µL) and TCEP (50 mM in PBS, 20 µL)), analytical HIC (see 4.9) and analytical SEC (see 4.10). The remainder of purified sample (2000 µL total volume) was aliquoted, shock-frozen and stored at −70°C.

Intact protein MS acquisition details / data processing:

Acquisition of intact protein MS spectra for ADCs **39**, **40** and **41** resulted in fragmentation of the carbamate bond of payload **36** and/or **37**. To circumvent this, intact protein MS spectra for **39**, **40** and **41** were instead recorded without the usage of collision energy in the acquisition settings.

Processing of intact protein MS spectra for **38**, **39**, **40** and **41** was executed as described below. A similar procedure was used for nanobody conjugates **26**, **27**, **28**, **32** and **33** (see 7.5), with the omission of steps 2 and 3 and MaxEnt deconvolution in the range of 10000 - 20000 Da.

1) Integration of the chromatogram from 3.6 – 4.3 min (entire protein peak).
2) Background substraction of the m/z spectrum according to the parameters listed.

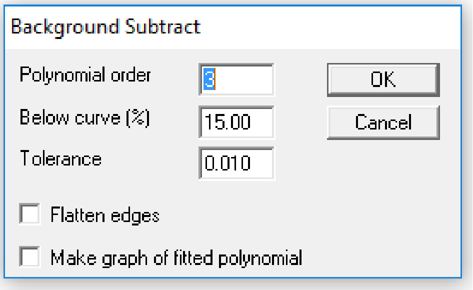

3) Signal smoothing of the m/z spectrum according to the parameters listed.

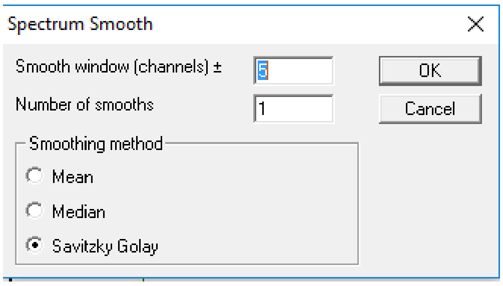

4) Selecting m/z range of ~ 900 – 2600.
5) Initiating MaxEnt calculation according to the parameters listed.

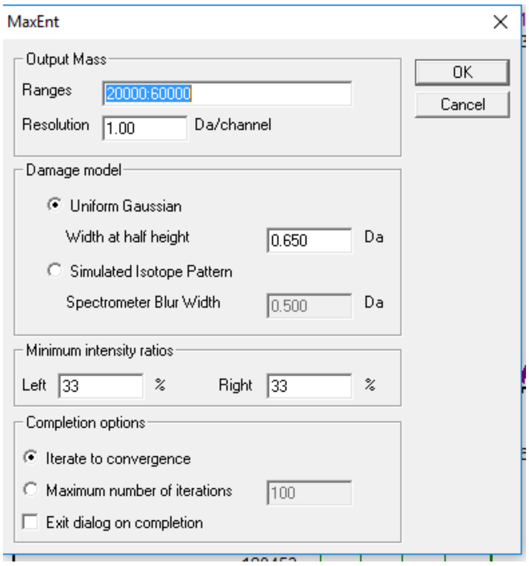

Intact protein MS: Raw and Deconvoluted Spectra – Table S5

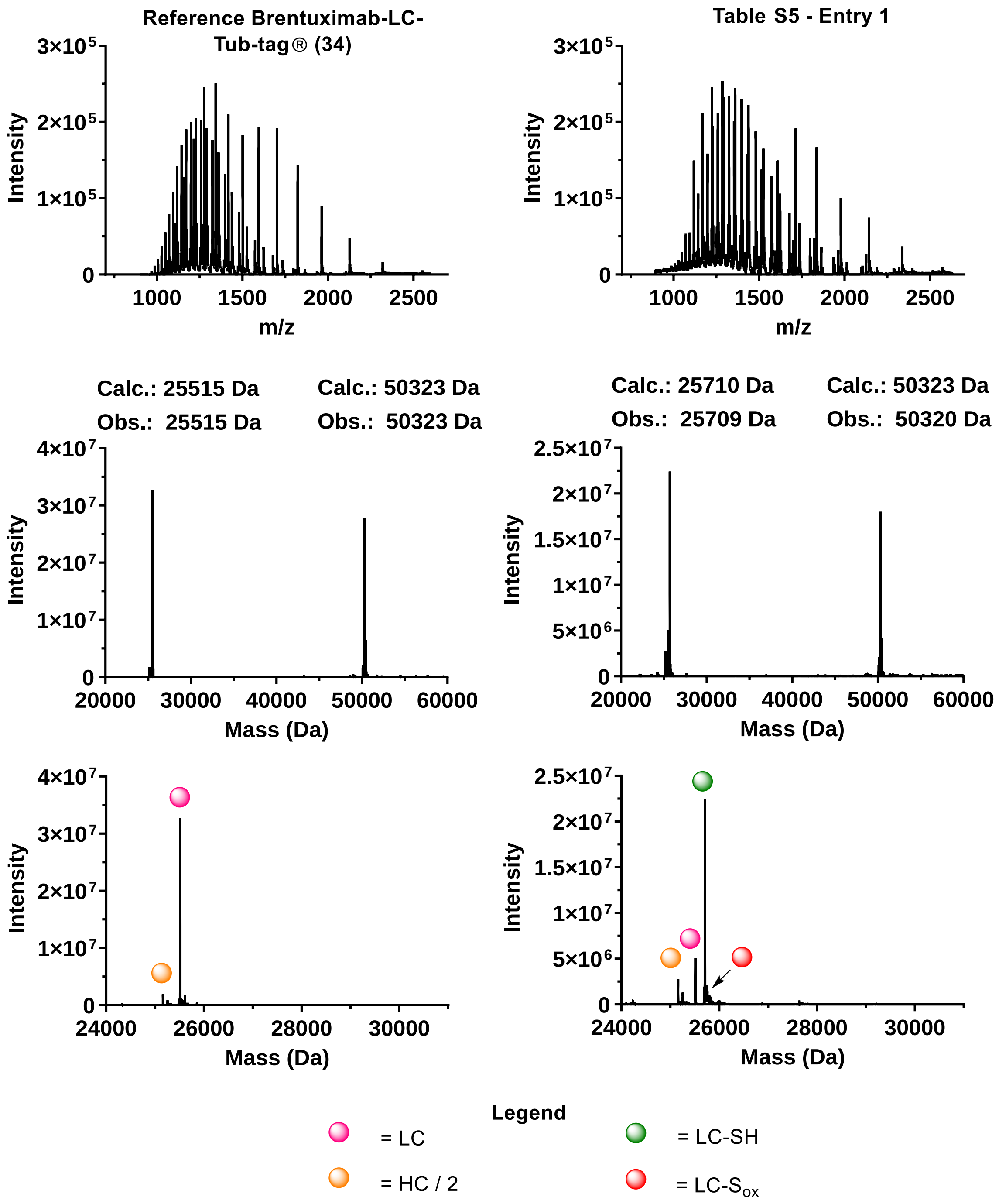

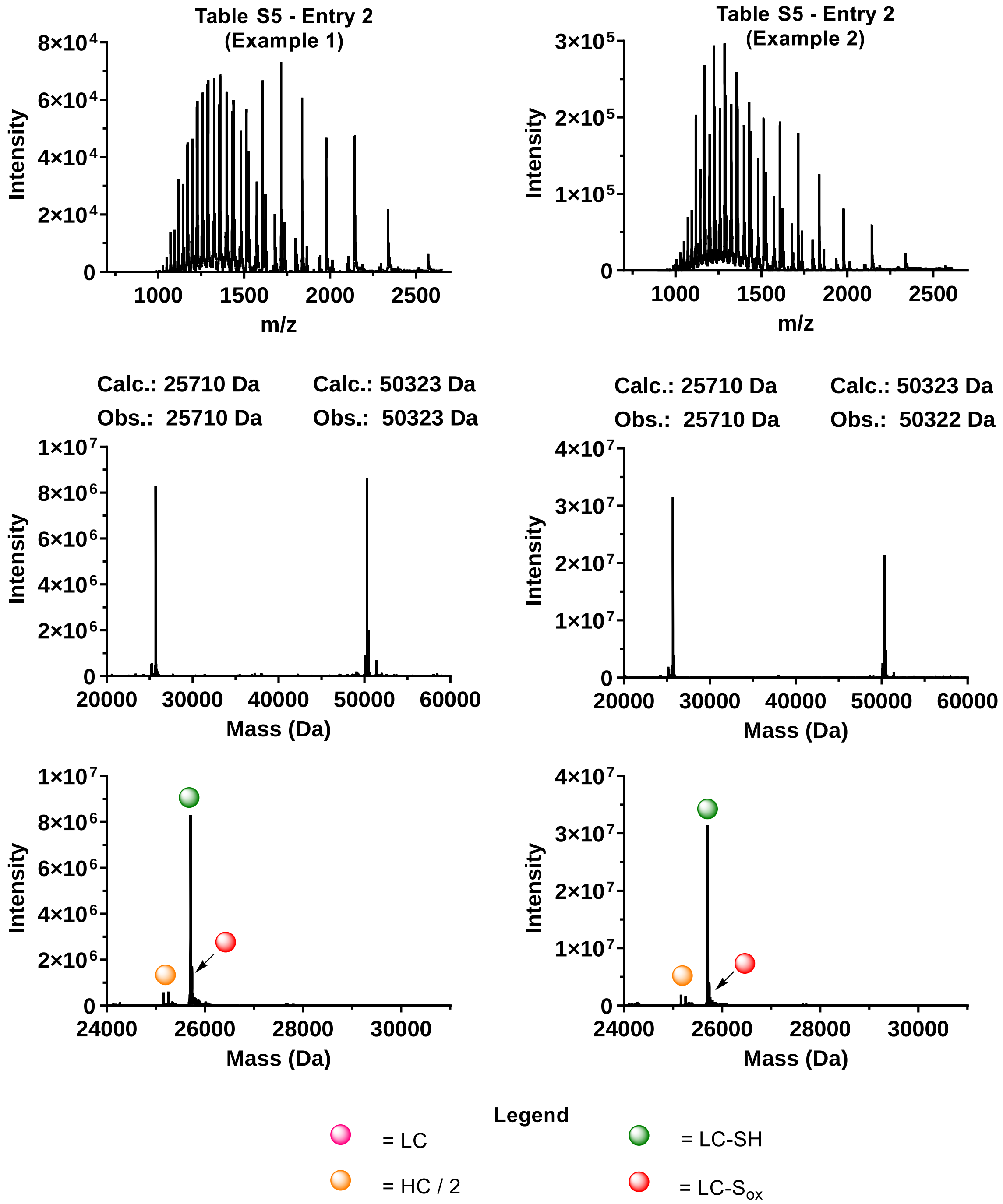

Table S5 - Entry 2
(Example 1)
Table S5 - Entry 2
(Example 2)
Intensity
m/z
8×10^4
6×10^4
4×10^4
2×10^4
0
3×10^5
2×10^5
1×10^5
1000
1500
2000
2500
Calc.: 25710 Da
Obs.: 25710 Da
Calc.: 50323 Da
Obs.: 50323 Da
Calc.: 25710 Da
Obs.: 25710 Da
Calc.: 50323 Da
Obs.: 50322 Da
1×10^7
8×10^6
6×10^6
4×10^6
2×10^6
4×10^7
3×10^7
2×10^7
1×10^7
20000
30000
40000
50000
60000
Mass (Da)
24000
26000
28000
30000
Legend
= LC
= HC / 2
= LC-SH
= LC-S$_{ox}$

Intact protein MS: Raw and Deconvoluted Spectra – Table S6

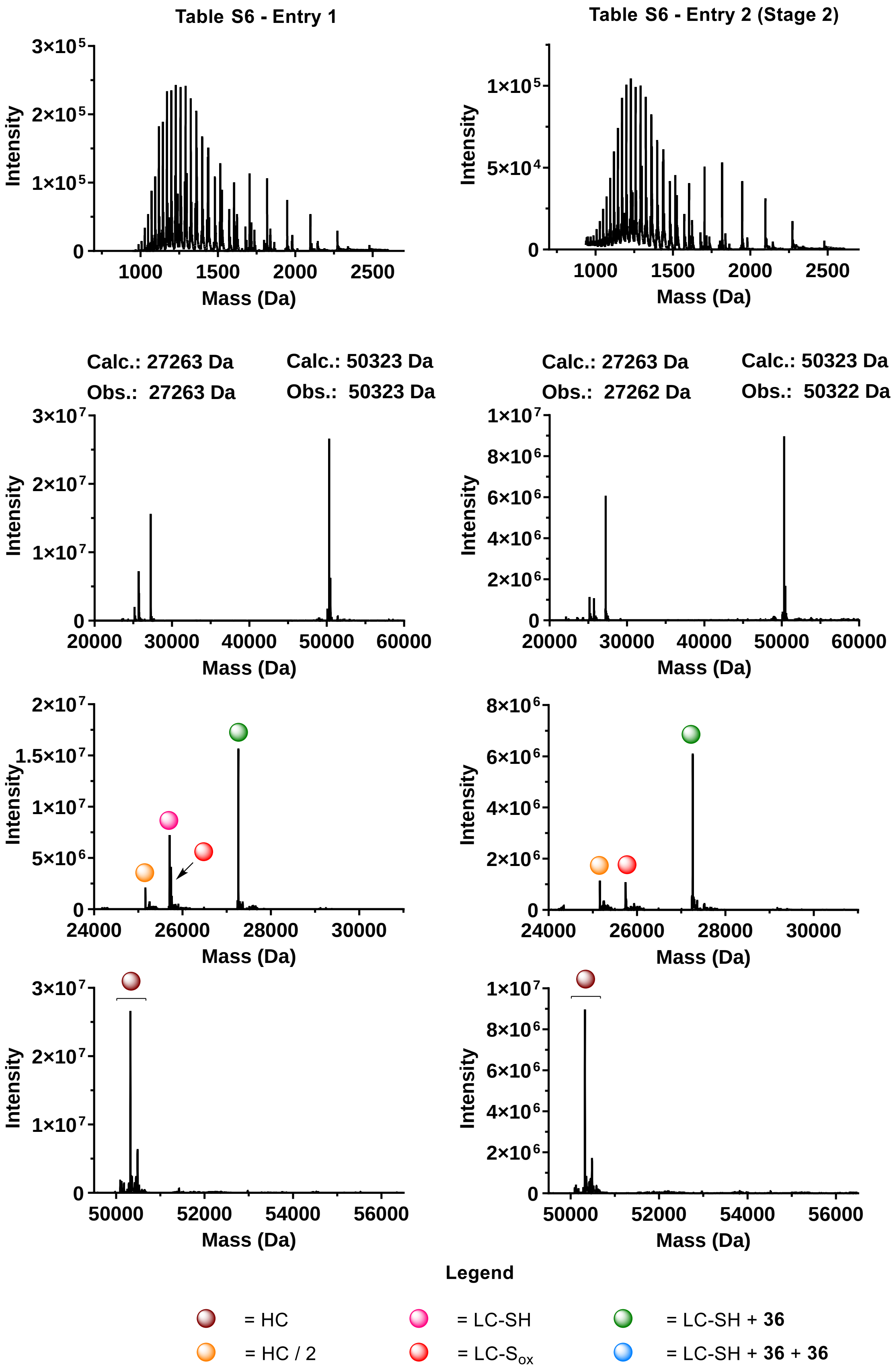

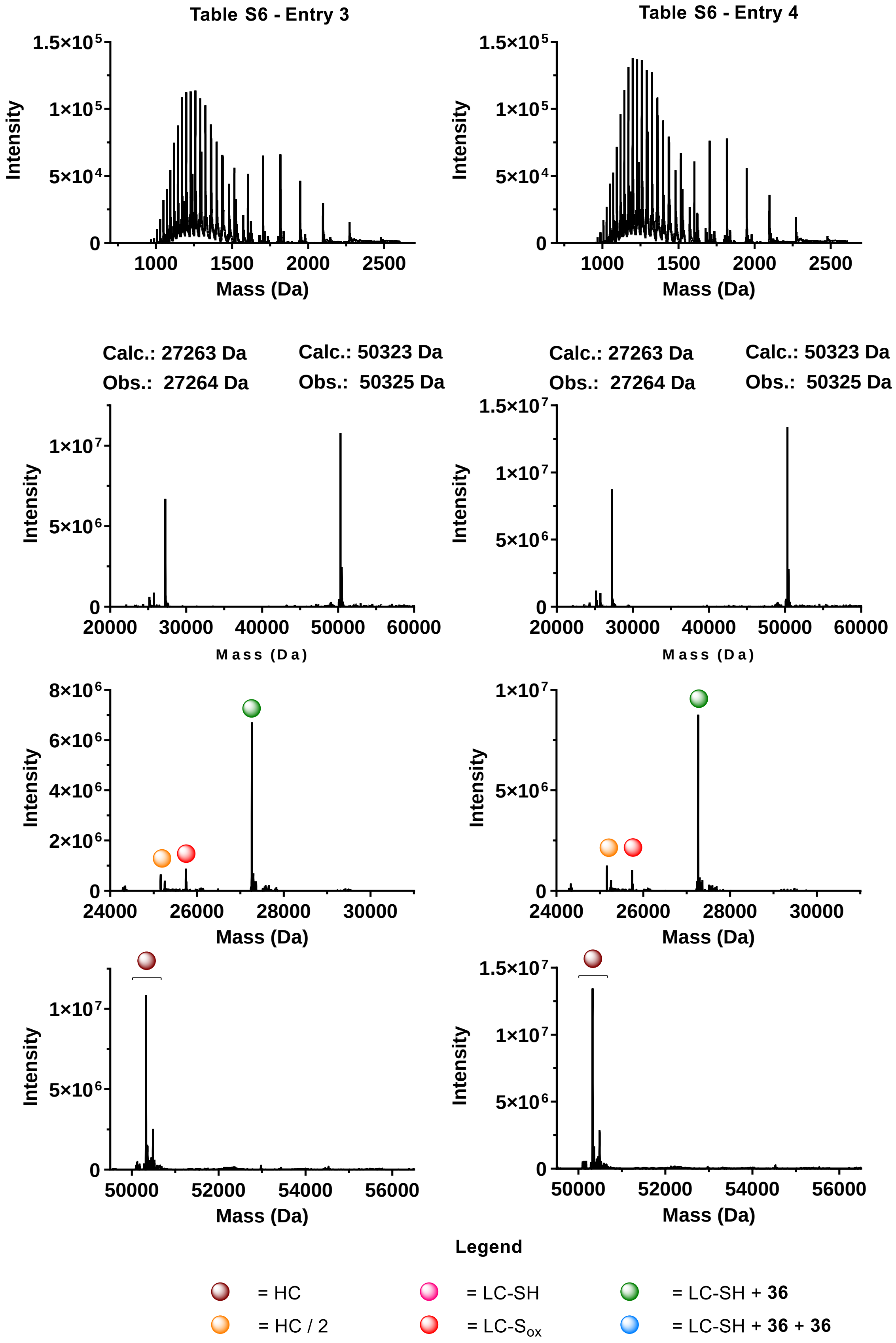
Table S6 - Entry 3
Table S6 - Entry 4
Intensity
Mass (Da)
Calc.: 27263 Da
Obs.: 27264 Da
Calc.: 50323 Da
Obs.: 50325 Da
Calc.: 27263 Da
Obs.: 27264 Da
Calc.: 50323 Da
Obs.: 50325 Da
Legend
= HC
= HC / 2
= LC-SH
= LC-S$_{ox}$
= LC-SH + **36**
= LC-SH + **36** + **36**

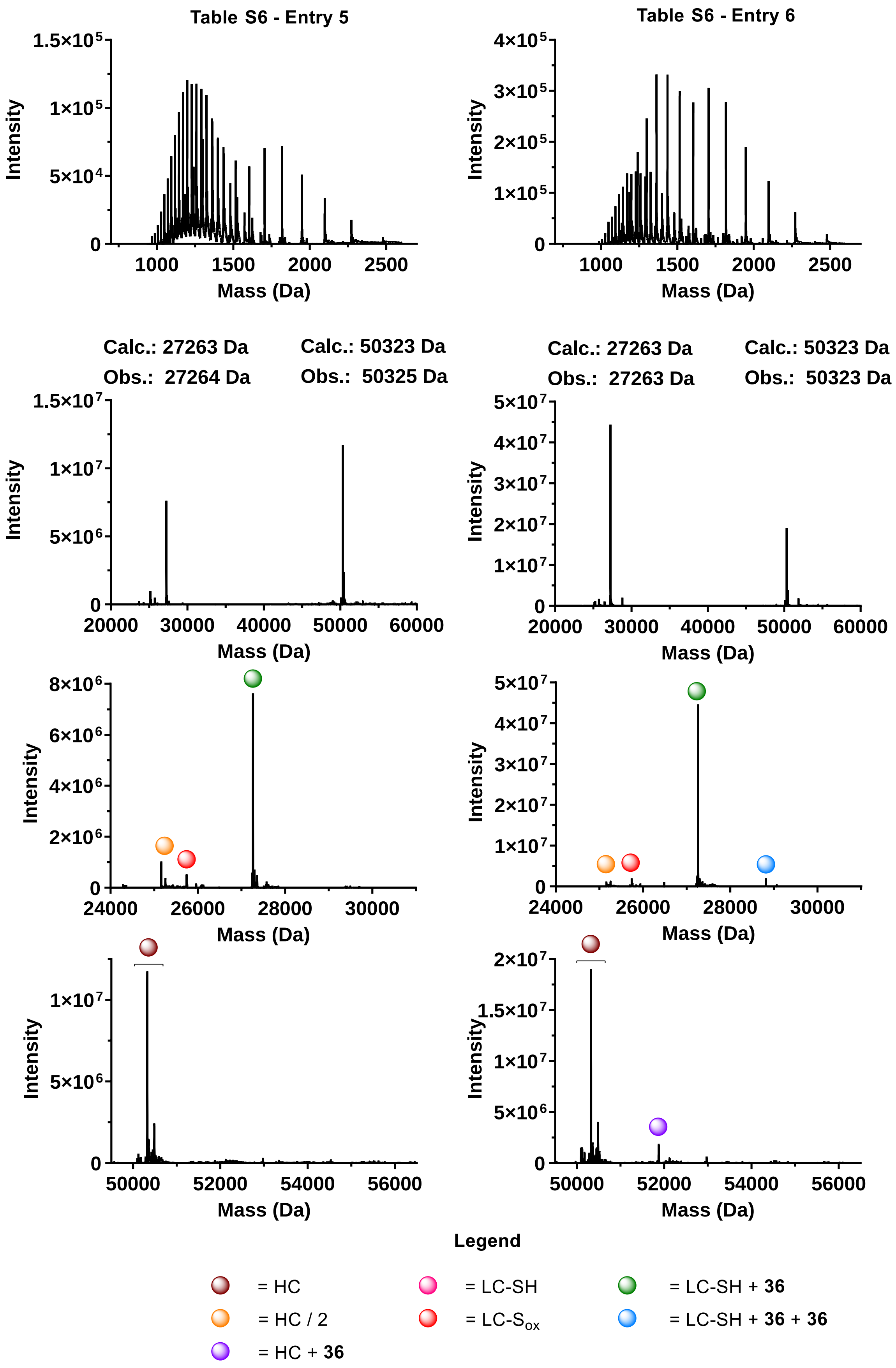
Table S6 - Entry 5
Table S6 - Entry 6
Intensity
Mass (Da)
Calc.: 27263 Da
Obs.: 27264 Da
Calc.: 50323 Da
Obs.: 50325 Da
Calc.: 27263 Da
Obs.: 27263 Da
Calc.: 50323 Da
Obs.: 50323 Da
Legend
= HC
= HC / 2
= HC + 36
= LC-SH
= LC-S$_{ox}$
= LC-SH + 36
= LC-SH + 36 + 36

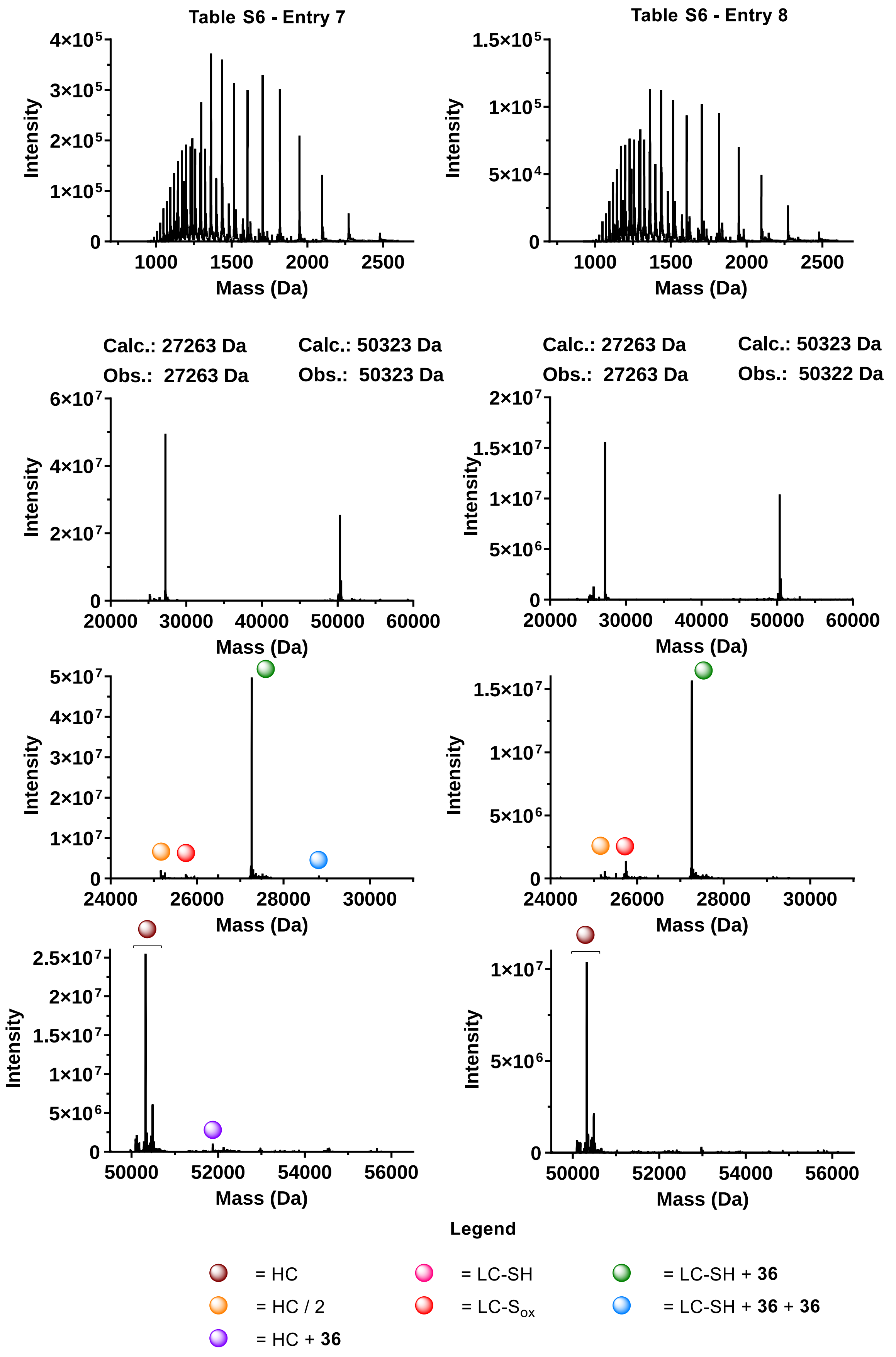
Table S6 - Entry 7
Table S6 - Entry 8
Intensity
Mass (Da)
Calc.: 27263 Da
Obs.: 27263 Da
Calc.: 50323 Da
Obs.: 50323 Da
Calc.: 27263 Da
Obs.: 27263 Da
Calc.: 50323 Da
Obs.: 50322 Da
Legend
= HC
= HC / 2
= HC + 36
= LC-SH
= LC-S$_{ox}$
= LC-SH + 36
= LC-SH + 36 + 36

Table S6 - Entry 9

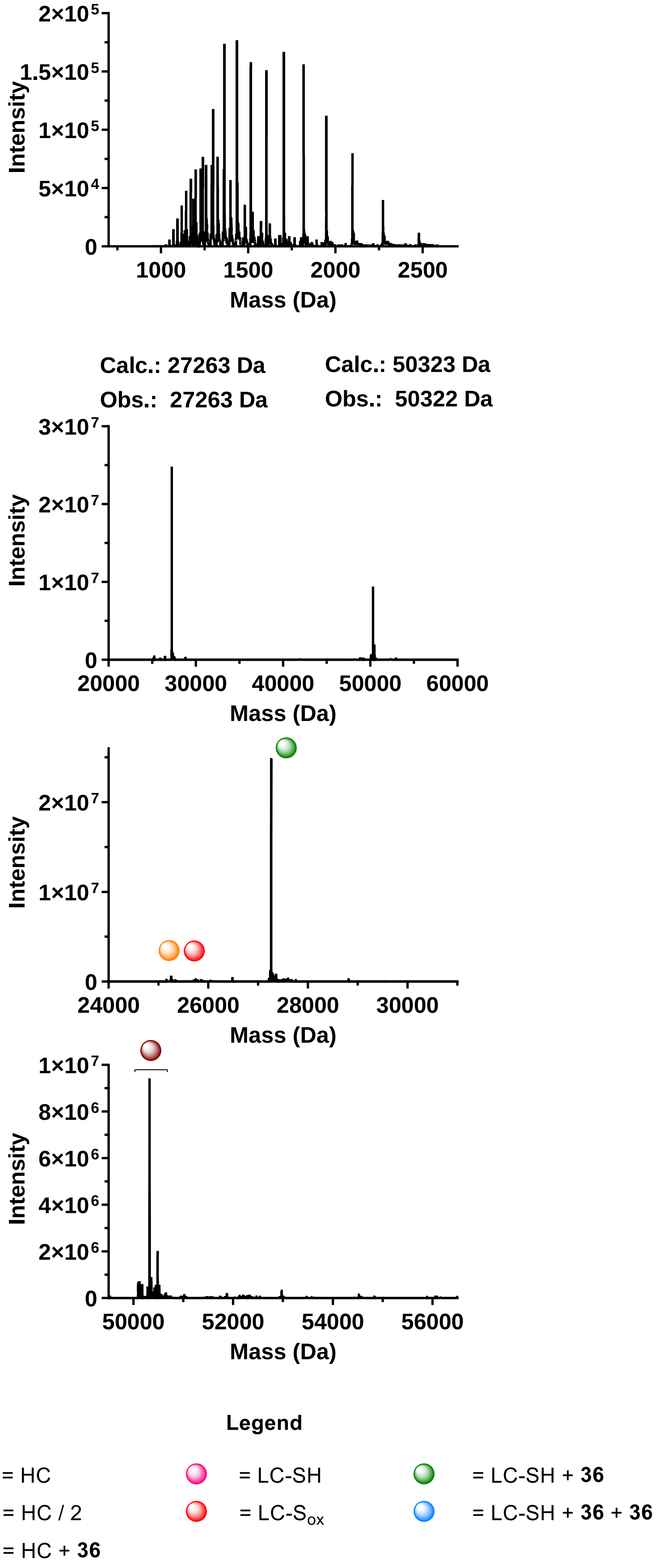

<u>Quantification of HIC chromatograms – Table S6</u>

Quantification of different DAR species (0, 1, 2) was achieved after integration of the peak areas at 280 nm in the Shimadzu software.

The DAR was determined with the following formula, where A corresponds to the area under the peak for the respective species: LC (light chain), LC + 1 (single modified light chain), LC + 2 (double modified light chain).

$$DAR = 2 * \frac{A\ (LC + 1) + 2 * A\ (LC + 2)}{A\ (LC) + A\ (LC + 1) + A\ (LC + 2)}$$

Table S6 – Entry 1

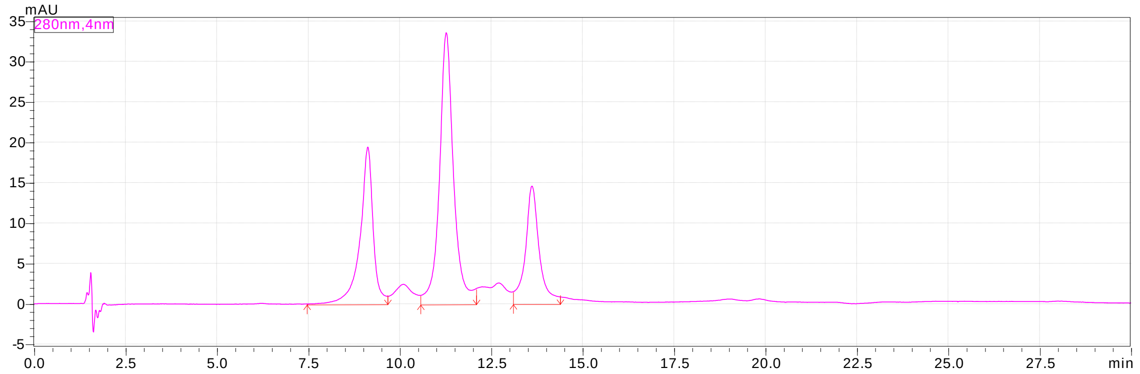

RT [min]	height [mAU]	area	Area %
9.133	484128	19425	27.267
11.278	906698	33601	51.066
13.136	384703	1533	21.667
	1775530	54559	100

Mod. Int.		DAR	0.67
	LC		
33601	LC + 1		
3066	LC + 2		

Table S6 – Entry 2

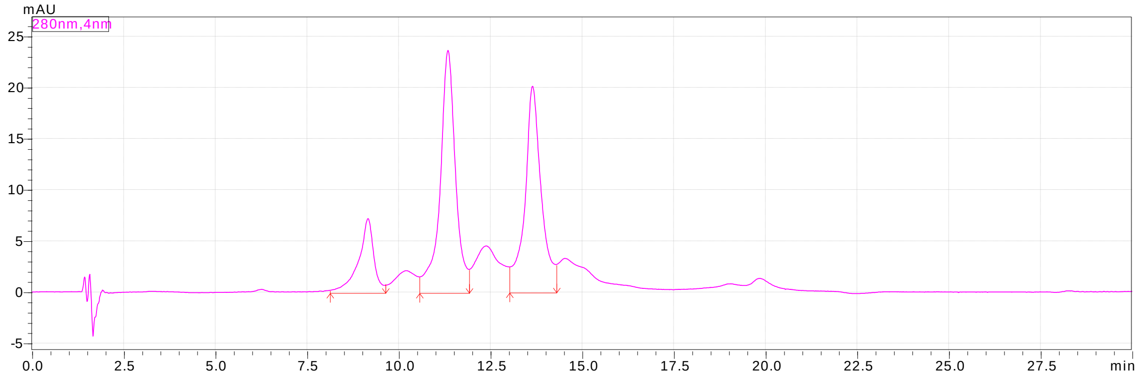

RT [min]	hight [mAU]	area	Area %
9.168	211423	7225	13.758
11.348	694453	23695	45.191
13.653	630837	20146	41.051
	1536713	51066	100

Mod. Int.		DAR	1.25
	LC		
23695	LC+1		
40292	LC+2		

Table S6 – Entry 8

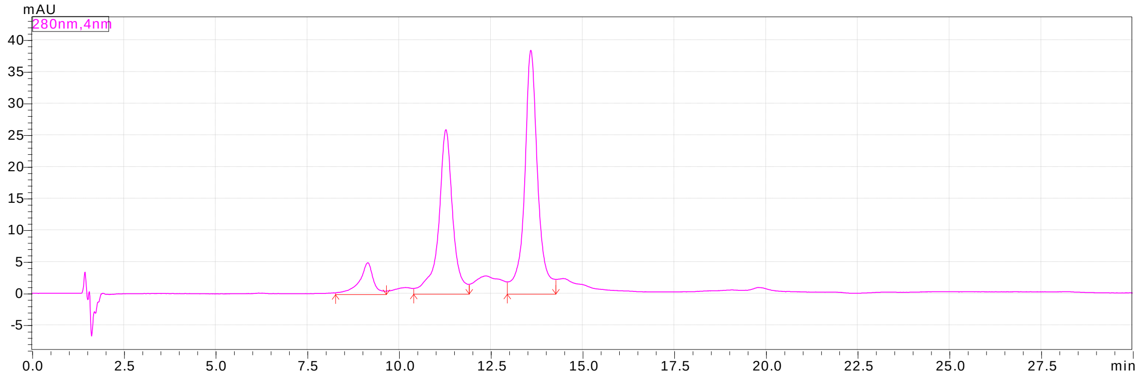

RT [min]	hight [mAU]	area	Area %
9.158	127352	4901	7.157
11.285	690790	25876	38.819
13.6	961371	38397	54.024
	1779513	69173	100

Mod. Int.		DAR	1.48
	LC		
25876	LC+1		
76794	LC+2		

Table S6 – Entry 9

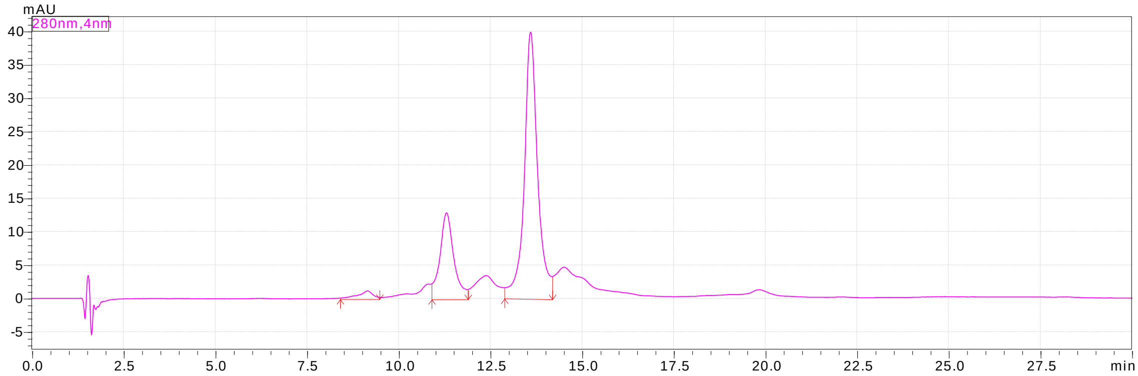

RT [min]	hight [mAU]	area	Area %
9.158	33352	1161	2.4
11.307	341366	12888	24.568
13.601	1014781	39913	73.032
	1389499	53963	100

Mod. Int.		DAR	1.72
	LC		
12888	LC+1		
79826	LC+2		

Intact protein MS: Raw and Deconvoluted Spectra – ADCs 40 and 41 (Figure 5A)

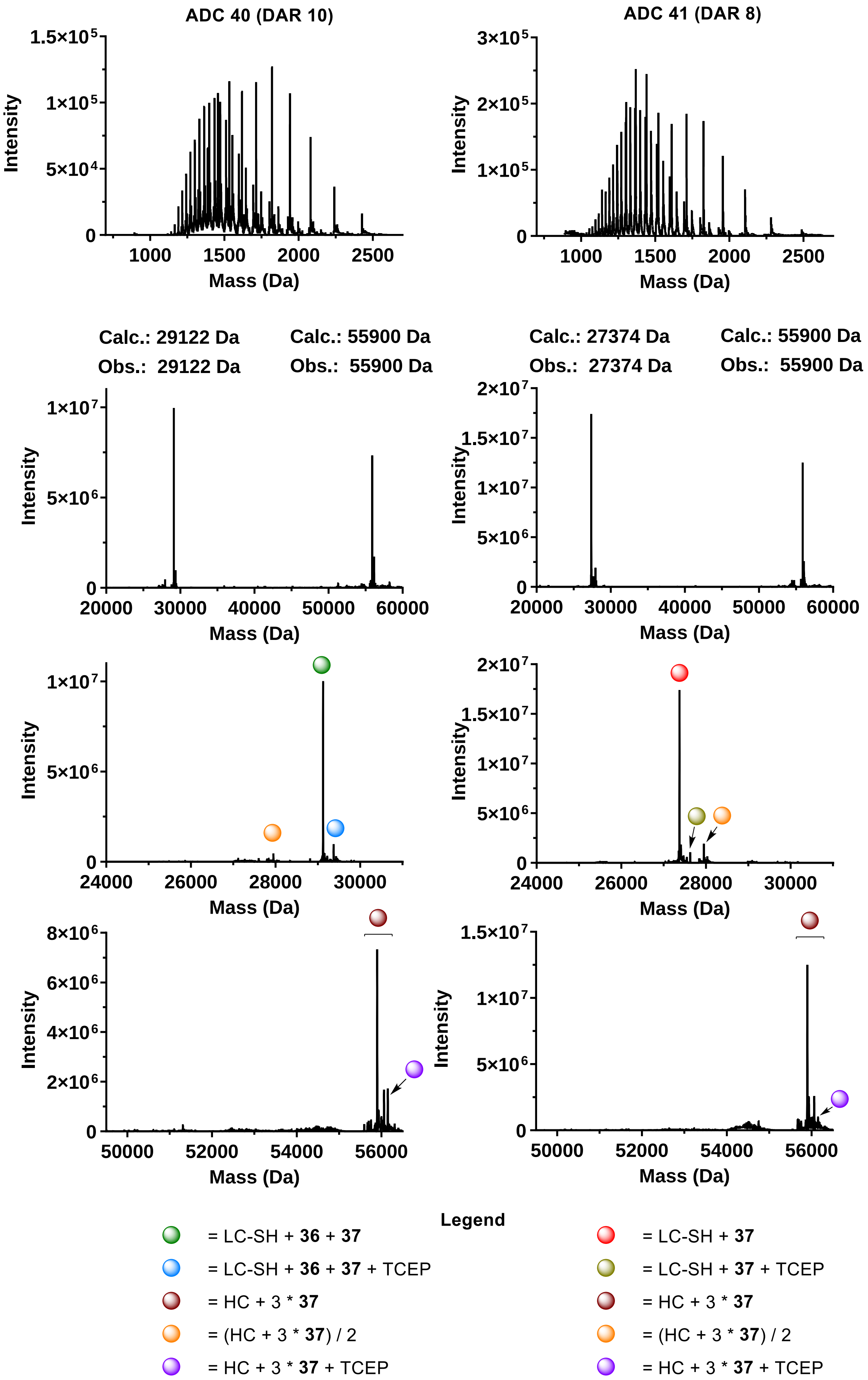

7.9 CD30 bystander cell killing assays

In vitro cytotoxicity – direct killing:

To investigate the direct cytotoxicity of ADCs, 5,000 CD30-positive cells were seeded in 100 µl medium per well and incubated for 4 days with increasing concentrations of ADCs **39** – **41** (0.27 pM – 80 nM) to generate a dose–response curve. Killing was analysed using resazurin cell viability dye at a final concentration of 55 µmol/l (Merck, Germany) by dividing the fluorescence from control cells in medium by the fluorescence of ADC-treated cells. Fluorescence emission at 590 nmol/l was measured on a microplate reader Infinite 200 PRO (Tecan Group Ltd., Switzerland).

In vitro cytotoxicity - supernatant-based bystander killing:

For the supernatant-based bystander experiment, 20,000 CD30-positive cells were seeded in 100 µl medium and incubated for 4 days with increasing concentrations of ADCs **39** – **41** (0.27 pM – 80 nM). Afterwards, 50 µl of the supernatant of the treated cells was transferred to 50 µl of CD30-negative HL-60 cells (5,000 cells/well) and incubated for 4 days. Resazurin readout was performed as described before.

Cell lines:

Cell lines were purchased from the "German Collection of Microorganisms and Cell Cultures" (DSMZ, Leibniz Institute, Braunschweig, Germany), American Type Culture Collection (ATCC, USA) or Merck (Germany):

Karpas-299 (Merck),
HL-60 (DSMZ),
SR-786 (ACC 369, DSMZ),
L-540 (ACC 72, DSMZ),
L-428 (ACC 197, DSMZ),
SU-DHL-1 (ACC 356, DSMZ),

Cells were cultured according to the manufacturer's instructions in either RPMI 1640 or DMEM medium supplemented with GlutaMAX (Gibco, Thermo Fisher Scientific, USA) and 10-20% fetal bovine serum (FBS; Gibco, Thermo Fisher Scientific, USA).

EC50 calculations: nonlinear fit: [inhibitor] vs. response – Figure 6; Figure S25

Nonlinear regression was performed in Graphpad Prism 10.3.1 using concentration in nM on the x-axis.

Nonlinear fit: [inhibitor] vs. response – SR-786 cells (Figure 6B)

Table of results

	Nonlin fit<br>Table of results	A<br>**39** (DAR2 MMAF)	B<br>**40** (DAR8 MMAE DAR2 MMAF)	C<br>**41** (DAR8 MMAE)
1	**[Inhibitor] vs. response -- Variable slope**			
2	**Best-fit values**			
3	Bottom	3.833	0.5900	0.8940
4	Top	97.86	99.80	98.41
5	HillSlope	2.566	1.920	2.301
6	IC50	0.02676	0.004804	0.01330
7	logIC50	-1.572	-2.318	-1.876
8	Span	94.03	99.21	97.51
9	**95% CI (profile likelihood)**			
10	Bottom	1.594 to 6.060	-0.8230 to 1.993	-0.01453 to 1.801
11	Top	94.90 to 100.9	96.55 to 103.3	96.81 to 100.0
12	HillSlope	2.016 to 4.639	1.615 to 2.409	2.083 to 2.583
13	IC50	0.02317 to 0.03030	0.004390 to 0.005219	0.01256 to 0.01408
14	logIC50	-1.635 to -1.519	-2.358 to -2.282	-1.901 to -1.851
15	**Goodness of Fit**			
16	Degrees of Freedom	16	16	16
17	R squared	0.9952	0.9971	0.9991
18	Sum of Squares	174.5	84.62	33.47
19	Sy.x	3.302	2.300	1.446
20	**Constraints**			
21	Bottom	Bottom > -10	Bottom > -10	Bottom > -10
22	Top	Top < 110	Top < 110	Top < 110
23	IC50	IC50 > 0	IC50 > 0	IC50 > 0
24				
25	**Number of points**			
26	# of X values	20	20	20
27	# Y values analyzed	20	20	20
28				

Nonlinear fit: [inhibitor] vs. response – SR-786 cells Bystander HL60 (Figure 6B)

Table of results

	Nonlin fit Table of results	A 39 (DAR2 MMAF)	B 40 (DAR8 MMAE DAR2 MMAF)	C 41 (DAR8 MMAE)
1	**[Inhibitor] vs. response -- Variable slope**			
2	**Best-fit values**			
3	Bottom	94.60	7.281	6.317
4	Top	110.0	96.01	96.67
5	HillSlope	Unstable	3.217	1.964
6	IC50	0.2945	0.2341	0.2504
7	logIC50	-0.5309	-0.6307	-0.6013
8	Span	15.40	88.73	90.36
9	**95% CI (profile likelihood)**			
10	Bottom	91.15 to 98.05	4.245 to 10.30	4.199 to 8.396
11	Top	107.0 to ???	93.03 to 99.03	94.70 to 98.67
12	HillSlope	(Very wide)	2.175 to ???	1.639 to 2.478
13	IC50	??? to 0.3878	0.1971 to ???	0.2263 to 0.2754
14	logIC50	??? to -0.4114	-0.7054 to ???	-0.6454 to -0.5600
15	**Goodness of Fit**			
16	Degrees of Freedom	16	16	16
17	R squared	0.7466	0.9929	0.9970
18	Sum of Squares	419.1	253.0	105.5
19	Sy.x	5.118	3.977	2.568
20	**Constraints**			
21	Bottom	Bottom > -10	Bottom > -10	Bottom > -10
22	Top	Top < 110	Top < 110	Top < 110
23	IC50	IC50 > 0	IC50 > 0	IC50 > 0
24				
25	**Number of points**			
26	# of X values	20	20	20
27	# Y values analyzed	20	20	20

Nonlinear fit: [inhibitor] vs. response – L-428 cells (Figure 6C)

Table of results

	Nonlin fit<br>Table of results	A<br>**39** (DAR2 MMAF)	B<br>**40** (DAR8 MMAE DAR2 MMAF)	C<br>**41** (DAR8 MMAE)
1	**[Inhibitor] vs. response -- Variable slope**			Hit constraint
2	**Best-fit values**			
3	Bottom	16.15	13.80	32.46
4	Top	93.06	97.88	~ 110.0
5	HillSlope	1.722	1.070	0.3080
6	IC50	0.02317	0.01053	0.03507
7	logIC50	-1.635	-1.978	-1.455
8	Span	76.92	84.08	~ 77.54
9	**95% CI (profile likelihood)**			
10	Bottom	12.90 to 19.30	11.44 to 16.09	5.543 to 43.29
11	Top	88.41 to 98.47	92.40 to 104.7	
12	HillSlope	1.155 to 3.407	0.8508 to 1.353	0.1985 to 0.5691
13	IC50	0.01827 to 0.02910	0.007824 to 0.01362	0.01003 to 0.6854
14	logIC50	-1.738 to -1.536	-2.107 to -1.866	-1.999 to -0.1640
15	**Goodness of Fit**			
16	Degrees of Freedom	16	16	16
17	R squared	0.9853	0.9916	0.9426
18	Sum of Squares	333.4	174.4	462.5
19	Sy.x	4.565	3.301	5.376
20	**Constraints**			
21	Bottom	Bottom > -10	Bottom > -10	Bottom > -10
22	Top	Top < 110	Top < 110	Top < 110
23	IC50	IC50 > 0	IC50 > 0	IC50 > 0
24				
25	**Number of points**			
26	# of X values	20	20	20
27	# Y values analyzed	20	20	20

Nonlinear fit: [inhibitor] vs. response – L-428 cells Bystander HL60 (Figure 6C)

Table of results

Nonlin fit<br>Table of results	A<br>39 (DAR2 MMAF)	B<br>40 (DAR8 MMAE DAR2 MMAF)	C<br>41 (DAR8 MMAE)
1 **[Inhibitor] vs. response -- Variable slope**			
2 **Best-fit values**			
3 Bottom	-9.950	14.92	9.164
4 Top	101.1	95.58	96.38
5 HillSlope	0.2629	1.171	1.467
6 IC50	54452	0.1786	0.1619
7 logIC50	4.736	-0.7480	-0.7908
8 Span	111.0	80.66	87.21
9 **95% CI (profile likelihood)**			
10 Bottom	???	5.365 to 22.07	6.129 to 12.07
11 Top	???	88.02 to 106.0	93.44 to 99.46
12 HillSlope	???	0.5935 to 3.695	1.154 to 1.867
13 IC50	???	0.1017 to 0.2837	0.1365 to 0.1916
14 logIC50	???	-0.9925 to -0.5471	-0.8648 to -0.7176
15 **Goodness of Fit**			
16 Degrees of Freedom	16	16	16
17 R squared	0.6391	0.9645	0.9938
18 Sum of Squares	298.8	902.0	191.0
19 Sy.x	4.322	7.508	3.455
20 **Constraints**			
21 Bottom	Bottom > -10	Bottom > -10	Bottom > -10
22 Top	Top < 110	Top < 110	Top < 110
23 IC50	IC50 > 0	IC50 > 0	IC50 > 0
24			
25 **Number of points**			
26 # of X values	20	20	20
27 # Y values analyzed	20	20	20

Nonlinear fit: [inhibitor] vs. response – SU-DHL-1 cells (Figure S25A)

	Nonlin fit Table of results	A 39 (DAR2 MMAF)	B 40 (DAR8 MMAE DAR2 MMAF)	C 41 (DAR8 MMAE)
1	**[Inhibitor] vs. response -- Variable slope**			
2	**Best-fit values**			
3	Bottom	8.896	8.420	9.386
4	Top	99.00	100.0	99.22
5	HillSlope	1.277	2.111	2.339
6	IC50	0.06248	0.008326	0.02318
7	logIC50	-1.204	-2.080	-1.635
8	Span	90.10	91.58	89.83
9	**95% CI (profile likelihood)**			
10	Bottom	5.362 to 12.22	7.771 to 9.068	7.870 to 10.89
11	Top	95.10 to 103.2	98.81 to 101.2	97.12 to 101.4
12	HillSlope	0.9785 to 1.738	1.984 to 2.248	1.920 to 3.234
13	IC50	0.05076 to 0.07745	0.007977 to 0.008695	0.02158 to 0.02509
14	logIC50	-1.295 to -1.111	-2.098 to -2.061	-1.666 to -1.601
15	**Goodness of Fit**			
16	Degrees of Freedom	16	16	16
17	R squared	0.9916	0.9994	0.9975
18	Sum of Squares	259.4	17.13	80.49
19	Sy.x	4.026	1.035	2.243
20	**Constraints**			
21	Bottom	Bottom > -10	Bottom > -10	Bottom > -10
22	Top	Top < 110	Top < 110	Top < 110
23	IC50	IC50 > 0	IC50 > 0	IC50 > 0
24				
25	**Number of points**			
26	# of X values	20	20	20
27	# Y values analyzed	20	20	20

Nonlinear fit: [inhibitor] vs. response – SU-DHL-1 cells Bystander HL60 (Figure S25A)

Nonlin fit<br>Table of results	A<br>**39** (DAR2 MMAF)	B<br>**40** (DAR8 MMAE DAR2 MMAF)	C<br>**41** (DAR8 MMAE)
1 **[Inhibitor] vs. response -- Variable slope**			
2 **Best-fit values**			
3 Bottom	98.52	8.992	9.537
4 Top	105.1	96.94	94.88
5 HillSlope	Unstable	1.626	1.721
6 IC50	1.541	0.2713	0.2760
7 logIC50	0.1878	-0.5666	-0.5590
8 Span	6.593	87.95	85.34
9 **95% CI (profile likelihood)**			
10 Bottom	98.07 to 101.8	5.382 to 12.42	3.871 to 14.72
11 Top	100.1 to ???	93.84 to 100.2	90.11 to 99.98
12 HillSlope	(Very wide)	1.238 to 2.326	1.074 to ???
13 IC50	???	0.2268 to 0.3233	0.2080 to 0.3610
14 logIC50	???	-0.6443 to -0.4905	-0.6820 to -0.4425
15 **Goodness of Fit**			
16 Degrees of Freedom	16	16	16
17 R squared	0.2565	0.9920	0.9803
18 Sum of Squares	527.1	253.9	602.7
19 Sy.x	5.740	3.984	6.137
20 **Constraints**			
21 Bottom	Bottom > -10	Bottom > -10	Bottom > -10
22 Top	Top < 110	Top < 110	Top < 110
23 IC50	IC50 > 0	IC50 > 0	IC50 > 0
24			
25 **Number of points**			
26 # of X values	20	20	20
27 # Y values analyzed	20	20	20

<u>Nonlinear fit: [inhibitor] vs. response – Karpas-299 cells (Figure S25B)</u>

Table of results

	Nonlin fit<br>Table of results	A<br>39 (DAR2 MMAF)	B<br>40 (DAR8 MMAE DAR2 MMAF)	C<br>41 (DAR8 MMAE)
1	**[Inhibitor] vs. response -- Variable slope**			
2	**Best-fit values**			
3	Bottom	19.62	5.566	6.676
4	Top	97.80	99.09	100.4
5	HillSlope	2.376	1.685	2.400
6	IC50	0.03313	0.009110	0.03171
7	logIC50	-1.480	-2.041	-1.499
8	Span	78.18	93.53	93.71
9	**95% CI (profile likelihood)**			
10	Bottom	16.33 to 22.89	4.622 to 6.506	4.565 to 8.779
11	Top	93.46 to 102.2	97.27 to 101.0	97.62 to 103.2
12	HillSlope	1.755 to 3.759	1.542 to 1.841	1.997 to 3.005
13	IC50	0.02646 to 0.04190	0.008528 to 0.009733	0.02811 to 0.03583
14	logIC50	-1.577 to -1.378	-2.069 to -2.012	-1.551 to -1.446
15	**Goodness of Fit**			
16	Degrees of Freedom	16	16	16
17	R squared	0.9854	0.9988	0.9958
18	Sum of Squares	376.6	34.09	152.8
19	Sy.x	4.851	1.460	3.090
20	**Constraints**			
21	Bottom	Bottom > -10	Bottom > -10	Bottom > -10
22	Top	Top < 110	Top < 110	Top < 110
23	IC50	IC50 > 0	IC50 > 0	IC50 > 0
24				
25	**Number of points**			
26	# of X values	20	20	20
27	# Y values analyzed	20	20	20

Nonlinear fit: [inhibitor] vs. response – Karpas-299 cells Bystander HL60 (Figure S25B)

Table of results

	Nonlin fit<br>Table of results	A<br>39 (DAR2 MMAF)	B<br>40 (DAR8 MMAE DAR2 MMAF)	C<br>41 (DAR8 MMAE)
1	**[Inhibitor] vs. response -- Variable slope**	Hit constraint		
2	**Best-fit values**			
3	Bottom	95.66	6.188	4.848
4	Top	~ 110.0	97.45	97.33
5	HillSlope	Unstable	2.816	2.297
6	IC50	0.08003	0.2086	0.2251
7	logIC50	-1.097	-0.6808	-0.6476
8	Span	~ 14.34	91.26	92.49
9	**95% CI (profile likelihood)**			
10	Bottom	89.08 to 104.6	3.887 to 8.481	2.648 to 7.027
11	Top		95.17 to 99.75	95.20 to 99.49
12	HillSlope	(Very wide)	2.253 to 5.000	1.892 to 3.031
13	IC50	???	0.1840 to 0.2497	0.2021 to 0.2496
14	logIC50	???	-0.7353 to -0.6026	-0.6945 to -0.6027
15	**Goodness of Fit**			
16	Degrees of Freedom	16	16	16
17	R squared	0.4043	0.9961	0.9966
18	Sum of Squares	2355	145.5	125.6
19	Sy.x	12.13	3.015	2.802
20	**Constraints**			
21	Bottom	Bottom > -10	Bottom > -10	Bottom > -10
22	Top	Top < 110	Top < 110	Top < 110
23	IC50	IC50 > 0	IC50 > 0	IC50 > 0
24				
25	**Number of points**			
26	# of X values	20	20	20
27	# Y values analyzed	20	20	20

Nonlinear fit: [inhibitor] vs. response – L-540 cells (Figure S25C)

Table of results

Nonlin fit<br>Table of results	A<br>39 (DAR2 MMAF)	B<br>40 (DAR8 MMAE DAR2 MMAF)	C<br>41 (DAR8 MMAE)
1 [Inhibitor] vs. response -- Variable slope			
2 Best-fit values			
3 Bottom	4.538	-1.830	-0.7284
4 Top	91.44	105.5	96.74
5 HillSlope	1.329	0.7845	1.185
6 IC50	0.03731	0.008216	0.03181
7 logIC50	-1.428	-2.085	-1.497
8 Span	86.90	107.3	97.47
9 95% CI (profile likelihood)			
10 Bottom	-0.01492 to 8.931	-3.828 to 0.09229	-2.979 to 1.474
11 Top	84.99 to 99.16	99.57 to ???	93.34 to 100.4
12 HillSlope	0.9091 to 1.949	0.6844 to 0.9002	1.010 to 1.394
13 IC50	0.02569 to 0.05221	0.006434 to 0.01033	0.02704 to 0.03724
14 logIC50	-1.590 to -1.282	-2.192 to -1.986	-1.568 to -1.429
15 Goodness of Fit			
16 Degrees of Freedom	16	16	16
17 R squared	0.9792	0.9964	0.9959
18 Sum of Squares	593.3	98.59	138.9
19 Sy.x	6.089	2.482	2.947
20 Constraints			
21 Bottom	Bottom > -10	Bottom > -10	Bottom > -10
22 Top	Top < 110	Top < 110	Top < 110
23 IC50	IC50 > 0	IC50 > 0	IC50 > 0
24			
25 Number of points			
26 # of X values	20	20	20
27 # Y values analyzed	20	20	20

<u>Nonlinear fit: [inhibitor] vs. response – L-540 cells Bystander HL60 (Figure S25C)</u>

Table of results

	Nonlin fit Table of results	A 39 (DAR2 MMAF)	B 40 (DAR8 MMAE DAR2 MMAF)	C 41 (DAR8 MMAE)
1	**[Inhibitor] vs. response -- Variable slope**	Hit constraint		
2	**Best-fit values**			
3	Bottom	97.93	2.135	2.250
4	Top	~ 110.0	94.63	102.9
5	HillSlope	Unstable	2.233	4.014
6	IC50	0.07812	0.1897	0.2540
7	logIC50	-1.107	-0.7219	-0.5951
8	Span	~ 12.07	92.49	100.7
9	**95% CI (profile likelihood)**			
10	Bottom	84.52 to 111.0	-1.147 to 5.382	-2.004 to 6.478
11	Top		91.40 to 97.89	99.00 to 107.1
12	HillSlope	(Very wide)	1.735 to 3.061	2.203 to ???
13	IC50	???	0.1604 to 0.2237	0.2028 to ???
14	logIC50	???	-0.7948 to -0.6504	-0.6928 to ???
15	**Goodness of Fit**			
16	Degrees of Freedom	16	16	16
17	R squared	0.1194	0.9925	0.9892
18	Sum of Squares	5121	281.7	503.3
19	Sy.x	17.89	4.196	5.609
20	**Constraints**			
21	Bottom	Bottom > -10	Bottom > -10	Bottom > -10
22	Top	Top < 110	Top < 110	Top < 110
23	IC50	IC50 > 0	IC50 > 0	IC50 > 0
24				
25	**Number of points**			
26	# of X values	20	20	20
27	# Y values analyzed	20	20	20

Nonlinear fit: [inhibitor] vs. response – HL-60 cells (Figure S25D)

Table of results

	Nonlin fit<br>Table of results	A<br>39 (DAR2 MMAF)	B<br>40 (DAR8 MMAE DAR2 MMAF)	C<br>41 (DAR8 MMAE)
1	**[Inhibitor] vs. response -- Variable slope**	Hit constraint		Hit constraint
2	**Best-fit values**			
3	Bottom	74.35	28.22	40.75
4	Top	~ 110.0	108.1	~ 110.0
5	HillSlope	0.2809	0.4129	0.3035
6	IC50	0.001703	0.03885	0.1164
7	logIC50	-2.769	-1.411	-0.9339
8	Span	~ 35.65	79.93	~ 69.25
9	**95% CI (profile likelihood)**			
10	Bottom	??? to 79.88	23.42 to 32.37	7.024 to 52.60
11	Top		100.8 to ???	
12	HillSlope	0.07619 to 0.6955	0.3504 to 0.5298	0.1966 to 0.5231
13	IC50	0.0003295 to ???	0.02536 to 0.06466	0.02678 to 5.579
14	logIC50	-3.482 to ???	-1.596 to -1.189	-1.572 to 0.7465
15	**Goodness of Fit**			
16	Degrees of Freedom	16	16	16
17	R squared	0.8222	0.9936	0.9523
18	Sum of Squares	210.0	73.99	312.4
19	Sy.x	3.623	2.150	4.419
20	**Constraints**			
21	Bottom	Bottom > -10	Bottom > -10	Bottom > -10
22	Top	Top < 110	Top < 110	Top < 110
23	IC50	IC50 > 0	IC50 > 0	IC50 > 0
24				
25	**Number of points**			
26	# of X values	20	20	20
27	# Y values analyzed	20	20	20

8. LC-MS spectra [Organic Synthesis]

LC-MS spectrum of 1 [Crude reaction mixture]

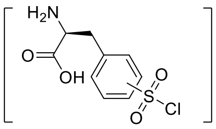

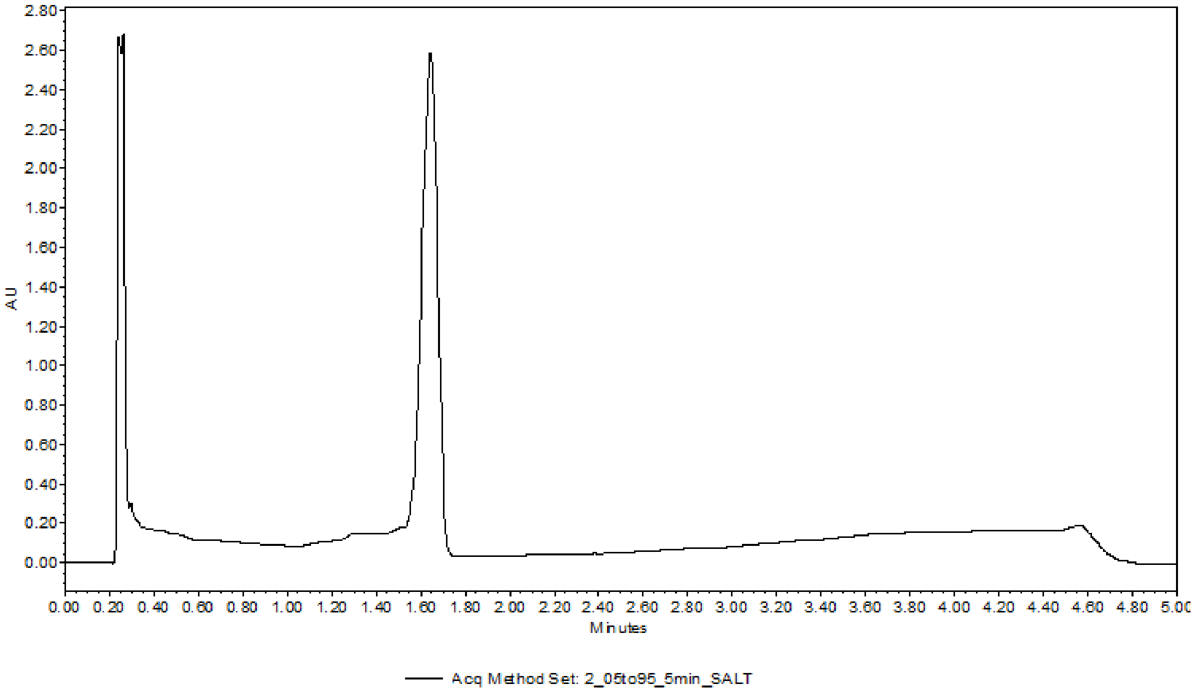

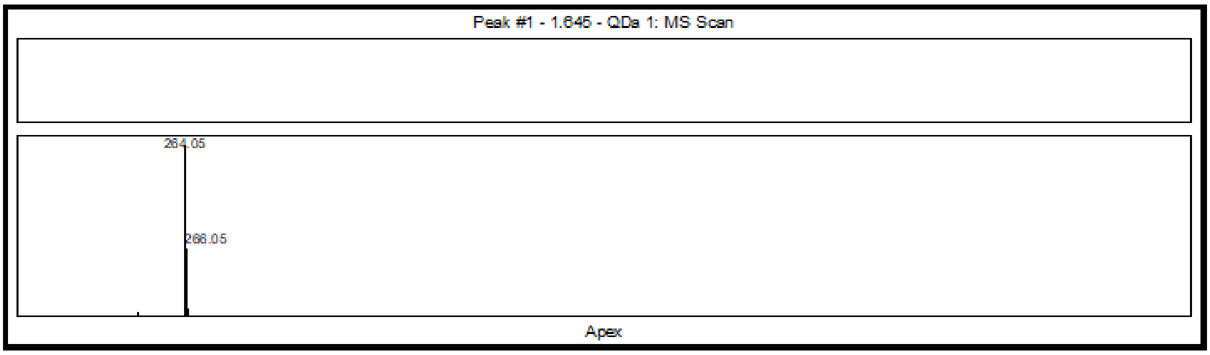

LC-MS spectrum of 2 [Crude reaction mixture]

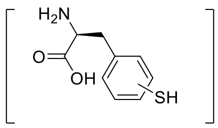

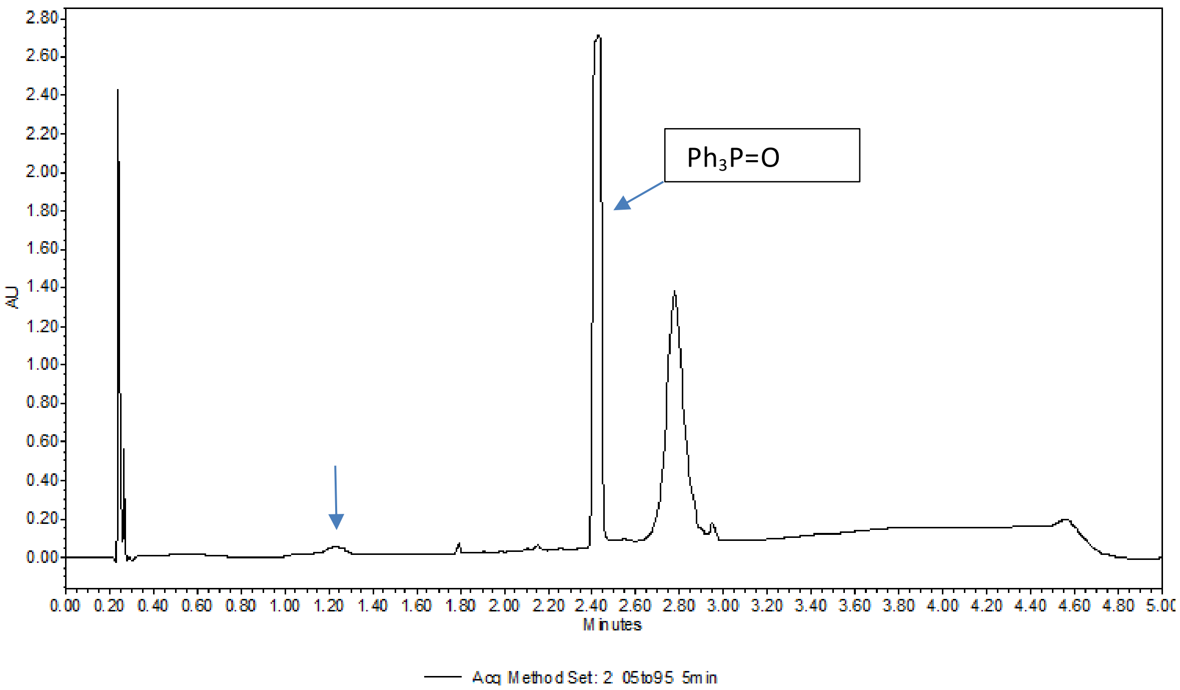

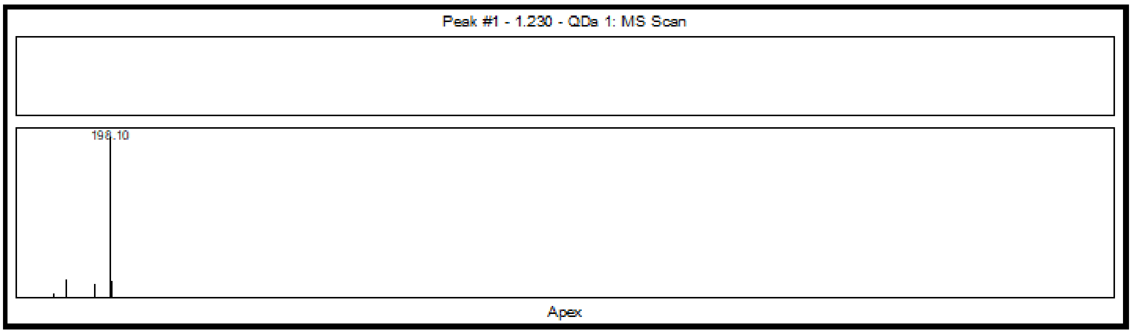

LC-MS spectrum of 3ab

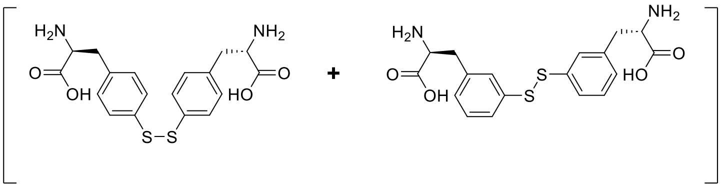

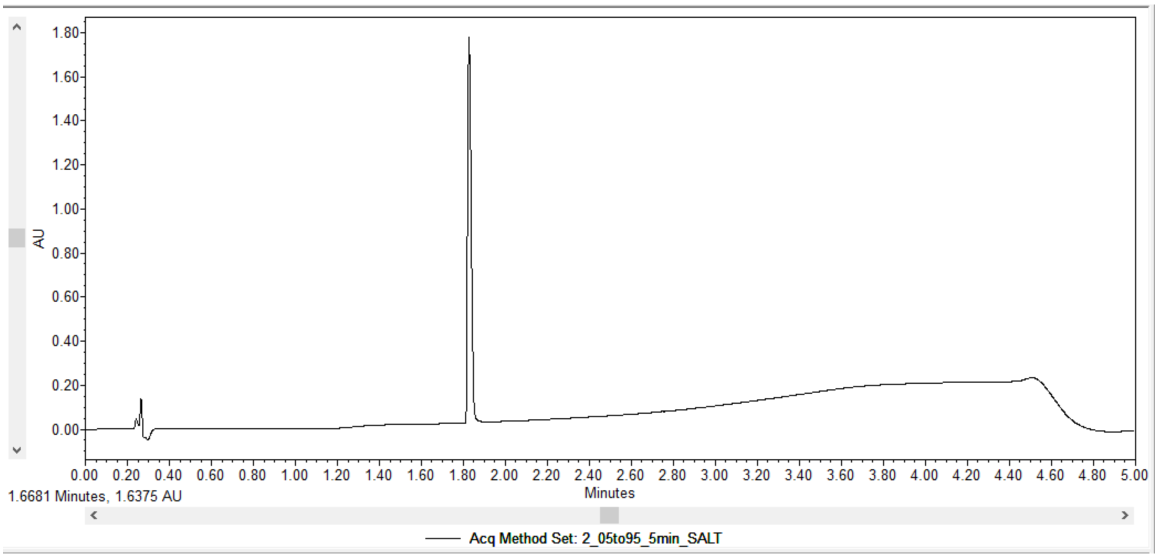

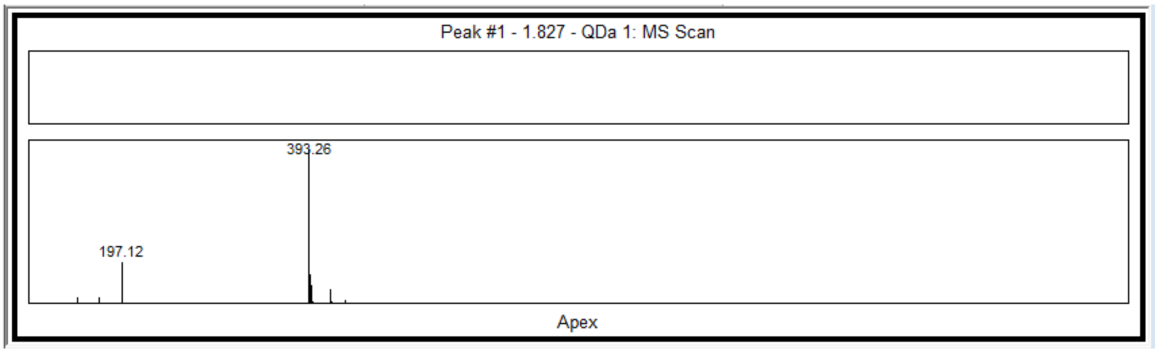

LC-MS spectrum of 4 [Crude reaction mixture]

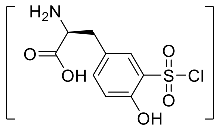

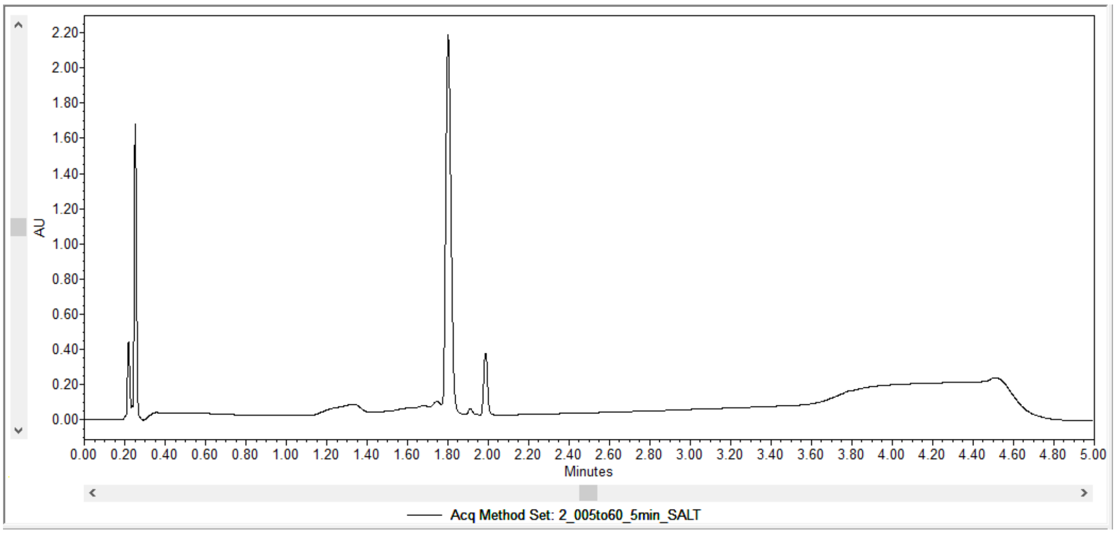

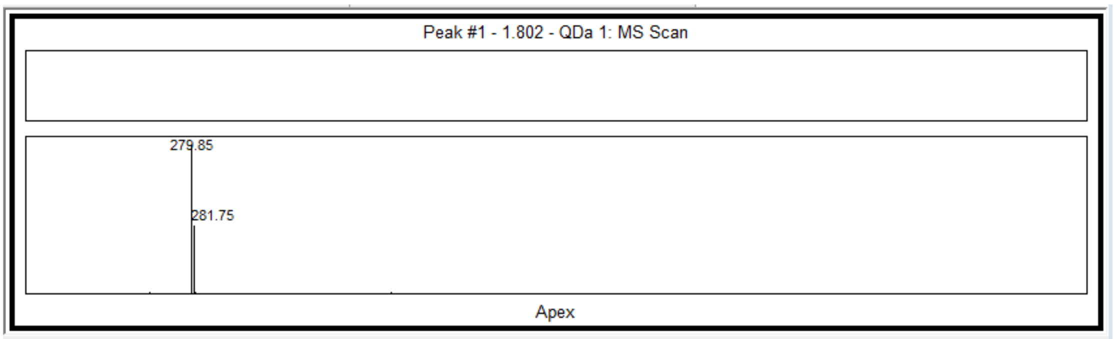

LC-MS spectrum of 5 [Crude reaction mixture]

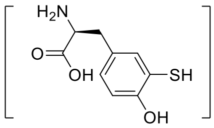

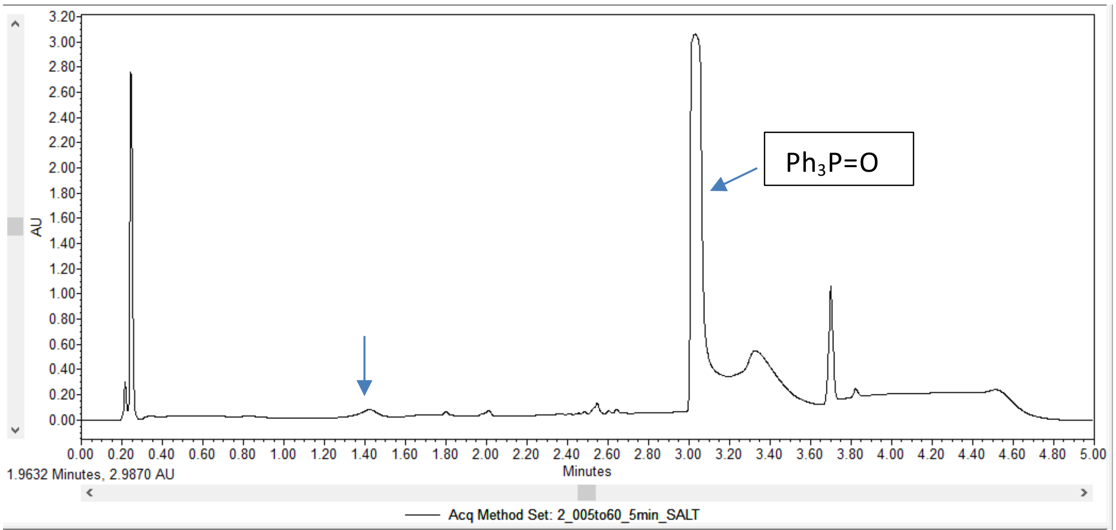

Name	Migration Time (min)	Area (µV*sec)	% Area	Height (µV)	Int Type	Amount	Units	Peak Type	Peak Codes	Corrected Area~ (µV*sec)	MS Match1 Score	MS Match2 Score	MS Match3 Score	Base Peak (Combined) (m/z)	Assigned Mass	Assigned Mass Value (m/z)	Notes

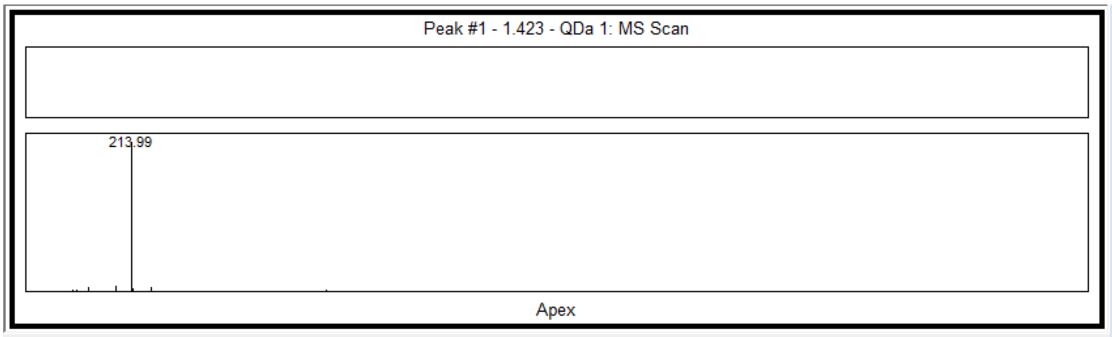

LC-MS spectra of 6

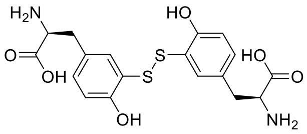

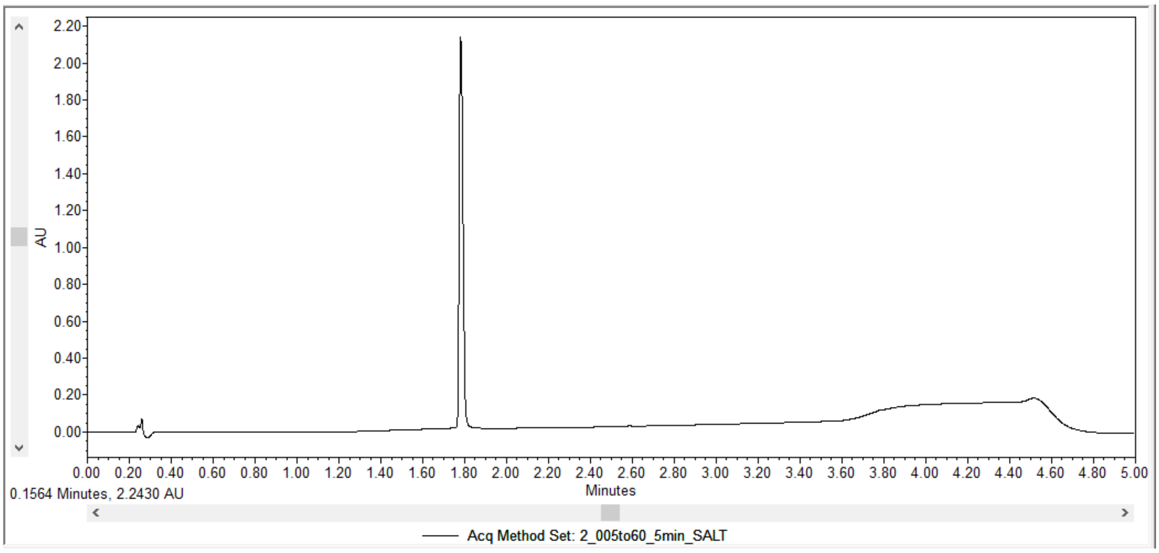

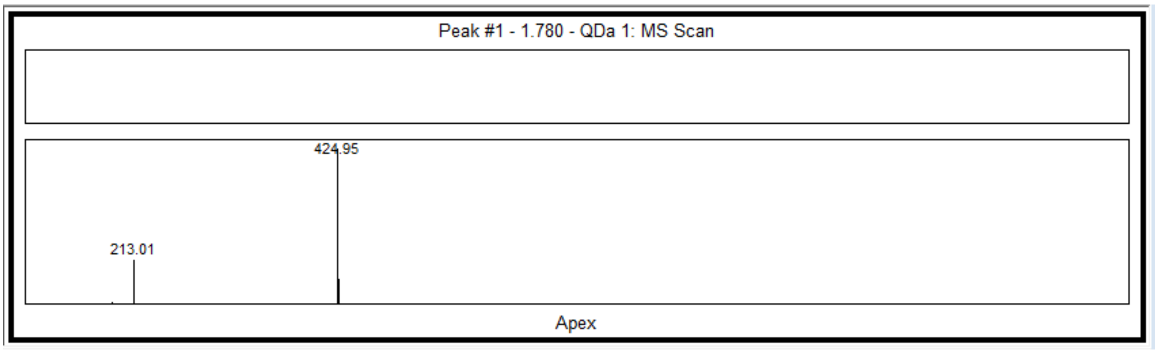

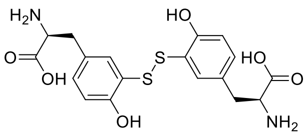
H2N
HO
O
S
HO
OH
S
O
OH
NH2

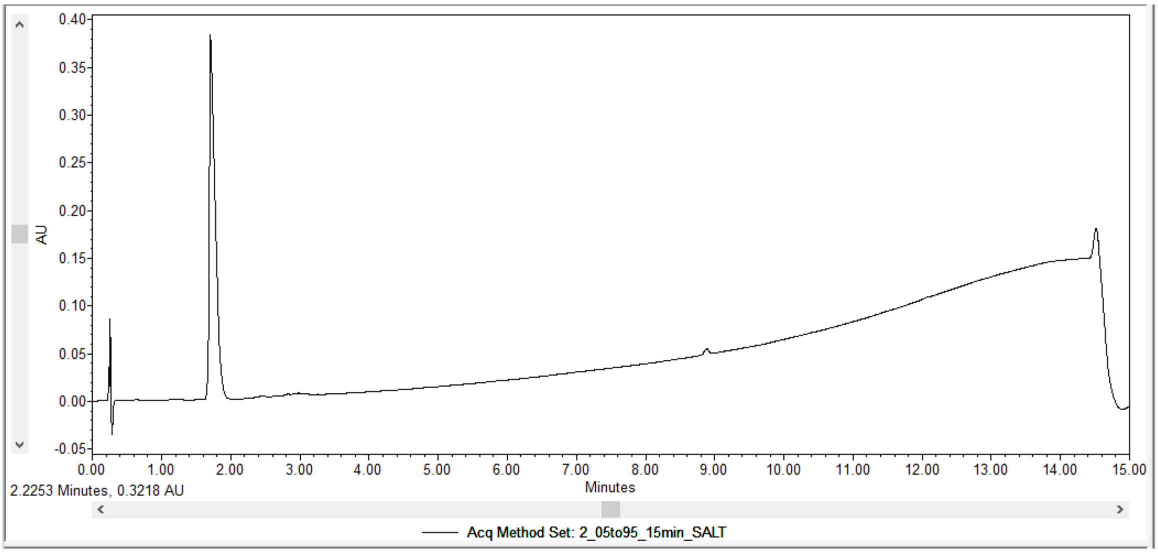
0.40
0.35
0.30
0.25
0.20
0.15
0.10
0.05
0.00
-0.05
AU
0.00
1.00
2.00
3.00
4.00
5.00
6.00
7.00
8.00
9.00
10.00
11.00
12.00
13.00
14.00
15.00
Minutes
2.2253 Minutes, 0.3218 AU
Acq Method Set: 2_05to95_15min_SALT

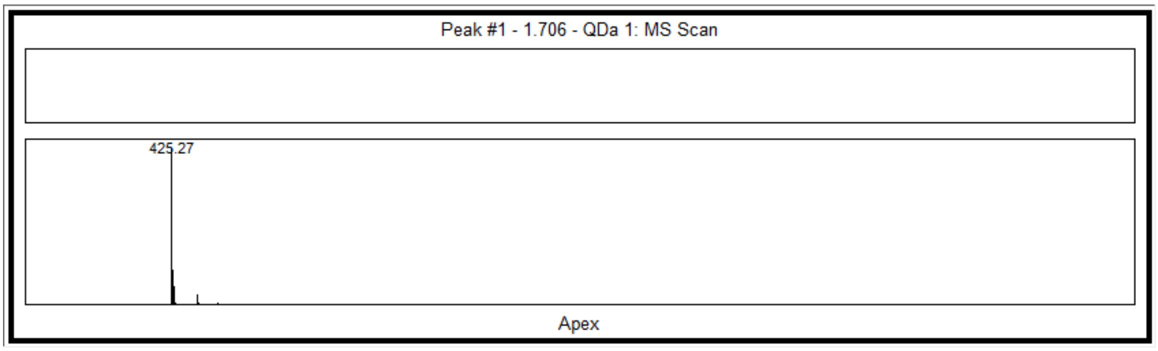
Peak #1 - 1.706 - QDa 1: MS Scan
425.27
Apex

LC-MS spectrum of 7

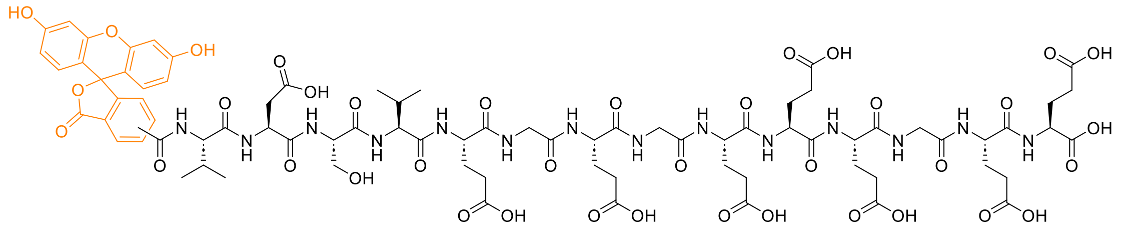

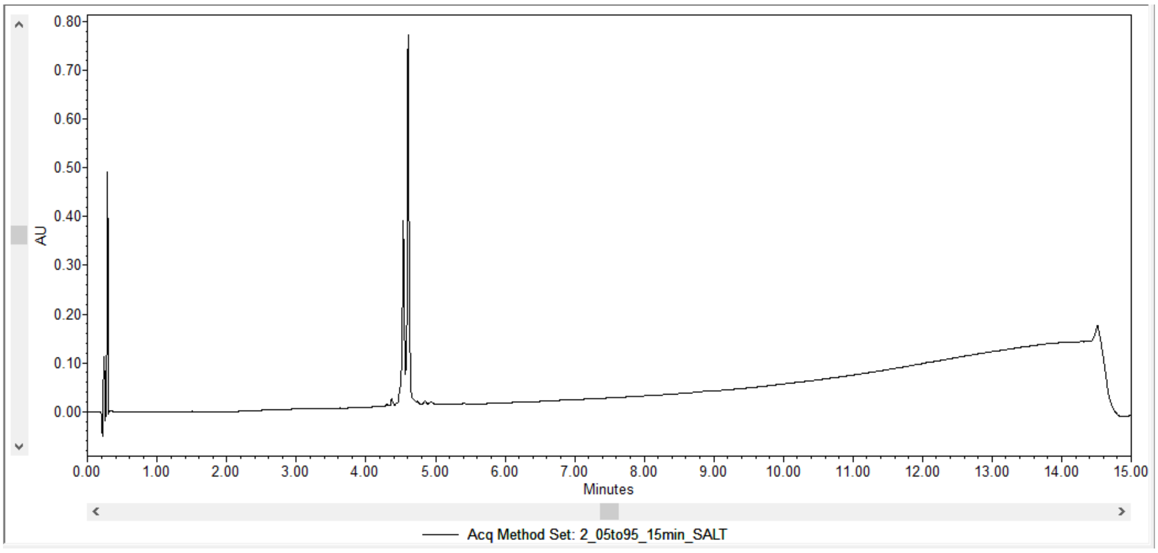

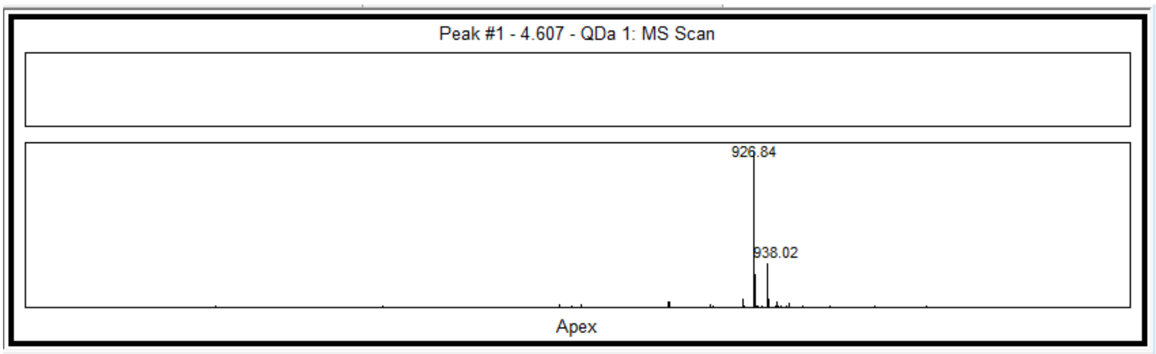

LC-MS spectrum of 9

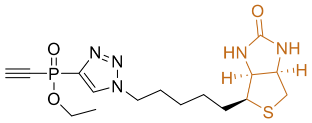

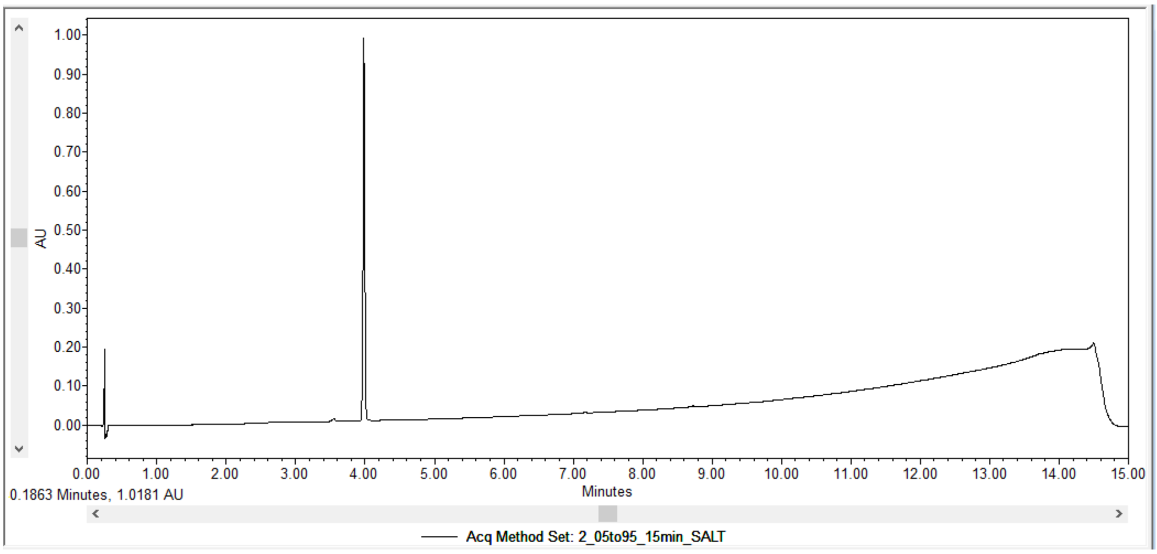

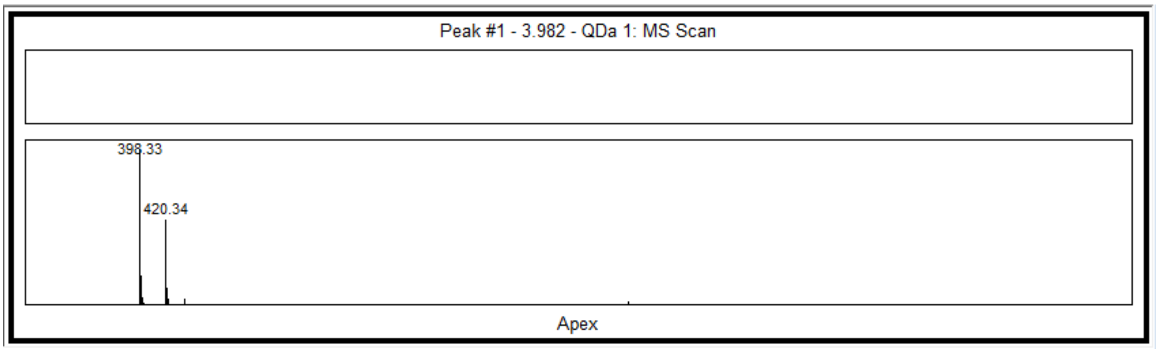

LC-MS spectrum of 10-Z/E

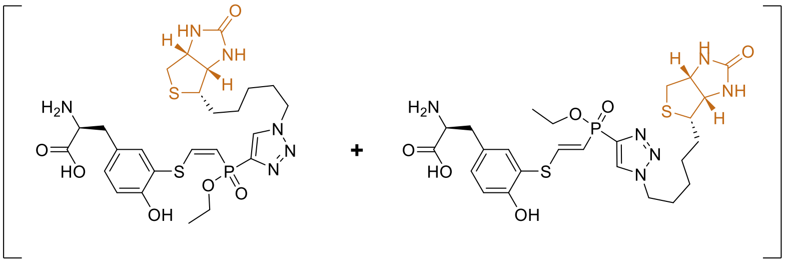

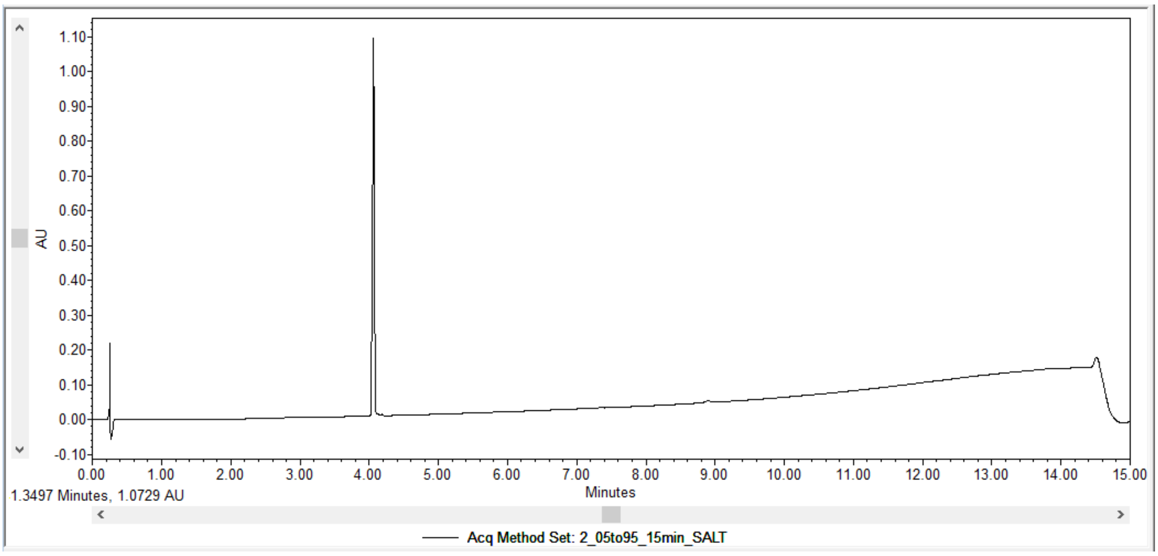

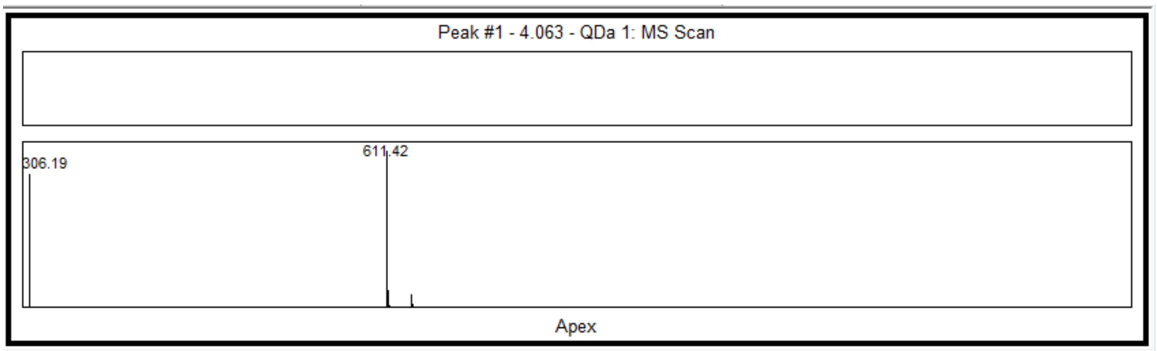

LC-MS spectrum of 11-Z

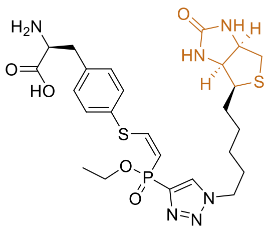

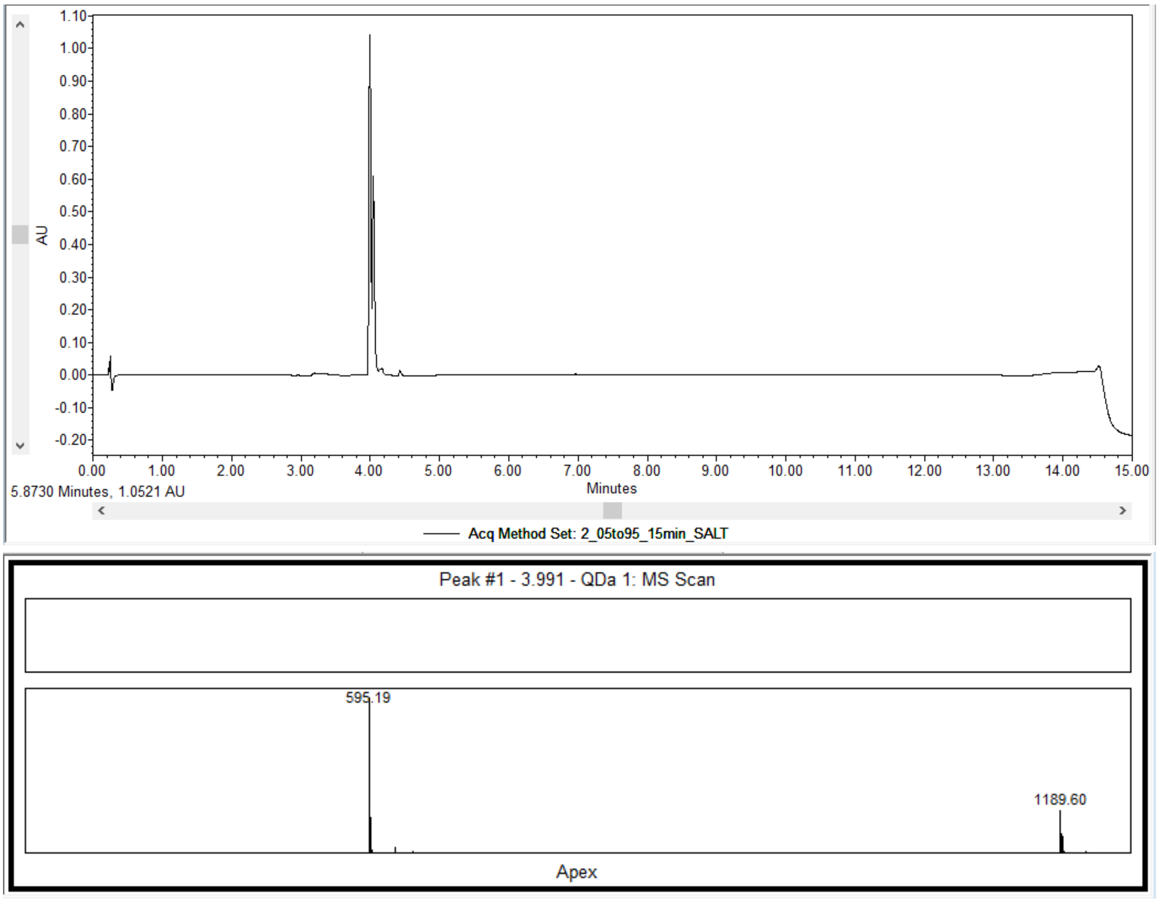

LC-MS spectrum of 17

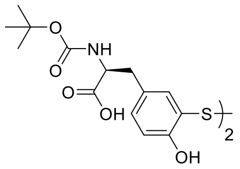

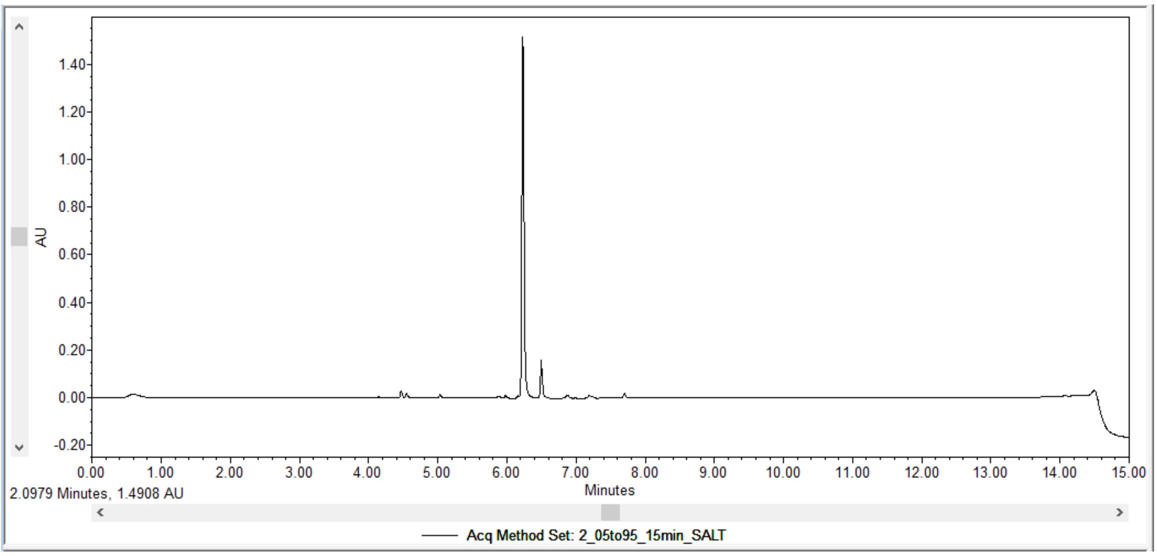

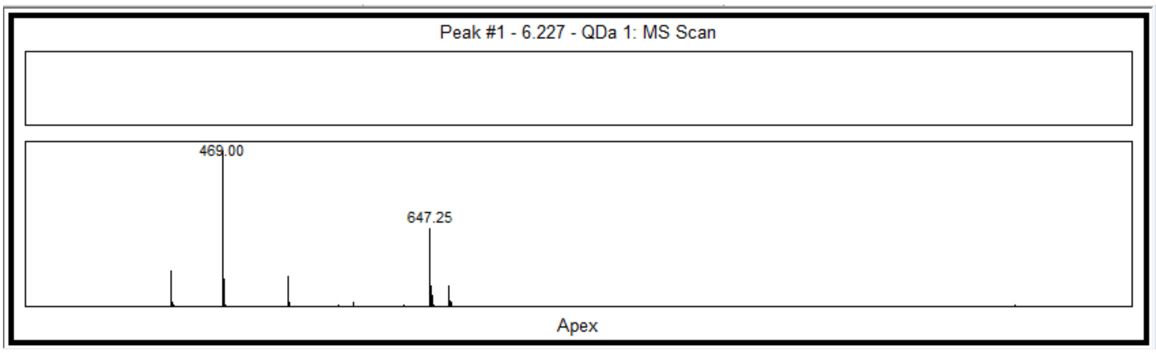

LC-MS spectrum of 13

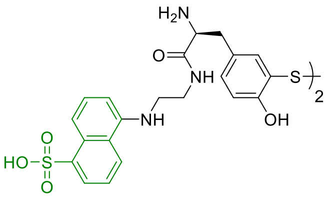

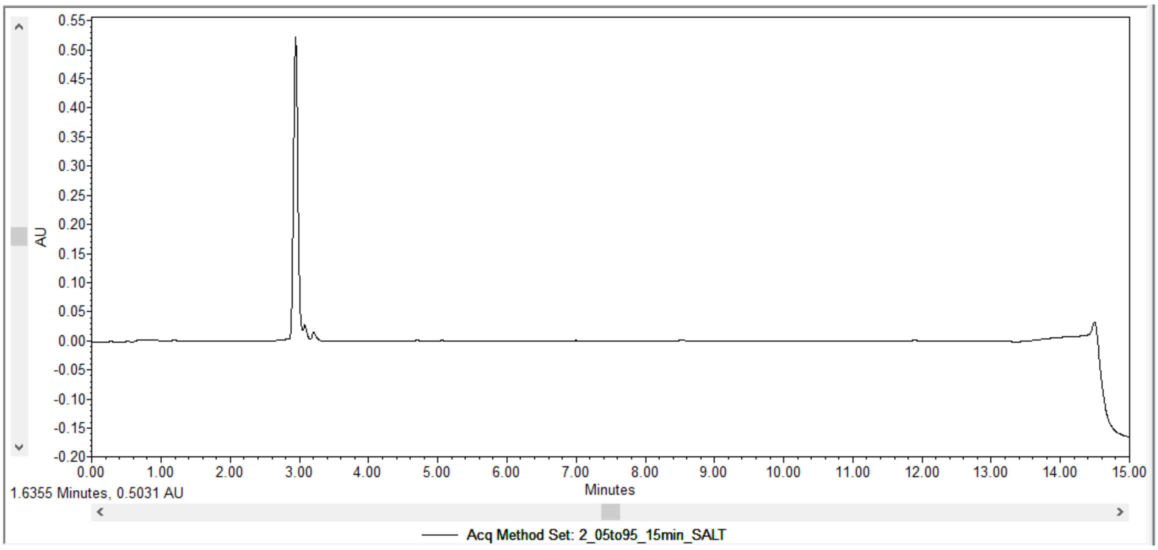

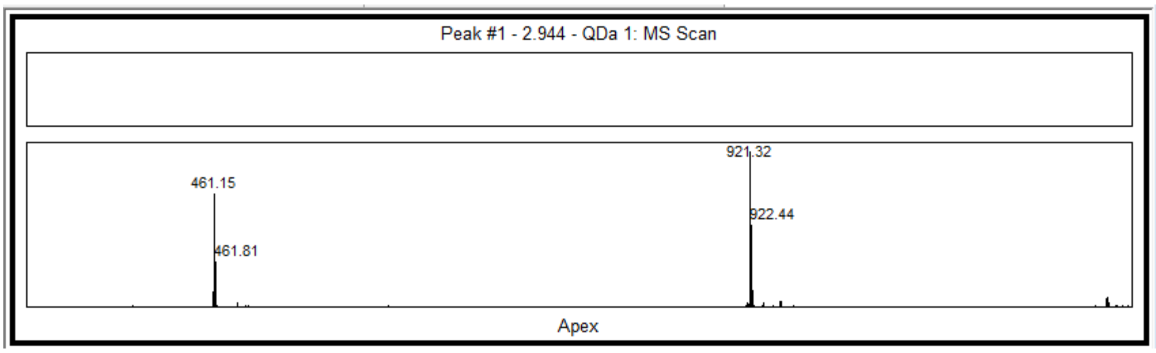

LC-MS spectrum of 19

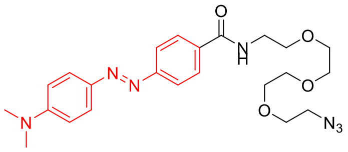

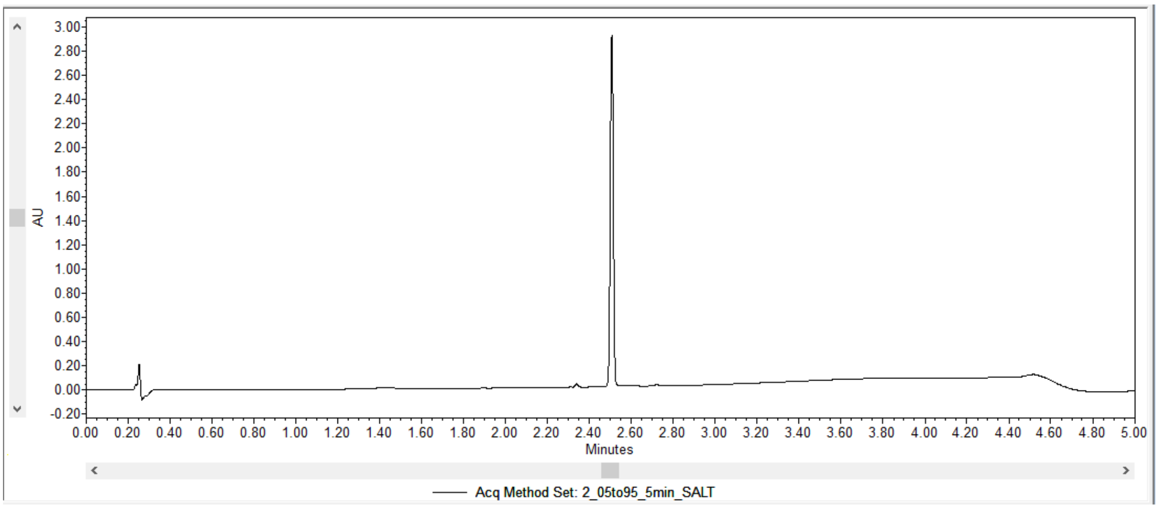

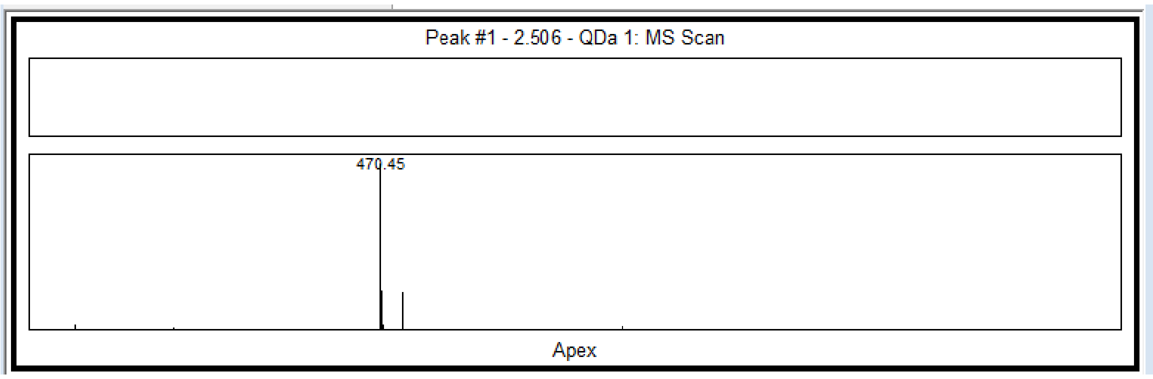

LC-MS spectrum of 15

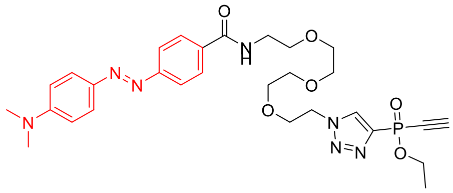

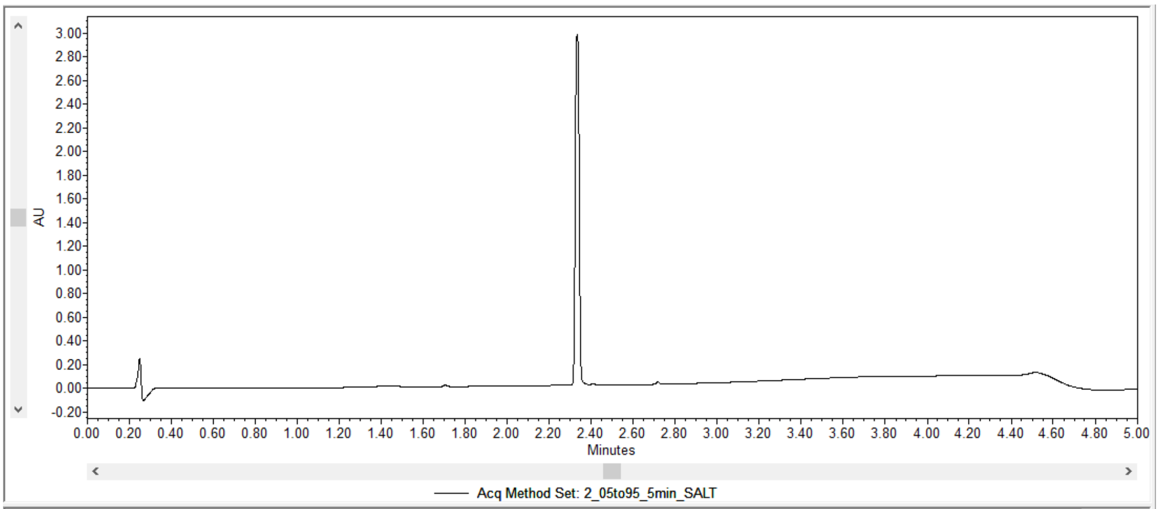

Name	Migration Time (min)	Area (µV*sec)	% Area	Height (µV)	Int Type	Amount	Units	Peak Type	Peak Codes	Corrected Area~ (µV*sec)	MS Match1 Score	MS Match2 Score	MS Match3 Score	Base Peak (Combined) (m/z)	Assigned Mass	Assigned Mass Value (m/z)	Notes

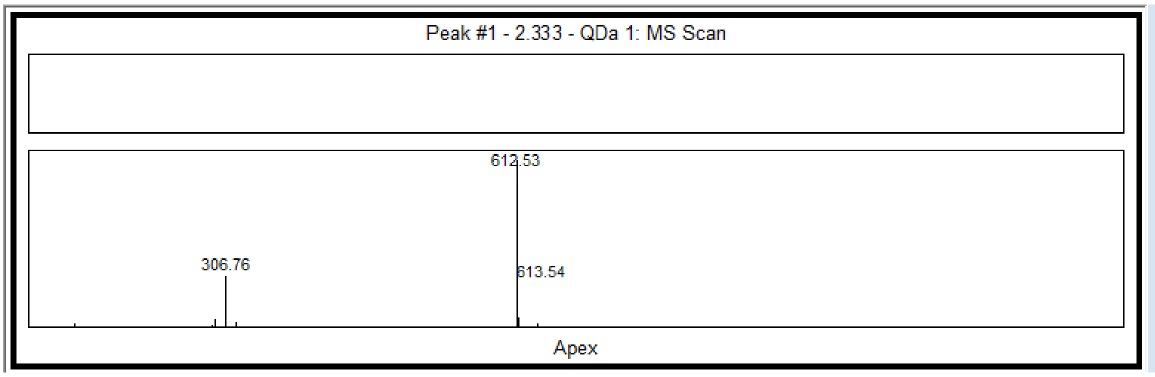

LC-MS spectrum of 16

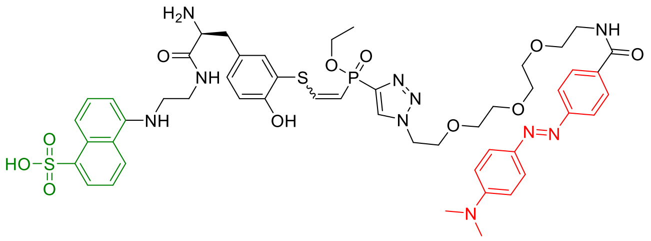

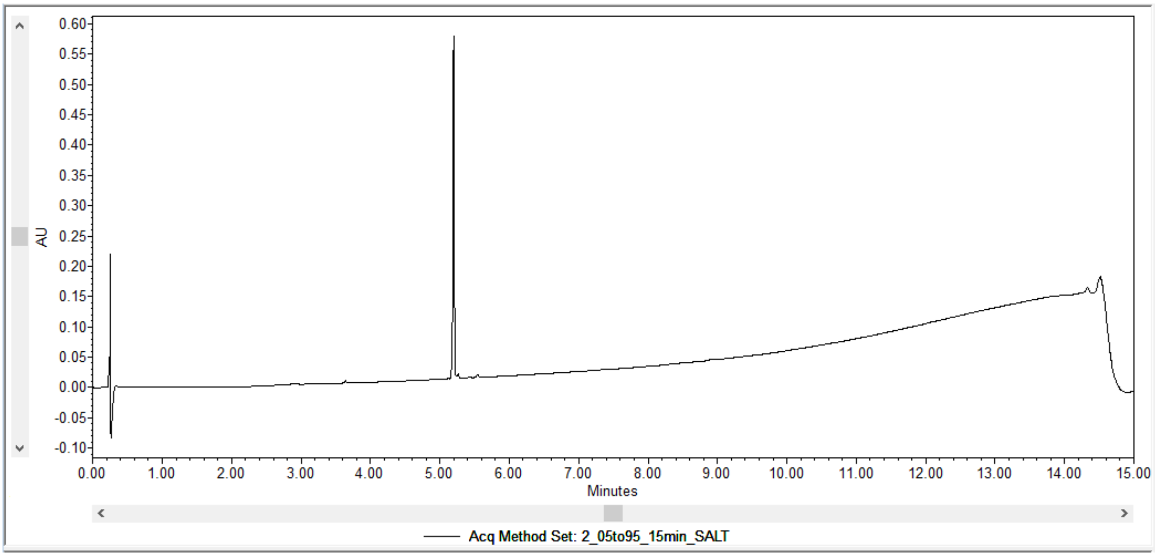

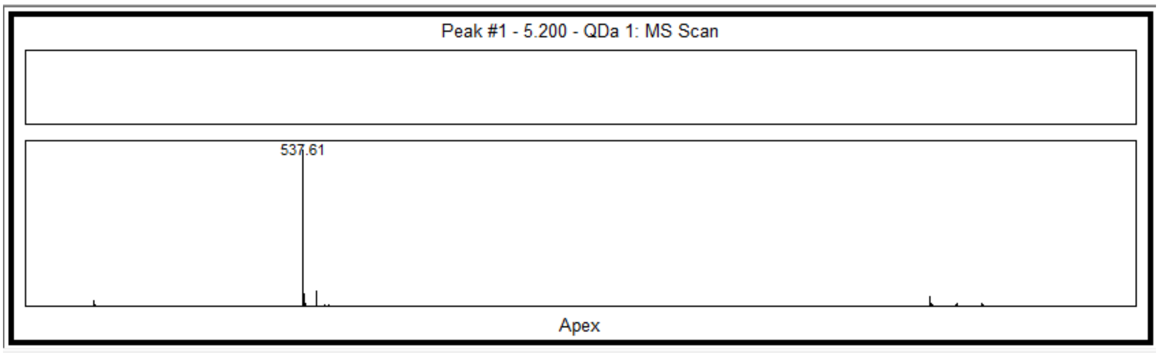

LC-MS spectrum of 20 [Crude reaction mixture]

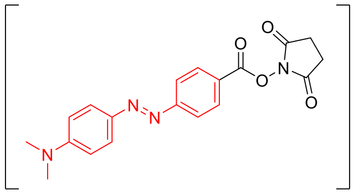

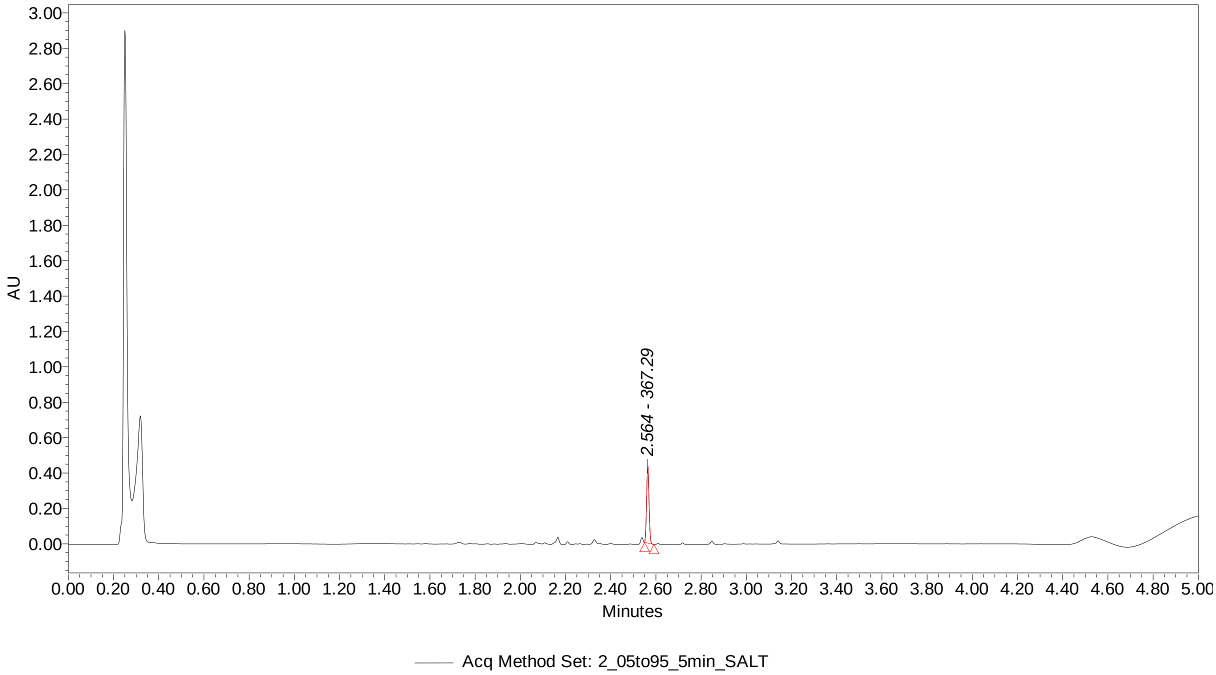

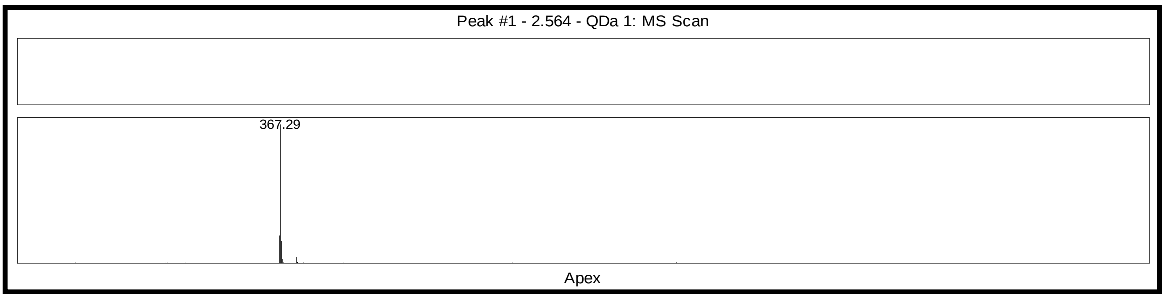

LC-MS spectrum of 21

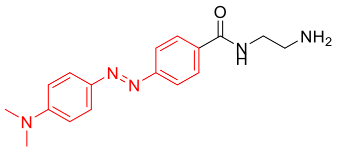

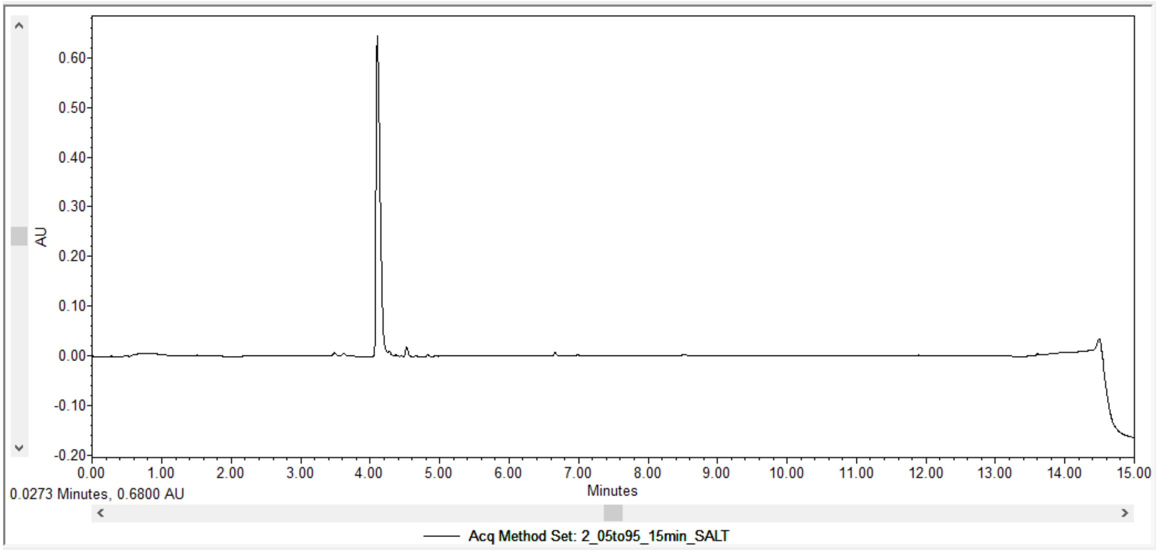

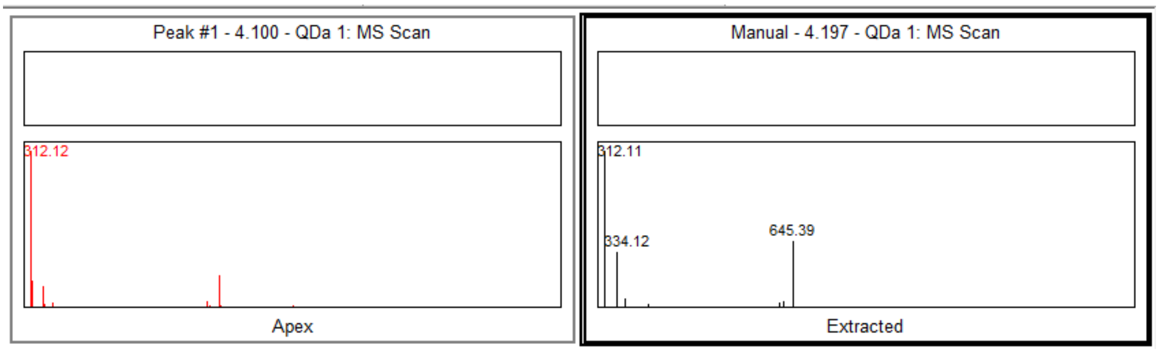

LC-MS spectrum of 23

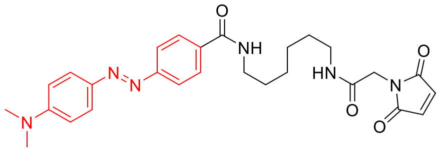

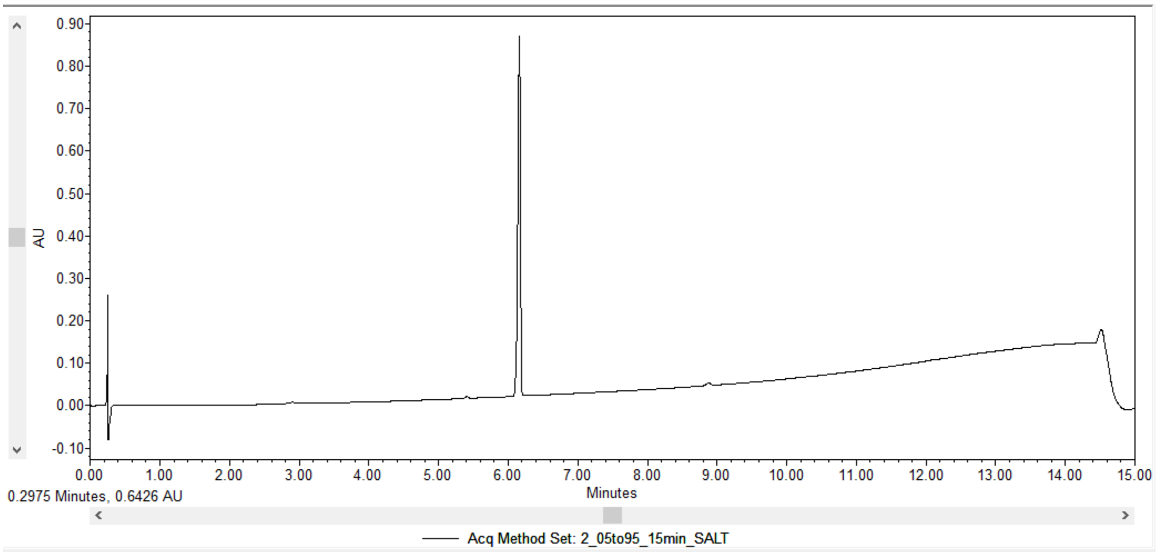

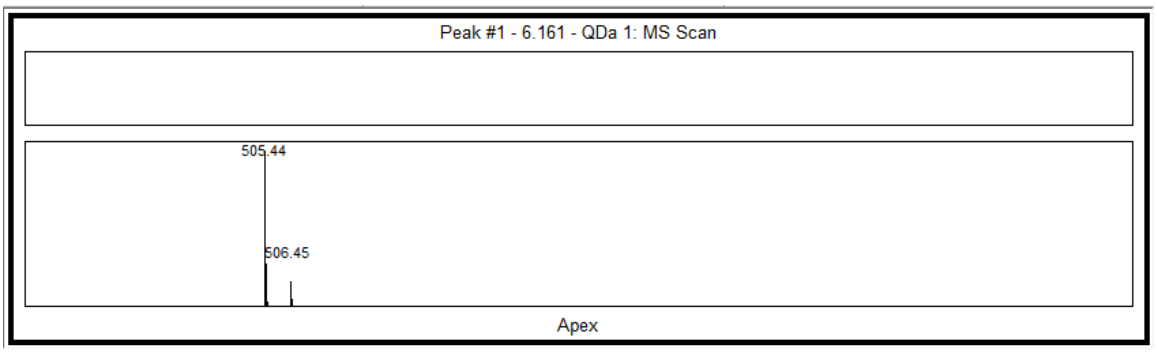

LC-MS spectrum of 24

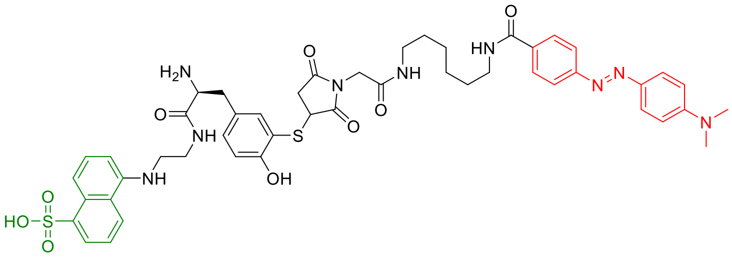

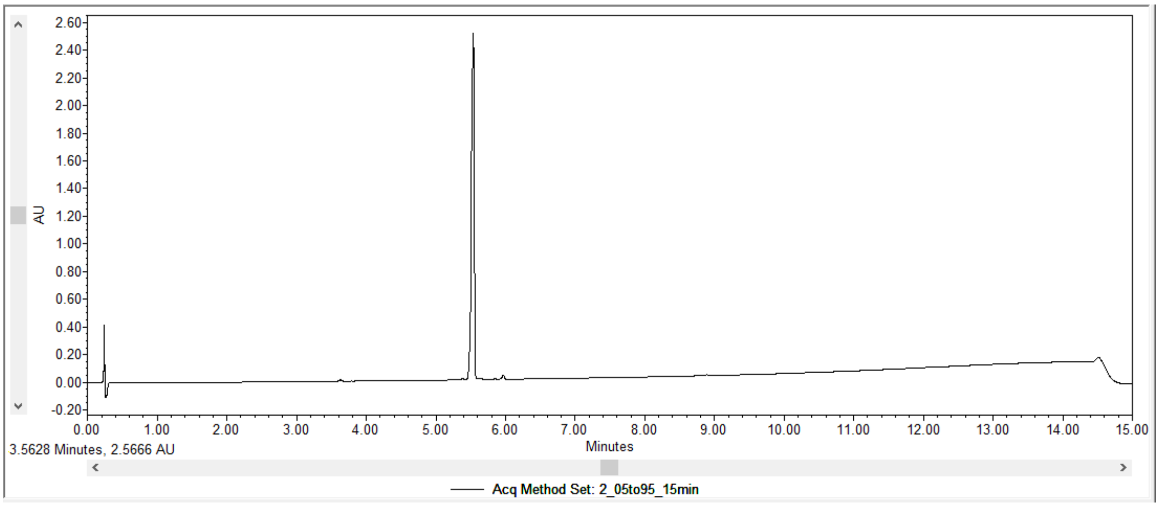

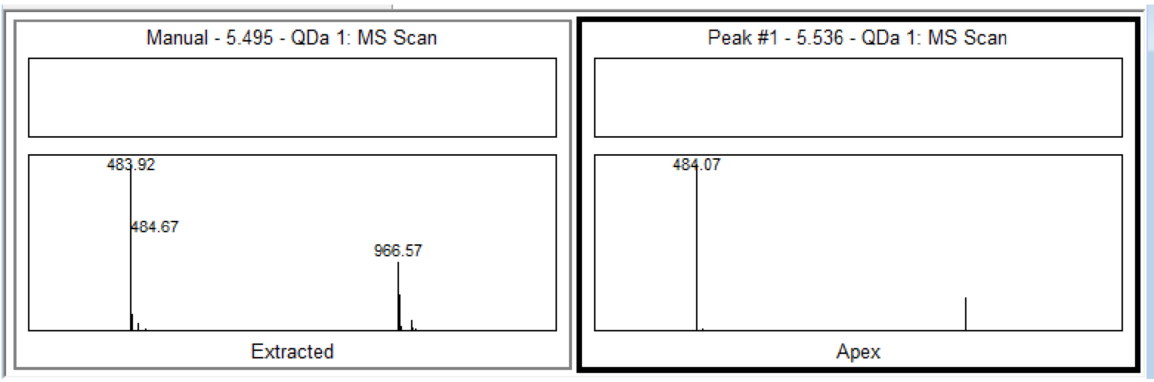

LC-MS spectrum of 29

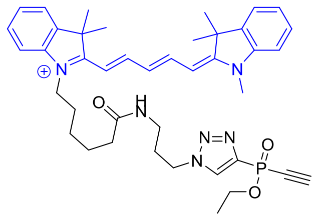

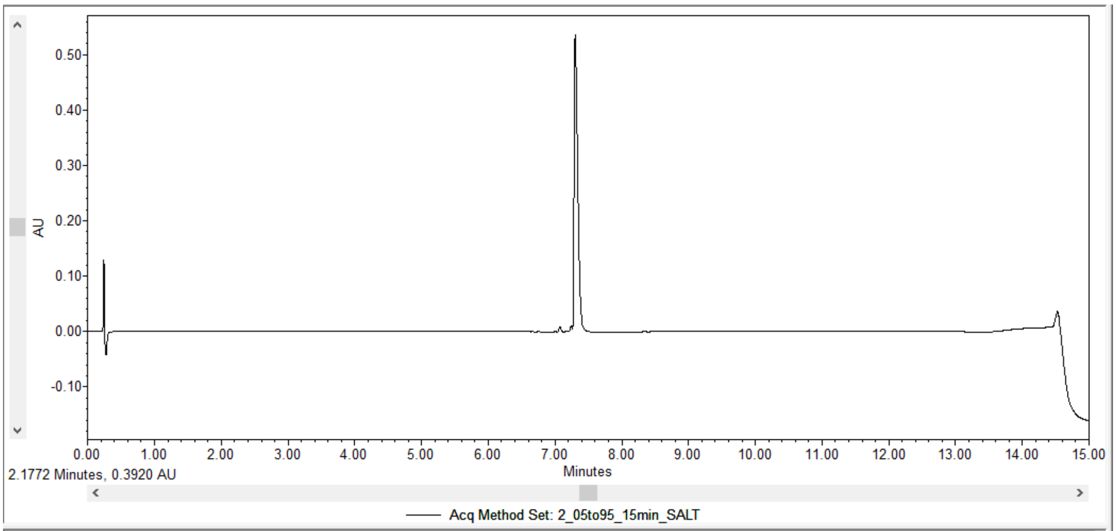

Name	Migration Time (min)	Area (µV*sec)	% Area	Height (µV)	Int Type	Amount	Units	Peak Type	Peak Codes	Corrected Area~ (µV*sec)	MS Match1 Score	MS Match2 Score	MS Match3 Score	Base Peak (Combined) (m/z)	Assigned Mass	Assigned Mass Value (m/z)	Notes

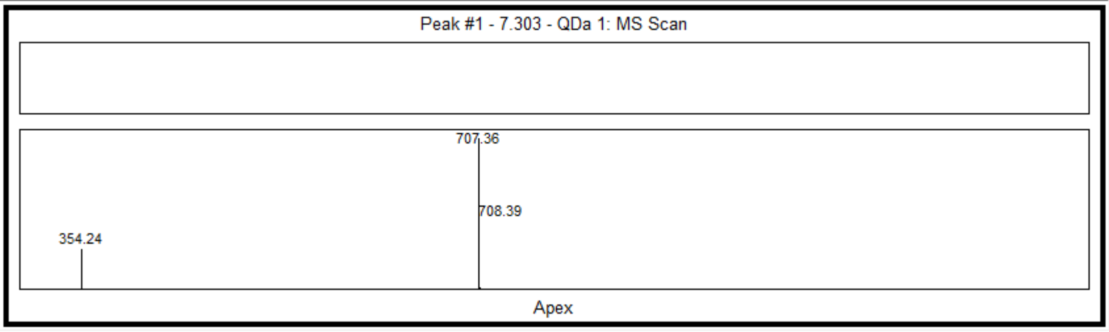

LC-MS spectrum of 30

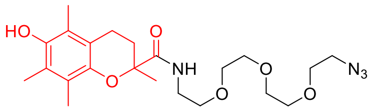

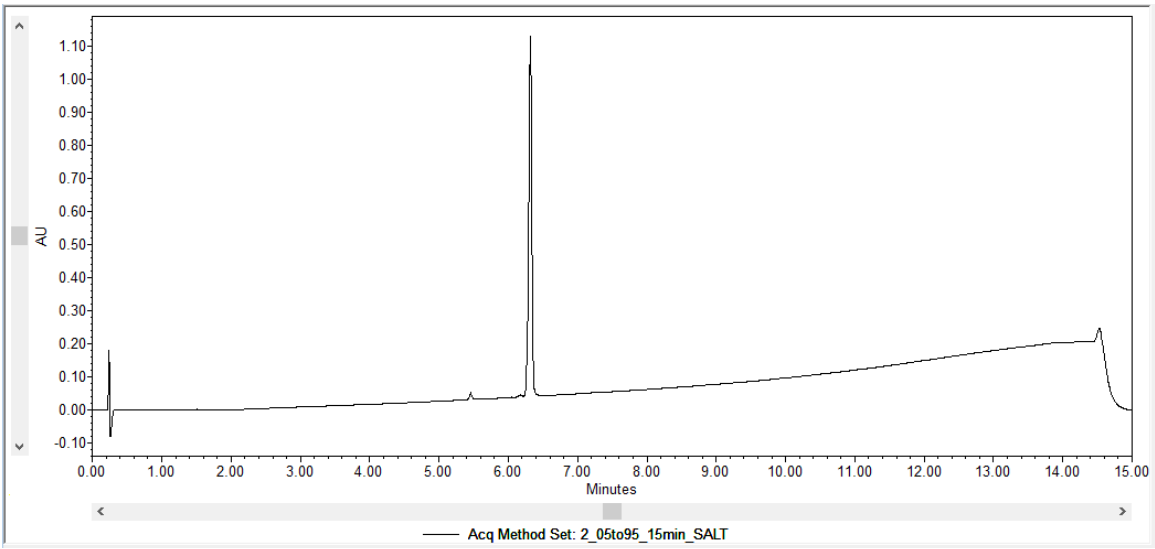

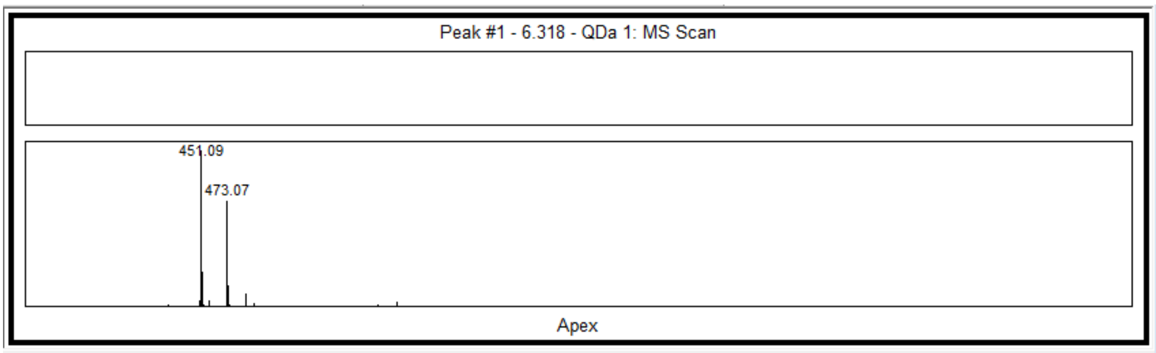

LC-MS spectrum of 31

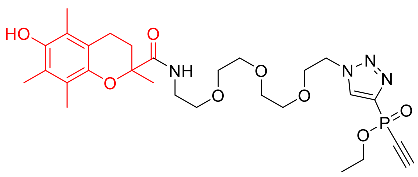

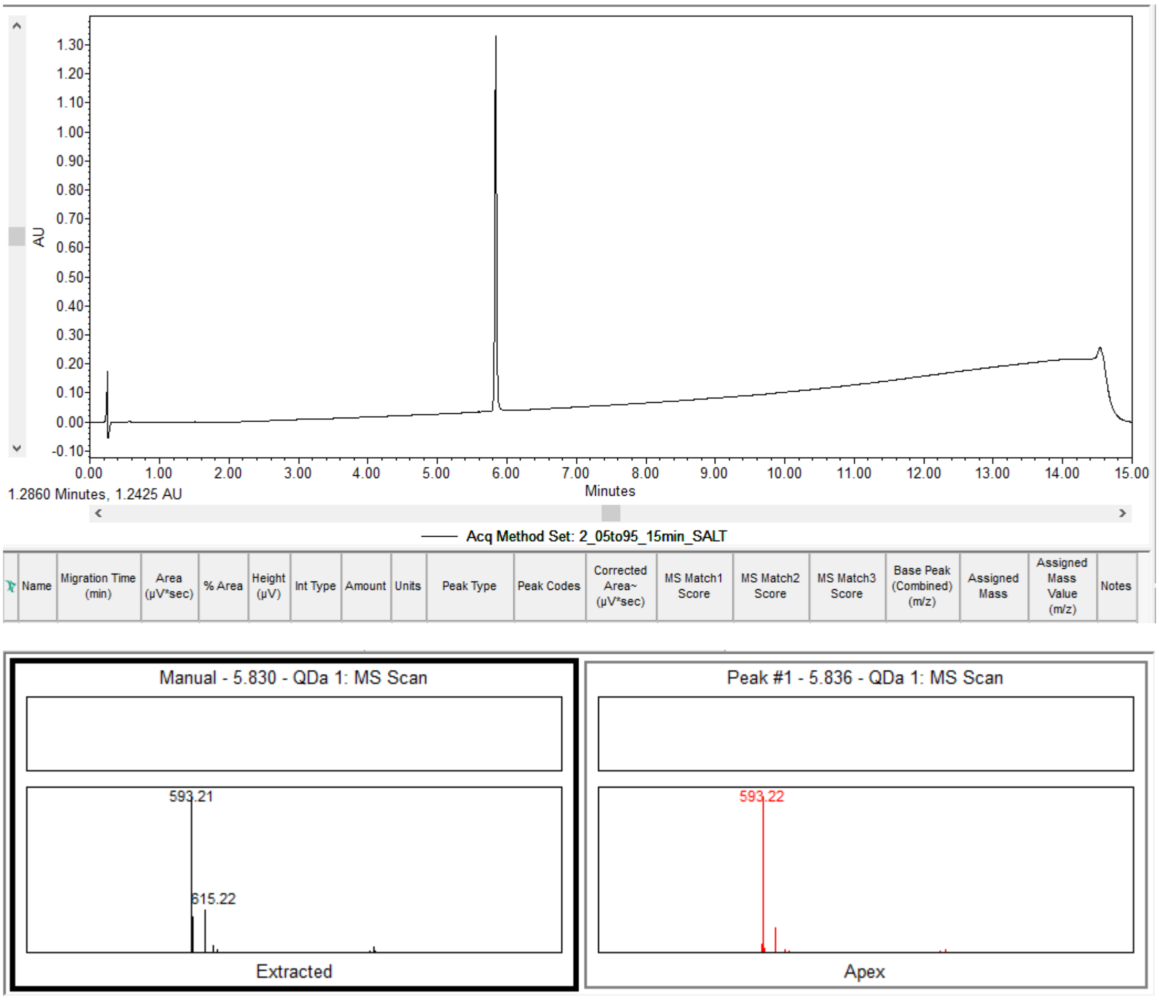

LC-MS spectrum of 35

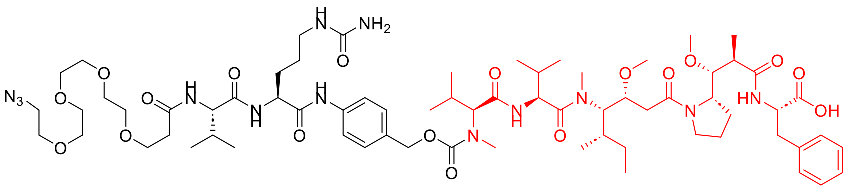

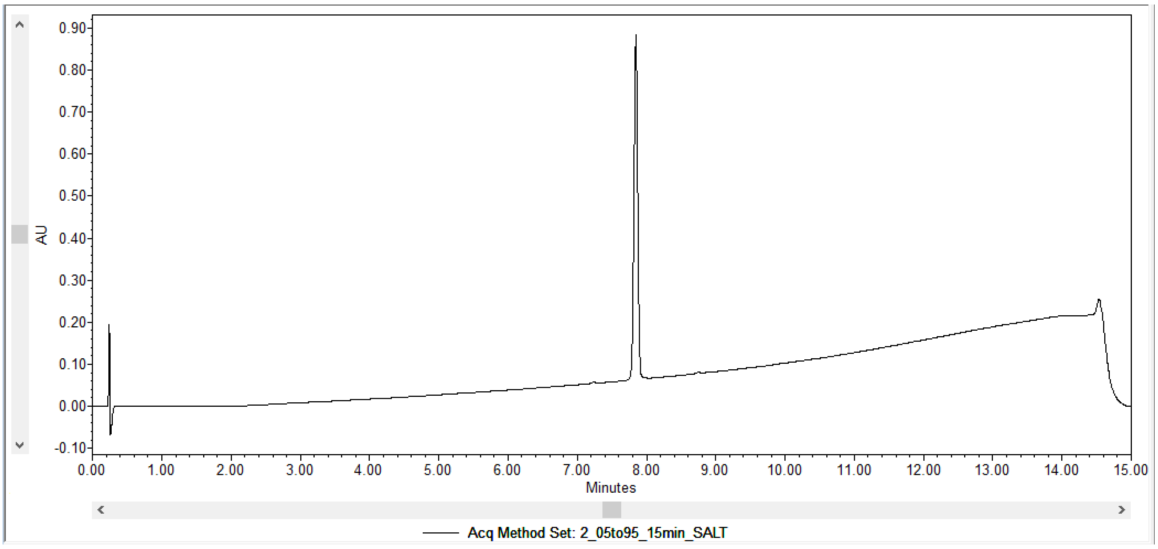

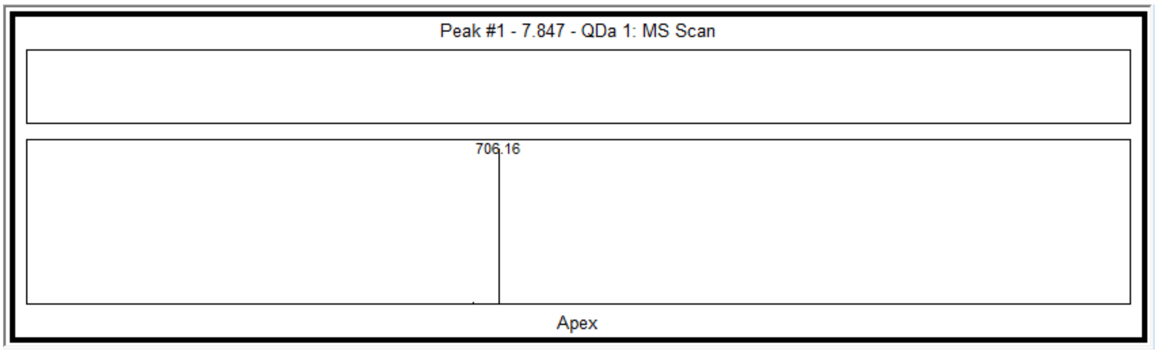

LC-MS spectrum of 36

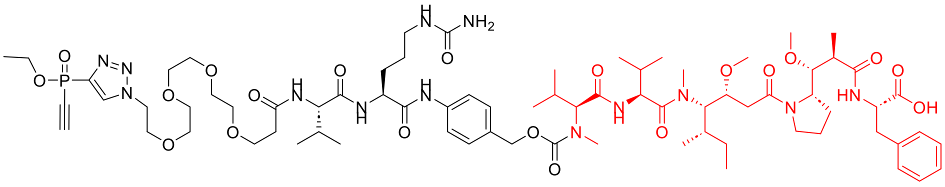

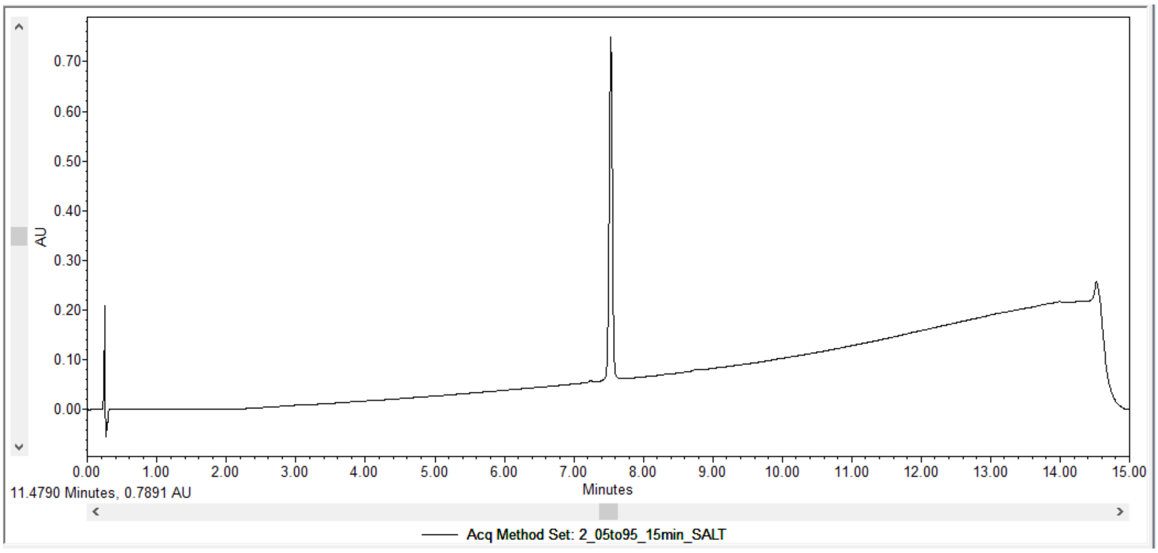

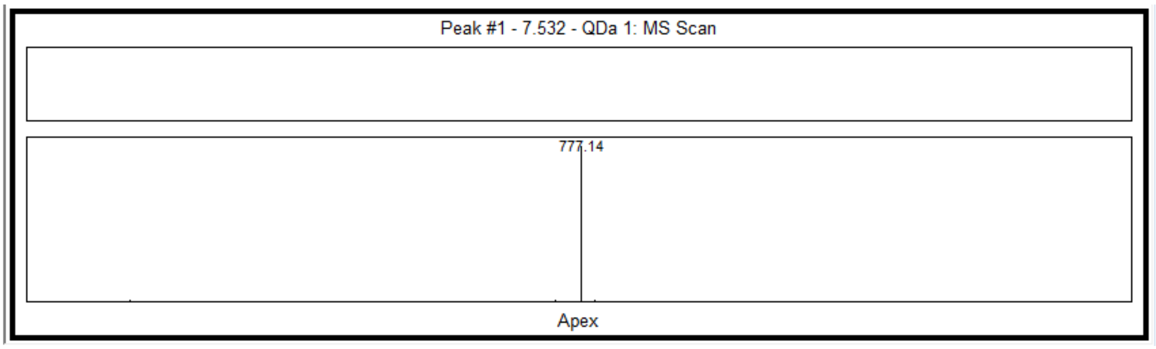

9. NMR spectra [Organic Synthesis]

^{1}H, ^{13}C, ^{1}H-COSY, (^{1}H,^{13}C)-HSQC, (^{1}H,^{13}C)-HMBC spectra of 3_{ab} (DMSO-d_6 – 310K)

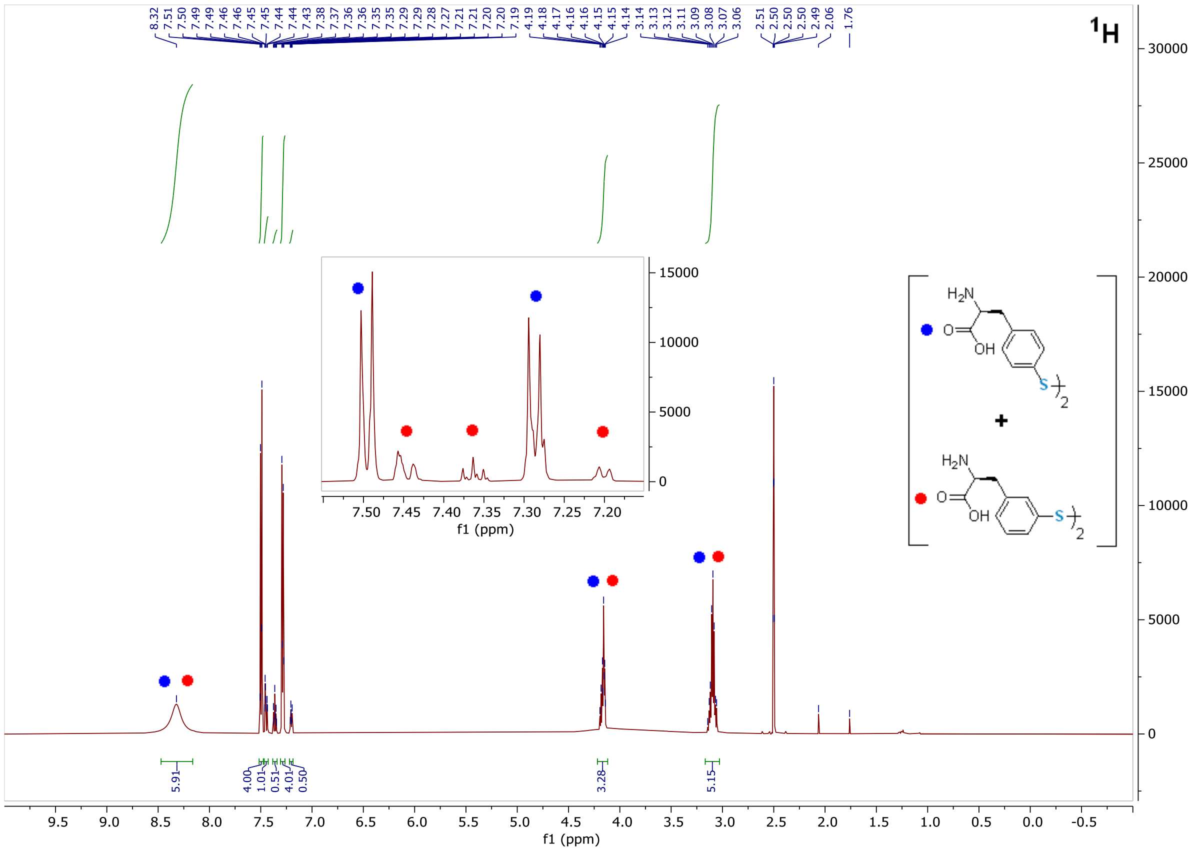

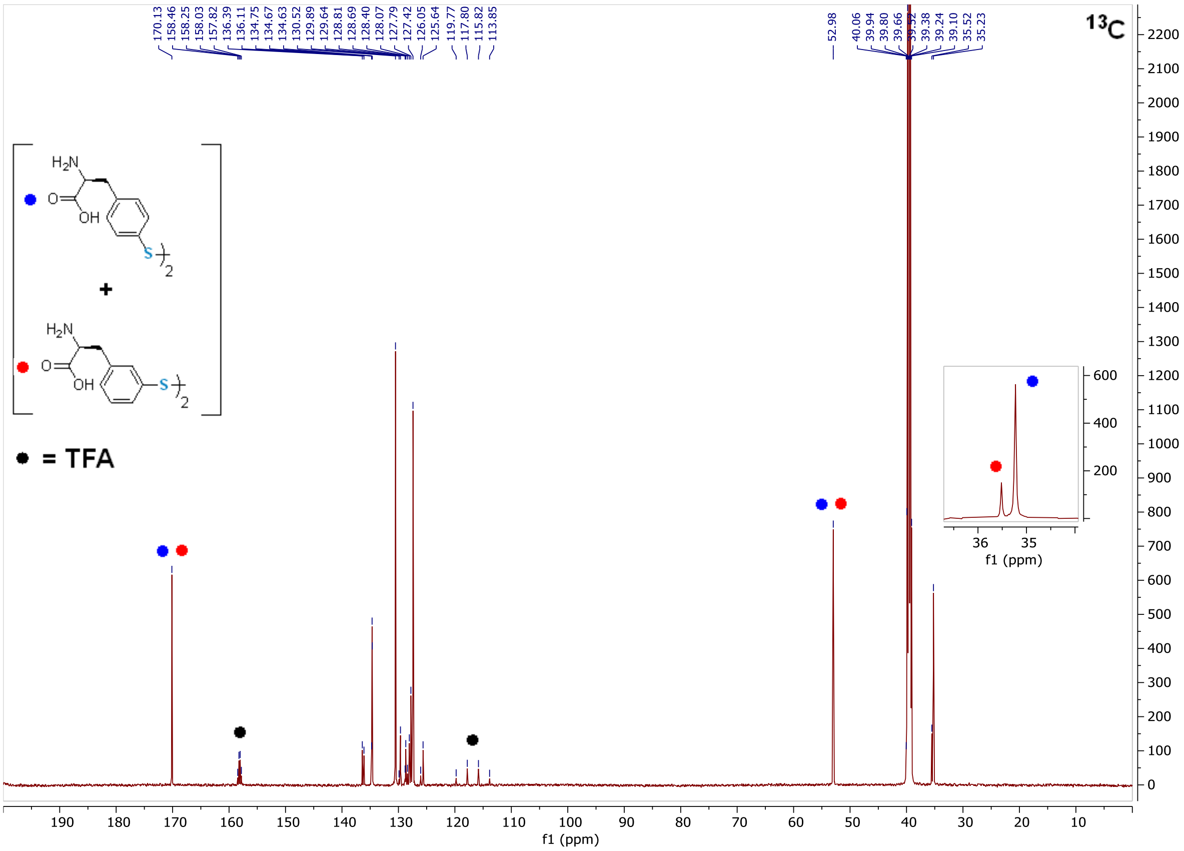
13C
170.13
158.46
158.25
158.03
157.82
136.39
136.11
134.75
134.67
134.63
130.52
129.89
129.64
128.81
128.69
128.40
128.07
127.79
127.42
126.05
125.64
119.77
117.80
115.82
113.85
52.98
40.06
39.94
39.80
39.66
39.52
39.38
39.24
39.10
35.52
35.23
H2N
O
OH
S
2
+
● = TFA
36
35
f1 (ppm)
f1 (ppm)

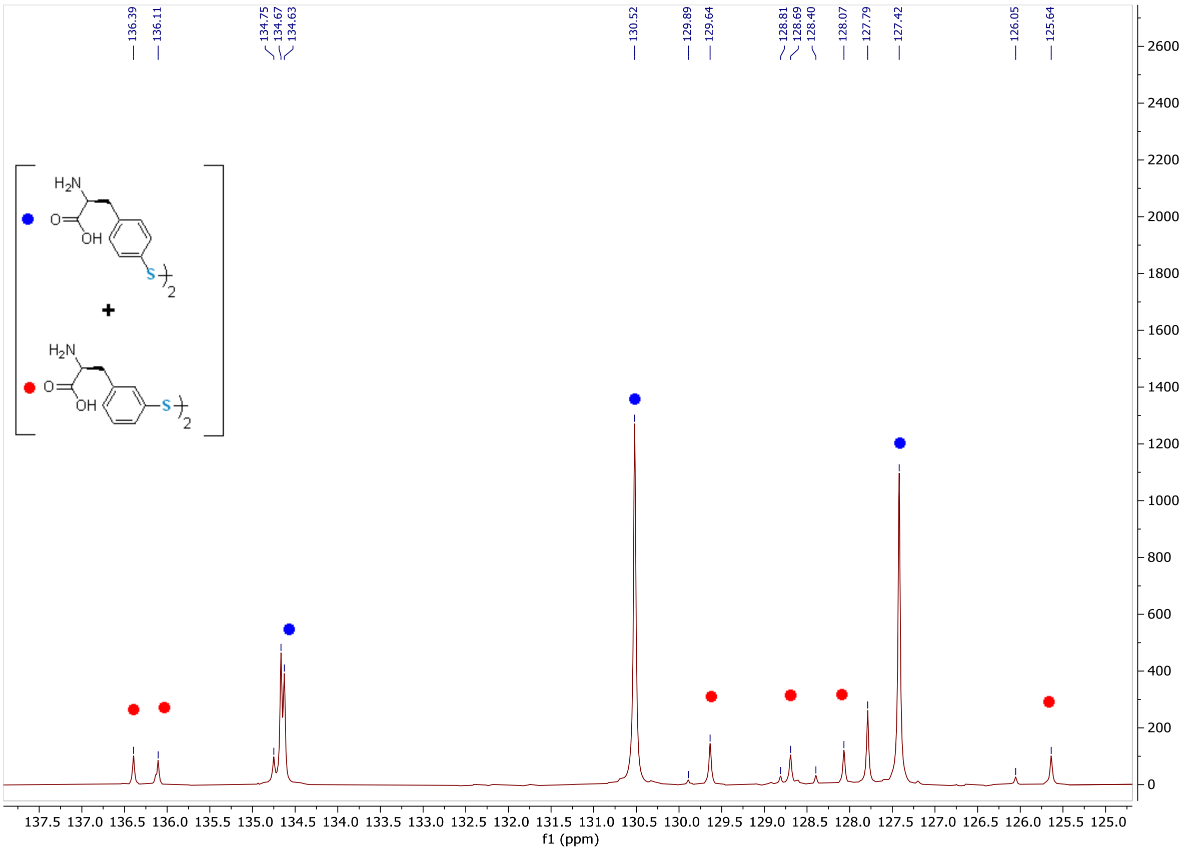
136.39
136.11
134.75
134.67
134.63
130.52
129.89
129.64
128.81
128.69
128.40
128.07
127.79
127.42
126.05
125.64
H2N
O
OH
S
2
+
f1 (ppm)

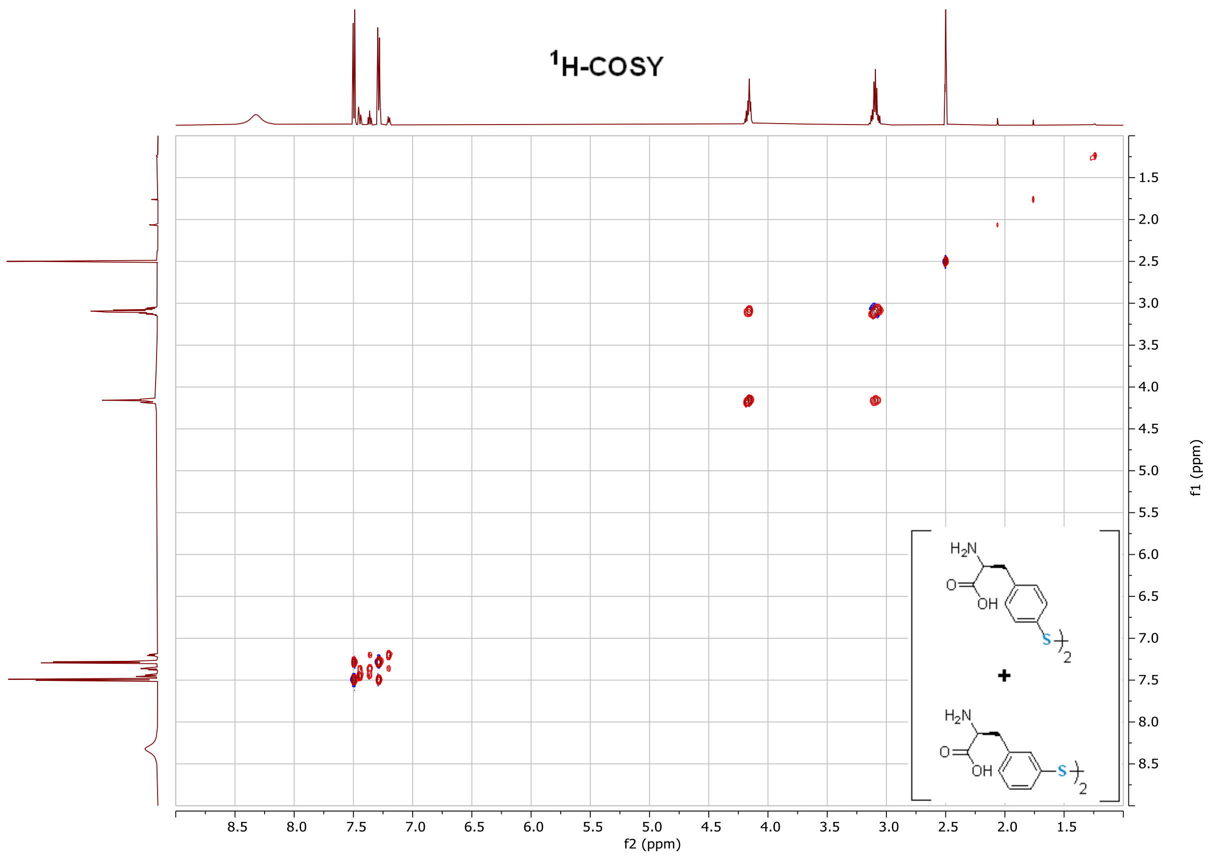

¹H-COSY
f2 (ppm)
f1 (ppm)
H2N
O
OH
S
2
+

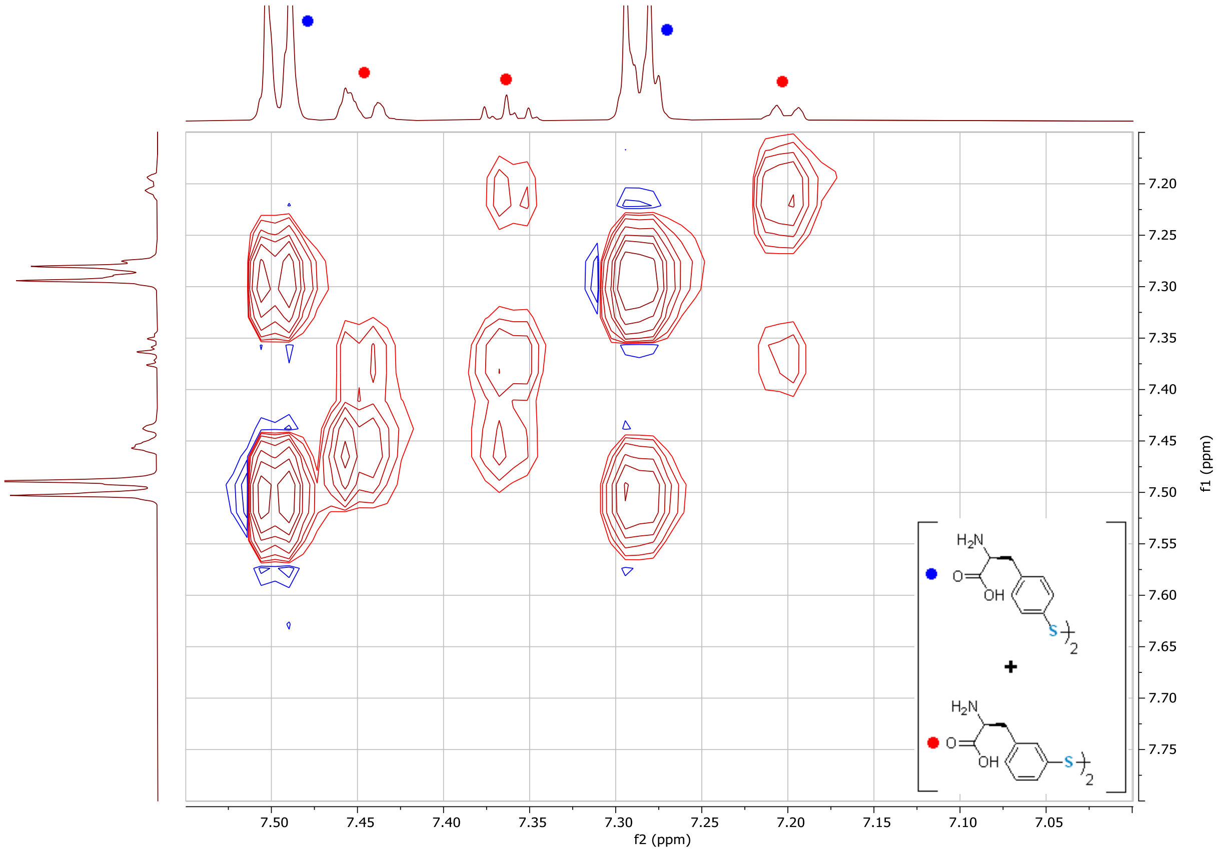

f2 (ppm)
f1 (ppm)
H2N
O
OH
S
2
+

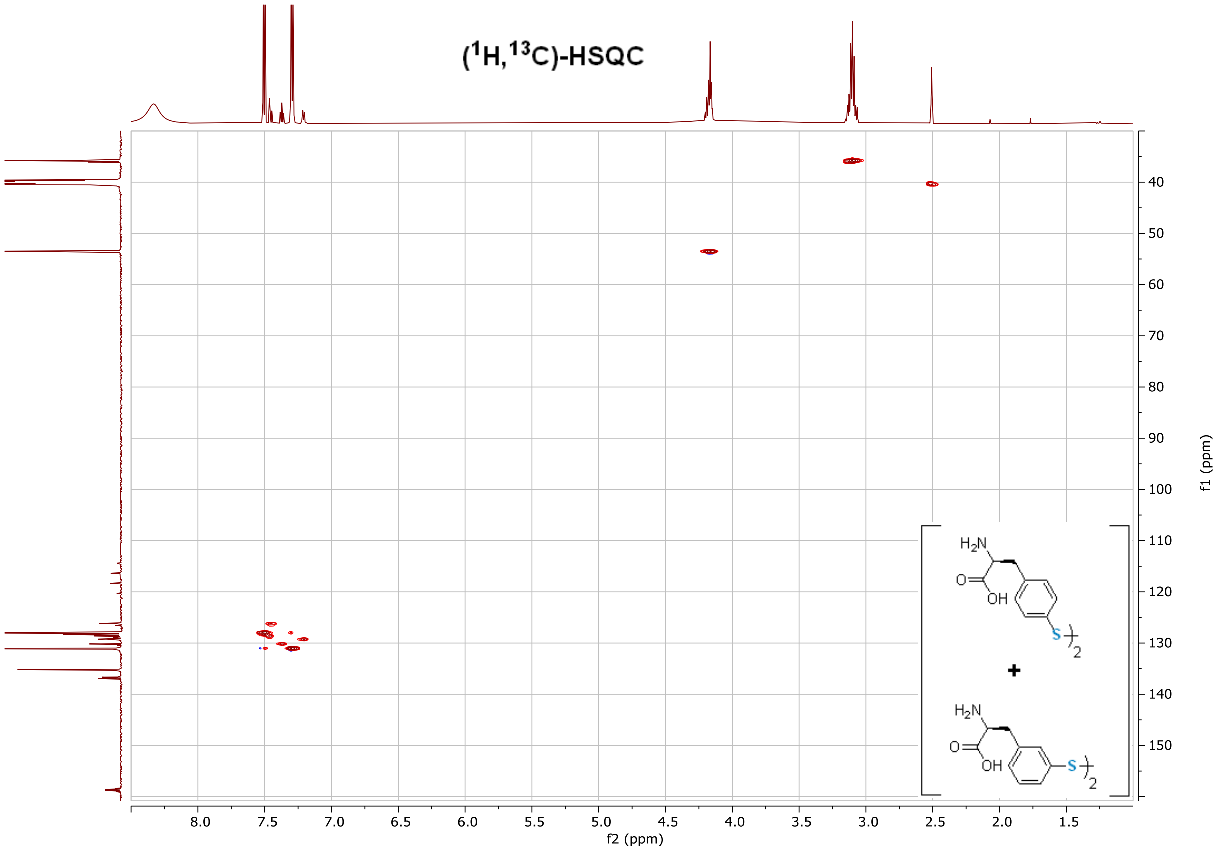

(¹H,¹³C)-HSQC
f2 (ppm)
f1 (ppm)
H2N
OH
O
S
2

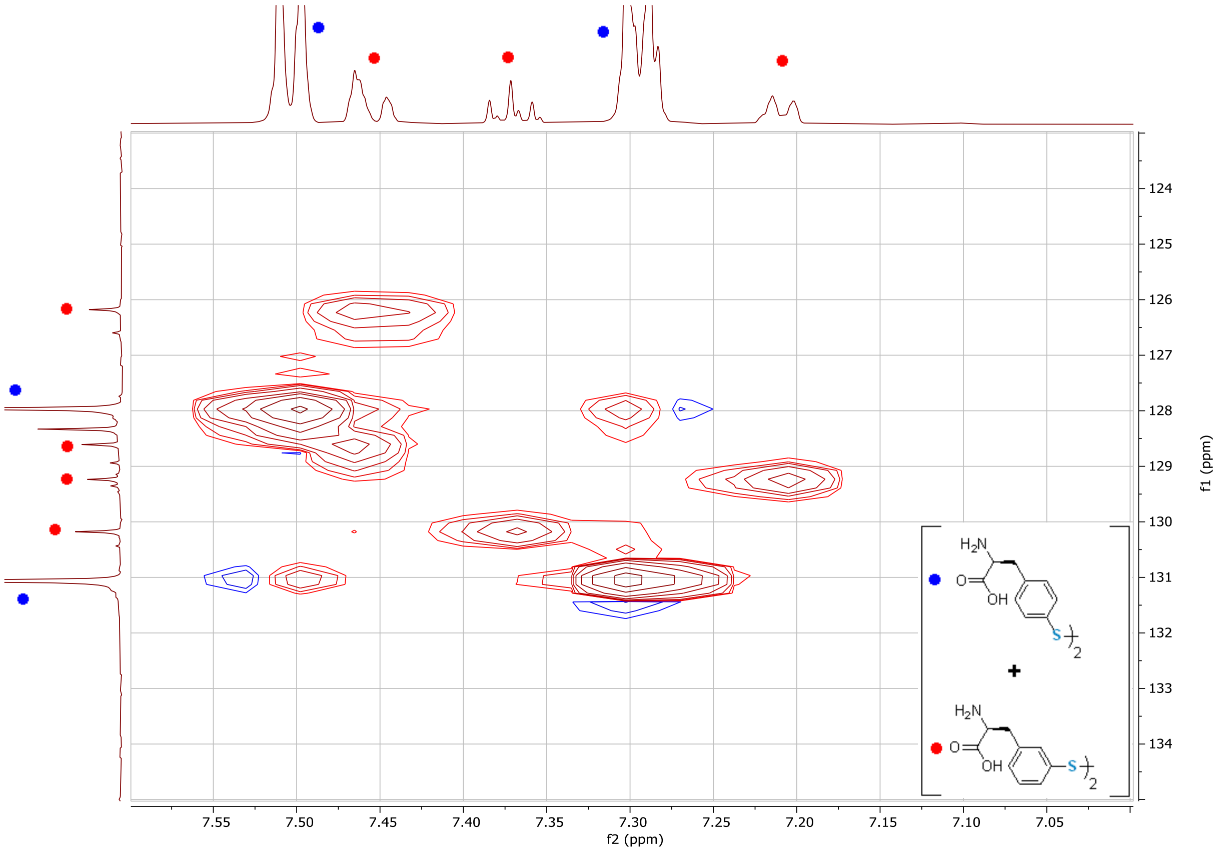

f2 (ppm)
f1 (ppm)
H2N
OH
O
S
2

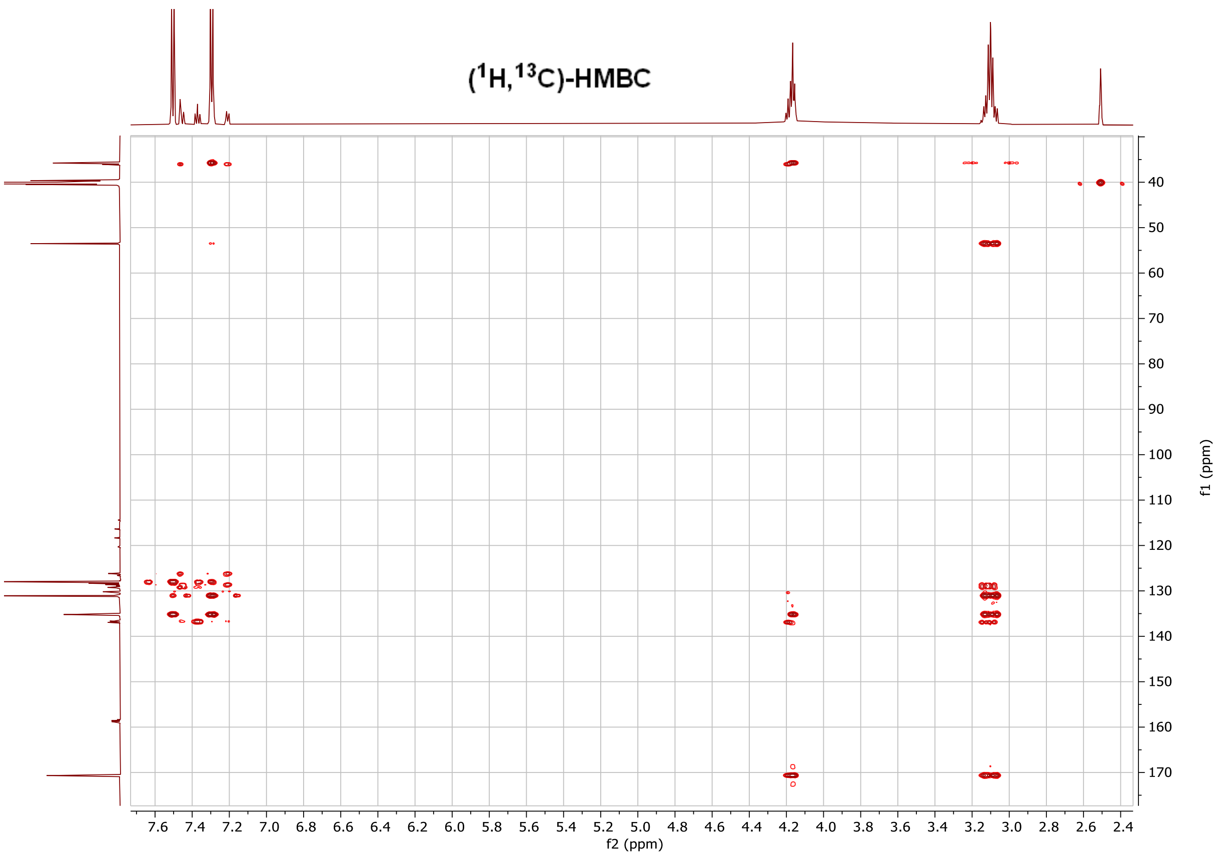
(¹H,¹³C)-HMBC
f1 (ppm)
f2 (ppm)

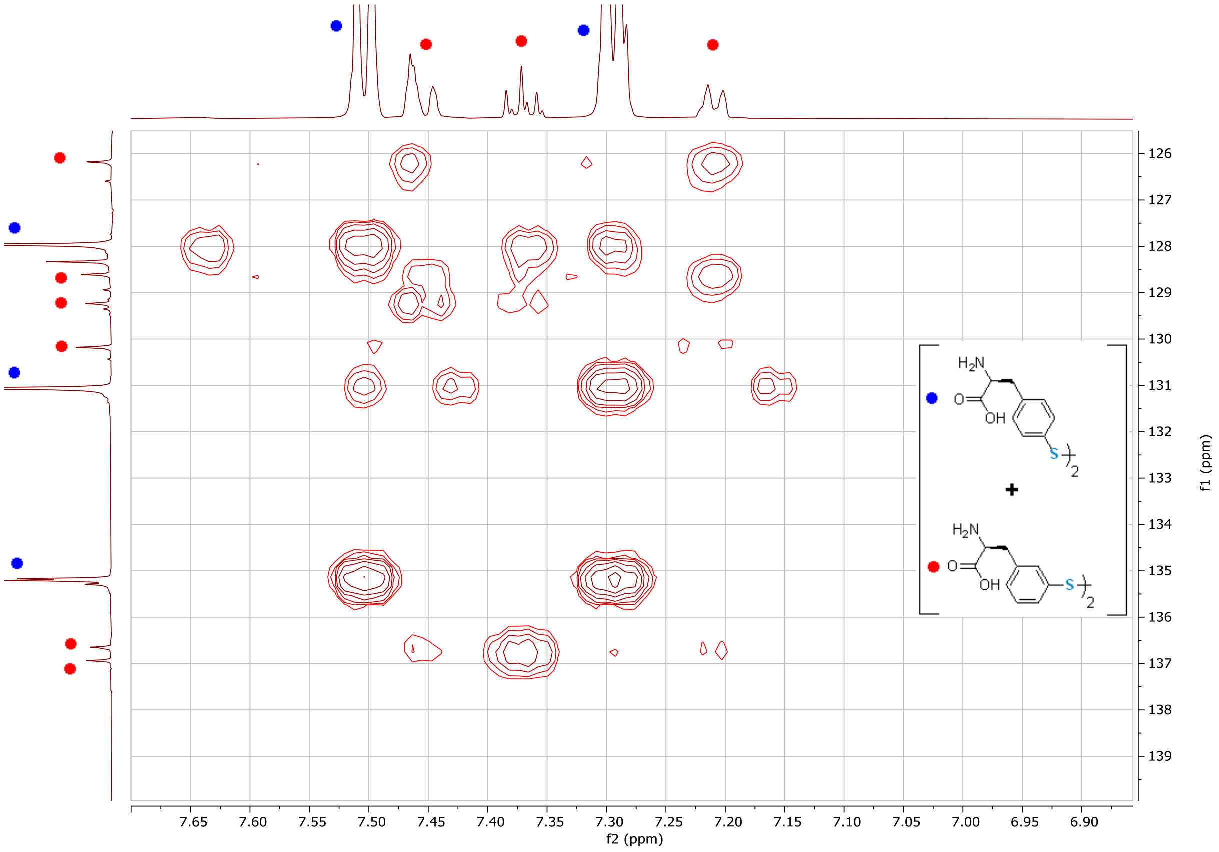
f1 (ppm)
f2 (ppm)
H2N
O
OH
S
2
+
H2N
O
OH
S
2

^{1}H, ^{13}C-APT, ^{1}H-COSY and (^{1}H,^{13}C)-HSQC spectra of 6 (D_2O)

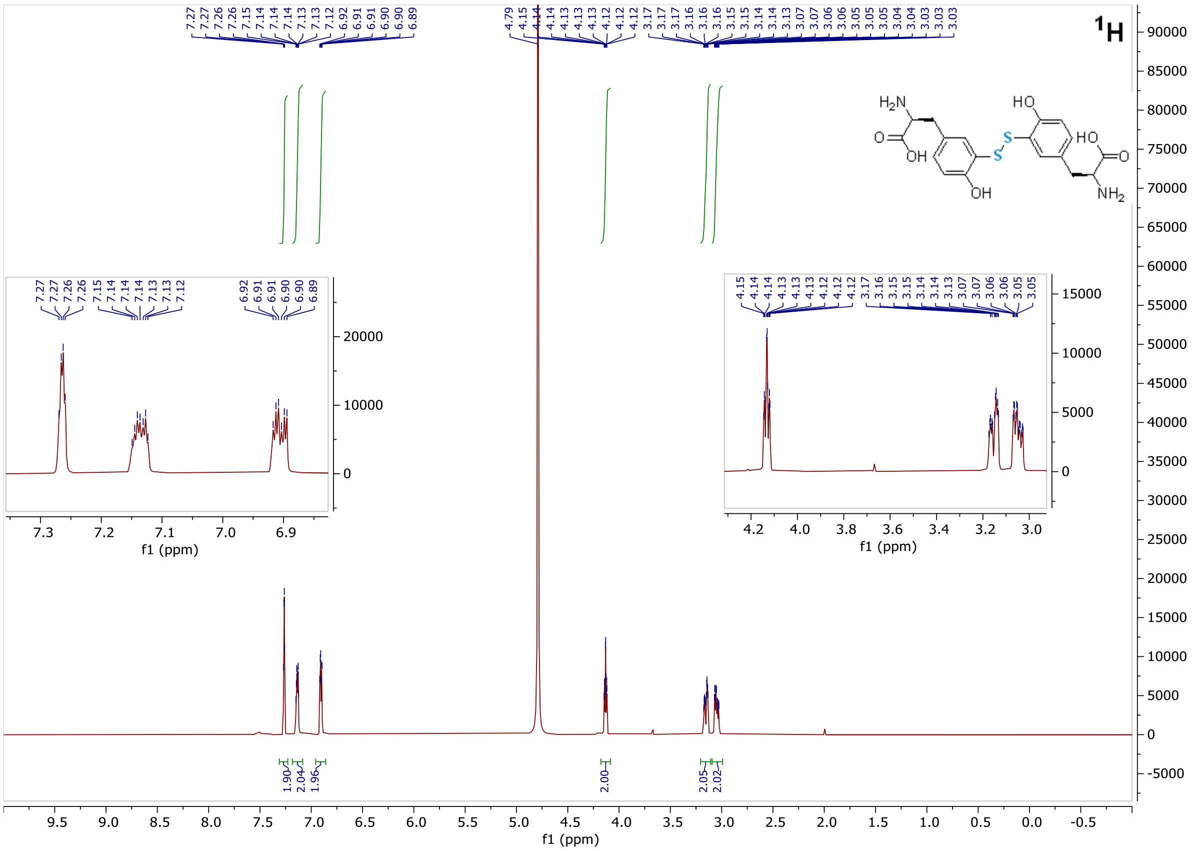

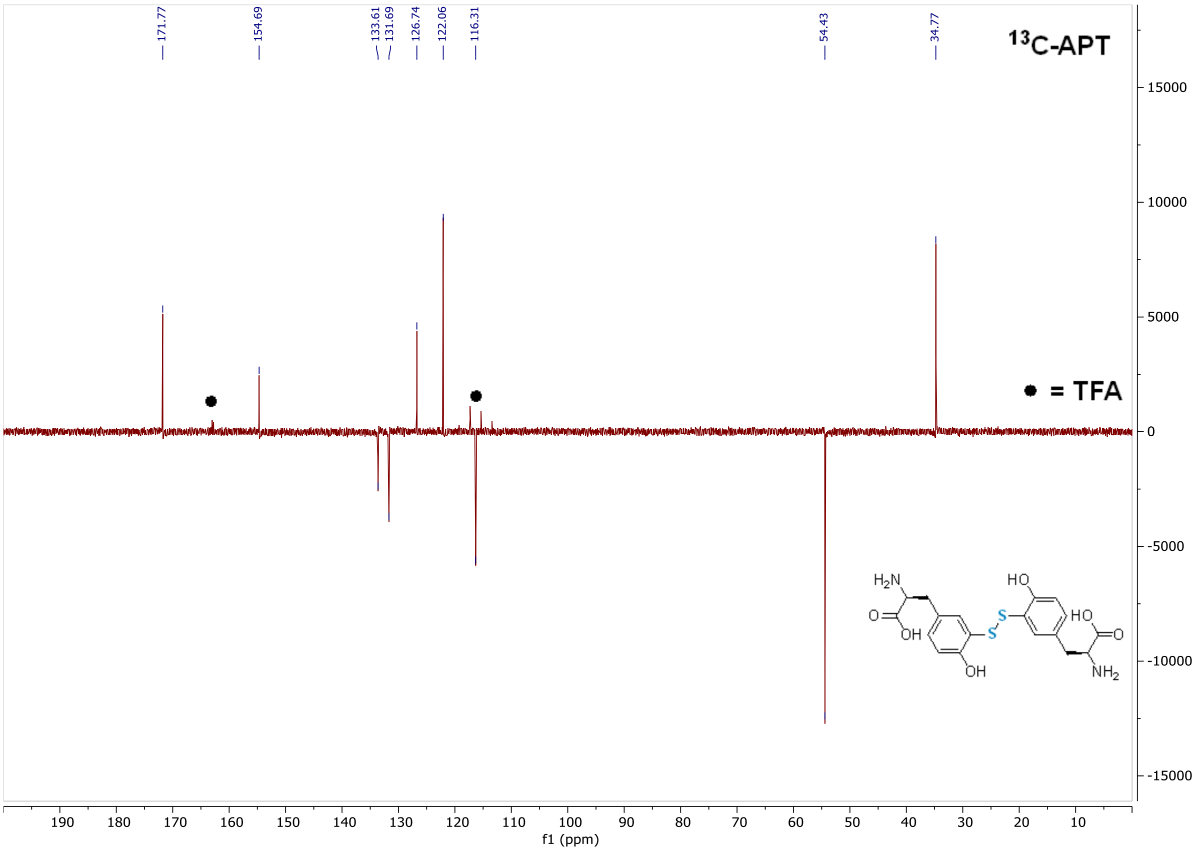

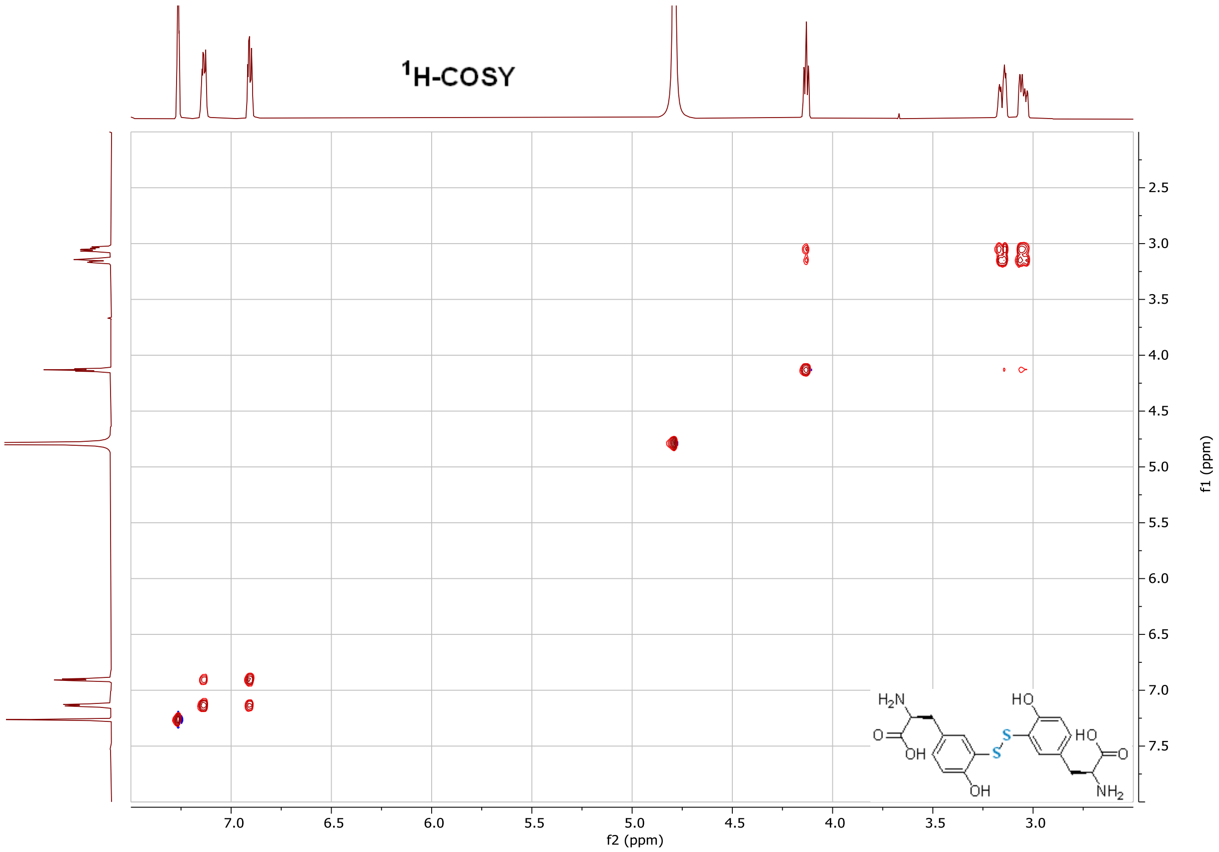
1H-COSY
f2 (ppm)
f1 (ppm)
H2N
HO
O
OH
S
S
HO
O
OH
NH2

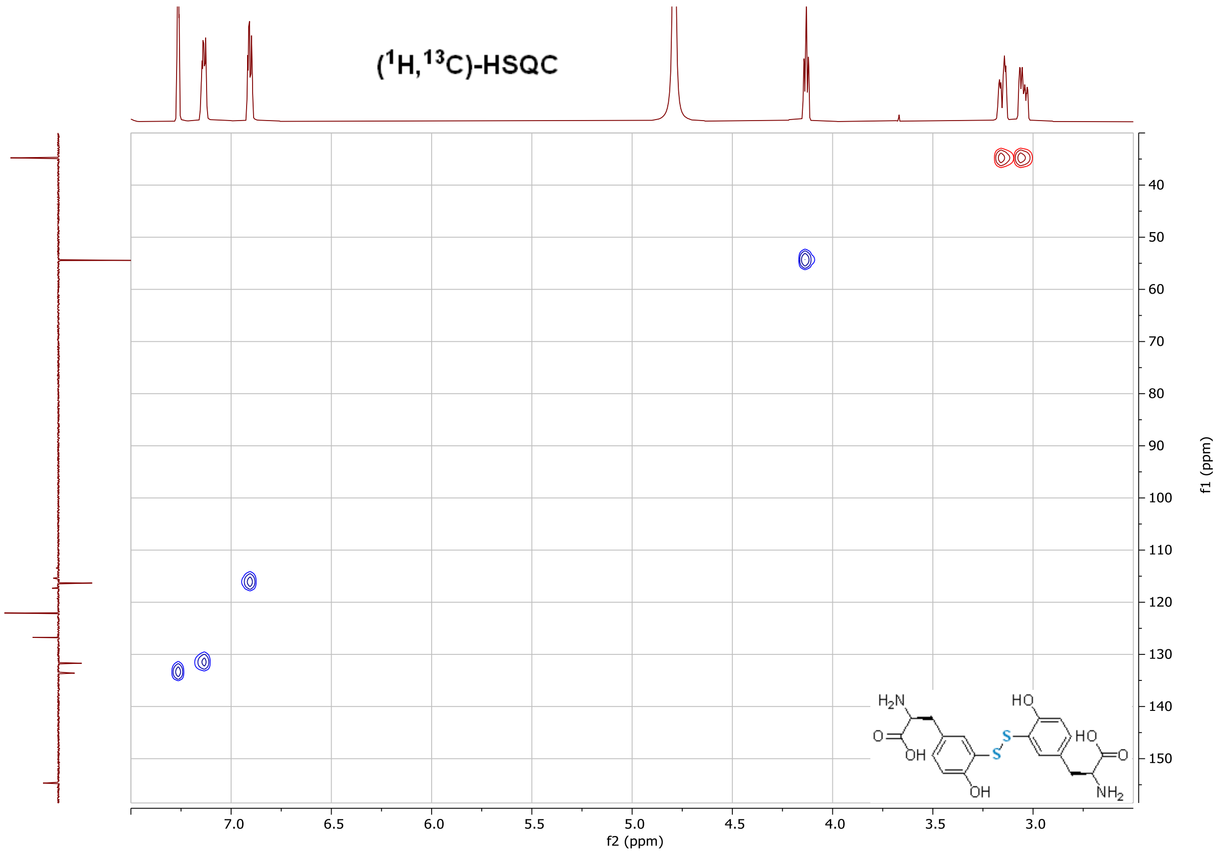
(1H,13C)-HSQC
f2 (ppm)
f1 (ppm)
H2N
HO
O
OH
S
S
HO
O
OH
NH2

^{1}H, ^{13}C-APT, ^{1}H-COSY and (^{1}H,^{13}C)-HSQC spectra of 6 (DMSO-d$_6$)

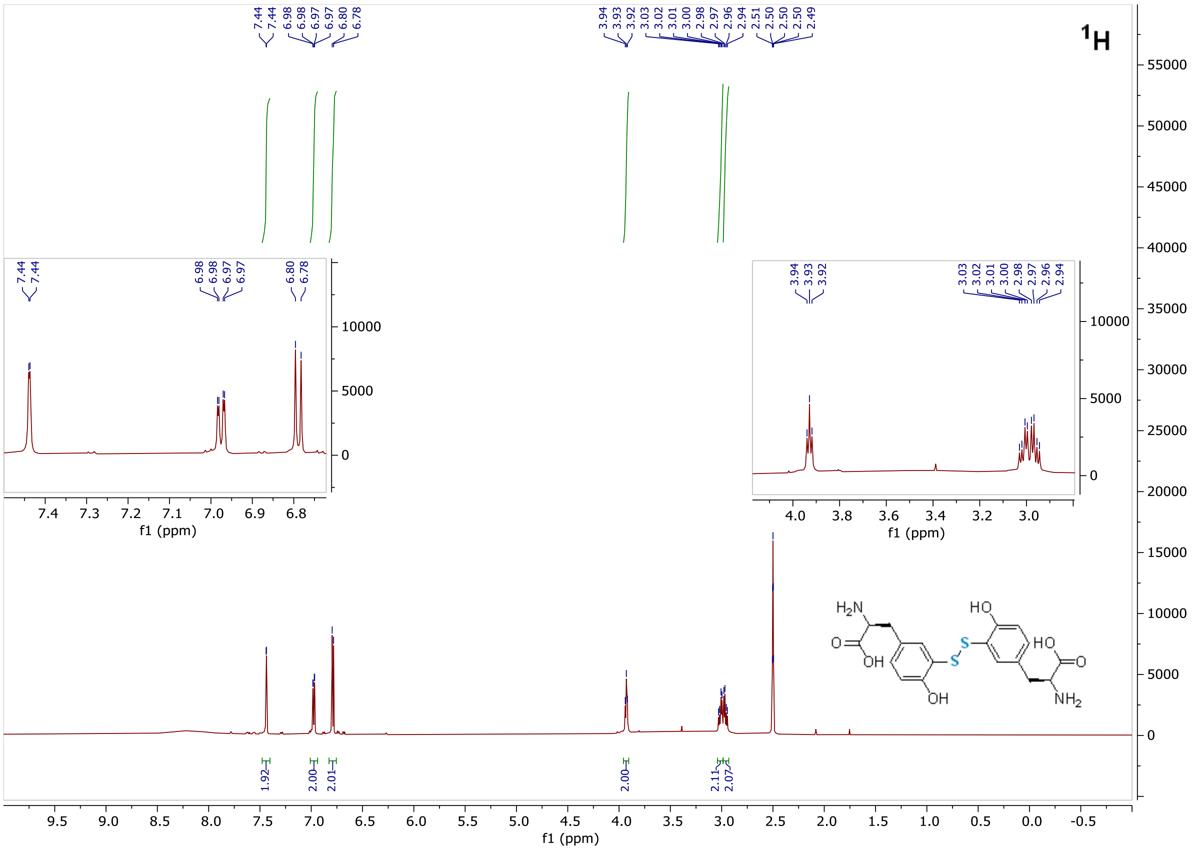

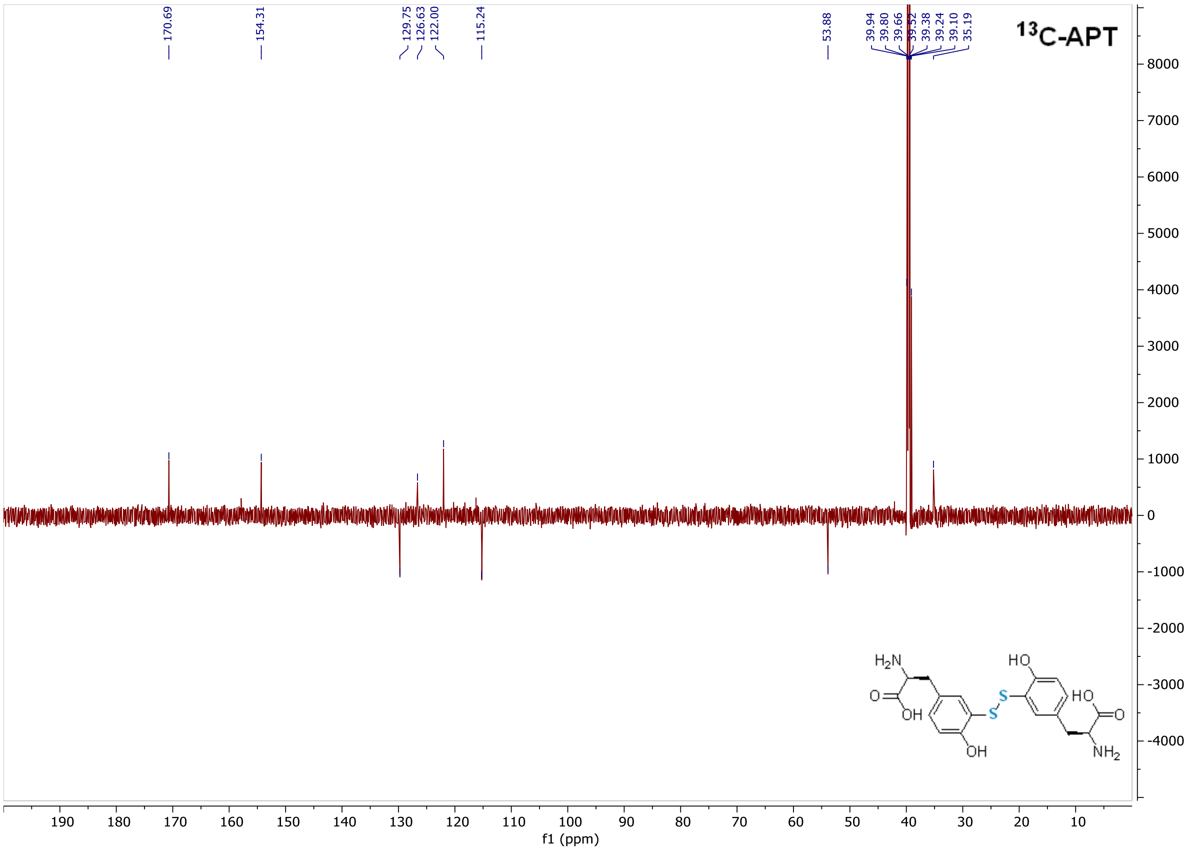

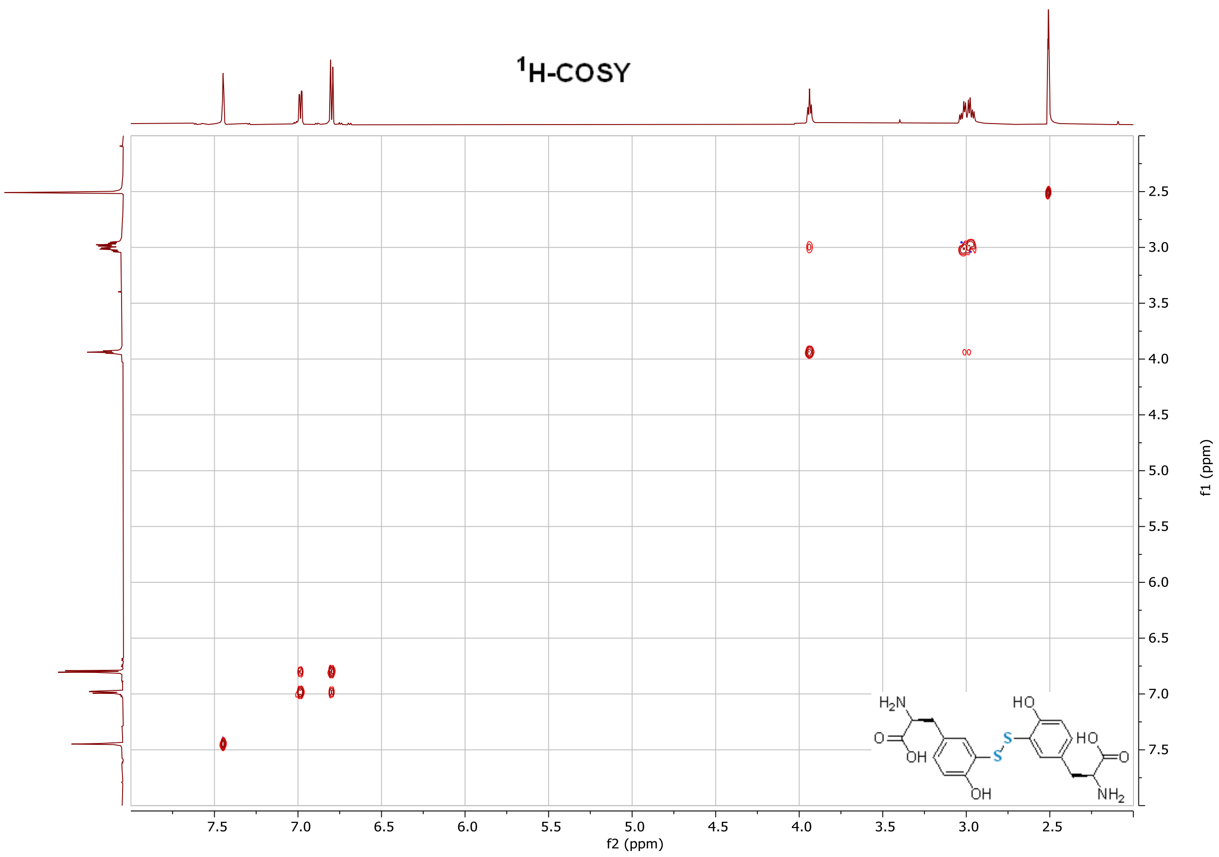

^{1}H-COSY
7.5
7.0
6.5
6.0
5.5
5.0
4.5
4.0
3.5
3.0
2.5
f2 (ppm)
f1 (ppm)
H2N
HO
O
OH
S
S
HO
O
OH
NH2

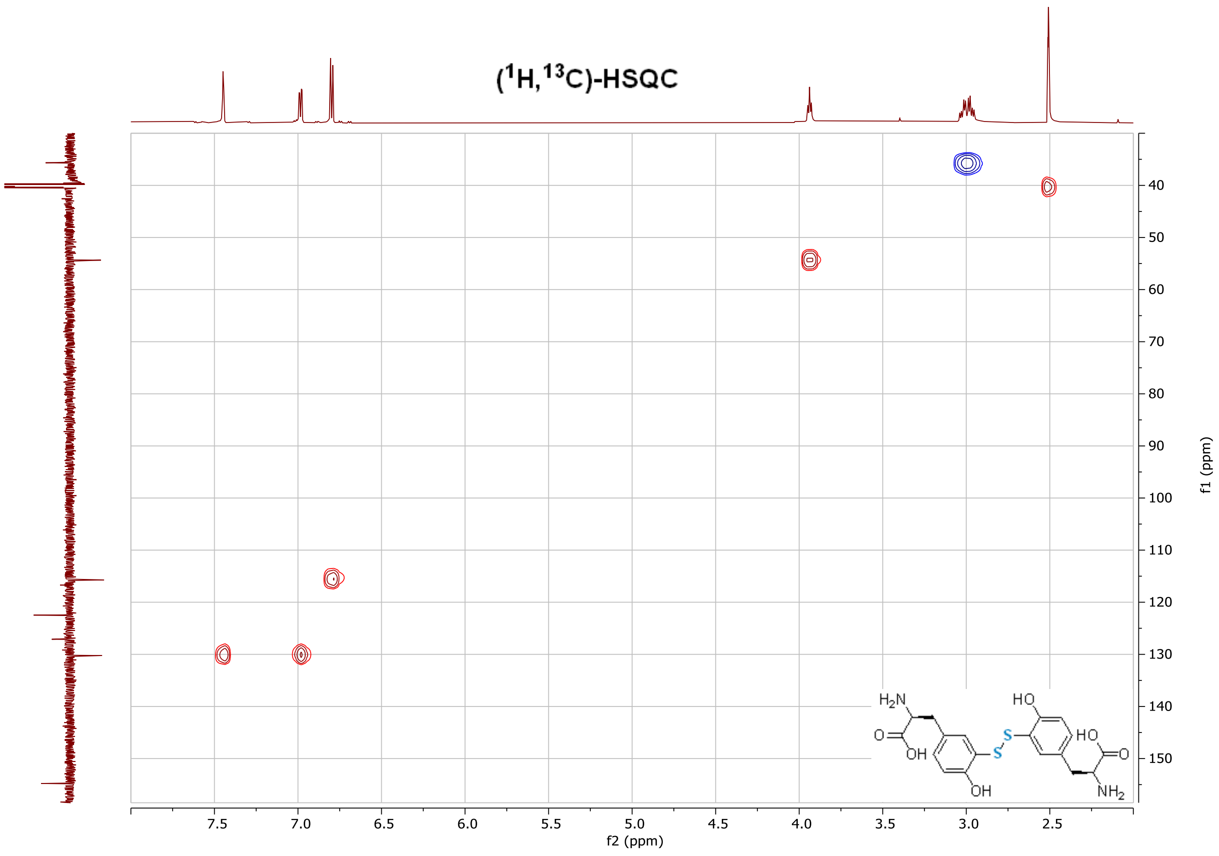

(^{1}H,^{13}C)-HSQC
7.5
7.0
6.5
6.0
5.5
5.0
4.5
4.0
3.5
3.0
2.5
f2 (ppm)
40
50
60
70
80
90
100
110
120
130
140
150
f1 (ppm)
H2N
HO
O
OH
S
S
HO
O
OH
NH2

^{1}H and ^{31}P spectra of ethyl diethynyl phosphinate[1,2] ($CDCL_3$)

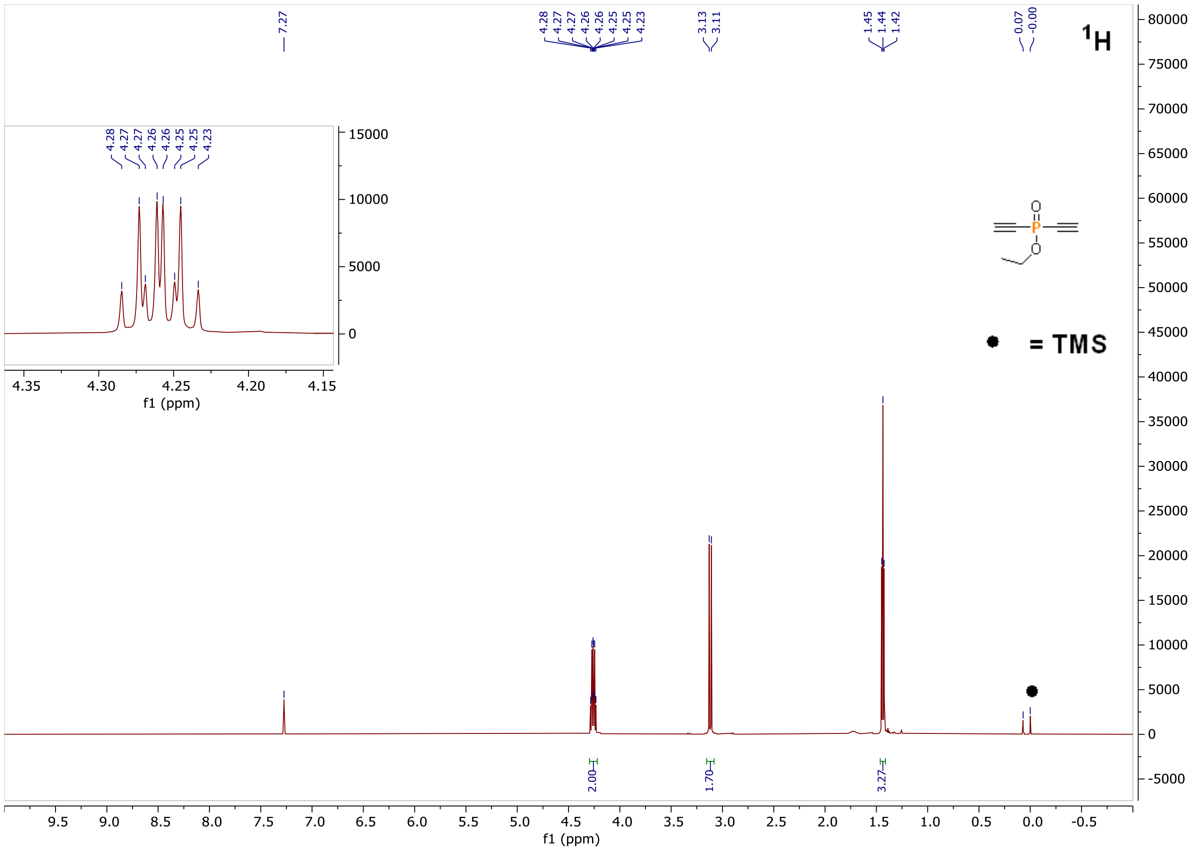

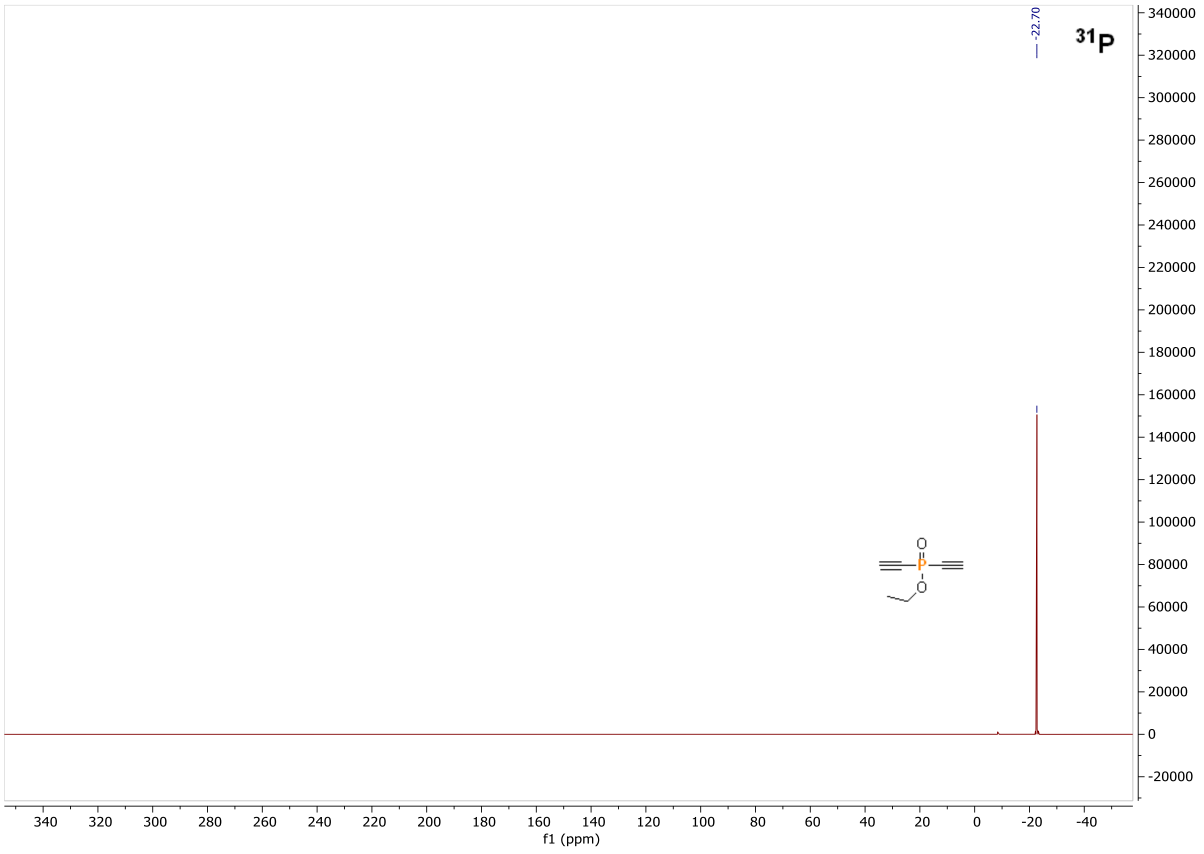

^{1}H, ^{1}H (^{31}P-decoupled), ^{1}H-COSY, ^{31}P, (^{1}H,^{31}P)-HMBC, ^{13}C, (^{1}H,^{13}C)-HSQC, (^{1}H,^{13}C)-HMQC and (^{1}H,^{13}C)-HMBC spectra of 10-Z/E (DMSO-d_6)

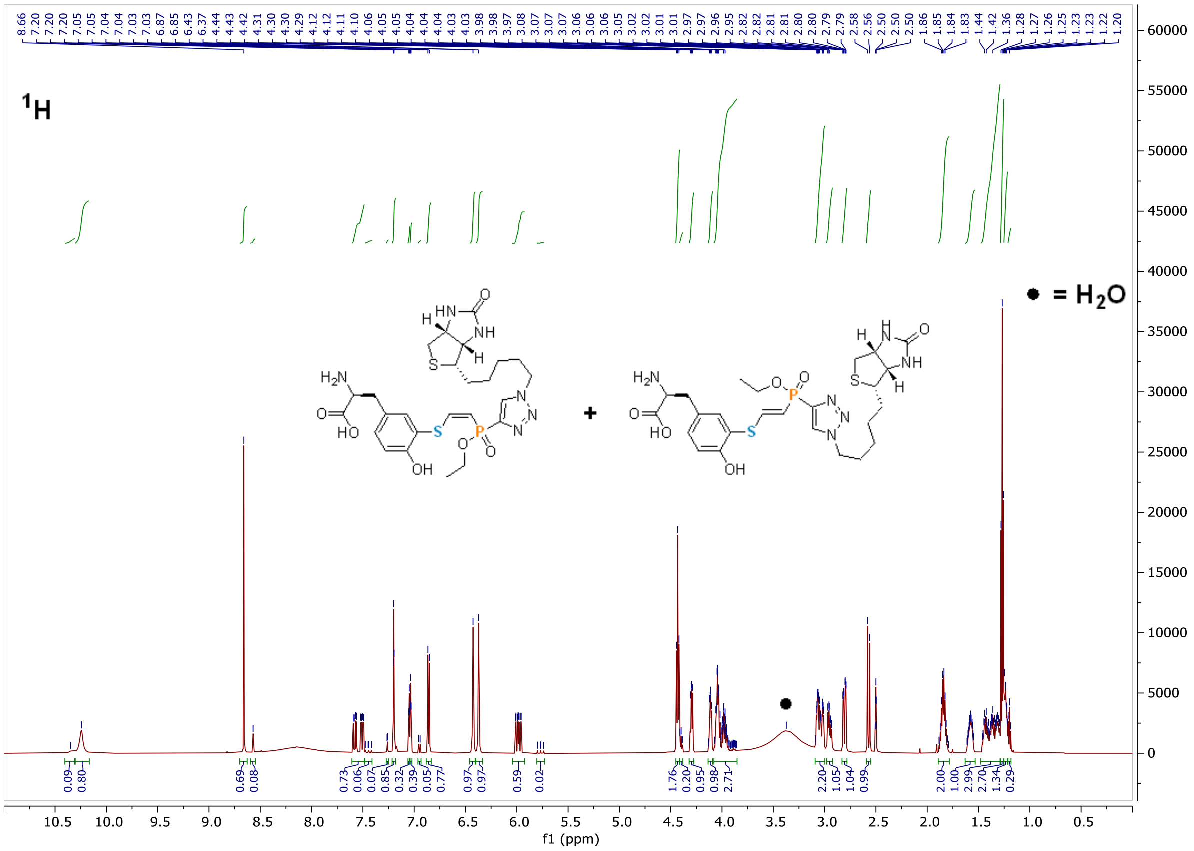

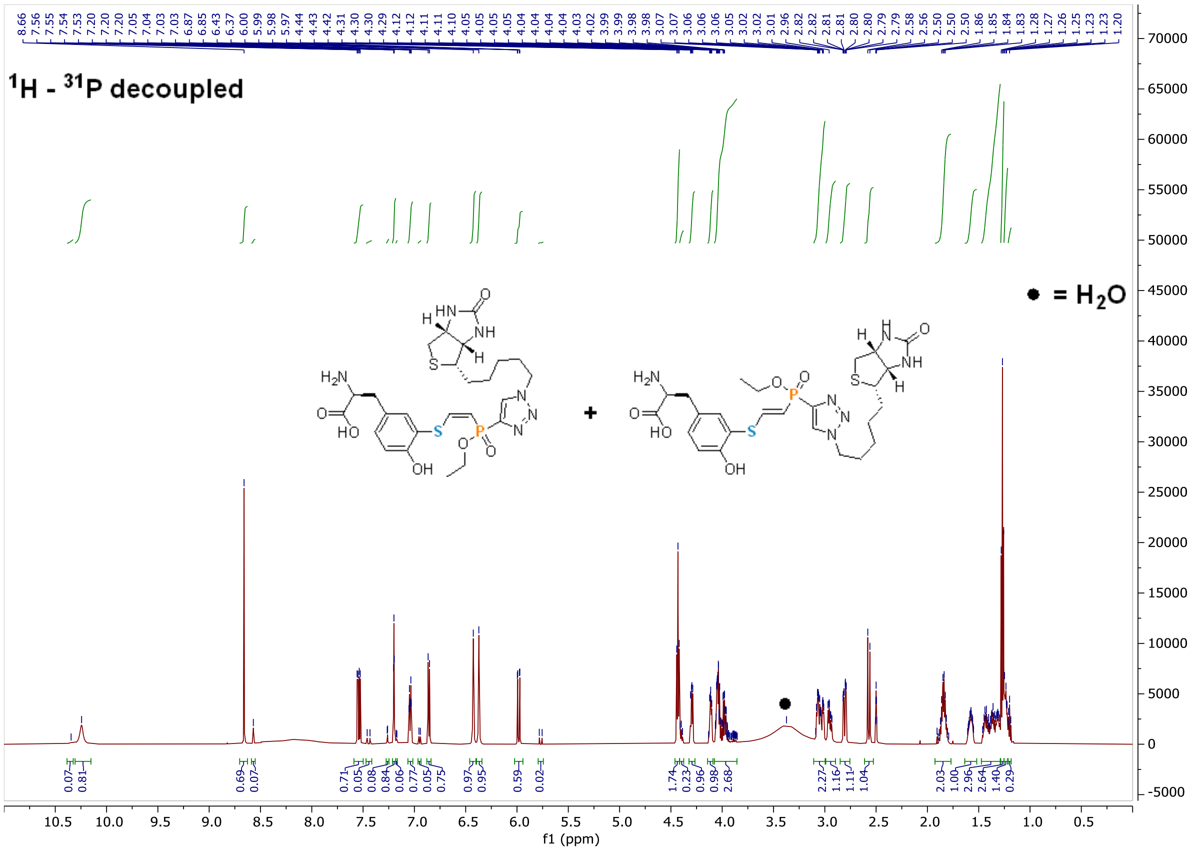

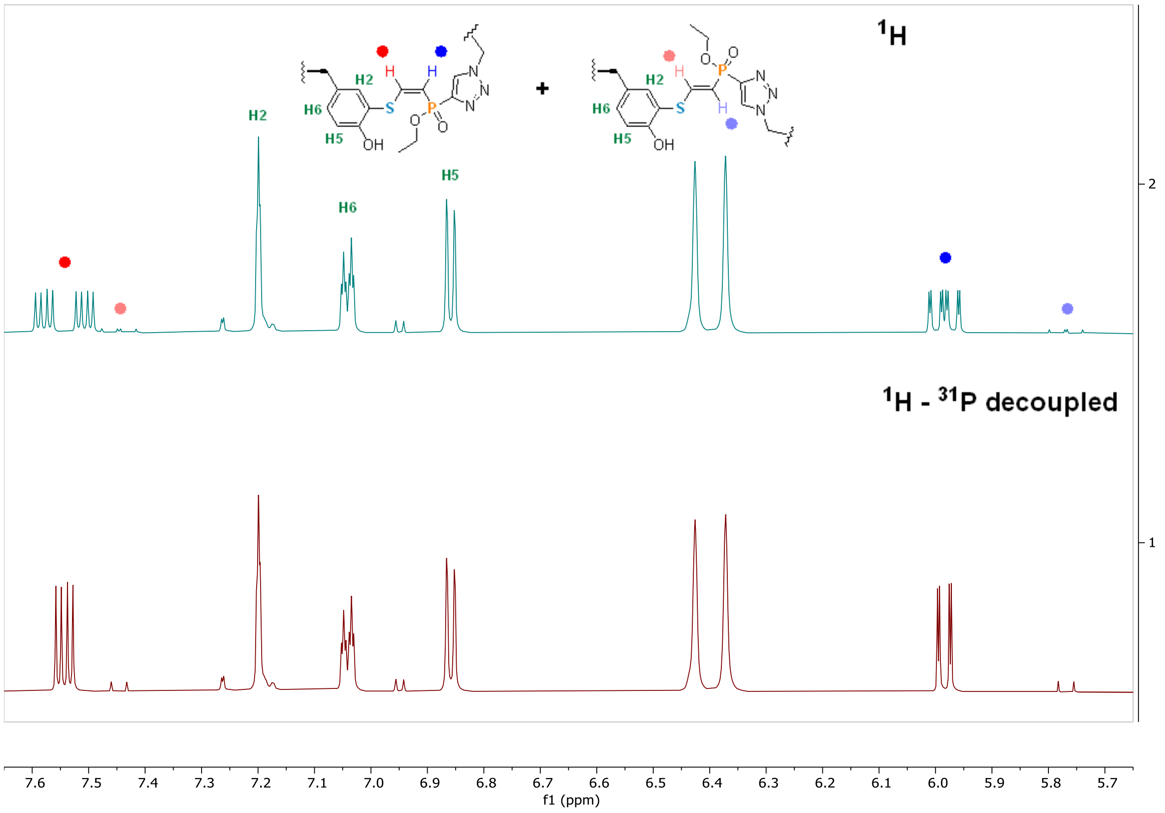

1H
1H - 31P decoupled
H2
H6
H5
S
P
O
OH
N
f1 (ppm)

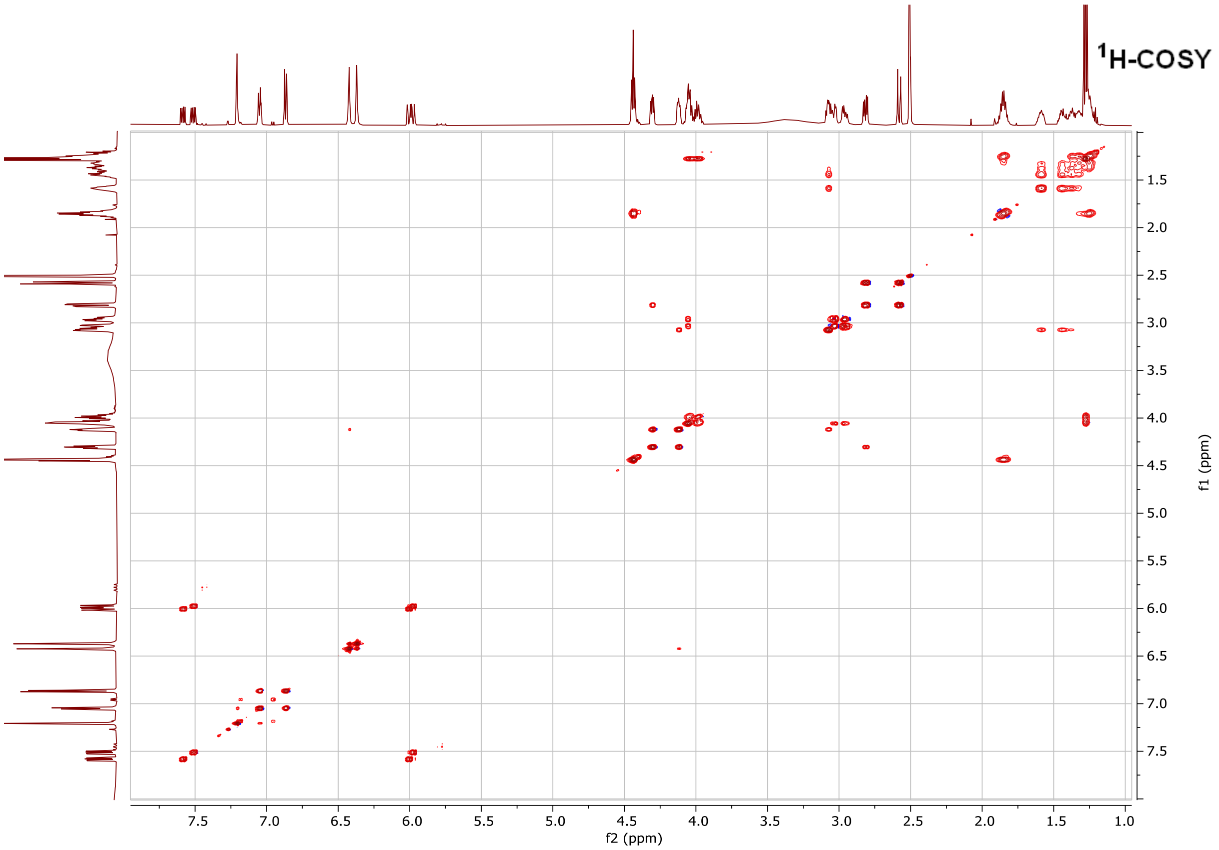

1H-COSY
f2 (ppm)
f1 (ppm)

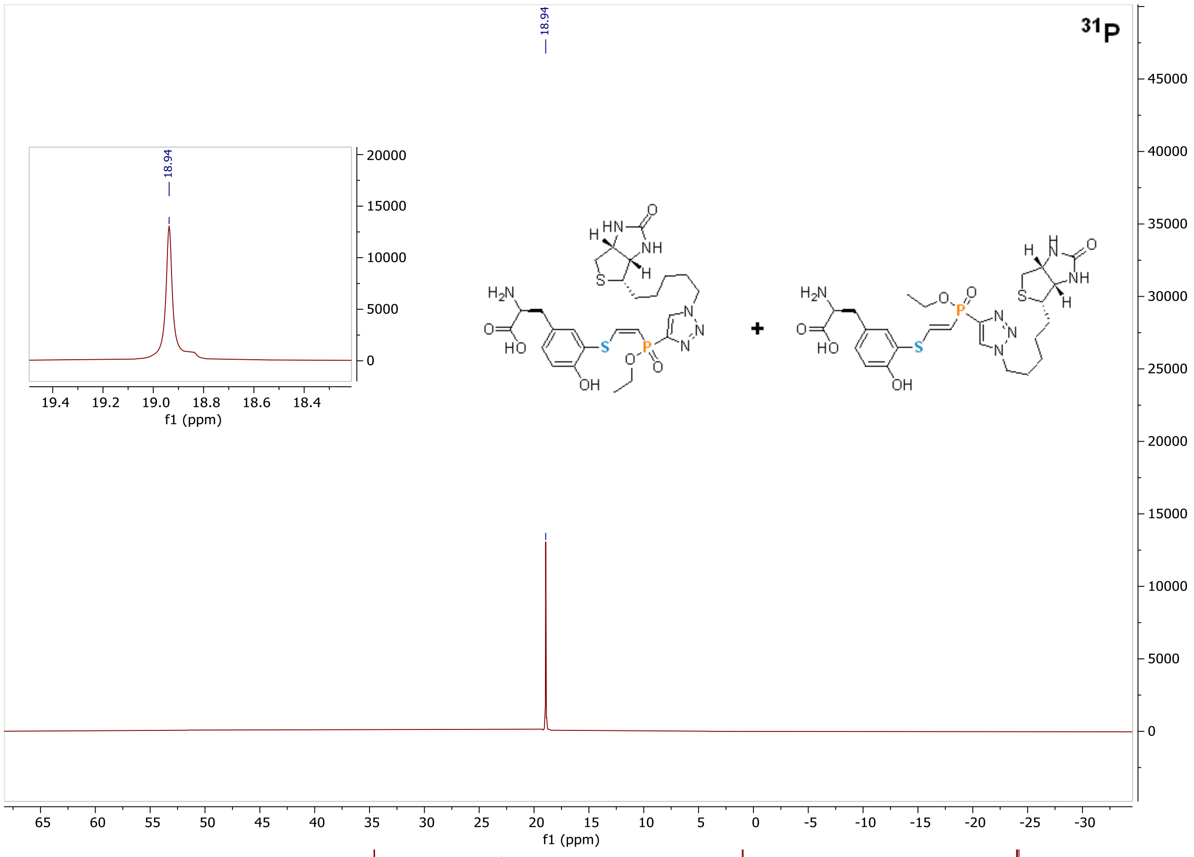
31P
18.94
f1 (ppm)

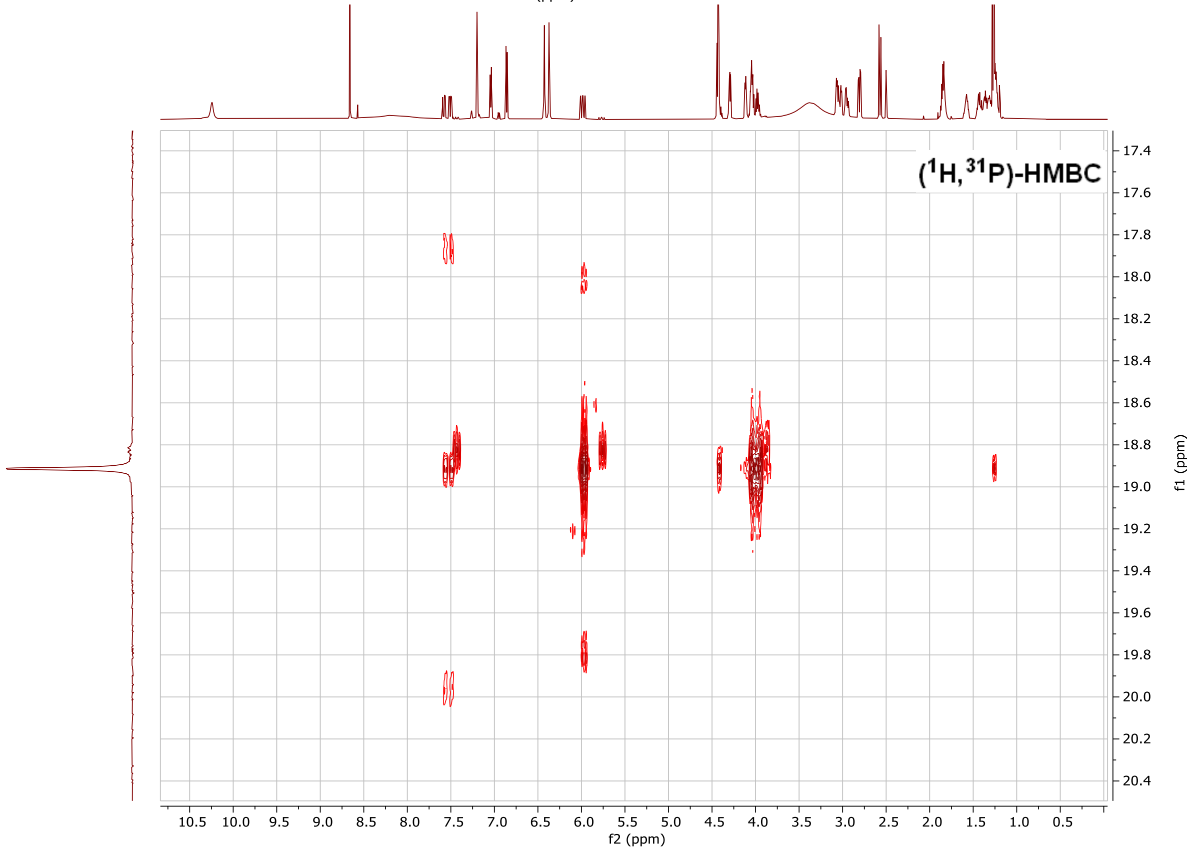
(1H,31P)-HMBC
f2 (ppm)
f1 (ppm)

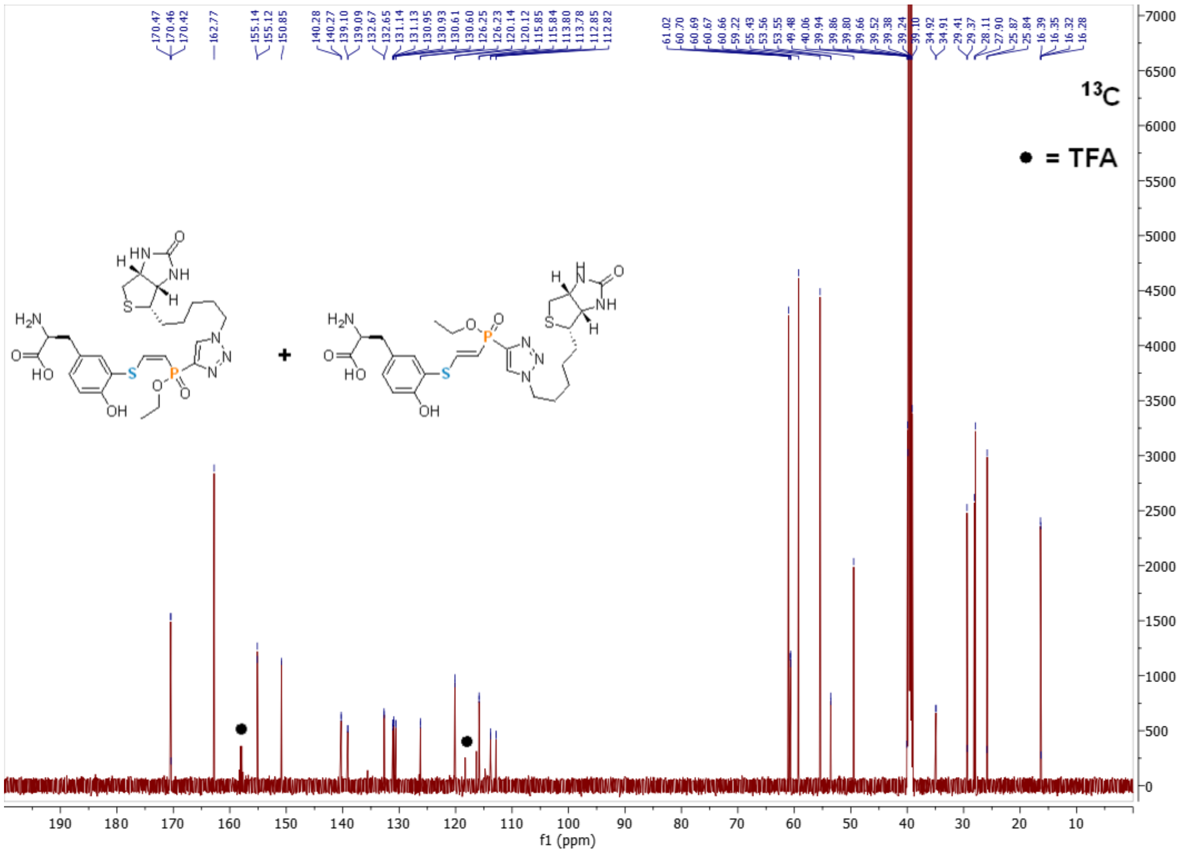

13C
● = TFA
f1 (ppm)

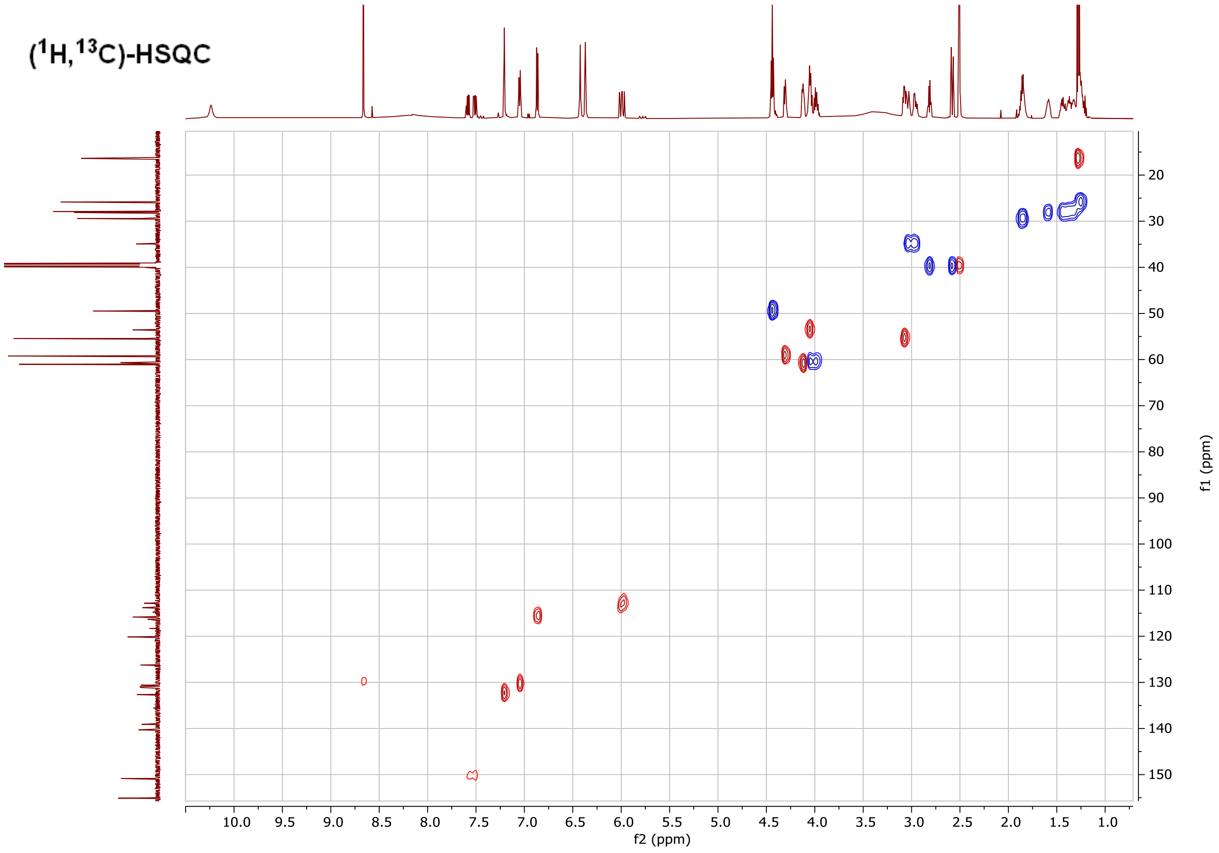

(1H,13C)-HSQC
f2 (ppm)
f1 (ppm)

(^1H,^{13}C)-HMQC

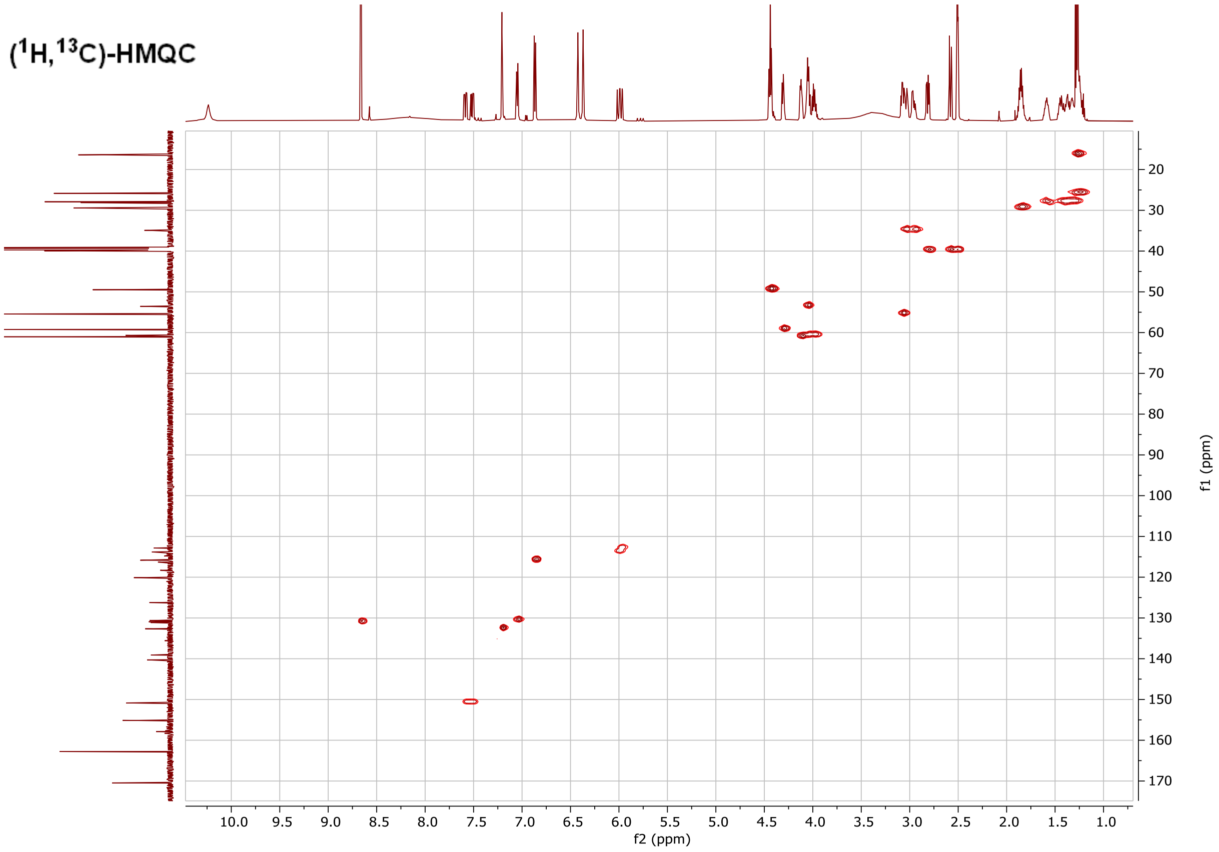

(^1H,^{13}C)-HMBC

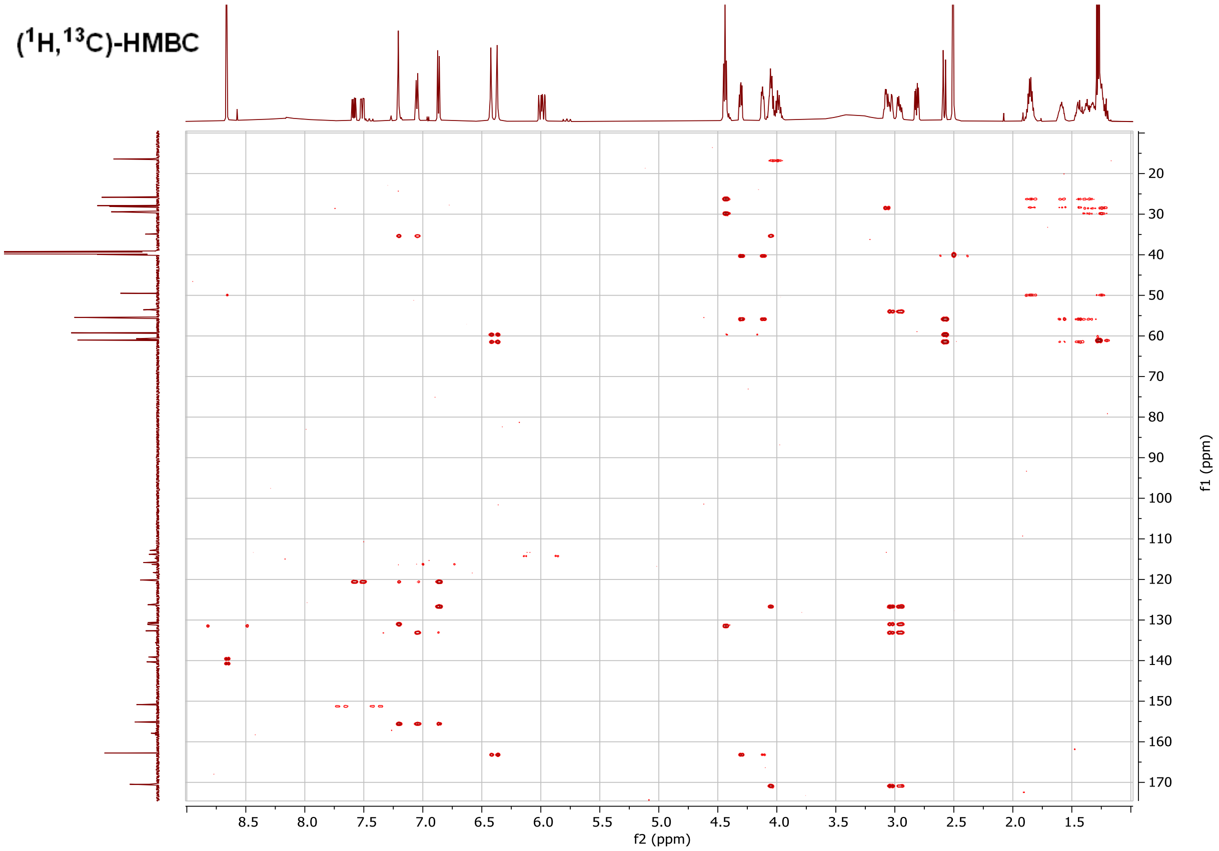

^{1}H, ^{31}P, (^{1}H,^{31}P)-HMBC spectra of 10-Z (D_2O)

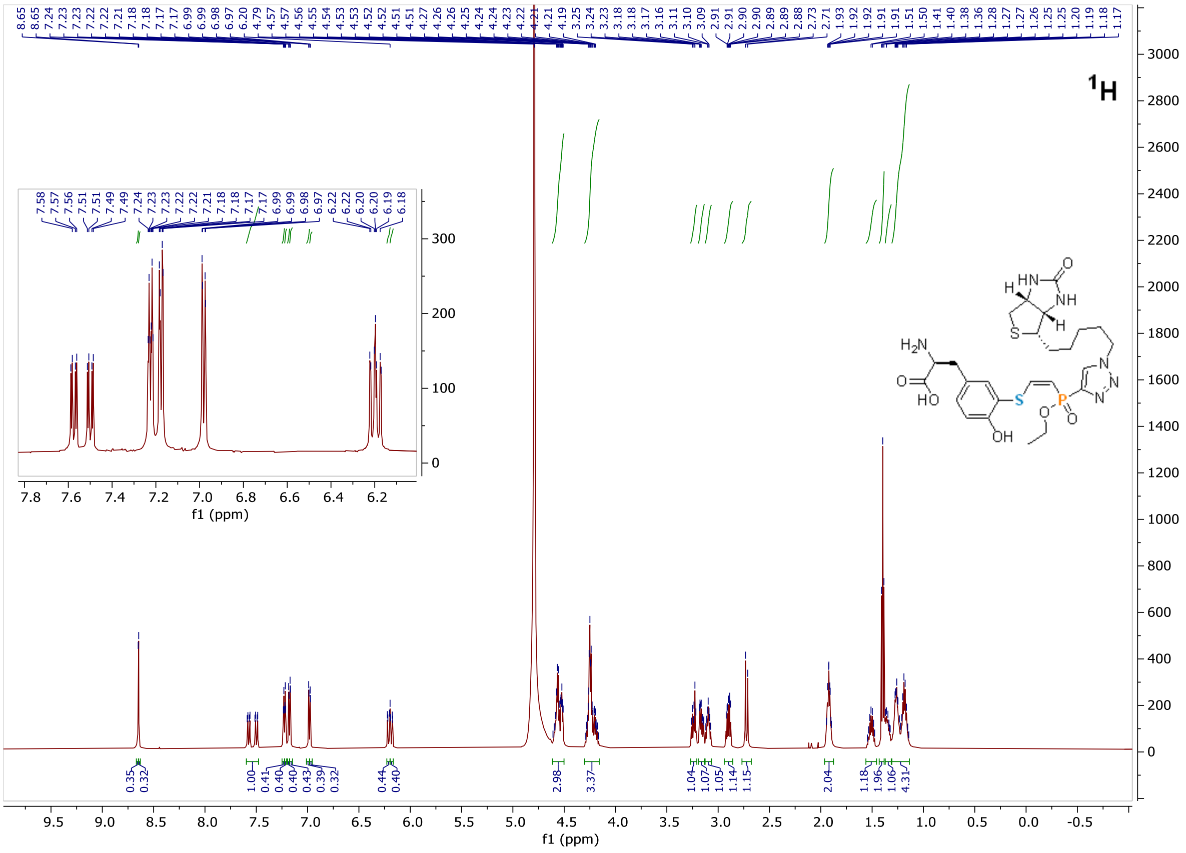

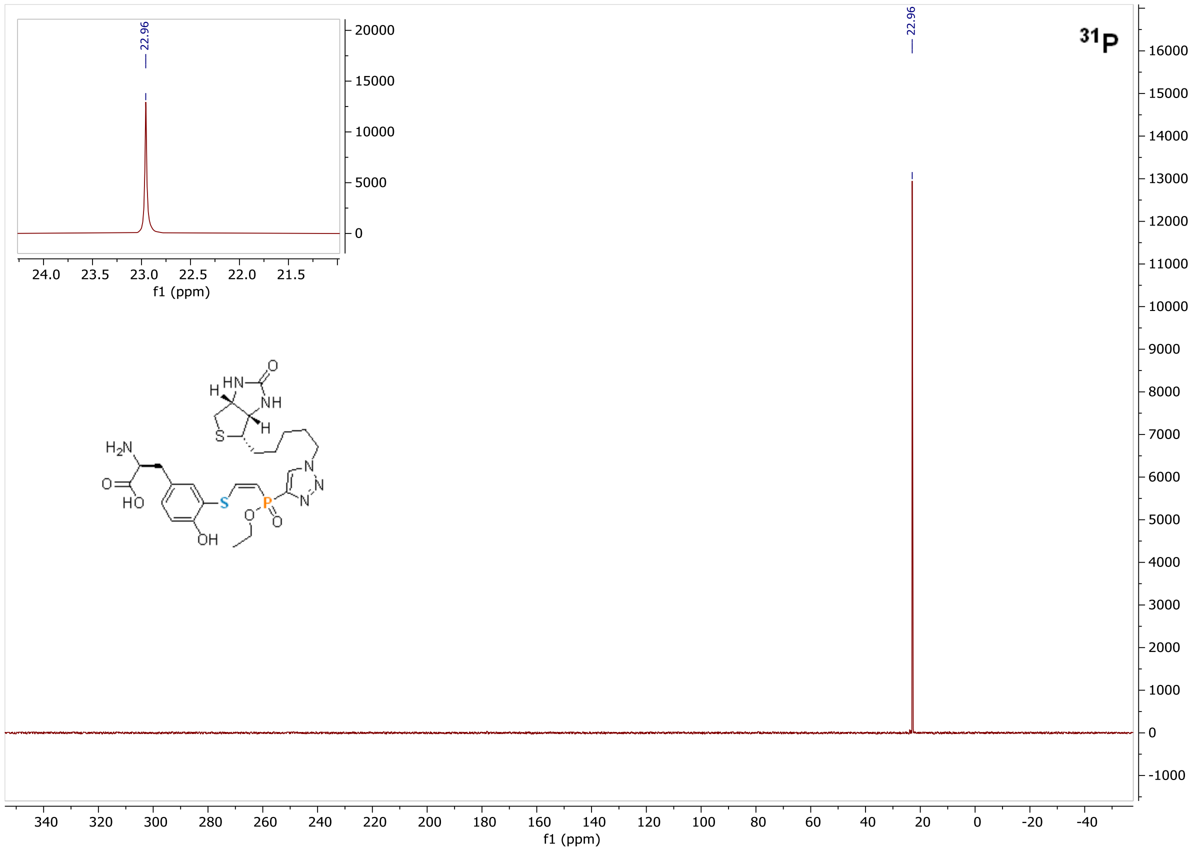

(^{1}H,^{31}P)-HMBC

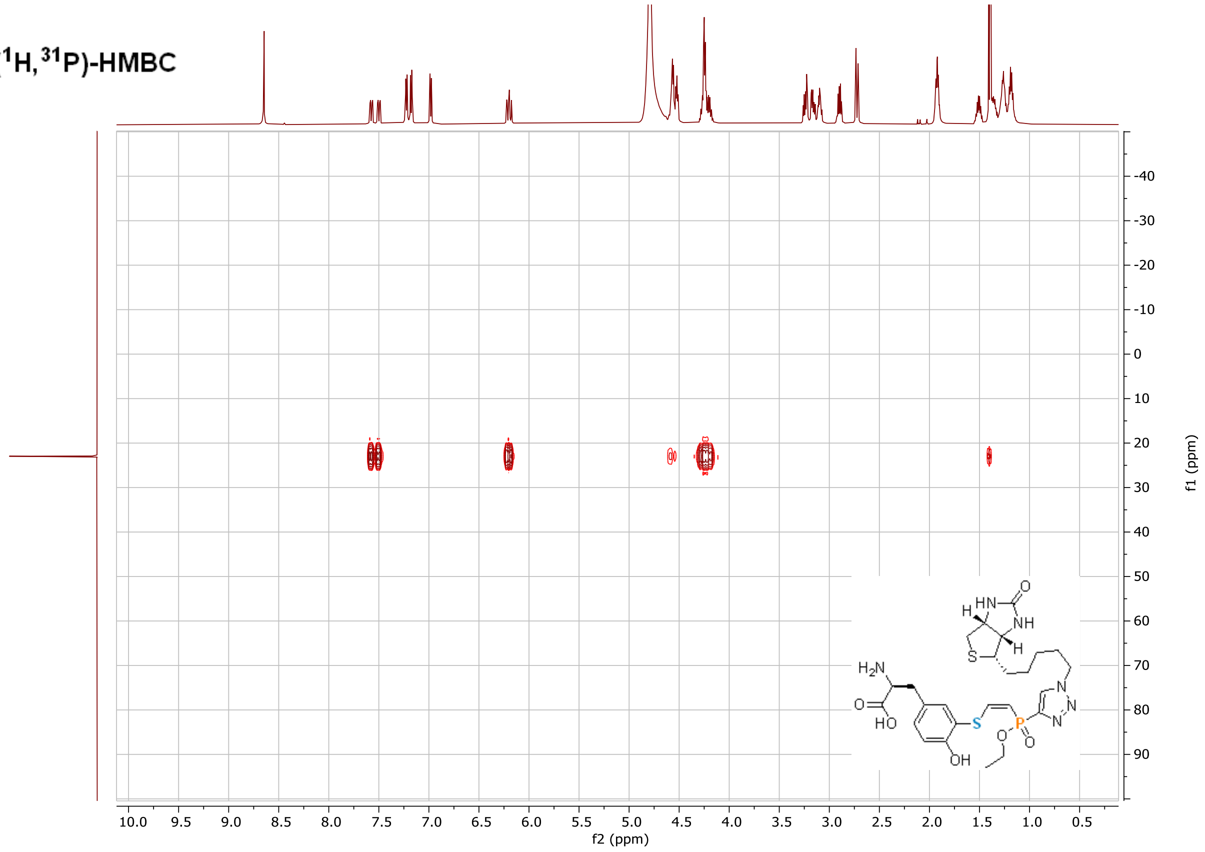

^1H, ^1H (^{31}P-decoupled), ^1H-COSY, ^{31}P, (^1H,^{31}P)-HMBC spectra of 11-Z (DMSO-d$_6$)

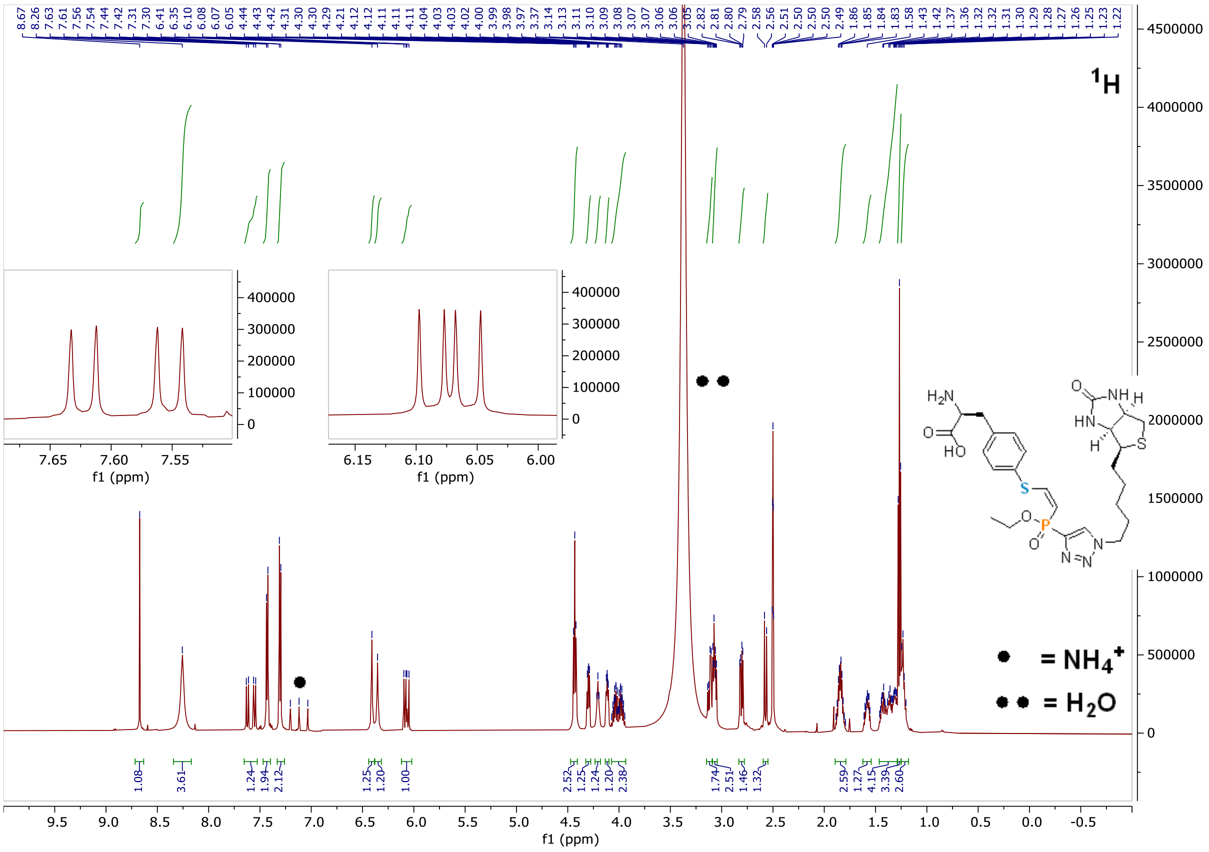

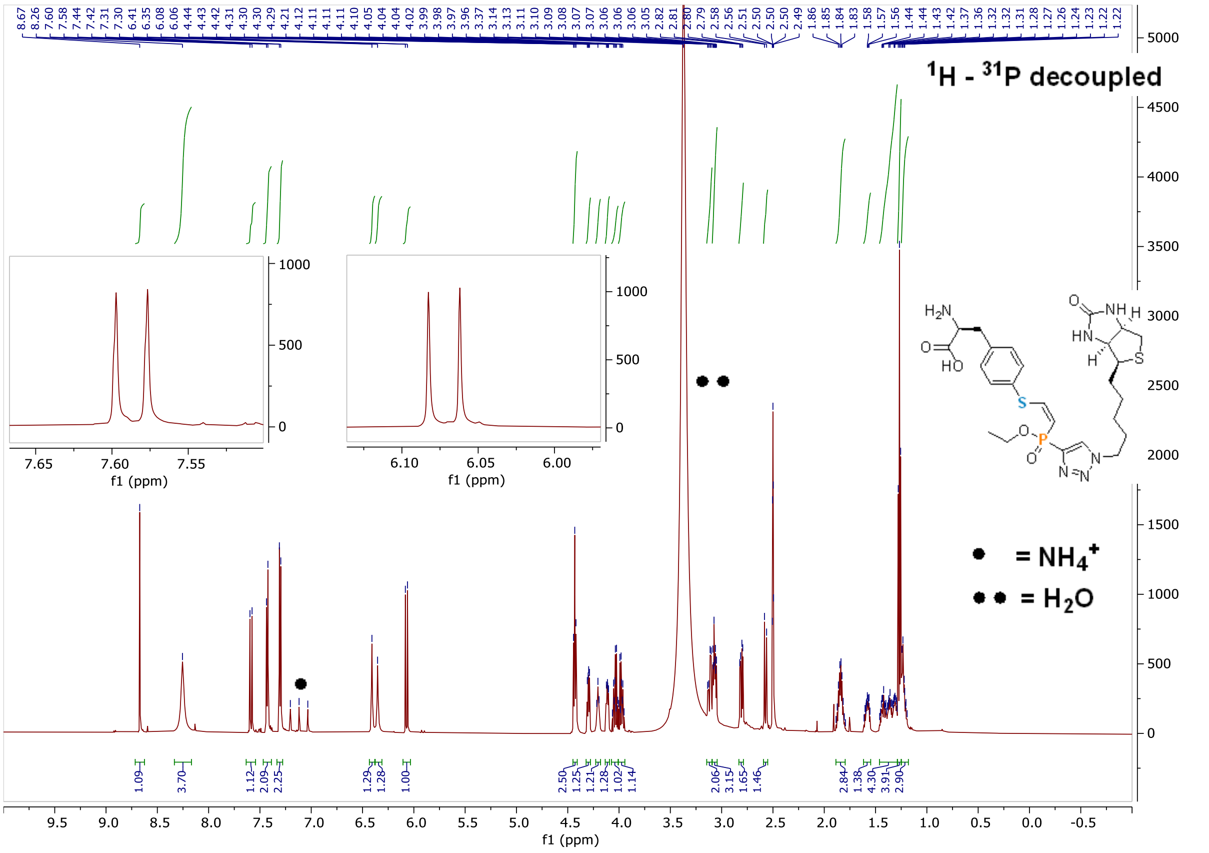

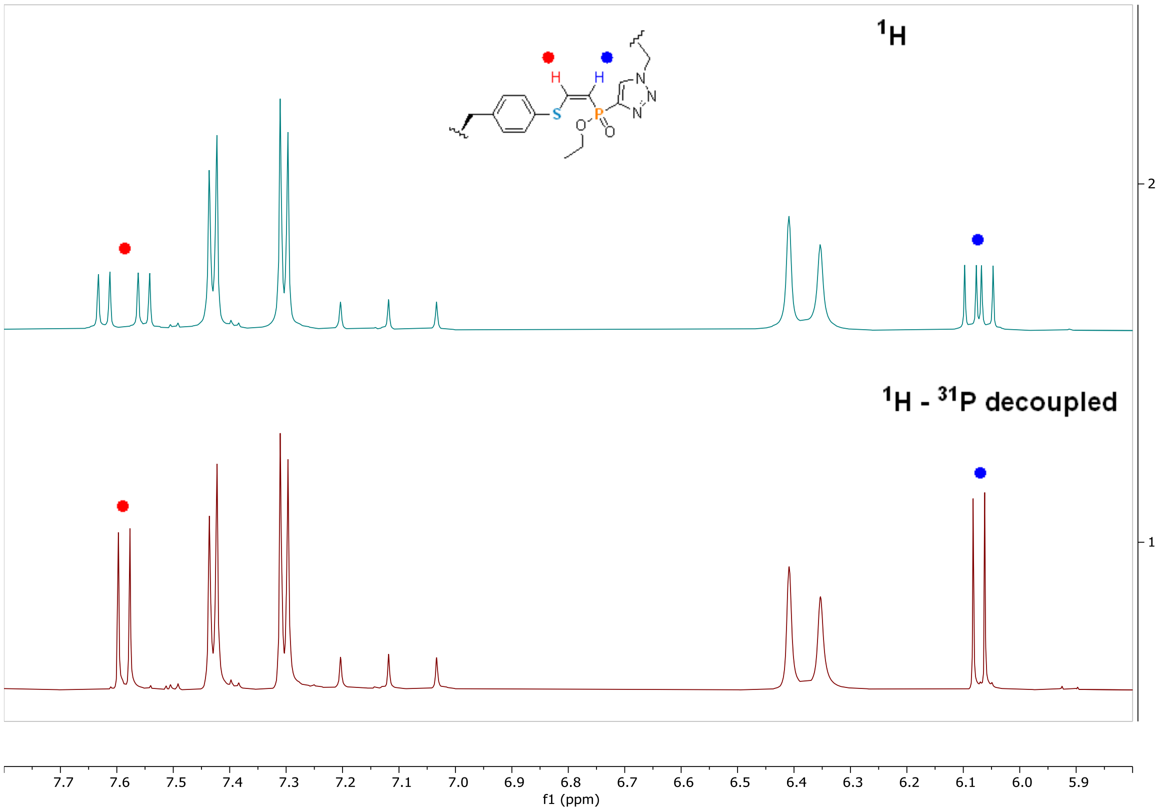
1H
1H - 31P decoupled
f1 (ppm)

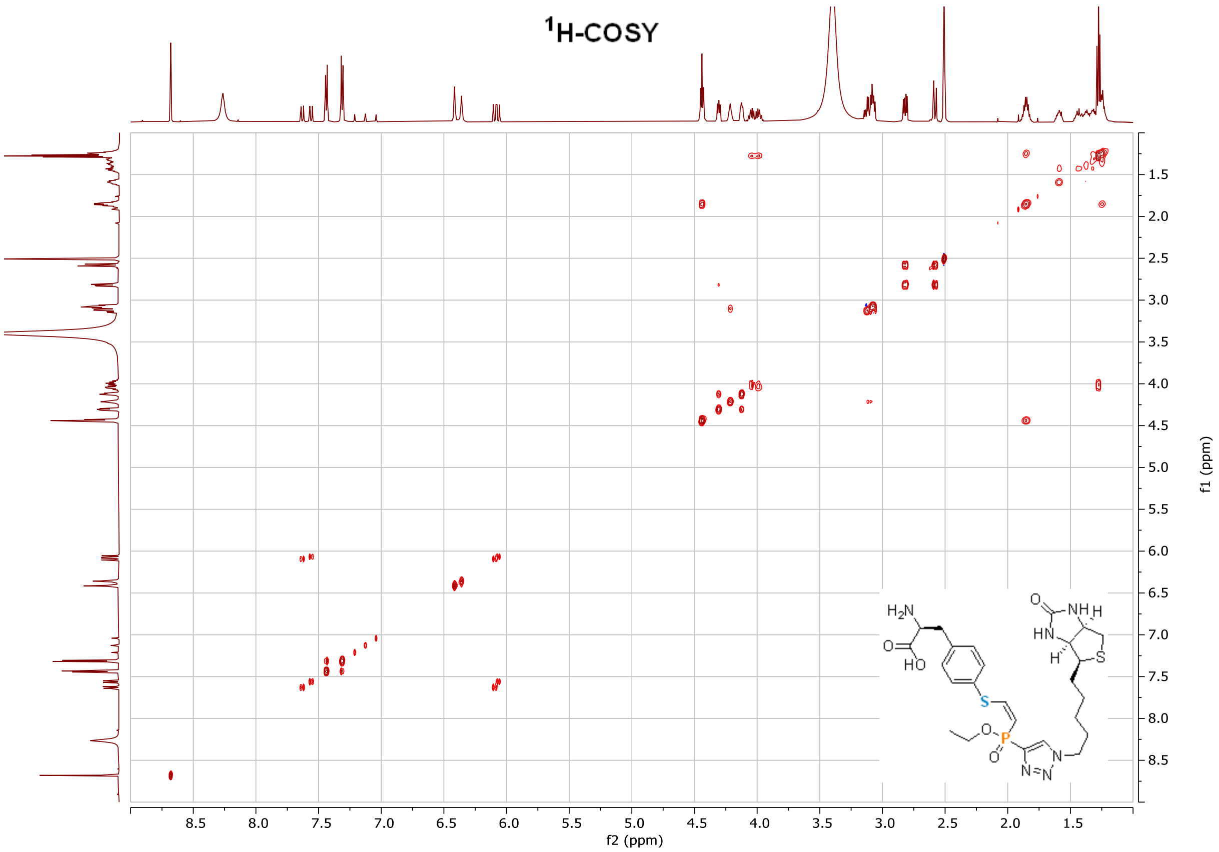
1H-COSY
f2 (ppm)
f1 (ppm)

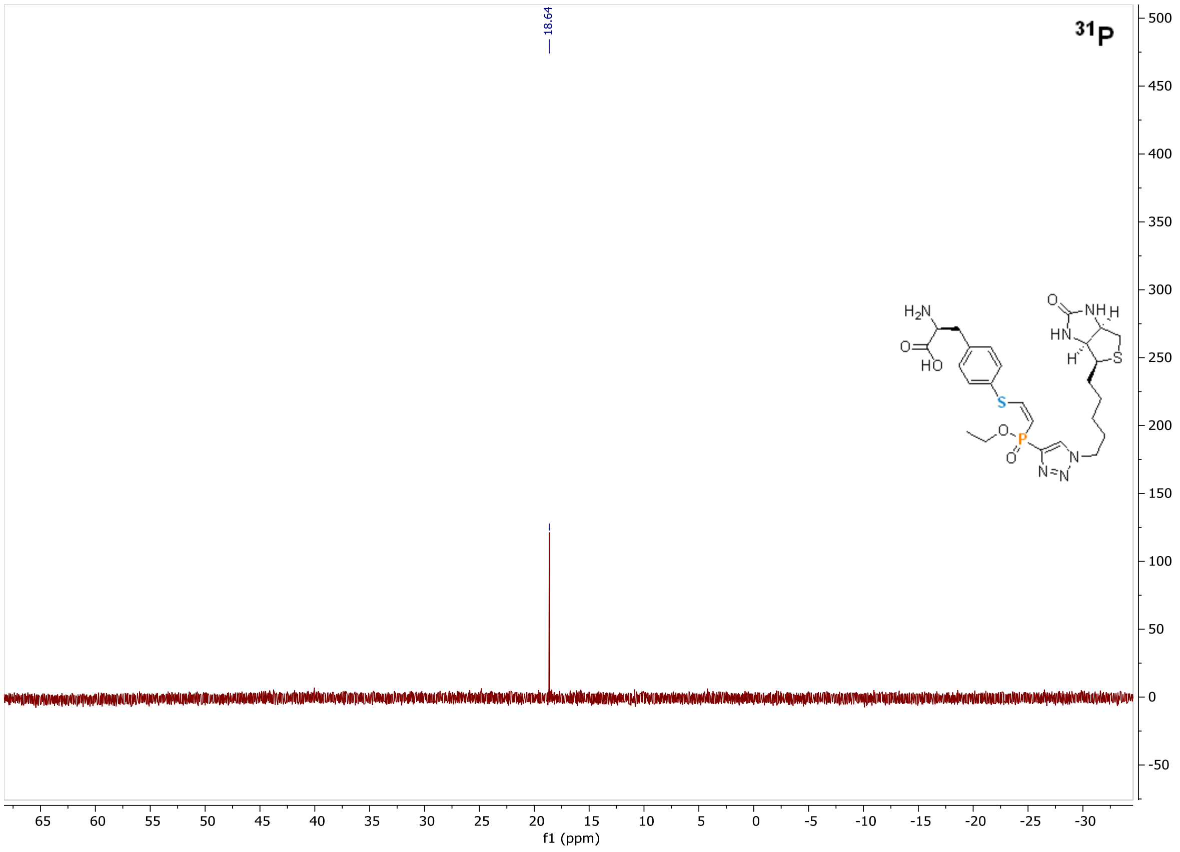

18.64
^{31}P
f1 (ppm)

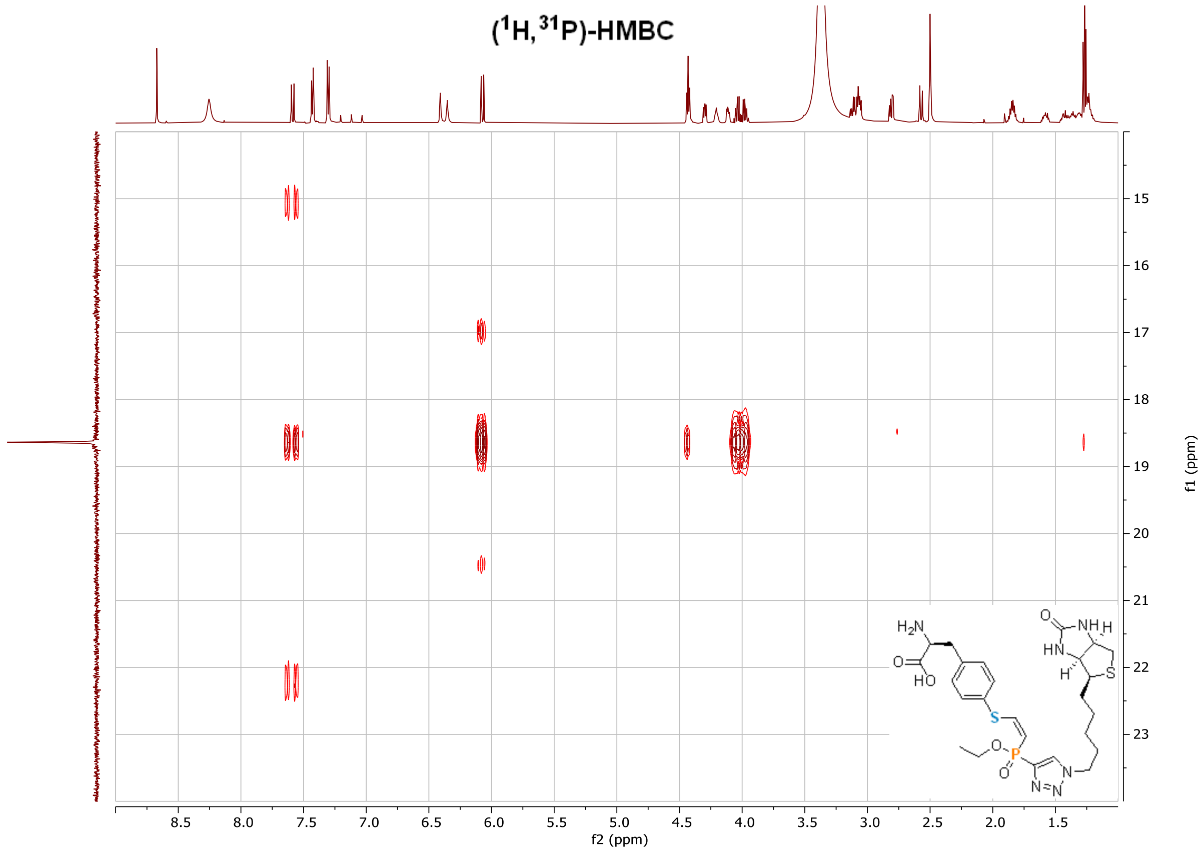

(^{1}H,^{31}P)-HMBC
f2 (ppm)
f1 (ppm)

^{1}H, ^{13}C-APT, ^{1}H-COSY and (^{1}H,^{13}C)-HSQC spectra of 17 (DMSO-d$_6$)

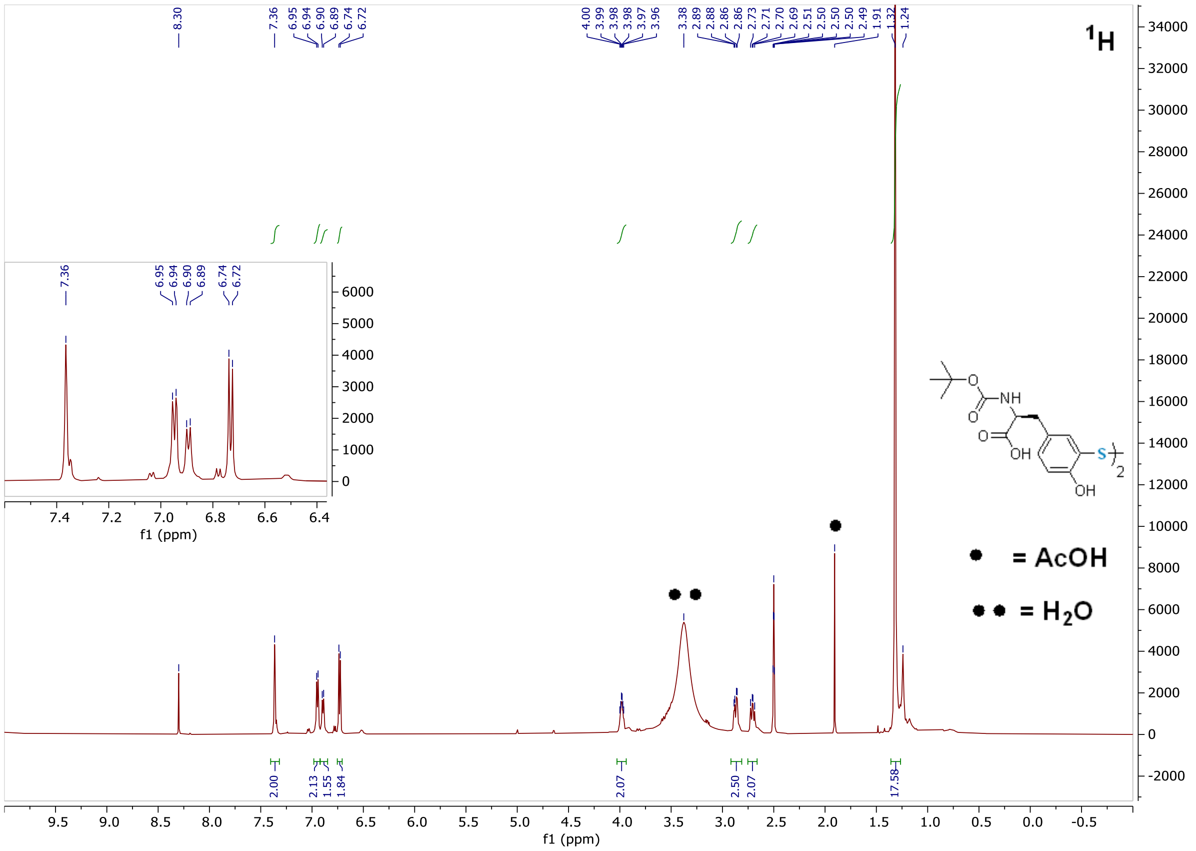

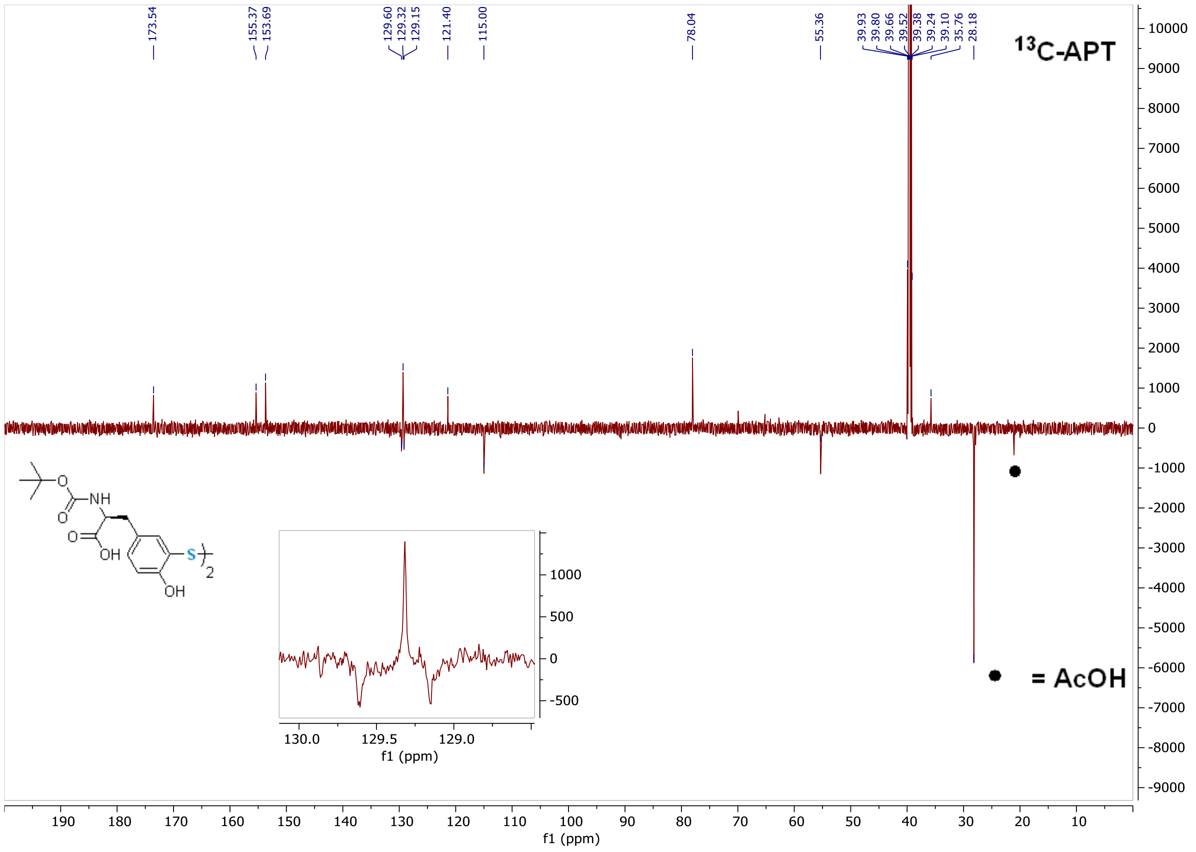

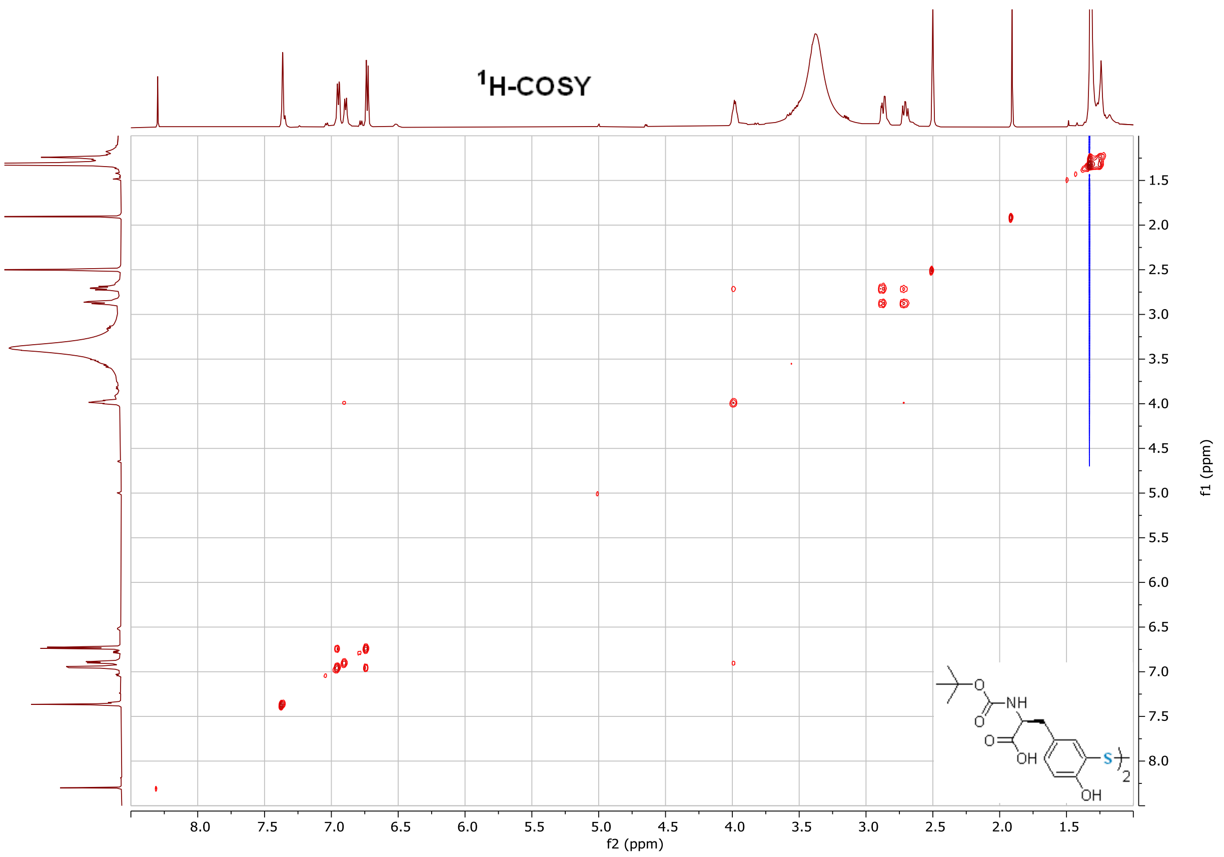

¹H-COSY
f2 (ppm)
f1 (ppm)
NH
O
OH
S
2

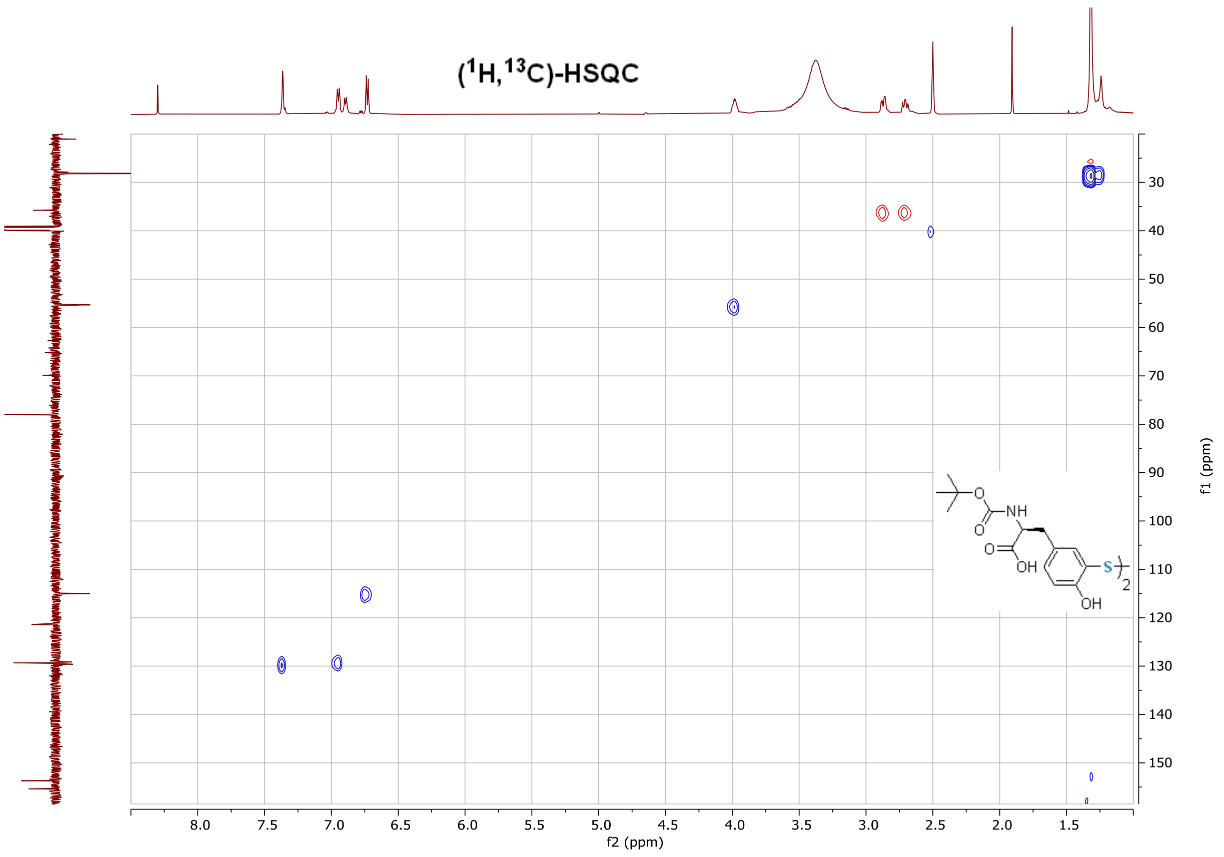

(¹H,¹³C)-HSQC
f2 (ppm)
f1 (ppm)
NH
O
OH
S
2

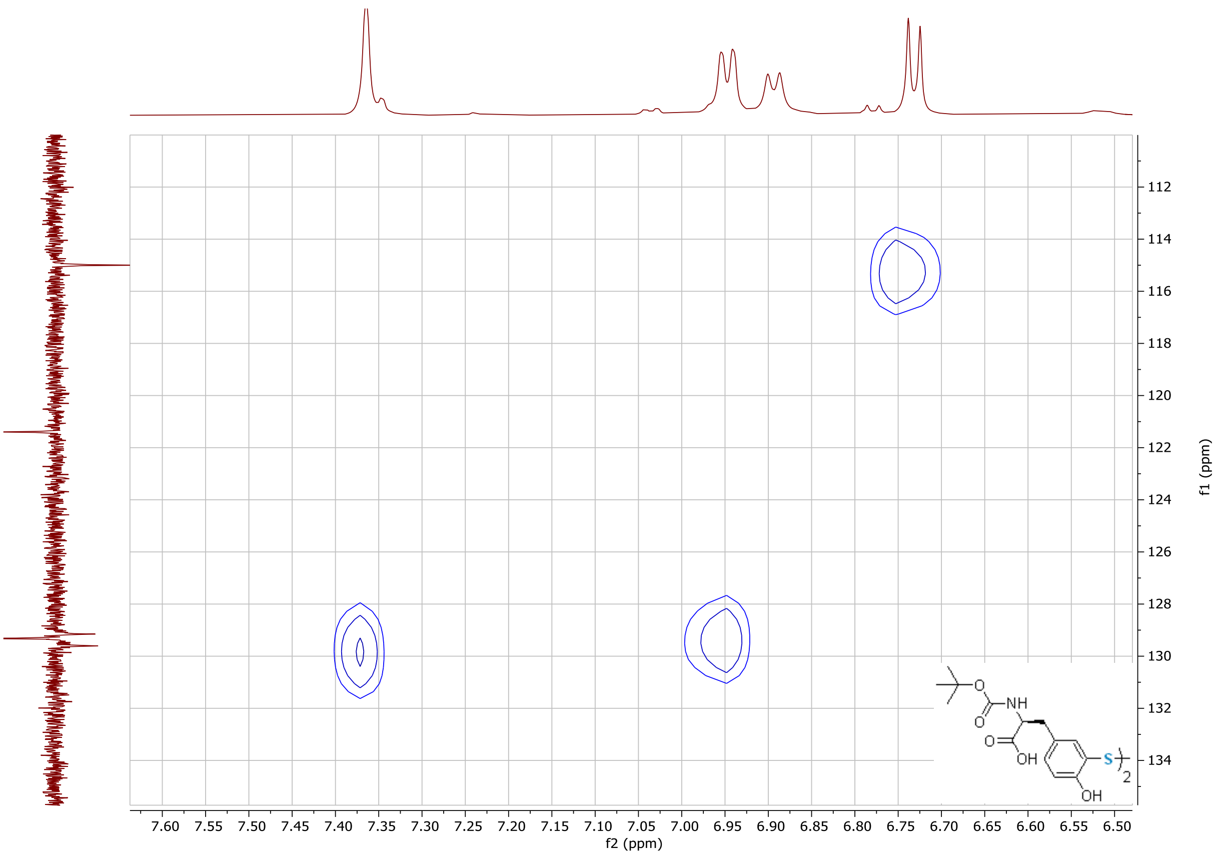

f2 (ppm)
f1 (ppm)
NH
O
OH
S
2
OH

^{1}H, ^{13}C, ^{1}H-COSY and (^{1}H,^{13}C)-HSQC spectra of 13 (DMSO-d_6)

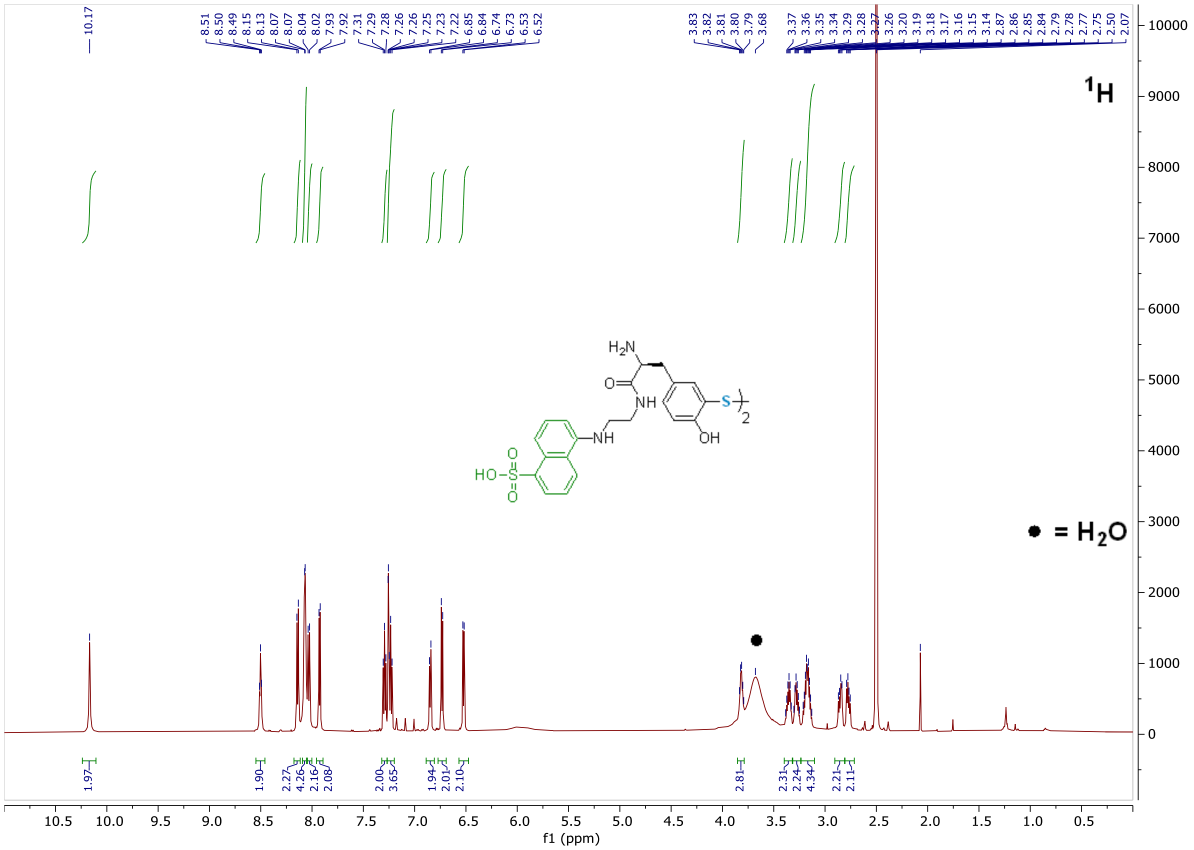

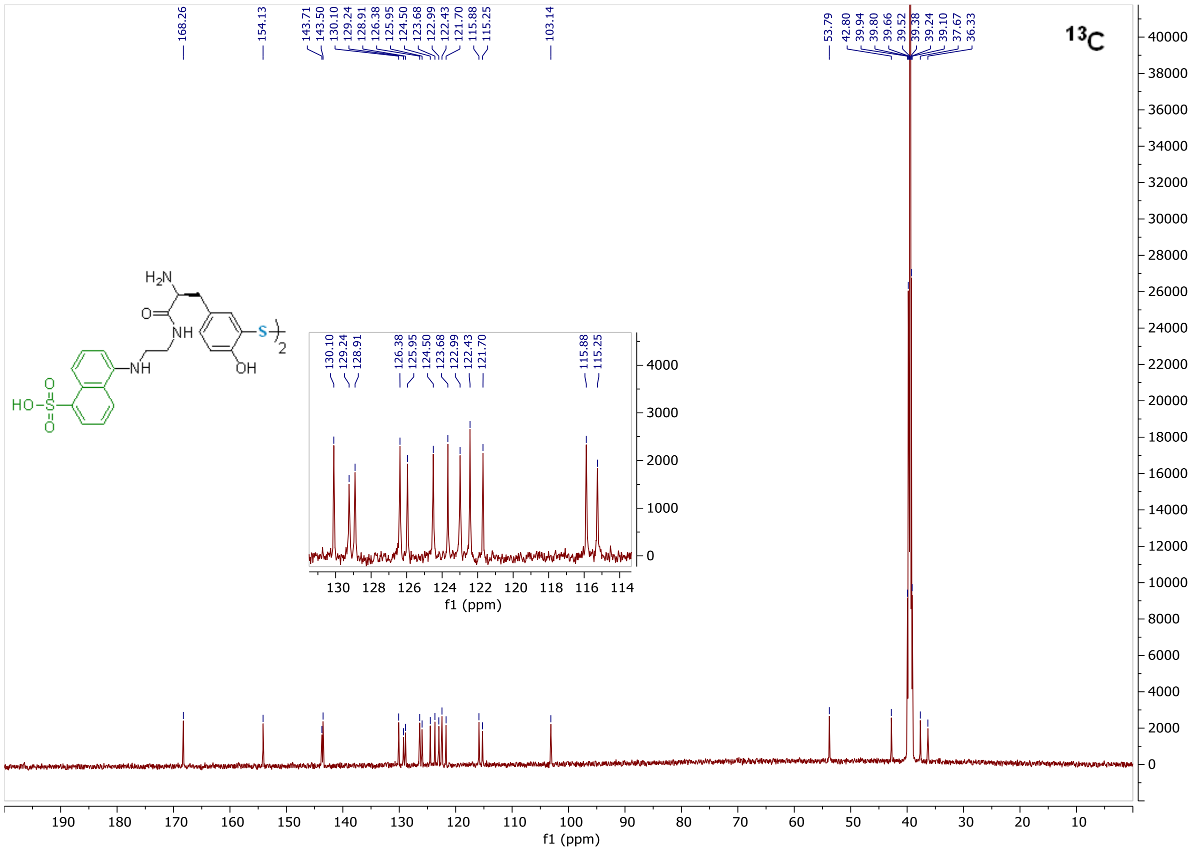

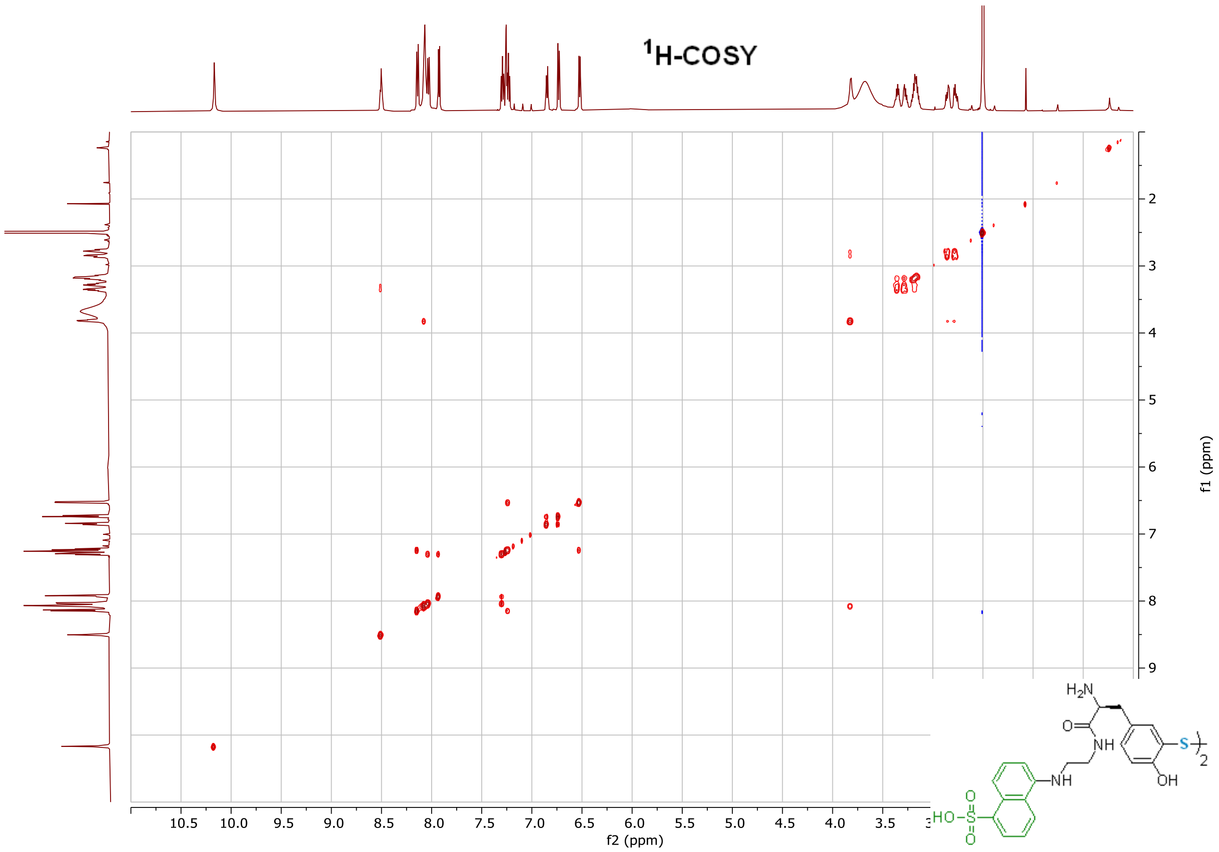
1H-COSY
f1 (ppm)
f2 (ppm)
H2N
O
NH
NH
S
2
OH
HO-S
O
O

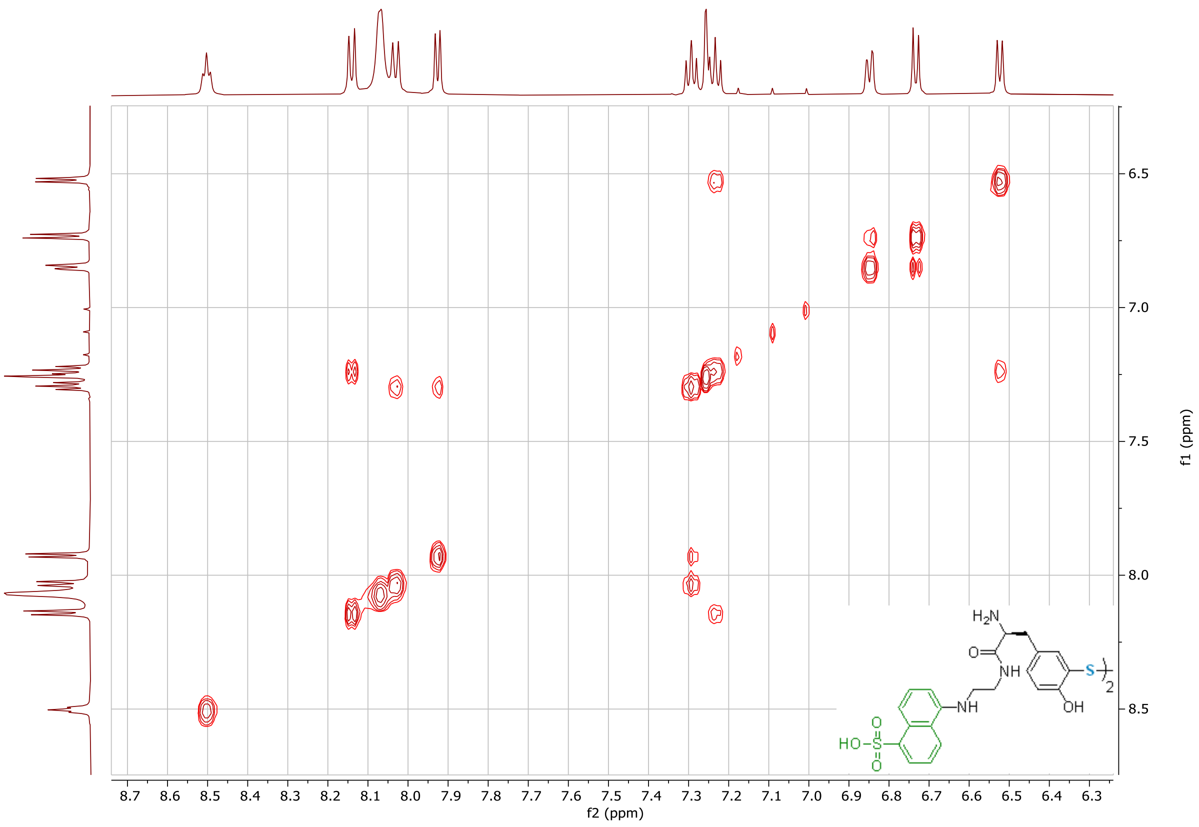
f1 (ppm)
f2 (ppm)
H2N
O
NH
NH
S
2
OH
HO-S
O
O

(^{1}H,^{13}C)-HSQC

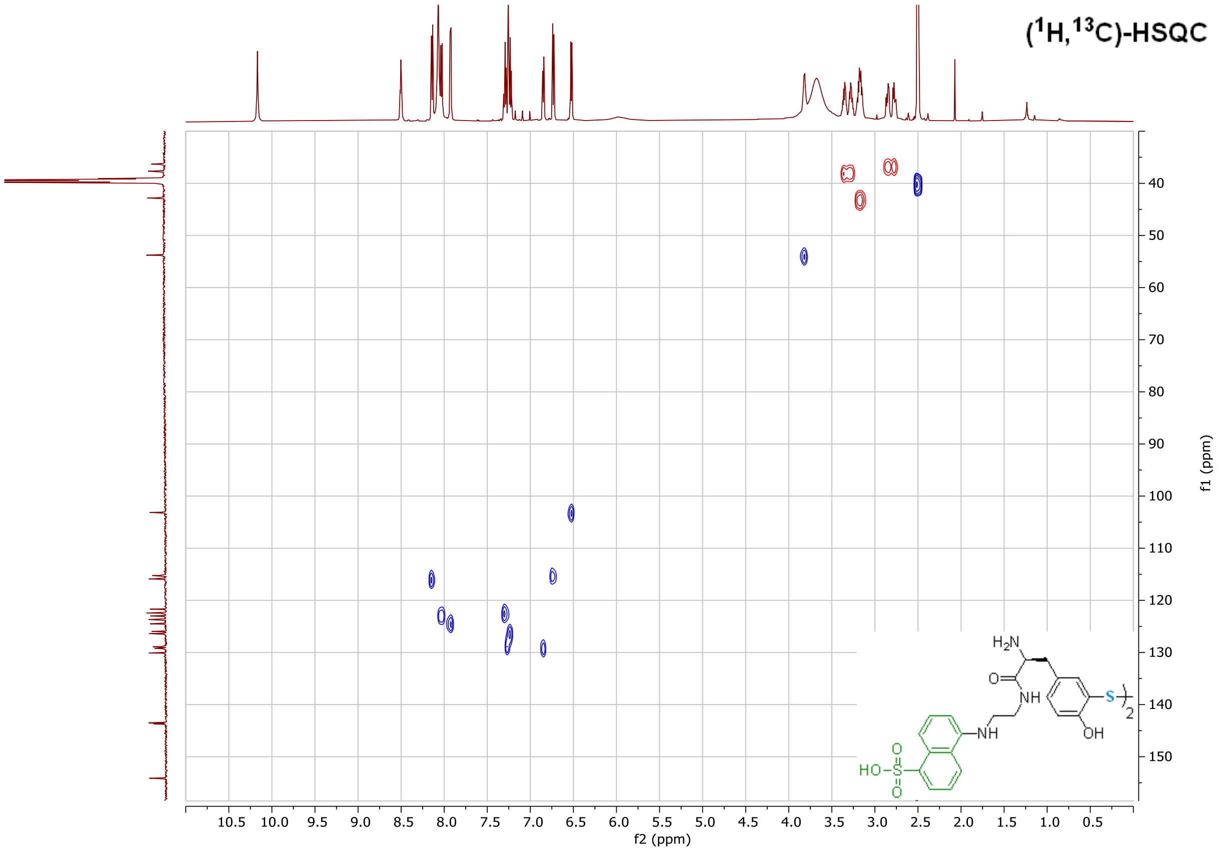

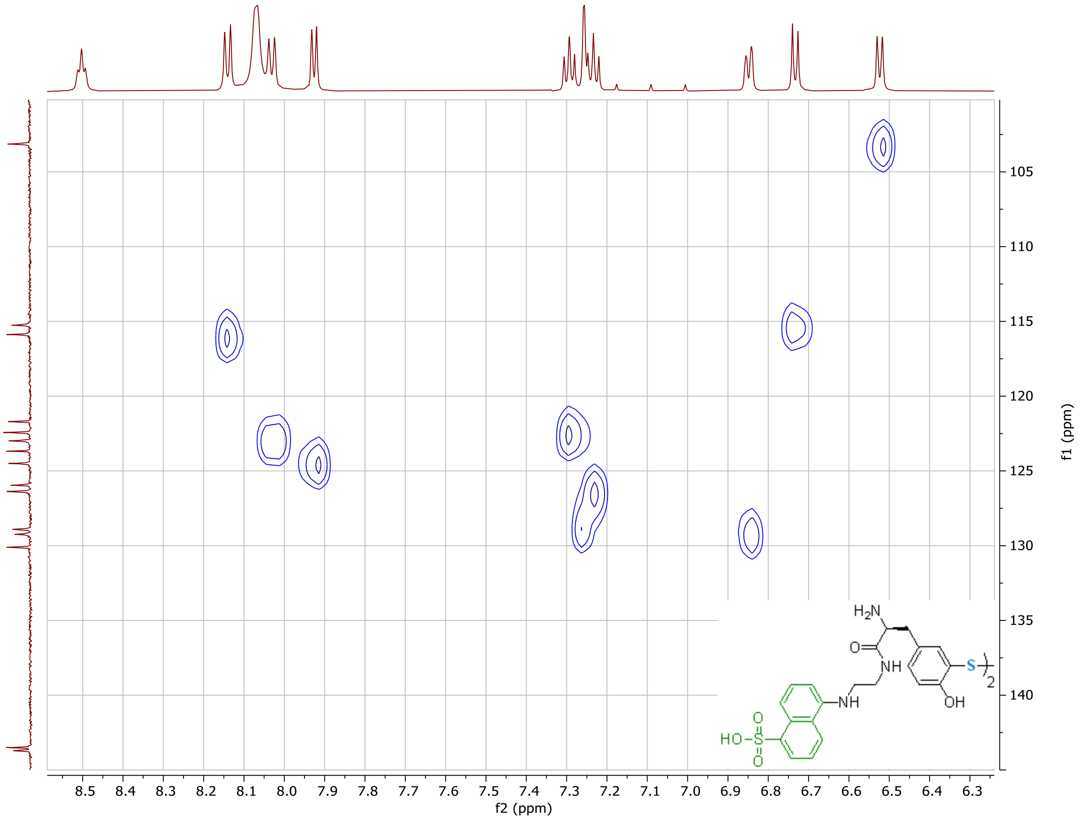

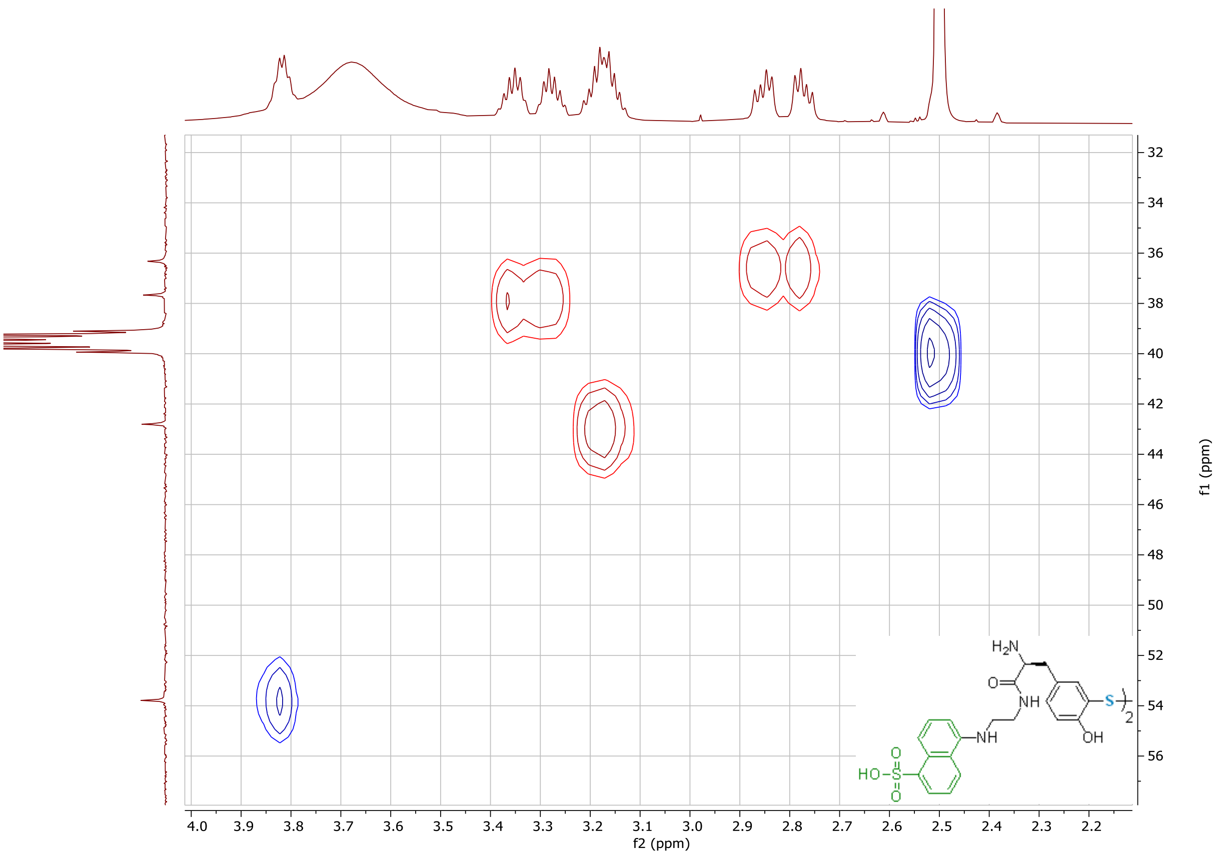
f2 (ppm)
f1 (ppm)
H_2N
O
NH
NH
S
2
OH
HO
S
O
O

^{1}H and ^{13}C spectra of 19 (DMSO-d$_6$).

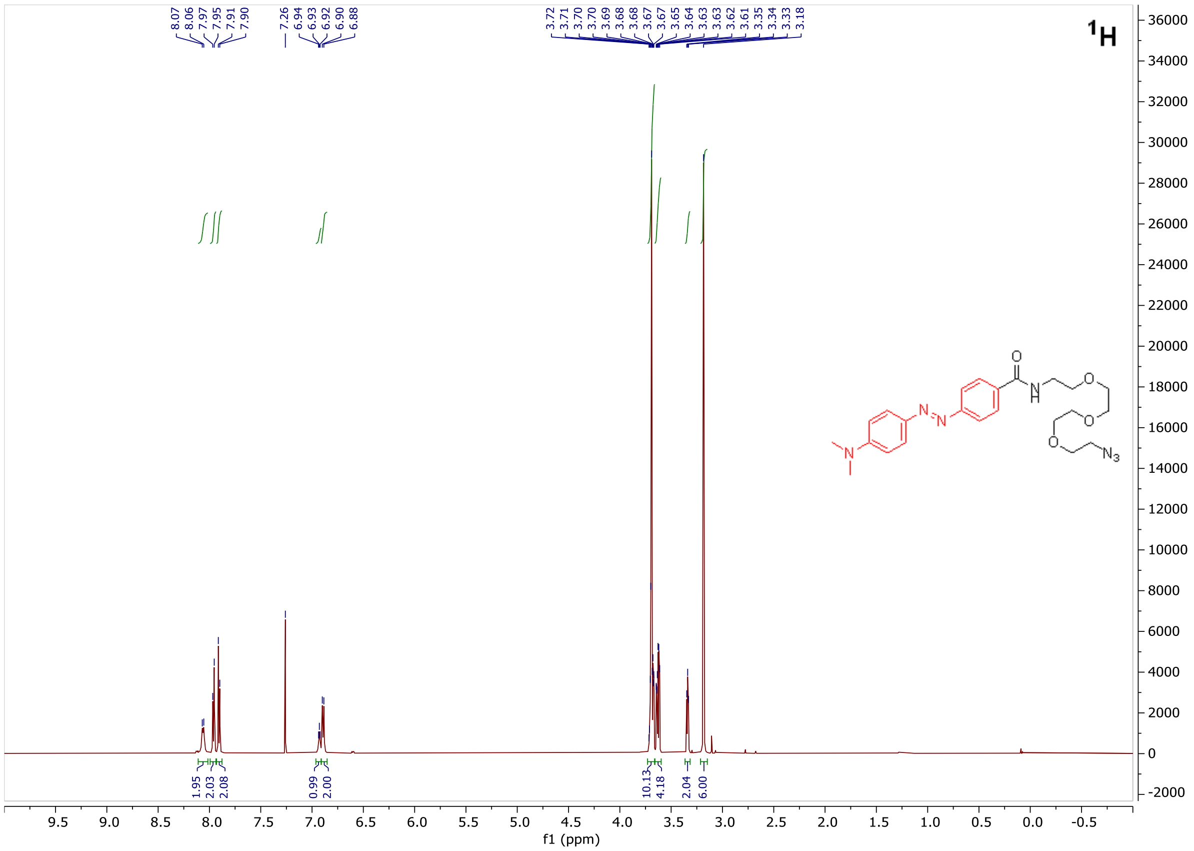

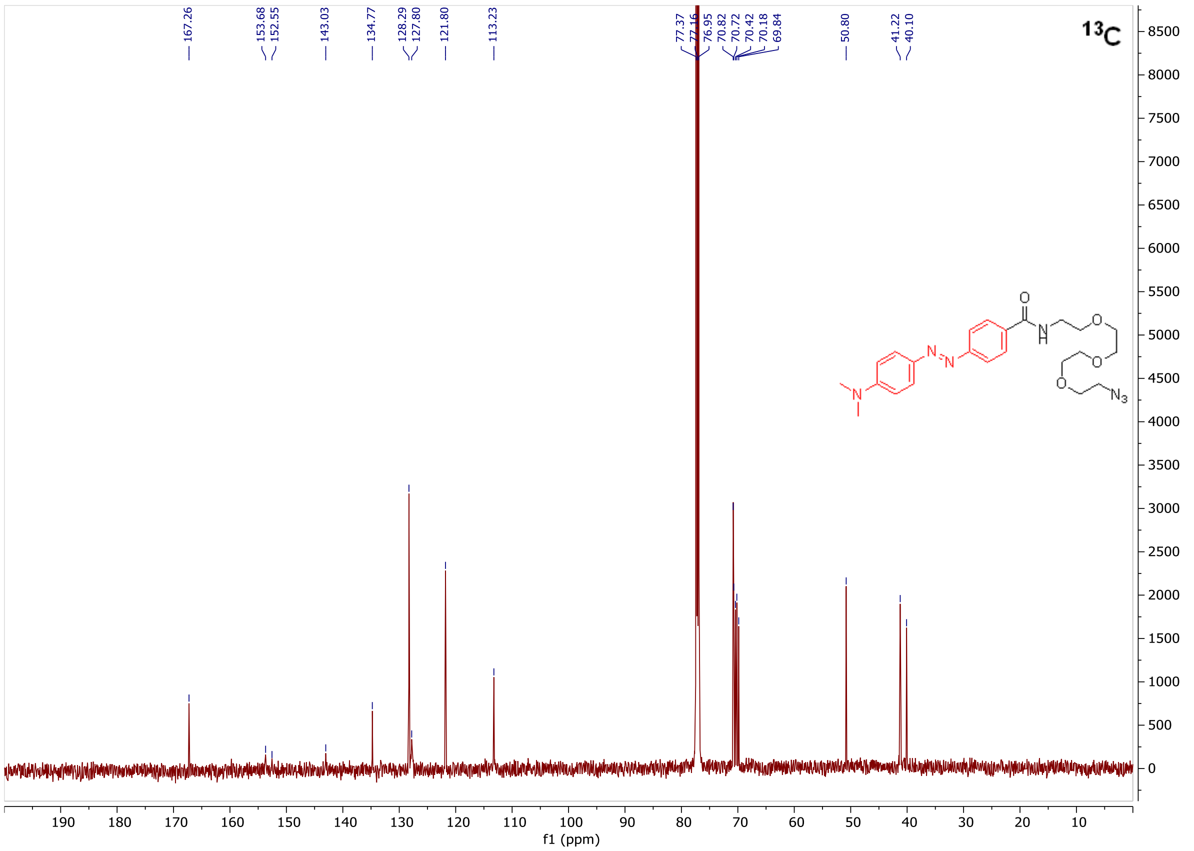

^{1}H, ^{13}C, ^{31}P and (^{1}H,^{13}C)-HSQC spectra of 15 (DMSO-d$_6$)

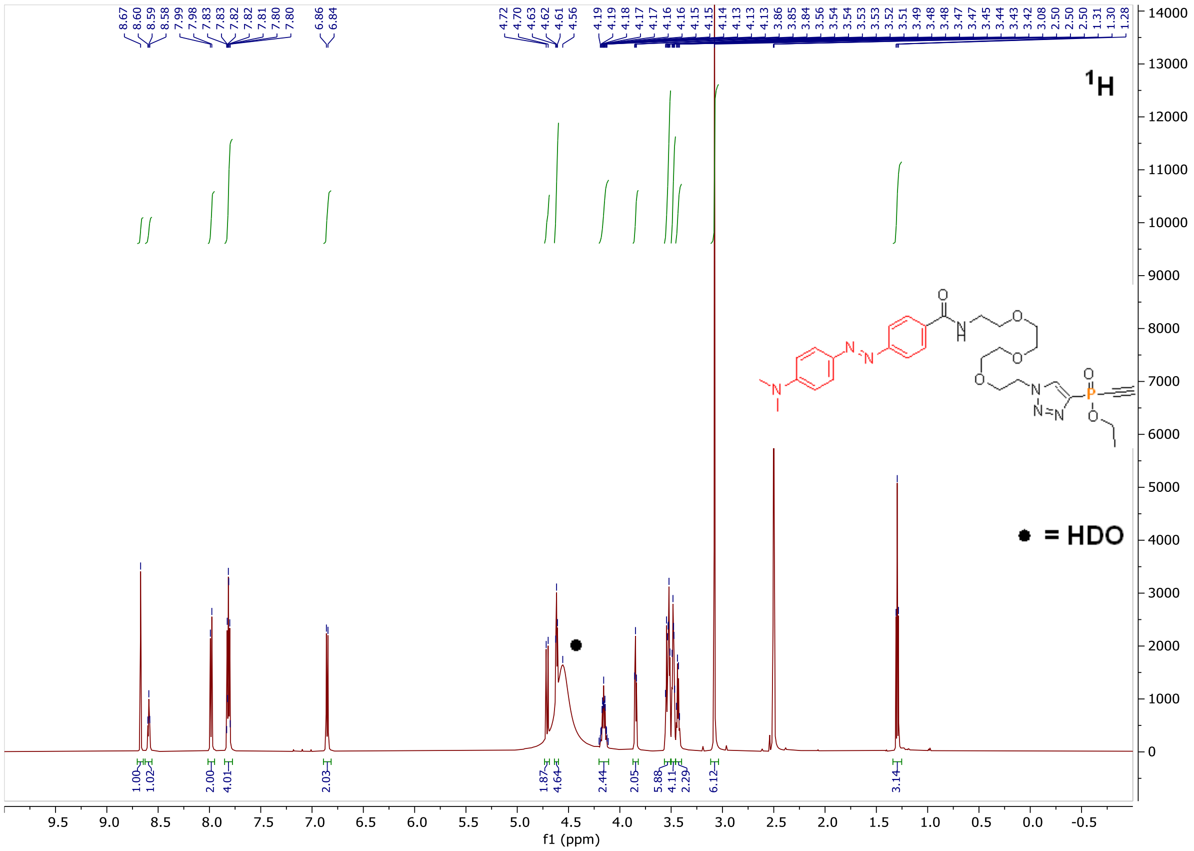

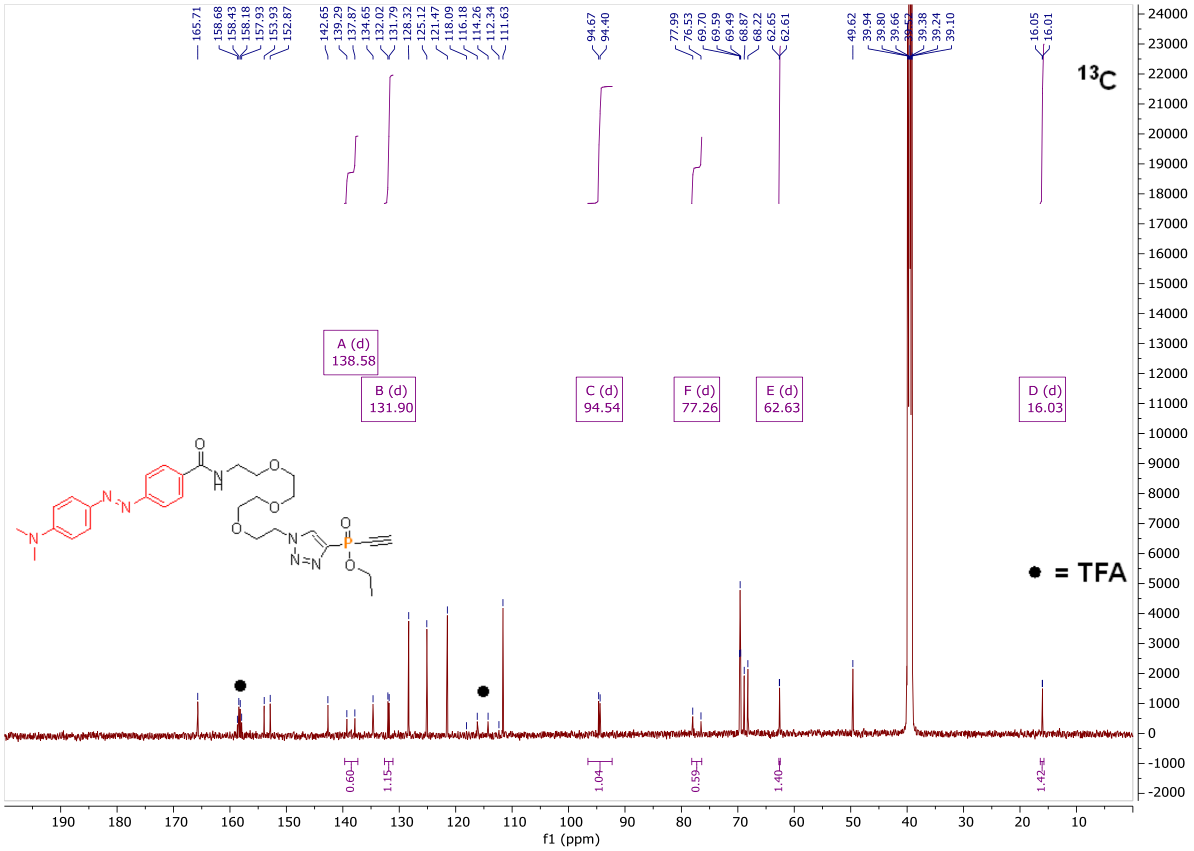

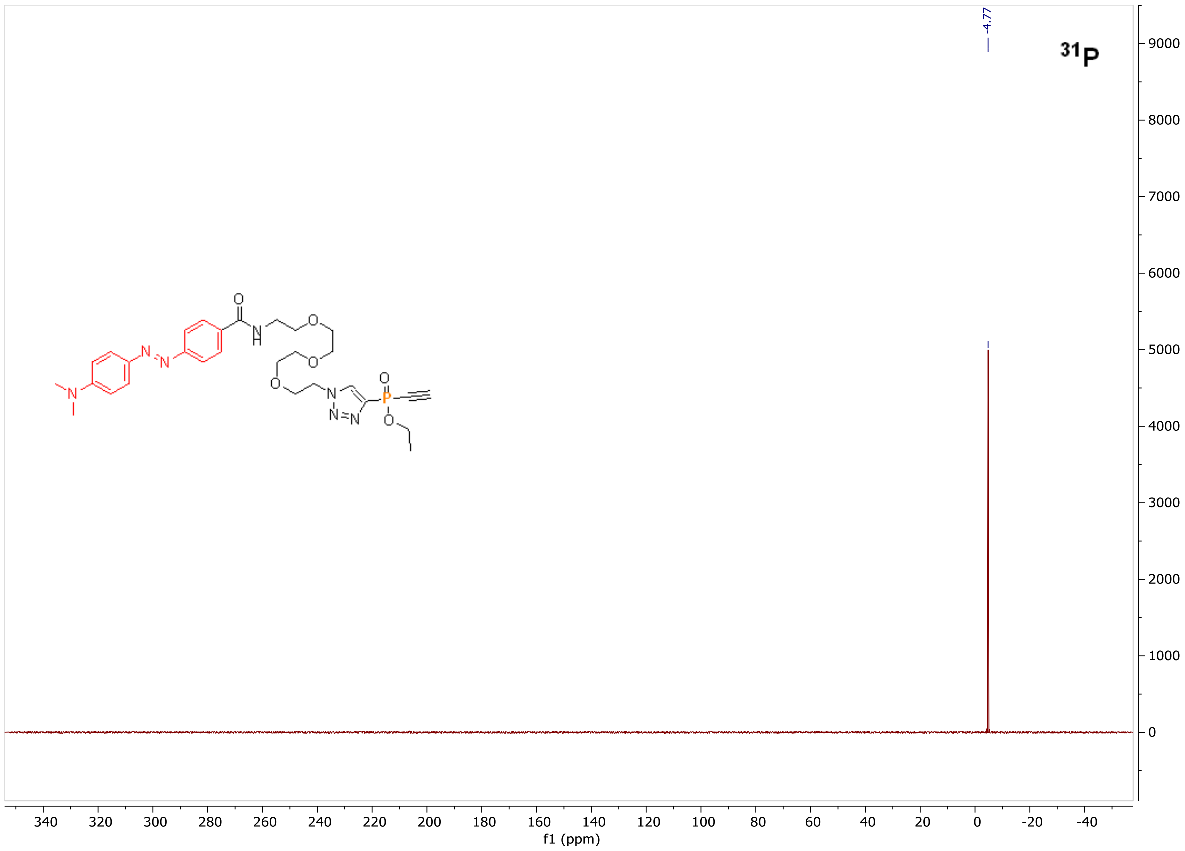
31P
— -4.77
f1 (ppm)

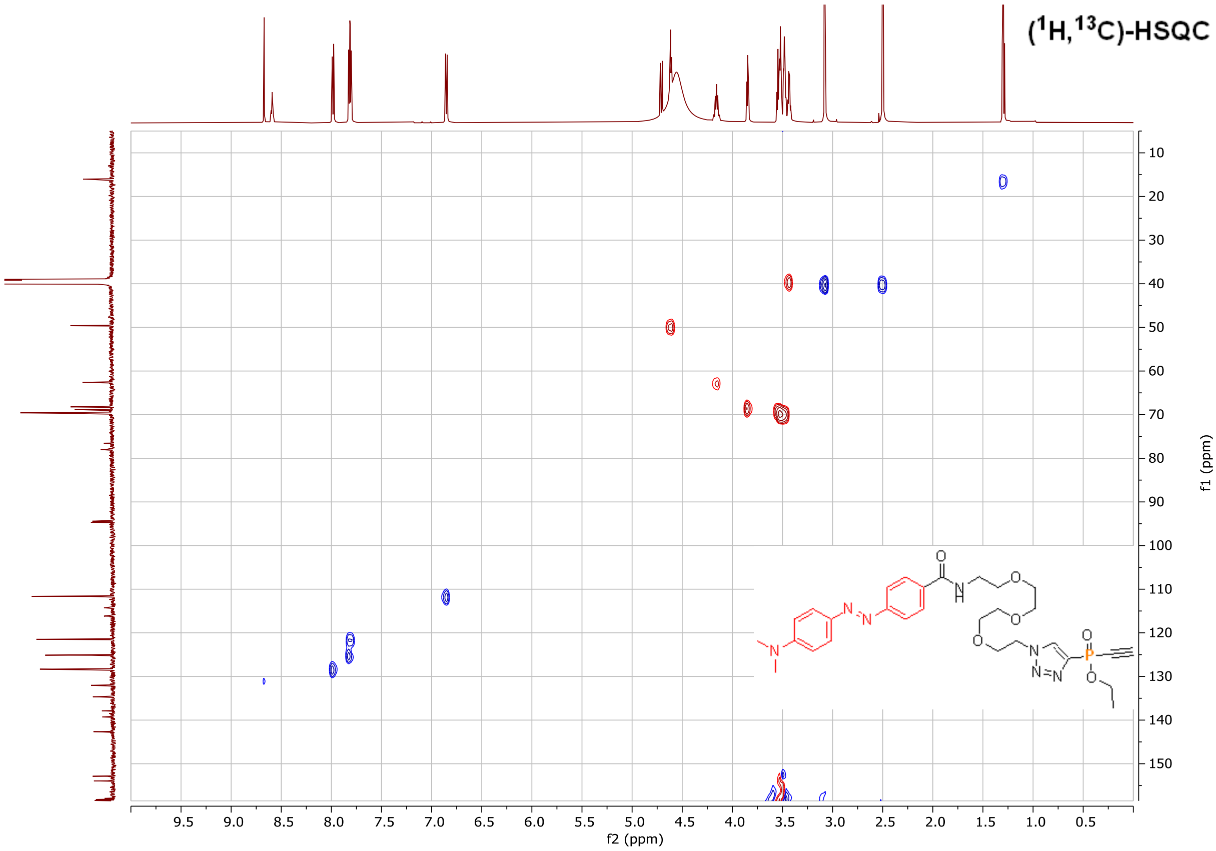
(1H,13C)-HSQC
f2 (ppm)
f1 (ppm)

^1H, ^{13}C-APT, ^1H-COSY and (^1H,^{13}C)-HSQC spectra of 21 (MeOD)

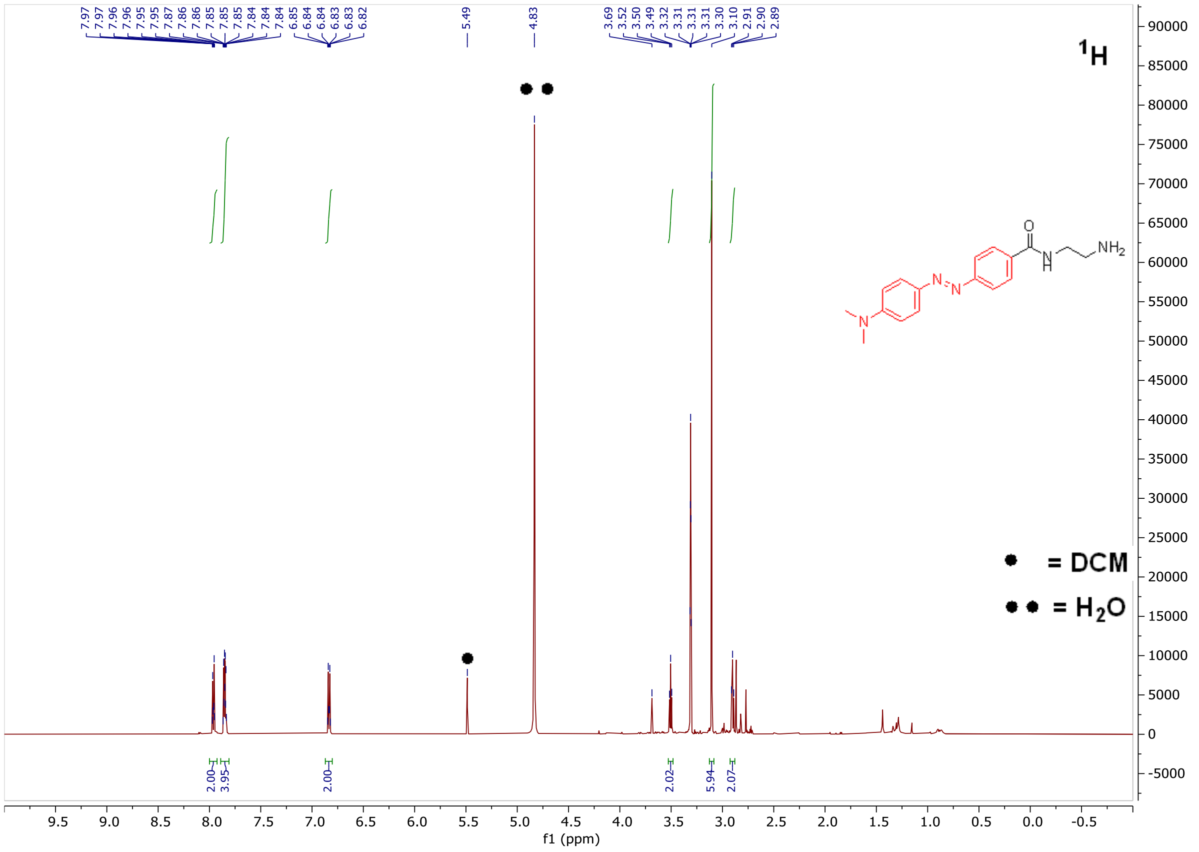

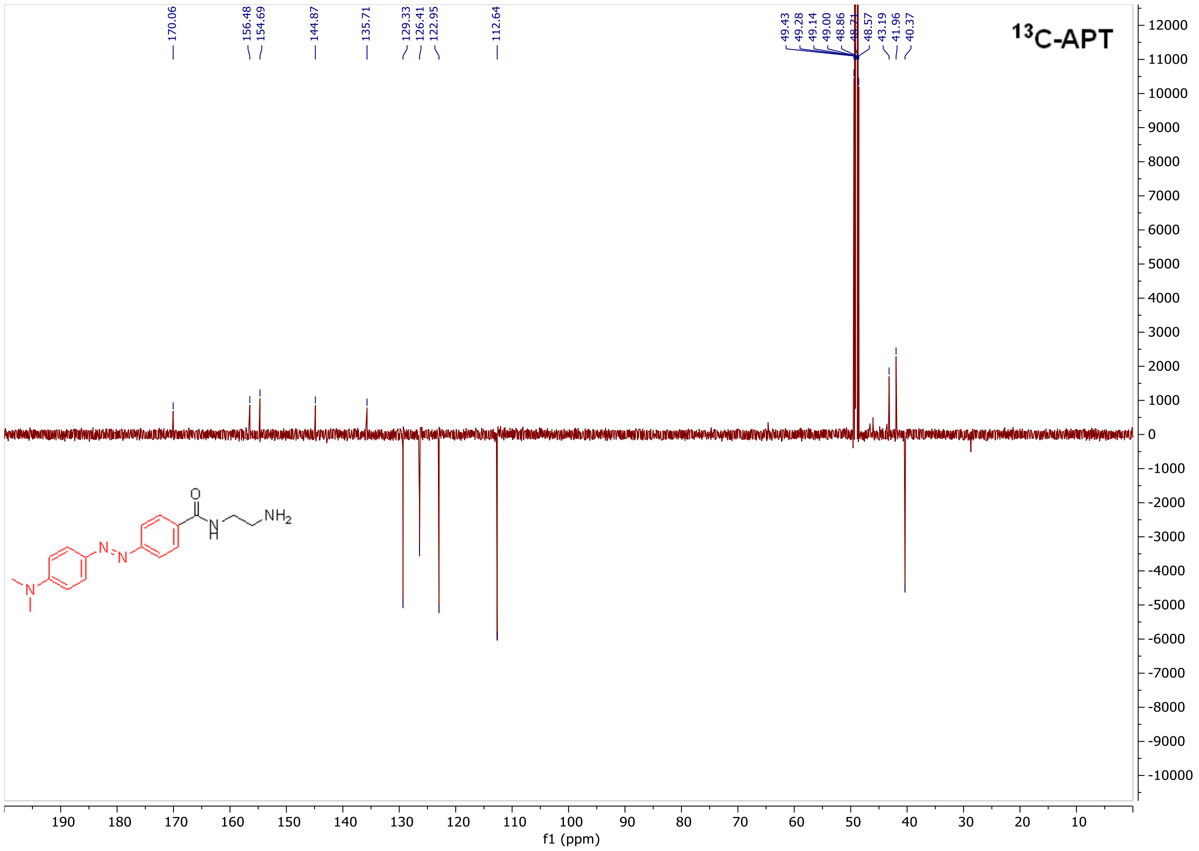

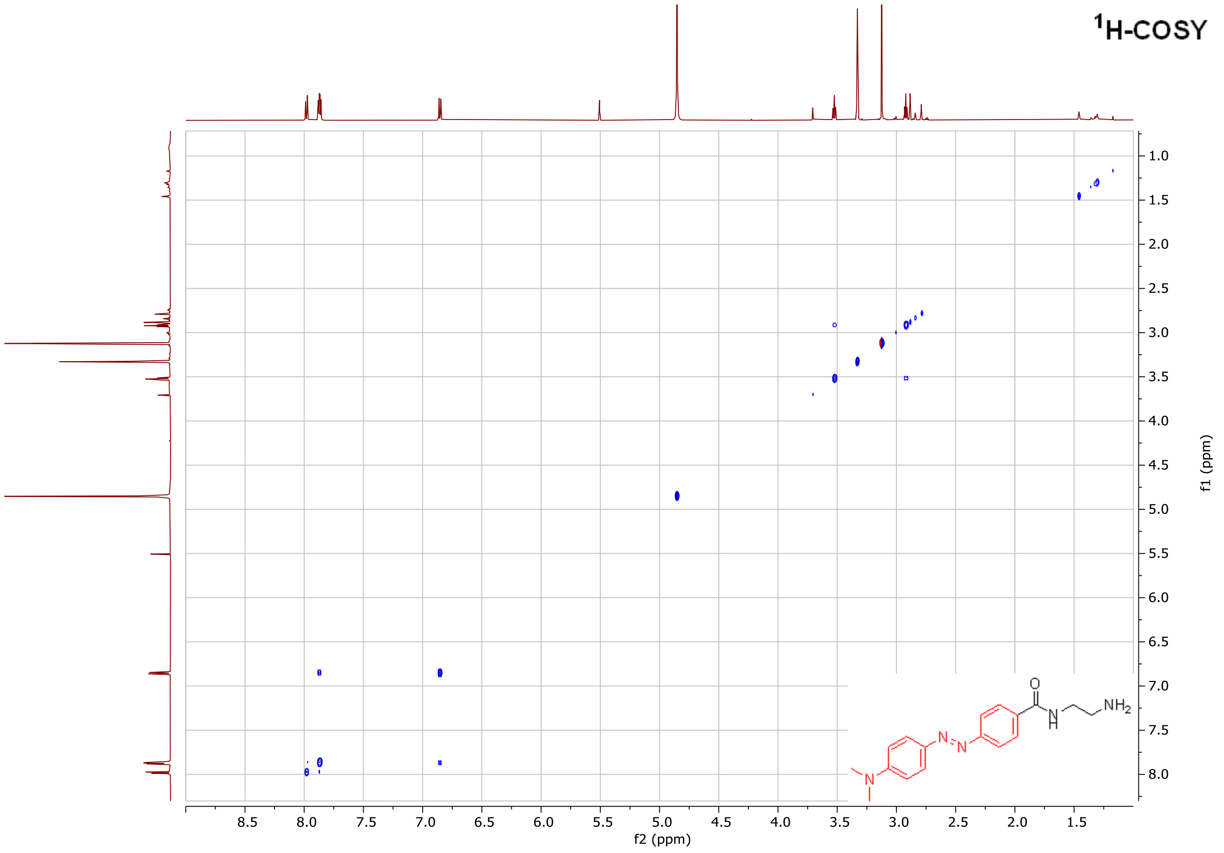

^{1}H-COSY
f1 (ppm)
f2 (ppm)
NH$_2$

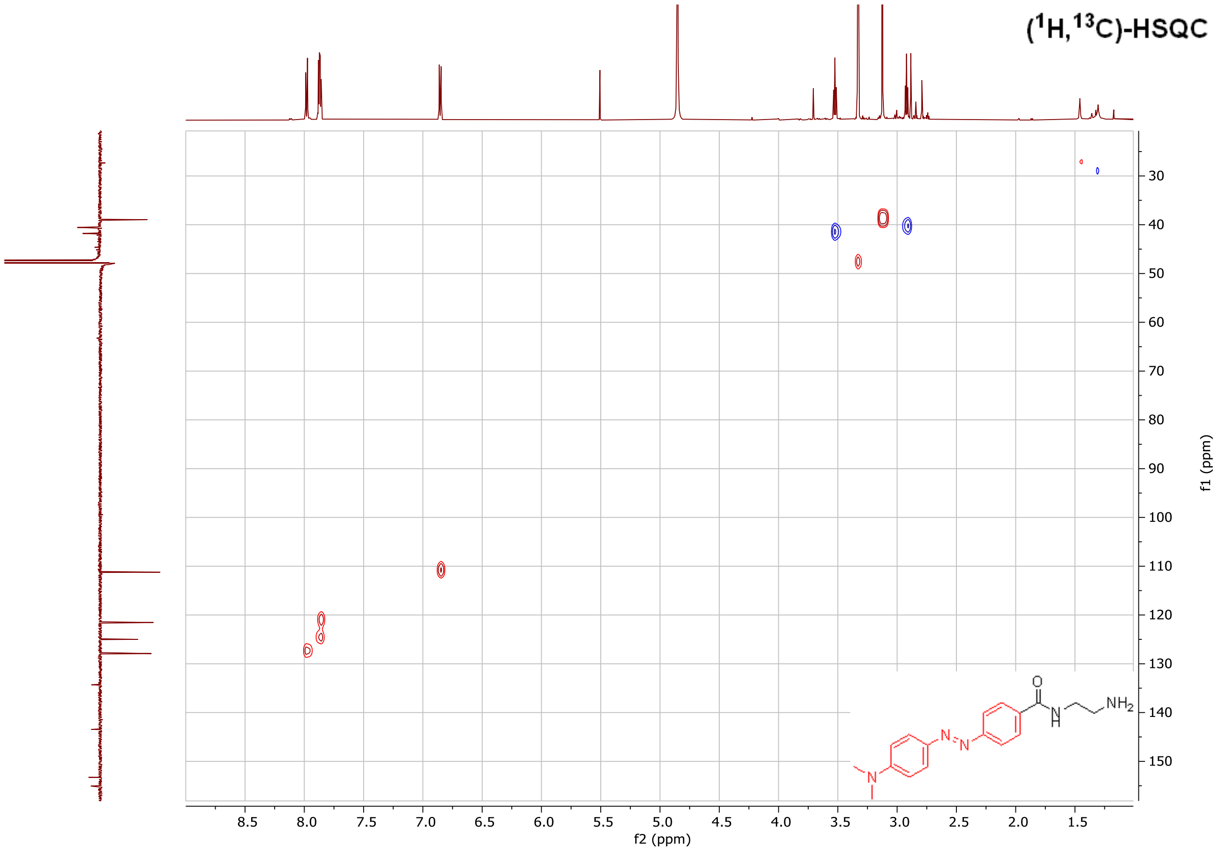

(^{1}H,^{13}C)-HSQC
f1 (ppm)
f2 (ppm)
NH$_2$

¹H, ¹³C, ¹H-COSY and (¹H,¹³C)-HSQC spectra of 23 (DMSO-d$_6$)

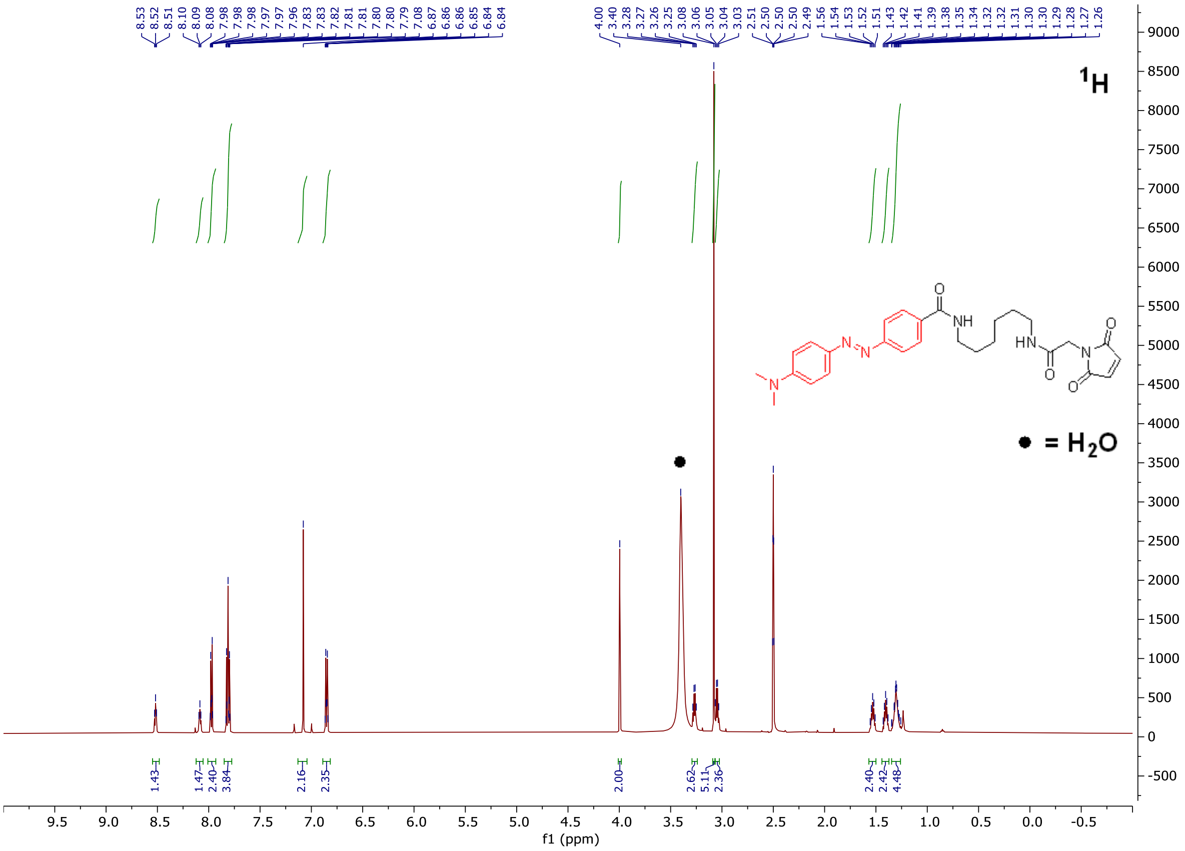

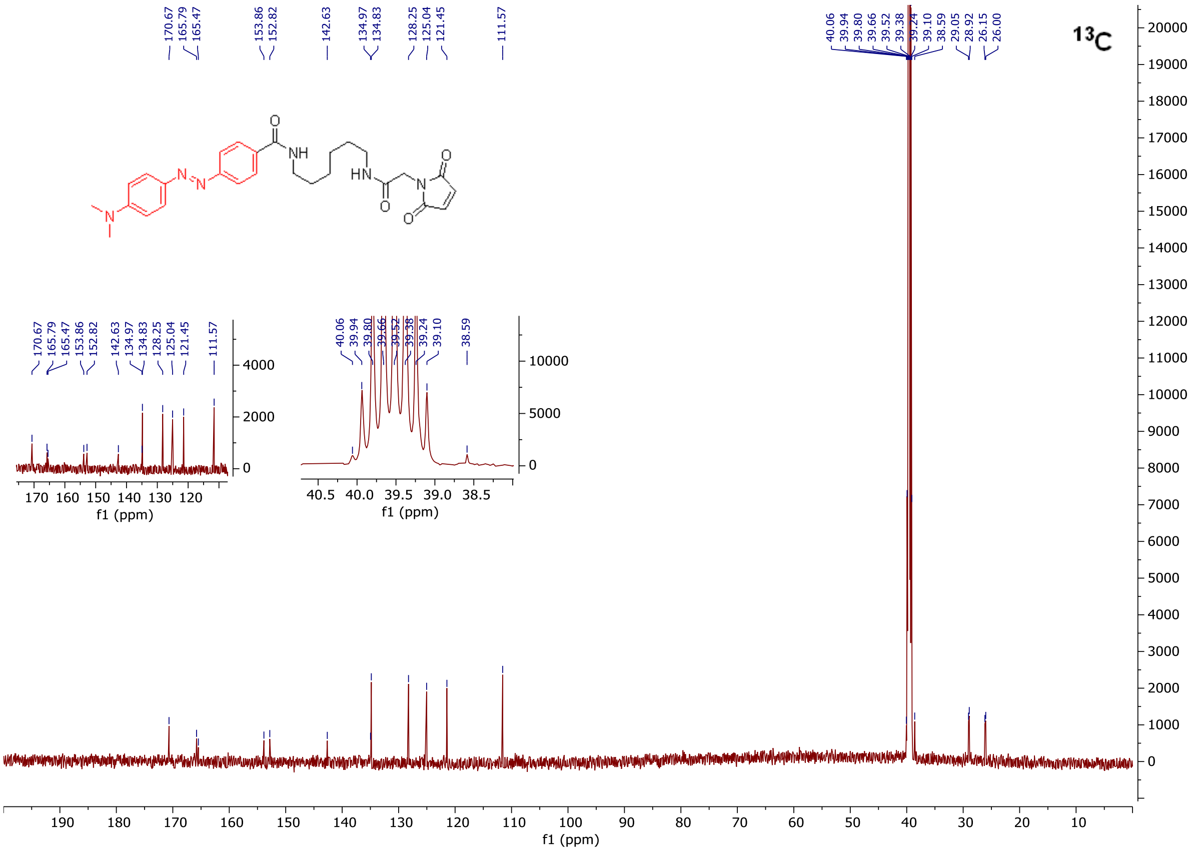

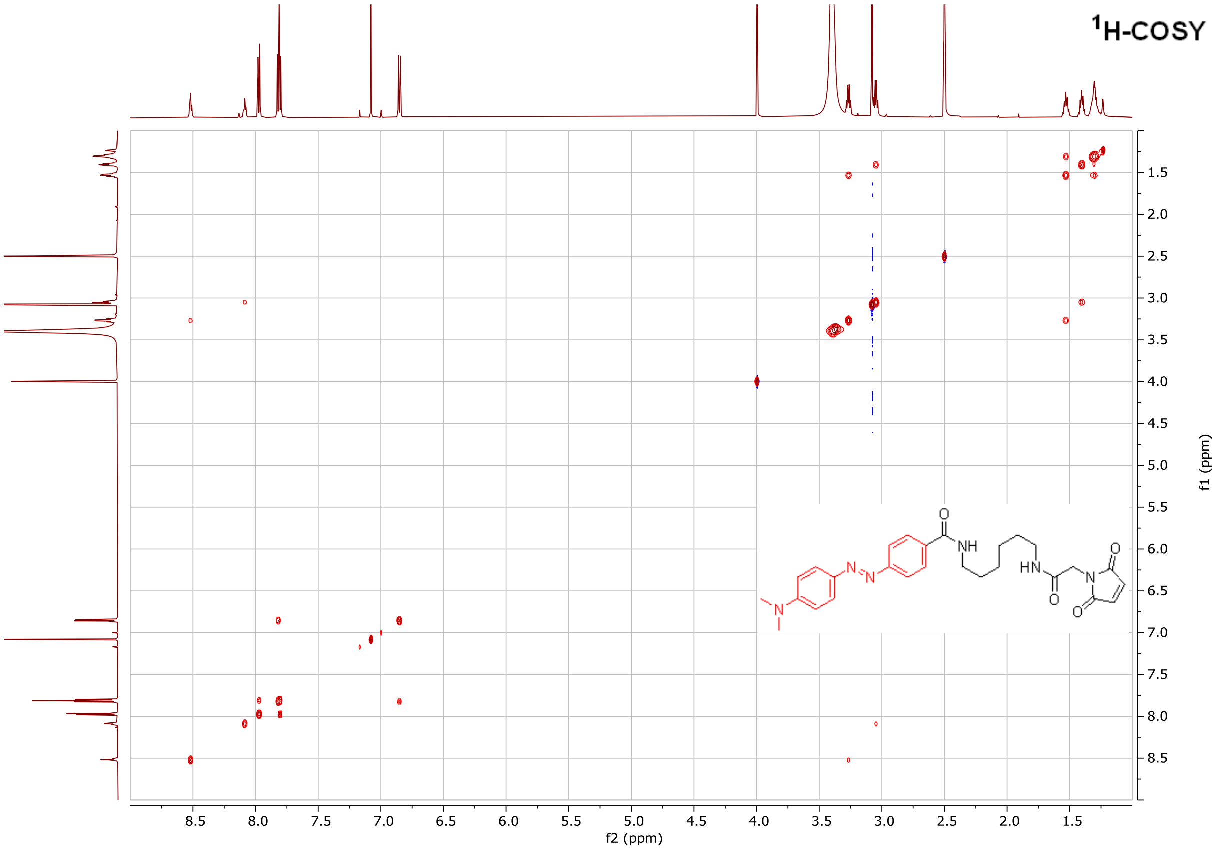

1H-COSY
f1 (ppm)
f2 (ppm)

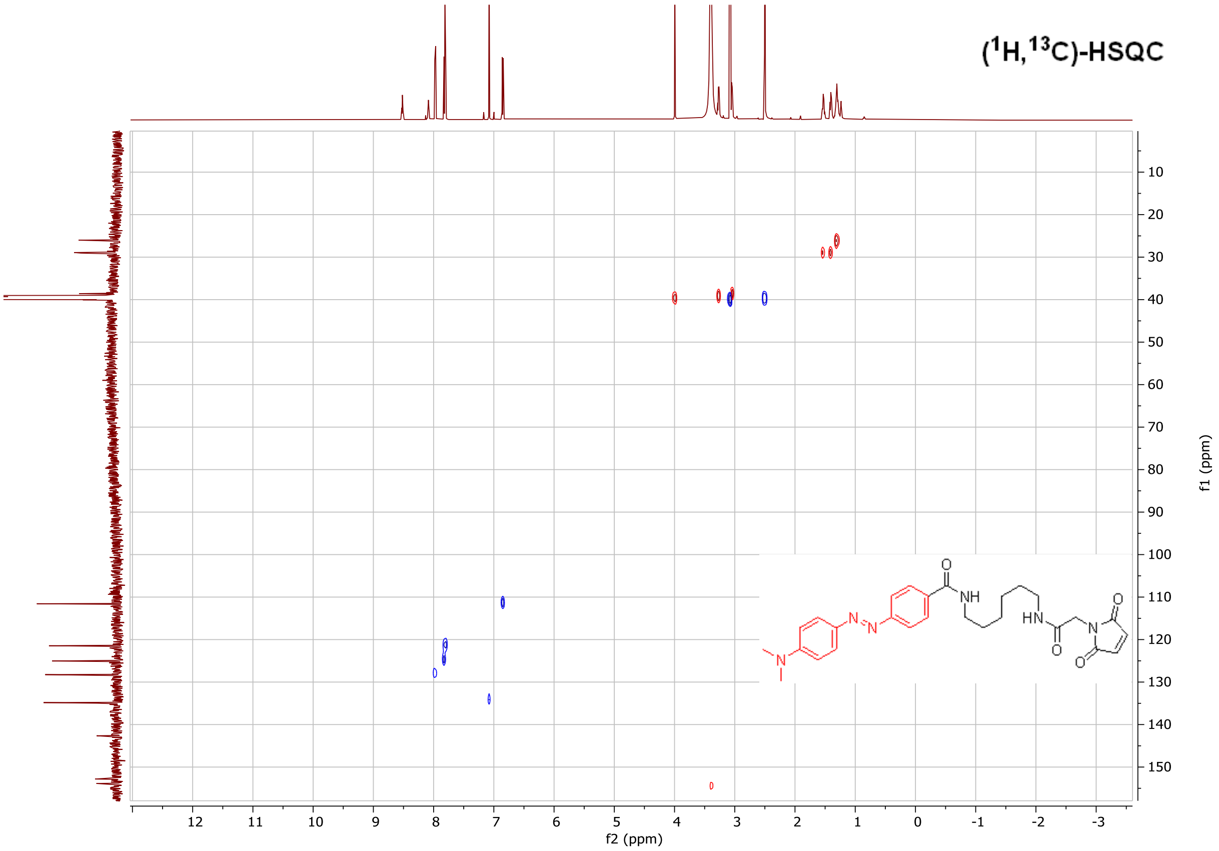

(1H,13C)-HSQC
f1 (ppm)
f2 (ppm)

^1H, ^{13}C, ^1H-COSY and (^1H,^{13}C)-HSQC spectra of 30 (DMSO-d$_6$)

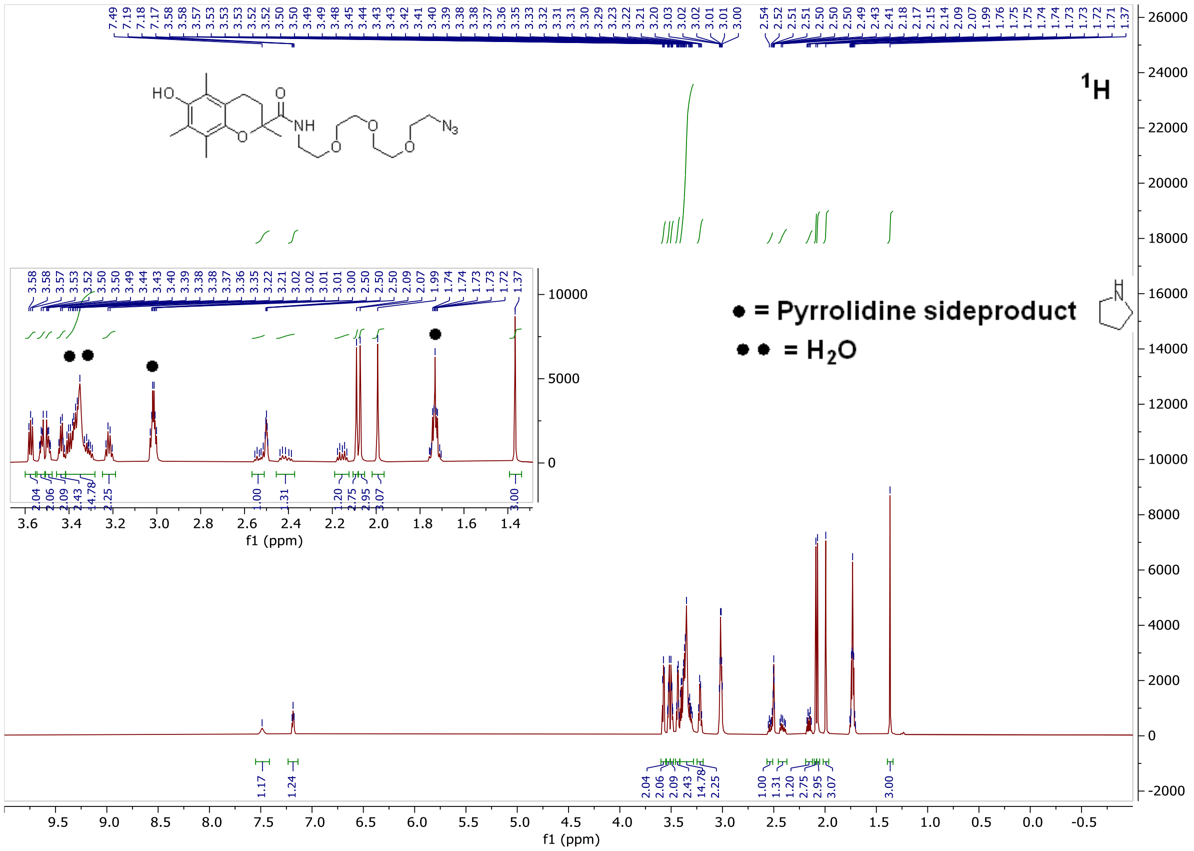

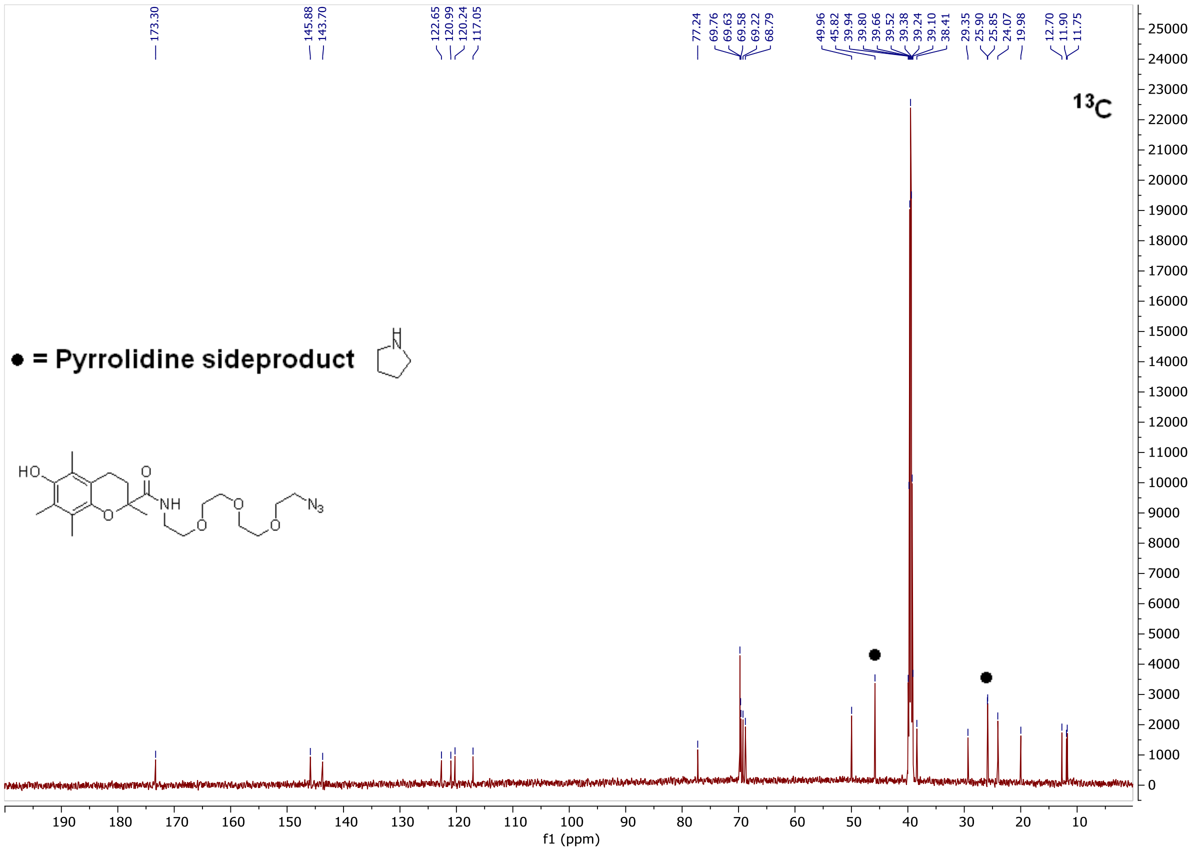

^{1}H-COSY

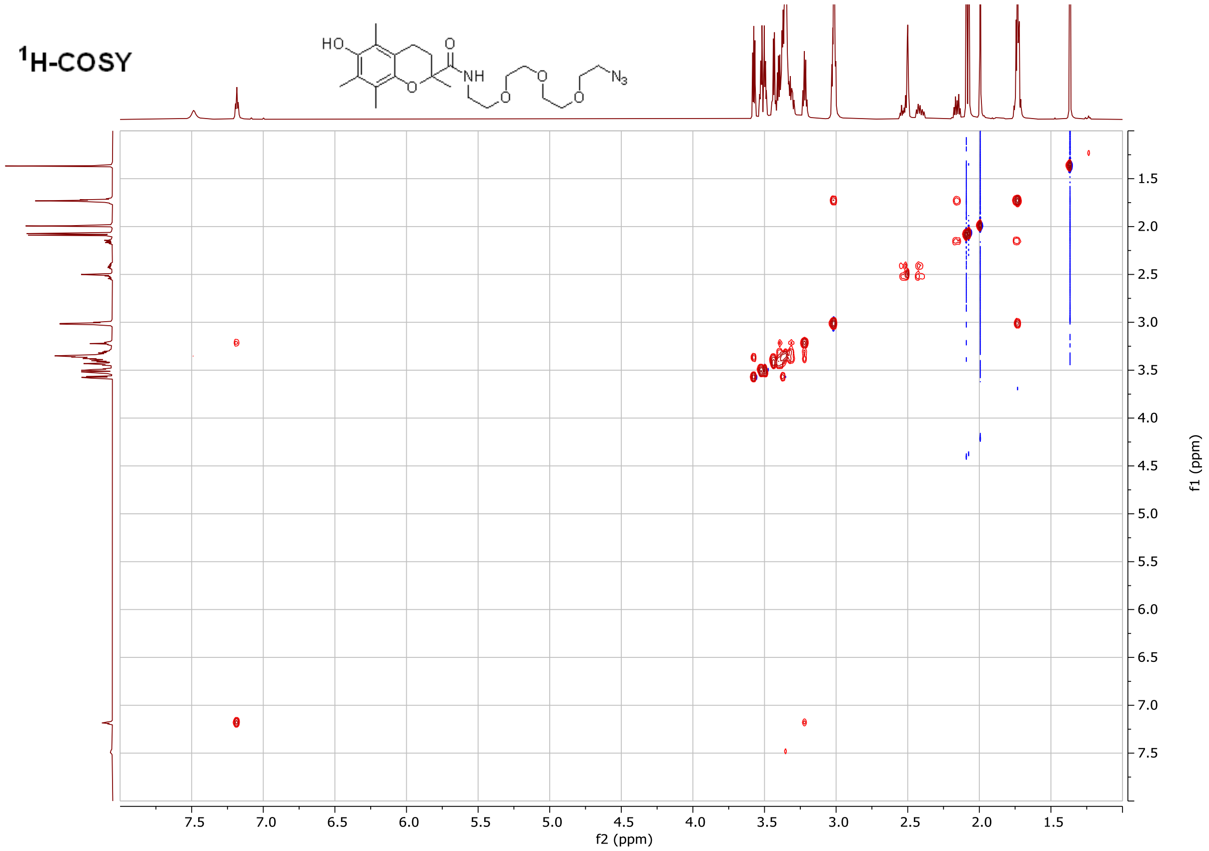

(^{1}H,^{13}C)-HSQC

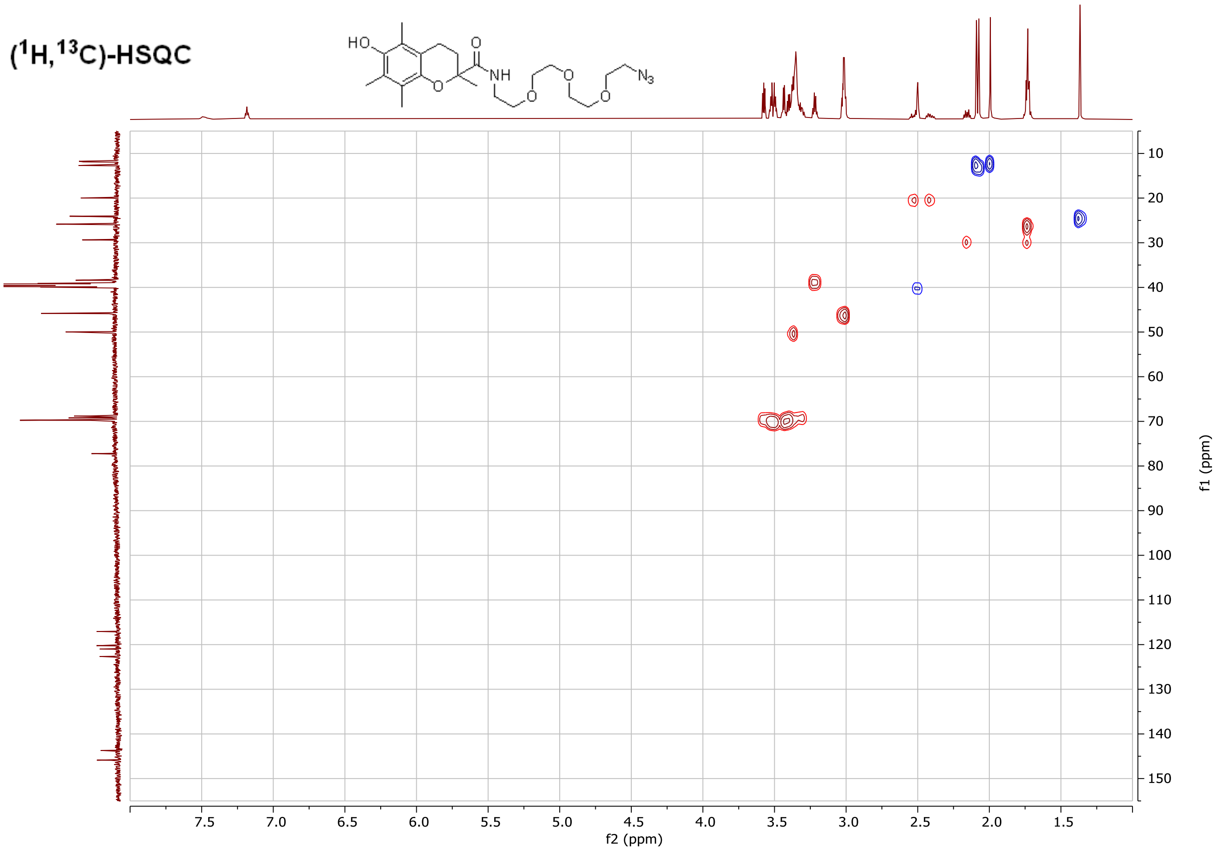

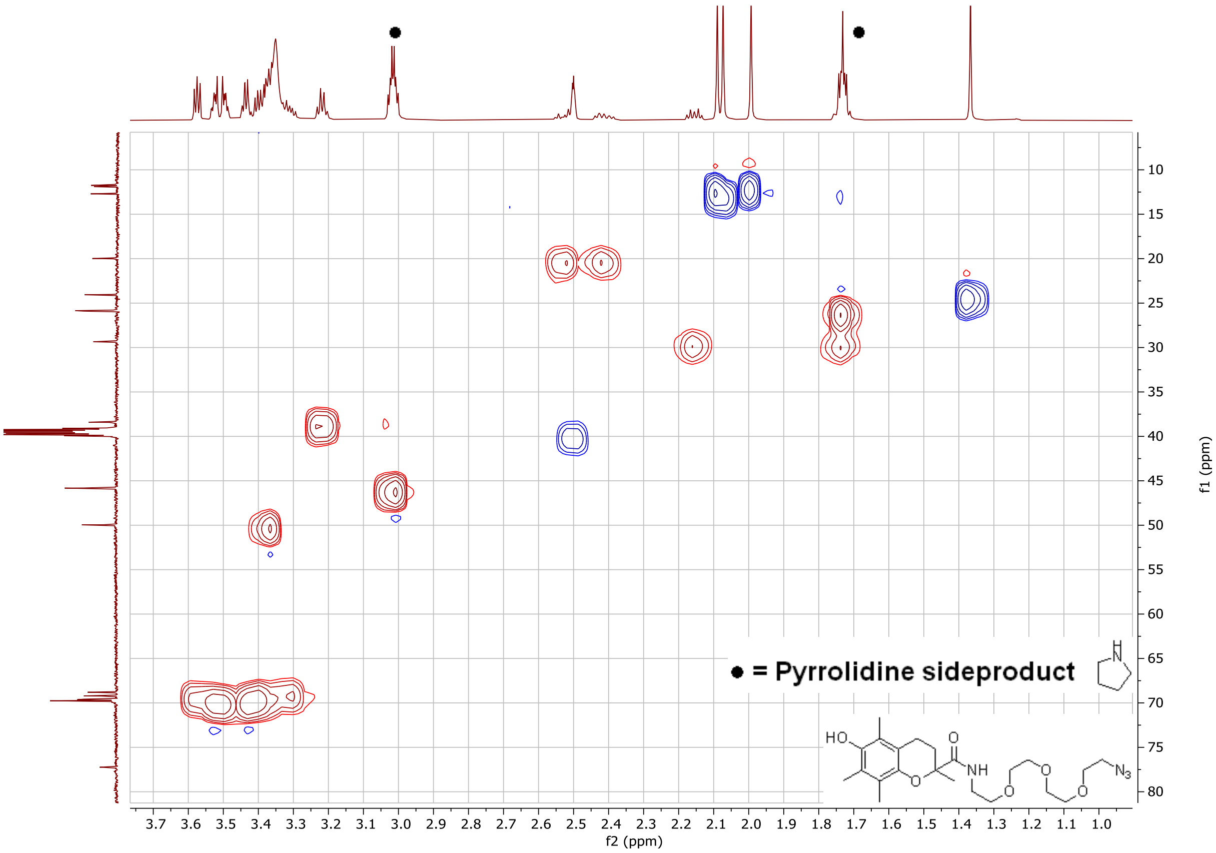
● = Pyrrolidine sideproduct
f2 (ppm)
f1 (ppm)
HO
O
NH
O
O
O
N3

^1H, ^{13}C, ^{31}P, ^1H-COSY and (^1H,^{13}C)-HSQC spectra of 31 (DMSO-d$_6$)

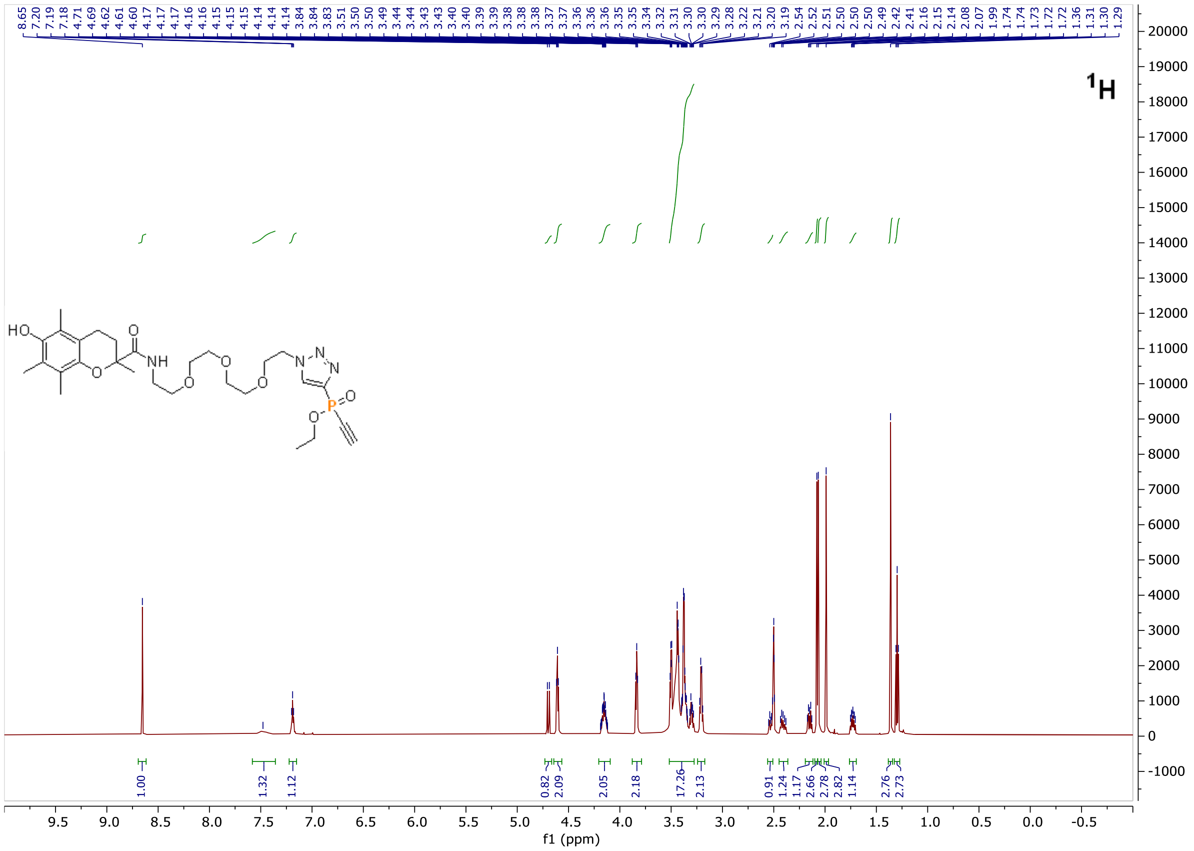

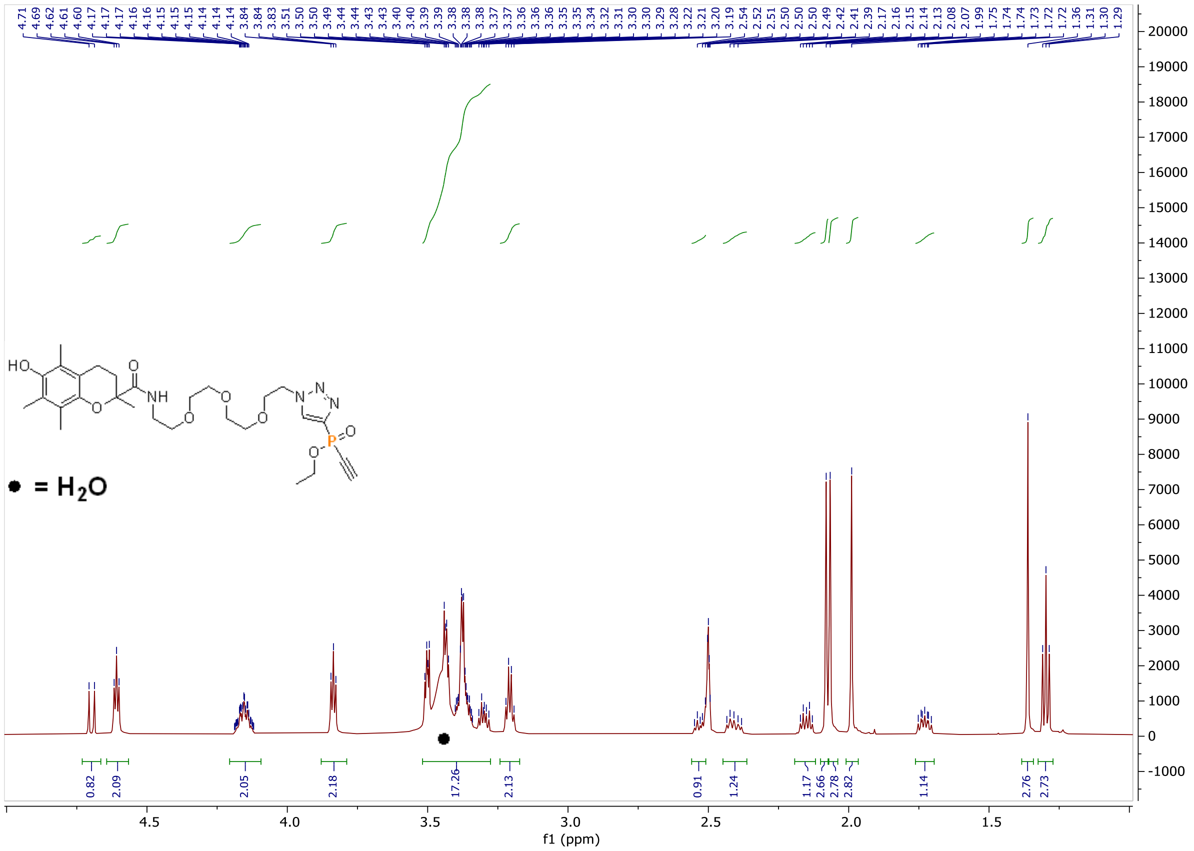

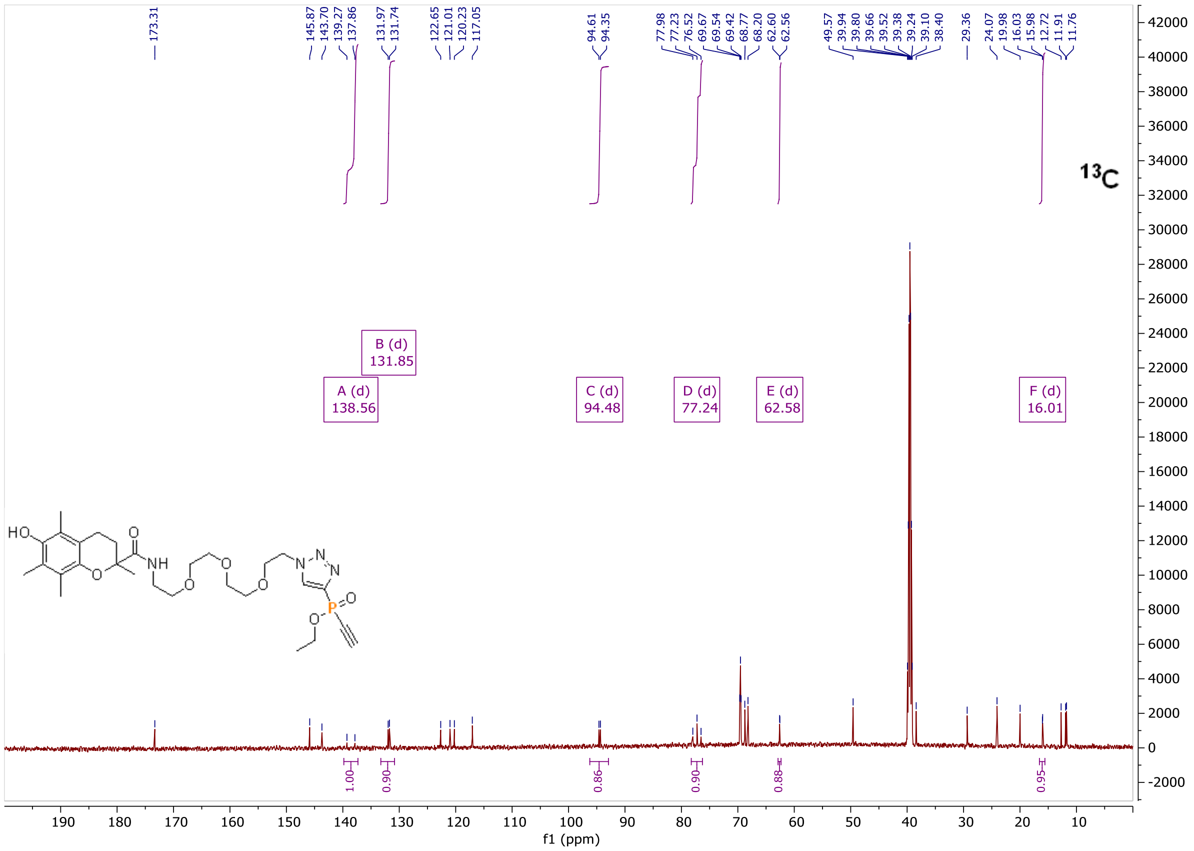
13C
173.31
145.87
143.70
139.27
137.86
131.97
131.74
122.65
121.01
120.23
117.05
94.61
94.35
77.98
77.23
76.52
69.67
69.54
69.42
68.77
68.20
62.60
62.56
49.57
39.94
39.80
39.66
39.52
39.38
39.24
39.10
38.40
29.36
24.07
19.98
16.03
15.98
12.72
11.91
11.76
A (d) 138.56
B (d) 131.85
C (d) 94.48
D (d) 77.24
E (d) 62.58
F (d) 16.01
1.00
0.90
0.86
0.90
0.88
0.95
HO
O
NH
O
O
O
N
N
N
P
O
O
f1 (ppm)

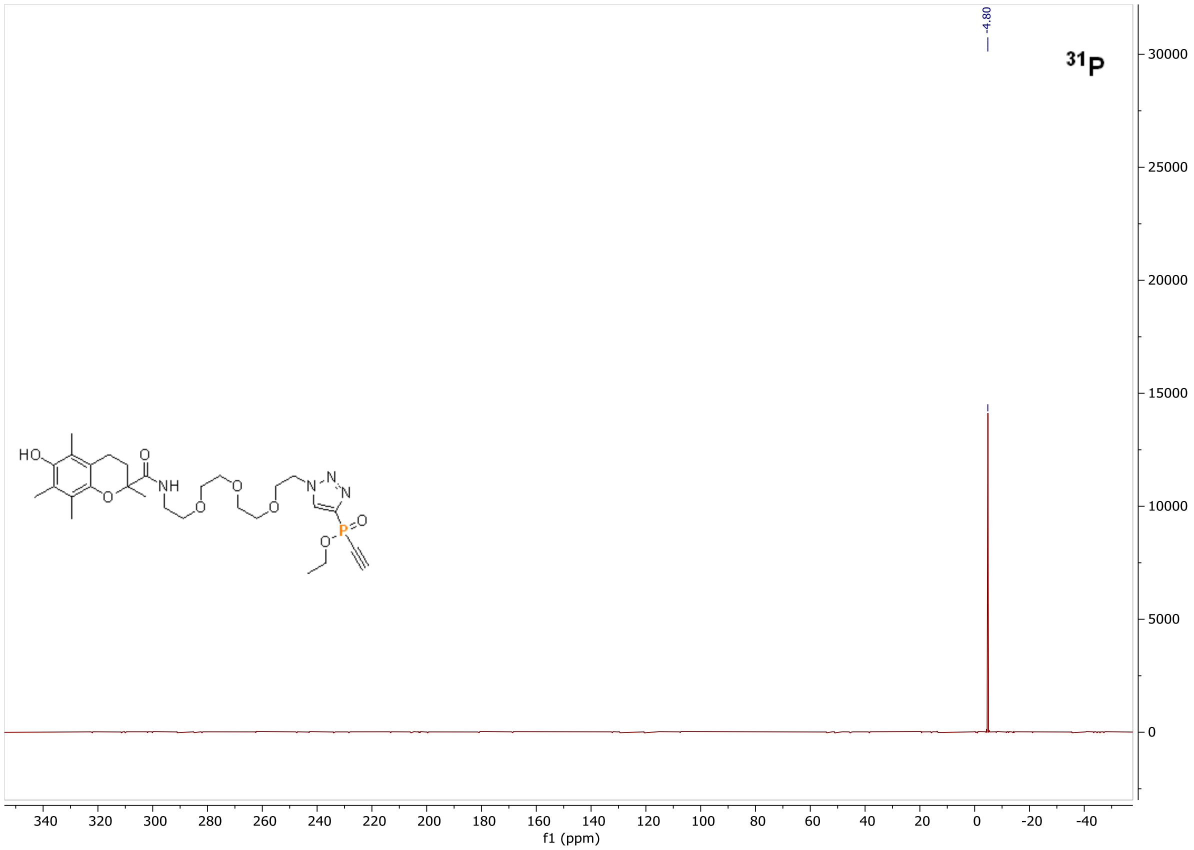
31P
-4.80
HO
O
NH
O
O
O
N
N
N
P
O
O
f1 (ppm)

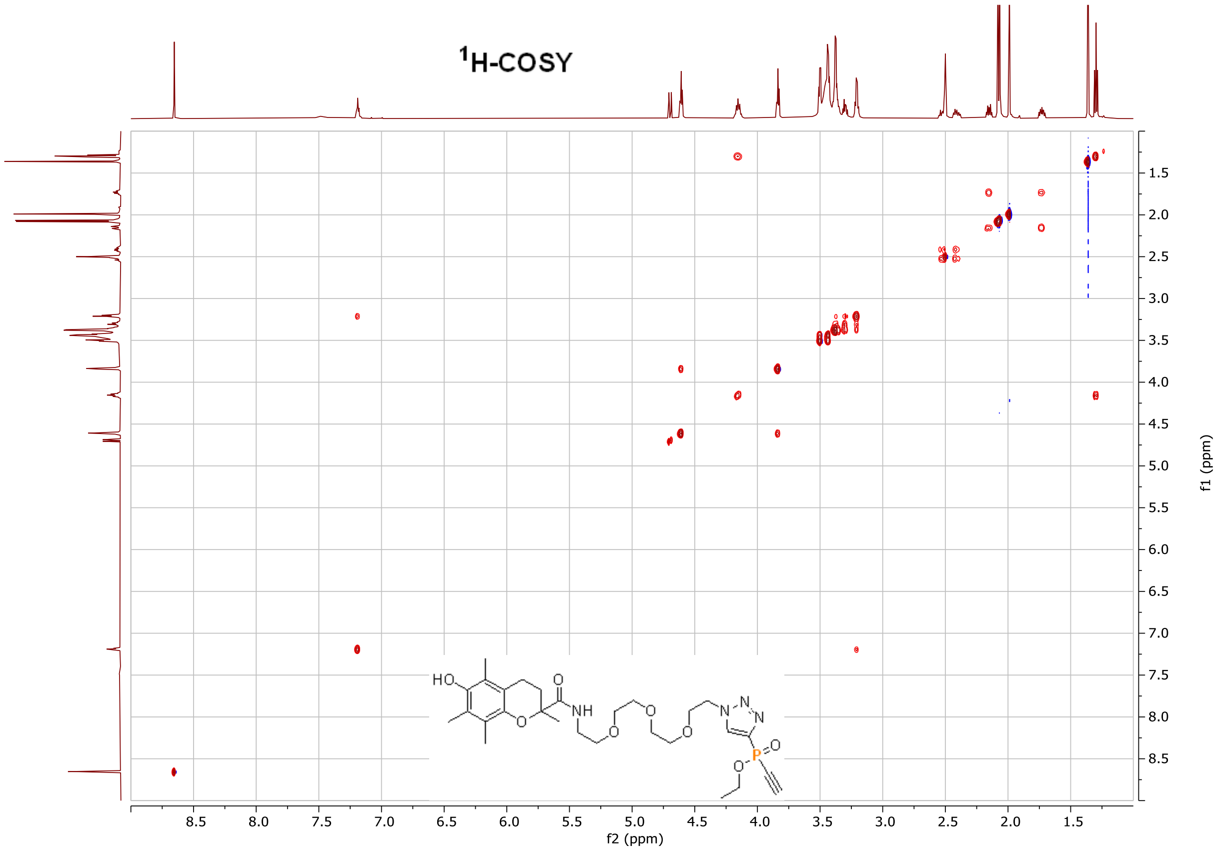

¹H-COSY
HO
O
NH
O
O
O
N
N
N
P
O
O
f2 (ppm)
f1 (ppm)

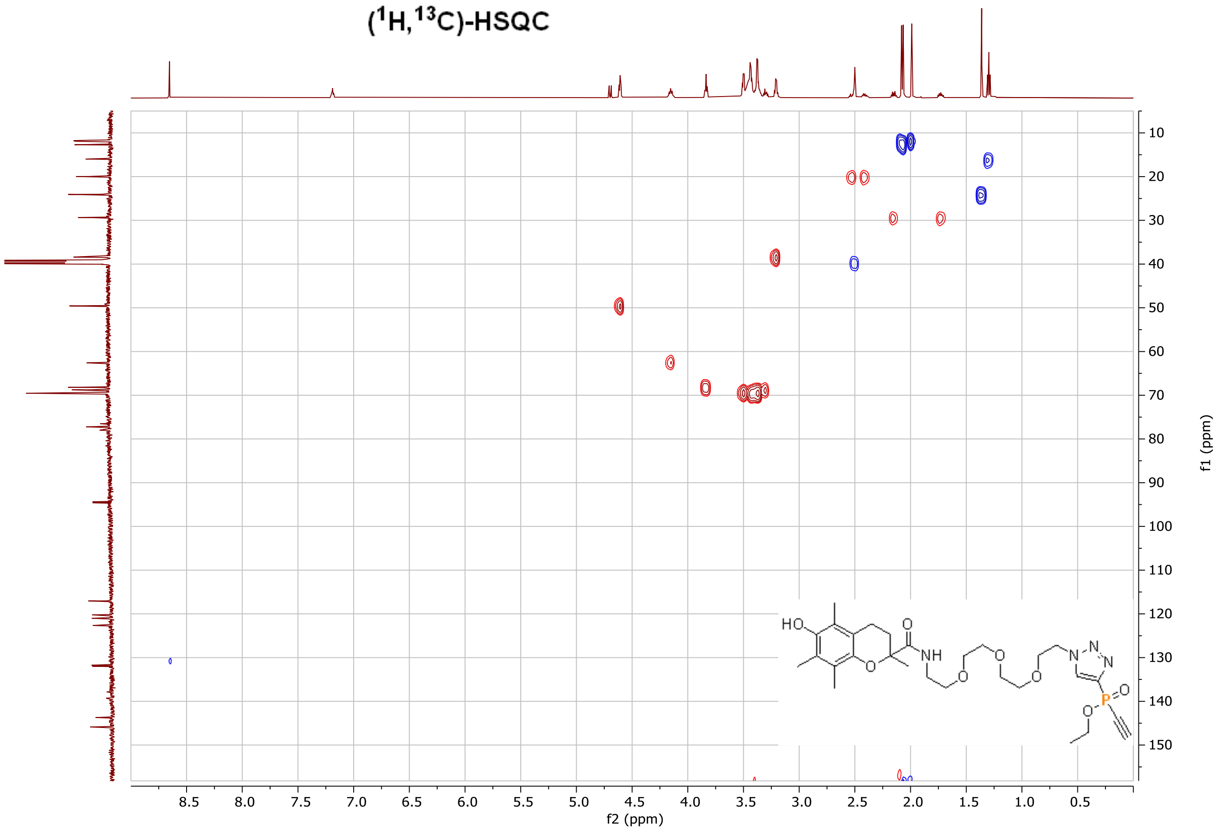

(¹H,¹³C)-HSQC
HO
O
NH
O
O
O
N
N
N
P
O
O
f2 (ppm)
f1 (ppm)

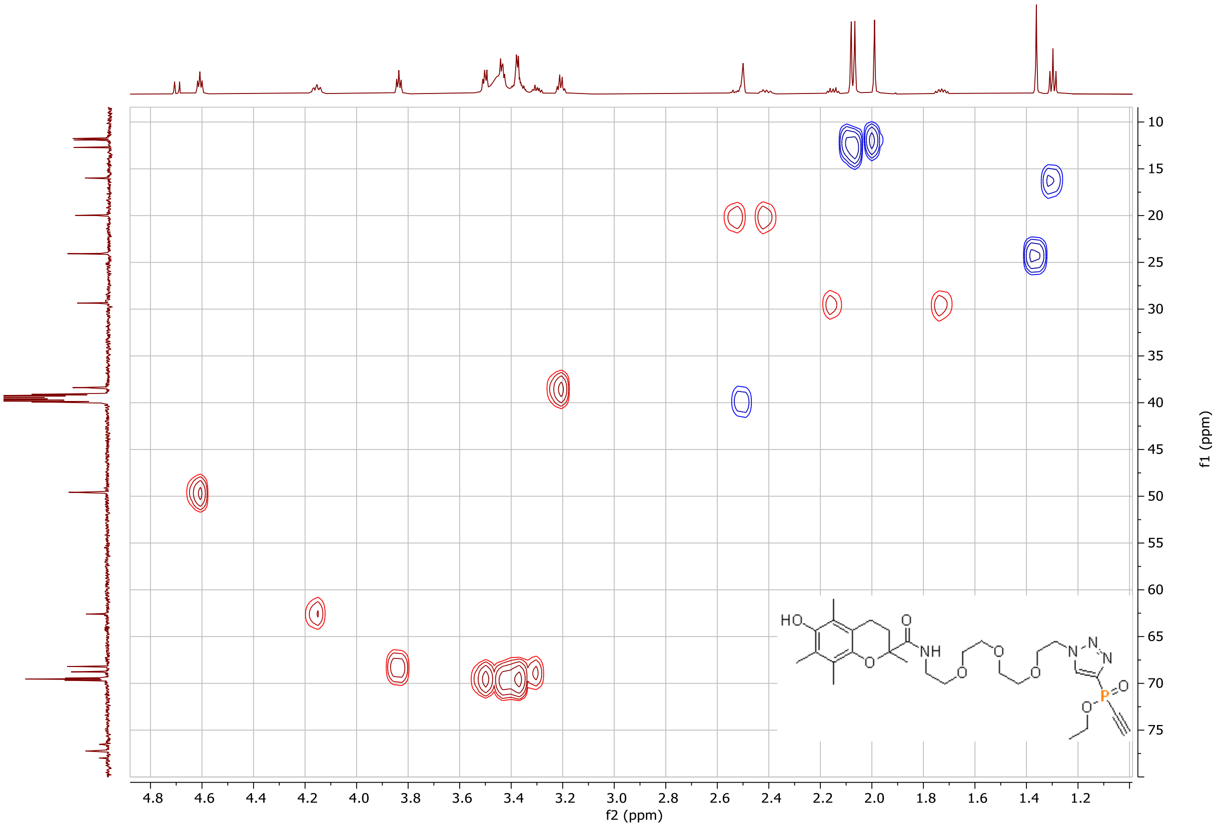

f1 (ppm)
f2 (ppm)
HO
O
NH
O
O
O
N
N
N
P
O
O

10. Supporting References

(1) Stieger, C. E.; Franz, L.; Körlin, F.; Hackenberger, C. P. R. Diethynyl Phosphinates for Cysteine-Selective Protein Labeling and Disulfide Rebridging. *Angew. Chem. Int. Ed.* **2021**, *60* (28), 15359–15364. https://doi.org/10.1002/anie.202100683.

(2) Stieger, C. E.; Park, Y.; de Geus, M. A. R.; Kim, D.; Huhn, C.; Slenczka, J. S.; Ochtrop, P.; Müchler, J. M.; Süssmuth, R. D.; Broichhagen, J.; Baik, M.; Hackenberger, C. P. R. DFT-Guided Discovery of Ethynyl-Triazolyl-Phosphinates as Modular Electrophiles for Chemoselective Cysteine Bioconjugation and Profiling. *Angew. Chem. Int. Ed.* **2022**, *61* (41), e202205348. https://doi.org/10.1002/anie.202205348.

(3) Escher, E.; Bernier, M.; Parent, P. Angiotensin II Analogues. Part II. Synthesis and Incorporation of the Sulfur-containing Aromatic Amino Acids: L-(4′-SH)Phe, L-(4′-SO2NH2)Phe, L-(4′-SO3-)Phe and L-(4′-S-CH3)Phe. *Helv. Chim. Acta* **1983**, *66* (5), 1355–1365. https://doi.org/10.1002/hlca.19830660504.

(4) Lu, H. S. M.; Volk, M.; Kholodenko, Y.; Gooding, E.; Hochstrasser, R. M.; DeGrado, W. F. Aminothiotyrosine Disulfide, an Optical Trigger for Initiation of Protein Folding. *J. Am. Chem. Soc.* **1997**, *119* (31), 7173–7180. https://doi.org/10.1021/ja970567o.

(5) Schumacher, D.; Helma, J.; Mann, F. A.; Pichler, G.; Natale, F.; Krause, E.; Cardoso, M. C.; Hackenberger, C. P. R.; Leonhardt, H. Versatile and Efficient Site-Specific Protein Functionalization by Tubulin Tyrosine Ligase. *Angew. Chem. Int. Ed.* **2015**, *54* (46), 13787–13791. https://doi.org/10.1002/anie.201505456.

(6) Schumacher, D.; Lemke, O.; Helma, J.; Gerszonowicz, L.; Waller, V.; Stoschek, T.; Durkin, P. M.; Budisa, N.; Leonhardt, H.; Keller, B. G.; Hackenberger, C. P. R. Broad Substrate Tolerance of Tubulin Tyrosine Ligase Enables One-Step Site-Specific Enzymatic Protein Labeling. *Chem. Sci.* **2017**, *8* (5), 3471–3478. https://doi.org/10.1039/C7SC00574A.

(7) Siebertz, K. D.; Hackenberger, C. P. R. Chemoselective Triazole-Phosphonamidate Conjugates Suitable for Photorelease. *Chem. Commun.* **2018**, *54* (7), 763–766. https://doi.org/10.1039/C7CC08605A.

(8) de Geus, M. A. R.; Maurits, E.; Sarris, A. J. C.; Hansen, T.; Kloet, M. S.; Kamphorst, K.; ten Hoeve, W.; Robillard, M. S.; Pannwitz, A.; Bonnet, S. A.; Codée, J. D. C.; Filippov, D. V.; Overkleeft, H. S.; van Kasteren, S. I. Fluorogenic Bifunctional Trans -Cyclooctenes as Efficient Tools for Investigating Click-to-Release Kinetics. *Chem. Eur. J.* **2020**, *26* (44), 9900–9904. https://doi.org/10.1002/chem.201905446.

(9) Piotto, M.; Saudek, V.; Sklenář, V. Gradient-Tailored Excitation for Single-Quantum NMR Spectroscopy of Aqueous Solutions. *J. Biomol. NMR* **1992**, *2* (6), 661–665. https://doi.org/10.1007/BF02192855.

(10) Mueller, L. Sensitivity Enhanced Detection of Weak Nuclei Using Heteronuclear Multiple Quantum Coherence. *J. Am. Chem. Soc.* **1979**, *101* (16), 4481–4484. https://doi.org/10.1021/ja00510a007.

(11) Bax, A.; Griffey, R. H.; Hawkins, B. L. Correlation of Proton and Nitrogen-15 Chemical Shifts by Multiple Quantum NMR. *J. Magn. Reson.* **1983**, *55* (2), 301–315. https://doi.org/10.1016/0022-2364(83)90241-X.

(12) Bax, A.; Summers, M. F. Proton and Carbon-13 Assignments from Sensitivity-Enhanced Detection of Heteronuclear Multiple-Bond Connectivity by 2D Multiple Quantum NMR. *J. Am.*

Chem. Soc. **1986**, *108* (8), 2093–2094. https://doi.org/10.1021/ja00268a061.

(13) Cicero, D. O.; Barbato, G.; Bazzo, R. Sensitivity Enhancement of a Two-Dimensional Experiment for the Measurement of Heteronuclear Long-Range Coupling Constants, by a New Scheme of Coherence Selection by Gradients. *J. Magn. Reson.* **2001**, *148* (1), 209–213. https://doi.org/10.1006/jmre.2000.2234.

(14) Lee, W.; Tonelli, M.; Markley, J. L. NMRFAM-SPARKY: Enhanced Software for Biomolecular NMR Spectroscopy. *Bioinformatics* **2015**, *31* (8), 1325–1327. https://doi.org/10.1093/bioinformatics/btu830.

(15) Zink, S.; Grosse, L.; Freikamp, A.; Bänfer, S.; Müksch, F.; Jacob, R. Tubulin Detyrosination Promotes Monolayer Formation and Apical Trafficking in Epithelial Cells. *J. Cell Sci.* **2012**, *125* (24), 5998–6008. https://doi.org/10.1242/jcs.109470.

(16) Yu, F.; Teo, G. C.; Kong, A. T.; Haynes, S. E.; Avtonomov, D. M.; Geiszler, D. J.; Nesvizhskii, A. I. Identification of Modified Peptides Using Localization-Aware Open Search. *Nat. Commun.* **2020**, *11* (1), 4065. https://doi.org/10.1038/s41467-020-17921-y.

(17) Kong, A. T.; Leprevost, F. V.; Avtonomov, D. M.; Mellacheruvu, D.; Nesvizhskii, A. I. MSFragger: Ultrafast and Comprehensive Peptide Identification in Mass Spectrometry-Based Proteomics. *Nat. Methods* **2017**, *14* (5), 513–520. https://doi.org/10.1038/nmeth.4256.

(18) Chang, H. Y.; Kong, A. T.; Da Veiga Leprevost, F.; Avtonomov, D. M.; Haynes, S. E.; Nesvizhskii, A. I.; Nesvizhskii, A. I. Crystal-C: A Computational Tool for Refinement of Open Search Results. *J. Proteome Res.* **2020**, *19* (6), 2511–2515. https://doi.org/10.1021/acs.jproteome.0c00119.

(19) Geiszler, D. J.; Kong, A. T.; Avtonomov, D. M.; Yu, F.; da Veiga Leprevost, F.; Nesvizhskii, A. I. PTM-Shepherd: Analysis and Summarization of Post-Translational and Chemical Modifications from Open Search Results. *Mol. Cell. Proteomics* **2021**, *20*, 100018. https://doi.org/10.1074/MCP.TIR120.002216.

(20) Yu, F.; Haynes, S. E.; Nesvizhskii, A. I. IonQuant Enables Accurate and Sensitive Label-Free Quantification with FDR-Controlled Match-between-Runs. *Mol. Cell. Proteomics* **2021**, *20*, 100077. https://doi.org/10.1016/J.MCPRO.2021.100077.

(21) Brademan, D. R.; Riley, N. M.; Kwiecien, N. W.; Coon, J. J. Interactive Peptide Spectral Annotator: A Versatile Web-Based Tool for Proteomic Applications. *Mol. Cell. Proteomics* **2019**, *18* (8), S193–S201. https://doi.org/10.1074/mcp.TIR118.001209.

(22) Schindelin, J.; Arganda-Carreras, I.; Frise, E.; Kaynig, V.; Longair, M.; Pietzsch, T.; Preibisch, S.; Rueden, C.; Saalfeld, S.; Schmid, B.; Tinevez, J.-Y.; White, D. J.; Hartenstein, V.; Eliceiri, K.; Tomancak, P.; Cardona, A. Fiji: An Open-Source Platform for Biological-Image Analysis. *Nat. Methods* **2012**, *9* (7), 676–682. https://doi.org/10.1038/nmeth.2019.

(23) Gerlach, M.; Schmitt, S.; Cyprys, P.; Kasper, M.-A.; Mai, I.; Klanova, M.; Maiser, A.; Leonhardt, H.; Hackenberger, C. P. R.; Fingerle-Rowson, G. R.; Vogl, A. M.; Schumacher, D.; Helma-Smets, J. TUB-010, a Novel Anti-CD30 Antibody-Drug Conjugate Based on Tub-Tag Technology, Widens the Therapeutic Window by Reducing Toxicity While Maintaining High Efficacy. *bioRxiv*. January 19, 2025, pp 1–19. https://doi.org/10.1101/2025.01.15.633119.
